# Synthesis and Development of Highly Selective Pyrrolo[2,3-*d*]pyrimidine CSF1R Inhibitors Targeting the Autoinhibited Form

**DOI:** 10.1021/acs.jmedchem.3c00428

**Published:** 2023-05-16

**Authors:** Thomas Ihle Aarhus, Frithjof Bjørnstad, Camilla Wolowczyk, Kristin Uhlving Larsen, Line Rognstad, Trygve Leithaug, Anke Unger, Peter Habenberger, Alexander Wolf, Geir Bjørkøy, Clare Pridans, Jan Eickhoff, Bert Klebl, Bård H. Hoff, Eirik Sundby

**Affiliations:** † Department of Materials Science & Engineering, Norwegian University of Science and Technology (NTNU), NO-7491 Trondheim, Norway; ‡ Department of Chemistry, Norwegian University of Science and Technology (NTNU), NO-7491 Trondheim, Norway; § Department of Biomedical Laboratory Science, Norwegian University of Science and Technology (NTNU), NO-7491 Trondheim, Norway; ∥ Skogmo IndustriomrÅde, Industrivegen 50, N-7863 Overhalla, Norway; ⊥ University of Edinburgh Centre for Inflammation Research, Queen’s Medical Research Institute, University of Edinburgh, Edinburgh EH16 4TJ, U.K.; # Lead Discovery Center GmbH, Otto-Hahn-Straße 15, 44227 Dortmund, Germany

## Abstract

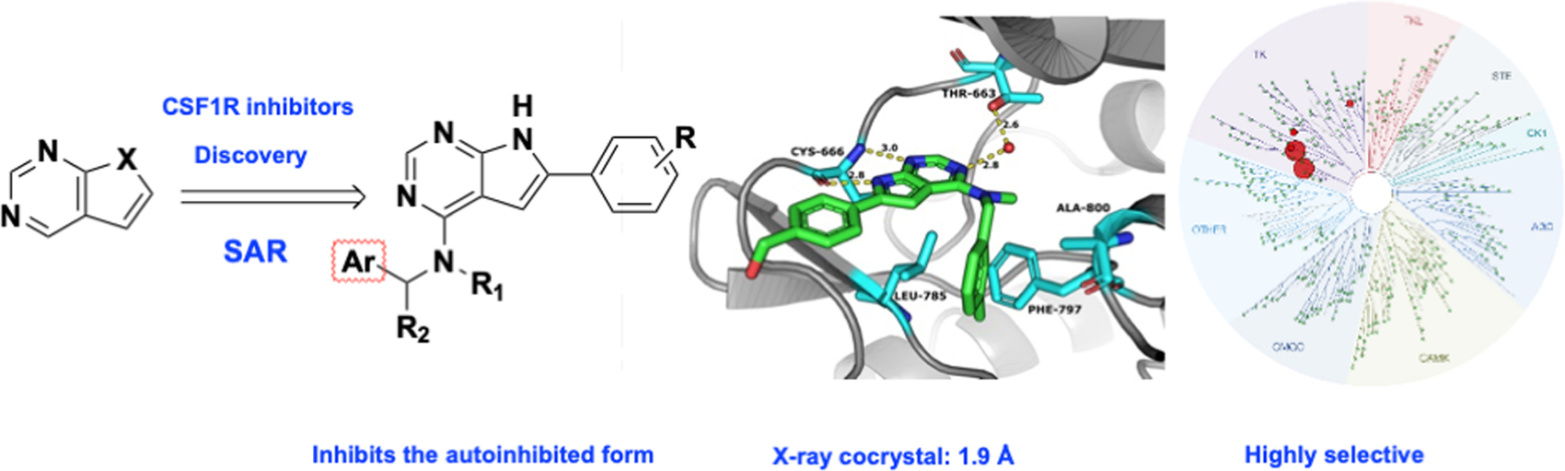

Colony-stimulating factor-1 receptor (CSF1R) is a receptor tyrosine kinase that controls the differentiation and maintenance of most tissue-resident macrophages, and the inhibition of CSF1R has been suggested as a possible therapy for a range of human disorders. Herein, we present the synthesis, development, and structure–activity relationship of a series of highly selective pyrrolo[2,3-*d*]pyrimidines, showing subnanomolar enzymatic inhibition of this receptor and with excellent selectivity toward other kinases in the platelet-derived growth factor receptor (PDGFR) family. The crystal structure of the protein and **23** revealed that the binding conformation of the protein is DFG-out-like. The most promising compounds in this series were profiled for cellular potency and subjected to pharmacokinetic profiling and *in vivo* stability, indicating that this compound class could be relevant in a potential disease setting. Additionally, these compounds inhibited primarily the autoinhibited form of the receptor, contrasting the behavior of pexidartinib, which could explain the exquisite selectivity of these structures.

## Introduction

The development of selective kinase inhibitors is a major challenge due to a large number of kinases and other adenosine triphosphate (ATP) binding proteins. A shared structural component in many kinases is the activation loop, whose conformation controls the catalytic activity and access to the substrate binding pocket.^1^ The movement of the activation loop is controlled by the phosphorylation state of the protein.^2^ Although these dynamic processes are likely to involve several intermediate states, the extreme outlier cases are the active state assuming a “DFG-in” conformation and an inactive state having a “DFG-out” structure. Inhibitors that preferentially bind to the “DFG-out” conformation have been named “type II” inhibitors, whereas “type I” inhibitors bind to the “DFG-in” conformation.^3^ Thus, an option for the development of selective kinase inhibitors is to design the antagonists so that they mainly bind the kinase in its inactivated state, where the conformations of the kinases are more likely to differ. One successful example includes the clinically approved drug imatinib, which inhibits the ABL kinase.^4^ However, Zhao et al.^5^ found that type II inhibitors do not necessarily have a selectivity advantage over type I inhibitors.

The colony-stimulating factor-1 receptor (CSF1R) is a tyrosine kinase embedded in the cell membrane of macrophages. The receptor is activated by colony-stimulating factor-1 (CSF-1) and interleukin-34, and signaling via CSF1R is crucial for the differentiation, proliferation, and survival of macrophages. Macrophages are part of the innate immune system and are essential components of the inflammatory microenvironment of diseased tissues.^6^ In cancers, a special class, termed tumor-associated macrophages (TAMs), engage in a complex interplay with tumor cells and other immune cells. Although macrophages have the potential to kill tumor cells, it has been found that TAMs can be drivers of tumor progression by deregulating effective T-cell responses. Targeting CSF1R to modulate macrophage populations may therefore result in therapeutic effects in several cancers.^7,8^ Among others, it has been shown that CSF1R signaling blockade in mouse models for breast cancer enhances the anticancer efficacy of platinum-based chemotherapeutics,^9^ BRAF inhibitors,^10^ and Src homology region 2 domain phosphatase inhibitors.^11^ Signaling via CSF1R also has a role in ovarian cancer progression.^12^ Besides cancers, pharmacological targeting of CSF1R might prevent the progression of neurodegenerative disorders by regulating microglial proliferation,^13^ and several investigational CSF1R inhibitors affect human osteoclasts, which indicate the potential for treating osteoporosis.^8^ The most advanced CSF1R-inhibiting drug entity is the low molecular weight inhibitor pexidartinib (Turalio), which has entered a number of clinical trials,^14^ and has been approved for use against tenosynovial giant-cell tumors.^15^

Unlike most kinases, CSF1R and other members of the platelet-derived growth factor receptor (PDGFR) family contain both an activation loop and a juxtamembrane domain (JM domain). In the autoinhibited form, the folding of the activation loop prevents the binding of ATP, and the JM domain forms bonding interactions with the DFG aspartate as well as other residues in the cleft between the N- and C-lobes of the protein, stabilizing an inactive form.^16,17^ Upon phosphorylation of the JM domain, its affinity for the binding site is lost, and an outward conformational movement of the JM domain occurs, opening up for ATP binding. The kinase is now in what is called the non-autoinhibited form, still with DFG-out conformation. ATP binding then triggers a more pronounced conformational change where the activation loop also moves out of the binding site, and the DFG motif is rotated to a DFG-in conformation. The kinase is now active and can accept and phosphorylate protein substrates. Further phosphorylation of the activation loop stabilizes the kinase domain in the locked non-autoinhibited form.

Herein, we report on a structure–activity study and metabolic profiling leading to the development of highly active and selective CSF1R inhibitors. The X-ray co-crystal structure of a representative inhibitor alongside binding assays shows the structures to preferably bind to the autoinhibited form of the kinase, whereas pexidartinib appears to have a higher affinity for the non-autoinhibited form in our assays.

## Results and Discussion

### Screening of In-House Library and Compound Design

**Scheme 1:**
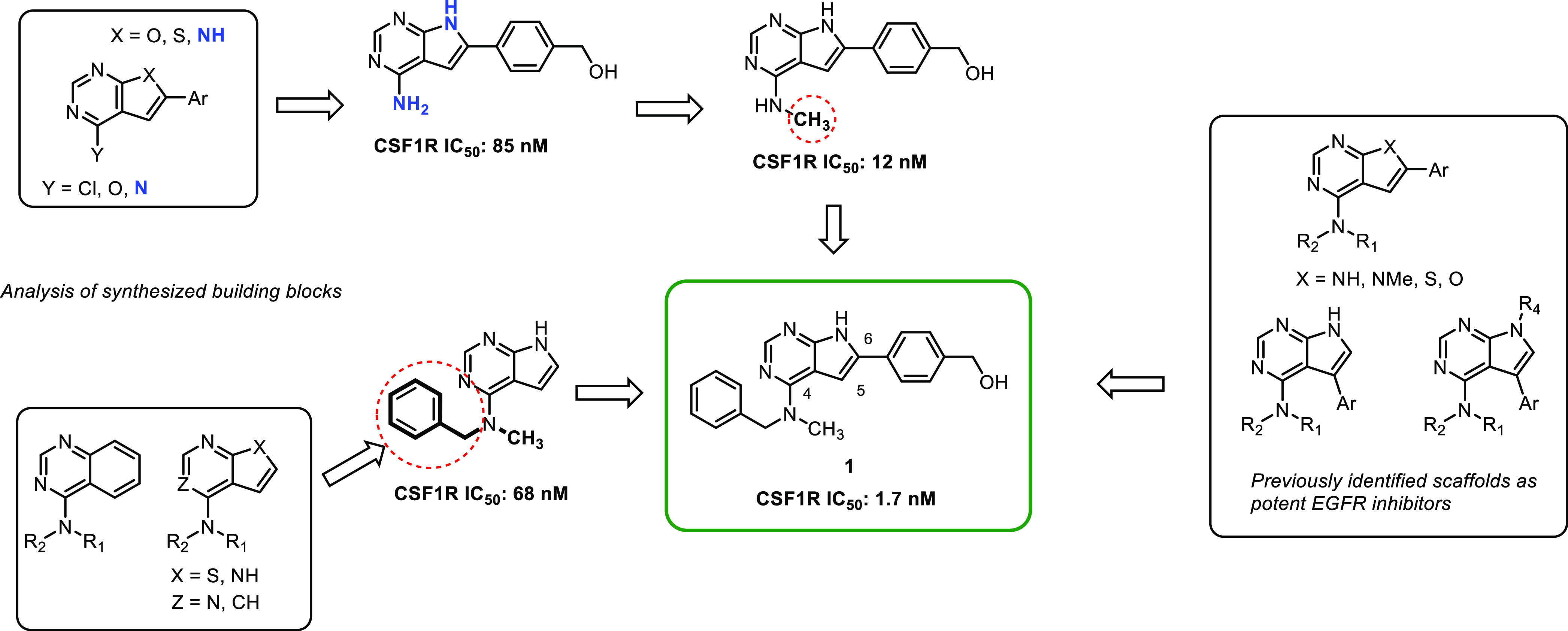
Preliminary Structure–Activity Relationship Identified by CSF1R and EGFR Screen

From previous work, we knew that some of our EGFR inhibitors also had CSF1R activity.^18^ In addition, we have recently reported benzyl-substituted pyrrolo[2,3-*d*]pyrimidines as active CSF1R inhibitors.^19^ Thus, our initial aim was to identify structural elements inducing CSF1R activity while at the same time reducing EGFR inhibitory potency. A screen was initiated with some previously described compounds^18,20−22^ and new materials (see the Supporting Information). The major findings are described in Scheme 1.

Activity testing of some low molecular weight quinazolines and thieno-, pyrrolo-, and furopyrimidines revealed that the CSF1R activity was highly dependent on the NH pyrrole unit of the pyrrolopyrimidines (Scheme 1, left-hand side). From previous studies on other pyrrolopyrimidines^23^ and thienopyrimidines,^22^ we knew that methylating the N-4 nitrogen reduced EGFR activity. Encouragingly, this was also the case for this compound class, and with the bonus of increased CSF1R activity. The evaluation of other fused pyrimidines (Scheme 1, right-hand side) also concluded that 6-arylated pyrrolopyrimidines were the preferred scaffold. In contrast, 5-arylated pyrrolopyrimidines had low activity. The initial study showed that including a polar *para-*substituent increased the CSF1R activity. The prototypic inhibitor **1** had an enzymatic CSF1R IC_50_ of 1 nM and an EGFR IC_50_ of 20 nM.

### Structure–Activity Relationship

Proceeding from our initial hit, compound **1**, we wanted to improve the CSF1R inhibitory properties while at the same time maintaining a low EGFR activity (Table 1). Pexidartinib and Erlotinib were used as positive controls having IC_50_ values of 9.7 and 0.4 nM toward CSF1R and EGFR, respectively.

**Table 1:**
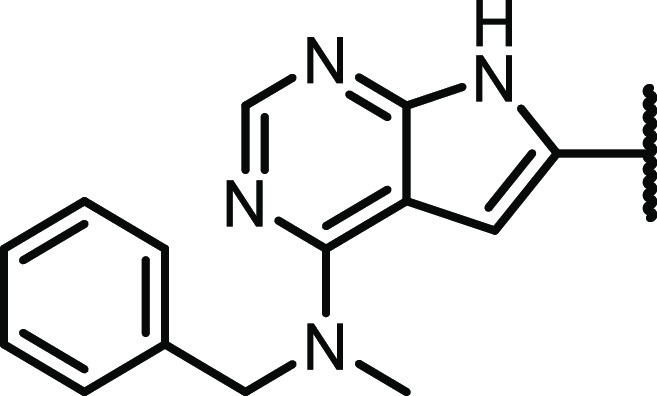

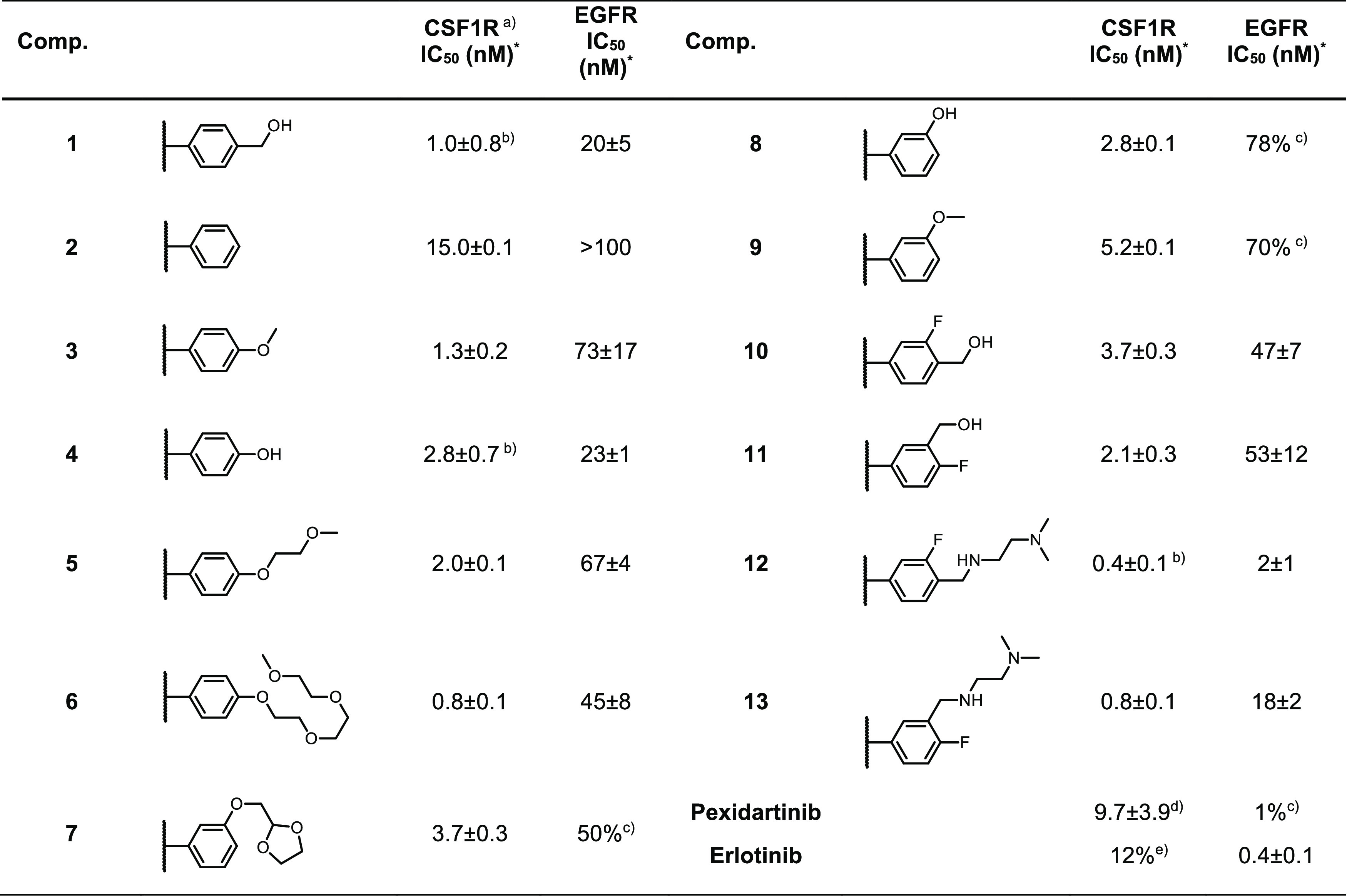
Effect of Varying the 6-Aryl Group (Compounds **1–13**) on CSF1R and EGFR Inhibitory Activity

a *Unless otherwise noted, the assays are based on two titrations (20 data points). The ATP level was equal to *K*_M_ in both cases. ^a^Protein construct containing amino acid residues 530–910.

b IC_50_ values were based on four titration curves (40 data points).

c Inhibition (%) at 100 nM test concentration, an average of duplicate measurements.

d Average based on 10 titration curves (100 data points).

e Inhibition (%) at 500 nM test concentration, an average of duplicate measurements.

We first evaluated thirteen different 6-aryl groups, see Table 1. Except for the naked phenyl derivative **2**, most of the compounds were highly potent toward CSF1R with IC_50_ values < 5 nM and with relatively low EGFR activity. Considering previous results from our group,^21^ the data demonstrates that methylation of the 4-amino group effectively reduces EGFR activity. One exception was the ethylenediamine-containing compound **12** with an EGFR IC_50_ of 2.3 nM. We have also previously noted that this group increases EGFR activity.^21^ We theorize that the observed increase in EGFR activity for **12** can be attributed to favorable salt-bridge interactions between the ethylenediamine group and the acidic residues Asp-800 and/or Glu-804 of the EGFR protein. The m-substituted analogue **13** would likely not be able to form a strong salt bridge to Glu-804 due to the large distance between its side chain and the hinge region.

We then went on to evaluate the effect of different amines at C-4, retaining the 6-aryl substituent as *p*-hydroxymethyl, see Table 2. The CSF1R activity was quite tolerant to small variations in this part of the structure. However, *N*-isopropyl substituted **16**, the *p*-*t*-butyl derivative **19**, and introducing an amide (**20**) abolish most of the activity. The deutero analogue of the initial hit **1**, compound **21**, was synthesized for later investigation of metabolic stability.

**Table 2:**
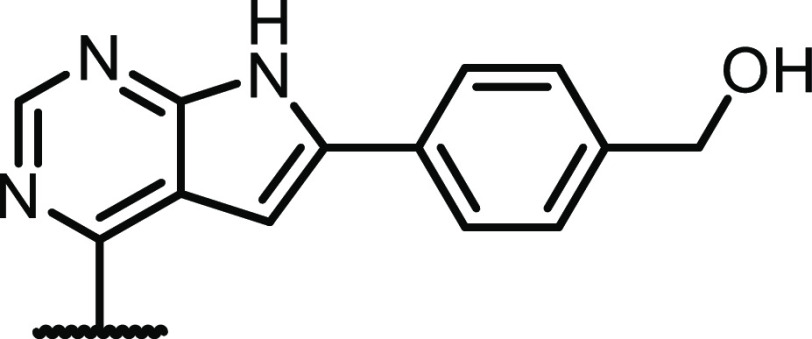

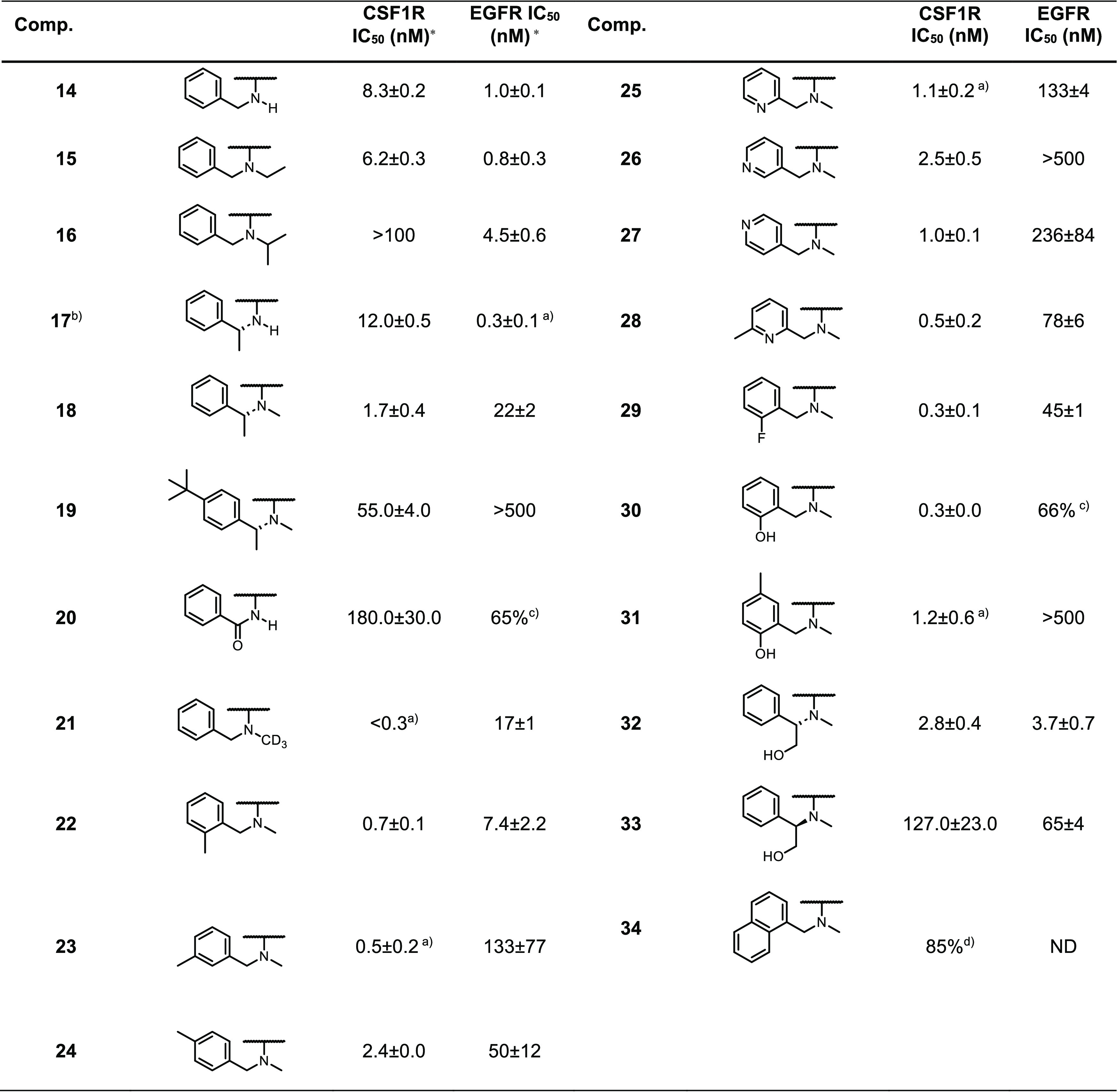
Effect of Varying the 4-Amino Group (Compounds **14–34**) on CSF1R and EGFR Inhibitory Activity

a *Unless otherwise noted, the assays are based on two titrations (20 data points). The ATP level was equal to *K*_M_ in both cases. ^a^IC_50_ values were based on four titration curves (40 data points).

b Previously identified EGFR inhibitor.^18^

c Inhibition (%) at 100 nM test concentration, an average of duplicate measurements.

d Inhibition (%) at 500 nM test concentration, an average of duplicate measurements.

One of the more active compounds was the *m*-methyl derivative **23**. *O*-methylated analogue **22** was also highly active but suffered from low solubility. All of the four pyridyl substituted derivatives (compounds **25**–**28**) possessed good activity toward CSF1R and a very low potency toward EGFR. Moreover, the pyridyl fragment likely provides better solubility, and exchanging carbo-aromatic with heteroaromatic rings is generally beneficial for absorption, distribution, metabolism, and excretion (ADME) properties.^24^ Excellent enzymatic potency was also seen for the *o-*fluoro **29** and *o-*hydroxy **30** analogues, although it is important to mention that compound **30** as well as compound **31** are phenolic Mannich bases, a structural motif known to cause pan-assay interference and cytotoxicity.^25^ It is also worth noting that the introduction of a hydroxymethyl group in the benzylic position retains the enzymatic activity for the (*S*)-enantiomer but loses most of the activity for the (*R*)-enantiomer (**32** vs **33**).

In addition to further improving CSF1R activity, the introduction of an *m*-methyl group on the benzylamine moiety also significantly suppressed EGFR activity (compound **1** vs **23**). Impressed by this apparent boost in selectivity, a new series of inhibitors was made employing the core of compound **23** as a starting point. At this stage, metabolic profiling in rat and mouse liver microsomes indicated that the benzylic alcohol was a metabolic soft spot. We therefore made analogous compounds containing alternative substituents in the 6-aryl part, see Table 3.

**Table 3:**
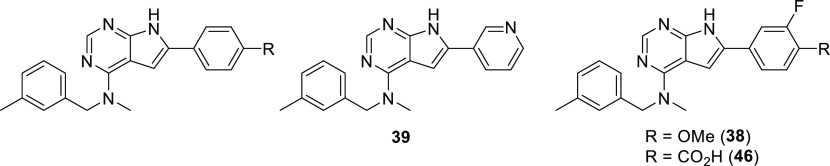
Effect of Varying the 6-Aryl Group on CSF1R and EGFR Inhibitory Activity

		enzymatic IC_50_ values (nM)			
comp.	R	CSF1R (Z′-LYTE)^a^	CSF1R IC_50_^b^ LANCE (ATP: 25 μM)	CSF1R IC_50_^c^ LANCE (ATP: 2500 μM)	EGFR IC_50_^a^ (Z′-LYTE)^a^
**Pexidartinib**	-	5	20	35	0%^e^
**23**	–CH_2_OH	0.5 ± 0.2	<3	-	118 ± 52
**35**	–H	1.8 ± 0.1	ND^d^	17	>1000
**36**	–OH	1.4 ± 0.1	10	16	745 ± 130
**37**	–F	2.7 ± 0.0	ND	29	>1000
**38**	–OMe (3-F)	1.4 ± 0.0	18	53	11%^e^
**39**	-	0.9 ± 0.2^f^	<3	10	413 ± 57
**40**	O(CH_2_CH_2_O)_3_CH_3_	0.4 ± 0.3	ND	ND	117 ± 3
**41**	–CF_3_	4.9 ± 0.2	274	540	3%^e^
**42**	–CO_2_Me	4.0 ± 0.2	253	404	4%^e^
**43**	–(CH_2_)_4_CO_2_Me	7.7 ± 2.0^f^	104	ND	0%^e^
**44**	–CO(CH_2_)_5_CO_2_Me	11 ± 4^f^	ND	ND	0%^e^
**45**	–CO_2_H	0.3 ± 0.1^g^	<3	<3	53 ± 3
**46**	–CO_2_H (3-F)	0.4 ± 0.0	<3	<3	62%^e^
**47**	–(CH_2_)_2_CO_2_H	<0.3	ND	ND	30 ± 10
**48**	–(CH_2_)_4_CO_2_H	0.4 ± 0.2^f^	<3	ND	71%^e^
**49**	*p*-CHF_2_	3.0 ± 0.3	29	55	>1000
**50**	*p*-SO_2_NH_2_	0.4 ± 0.0	<3	<3	98 ± 40
**51**	*m*-CO_2_Me	6.4 ± 0.5	30	80	>1000
**52**	*m*-CO_2_H	0.6 ± 0.1	<3	7	127

a *The assays are based on two titrations (20 data points) unless otherwise noted. The ATP level was equal to *K*_M_ in both cases. ^a^IC_50_ (nM) measured by Z′-LYTE assay technology (Thermo Fisher).^26^ The ATP level was equal to *K*_M_ 10 μM. Unless otherwise noted, IC_50_ values are based on two titration curves (20 data points).

b IC_50_ (nM) measured LANCE *Ultra* assay (Perkin Elmer). The ATP level was equal to *K*_M_ (25 μM).

c IC_50_ (nM) measured LANCE *Ultra* assay (Perkin Elmer). ATP level: 2500 μM.

d ND = Not determined.

e Percent inhibition at 100 nM test concentration.

f Based on 4 titration curves (40 data points).

g Based on 6 titration curves (60 data points).

All of the unsubstituted phenyl derivative **35**, *p*-fluoro **36**, *p*-hydroxyl **37**, and 4-methoxy-3-fluorophenyl **38** possessed very good CSF1R activity. The 3-pyridyl derivative **39** and the ethylene ether **40**, designed as more soluble derivatives, were more active, while compounds containing lipophilic substituents at the *para* position, the trifluoromethyl **41** and the esters **42–44**, experienced a moderate drop in activity. All of the carboxylic acids **45–48** showed a very low CSF1R enzymatic IC_50_. It was later shown that the benzoic acid **45** is the main metabolite of the benzyl alcohol functionalized **23** in mice.

Selected derivatives were also assayed in an alternative CSF1R enzymatic assay (LANCE) using an ATP concentration of 25 and 2500 μM. The most potent compounds in the Z′-LYTE primary assay were also found to be highly active in the alternative LANCE assay, even at high ATP concentrations, albeit at around a factor of 10 higher IC_50_ values. This includes compounds **36**, **39**, **45**, and **46**. In contrast, the trifluoro- and methyl ester analogues **41** and **42** displayed especially high IC_50_ values in the LANCE assays.

### Protein Crystal and Molecular Modeling

**Figure 1:**
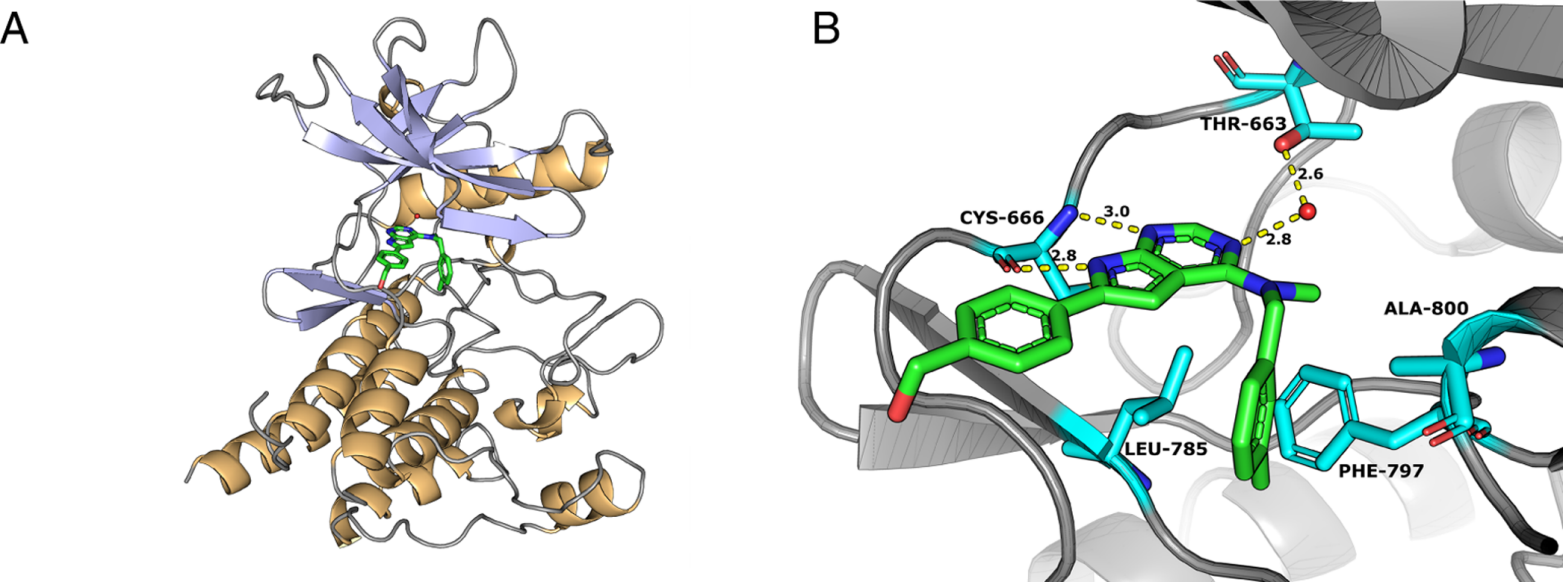
(A) Co-crystal structure of inhibitor **23** (green) with CSF1R at 1.9 Å resolution (PDB 8CGC). (B) Binding mode of inhibitor **23** (green) with CSF1R (PDB: 8CGC). CSF1R is shown in gray ribbons with selected residues colored cyan. Hydrogen bonds are drawn as yellow dashed lines.

A protein co-crystal of CSF1R in complex with **23** was obtained using a sitting-drop vapor diffusion set-up. The construct used for the crystallization was that of a published structure (PDB entry 4HW7) bearing the mutation S688A and the deletion of residues 696 to 741. A complete 1.9 Å data set of the CSF1R/**23** crystal was collected at the ESRF synchrotron radiation source (Grenoble, FR, beamline ID29). The structure is shown in Figure 1.

Inhibitor **23** binds via two hydrogen bonds to Cys-666 in the hinge backbone of the kinase: the pyrimidine N1-atom accepts a hydrogen bond from the NH of Cys-666, while the pyrrole NH donates a hydrogen bond to the Cys-666 carbonyl oxygen. The latter interaction likely explains why similar thieno- and furopyrimidines (see the Supporting Information) possess low inhibitory potency. Additionally, a water-mediated hydrogen bond is formed between atom N3 of the pyrimidine moiety and Thr663. The methylbenzene ring points toward the C-terminal subdomain. It is sandwiched between Leu-785 and Ala-800 and almost perpendicular to the side chain of Phe-797 from the DFG motif, which adopts the “DFG-out” conformation. The phenylmethanol moiety is oriented toward the protein surface, with the OH group possessing a higher degree of flexibility and thus higher B-values than the rest of the molecule.

Although the inhibitor binds to CSF1R in the DFG-out conformation, the ligand does not occupy the allosteric pocket that is formed underneath the αC-helix when the kinase adopts this inactive conformation, as is typically the case with type II inhibitors. Comparing the crystal structure to a traditional DFG-out, type II inhibitor–protein complex (PDB: 4R7H, CSF1R–pexidartinib) reveals that the protein backbone of the DFG-Asp residue is positioned closer to the αC-helix (7.7 vs 10.1 Å), significantly reducing the size and accessibility of this binding cleft. This type of kinase conformation has been referred to as DFG-out-like.^27^ Instead, the 3-methylbenzylamine moiety of compound **23** occupies a hydrophobic binding cleft formed by residues Leu-785, Ala-800, and Phe-797 termed the front pocket.^28^ The methyl group of the aromatic ring points toward the C-lobe of the kinase into a small complementary cavity in the front binding cleft. Thus, compound **23** cannot be classified as a typical type II inhibitor as its binding mode resembles that of a Type I inhibitor.

With the crystal structure of **23** in hand, we performed *in silico* docking of compounds **23**–**52** using Glide.^29^ All of the top-scored conformations displayed binding modes almost identical to the crystallized structure.

### Kinase Selectivity

The X-ray co-crystal structure of **23** showed the compound to bind to the DFG-out inactive conformation of the kinase. To further evaluate the binding mode, a selection of compounds was subjected to binding assays toward two versions of the CSF1R kinase domain developed to mimic the non-autoinhibited and autoinhibited forms, respectively. The assay is a competition binding assay that quantitatively measures the ability of a compound to compete with an immobilized, active site-directed ligand. The data are compared with the IC_50_ values in Table 4. Interestingly, all nine new pyrrolopyrimidines preferably bind to the protein representing the autoinhibited form of CSF1R. In some cases, a more than 50-fold affinity difference between the autoinhibited and non-autoinhibited states of the kinase is observed, far outside the range of affinity shifts reported for type II TKIs.^17^ In addition, the reference compound pexidartinib shows considerably higher binding toward the non-autoinhibited form of the receptor in our assays, contrasting previous reports for the protein in a crystallized state.^30^ A carboxylic acid substituent at C-6 seems to favor binding to the autoinhibited form (compounds **45** and **47**) while introducing an α-methyl group on the benzylamine substituent as the (*R*)-stereoisomer appears to strengthen binding to the non-autoinhibited variant (compound **18**). The consequences of conformationally selective CSF1R inhibitors in a therapeutic setting are unclear. However, one might speculate that a specific conformation leads to improved potency if the conformation has a lower affinity to ATP. Kinase selectivity might also improve, given that unique pockets could be formed in a given conformation. On the other hand, the unactivated state of CSF1R might not be present in the diseased tissue. Even so, several approved kinase inhibitor drugs act by binding to an unactivated state of the kinase (sorafenib, imatinib, lapatinib, sunitinib, nilotinib, and others), which points to the usefulness of this strategy.

**Table 4:**
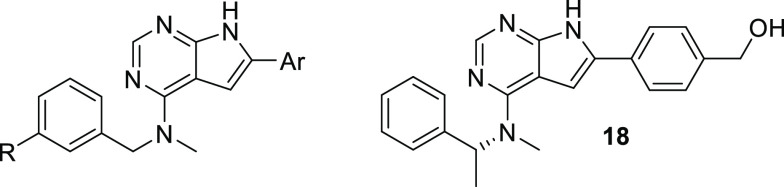
Binding Assays (*K*_d_, nM) toward the Non-Autoinhibited and the Autoinhibited Form of CSF1R

compound	R	Ar	CSF1R (IC_50_, nM)^a^	binding assay non-autoinhibited CSF1R (*K*_d_, nM)^b^	binding assay autoinhibited CSF1R (*K*_d_, nM)^c^
**2**	H	Ph	15.0	>1000	52
**4**	H	*p*-C_6_H_4_OH	2.6	410	6.1
**8**	H	*m*-C_6_H_4_OH	2.8	>1000	72
**18**	d	d	1.7	53	38
**23**	CH_3_	*p*-C_6_H_4_CH_2_OH	0.5	320	26
**36**	CH_3_	*p*-C_6_H_4_OH	1.4	>1000	7.9
**39**	CH_3_	3-Pyridyl	0.9	340	6.8
**45**	CH_3_	*p*-C_6_H_4_CO_2_H	0.3	170	2.3
**47**	CH_3_	*p*-C_6_H_4_CH_2_CH_2_CO_2_H	<0.3	130	7.2
**Pexidartinib**			9.7	5.8	360

a Assay performed by Thermo Fisher using the Z′-LYTE technology. CSF1R kinase containing the amino acid fragment: 538–910, the ATP level was equal to *K*_M_.

b Binding assay by Eurofins, the model of the non-autoinhibited form of the CSF1R kinase, containing the amino acid fragment: 564–939.

c Binding assay by Eurofins, the model of the autoinhibited form of the CSF1R kinase, containing the amino acid fragment: 538–939.

d See the structure of the amine part above the table.

**Figure 2:**
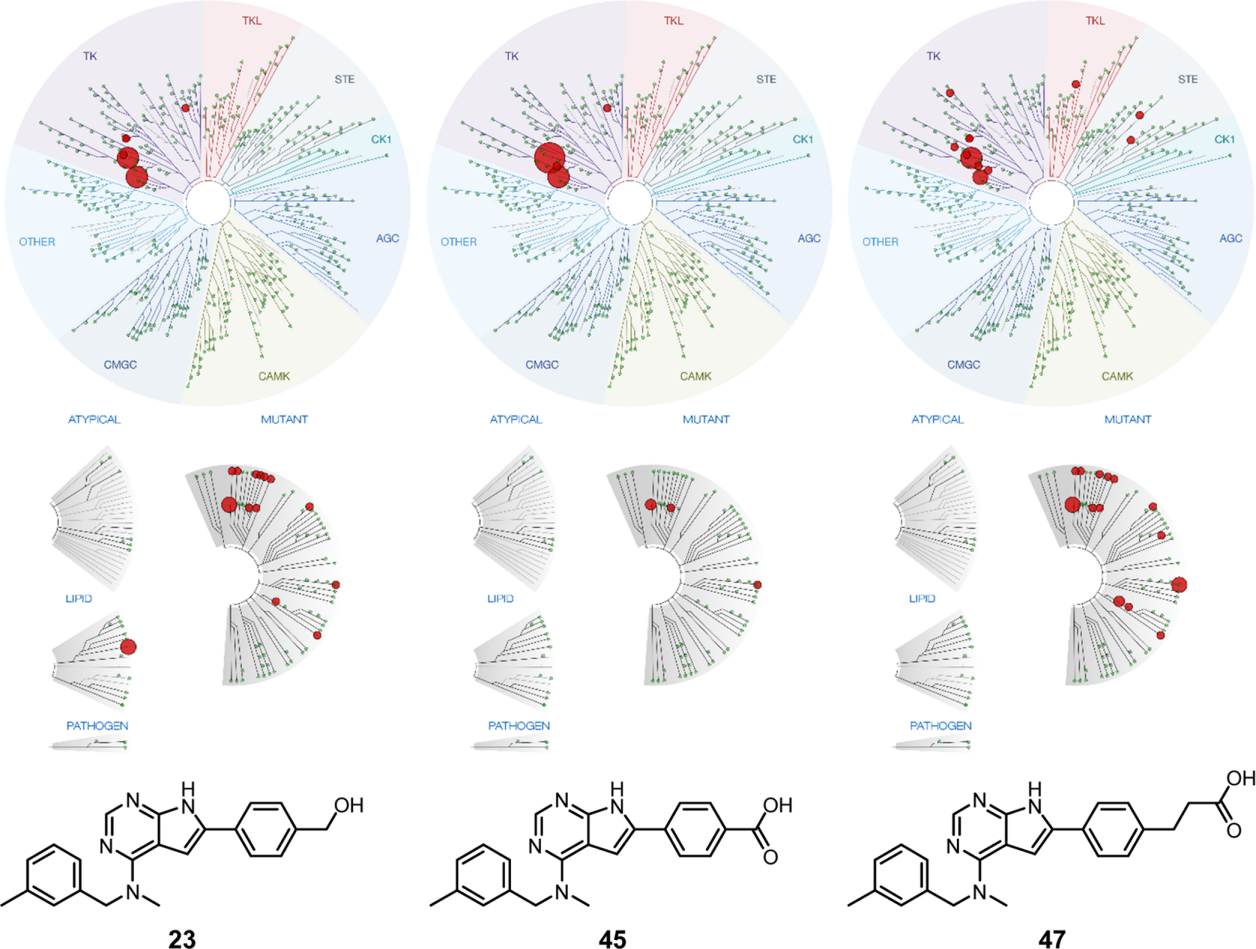
Selectivity profiling of compounds **23** (left), **45** (middle), and **47** (right) against 489 kinases at 500 nM test concentration (larger spheres indicate higher potency).

Three of the inhibitors, the benzyl alcohol **23**, the benzoic acid **45**, and the propanoic acid **47** were also assayed in a panel of 468 kinases. The assay is an active site-directed competition binding assay to quantitatively measure interactions between test compounds and relevant human kinases. The assays do not require ATP and thus report true thermodynamic interaction affinities. Compounds that bind to the kinase active site and directly or indirectly prevent kinases from binding to the immobilized ligand will reduce the amount of kinase captured on the solid support giving a low recovery of kinase. Conversely, test molecules that do not bind the kinase have no effect on the amount of kinase captured on the solid support, and a high recovery of kinase will be monitored. The data is illustrated as plotted kinome trees in Figure 2.

An off-target kinase for the three inhibitors was ephrin type-B receptor 6 (EPHB6). As EPHB6 lacks kinase activity, it is unknown if the CSF1R inhibitors will affect its function. Ephrin-type receptors have generally been recognized for their role in immune cell development.^31^ Elevated expression levels of EPHB6 are seen in some cancers,^32,33^ and although being tumor-promoting, higher sensitivity toward chemotherapeutics has been observed.^33^ A clear difference between the three compounds was that **23** was found to bind strongly to the catalytic subunit α of phosphatidylinositol-4,5-bisphosphate 3-kinase (PI3CA), whereas this was not the case for the carboxylic acid derivatives **45** and **47**. Inhibition of PI3CA is of relevance in hormone receptor-positive, HER2-negative subtype of breast cancer, but inhibition is also linked to adverse effects such as hyperglycemia and liver toxicity.^34^ Two mutant kinases, namely, ABL1(H396P)-nonphosphorylated and FLT3(D835V) were also effectively inhibited while leaving the wild-type kinases untouched. The inhibitors displayed high selectivity for CSF1R over other members of the PDGFR III kinase family. The selectivity score^35^ (S-score), using 50% inhibition as a threshold, showed **45** to be the most selective (S-score: 0.05), followed by **23** (S-score: 0.07) and **47** (S-score 0.09). Overall, the compounds tested in this assay displayed remarkably high selectivity toward CSF1R.

**Figure 3:**
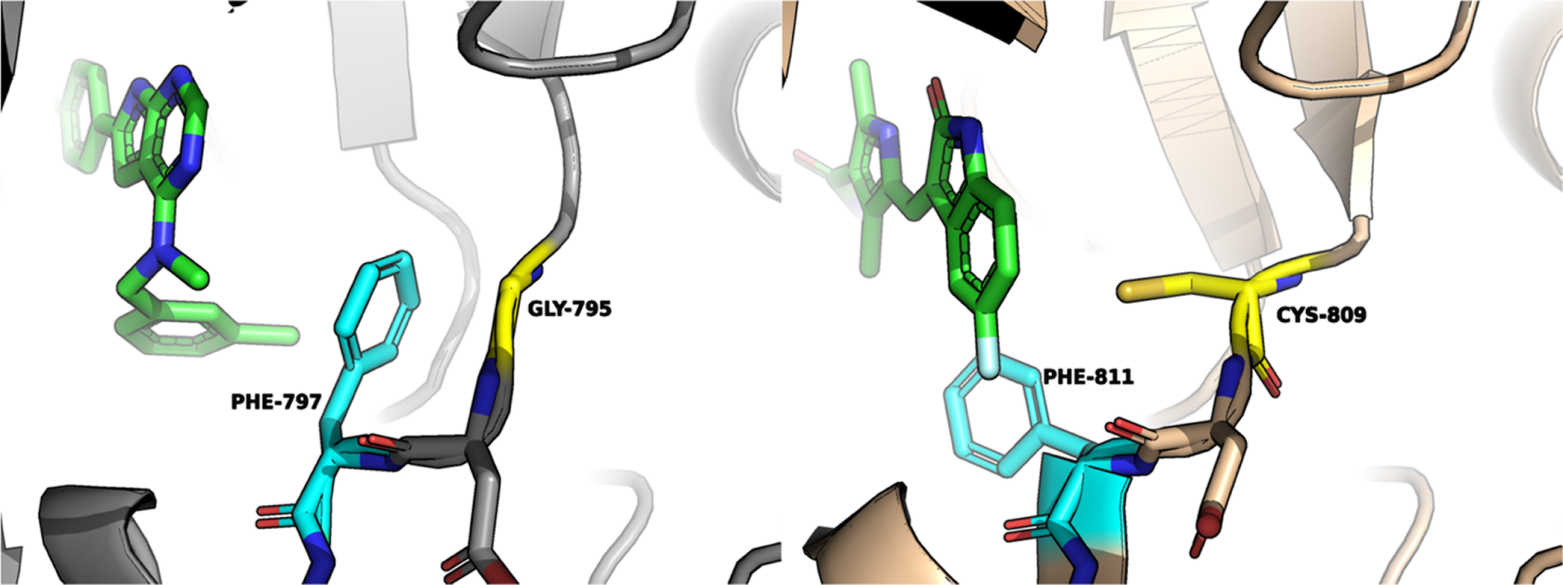
Comparison between **23** in complex with CSF1R (left) and sunitinib in complex with KIT (right, PDB-ID 3G0E(36)). The side chain of Phe-797 folds toward Gly795 in the CSF1R complex, while the Cys-809 residue precludes an analogous folding of the corresponding Phe811 side chain in KIT.

**Figure 4:**
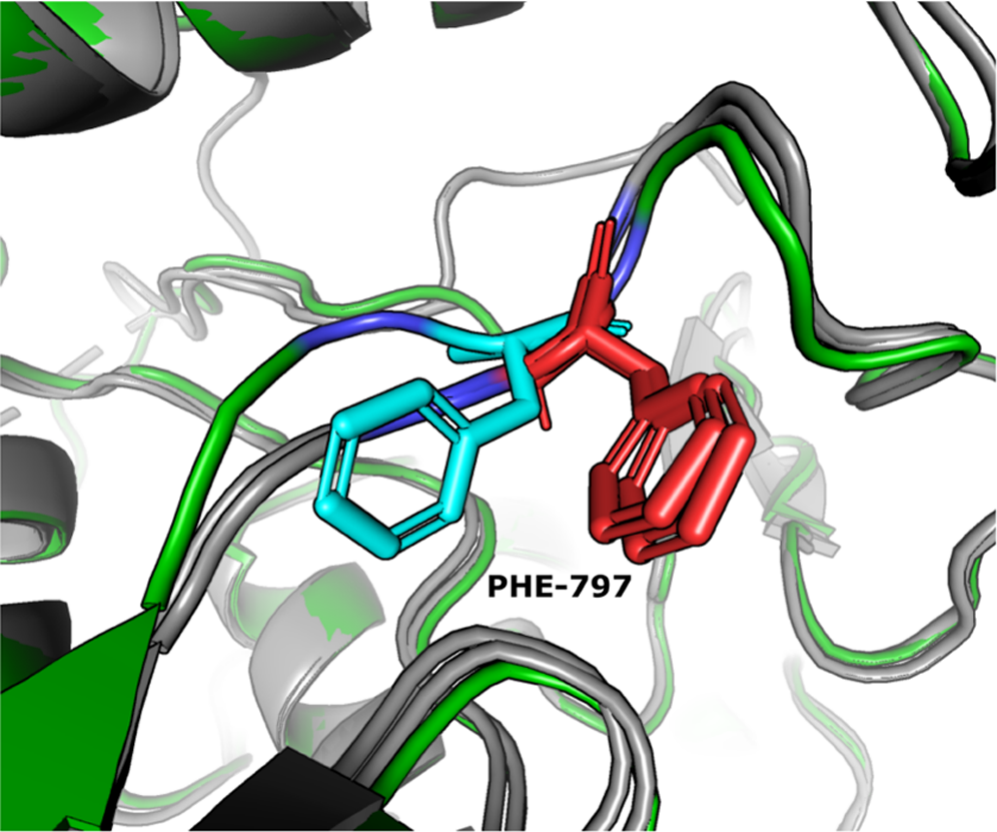
Overlay of various CSF1R DFG motifs using proteins crystallized with pexidartinib, acyl urea (PDB: 4R7H and 7TNH, respectively), and **23** (inhibitors not shown), clearly displaying the unique binding mode of our inhibitors. 4R7H and 7TNH are displayed in gray ribbons with Phe-797 highlighted in red, while our protein is displayed with green ribbons and Phe-797 highlighted in cyan. The DFG motif is colored blue for all proteins.

Selectivity toward one particular kinase in the PDGFR family of enzymes is often difficult to achieve because of the high structural similarity they share.^37^ One distinct feature of CSF1R, however, is the absence of a cysteine residue immediately preceding the DFG motif that is present in the other enzymes of the family.^38^ In CSF1R, this residue is replaced with a smaller glycine unit. Although our inhibitors do not appear to occupy the same space as the cysteine residue in computer models, the proximity of the cysteine side chain to the phenylalanine side chain of the DFG motif would likely prevent free rotation of the benzyl moiety of the phenylalanine residue. The co-crystal structure of CSF1R and **23** reveals that the benzyl group of Phe-797 is folded toward the glycine residue (Figure 3). It is hypothesized that, for our inhibitors, the folded position of the phenylalanine side chain is a crucial feature of the inhibitor-bound protein conformation and that restriction of its movement due to the presence of a bulkier cysteine residue as seen in KIT, FLT3, PDGFRα, and β results in weaker affinities toward these enzymes. Furthermore, when superimposing CSF1R proteins crystallized with various inhibitors (exemplified in Figure 4), it becomes immediately obvious that the movement of Phe-797 is substantial when bound to our pyrrolopyrimidines, giving our inhibitors a unique binding mode to this receptor.

### ADME, Cell Profiling, and *In Vivo* Pharmacokinetics

The identified inhibitor structures contain potential metabolic soft spots, and we expected that metabolic stability could be a main hurdle. Profiling of selected CSF1R inhibitors toward human, mice, and rat liver microsomes, mice phase II metabolism, plasma stability, and protein binding are compared with data for pexidartinib in Table 5.

**Table 5:**
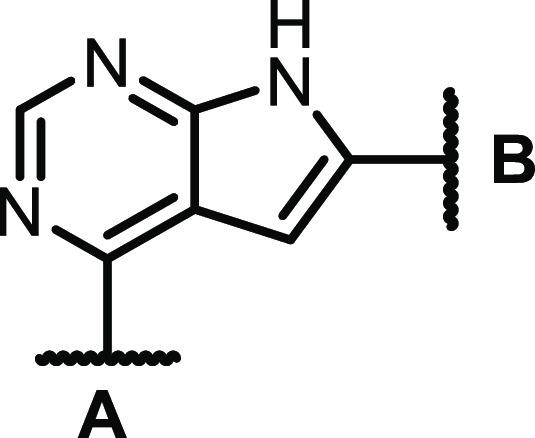

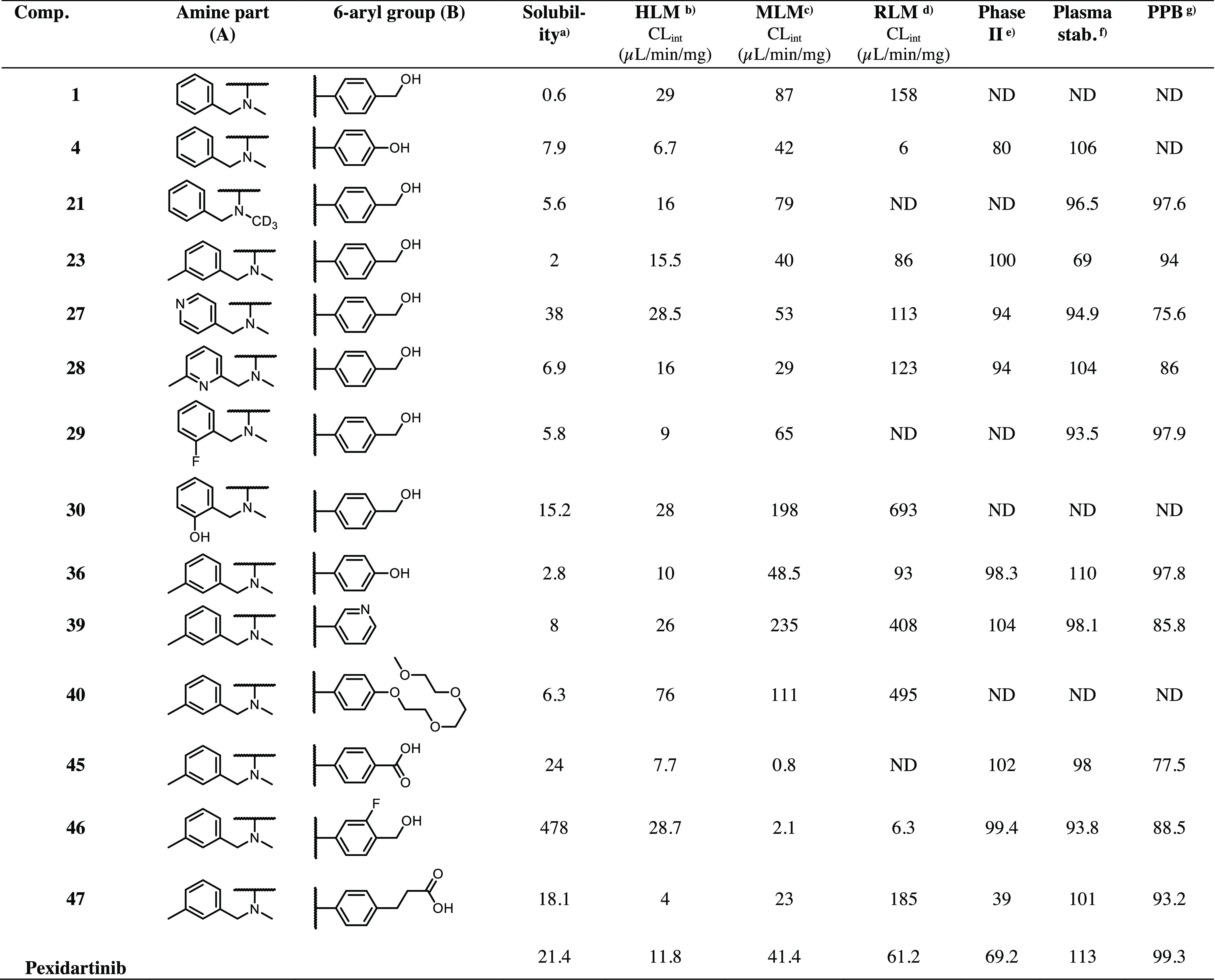
Stability Profiling of Selected CSF1R Inhibitors toward Human, Mice, and Rat Liver Microsomes, Mice Phase II Metabolism, Plasma Stability, and Protein Binding

a Kinetic solubility determined spectrophotometrically at pH 7.4.

b *In vitro* phase I metabolism in human liver microsomes (HLM).

c *In vitro* phase I metabolism in mice liver microsomes (MLM).

d *In vitro* phase I metabolism in mice liver microsomes (MLM).

e Microsomal stability phase II mouse (% remaining at 5.0 μM).

f Plasma stability mouse (% remaining at 5.0 μM).

g Plasma protein binding mouse (% bound at 5.0 μM).

When the benzyl alcohol-containing derivatives were analyzed, it was apparent that although some derivatives had reasonable stability toward human liver microsomes (HLM), they had a high clearance in mice and rat liver microsomes. This was highly undesirable for subsequent *in vivo* experiments. As the observed high clearance values were suspected to be caused by *N*-demethylation, the deuterated analogue **21** was synthesized. Although clearance improved twofold in HLM, high metabolism was still observed in MLM, suggesting that an additional metabolic pathway also played a role. Mass spectroscopic analysis of liver microsome samples proved the major metabolite of **23** to be the benzoic acid **45**. This finding led us to abandon all benzyl alcohols in subsequent studies (See the Supporting Information).

The phenol **36** had stability in HLM and MLM assays comparable to that seen for pexidartinib. Phase II metabolism could be an obstacle for such ligands,^39^ but this was not seen in the mice phase II assay. The introduction of pyridines is a common method to increase solubility and reduce CYP-mediated oxidation. However, inserting a 3-pyridyl group at C-6 resulted in rapid metabolism in MLM and RLM of compound **39**, probably due to aldehyde oxidase-mediated oxidation of the pyridyl ring.^40^ Also, the PEGylated derivative **40** was highly unstable. The benzoic acid **45**, the main metabolite of **23**, showed high stability toward both HLM and MLM and was a good candidate for further development. Surprisingly, the corresponding fluorinated version **46** was found to be more unstable. Finally, phenylpropanoic acid **47** also appeared to be stable enough for further development.

To assess the pharmacokinetic properties of the compounds *in vivo*, a cassette dosing study in mice was carried out. The data is listed in Table 6. The inhibitors exhibited low drug exposure in plasma, at only a fraction of the reference pexidartinib. The benzoic acid **45** displayed the highest exposures and most promising pharmacokinetic (PK) parameters, in line with the previously determined MLM clearance value for this compound.

**Table 6:**
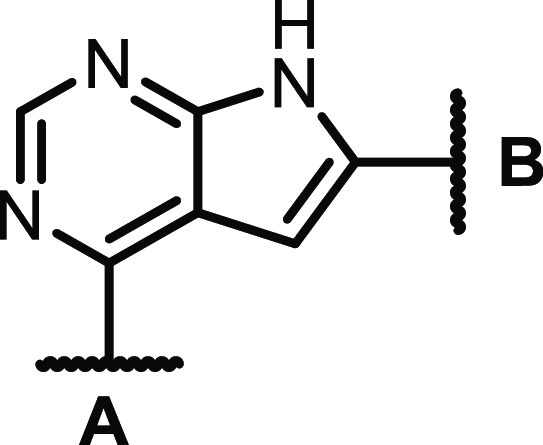

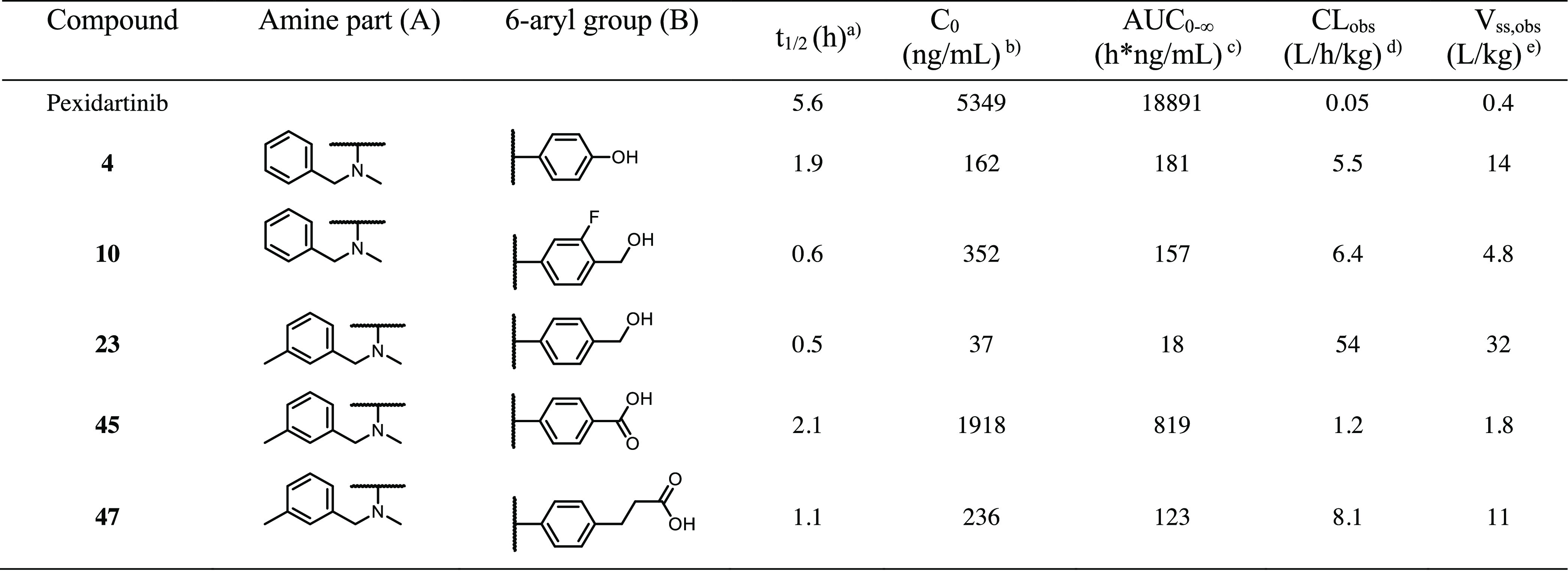
Determination of Pharmacokinetic Parameters of Selected CSF1R Inhibitors *In Vivo*

a *In vivo* half-life in C57BLKS, female mice (*n* = 3) by IV (1 mg/kg).

b Initial concentration *in vivo* using C57BLKS, female mice (*n* = 3) by IV (1 mg/kg).

c The total area under the curve in C57BLKS, female mice (*n* = 3) by IV (1 mg/kg).

d *In vivo* clearance in C57BLKS, female mice (*n* = 3) by IV (1 mg/kg).

e *In vivo* steady-state volume of distribution in C57BLKS, female mice (*n* = 3) by IV (1 mg/kg).

f The experiment was performed as a cassette dosing study at a dose of 1 mg/kg per compound intravenously in mice.

To ensure that the simultaneous exposure to multiple compounds in a cassette dosing study did not cause interference, we also performed single compound PK studies for selected compounds and observed comparable ratios of the half-life for pexidartinib and **4**, respectively (data not shown).

### Cell-Based Assays

Selected compounds were also profiled in two cell-based assays: Ba/F3 cells engineered to be dependent on CSF1R activation and bone marrow-derived macrophages from mice, and the data are presented in Table 7.

**Table 7:**
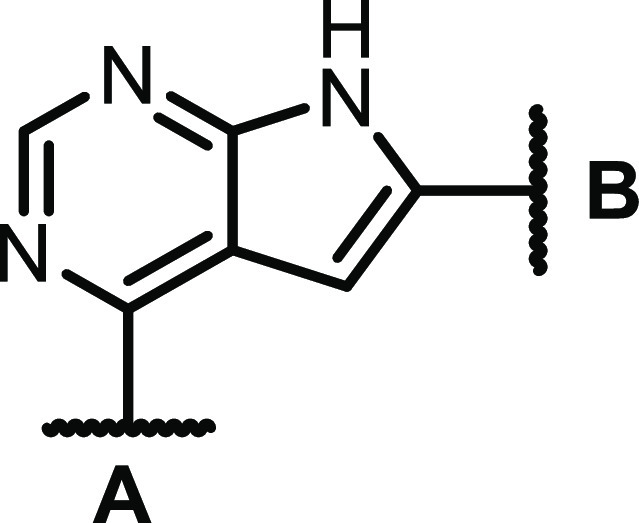

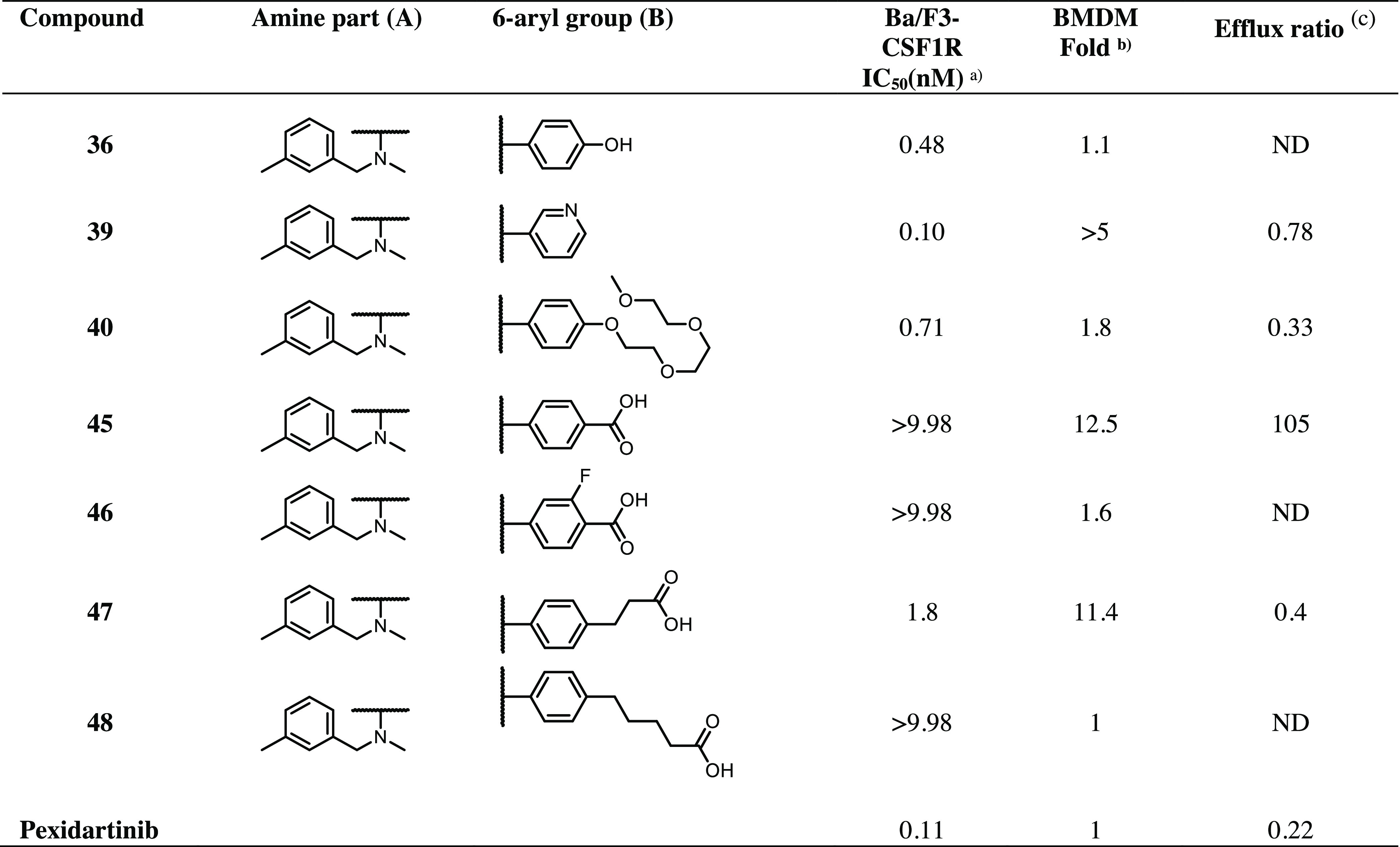
Cellular Potency of Selected Compounds toward Genetically Modified Ba/F3 cells, Bone Marrow-Derived Macrophages from Mice, Osteoclast Differentiation, and the Corresponding Caco2–Efflux Ratio

a IC_50_ values for the proliferation of a Ba/F3-CSF1R cell line.

b Bone marrow-derived macrophages (BMDM) isolated from mice measured for phosphorylated MAPK. The experiment was run at five inhibitor concentrations: 500, 300, 100, 50, and 10 nM. The data was normalized to expressed p-MAPK, and four-parameter logistic dose–response curves were fitted to the data points.

c The Caco-2–efflux ratio (Mean *P*_app_ B2A / Mean *P*_app_ A2B).

Disappointingly, there was a very low correlation with the potency seen for the primary CSF1R kinase assay, but when subjected to the murine bone marrow-derived macrophage assay, several compounds displayed activity on par with pexidartinib. The Ba/F3 cell assay showed superior activity for pexidartinib, whereas some compounds found in the kinase assays as excellent inhibitors were inactive. Of the new inhibitors, the metabolically unstable 3-pyridyl **39** performed best toward Ba/F3 cells. Unfortunately, both the phenol **36** and 3-pyridyl **39** severely affected the viability of the Ba/F3 cells when interleukin-3 was simultaneously administered as a control for toxicity. The efflux ratios also clearly indicated the carboxylic acid group to be highly unfavorable, which probably also contributed to them being inactive in the Ba/F3 assay.

### Chemistry

**Scheme 2:**
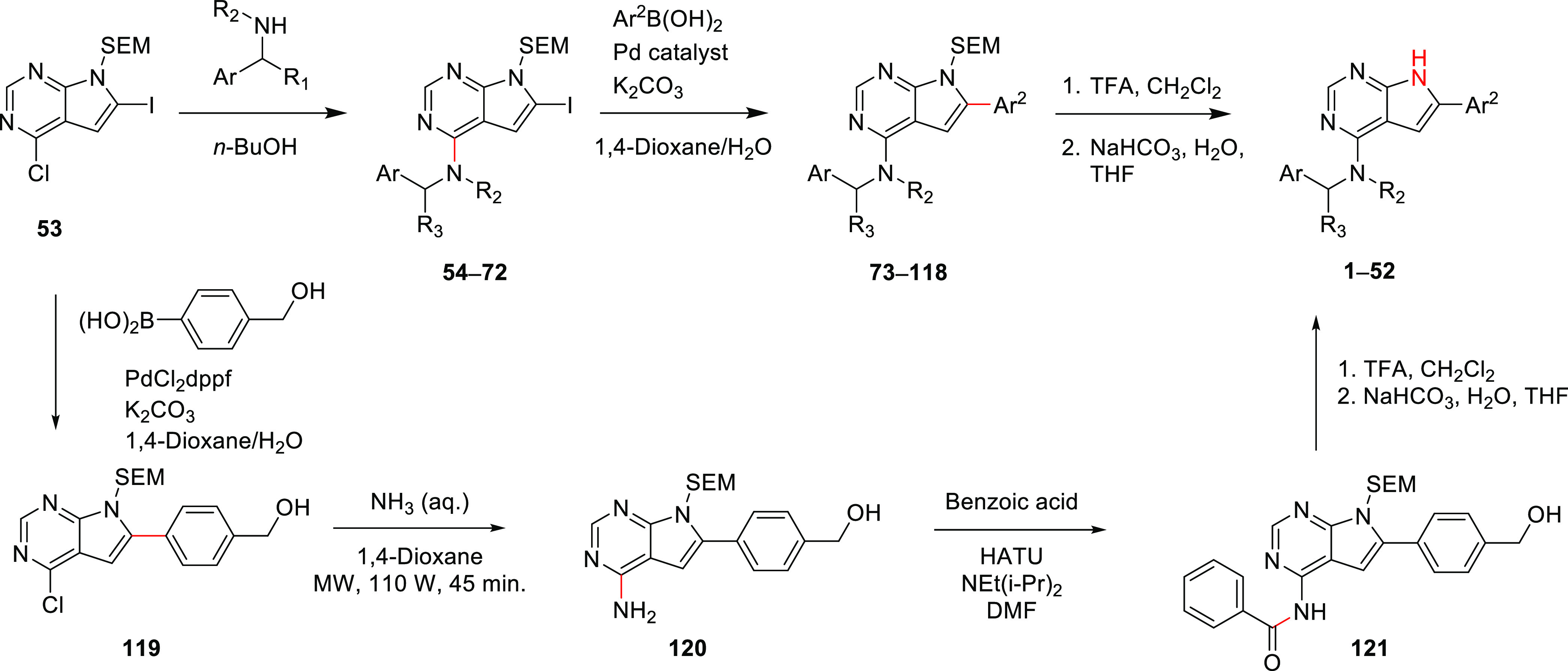
Synthesis of the CSF1R Inhibitors **1–52**

The CSF1R inhibitor structures were prepared from trimethylsilylethoxymethyl (SEM)-protected 4-chloro-6-iodopyrrolopyrimidine **48**, which can easily be prepared on a gram scale. Protection of the pyrrole NH ensures higher reaction rates and easier purifications in the following two steps. First, an amination under thermal conditions, using *n*-BuOH or *i*-PrOH as a solvent, was performed using 21 different amines yielding the advanced precursors **49**–**69**, see Scheme 2. The conversion times largely depended on the steric bulk near the NH center of the amine in question. Next, a Suzuki–Miyaura cross-coupling reaction was used to introduce the C-6 aryl substituents. Most of the couplings were performed with Pd(dppf)_2_Cl_2_ as a catalyst, which resulted in a very rapid conversion of starting material (minutes); however, other catalysts were also used to good effect. Four post-modifications were performed on the Suzuki-derived products. These included the reduction of two benzaldehydes to the corresponding benzylic alcohols and two reductive aminations. Deprotection of the 4,6-disubstituted derivatives was carried out using trifluoroacetic acid (TFA), followed by neutralization in aqueous NaHCO_3_. The carboxylic acid derivatives **45** and **48** were derived from the corresponding methyl esters. Hydrolysis of methyl esters after SEM deprotection was found to be most favorable in terms of purification.

The tool compound **20**, containing a benzamide at C-4, could not be obtained following the route shown above, as the Suzuki reaction failed for the first time. Instead, a chemoselective Suzuki–Miyaura cross-coupling reaction at C-6 was used to obtain **119**, which was then transformed into **20** via the intermediates **120** and **121**.

## Conclusions

Rational molecular design and activity optimization allowed us to develop a range of exquisite selective pyrrolo[2,3-*d*] pyrimidines as inhibitors for CSF1R. The binding mode for compound **23** was established by crystallography and revealed that this compound class binds to the protein in a nonclassical DFG-out-like conformation. A binding mode assay further established that these structures bind preferably to the autoinhibited form of the kinase, contrasting that of pexidartinib.

Cellular potency was evaluated by blocking the survival mediated by CSF1R in primary murine bone marrow-derived macrophages (BMDM) and in the CSF-dependent cell line Ba/F3. Although most of the compounds had mediocre activity when compared to pexidartinib in the Ba/F3 cell line, a handful displayed activity similar to Pexidartinib in the BMDM assay.

The medicinal relevance of conformation-selective inhibitors is unclear. However, access to highly selective CSF1R inhibitors is still quite limited. To the best of our knowledge, our series of pyrrolo[2,3-*d*] pyrimidines represent the first set of inhibitors that exclusively bind to CSF1R in a true autoinhibited conformation, potentially leading to drug leads with unique characteristics. Further optimizations to improve cellular potency, pharmacokinetic properties, and *in vivo* stability and efficacy for this compound class are presently ongoing, and the results will be presented in due course.

## Experimental Section

### General Methods

Most reagents and solvents used were purchased from Merch, VWR, or Alfa Aesar and used without further purification. Pexidartinib/PLX3397 was obtained from Selleckchem, Erlotinib was from Apollo Scientific, and 4-chloro-7*H*-pyrrolo[2,3-*d*]pyrimidine was from 1ClickChemisry Inc. Compounds **17**(18) and **34**(41) were previously prepared materials. Reactions sensitive to moisture or oxygen were conducted under a N_2_ atmosphere using oven-dried glassware and solvents dried over molecular sieves for 24 h or collected from an MBraun SPS-800 solvent purifier. High-performance liquid chromatography (HPLC) was performed on either a Waters Acquity ultrahigh-pressure liquid chromatography (UPLC) system or an Agilent 1100 series instrument. Waters MassLynx 4.1 or Agilent Chemstation was used as software. HPLC method A: A Waters Acquity BEH C18 (50 mm × 2.1 mm, 1.7 μm) column was used, running at a flow rate of 0.5 mL/min. A gradient elution using MeCN/H_2_O as a mobile phase was performed as follows: 5% MeCN for 0.5 min, then a linear gradient up to 95% MeCN over 7.5 min, and finally 95% MeCN for 1.5 min. The column was kept at a temperature of 60 °C. For each run, 5 μL of a 200 μM solution of the analyte dissolved in MeOH/H_2_O (75:25 vol %) was injected. Method B: The column used was a Poroshell 120 EC-C18 (100 mm × 4.6 mm, 2.7 μm pore size), with a flow of 1 mL/min, 15 min linear gradient of MeCN/(H_2_O + 0.1% TFA), 10:90 to MeCN/(H_2_O + 0.1% TFA), 0:100, followed by 5 min elution at 100% MeCN. Accurate mass determination in positive and negative modes was performed on a “Synapt G2-S” Q-TOF instrument from WatersTM. Samples were ionized using an ASAP probe (APCI). No chromatographic separation was done prior to the mass analysis. Calculated exact mass and spectra processing was done by WatersTM Software (MassLynx V4.1 SCN871). NMR spectra were recorded using the Bruker DPX 400 and 600 MHz Avance III HD NMR spectrometers. Chemical shifts (δ) are recorded in parts per million relative to TMS (δH = 0.00, δC = 0.0) or DMSO-*d*_6_ (δH = 2.50, δC = 39.5), and coupling constants (*J*) are measured in hertz (Hz).

### CSF1R Enzymatic Inhibitory Assay (Z-LYTE)

The compounds were supplied in a 10 mM dimethyl sulfoxide (DMSO) solution, and enzymatic CSF1R inhibition potency was determined by Invitrogen (Thermo Fisher) using their Z′-LYTE assay technology.^42^ The assay is based on fluorescence resonance energy transfer (FRET). In the primary reaction, the kinase transfers the γ-phosphate of ATP to a single tyrosine residue in a synthetic FRET peptide. In the secondary reaction, a site-specific protease recognizes and cleaves nonphosphorylated FRET peptides. Thus, phosphorylation of FRET peptides suppresses cleavage by the development reagent. Cleavage disrupts FRET between the donor (i.e., coumarin) and acceptor (i.e., fluorescein) fluorophores on the FRET peptide, whereas uncleaved, phosphorylated FRET peptides maintain FRET. A ratiometric method, which calculates the ratio (the emission ratio) of donor emission to acceptor emission after excitation of the donor fluorophore at 400 nm, is used to quantitate inhibition. All compounds were first tested for their inhibitory activity at 500 nM in duplicates. The potency observed at 500 nM was used to set the starting point of the IC_50_ titration curve, in which three levels were used, 1000 or 10 000 nM. The IC_50_ values reported are based on the average of at least 2 titration curves (minimum 20 data points) and calculated from activity data with a four-parameter logistic model using SigmaPlot (Windows Version 12.0 from Systat Software, Inc.). Unless stated otherwise, the ATP concentration used was equal to *K*_m_ (ca 10 mM). The average standard deviations for single-point measurements were <4%.

### CSF1R Enzymatic Inhibitory Assay (LANCE)

The TR-FRET-based LANCE *Ultra* assay (Perkin Elmer) was used to determine IC_50_ values for various CSF1R inhibitors. Kinase activity and inhibition in this assay were measured as recommended by the manufacturer. Briefly, a specific Ultra ULight GT peptide substrate (50 nM final concentration) was allowed to get phosphorylated by CSF1R (0.5 nM final concentration) in enzymatic buffer (50 mM HEPES pH 7.5, 10 mM MgCl_2_, 1 mM EGTA, 0.01% Tween 20, 2 mM dithiothreitol, 1% DMSO) containing ATP at the concentration of the *K*_m_ value (25 μM) of the kinase for 1 h at room temperature. All compounds were tested in an 8-point dose–response curve up to a final concentration of 10 μM. The compound transfer was facilitated via acoustic dispensing with the Echo 520 (Beckman Labcyte) using the Echo Dose–Response software package. Subsequently, phosphorylation or inhibition was detected by the addition of specific europium (Eu)-labeled anti-phospho antibodies (2 nM), which upon binding to the phospho-peptide gives rise to a FRET signal. The FRET signal was recorded in a time-resolved manner in a Perkin Elmer EnVision reader. All assays were performed in a final volume of 20 μL in low-volume white 384-well plates from Corning (4513). All assay data were analyzed with the Quattro Workflow software package from Quattro Research.

### Kinase Binding Assay (*K*_d_)

Kinase-tagged T7 phage strains were prepared in an *Escherichia coli* host derived from the BL21 strain. *E. coli* were grown to log-phase and infected with T7 phage and incubated with shaking at 32 °C until lysis. The lysates were centrifuged and filtered to remove cell debris. The remaining kinases were produced in HEK-293 cells and subsequently tagged with DNA for quantitative polymerase chain reaction (qPCR) detection. Streptavidin-coated magnetic beads were treated with biotinylated small molecule ligands for 30 min at room temperature to generate affinity resins for kinase assays. The liganded beads were blocked with excess biotin and washed with blocking buffer (SeaBlock (Pierce), 1% BSA, 0.05% Tween 20, 1 mM DTT) to remove unbound ligands and to reduce nonspecific binding. Binding reactions were assembled by combining kinases, liganded affinity beads, and test compounds in 1× binding buffer (20% SeaBlock, 0.17× PBS, 0.05% Tween 20, 6 mM DTT). Test compounds were prepared as 111X stocks in 100% DMSO. *K*_d_ values were determined using an 11-point 3-fold compound dilution series with three DMSO control points. All compounds for *K*_d_ measurements are distributed by acoustic transfer (non-contact dispensing) in 100% DMSO. The compounds were then diluted directly into the assays such that the final concentration of DMSO was 0.9%. All reactions were performed in the polypropylene 384-well plate. Each well contained a final volume of 0.02 mL. The assay plates were incubated at room temperature with shaking for 1 h, and the affinity beads were washed with wash buffer (1× PBS, 0.05% Tween 20). The beads were then resuspended in elution buffer (1× PBS, 0.05% Tween 20, 0.5 μM non-biotinylated affinity ligand) and incubated at room temperature with shaking for 30 min. The kinase concentration in the eluates was measured by qPCR. Binding constants (*K*_d_) were calculated with a standard dose–response curve using the Hill equation with the Hill Slope set to −1. Curves were fitted using a non-linear least square fit with the Levenberg–Marquardt algorithm. The experiments were performed by Eurofins.

### Kinase Panel

The kinase panel assays were run as described above but at a single concentration (500 nM). The experiments were performed by Eurofins.

### ADME Properties

#### Kinetic Solubility

The aqueous solubility of compounds was determined by spectrophotometrical measurement of the kinetic solubility of a 500 μM compound solution in an aqueous buffer, pH 7.4, compared to a solution in the organic solvent acetonitrile after 90 min of vigorous shaking at room temperature.

#### Microsomal Stability Phase I

Metabolic stability under oxidative conditions was measured in liver microsomes from different species supplemented with NADP, glucose-6-phosphate (G6P), and G6P-dihydrogenase by liquid chromatography–mass spectrometry (LCMS)-based measuring of depletion of a compound at a concentration of 3 μM over time up to 50 min at 37 °C. Based on compound half-life *t*_1/2_, in vitro intrinsic clearance CL_int_ was calculated: Cl_int_ = *V* × 0.693/(*t*_1/2_ × mg).

#### Microsomal Stability Phase II

Metabolic stability under conjugative conditions was measured in the glucuronidation assay by LCMS-based determination of %remaining of selected compounds at a concentration of 5 μM following incubation with liver microsomes from different species supplemented with UDPGA for 1 h at 37 °C.

#### Plasma Stability

Plasma stability was measured by LCMS-based determination of %remaining of selected compounds at a concentration of 5 μM after incubation in 100% plasma obtained from different species for 1 h at 37 °C.

#### Plasma Protein Binding

Assessment of plasma protein binding was measured by equilibrium dialysis by incubating plasma with the compound of interest at a concentration of 5 μM for 6 h at 37 °C, followed by LCMS-based determination of final compound concentrations.

#### Caco-2

In the Caco-2 cell assay, a 10 mM DMSO stock of the inhibitor was diluted to a final concentration of 5 μM in Hanks’ balanced salt solution (HBSS) buffer at pH 7.4 and incubated for 2 h at 37 °C and 5% CO_2_ on a monolayer of Caco-2 cells (ATCC) that had been grown on a Transwell membrane (Millipore, Schwalbach, Germany) for 21 days. The compound concentration was measured in the receiver and the donor well. Apparent permeability (*P*_app_) from the apical to basolateral direction and from the basolateral apical direction was calculated by the equation *P*_app_ = [1/(*AC*_0_)](d*Q*/d*t*), where *A* is the membrane surface area, *C*_0_ is the inhibitor concentration at *t* = 0, and d*Q*/d*t* is the amount of inhibitor transported within the given time period of 2 h.

### Cell Assays

#### Cell Viability Assay with Ba/F3-hCSF1R Cells

Ba/F3-hCSF1R cells expressing human CSF1R were kindly provided by C. Pridans, Center for Inflammation Research, University of Edinburgh. The cells were maintained in RPMI 1640 (PAN Biotech, Cat. No.: P04-22100) supplemented with 10% fetal calf serum, 1% glutamine, and 100 ng/mL human M-CSF (Thermo, #14-8789-80, 0.1 mg/mL) and grown at 37 °C in 5% CO_2_. For the cell viability assay, cells were seeded with a density of 1200 cells per well in 25 μL in 384-well plates (Greiner Bio-One, Frickenhausen, Germany; order no. 781080) in a medium without human M-CSF. Shortly after seeding, compounds were added to each sample well by using Echo Acoustic Liquid Transfer technology (Labcyte) with 10 μM as the highest concentration and 7 further 3-fold dilution steps down to 0.007 μM. Wells with cells and 0.1% DMSO in the culture medium were used as positive controls, and wells with cells and 10 μM staurosporine in the culture medium were used as negative controls. After a 30 min incubation time at 37 °C/5% CO_2_, the cells were stimulated by adding 10 μM human M-CSF (final conc.) to get a final assay volume of 35 μL. The cells were incubated with the compounds for 72 h at 37 °C/5% CO_2_. For measurement of cell viability, 35 μL of Cell Titer Glo reagent (Promega, Madison; order no. G7573)—1:2 diluted with cell culture medium—was added to each well. The 384-well plates were placed for 2 min on an orbital microplate shaker and incubated for further 10 min at room temperature to stabilize the luminescence signal. Luminescence was measured using a Victor X5 Reader (Perkin Elmer). EC_50_ values were calculated with the software Excel Fit (IDBS, Guildford, U.K.) from 3-fold dilution series comprising at least 8 concentrations in duplicates.

#### Effect of CSF1R Inhibitors on MAPK Signaling in Mouse Bone Marrow-Derived Macrophages

Bone marrow-derived macrophages were obtained by flushing the femur and tibia of sacrificed C57BLKS mice with HBSS (Hanks’ balanced salt solution) (Sigma-Aldrich; order no. H9269) using a syringe with a 25G needle. The cells were centrifuged at 1500 rpm for 8 min, the resulting supernatant was decanted, and the cells were resuspended in 5 mL of RBC (red blood cell) lysis buffer (Thermo Fisher; order no. 00-4333-27). Lysis was stopped by adding 30 mL of RPMI medium (Sigma-Aldrich; order no. R8758) containing 10% FCS (Fetal Calf Serum) (Gibco; order no. 10270). The cells were centrifuged at 1500 rpm for 8 min. The supernatant was decanted, and the cells were resuspended in RPMI medium containing 0.02 mg/mL Gensumycin (Sanofi-Aventis; order no. 453130), 2 mM glutamine (Sigma-Aldrich; order no. G7513), and 10% FCS with 10 ng/mL CSF-1 (R&D systems; order no. 416-ML). The cells were seeded in bacterial plates. After 2 days, fresh medium with 10 ng/mL CSF-1 was added, and after another 2 days, 50% of the medium was replaced with fresh medium containing 10 ng/mL CSF-1 while the other 50% was centrifuged to get rid of dead cells before being transferred back to the cells. After incubating for one week, the differentiated cells were washed twice with PBS, PBS EDTA (0.2 mM) was added, and incubated for 10 min. Cells were detached by scraping and centrifuged at 1200 rpm for 7 min. The supernatant was decanted, and the cells were resuspended in the medium with 10 ng/mL CSF-1. The cells were seeded out in 96-well glass bottom plates (Cellvis; order no. P96-1.5H-N) at 50.000 cells in 100 μL per well and incubated at 37 °C overnight. The medium was removed, and the cells were washed three times with PBS before being starved overnight in 0.1% FCS medium without CSF-1. CSF1R inhibitors dissolved in DMSO were added to the wells in appropriate concentrations and incubated for 30 min at 37 °C. DMSO was added to control wells at the highest inhibitor concentration. CSF-1 (0.1 mg/mL) was added to all wells, except for the CSF-1 negative control, to obtain an end concentration of CSF-1 of 10 ng/mL. After incubating for another 10 min at 37 °C, the cells were fixed by adding paraformaldehyde (PFA) (16%) to obtain an end concentration of 4% for 10 min. The cells were washed twice with *tert*-butyldimethylsilyl (TBS) and permeabilized by MeOH for 10 min on ice. The cells were washed twice with TBS and blocked in Odyssey blocking solution (Licor; order no. 927-60001) diluted 1:1 in TBS-Tween (0,1%) for 1.5 h under careful agitation. The blocking solution was removed, and appropriately diluted primary antibody solution (P-MAPK (ERK1/2, Thr202/Tyr204) (Cell Signaling Technology; order no. 4370, rabbit) 1:1200 and MAPK (ERK1/2), (BioLegend; order no. 686902, rat) 1:300 in Odyssey blocking buffer:TBS-Tween (1:1)) was added to the wells. After incubating overnight with careful shaking at 4 °C, the cells were washed with TBST five times for 5 min while agitating. The secondary antibody solution was added, and the wells were incubated for 1 h in the dark with IRdye 800CW goat anti-rabbit (Licor; order no. 926-32211) and IRdye 680RD goat anti-rat (Licor; order no. 962-68076) diluted 1:800 in Odyssey blocking buffer:TBS-Tween (1:1). The antibody solution was removed, and the wells were washed 4 × 5 min with TBST and 2 × 5 min with TBS while carefully agitating. The TBS was removed, and the plate was scanned on an Odyssey Near-Infrared scanner (Licor, Lincoln, Nebraska). Using Image Studio software, the intensity of fluorescence of each well is recorded after subtracting the background noise (primary antibody was not added to the wells). The results were normalized by dividing the P-MAPK intensity by the total MAPK intensity for all of the wells. The average value of triplicate wells was calculated for every concentration of inhibitor used. The average values are then divided by the CSF-1 positive control value. PLX3397 was included as a reference on all plates. Due to some inter-assay variation, the activity of the inhibitors is also reported as fold change relative to PLX3397 (Fold change: IC_50_ inhibitor/IC_50_ PLX3397).

#### In Vivo Pharmacokinetic Study

The in vivo pharmacokinetic profiling of **4**, **10**, **13**, **45**, and **47** and PLX3397 was performed in female C57BLKS mice (*n* = 3) by cassette intravenous (iv) single dosing of drugs (1 mg/kg each) in a 20% DMSO, 80% PEG400 formulation. Blood sampling was done after 10, 30, 60, 120, 240, and 480 min. The work, following the EU Directive 2010/63/EU for animal experiments, was conducted under the global project 2017072717008661#10796 V8 approved by the ethical committee and national authorities (CEEA-LR-n°036—authorization number 10796) on November 27th, 2019 for 5 years and was conducted at Eurofins ADME Bioanalyses, 30310 Vergèze, France (accreditation number D303441). Analysis was performed by LCMS.

#### Crystallization

The CSF1R construct used for crystallization was produced as described previously (PDB entry 4hw7),^43^ except that the present construct used lacks the mutations C667T, C830S, and C907T.

CSF1R in complex with compound **23** was crystallized by sitting-drop vapor diffusion at 20 °C. CSF1R (10.5 mg/mL in 150 mM NaCl, 20 mM HEPES–NaOH (pH 7.0), and 10 mM DTT) was incubated with 1.5 mM compound **23** and 0.1% w/w V8 protease for 1 h at 4 °C. The digest was then stopped by the addition of 5 mM benzamidine (final concentration). Then, 0.14 μL of the sample was mixed with 0.28 μL of crystallization solution (0.3 M DL-malic acid (pH 7.0) and 23.0% w/v PEG 3350) and equilibrated against a reservoir containing 0.06 mL crystallization solution. The crystals were mounted after 4 days. Crystals were cryo-protected in a crystallization solution supplemented with 20% v/v glycerol and cooled in liquid nitrogen. Data were collected at beamline ID29 of the European Synchrotron Radiation Facility (Grenoble, France).

#### Structure Determination

Diffraction data were integrated, analyzed, and scaled with energy-dispersive X-ray spectrum (XDS),^44^ POINTLESS, and AIMLESS,^45^ respectively, in AUTOPROC.^46^ The structure was determined by rigid-body refinement with REFMAC5^47^ using an isomorphous model CSF1R (without any ligands) as a starting model (PDB entry 2i1m). The model was improved through the manual rebuilding of the model in COOT^48^ and restrained refinement with REFMAC5. Atomic displacement factors were modeled with an isotropic B-factor per atom. The backbone geometry was analyzed with MOLPROBITY.^49^ The restraints for the modeled compounds were generated with LIBCHECK.

### General Procedures

#### General Procedure A: Amination of Protected Pyrrolopyrimidines

4-Chloro-6-iodo-7-((2-(trimethylsilyl)-ethoxy)methyl)-7*H*-pyrrolo[2,3-*d*]pyrimidine (1.00 g, 1 equiv) was dissolved in dry *n*-BuOH or dioxane (10 mL), and benzylamine (1.5–3 equiv) and optionally *N,N*-diisopropylethylamine (3 equiv) were added. The reaction was stirred at 100–140 °C for 4–24 h. Following the evaporation of the solvent, water (20 mL) and EtOAc (50 mL) were added to the residue. After phase separation, the water phase is extracted with more EtOAc (3 × 50 mL). The combined organic phase is then dried over MgSO_4_ and concentrated at low pressure. The products were purified by silica-gel column chromatography as specified below.

#### General Procedure B: Suzuki-Cross-Coupling of Aminated Pyrrolopyrimidines

4-Amino-6-iodo-7-((2-(trimethylsilyl)-ethoxy)methyl)-7*H*-pyrrolo[2,3-*d*]pyrimidine (1.0 equiv), aryl boronic acid or pinacol ester (1.0–1.2 equiv), PdCl_2_dppf (2–5 mol %), and potassium carbonate (3.0 equiv) are charged in an appropriate reaction vessel. The atmosphere is evacuated and back-filled with N_2_ three times before adding degassed 1,4-dioxane (6 mL/mmol starting material) and degassed water (3 mL/mmol starting material). The reaction vessel is lowered into an oil bath set at 60–80 °C and stirred vigorously. Upon reaction completion, the reaction vessel is raised from the oil bath and allowed to cool for 5 min before the reaction mixture is transferred to a round-bottomed flask, and the volatiles are removed by rotary evaporation. Water is added to the residue (20 mL/mmol starting material) and extracted with CH_2_Cl_2_ (3 × 20 mL/mmol starting material). The combined organic layers are washed with brine (20 mL/mmol), dried with anhydrous Na_2_SO_4_, and filtered. The organic solvent is removed under reduced pressure, and the crude product is purified by silica-gel column chromatography. Some transformations were performed with alternative catalysts. This is specified.

#### General Procedure C: SEM Deprotection

The SEM-protected pyrrolopyrimidine (0.2 mmol, 1 equiv) was stirred in TFA (2 mL) and CH_2_Cl_2_ (10 mL) at 50 °C for 3–24 h. The reaction mixture was then concentrated *in vacuo* before it was taken up in MeOH (10 mL) and NH_3_ (20 mL, 25% aqueous) and stirred for 2–24 h at 22 °C. The reaction mixture was concentrated *in vacuo*, and the crude product was purified by silica-gel column chromatography. For some compounds, the last step of the procedure was run with NaHCO_3_ instead of ammonia and THF instead of MeOH.

##### (4-(4-(Benzyl(methyl)amino)-7*H*-pyrrolo[2,3-*d*]pyrimidin-6-yl)phenyl)methanol (**1**)

Compound **73** (179 mg, 0.377 mmol) was treated as described in General Procedure C. The solid mass obtained was triturated with EtOAc, resulting in 121 mg (0.352 mmol, 93%) of a white solid, mp 153–157 °C (decomp.); HPLC purity: 97.6% (method A); ^1^H NMR (600 MHz, DMSO-*d*_6_) δ 12.15 (br s, 1H), 8.14 (s, 1H), 7.82–7.79 (m, 2H), 7.36–7.33 (m, 2H), 7.33–7.31 (m, 2H), 7.30–7.26 (m, 2H), 7.26–7.22 (m, 1H), 7.02 (s, 1H), 5.25–5.21 (m, 1H), 5.05 (s, 2H), 4.52–4.50 (m, 2H), 3.37 (s, 3H); ^13^C NMR (150 MHz, DMSO-*d*_6_) δ 156.4, 152.9, 151.0, 141.7, 138.5, 133.3, 129.9, 128.5 (2C), 127.0 (2C), 126.9, 126.8 (2C), 124.5 (2C), 103.3, 98.5, 62.6, 52.7, 37.4; IR (neat, cm^–1^): 3273 (w), 3107 (w), 3023 (w), 2869 (w), 1572 (s), 1513 (m), 1404 (m), 1317 (m), 1161 (w), 1057 (m), 937 (m), 827 (m), 765 (m), 724 (s), 697 (m). HRMS (ASAP+, *m*/*z*): found 345.1715, calcd for C_21_H_21_N_4_O, [M + H]^+^, 345.1715.

##### *N*-Benzyl-*N*-methyl-6-phenyl-7*H*-pyrrolo[2,3-*d*]pyrimidin-4-amine (**2**)

Compound **74** (166 mg, 0.374 mmol) was treated as described in General Procedure C. The solid mass obtained was triturated with CH_2_Cl_2_, resulting in 141 mg (0.49 mmol, 85%) of a white solid, mp 285–287 °C (decomp.). HPLC purity: 99% (method B); ^1^H NMR (400 MHz, DMSO-*d*_6_) δ 12.20 (br s, 1H), 8.15 (s, 1H), 7.87–7.82 (m, 2H), 7.43–7.38 (m, 2H), 7.36–7.30 (m, 2H), 7.29–7.22 (m, 4H), 7.06 (s, 1H), 5.05 (s, 2H), 3.37 (s, 3H); ^13^C NMR (100 MHz, DMSO-*d*_6_) δ 156.5, 153.0, 151.2, 138.5, 133.2, 131.5, 128.8 (2C), 128.5 (2C), 127.3, 127.0 (2C), 126.9, 124.7 (2C), 103.3, 98.9, 52.7, 37.4; IR (neat, cm^–1^): 3090 (w), 2919 (w), 2846 (w), 2732 (w), 2353 (w), 1571 (s), 1509 (m), 1405 (m), 1322 (m), 1257 (w), 1075 (w), 936 (m), 751 (m), 701 (w); HRMS (ASAP+, *m*/*z*): found 315.1605, calcd for C_20_H_19_N_4_, [M + H]^+^, 315.1610.

##### *N*-Benzyl-6-(4-methoxyphenyl)-*N*-methyl-7*H*-pyrrolo[2,3-*d*]pyrimidin-4-amine (**3**)

Compound **75** (92 mg, 0.194 mmol) was treated as described in General Procedure C. The crude product was purified by silica-gel column chromatography (CH_2_Cl_2_/MeOH–19:1, *R_f_* = 0.30). This gave 57 mg (0.165 mmol, 85%) of a white powder, mp 250–252 °C; HPLC purity 98.4% (method A); ^1^H NMR (600 MHz, DMSO-*d*_6_) δ 12.06 (s, 1H), 8.13 (s, 1H), 7.77 (d, *J* = 8.9 Hz, 2H), 7.34–7.31 (m, 2H), 7.28–7.23 (m, 3H). 6.97 (d, *J* = 8.9 Hz, 2H), 6.90 (s, 1H), 5.04 (s, 2H), 3.78 (s, 3H), 3.36 (s, 3H); ^13^C NMR (150 MHz, DMSO-*d*_6_) δ 158.7, 156.2, 152.8, 150.7, 138.5, 133.3, 128.5 (2C), 127.0 (2C), 126.8, 126.1 (2C) 124.2, 114.2 (2C), 103.3, 97.3, 55.2, 52.7, 37.3; IR (neat, cm^–1^): 3105 (w), 2962 (w), 1732 (w), 1566 (s), 1545 (s), 1401 (m), 1248 (s), 1022 (m), 831 (m). HRMS (ASAP+, *m*/*z*): found 345.1715, calcd for C_21_H_21_N_4_O, [M + H]^+^, 345.1715.

##### 4-(4-(Benzyl(methyl)amino)-7*H*-pyrrolo[2,3-*d*]pyrimidin-6-yl)phenol (**4**)

Compound **76** (217 mg, 0.471 mmol) was treated as described in General Procedure C. The crude product was purified by silica-gel column chromatography (CH_2_Cl_2_/MeOH–9:1, *R_f_* = 0.37). This gave 128 mg (0.386 mmol, 82%) of a white powder. HPLC purity > 99% (method B); ^1^H NMR (600 MHz, DMSO-*d*_6_) δ 11.98 (s, 1H), 9.59 (s, 1H), 8.11 (s, 1H), 7.67–7.62 (m, 2H), 7.35–7.30 (m, 2H), 7.29–7.26 (m, 2H), 7.26–7.22 (m, 1H), 6.82 (s, 1H), 6.81–6.76 (m, 2H), 5.03 (s, 2H), 3.34 (s, 3H); ^13^C NMR (151 MHz, DMSO-*d*_6_) δ 157.0, 156.2, 152.7, 150.5, 138.5, 133.9, 128.5 (2C), 127.0 (2C), 126.8, 126.2 (2C), 122.6, 115.5 (2C), 103.3, 96.6, 52.7, 37.3; IR (neat, cm^–1^): 3106 (w), 2959 (w), 2856 (w), 1735 (w), 1570 (s), 1405 (m), 1207 (m), 1151 (m), 862 (m). HRMS (ASAP+, *m*/*z*): found 331.1554, calcd for C_20_H_19_N_4_O, [M + H]^+^, 331.1559.

##### *N*-Benzyl-6-(4-(2-methoxyethoxy)phenyl)-*N*-methyl-7*H*-pyrrolo[2,3-*d*]pyrimidin-4-amine (**5**)

Compound **77** (123 mg, 0.237 mmol) was treated as described in General Procedure C. The crude product was purified by silica-gel column chromatography (CH_2_Cl_2_/MeOH–19:1, *R_f_* = 0.25). Drying resulted in 62.4 mg (0.161 mmol, 68%) of a white powder, mp 217–219 °C. HPLC purity: 98% (method A); ^1^H NMR (600 MHz, DMSO-*d*_6_) δ 12.06 (s, 1H), 8.13 (s, 1H), 7.76 (d, *J* = 8.9 Hz, H), 7.34–7.31 (m, 2H), 7.28–7.23 (m, 3H), 6.99 (d, *J* = 8.9 Hz, 2H), 6.90 (s, 1H), 5.04 (s, 2H), 4.14–4.10 (m, 2H) 3.70–3.64 (m, 2H) 3.35 (s, 3H), 3.31 (s, 3H); ^13^C NMR (150 MHz, DMSO-*d*_6_) δ 157.9, 156.2, 152.7, 150.7, 138.5, 133.3, 128.5 (2C), 127.0 (2C), 126.8, 126.1 (2C), 124.2, 114.7 (2C), 103.3, 97.3, 70.3, 66.9, 58.1, 52.6, 37.3; IR (neat, cm^–1^): 3108 (w), 2984 (w), 1562 (s), 1499 (m), 1248 (m), 1062 (m), 935 (w) 837 (w). HRMS (ASAP+, *m*/*z*): found 398.1974, calcd for C_23_H_25_N_4_O_2_, [M + H]^+^, 389.1978.

##### *N*-Benzyl-6-(4-(2-(2-(2-methoxyethoxy)ethoxy)ethoxy)phenyl)-*N*-methyl-7*H*-pyrrolo[2,3-*d*]pyrimidin-4-amine (**6**)

Compound **78** (54 mg, 0.089 mmol) was treated as described in General Procedure C. The crude product was purified by silica-gel column chromatography (CH_2_Cl_2_/MeOH–97.5:2.5, *R_f_* = 0.38). Drying resulted in 37 mg (0.077 mmol, 87%) of a yellow solid, mp 123–126 °C; HPLC purity: 96.6% (method A); ^1^H NMR (400 MHz, DMSO-*d*_6_) δ 12.09 (s, 1H), 8.13 (s, 1H), 7.80–7.76 (m, 2H), 7.37–7.22 (m, 5H), 7.02–6.98 (m, 2H), 6.91 (s, 1H), 5.05 (s, 2H), 4.15–4.10 (m, 2H), 3.77–3.72 (m, 2H), 3.62–3.58 (m, 2H), 3.56–3.51 (m, 4H), 3.46–3.42 (m, 2H), 3.37 (s, 3H), 3.24 (s, 3H); ^13^C NMR (100 MHz, DMSO-*d*_6_) δ 159.1, 154.3, 148.2, 146.3, 139.0, 133.9, 129.0 (6C), 127.5, 126.6, 115.2 (2C), 111.4, 103.7, 80.2, 71.7 (2C), 70.4, 70.1, 69.4, 58.6, 55.1, 37.8; HRMS (ASCI/ASAP, *m*/*z*): found 477.2496, calcd for C_27_H_32_N_4_O_4_, [M + H]^+^, 477.2502.

##### 6-(3-((1,3-Dioxolan-2-yl)methoxy)phenyl)-*N*-benzyl-*N*-methyl-7*H*-pyrrolo[2,3-*d*]pyrimidin-4-amine (**7**)

Compound **79** (78.5 mg, 0.144 mmol) was treated as described in General Procedure C. The crude product was purified by silica-gel column chromatography (CH_2_Cl_2_/MeOH–9:1, *R_f_* = 0.38). Drying resulted in 24 mg (0.057 mmol, 40%) of a light beige powder, mp 203–205 °C. ^1^H NMR (600 MHz, DMSO-*d*_6_) δ 12.15 (s,1H), 8.15 (s, 1H), 7.47–7.43 (m, 2H), 7.34–7.23 (m, 6H), 7.10 (s, 1H), 6.87–6.84 (m, 1H) 5.23 (t, *J* = 4.1 Hz, 1H), 5.05 (s, 2H), 4.06 (d, *J* = 4.1 Hz, 2H), 3.99–3.86 (m, 4H), 3.37 (s, 3H); ^13^C NMR (150 MHz, DMSO-*d*_6_) δ 158.6, 156.5, 152.9, 151.3, 138.4, 133.0, 132.9, 129.9, 128.5 (2C), 127.0 (2C), 126.9, 117.4, 113.7, 110.4, 103.2, 101.3, 99.3, 68.2, 64.5 (2C), 52.6, 37.4; IR (neat, cm^–1^): 3104 (w), 2957 (w), 2857 (w), 1572 (s), 1502 (m), 1354 (w), 1270 (m), 1068 (w), 937 (m).HRMS (ASAP+, *m*/*z*): found 417.1927, calcd for C_24_H_25_N_4_O_3_, [M + H]^+^, 417.1927.

##### 3-(4-(Benzyl(methyl)amino)-7*H*-pyrrolo[2,3-*d*]pyrimidin-6-yl)phenol (**8**)

Compound **80** (93 mg, 0.201 mmol) was treated as described in General Procedure C. The crude product was purified by silica-gel column chromatography (CH_2_Cl_2_/MeOH–19:1, *R_f_* = 0.20). Drying resulted in 46.5 mg (0.141 mmol, 70%) of a pale-yellow powder, mp 252–254 °C; HPLC purity: 95% (method A). ^1^H NMR (600 MHz, DMSO-*d*_6_) δ 12.09 (s, 1H), 9.46 (s, 1H), 8.14 (s, 1H), 7.34–7.32 (m, 2H), 7.28–7.23 (m, 4H), 7.20–7.18 (m, 2H), 6.92 (s, 1H), 6.71–6.69 (m, 1H), 5.04 (s, 2H), 3.36 (s, 3H); ^13^C NMR (150 MHz, DMSO-*d*_6_) δ 157.6, 156.4, 152.8, 151.1, 138.4, 133.5, 132.8, 129.7, 128.4 (2C), 127.0 (2C), 126.9, 115.7, 114.4, 111.7, 103.1, 98.6, 52.7, 37.3; IR (neat, cm^–1^): 3205 (w), 3112 (w), 2922 (w), 1567 (s), 1445 (m), 1405 (m), 1237 (w), 933 (m), 693 (m); HRMS (ASAP+, *m*/*z*): found 331.1558, calcd for C_20_H_19_N_4_O, [M + H]^+^, 331.1559.

##### *N*-Benzyl-6-(3-methoxyphenyl)-*N*-methyl-7*H*-pyrrolo[2,3-*d*]pyrimidin-4-amine (**9**)

Compound **81** (67 mg, 0.141 mmol) was treated as described in General Procedure C. The crude product was purified by silica-gel column chromatography (CH_2_Cl_2_/MeOH–9:1, *R_f_* = 0.55). Drying resulted in 32 mg (0.0917 mmol, 65%) of a yellow powder, mp 210–212 °C. ^1^H NMR (600 MHz, DMSO-*d*_6_) δ 12.17 (s, 1H), 8.15 (s, 1H), 7.43–7.41 (m, 2H), 7.35–7.24 (m, 6H), 7.07 (s, 1H), 6.85–6.83 (m, 1H), 5.05 (s, 2H), 3.81 (s, 3H), 3.37 (s, 3H); ^13^C NMR (150 MHz, DMSO-*d*_6_) δ 159.7, 156.5, 152.9, 151.2, 138.4, 133.1, 132.8, 129.8, 128.5 (2C), 127.0 (2C), 126.9, 117.1, 113.1, 110.0, 103.2, 99.2, 55.2, 52.7, 37.4; HRMS (ASAP+, *m*/*z*): found 345.1712, calcd for C_21_H_21_N_4_O, [M + H]^+^, 345.1715.

##### (4-(4-(Benzyl(methyl)amino)-7*H*-pyrrolo[2,3-*d*]pyrimidin-6-yl)-2-fluorophenyl)methanol (**10**)

Compound **82** was treated as described in General Procedure C. The crude product was washed with CH_2_Cl_2_ and dried before purification by silica-gel chromatography (CH_2_Cl_2_/MeOH–92:8, *R_f_* = 0.28). Drying gave 48 mg (0.134 mmol, 56%) of a white solid, mp 265–269 °C; HPLC purity: 98.7 (method B); ^1^H NMR (600 MHz, DMSO-*d*_6_) δ 12.20 (br s, 1H), 8.16 (s, 1H), 7.70–7.66 (m, 2H), 7.49–7.45 (m, 1H), 7.35–7.30 (m, 2H), 7.29–7.22 (m, 3H), 7.17 (br s, 1H), 5.26 (t, *J* = 5.7 Hz, 1H), 5.05 (s, 2H), 4.54 (d, *J* = 5.7 Hz), 3.37 (s, 3H); ^13^C NMR (150 MHz, DMSO-*d*_6_) δ 160.0 (d, *J* = 243.2 Hz), 156.5, 153.0, 151.4, 138.4, 132.3 (d, *J* = 8.8 Hz), 132.0 (d, *J* = 2.6 Hz), 129.5 (d, *J* = 5.5 Hz), 128.5 (2C), 127.8 (d, *J* = 15.4 Hz), 127.1 (2C), 126.9, 120.4 (d, *J* = 2.1 Hz), 110.9 (d, *J* = 23.8 Hz), 103.2, 99.8, 56.6, 52.6, 37.4; HRMS (ASAP+, *m*/*z*): found 363.1616, calcd for C_21_H_20_N_4_OF, [M + H]^+^, 363.1621.

##### (5-(4-(Benzyl(methyl)amino)-7*H*-pyrrolo[2,3-*d*]pyrimidin-6-yl)-2-fluorophenyl)methanol (**11**)

Compound **83** was prepared as described in General Procedure C. The crude product was washed with CH_2_Cl_2_ and dried before purification by silica-gel chromatography (CH_2_Cl_2_/MeOH–92:8, *R_f_* = 0.19). This gave 99 mg (0.273 mmol, 76%) of a white solid, mp 212–215 °C; HPLC purity: 99% (method A); ^1^H NMR (600 MHz, DMSO-*d*_6_) δ 12.19 (br s, 1H), 8.15 (s, 1H), 7.96–7.92(m, 1H), 7.78–7.74 (m, 1H), 7.35–7.30 (m, 2H), 7.29–7.26 (m, 2H), 7.26–7.23 (m, 1H), 7.23–7.18 (m, 1H), 6.99 (bs, 1H), 5.28 (t, *J* = 5.6 Hz, 1H), 5.05 (s, 2H), 4.56 (d, *J* = 5.6 Hz, 2H), 3.37 (s, 3H); ^13^C NMR (150 MHz, DMSO-*d*_6_) δ 159.1 (d, *J* = 245.6 Hz), 156.4, 153.0, 151.1, 138.4, 132.6, 129.5 (d, *J* = 15.5 Hz), 128.5 (2C), 127.9 (d, *J* = 3.1 Hz), 127.0 (2C), 126.9, 125.9 (d, *J* = 4.7 Hz), 125.1 (d, *J* = 8.3 Hz), 115.3 (d, *J* = 21.9 Hz), 103.2, 98.6, 56.9 (d, *J* = 3.4 Hz), 52.6, 37.3; IR (neat, cm^–1^): 3294 (w), 3107 (w), 2962 (w), 2920 (w), 2873 (w), 2733 (w), 1679 (w), 1573 (s), 1492 (m), 1417 (s), 1318 (m), 1240 (s), 1123 (m), 1011 (s), 925 (m), 828 (m), 767 (s), 695 (s), 611 (m); HRMS (ASAP+, *m*/*z*): found 363.1617, calcd for C_21_H_20_N_4_OF, [M + H]^+^, 363.1621.

##### *N*^1^-(4-(4-(Benzyl(methyl)amino)-7*H*-pyrrolo[2,3-*d*]pyrimidin-6-yl)-2-fluorobenzyl)-*N*^2^,*N*^2^-dimethylethane-1,2-diamine (**12**)

Compound **84** was treated as described in General Procedure C, except that NH_4_Cl was added to the extraction. The product was purified by silica-gel column chromatography (CH_2_C_2_/MeOH/25% NH_3 (aq)_–80:10:1, *R_f_* = 0.24) giving 84 mg (0.194 mmol, 60%) of a pale-yellow solid, mp 190–196 °C; HPLC purity: 95% (method A); ^1^H NMR (600 MHz, DMSO-*d*_6_) δ 12.19 (br s, 1H), 8.15 (s, 1H), 7.70–7.65 (m, 2H), 7.46–7.43 (m, 1H), 7.35–7.30 (m, 2H), 7.29–7.23 (m, 3H), 7.16 (br s, 1H), 5.05 (s, 2H), 3.73 (s, 2H), 3.37 (s, 3H), 2.56 (t, *J* = 6.4 Hz, 2H), 2.32 (t, *J* = 6.4 Hz, 2H), 2.10 (s, 6H); ^13^C NMR (150 MHz, DMSO-*d*_6_) δ 160.8 (d, *J* = 242.5 Hz), 156.5, 53.0, 151.4, 138.4, 132.1 (d, *J* = 8.7 Hz), 132.0 (d, *J* = 1.8 Hz), 130.7 (d, *J* = 5.6 Hz), 128.5 (2C), 127.0 (2C), 126.9, 126.3 (d, *J* = 15.5 Hz), 120.4 (d, *J* = 2.4 Hz), 111.0 (d, *J* = 24.2 Hz), 103.2, 99.7, 58.7, 52.6, 46.1, 45.8 (d, *J* = 1.5 Hz), 45.2 (2C), 37.4; IR (neat, cm^–1^): 3210 (w), 3111 (w), 3023 (w), 2940 (w), 2810 (w), 2758 (w), 1567 (s), 1546 (m), 1508 (m), 1403 (m), 1320 (m), 1251 (m), 1166 (w), 1072 (m), 934 (m), 858 (m), 768 (s), 728 (s), 695 (m), 639 (m). HRMS (ASAP+, *m*/*z*): found 433.2509, calcd for C_25_H_30_N_6_F, [M + H]^+^, 433.2516.

##### *N*^1^-(5-(4-(Benzyl(methyl)amino)-7*H*-pyrrolo[2,3-*d*]pyrimidin-6-yl)-2-fluorobenzyl)-*N*^2^,*N*^2^-dimethylethane-1,2-diamine (**13**)

Compound **85** was treated as described in General Procedure C, except that NH_4_Cl was added to the extraction. Purification was performed by silica-gel column chromatography (CH_2_Cl_2_/MeOH/25% NH_3 (aq)_–80:10:1, *R_f_* = 0.17) giving 96 mg (0.222 mmol, 73%) of a white crystalline solid, mp 145–147 °C; HPLC purity > 99% (method B); ^1^H NMR (600 MHz, DMSO-*d*_6_) δ 12.16 (br s, 1H), 8.15 (s, 1H), 7.92–7.88 (m, 1H), 7.76–7.72 (m, 1H), 7.35–7.31 (m, 2H), 7.29–7.26 (m, 2H), 7.26–7.23 (m, 1H), 7.22–7.18 (m, 1H), 7.00 (br s, 1H), 5.05 (s, 2H), 3.75 (s, 2H), 3.37 (s, 3H), 2.59 (t, *J* = 6.4 Hz, 2H), 2.32 (t, *J* = 6.4 Hz, 2H), 2.09 (s, 6H); ^13^C NMR (150 MHz, DMSO-*d*_6_) δ 159.8 (d, *J* = 245.1 Hz), 156.4, 153.0, 151.1, 138.4, 132.6, 128.5 (2C), 128.0 (d, *J* = 16.0 Hz), 127.8 (d, *J* = 3.0 Hz), 127.03 (2C), 126.96 (d, *J* = 4.7 Hz), 126.9, 124.9 (d, *J* = 8.0 Hz), 115.4 (d, *J* = 22.5 Hz), 103.2, 98.6, 58.7, 52.6, 46.3 (d, *J* = 1.5 Hz), 46.2, 45.2 (2C), 37.4; IR (neat, cm^–1^): 3106 (w), 2940 (w), 2810 (w), 2758 (w), 1564 (s), 1489 (m), 1457 (m), 1407 (m), 1318 (m), 1231 (m), 1153 (w), 1070 (m), 933 (m), 818 (m), 765 (s), 726 (m), 696 (s), 633 (m). HRMS (ASAP+, *m*/*z*): found: 433.2510, calcd for C_25_H_30_N_6_F, [M + H]^+^, 433.2516.

##### (4-(4-(Benzylamino)-7*H*-pyrrolo[2,3-*d*]pyrimidin-6-yl)phenyl)methanol (**14**)

Compound **86** (15 mg, 0.028 mmol) was treated as described in General Procedure C, using NaHCO_3_ in the second step. The crude product was purified by silica-gel column chromatography (MeOH/CH_2_Cl_2_–1:9). Drying gave 39 mg (0.118 mmol, 95%) of a colorless solid, mp 276–277 °C (decomp.). TLC (CH_2_Cl_2_/MeOH–94:6): *R_f_* = 0.13; HPLC purity 97.8 (method B) ^1^H NMR (600 MHz, DMSO-*d*_6_) δ 12.10 (s, 1H), 8.13 (s, 2H), 7.76–7.71 (m, 2H), 7.40–7.33 (m, 4H), 7.36–7.29 (m, 2H), 7.27–7.21 (m, 1H), 7.00 (s, 1H), 5.21 (s, 1H), 4.75 (d, *J* = 6.0 Hz, 2H), 4.52 (s, 2H); ^13^C NMR (151 MHz, DMSO-*d*_6_) δ 155.3, 151.2 (2C), 141.8, 140.0, 133.8, 130.1, 128.3 (2C), 127.3 (2C), 127.0 (2C), 126.7, 124.4 (2C), 103.8, 95.7, 62.6, 43.3; IR (neat, cm^–1^): 3289 (w), 3110 (w), 3024 (w), 2927 (w), 1608 (s), 1595 (s), 1485 (m), 1347 (m), 1316 (m), 1026 (m), 1013 (s), 811 (m), 789 (m), 695 (s); HRMS (ASAP+, *m*/*z*): found 331.1555, calcd for C_20_H_19_N_4_O, [M + H]^+^, 331.1559.

##### (4-(4-(Benzyl(ethyl)amino)-7*H*-pyrrolo[2,3-*d*]pyrimidin-6-yl)phenyl)methanol (**15**)

Compound **87** (57.6 mg, 0.118 mmol) was treated as described in General Procedure C, using NaHCO_3_ in the second step. The crude product was purified by silica-gel column chromatography (MeOH/CH_2_Cl_2_–1:9). Drying gave 38 mg (0.106 mmol, 90%) of a colorless solid, mp 264–266 °C (decomp.). TLC (silica, CH_2_Cl_2_/MeOH–94:6): *R_f_* = 0.17; HPLC purity > 99 (method B); ^1^H NMR (600 MHz, DMSO-*d*_6_): 12.12 (br s, 1H), 8.14 (s, 1H), 8.13 (bs, 1H), 7.78–7.76 (m, 2H), 7.34–7.30 (m, 6H), 7.25–7.23 (m, 1H), 6.85 (m, 1H), 5.19 (t, *J* = 5.7 Hz, 1H), 5.03 (s, 2H), 4.50 (d, *J* = 5.7 Hz, 2H), 3.78 (q, *J* = 7.0 Hz, 2H), 1.24 (t, *J* = 7.0 Hz, 3H); ^13^C NMR (150 MHz, DMSO-*d*_6_): 155.7, 152.9, 151.1, 141.7, 138.9, 133.5, 129.9, 128.4 (2C), 127.0 (2C), 126.8 (2C), 126.8, 124.5 (2C), 102.5, 98.1, 62.6, 50.5, 43.1, 13.3; IR (neat, cm^–1^): 3261 (w), 3108 (w), 2976 (w), 2932 (w), 2864 (w), 2736 (w), 1567 (s), 1498 (m), 1432 (m), 1346 (m), 1315 (m), 1285 (m), 1157 (w), 1058 (w), 984 (w), 916 (m), 826 (m), 766 (s), 724 (s); HRMS (ASAP+, *m*/*z*): found 359.1866, calcd for C_22_H_23_N_4_O, [M + H]^+^, 359.1872.

##### (4-(4-(Benzyl(isopropyl)amino)-7*H*-pyrrolo[2,3-*d*]pyrimidin-6-yl)phenyl)methanol (**16**)

Compound **88** (60.4 mg, 0.120 mmol) was treated as described in General Procedure C, using NaHCO_3_ in the second step. The crude product was purified by silica-gel column chromatography (CH_2_Cl_2_/MeOH–19:1). Drying gave 33.4 mg (0.090 mmol, 75%) of a yellow solid, mp 267–268 °C (decomp.). TLC (silica, CH_2_Cl_2_/MeOH–9:1): *R_f_* = 0.35; HPLC purity > 99% (method B); ^1^H NMR (400 MHz, DMSO-*d*_6_) δ 12.10 (br s, 1H), 8.13 (s, 1H), 7.69–7.67 (m, 2H), 7.33–7.31 (m, 2H), 7.29–7.28 (m, 4H), 7.22–7.16 (m, 1H), 6.70 (s, 1H), 5.27–5.20 (m, 1H), 5.18 (t, *J* = 5.8 Hz, 1H), 4.97 (s, 2H), 4.49 (d, *J* = 5.7 Hz, 2H), 1.22–1.20 (m, 6H); ^13^C NMR (151 MHz, DMSO-*d*_6_) δ 156.5, 153.1, 151.0, 141.8, 140.6, 133.4, 130.0, 128.3 (2C), 127.0 (2C), 126.4, 126.3 (2C), 124.5 (2C), 103.1, 98.5, 62.7, 47.4, 45.8, 20.4 (2C); IR (neat, cm^–1^): 3209 (w), 3125 (w), 2979 (w), 2875 (w), 2738 (w), 1569 (s), 1557 (s), 1488 (s), 1435 (s), 1320 (m), 1291 (m), 1056 (m), 1027 (s), 1018 (m), 927 (m), 838 (m), 725 (s); HRMS (ASAP+, *m*/*z*): found 373.2022, calcd for C_23_H_25_N_4_O, [M + H]^+^, 373.2028.

##### (*R*)-(4-(4-(Methyl(1-phenylethyl)amino)-7*H*-pyrrolo[2,3-*d*]pyrimidin-6-yl)phenyl)methanol (**18**)

Compound **89** (99 mg, 0.203 mmol) was treated as described in General Procedure C, using NaHCO_3_ in the second step. The crude product was purified by silica-gel column chromatography (CH_2_Cl_2_/7 M NH_3_ in MeOH–92.5:7.5, *R_f_* = 0.23). Drying gave 28 mg (0.077 mmol, 38%) of a white solid, mp >250 °C (decomp.); HPLC purity: 96.3 (method A); ^1^H NMR (600 MHz, DMSO-*d*_6_) δ 12.15 (br s, 1H), 8.17 (s, 1H), 7.86–7.81 (m, 2H), 7.39–7.31 (m, 6H), 7.29–7.24 (m, 1H), 7.08 (s, 1H), 6.45 (s, 1H), 5.19 (t, *J* = 5.7 Hz, 1H), 4.51 (d, *J* = 5.7 Hz, 2H), 3.07 (s, 3H), 1.60 (d, *J* = 7.0 Hz, 3H); ^13^C NMR (151 MHz, DMSO-*d*_6_) δ 156.5, 153.0, 151.0, 141.7, 141.6, 133.2, 130.0, 128.4 (2C), 126.90, 126.86 (2C), 126.8 (2C), 124.5 (2C), 103.5, 98.7, 62.6, 52.1, 31.6, 16.2; HRMS (ES+, *m*/*z*): found 359.1874, calcd for C_22_H_23_N_4_O, [M + H]^+^, 359.1872.

##### (*R*)-(4-(4-((1-(4-(*tert*-Butyl)phenyl)ethyl)(methyl)amino)-7*H*-pyrrolo[2,3-*d*]pyrimidin-6-yl)phenyl)methanol (**19**)

Compound **90** (15 mg, 0.028 mmol) was treated as described in General Procedure C, using NaHCO_3_ in the second step. The crude product was purified by silica-gel column chromatography (CH_2_Cl_2_/MeOH, 9:1, *R_f_* = 0.32). Drying gave 8.5 mg (0.021 mmol, 73%) of a yellow solid, mp 259–263 °C (decomp.); [α]_D_^20^ = +72.8 (*c* 1.00, CHCl_3_); ^1^H NMR (400 MHz, DMSO-*d*_6_) δ 12.20 (br s, 1H), 8.17 (s, 1H), 7.90–7.85 (m, 2H), 7.44–7.38 (m, 2H), 7.37–7.32 (m, 4H), 7.30–7.24 (m, 2H), 7.11 (s, 1H), 6.50–6.42 (m, 1H), 5.19 (t, *J* = 5.7 Hz, 1H), 4.51 (d, *J* = 5.7 Hz, 2H), 3.08 (s, 3H), 1.60 (d, *J* = 7.0 Hz, 3H), 1.25 (s, 9H); ^13^C NMR (100 MHz, DMSO-*d*_6_) δ 156.6, 153.1, 151.1, 149.4, 141.6, 138.5, 133.1, 130.0, 127.0 (2C), 126.8 (2C), 125.3 (2C), 124.5 (2C), 103.5, 99.1, 62.6, 52.1, 34.5, 31.6, 31.5 (3C), 16.2; IR (neat, cm^–1^): 3346 (w), 3130 (w), 2959 (w), 2867 (w), 1597 (s), 1489 (m), 1397 (m), 1360 (m), 1311 (m), 1167 (m), 898 (m), 824 (m), 802 (m), 765 (s); HRMS (ASAP+, *m*/*z*): found 415.2487, calcd for C_26_H_31_N_4_O, [M + H]^+^, 415.2492.

##### *N*-(6-(4-(Hydroxymethyl)phenyl)-7*H*-pyrrolo[2,3-*d*]pyrimidin-4-yl)benzamide (**20**)

Compound **121** (30 mg, 0.064 mmol) was treated as described in General Procedure C, using NaHCO_3_ in the second step. The crude product was purified by silica-gel column chromatography (CH_2_Cl_2_/MeOH–92.5:7.5, *R_f_* = 0.17). Drying gave 18 mg (0.053 mmol, 83%) of a yellow solid, mp >252 °C (decomp.); HPLC purity > 99% (method B); ^1^H NMR (400 MHz, DMSO-*d*_6_) δ 12.53 (s, 1H), 11.07 (s, 1H), 8.56 (s, 1H), 8.13–8.06 (m, 2H), 7.91–7.85 (m, 2H), 7.69–7.60 (m, 1H), 7.60–7.52 (m, 2H), 7.44–7.38 (m, 2H), 6.97 (s, 1H), 5.25 (t, *J* = 5.7 Hz, 1H), 4.54 (d, *J* = 5.8 Hz, 2H). ^13^C NMR (101 MHz, DMSO-*d*_6_) δ 165.6, 154.5, 154.5, 150.3, 142.9, 136.7, 133.6, 132.3, 129.3, 128.5, 128.4, 127.0, 125.2, 110.1, 98.8, 62.6.; IR (neat, cm^–1^): 3309 (w), 3140 (w), 3061 (w), 3002 (w), 1705 (m), 1593 (m), 1510 (s), 1489 (s), 1455 (m), 1347 (m), 1258 (s), 1037 (m), 1018 (m), 780 (s), 762 (m), 692 (s), 647 (m), 638 (m); HRMS (ES+, *m*/*z*): found 345.1353, calcd for C_20_H_17_N_4_O_2_, [M + H]^+^, 345.1352.

##### (4-(4-(Benzyl(methyl-*d*_3_)amino)-7*H*-pyrrolo[2,3-*d*]pyrimidin-6-yl)phenyl)methanol (**21**)

Compound **91** (148 mg, 0.310 mmol) was treated as described in General Procedure C, using NaHCO_3_ in the second step. The crude product was purified by silica-gel column chromatography (CH_2_Cl_2_/THF/MeOH–87.5: 12.5:5, *R_f_* = 0.15). Drying gave 103 mg (0.297 mmol, 96%) of a white solid, mp 255–258 °C; HPLC purity: 96.5 (method A); ^1^H NMR (600 MHz, DMSO-*d*_6_) δ 12.15 (s, 1H), 8.14 (s, 1H), 7.83–7.78 (m, 2H), 7.37–7.31 (m, 4H), 7.29–7.26 (m, 2H), 7.26–7.22 (m, 1H), 7.03 (s, 1H), 5.19 (t, *J* = 5.7 Hz, 1H), 5.04 (s, 2H), 4.51 (d, *J* = 5.7 Hz, 2H); ^13^C NMR (151 MHz, DMSO-*d*_6_) δ 156.4, 152.9, 151.1, 141.7, 138.5, 133.3, 130.0, 128.5 (2C), 127.0 (2C), 126.9, 126.8 (2C), 124.5 (2C), 103.2, 98.5, 62.6, 52.6, 36.6; HRMS (ES+, *m*/*z*): found 348.1902, calcd for C_21_H_18_D_3_N_4_O, [M + H]^+^, 348.1904.

##### (4-(4-(Methyl(2-methylbenzyl)amino)-7*H*-pyrrolo[2,3-*d*]pyrimidin-6-yl)phenyl)methanol (**22**)

Compound **92** (122 mg, 0.251 mmol) was treated as described in General Procedure C, using NaHCO_3_ in the second step. The crude product was purified by silica-gel column chromatography (CH_2_Cl_2_/MeOH–9:1, *R_f_* = 0.33). Drying gave 72 mg (0.200 mmol, 80%) of a white solid, mp >300 °C (decomp.); HPLC purity >99 (method A); ^1^H NMR (600 MHz, DMSO-*d*_6_) δ 12.14 (s, 1H), 8.12 (s, 1H), 7.80–7.76 (m, 2H), 7.37–7.32 (m, 2H), 7.24–7.20 (m, 1H), 7.18–7.13 (m, 1H), 7.13–7.09 (m, 1H), 7.01–6.97 (m, 1H), 6.95 (s, 1H), 5.19 (t, *J* = 5.7 Hz, 1H), 5.00 (s, 2H), 4.50 (d, *J* = 5.7 Hz, 2H), 3.38 (s, 3H), 2.31 (s, 3H); ^13^C NMR (151 MHz, DMSO-*d*_6_) δ 156.5, 152.9, 151.1, 141.7, 135.9, 135.6, 133.3, 130.2, 130.0, 126.8 (2C), 126.6, 125.9, 125.8, 124.5 (2C), 103.3, 98.4, 62.6, 51.1, 37.5, 18.7; HRMS (ASAP+, *m*/*z*): found 359.1867, calcd for C_22_H_23_N_4_O, [M + H]^+^, 359.1872.

##### (4-(4-(Methyl(3-methylbenzyl)amino)-7*H*-pyrrolo[2,3-*d*]pyrimidin-6-yl)phenyl)methanol (**23**)

Compound **93** (249 mg, 0.509 mmol) was treated as described in General Procedure C, using NaHCO_3_ in the second step. The crude product was purified by silica-gel column chromatography (CH_2_Cl_2_/MeOH–19:1). Drying gave 176 mg (0.491 mmol, 96%) of a white solid, mp 266–270 °C; HPLC purity: 99% (method B); ^1^H NMR (600 MHz, DMSO-*d*_6_) δ 12.14 (s, 1H), 8.14 (s, 1H), 7.82–7.78 (m, 2H), 7.37–7.32 (m, 2H), 7.24–7.18 (m, 1H), 7.11–7.08 (m, 1H), 7.08–7.04 (m, 2H), 7.03 (s, 1H), 5.19 (t, *J* = 5.7 Hz, 1H), 5.01 (s, 2H), 4.50 (d, *J* = 5.7 Hz, 2H), 3.35 (s, 3H), 2.27 (s, 3H); ^13^C NMR (151 MHz, DMSO-*d*_6_) δ 156.4, 152.9, 151.1, 141.7, 138.4, 137.6, 133.3, 130.0, 128.4, 127.58, 127.55, 126.8 (2C), 124.5 (2C), 124.1, 103.2, 98.5, 62.6, 52.6, 37.3, 21.1; HRMS (ASAP+, *m*/*z*): found 359.1868, calcd for C_22_H_23_N_4_O, [M + H]^+^, 359.1872.

##### (4-(4-(Methyl(4-methylbenzyl)amino)-7*H*-pyrrolo[2,3-*d*]pyrimidin-6-yl)phenyl)methanol (**24**)

Compound **94** (133 mg, 0.273 mmol) was treated as described in General Procedure C, using NaHCO_3_ in the second step. The crude product was purified by silica-gel column chromatography (CH_2_Cl_2_/MeOH–9:1). Drying gave 63 mg (0.175 mmol, 64%) of a white solid, mp 263–265 °C; HPLC purity: 94% (method A); ^1^H NMR (600 MHz, DMSO-*d*_6_) δ 12.14 (s, 1H), 8.14 (s, 1H), 7.82–7.78 (m, 2H), 7.36–7.32 (m, 2H), 7.19–7.15 (m, 2H), 7.14–7.10 (m, 2H), 7.02 (s, 1H), 5.19 (t, *J* = 5.7 Hz, 1H), 5.00 (s, 2H), 4.50 (d, *J* = 5.7 Hz, 2H), 3.32 (s, 3H), 2.26 (s, 3H); ^13^C NMR (151 MHz, DMSO-*d*_6_) δ 156.4, 152.9, 151.1, 141.7, 135.9, 135.3, 133.3, 130.0, 129.1 (2C), 127.0 (2C), 126.8 (2C), 124.5 (2C), 103.2, 98.5, 62.6, 52.4, 37.2, 20.6; HRMS (ASAP+, *m*/*z*): found 359.1870, calcd for C_22_H_23_N_4_O, [M + H]^+^, 359.1872.

##### (4-(4-(Methyl(pyridin-2-ylmethyl)amino)-7*H*-pyrrolo[2,3-*d*]pyrimidin-6-yl)phenyl)methanol (**25**)

Compound **95** (126 mg, 0.266 mmol) was treated as described in General Procedure C, using NaHCO_3_ in the second step. The crude product was purified by silica-gel column chromatography (CH_2_Cl_2_/MeOH–9:1). Drying gave 67 mg (0.193 mmol, 73%) of a white solid, mp 276–279 °C (decomp.); HPLC purity > 99% (method A); ^1^H NMR (600 MHz, DMSO-*d*_6_) δ 12.14 (s, 1H), 8.56–8.51 (m, 1H), 8.11 (s, 1H), 7.82–7.77 (m, 2H), 7.75–7.69 (m, 1H), 7.37–7.33 (m, 2H), 7.28–7.24 (m, 1H), 7.24–7.20 (m, 1H), 7.03 (s, 1H), 5.20 (t, *J* = 5.7 Hz, 1H), 5.11 (s, 2H), 4.51 (d, *J* = 5.7 Hz, 2H), 3.48 (s, 3H); ^13^C NMR (151 MHz, DMSO-*d*_6_) δ 158.3, 156.4, 152.9, 151.0, 149.2, 141.7, 136.8, 133.3, 129.9, 126.8 (2C), 124.5 (2C), 122.1, 120.9, 103.4, 98.5, 62.6, 55.0, 38.2; HRMS (ASAP+, *m*/*z*): found 346.1663, calcd for C_20_H_20_N_5_O, [M + H]^+^, 346.1668.

##### (4-(4-(Methyl(pyridin-3-ylmethyl)amino)-7*H*-pyrrolo[2,3-*d*]pyrimidin-6-yl)phenyl)methanol (**26**)

Compound **96** (31 mg, 0.065 mmol) was treated as described in General Procedure C, using NaHCO_3_ in the second step. The crude product was purified by silica-gel column chromatography (CH_2_Cl_2_/MeOH–9:1). Drying gave 21 mg (0.060 mmol, 92%) of a white solid, mp 242–244 °C (decomp.); HPLC purity > 99 (method A); ^1^H NMR (400 MHz, DMSO-*d*_6_) δ 12.17 (s, 1H), 8.57–8.52 (m, 1H), 8.49–8.43 (m, 1H), 8.15 (s, 1H), 7.85–7.79 (m, 2H), 7.71–7.64 (m, 1H), 7.38–7.30 (m, 3H), 7.10–7.05 (m, 1H), 5.19 (t, *J* = 5.7 Hz, 1H), 5.06 (s, 2H), 4.51 (d, *J* = 5.7 Hz, 2H), 3.41 (s, 3H); ^13^C NMR (101 MHz, DMSO-*d*_6_) δ 156.3, 152.9, 151.0, 148.8, 148.2, 141.7, 134.9, 134.1, 133.5, 129.9, 126.8 (2C), 124.5 (2C), 123.6, 103.4, 98.4, 62.6, 50.4, 37.5; IR (neat, cm^–1^): 3227 (w), 3101 (w), 2954 (w), 2850 (w), 2739 (w), 1569 (s), 1558 (s), 1504 (m), 1416 (s), 1315 (m), 1292 (m), 1255 (m), 1062 (m), 1016 (m), 929 (s), 801 (s), 759 (s); HRMS (ASAP+, *m*/*z*): found 346.1673, calcd for C_20_H_20_N_5_O, [M + H]^+^, 346.1668.

##### (4-(4-(Methyl(pyridin-4-ylmethyl)amino)-7*H*-pyrrolo[2,3-*d*]pyrimidin-6-yl)phenyl)methanol (**27**)

Compound **97** (123 mg, 0.259 mmol) was treated as described in General Procedure C, using NaHCO_3_ in the second step. The crude product was purified by silica-gel column chromatography (CH_2_Cl_2_/MeOH–9:1, *R_f_* = 0.21). Drying gave 71 mg (0.206 mmol, 79%) of a colorless solid, mp 252–255 °C (decomp); HPLC purity > 99 (method A); ^1^H NMR (400 MHz, DMSO-*d*_6_) δ 12.18 (s, 1H), 8.52–8.46 (m, 2H), 8.12 (s, 1H), 7.85–7.78 (m, 2H), 7.38–7.32 (m, 2H), 7.28–7.22 (m, 2H), 7.05 (s, 1H), 5.20 (t, *J* = 5.7 Hz, 1H), 5.06 (s, 2H), 4.51 (d, *J* = 5.7 Hz, 2H), 3.44 (s, 3H); ^13^C NMR (101 MHz, DMSO-*d*_6_) δ 156.3, 152.9, 151.0, 149.7 (2C), 147.9, 141.8, 133.6, 129.9, 126.8 (2C), 124.5 (2C), 122.0 (2C), 103.3, 98.4, 62.6, 52.1, 37.9; IR (neat, cm^–1^): 3227 (w), 3101 (w), 2954 (w), 2850 (w), 2739 (w), 1569 (s), 1558 (s), 1504 (m), 1416 (s), 1315 (m), 1292 (m), 1255 (m), 1062 (m), 1016 (m), 929 (s), 801 (s), 759 (s); HRMS (ASAP+, *m*/*z*): found 346.1662, calcd for C_20_H_20_N_5_O, [M + H]^+^, 346.1668.

##### (4-(4-(Methyl((6-methylpyridin-2-yl)methyl)amino)-7*H*-pyrrolo[2,3-*d*]pyrimidin-6-yl)phenyl)methanol (**28**)

Compound **98** (62 mg, 0.126 mmol) was treated as described in General Procedure C using NaHCO_3_ in the second step. The crude product was purified by silica-gel column chromatography (CH_2_Cl_2_/7 M NH_3_ in MeOH–92.5:7.5). Drying gave 36 mg (0.100 mmol, 80%) of a colorless solid, mp 269–271 °C (decomp.); HPLC purity > 98.6% (method A); ^1^H NMR (400 MHz, DMSO-*d*_6_) δ 12.14 (s, 1H), 8.11 (s, 1H), 7.81–7.77 (m, 2H), 7.62–7.57 (m, 1H), 7.37–7.33 (m, 2H), 7.13–7.10 (m, 1H), 7.04 (s, 1H), 6.98–6.94 (m, 1H), 5.19 (t, *J* = 5.7 Hz, 1H), 5.05 (s, 2H), 4.51 (d, *J* = 5.7 Hz, 2H), 3.47 (s, 3H), 2.47 (s, 3H); ^13^C NMR (101 MHz, DMSO-*d*_6_) δ 157.7, 157.5, 156.3, 152.9, 151.0, 141.7, 137.1, 133.3, 129.9, 126.9 (2C), 124.5 (2C), 121.4, 117.5, 103.4, 98.5, 62.6, 55.2, 38.2, 24.1; IR (neat, cm^–1^): 3249 (br w), 3080 (w), 2966 (w), 2866 (w), 2738 (w), 1570 (s), 1549 (m), 1519 (m), 1435 (m), 1405 (m), 1319 (m), 1302 (m), 1054 (m), 934 (m), 828 (m), 790 (m), 772 (m), 756 (m); HRMS (ASAP+, *m*/*z*): found 360.1825, calcd for C_21_H_22_N_5_O, [M + H]^+^, 360.1824.

##### (4-(4-((2-Fluorobenzyl)(methyl)amino)-7*H*-pyrrolo[2,3-*d*]pyrimidin-6-yl)phenyl)methanol (**29**)

Compound **99** (99 mg, 0.200 mmol) was treated as described in General Procedure C using NaHCO_3_ in the second step. The crude product was purified by silica-gel column chromatography (CH_2_Cl_2_/7 M NH_3_ in MeOH–92.5:7.5). Drying gave 50 mg (0.137 mmol, 69%) of a colorless solid, mp 269–271 °C (decomp.); HPLC purity: 98.9% (method B); ^1^H NMR (600 MHz, DMSO-*d*_6_) δ 12.16 (s, 1H), 8.13 (s, 1H), 7.84–7.79 (m, 2H), 7.37–7.34 (m, 2H), 7.34–7.28 (m, 1H), 7.26–7.17 (m, 2H), 7.16–7.10 (m, 1H), 7.06 (s, 1H), 5.19 (t, *J* = 5.7 Hz, 1H), 5.08 (s, 2H), 4.51 (d, *J* = 5.8 Hz, 2H), 3.42 (s, 3H); ^13^C NMR (151 MHz, DMSO-*d*_6_) δ 160.4 (d, *J* = 244.0 Hz, 1C), 156.3, 152.9, 151.0, 141.7, 133.5, 129.9, 128.85 (d, *J* = 8.0 Hz, 1C), 128.76 (d, *J* = 4.5 Hz, 1C), 126.8 (2C), 125.1 (d, *J* = 14.7 Hz, 1C), 124.51 (2C), 124.49, 115.3 (d, *J* = 21.2 Hz, 1C), 103.4, 98.3, 62.6, 46.9, 37.7; HRMS (ES+, *m*/*z*): found 363.1622, calcd for C_21_H_20_N_4_OF, [M + H]^+^, 363.1621.

##### 2-(((6-(4-(Hydroxymethyl)phenyl)-7*H*-pyrrolo[2,3-*d*]pyrimidin-4-yl)(methyl)amino)methyl)phenol (**30**)

Compound **100** (45 mg, 0.092 mmol) was treated as described in General Procedure C, using NaHCO_3_ in the second step. The crude product was purified by silica-gel column chromatography (CH_2_Cl_2_/MeOH–100:6). Drying gave 21 mg (0.057 mmol, 62%) of a colorless solid, mp 275–280 °C (decomp.); HPLC purity: 99% (method B); ^1^H NMR (400 MHz, DMSO-*d*_6_) δ 12.18 (s, 1H), 10.36 (s, 1H), 8.15 (s, 1H), 7.82–7.75 (m, 2H), 7.38–7.31 (m, 2H), 7.14–7.06 (m, 2H), 7.01 (s, 1H), 6.88–6.81 (m, 1H), 6.78–6.69 (m, 1H), 5.19 (t, *J* = 5.7 Hz, 1H), 4.87 (s, 2H), 4.51 (d, *J* = 5.6 Hz, 2H), 3.43 (s, 3H); ^13^C NMR (151 MHz, DMSO-*d*_6_) δ 156.1, 155.5, 152.6, 150.7, 141.8, 133.4, 129.9, 128.5, 128.3, 126.9 (2C), 124.5 (2C), 123.9, 119.0, 115.6, 103.4, 98.6, 62.6, 49.1, 37.6; HRMS (ES+, *m*/*z*): found 361.1663, calcd for C_21_H_21_N_4_O_2_, [M + H]^+^, 361.1665.

##### 2-(((6-(4-(Hydroxymethyl)phenyl)-7*H*-pyrrolo[2,3-*d*]pyrimidin-4-yl)(methyl)amino)methyl)-4-methylphenol (**31**)

Compound **101** (100 mg, 0.197 mmol) was treated as described in General Procedure C, using NaHCO_3_ in the second step. The crude product was purified by silica-gel column chromatography (CH_2_Cl_2_/MeOH–95:5). Drying gave 47 mg (0.126 mmol, 64%) of a colorless solid, mp 220.5–223 °C (decomp.). ^1^H NMR (400 MHz, DMSO-*d*_6_) δ 12.19 (s, 1H), 10.13 (s, 1H), 8.15 (s, 1H), 7.82–7.76 (m, 2H), 7.38–7.32 (m, 2H), 7.04–6.99 (m, 1H), 6.93–6.86 (m, 2H), 6.77–6.70 (m, 1H), 5.20 (t, *J* = 5.7 Hz, 1H), 4.83 (s, 2H), 4.51 (d, *J* = 5.7 Hz, 2H), 3.43 (s, 3H), 2.14 (s, 3H); ^13^C NMR (101 MHz, DMSO-*d*_6_) δ 156.1, 153.1, 152.6, 150.7, 141.7, 133.4, 129.9, 128.7 (2C), 127.3, 126.8 (2C), 124.5 (2C), 123.6, 115.5, 103.3, 98.6, 62.6, 49.0, 37.6, 20.3; IR (neat, cm^–1^): 3289 (br w), 3220 (w), 3113 (w), 2990 (w), 2886 (w), 2746 (w), 1599 (m), 1579 (s), 1517 (m), 1501 (m), 1406 (w), 1344 (w), 1318 (w), 1299 (w), 1069 (w), 1014 (w), 925 (m), 774 (s); HRMS (ASAP+, *m*/*z*): found 375.1814, calcd for C_22_H_23_N_4_O_2_, [M + H]^+^, 375.1821.

##### (*S*)-2-((6-(4-(Hydroxymethyl)phenyl)-7*H*-pyrrolo[2,3-*d*]pyrimidin-4-yl)(methyl)amino)-2-phenylethan-1-ol (**32**)

Compound **102** (135 mg, 0.268 mmol) was treated as described in General Procedure C, using NaHCO_3_ in the second step. The crude product was purified by silica-gel column chromatography (CH_2_Cl_2_/MeOH–9:1). Drying gave 26 mg (0.068 mmol, 25%) of a beige solid. TLC (silica, CH_2_Cl_2_/MeOH–8.75:1.25): *R_f_* = 0.07; HPLC purity: 97.8 (method A); ^1^H NMR (600 MHz, DMSO-*d*_6_) δ 12.12 (s, 1H), 8.14 (s, 1H), 7.84–7.80 (m, 2H), 7.39–7.31 (m, 6H), 7.29–7.23 (m, 1H), 7.07 (s, 1H), 6.29 (s, 1H), 5.19 (t, *J* = 5.7 Hz, 1H), 4.99 (t, *J* = 5.3 Hz, 1H), 4.51 (d, *J* = 5.6 Hz, 2H), 4.12–4.05 (m, 1H), 4.02–3.95 (m, 1H), 3.21 (s, 3H); ^13^C NMR (151 MHz, DMSO-*d*_6_) δ 157.3, 153.0, 150.9, 141.6, 139.1, 133.1, 130.0, 128.4 (2C), 127.4 (2C), 127.0, 126.8 (2C), 124.5 (2C), 103.6, 98.8, 62.6, 60.6, 58.9, 32.4; HRMS (ASAP+, *m*/*z*): found 375.1824, calcd for C_22_H_23_N_4_O_2_, [M + H]^+^, 375.1821.

##### (*R*)-2-((6-(4-(Hydroxymethyl)phenyl)-7*H*-pyrrolo[2,3-*d*]pyrimidin-4-yl)(methyl)amino)-2-phenylethan-1-ol (**33**)

Compound **103** (110 mg, 0.217 mmol) was treated as described in General Procedure C using NaHCO_3_ in the second step. The crude product was purified by silica-gel column chromatography (CH_2_Cl_2_/MeOH–9:1). Drying gave 24 mg (0.063 mmol, 29%) of a beige solid. TLC (silica, CH_2_Cl_2_/MeOH–8.75:1.25): *R_f_* = 0.07; ^1^H NMR (600 MHz, DMSO-*d*_6_) δ 12.12 (s, 1H), 8.14 (s, 1H), 7.82 (d, *J* = 8.0 Hz, 2H), 7.38–7.32 (m, 6H), 7.28–7.24 (m, 1H), 7.07 (s, 1H), 6.29 (s, 1H), 5.19 (t, *J* = 5.7 Hz, 1H), 4.99 (t, *J* = 5.3 Hz, 1H), 4.51 (d, *J* = 5.6 Hz, 2H), 4.11–4.05 (m, 1H), 4.02–3.96 (m, 1H), 3.21 (s, 3H); HRMS (ASAP+, *m*/*z*): found 375.1820, calcd for C_22_H_23_N_4_O_2_, [M + H]^+^, 375.1821.

##### *N*-Methyl-*N*-(3-methylbenzyl)-6-phenyl-7*H*-pyrrolo[2,3-*d*]pyrimidin-4-amine (**35**)

Compound **104** (196 mg, 0,43 mmol) was treated as described in General Procedure C using NaHCO_3_ in the second step. The crude product was purified by silica-gel column chromatography (CH_2_Cl_2_/MeOH–93:7, *R_f_* = 0.44). This gave 119 mg (0.36 mmol, 83%) of a white powder, mp 234–236 °C; HPLC purity > 99 (method A); ^1^H NMR (600 MHz, DMSO-*d*_6_) δ 12.19 (s, 1H), 8.16 (s, 1H), 7.86 (d, *J* = 7.8 Hz, 2H), 7.43 (t, *J* = 7.7 Hz, 2H), 7.29 (t, *J* = 7.4 Hz, 1H), 7.23 (t, *J* = 7.6 Hz, 1H), 7.15–7.04 (m, 4H), 5.03 (s, 2H), 3.37 (s, 4H), 2.28 (s, 3H); ^13^C NMR (151 MHz, DMSO-*d*_6_) δ 156.5, 153.0, 151.2, 138.4, 137.7, 133.2, 131.5, 128.8 (2C), 128.5, 127.6 (2C), 127.3, 124.7 (2C), 124.2, 103.3, 98.9, 52.7, 39.5, 37.4, 21.1; IR (neat, cm^–1^): 3109 (s, w), 3025 (s, w), 2989 (w), 2906 (s), 2855 (w), 1568 (s), 1539 (s), 1510 (s), 1410 (s), 1321(s), 937(s), 777 (s), 743 (s), 692 (s); HRMS (ES+, *m*/*z*): found 329.1772, calcd for C_21_H_20_N_4_, [M + H]^+^, 329.1687.

##### 4-(4-(Methyl(3-methylbenzyl)amino)-7*H*-pyrrolo[2,3-*d*]pyrimidin-6-yl)phenol (**36**)

Compound **105** (196 mg, 0.41 mmol) was treated as described in General Procedure C, using NaHCO_3_ in the second step. The crude product was purified by silica-gel column chromatography (CH_2_Cl_2_/MeOH/AcOH–93:6:1, *R_f_* = 0.30). This gave 164 mg (0.36 mmol, 87%) of a white powder, mp 241–243 °C (decomp.); HPLC purity: 99% (method B); ^1^H NMR (600 MHz, DMSO-*d*_6_) δ 11.99 (s, 1H), 9.59 (s, 1H), 8.12 (s, 1H), 7.66 (d, *J* = 8.6 Hz, 2H), 7.22 (t, *J* = 7.6 Hz, 1H), 7.09 (s, 1H), 7.04–7.07 (m, 2H), 6.83 (s, 1H), 6.82–6.77 (m, 2H), 5.00 (s, 2H), 3.34 (s, 3H), 2.28 (s, 3H); ^13^C NMR (151 MHz, DMSO-*d*_6_) δ 157.0, 156.2, 152.7, 150.6, 138.5, 137.6, 133.8, 128.4, 127.6, 127.5, 126.2 (2C), 124.1, 122.6, 115.5 (2C), 103.3, 96.6, 52.6, 37.2, 21.1; IR (neat, cm^–1^): 3103 (w), 2917 (w), 2849 (w), 1712 (s), 1571 (s), 1501 (s), 1316 (s), 936 (s), 832 (s), 767 (s); HRMS (ES+, *m*/*z*): found 345.1826, calcd for C_21_H_20_N_4_O, [M + H]^+^, 345.163711.

##### 6-(4-Fluorophenyl)-*N*-methyl-*N*-(3-methylbenzyl)-7*H*-pyrrolo[2,3-*d*]pyrimidin-4-amine (**37**)

Compound **106** (234 mg, 0.49 mmol) was treated as described in General Procedure C, using NaHCO_3_ in the second step. The crude product was purified by silica-gel column chromatography (CH_2_Cl_2_/MeOH–93:7, *R_f_* = 0.56). This gave 134 mg (0.38 mmol, 77%) of a white powder, mp 247–249 °C (decomp.); HPLC purity: 97.7 (method A); ^1^H NMR (600 MHz, DMSO-*d*_6_) δ 12.20 (s, 1H), 8.17 (s, 1H), 7.94–7.87 (m, 2H), 7.31–7.24 (m, 2H), 7.23 (t, *J* = 7.6 Hz, 1H), 7.13–7.03 (m, 4H), 5.03 (s, 2H), 3.37 (s, 3H), 2.28 (s, 3H); ^13^C NMR (151 MHz, DMSO-*d*_6_) δ 162.1 (d, *J* = 247 Hz), 156.5, 153.0, 151.2, 138.4, 137.6, 132.3, 128.4, 128.2 (d, *J* = 3.2 Hz), 127.6 (2C), 126.6 (d, *J* = 8.7 Hz, 2C), 124.12, 115.6 (d, *J* = 22 Hz, 2C), 103.2, 98.9, 52.6, 39.5, 37.3, 21.1; ^19^F NMR (376 MHz, DMSO-*d*_6_, C_6_F_6_) δ −116.1 (s); IR (neat, cm^–1^): 3108 (w), 2965 (w), 2969 (w), 2734(s), 1571 (s), 1543 (s), 1510(s), 1499(s), 1410 (s), 1318(s), 1228 (s), 936(s), 831 (s), 774 (s), 760(s); HRMS (ES+, *m*/*z*): found 347.1677, calcd for C_21_H_19_FN_4_, [M + H]^+^, 347.1593.

##### 6-(3-Fluoro-4-methoxyphenyl)-*N*-methyl-*N*-(3-methylbenzyl)-7*H*-pyrrolo[2,3-*d*]pyrimidin-4-amine (**38**)

Compound **107** (50 mg, 0.1 mmol) was treated as described in General Procedure C using NaHCO_3_ in the second step. The crude product was purified by silica-gel column chromatography (CH_2_Cl_2_/MeOH–93:7, *R_f_* = 0.73). This gave 35 mg (0.09 mmol, 94%) of a white powder, mp 236–238 °C (decomp.); HPLC purity: 96.7 (method A); ^1^H NMR (600 MHz, DMSO-*d*_6_) δ 12.13 (s, 1H), 8.14 (s, 1H), 7.76 (dd, *J* = 12.9, 2.1 Hz, 1H), 7.65 (dd, *J* = 8.64, 1.3 Hz 1H), 7.22 (m, 2H), 7.09 (s, 1H), 7.03–7.07 (m, 3H), 5.01 (s, 2H), 3.87 (s, 3H), 3.35 (s, 3H), 2.27 (s, 3H); ^13^C NMR (151 MHz, DMSO-*d*_6_) δ 156.4, 152.9, 151.6 (d, *J* = 245 Hz), 151.0, 146.3 (d, *J* = 10.9 Hz), 138.4, 137.6, 132.1, 128.4, 127.6 (2C), 124.8 (d, *J* = 7.6 Hz), 124.1, 121.0 (d, *J* = 2.7 Hz), 114.2 (d, *J* = 1.6 Hz), 112.4 (d, *J* = 20 Hz), 103.2, 98.6, 56.1, 52.5, 37.3, 21.1; ^19^F NMR (376 MHz, DMSO-*d*_6_, C_6_F_6_) δ −137.5 (s); IR (neat, cm^–1^): 3130 (w), 3001 (w), 2969 (w), 2914 (w), 2876 (w), 2793 (w), 1601 (s),1547 (s),1409 (s), 1324 (s), 1274 (s), 1073 (s), 935 (s), 863 (s), 764 (s). HRMS (ES+, *m*/*z*): found 377.1783, calcd for C_22_H_21_FN_4_O, [M + H]^+^, 376.1770.

##### *N*-Methyl-*N*-(3-methylbenzyl)-6-(pyridin-3-yl)-7*H*-pyrrolo[2,3-*d*]pyrimidin-4-amine (**39**)

The precursor **108** (190 mg, 0.41 mmol) was treated as described in General Procedure C, using NaHCO_3_ in the second step. The crude product was purified by silica-gel column chromatography (CH_2_Cl_2_/MeOH–97:3, *R_f_* = 0.35). This gave 103 mg (0.31 mmol, 75%) of a white powder, mp 220–223 °C (decomp.); HPLC purity > 99 (method A); ^1^H NMR (600 MHz, DMSO-*d*_6_) δ 12.34 (s, 1H), 9.10 (d, *J* = 2.3 Hz, 1H), 8.47 (dd, *J* = 4.7, 1.5 Hz, 1H), 8.20–8.22 (m, 1H), 8.18 (s, 1H) 7.45 (dd, *J* = 8.0, 4.7 Hz, 1H), 7.25 (s, 1H), 7.22 (t, *J* = 7.6 Hz, 1H), 7.10 (s, 1H) 7.04–7.08 (m, 2H), 5.03 (s, 2H), 3.38 (s, 3H), 2.28 (s, 3H); ^13^C NMR (151 MHz, DMSO-*d*_6_) δ 156.6, 153.2, 151.6, 148.0, 146.1, 138.3, 137.6, 131.6, 130.1, 128.5, 127.6 (2C), 127.5, 124.2, 123.8, 103.2, 100.3, 52.6, 37.5, 21.1; IR (neat, cm^–1^) v- 3130 (w), 2901 (w), 1576 (s), 1406 (m), 1207 (m), 1151 (m), 937 (m), 774 (m); HRMS (ES+, *m*/*z*): found 330.1725, calcd for C_20_H_19_N_5_, [M + H]^+^, 330.16404.

##### 6-(4-(2-(2-(2-Methoxyethoxy)ethoxy)ethoxy)phenyl)-*N*-methyl-*N*-(3-methylbenzyl)-7*H*-pyrrolo[2,3-*d*]pyrimidin-4-amine (**40**)

The precursor **109** (87 mg, 0.140 mmol) was treated as described in General Procedure C, using NaHCO_3_ in the second step. The product was purified twice by silica-gel column chromatography (CH_2_Cl_2_/MeOH–97.5:2.5, *R_f_* = 0.38). This gave 39 mg (0.079 mmol, 57%) of a solid; HPLC purity > 99 (method A); ^1^H NMR (400 MHz, DMSO-*d*_6_) δ 12.07 (s, 1H), 8.13 (s, 1H), 7.80–7.76 (m, 2H), 7.24–7.19 (m, 1H), 7.11–7.04 (m, 3H), 7.01–6.98 (m, 2H), 6.92 (s, 1H), 5.01 (s, 2H), 4.15–4.11 (m, 2H), 3.78–3.74 (m, 2H), 3.62–3.50 (m, 6H), 3.45–3.42 (m, 2H), 3.35 (s, 3H), 3.24 (s, 3H), 2.27 (s, 3H); ^13^C NMR (100 MHz, DMSO-*d*_6_) δ 159.6, 156.7, 153.4, 153.3, 138.9, 138.0, 135.2, 129.2 (2C), 128.9, 128.0, 126.6, 124.6, 115.2 (2C), 109.4, 103.7, 71.8 (C33), 70.4, 70.2, 70.1, 69.4, 67.7, 58.6, 53.2, 37.8, 21.6; HRMS (ASCI/ASAP, *m*/*z*): found 491.2657, calcd for C_28_H_34_N_4_O_4_, [M + H]^+^, 491.2658.

##### *N*-Methyl-*N*-(3-methylbenzyl)-6-(4-(trifluoromethyl)phenyl)-7*H*-pyrrolo[2,3-*d*]pyrimidin-4-amine (**41**)

Compound **110** (102 mg, 0.19 mmol) was treated as described in General Procedure C, using NaHCO_3_ in the second step. The crude product was purified by silica-gel column chromatography (CH_2_Cl_2_/MeOH–95:5, *R_f_* = 0.23). This gave 74 mg (0.18 mmol, 96%) of a white powder, mp 220–223 °C (decomp.); HPLC purity: 97.9 (method A); ^1^H NMR (600 MHz, DMSO-*d*_6_) δ 12.38 (s, 1H), 8.19 (s, 1H), 8.08 (d, *J* = 8.2 Hz, 2H), 7.77 (d, *J* = 8.3 Hz, 2H), 7.29 (s, 1H), 7.22 (t, *J* = 7.6 Hz, 1H), 7.10 (s, 1H), 7.08–7.05 (m, 2H), 5.03 (s, 2H), 3.38 (s, 3H), 2.28 (s, 3H); ^13^C NMR (151 MHz, DMSO-*d*_6_) δ 156.7, 153.3, 151.8, 138.2, 137.6, 135.5, 131.5 128.4, 127.6 (2C), 127.0 (q, *J* = 30 Hz) 126.1 (q, *J* = 273 Hz), 125.6 (q, *J* = 3.2 Hz, 2C), 125.0 (2C), 124.1, 103.3, 101.3, 52.6, 37.4, 2.1; ^19^F NMR (376 MHz, DMSO-*d*_6_, C_6_F_6_) δ -62.6 (s); IR (neat, cm^–1^): 3103 (w), 2966 (w), 2847 (w), 2740 (w), 1568 (s),1548 (s), 1415 (s), 1324 (s), 1162 (s), 1117 (s), 1073 (s), 1062 (s), 935 (s), 840 (s), 772 (s); HRMS (ES+, *m*/*z*): found 397.1643, calcd for C_22_H_19_F_3_N_4_, [M + H]^+^, 397.1561.

##### Methyl 4-(4-(Methyl(3-methylbenzyl)amino)-7*H*-pyrrolo[2,3-*d*]pyrimidin-6-yl)benzoate (**42**)

Compound **111** (2.57 g, 4.97 mmol) was treated as described in General Procedure C, using NaHCO_3_ in the second step. The crude product was purified by silica-gel column chromatography (CH_2_Cl_2_/MeOH–98:2, *R_f_* = 0.56). This gave 1.22 g (3.16 mmol, 63%) of a white powder, mp 232–233.5 °C (decomp.); HPLC purity > 99 (method A); ^1^H NMR (600 MHz, DMSO-*d*_6_) δ 12.35 (s, 1H), 8.18 (s, 1H), 8.03–7.95 (m, 4H), 7.28 (s, 1H), 7.22 (t, *J* = 7.6 Hz, 1H), 7.10 (s, 1H), 7.04–7.08 (m, 2H), 5.03 (s, 2H), 3.87 (s, 3H), 3.38 (s, 3H), 2.28 (s, 3H); ^13^C NMR (151 MHz, DMSO-*d*_6_) δ 165.9, 156.7, 153.4, 151.9, 138.2, 137.6, 136.0, 131.9, 129.7 (2C), 128.4, 127.7, 127.6 (2C), 124.6 (2C), 124.1, 103.4, 101.4, 52.6, 52.1, 37.5, 21.1; IR (neat, cm^–1^): 3116 (w), 2916 (w), 2849 (w), 2741 (w), 1714 (s), 1565 (s), 1277 (s), 1110 (s), 778 (s); HRMS (ES+, *m*/*z*): found 387.1826, calcd for C_22_H_19_F_3_N_4_, [M + H]^+^, 387.1742.

##### Methyl 5-(4-(4-(Methyl(3-methylbenzyl)amino)-7*H*-pyrrolo[2,3-*d*]pyrimidin-6-yl)phenyl)pentanoate (**43**)

The compound was prepared as described in General Procedure C, starting with **112** (153 mg, 0.267 mmol). The product was purified twice by silica-gel chromatography (CH_2_Cl_2_/MeOH–92:8, *R_f_* = 0.47) and gave 66 mg (0.149 mmol, 56%) of a white solid, mp 162–164 °C. HPLC purity: 91% (method B); ^1^H NMR (400 MHz, DMSO-*d*_6_) δ 12.12 (br s, 1H), 8.14 (s, 1H), 7.78–7.72 (m, 2H), 7.26–7.17 (m, 3H), 7.11–7.02 (m, 3H), 6.99 (ap d, *J* = 2.2 Hz, 1H), 5.00 (s, 2H), 3.57 (s, 3H), 3.35 (s, 3H), 2.59 (t, *J* = 7.1 Hz, 2H), 2.33 (t, *J* = 7.0 Hz, 2H), 2.27 (s, 3H), 1.65–1.49 (m, 4H); ^13^C NMR (101 MHz, DMSO*-d*_6_) δ 173.3, 156.4, 152.9, 151.0, 141.2, 138.4, 137.6, 133.4, 129.1, 128.7 (2C), 128.4, 127.6 (2C), 124.7 (2C), 124.1, 103.2, 98.3, 52.6, 51.2, 37.3, 34.4, 33.1, 30.1, 24.0, 21.1; IR (neat, cm^–1^): 3205 (w), 3110 (w), 3021 (w), 2945 (w), 2857 (w), 1735 (s), 1567 (s), 1435 (m), 1415 (m), 1320 (m), 1295 (w), 1248 (m), 1170 (w), 1070 (w), 837 (m), 770 (w), 694 (w); HRMS (ASAP+, *m*/*z*): found 443.2448, calcd for C_27_H_31_N_4_O_2_, [M + H]^+^, 443.2447.

##### 7-(4-(4-(Methyl(3-methylbenzyl)amino)-7*H*-pyrrolo[2,3-*d*]pyrimidin-6-yl)phenyl)-7-oxoheptanoate (**44**)

The compound was prepared as described in General Procedure C, starting with **113** (76 mg, 0.215 mmol). The product was purified by silica-gel chromatography (CH_2_Cl_2_/MeOH–96:4, *R_f_* = 0.37) and gave 50 mg (0.103 mmol, 82%) of a yellow solid, mp 182–184 °C; HPLC purity: 94% (method A); ^1^H NMR (600 MHz, DMSO-*d*_6_) δ 12.34 (br s, 1H), 8.18 (s, 1H), 8.02–7.95 (m, 4H), 7.26 (s, 1H), 7.21 (t, *J* = 7.6 Hz, 1H), 7.10–7.04 (m, 3H), 5.03 (s, 2H), 3.58 (s, 3H), 3.38 (s, 3H), 3.01 (t, *J* = 7.2 Hz, 2H), 2.31 (t, *J* = 7.4 Hz, 2H), 2.27 (s, 3H), 1.66–1.53 (m, 4H), 1.37–1.29 (m, 2H); ^13^C NMR (151 MHz, DMSO-*d*_6_) δ 199.1, 173.3, 156.7, 153.4, 151.8, 138.2, 137.6, 135.7, 134.9, 132.0, 128.5 (2C), 128.4, 127.6 (2C), 124.5 (2C), 124.1, 103.4, 101.3, 52.6, 51.2, 37.6, 37.5, 33.2, 28.1, 24.3, 23.5, 21.1; IR (neat, cm^–1^): 3206 (w), 3104 (w), 3020 (w), 2944 (w), 2866 (w), 1736 (s), 1675 (s) 1568 (s), 1405 (m), 1324 (m), 1188 (w), 1073 (w), 883 (w), 816 (w), 780 (m), 693 (w); HRMS (ASAP+, *m*/*z*): found 485.2551, calcd for C_29_H_33_N_4_O_3_, [M + H]^+^, 485.2553.

##### 4-(4-(Methyl(3-methylbenzyl)amino)-7*H*-pyrrolo[2,3-*d*]pyrimidin-6-yl)benzoic Acid (**45**)

Methyl ester **42** (800 mg, 2.07 mmol) was dissolved in MeOH/H_2_O/THF (vol ratio: 2:1:1, 100 mL), and LiOH (240 mg, 8.83 mmol, 4.3 equiv) was added. The reaction mixture was stirred at 50 °C for 24 h. Then, the solvents were removed under reduced pressure, and the resulting mixture was poured into a beaker containing distilled water (100 mL). The pH of the solution was adjusted to ca 3 by adding 2 M HCl. The solid material formed was isolated by filtration and then washed with cold distilled water and dried. This gave 739 mg (1.98 mmol, 95%) of an off-white powder, mp 241–243 °C (decomp.); HPLC purity >99% (method B); ^1^H NMR (600 MHz, DMSO-*d*_6_) δ 12.32 (s, 1H), 8.17 (s, 1H), 7.91 −7.98 (m, *J* = 8.4 Hz, 4H), 7.25 (s, 1H), 7.21 (t, *J* = 7.6 Hz, 1H), 7.10 (s, 1H), 7.08–7.05 (m, 2H), 5.03 (s, 2H), 3.37 (s, 3H), 2.27 (s, 3H); ^13^C NMR (MHz, DMSO-*d*_6_) δ 167.0, 156.6, 153.3, 151.7, 138.2, 137.6, 135.3, 132.1, 129.7 (3C), 128.4, 127.6 (2), 124.4 (2C), 124.1, 103.3, 101.0, 52.5, 37.4, 21.0; IR (neat, cm^–1^): 3360 (w), 2921 (w), 2621 (w), 1704 (s), 1629 (s),1592 (s), 1411 (s), 1086 (s), 759 (s), 691 (s); HRMS (ES+, *m*/*z*): found 373.1668, calcd for C_22_H_20_N_4_O_2_, [M + H]^+^, 373.1586.

##### 2-Fluoro-4-(4-(methyl(3-methylbenzyl)amino)-7*H*-pyrrolo[2,3-*d*]pyrimidin-6-yl)benzoic Acid (**46**)

The SEM-protected precursor **114** (35 mg, 0.067 mmol) was treated as described in General Procedure C, using NaHCO_3_ in the second step. The crude product was purified by silica-gel column chromatography (CH_2_Cl_2_/MeOH/AcOH–95:4:1, *R_f_* = 0.40). This gave 17 mg (0.04 mmol, 66%) of an off-white powder, mp 239–242 °C (decomp.); HPLC purity: 98. 8 (method A); ^1^H NMR (600 MHz, DMSO-*d*_6_) δ 12.34 (s, 1H), 8.18 (s, 1H), 7.78–7.89 (m, 3H), 7.35 (s, 1H), 7.22 (t, *J* = 7.6 Hz, 1H), 7.10 (s, 1H), 7.06 (m, 2H), 5.03 (s, 2H), 3.37 (s, 3H), 2.28 (s, 3H); ^13^C NMR (151 MHz, DMSO-*d*_6_) δ 164.9, 161.5 (d, *J* = 256 Hz) 156.7, 153.4, 152.0, 142. 4, 138.2, 137.6, 136.7 132.3, 131.0, 129.2, 128.4, 127.6 (2C), 124.1, 120.1 (d, *J* = 3.4 Hz), 112.2 (d, *J* = 25 Hz), 103.3, 102.0 53.0, 37.5, 21.1; ^19^F NMR (376 MHz, DMSO-*d*_6_, C_6_F_6_) δ −133.5 (s); IR (neat, cm^–1^): 3362 (w), 2923 (w), 2621 (w), 1723 (s), 1629 (s),1595 (s), 1412 (s), 1086 (m), 759 (s); HRMS (ES+, *m*/*z*): found 391.1738, calcd for C_22_H_20_N_4_O_2_, [M + H]^+^, 391.1492.

##### 3-(4-(4-(Benzyl(methyl)amino)-7*H*-pyrrolo[2,3-*d*]pyrimidin-6-yl)phenyl)propanoic Acid (**47**)

The SEM-protected precursor **115** (53 mg, 0.01 mmol) was treated as described in General Procedure C, using NaHCO_3_ in the second step. The crude product was purified by silica-gel column chromatography (CH_2_Cl_2_/MeOH/AcOH–95:5:1 *R_f_* = 0.43). This gave 27 mg (0.067 mmol, 67%) of an off-white powder, mp 243–246 °C (decomp.); HPLC purity: 98.7 (method B); ^1^H NMR (400 MHz, DMSO-*d*_6_) δ 12.13 (s, 1H), 8.14 (s, 1H), 7.78–7.72 (m, 2H), 7.30–7.24 (m, 2H), 7.24–7.17 (m, 1H), 7.11–7.02 (m, 3H), 7.00 (s, 1H), 5.00 (s, 2H), 3.35 (s, 3H), 2.83 (t, *J* = 7.6 Hz, 2H), 2.55 (t, *J* = 7.6 Hz, 2H), 2.27 (s, 3H); ^13^C NMR (101 MHz, DMSO-*d*_6_) δ 173.8, 156.4, 152.9, 151.1, 140.1, 138.4, 137.6, 133.3, 129.4, 128.7 (2C), 128.4, 127.6 (2C), 124.7 (2C), 124.1, 103.3, 98.4, 52.6, 37.4, 35.1, 30.1, 21.1; IR (neat, cm^–1^): 3099 (w), 3021 (w), 2962 (w), 2922 (w), 2852 (w) 1708 (s), 1577 (s), 1515 (s), 1442 (s), 1348 (s), 1298 (s) 1203 (s), 936 (s), 778 (s); HRMS (ES+, *m*/*z*): found 401.1979, calcd for C_24_H_25_N_4_O_2_ [M + H]^+^ 401.1978.

##### 5-(4-(4-(Methyl(3-methylbenzyl)amino)-7*H*-pyrrolo[2,3-*d*]-pyrimidin-6-yl)phenyl)pentanoic Acid (**48**)

To methyl ester **109** (80.5 mg, 0.182 mmol) was added a solution of conc. H_2_SO_4_ (0.68 mL), 1,4-dioxane (1.96 mL), and water (0.68 mL), and the mixture refluxed for 5 h. The mixture was then concentrated *in vacuo*, water (10 mL) was added, and extracted with EtOAc (4 × 10 mL). The combined organic phases were concentrated *in vacuo*. The crude product was purified by silica-gel chromatography (CH_2_Cl_2_/MeOH/AcOH–91:8:1, *R_f_* = 0.24). This gave 64 mg (0.150 mmol, 82%) of a white solid, mp 216–219 °C; HPLC purity > 99% (method B); ^1^H NMR (600 MHz, DMSO-*d*_6_) δ 12.13 (s, 1H), 11.97 (s, 1H), 8.15 (s, 1H), 7.77–7.73 (m, 2H), 7.25–7.18 (m, 3H), 7.10–7.03 (m, 3H), 6.99 (s, 1H), 5.01 (s, 2H), 3.35 (s, 3H), 2.59 (t, *J* = 7.5 Hz, 2H), 2.27 (s, 3H), 2.23 (t, *J* = 7.3 Hz, 2H), 1.63–1.56 (m, 2H), 1.54–1.48 (m, 2H); ^13^C NMR (151 MHz, DMSO*-d*_6_) δ 174.4, 156.3, 152.8, 150.9, 141.3, 138.4, 137.6, 133.4, 129.0, 128.7 (2C), 128.4, 127.6 (2C), 124.7 (2C), 124.1, 103.2, 98.3, 52.6, 37.3, 34.4, 33.5, 30.2, 24.1, 21.1; IR (neat, cm^–1^): 3099 (w), 3021 (w), 2927 (w), 2858 (w), 1708 (s), 1574 (s), 1462 (m), 1376 (m), 1295 (m), 1194 (w), 1070 (w), 848 (w), 770 (m), 675 (m); HRMS (ASAP+, *m*/*z*): found 429.2285, calcd for C_26_H_29_N_4_O_2_, [M + H]^+^, 429.2291.

##### 6-(4-(Difluoromethyl)phenyl)-*N*-methyl-*N*-(3-methylbenzyl)-7*H*-pyrrolo[2,3-*d*]pyrimidin-4-amine (**49**)

Compound **116** (101 mg, 0.19 mmol) was treated as described in General Procedure C, using NaHCO_3_ in the second step. The crude product was purified by silica-gel column chromatography (CH_2_Cl_2_/MeOH–95:5, *R_f_* = 0.27). This gave 67 mg (0.18 mmol, 93%) of a white powder, mp 234–238 °C; HPLC purity > 95 (method A); ^1^H NMR (600 MHz, DMSO-*d*_6_) δ 12.31 (s, 1H), 8.19 (s, 1H), 8.01 (d, *J* = 8.1 Hz, 2H), 7.62 (d, *J* = 8.1 Hz, 2H), 7.26–7.19 (m, 2H), 7.16–6.90 (m, 4H), 5.04 (s, 2H), 3.39 (s, 3H), 2.29 (s, 3H); ^13^C NMR (151 MHz, DMSO-*d*_6_) δ 156.6, 153.2, 151.6, 138.3, 137.60, 133.9, 132.4 (t, *J* = 22 Hz) 132.1, 128.4, 127.6 (2C), 126.2 (t, *J* = 7 Hz, 2C), 124.9 (2C), 124.1, 114.9 (t, *J* = 240 Hz), 103.3, 100.4, 52.6, 37.4, 21.0; HRMS (ES+, *m*/*z*): found 379.1740, calcd C_22_H_21_F_2_N_4_, [M + H]^+^, 379.1656.

##### 4-(4-(Methyl(3-methylbenzyl)amino)-7*H*-pyrrolo[2,3-*d*]pyrimidin-6-yl)benzenesulfonamide (**50**)

Compound **117** (72 mg, 0.14 mmol) was treated as described in General Procedure C, using NaHCO_3_ in the second step. The crude product was purified by silica-gel column chromatography (CH_2_Cl_2_/MeOH–95:5, *R_f_* = 0.65). This gave 53 mg (0.13 mmol, 94%) of a white powder, mp 213–226 °C; HPLC purity > 99 (method A); ^1^H NMR (600 MHz, DMSO-*d*_6_) δ 12.36 (s, 1H), 8.20 (s, 1H), 8.08–8.04 (m, 2H), 7.87–7.83 (m, 2H), 7.38 (s, 2H), 7.29 (s, 1H), 7.24 (t, *J* = 7.6 Hz, 1H), 7.14–7.06 (m, 3H), 5.05 (s, 2H), 3.40 (s, 3H), 2.30 (s, 3H); ^13^C NMR (151 MHz, DMSO-*d*_6_) δ 156.7, 153.3, 151.8, 142.2, 138.2, 137.6, 134.7, 131.6, 128.4, 127.6 (2C), 126.1 (2C), 124.7 (2C), 124.2, 103.3, 101.1, 52.6, 37.4, 21.1.HRMS (ES+, *m*/*z*): found 408.1495, calcd C_21_H_22_N_5_O_2_S, [M + H]^+^, 408.1415.

##### Methyl 3-(4-(Methyl(3-methylbenzyl)amino)-7*H*-pyrrolo[2,3-*d*]pyrimidin-6-yl)benzoate (**51**)

Compound **118** (233 mg, 0.45 mmol) was treated as described in General Procedure C, using NaHCO_3_ in the second step. The crude product was purified by silica-gel column chromatography (CH_2_Cl_2_/MeOH–95:5, *R_f_* = 0.56). This gave 151 mg (0.39 mmol, 86%) of a white powder, mp 231–235 °C; HPLC purity > 99 (method B); ^1^H NMR (600 MHz, DMSO-*d*_6_) δ 12.35 (s, 1H), 8.43 (s, 1H), 8.16 (s, 1H), 8.12 (dt, *J* = 7.8, 1.4 Hz, 1H), 7.85–7.83 (m, *J* = 7.8, 1.3 Hz, 1H), 7.57 (t, *J* = 7.8 Hz, 1H), 7.21 (t, *J* = 7.6 Hz, 1H), 7.16 (s, 1H), 7.12–7.04 (m, 3H), 5.02 (s, 2H), 3.89 (s, 3H), 3.37 (s, 3H), 2.27 (s, 3H); ^13^C NMR (151 MHz, DMSO-*d*_6_) δ 166.1, 156.6, 153.2, 151.5, 138.4, 137.6, 132.2, 132.1, 130.4, 129.3 (2C), 128.4, 127.7, 127.6 (2C), 125.2, 124.1, 103.2, 99.9, 52.6, 52.2, 37.4, 21.1; HRMS (ES+, *m*/*z*): found 387.1826, calcd C_23_H_23_N_4_O_2_, [M + H]^+^, 387.1742.

##### 3-(4-(Methyl(3-methylbenzyl)amino)-7*H*-pyrrolo[2,3-*d*]pyrimidin-6-yl)benzoic acid (**52**)

Methyl ester **51** (101 mg, 0.26 mmol) was dissolved in MeOH/H_2_O/THF (vol ratio: 2:1:1, 100 mL), and LiOH (31 mg, 1.3 mmol, 5 equiv) was added. The reaction mixture was stirred at 50 °C for 48 h. Then, the solvents were removed under reduced pressure, and the resulting mixture was poured into distilled water (15 mL). The pH of the solution was adjusted to ca 3 by adding 2 M HCl. The solid material formed was isolated by filtration and then washed with cold distilled water and dried. This gave 74 mg (0.2 mmol, 77%) of an off-white powder, mp 239–242 °C (decomp.); HPLC purity > 99 (method A); ^1^H NMR (600 MHz, DMSO-*d*_6_) δ 13.10 (s, 1H), 12.39 (s, 1H), 8.45–8.41 (m, 1H), 8.19 (s, 1H), 8.13–8.08 (m, 1H), 7.88–7.83 (m, 1H), 7.56 (t, *J* = 7.8 Hz, 1H), 7.26–7.20 (m, 1H), 7.17 (s, 1H), 7.12 (s, 1H), 7.11–7.06 (m, 2H), 5.04 (s, 2H), 3.40 (s, 3H), 2.29 (s, 3H); ^13^C NMR (151 MHz, DMSO-*d*_6_) δ 167.2, 156.4, 152.8, 151.1, 138.2, 137.6, 132.4, 131.9, 131.5, 129.1, 128.9, 128.4, 127.9, 127.6 (2C), 125.5, 124.1, 103.2, 99.9, 52.7, 37.5, 21.1; HRMS (ES+, *m*/*z*): found 373.1667, calcd C_22_H_21_N_4_O_2_, [M + H]^+^, 373.1742.

## Data Availability

The PDB code of the co-crystal structure of compound **23** with CSF1R is 8CGC. The authors will release the atomic coordinates and experimental data upon article publication.

## Supporting Information Available

^1^H NMR and ^13^C NMR for final compounds (**1**–**52**); synthetic procedures; biological data; and assay details (PDF)
Molecular formula strings (CSV)

## Abbreviations Used

δ: chemical shift in parts per million downfield from tetramethylsilane
μ: micro
Å: angstrom(s)
°C: degrees Celsius
aa: amino acid
ADME: absorption, distribution, metabolism and excretion
AUC: area under the curve
BSA: bovine serum albumin
DMSO: dimethyl sulfoxide
EGF: epidermal growth factor
EGFR: epidermal growth factor receptor
HPLC: high-performance liquid chromatography
i-Pr: isopropyl
IC_50_: half-maximum inhibitory concentration
*J*: coupling constant (in NMR spectrometry)
*K*_m_: Michaelis constant
M: molar (moles per liter)
m: multiplet (spectral)
MAPK: mitogen-activated protein kinase
mL: milliliter
mM: millimolar (millimoles per liter)
MS: mass spectrometry
PDB: Protein Data Bank
