# Supplementary material for: Synthesis and Development of Highly Selective Pyrrolo[2,3-d]pyrimidine CSF1R Inhibitors Targeting the Autoinhibited Form
Source: J Med Chem. 2023 May 16;66(10):6959–80. doi: 10.1021/acs.jmedchem.3c00428 (PMC10226129; doi:10.1021/acs.jmedchem.3c00428)
Supplement: Supplementary file 1 — jm3c00428_si_001.pdf [file jm3c00428_si_001.pdf]

Supporting Information:

Synthesis and Development of Highly Selective Pyrrolo[2,3-d]pyrimidine CSF1R Inhibitors Targeting the Autoinhibited Form

Thomas Ihle Aarhus[a,b], Frithjof Bjørnstad[a,b], Camilla Wolowczyk[c], Kristin U, Larsen[d], Line Rognstad[b] , Trygve Leithaug[b], Anke Unger[f], Peter Habenberger[f], Alexander Wolff[f], Geir Bjørkøy[c], Clare Pridans[e] Jan Eickhoff[f], Bert Klebl[f], Bård H. Hoff[b], Eirik Sundby[a]*

a) Department of Materials Science & Engineering, Norwegian University of Science and Technology (NTNU), NO-7491 Trondheim, Norway.
b) Department of Chemistry, Norwegian University of Science and Technology (NTNU), NO-7491 Trondheim, Norway.
c) Department of Biomedical Laboratory Science, Norwegian University of Science and Technology (NTNU), NO-7491 Trondheim, Norway.
d) Skogmo Industriområde, Industrivegen 50, N-7863 Overhalla, Norway
e) University of Edinburgh Centre for Inflammation Research, Queen's Medical Research Institute, University of Edinburgh, Edinburgh EH16 4TJ, UK
f) Lead Discovery Center GmbH, Otto-Hahn-Straße 15, 44227 Dortmund, Germany.

*Corresponding author: E. Sundby, E-mail: eirik.sundby@ntnu.no.

Contents of SI

1. Synthesis of building blocks S2
2. Amination of protected pyrrolopyrimidines S3
3. Suzuki-cross coupling of aminated pyrrolopyrimidines S13
4. CSF1R binding assay non-autoinhibited form S42
5. CSF1R binding assay autoinhibited form S44
6. Kinase panels S46
7. X-ray co-crystal structure S47
8. HPLC trace key compounds S51
9. NMR spectra of key compounds S56
10. Compounds screen for CSF1R activity S150
11. Synthetic protocols for compounds in the CSF1R screen S157
12. References S178

1. Synthesis of building blocks

4-Chloro-7-((2-(trimethylsilyl)ethoxy)methyl)-7*H*-pyrrolo[2,3-*d*]pyrimidine[1]

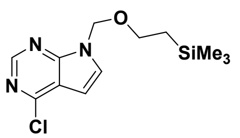

The reaction was performed using a Syrris Atlas HD 2L Jacketed Reactor equipped with a Huber Ministat 125 circulator pump. Dry dimethyl formamide (200 mL) was cooled to 0 ˚C and added sodium hydride (3.9 g, 162.5 mmol). 4-Chloro-7*H*-pyrrolo[2,3-*d*]pyrimidine (20.1 g, 131.0 mmol) was dissolved in dry dimethyl formamide (150 mL) and added stepwise in 20 mL increments to the chilled suspension over 15 min. The reaction mixture reached an internal temperature of 6.5 ˚C and was left stirring to re-cool to 0 ˚C. SEM-Cl (23 mL, 132.8 mmol) was added 35 min. after the first addition of starting material. The reaction mixture reached an internal temperature of 13 ˚C. The mixture was left stirring for 1 h, while cooling, before quenching with sat. aq. NH_4Cl (10 mL). The mixture was transferred to a round-bottom flask and concentrated in vacuo reducing the reaction volume by 345 mL. The concentrated reaction mixture was partitioned between CH_2Cl_2 (150 mL) and water (200 mL). The layers were separated and the water-phase extracted with CH_2Cl_2 (3 × 50 mL). The combined organic layers were washed with water (2 × 100 mL) and brine (225 mL), dried with anhydrous Na_2SO_4, filtered and concentrated in vacuo. The residue was purified by column chromatography on silica-gel (*n*-pentane/CH_2Cl_2/EtOAc – 10:10:1) yielding 32.9 g (115.8 mmol, 88%) of an off-white solid. ^{1}H NMR (400 MHz, DMSO-d_6) δ 8.68 (s, 1H), 7.87 (d, J = 3.6 Hz, 2H), 6.71 (d, J = 3.6 Hz, 1H), 5.65 (s, 1H), 3.52 (t, J = 8.0 Hz, 2H), 0.82 (t, J = 8.0 Hz, 2H), -0.10 (s, 9H).

4-Chloro-6-iodo-7-((2-(trimethylsilyl)-ethoxy)methyl)-7*H*-pyrrolo[2,3-*d*]pyrimidine (53)[2]

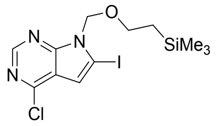

Under an N_2 atmosphere 4-chloro-7-((2-(trimethylsilyl)-ethoxy)methyl)-7*H*-pyrrolo[2,3-*d*]pyrimidine (5.00 g, 17.6 mmol) was dissolved in dry THF (70 mL) and cooled down to -78 °C. Then, LDA (2 M in THF/*n*-hexane/ethylbenzene, 13.3 mL, 26.6 mmol) was added dropwise over 30 min. This was followed by drop wise addition of I_2 (5.08 g, 20.1 mmol) dissolved in THF (12 mL). After another 30 min, the reaction mixture was quenched with saturated NH_4Cl solution (0.5 mL) and stirred until ambient was reached. The mixture was

concentrated and diluted with 10% $Na_2S_2O_3$ solution (20 mL), CH_2Cl_2 (25 mL) and water (30 mL). After phase separation, the water phase was extracted with more CH_2Cl_2 (4 × 20 mL). The combined organic phase was dried over Na_2SO_4 and the solvent was removed under reduced pressure. The crude product was purified by silica-gel flash chromatography (*n*-pentane/EtOAc - 9:1, R_f = 0.44) giving 6.60 g (16.1 mmol, 92%) of 4-chloro-6-iodo-7-((2-(trimethylsilyl)-ethoxy)methyl)-7*H*-pyrrolo[2,3-*d*]pyrimidine as a grey powder, mp. 99 - 101 °C; ^{1}H NMR (600 MHz, DMSO-d_6) δ 8.62 (s, 1H), 7.11 (s, 1H), 5.61 (s, 2H), 3.53, (t, J = 7.9 Hz, 2H), 0.82 (t, J = 7.9 Hz, 2H), -1.11 (s, 9H); ^{13}C NMR (150 MHz, DMSO-d_6) δ 152.5, 150.8, 149.0, 118.6, 109.8, 91.5, 73.5, 66.0, 17.1, -1.4 (3C).

2. Amination of protected pyrrolopyrimidines

General Procedure A-Amination of protected pyrrolopyrimidines

4-Chloro-6-iodo-7-((2-(trimethylsilyl)-ethoxy)methyl)-7*H*-pyrrolo[2,3-*d*]pyrimidine (1.00 g, 1 equiv.) was dissolved in dry *n*-BuOH or dioxane (10 mL), added the benzylamine (1.5-3 equiv.) and optionally *N,N*-diisopropylethylamine (3 equiv.). The reaction was stirred at 100-140 ˚C for 4-24 h. Following evaporation of solvent, the residue is added water (20 mL) and EtOAc (50 mL). After phase separation the water phase is extracted with more EtOAc (3 × 50 mL). The combined organic phase is then dried over $MgSO_4$ and concentrated at low pressure. The products were purified by silica gel column chromatography as specified below.

***N*-Benzyl-6-iodo-*N*-methyl-7-((2-(trimethylsilyl)ethoxy)methyl)-7*H*-pyrrolo[2,3-*d*]pyrimidin-4-amine (54)**

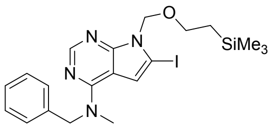

Compound **53** (3.04 g, 7.13 mmol) and *N*-methyl-1-phenylmethanamine (2.59 g, 21.4 mmol) were reacted as described in General Procedure A. The reaction time was 5 h. Purification by silica-gel chromatography (*n*-pentane/EtOAc - 3:1, R_f = 0.40) 3.17 g (6.42 mmol, 90%) of a solid was obtained, mp. 67 - 69 °C. ^{1}H NMR (400 MHz, DMSO-d_6) δ 8.14 (s, 1H), 7.34 - 7.28 (m, 2H), 7.27 - 7.20 (m, 3H), 6.94 (s, 1H), 5.50 (s, 2H), 4.99 (s, 2H), 3.55 - 3.49 (m, 2H), 3.29 (s, 3H), 0.85 - 0.79 (m, 2H), -0.09 (s, 9H); ^{13}C NMR (100 MHz, DMSO-d_6) δ 155.4, 152.8, 151.3, 138.1, 128.5 (2C), 127.0, 126.9 (2C), 112.3, 104.1, 80.4, 72.7, 65.5, 52.7, 37.4, 17.1, -1.3 (3C); HRMS (ASAP+, m/z): found 495.1080, calcd for $C_{20}H_{28}N_4OSiI$, $[M+H]^+$, 495.1077.

***N*-Benzyl-6-iodo-7-((2-(trimethylsilyl)ethoxy)methyl)-*7H*-pyrrolo[2,3-*d*]pyrimidin-4-amine (55)**

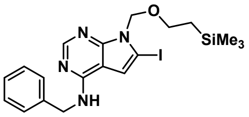

Compound **53** (198 mg, 0.48 mmol) and benzylamine (160 µL, 1.46 mmol) were reacted as described in General Procedure A. The reaction time was 5 h. Purification by silica-gel chromatography (*n*-pentane/EtOAc - 9:1, R_f = 0.19, then 4:1) gave 228 mg (0.47 mmol, 98%) of a faint yellow oil. ^{1}H NMR (400 MHz, DMSO-d_6) δ 8.11 (t, J = 6.0 Hz, 1H), 8.10 (s, 1H), 7.34 - 7.28 (m, 4H), 7.25 - 7.20 (m, 1H), 7.02 (s, 1H), 5.46 (s, 2H), 4.72 (d, J = 6.0 Hz, 2H), 3.53 - 3.49 (m, 2H), 0.83 - 0.79 (m, 2H), -0.09 (s, 9H); ^{13}C NMR (101 MHz, DMSO-d_6) δ 154.6, 152.0, 151.4, 139.9, 128.3 (2C), 127.2 (2C), 126.7, 109.9, 104.7, 79.8, 72.6, 65.4, 43.1, 17.1, -1.3 (3C); IR (neat, cm^{-1}): 3271 (w), 3030 (w), 2951 (w), 2893 (w), 1597 (s), 1562 (m), 1451 (m), 1337 (m), 1295 (m), 1246 (m), 1074 (s), 831 (s), 745 (s), 694 (s); HRMS (ASAP+, m/z): found 481.0920, calcd for $C_{19}H_{26}N_4OSiI$, $[M+H]^+$, 481.0921.

***N*-Benzyl-*N*-ethyl-6-iodo-7-((2-(trimethylsilyl)ethoxy)methyl)-*7H*-pyrrolo[2,3-*d*]pyrimidin-4-amine (56)**

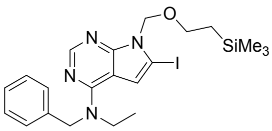

Compound **53** (212 mg, 0.52 mmol) and *N*-benzylethanamine (230 µL, 1.55 mmol) were reacted as described in General Procedure A. The reaction time was 13 h. Purification by silica-gel chromatography (*n*-pentane/EtOAc - 9:1, R_f = 0.32) gave 255 mg (0.50 mmol, 97%) as a faint yellow oil; ^{1}H NMR (400 MHz, DMSO-d_6) δ 8.14 (s, 1H), 7.34 - 7.30 (m, 2H), 7.26 - 7.22 (m, 3H), 6.76 (s, 1H), 5.49 (s, 2H), 4.97 (s, 2H), 3.70 (q, J = 7.0 Hz, 2H), 3.54 - 3.50 (m, 2H), 1.18 (t, J = 7.0 Hz, 3H), 0.83 - 0.79 (m, 2H), -0.09 (s, 9H); ^{13}C NMR (101 MHz, DMSO-d_6) δ 154.6, 152.8, 151.3, 138.4, 128.5 (2C), 126.9, 126.8 (2C), 111.9, 103.4, 80.8, 72.8, 65.5, 50.6, 43.2, 17.2, 13.0, -1.4 (3C); IR (neat, cm^{-1}): 3029 (w), 2950 (w), 2894 (w), 1562 (s), 1449 (m), 1430 (m), 1295 (m), 1247 (m), 1076 (m), 833 (m), 752 (m); HRMS (ASAP+, m/z): found 509.1233, calcd for $C_{21}H_{30}N_4OSiI$ $[M+H]^+$ 509.1234.

***N*-Benzyl-6-iodo-N-isopropyl-7-((2-(trimethylsilyl)ethoxy)methyl)-7*H*-pyrrolo[2,3-*d*]pyrimidin-4-amine (57)**

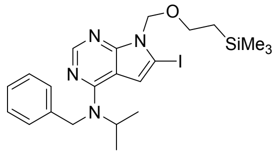

Compound **53** (292 mg, 0.712 mmol) and *N*-benzylpropan-2-amine (360 µL, 2.15 mmol) were reacted as described in General Procedure A. The reaction time was 48 h. Purification by silica-gel chromatography (*n*-pentane/EtOAc - 9:1, R_f = 0.38) gave 309 mg (0.591 mmol, 83%) as a faint yellow oil; ^{1}H NMR (600 MHz, $CDCl_3$) δ 8.29 (s, 1H), 7.33 – 7.21 (m, 5H), 6.50 (s, 1H), 5.58 (s, 2H), 5.21 – 5.18 (m, 1H), 4.89 (s, 2H), 3.62 – 3.56 (m, 2H), 1.23 (d, *J* = 6.7 Hz, 6H), 0.95 – 0.89 (m, 2H), -0.06 (s, 1H); ^{13}C NMR (151 MHz, $CDCl_3$) δ 156.2, 153.4, 151.7, 139.5, 128.6 (2C), 126.9, 126.3 (2C), 113.0, 104.8, 73.2, 66.3, 47.9, 46.8, 20.5, 17.8, -1.4 (3C); IR (neat, cm^{-1}): 3027 (w), 2952 (w), 2895 (w), 1556 (s), 1472 (m), 1449 (m), 1432 (m), 1294 (m), 1247 (m), 1225 (m), 1077 (s), 832 (s), 693 (m); HRMS (ASAP+, m/z): found 523.1386, calcd for $C_{22}H_{32}N_4OSiI$, $[M+H]^+$, 523.1390.

(*R*)-6-Iodo-*N*-methyl-*N*-(1-phenylethyl)-7-((2-(trimethylsilyl) ethoxy)methyl)-7*H*-pyrrolo[2,3-*d*]pyrimidin-4-amine (58)

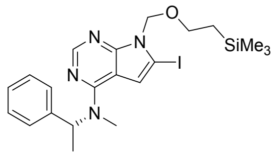

Compound **53** (297 mg, 0.73 mmol) and (*R*)-(+)-*N*,α-dimethylbenzylamine (0.32 mL, 2.2 mmol, 3 equiv.) were treated as described in General Procedure A. The reaction time was 29 h. The crude product was purified by silica-gel column chromatography (*n*-pentane/EtOAc - 3:1, R_f = 0.53). This gave 266 mg (0.52 mmol, 72%) of an orange oil; $[\alpha]_D^{20}$ = +67.7 (*c* 1.00, $CHCl_3$); ^{1}H NMR (400 MHz, DMSO-d_6) δ 8.15 (s, 1H), 7.36 - 7.32 (m, 2H), 7.32 - 7.24 (m, 3H), 7.01 (s, 1H), 6.37 (br s, 1H), 5.51 (s, 2H), 3.55 - 3.51 (m, 2H), 3.00 (s, 3H), 1.58 - 1.56 (m, 3H), 0.85 - 0.81 (m, 2H), -0.07 (s, 9H); ^{13}C NMR (151 MHz, $CDCl_3$) δ 156.0, 153.3, 151.8, 140.9, 128.6 (2C), 127.3, 127.1 (2C), 113.2, 105.2, 76.6, 73.2, 66.4, 53.0, 31.8, 17.8, 16.1, -1.4 (3C); IR (neat, cm^{-1}): 3033 (w), 2898 (w), 2893 (w), 2358 (w), 1561 (s), 1495 (m), 1451 (m), 1411 (m), 1295 (m), 1247 (m), 1083 (m), 834 (m), 748 (m), 697 (m); HRMS (ASAP+, m/z): found 509.1230, calcd for $C_{21}H_{30}N_4OSiI$, $[M+H]^+$, 509.1234.

(*R*)-*N*-(1-(4-(*tert*-Butyl)phenyl)ethyl)-6-iodo-*N*-methyl-7-((2-(trimethylsilyl)ethoxy)methyl)-*7H*-pyrrolo[2,3-*d*]pyrimidin-4-amine (59)

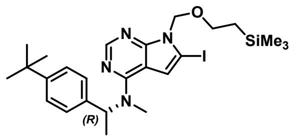

Compound **53** (234 mg, 0.30 mmol), (*R*)-1-(4-(*tert*-butyl)phenyl)-*N*-methylethan-1-amine (69 mg, 0.36 mmol) and *N*,*N*-diisopropylethylamine (210 µL, 1.21 mmol) were reacted as described in General Procedure A. The reaction time was 12.5 h. Purification by silica-gel chromatography (*n*-pentane/EtOAc - 19:1 then 9:1, R_f = 0.26) gave 147 mg (0.26 mmol, 87%) of a yellow oil. $[\alpha]_D^{20}$= +62.1 (*c* 1.00, $CHCl_3$); ^{1}H NMR (400 MHz, $CDCl_3$) δ 8.29 (s, 1H), 7.36 - 7.34 (m, 2H), 7.25 - 7.23 (m, 2H), 6.40 (br. s, 1H), 5.62 (s, 2H), 3.63 - 3.59 (m, 2H), 3.02 (s, 3H), 1.62 (d, *J* = 7.0 Hz, 3H), 1.31 (s, 9H), 0.96 - 0.92 (m, 2H), -0.03 (s, 9H); ^{13}C NMR (101 MHz, $CDCl_3$) δ 156.1, 153.4, 151.9, 150.1, 137.9, 126.8 (2C), 125.4 (2C), 113.2, 105.2, 76.3, 73.2, 66.3, 52.7, 34.5, 31.8, 31.3, 17.8, 16.2, -1.4 (3C); IR (neat, cm^{-1}): 2955 (w), 2901 (w), 2868 (w), 1558 (s), 1453 (m), 1410 (m), 1296 (m), 1268 (m), 1247 (m), 1075 (m), 856 (s), 748 (m); HRMS (ASAP+, m/z): found 565.1853, calcd for $C_{25}H_{38}N_4OSiI$, $[M+H]^+$, 565.1860.

***N*-Benzyl-6-iodo-*N*-(methyl-d$_3$)-7-((2-(trimethylsilyl)ethoxy)methyl)-7*H*-pyrrolo[2,3-*d*]pyrimidin-4-amine (60)**

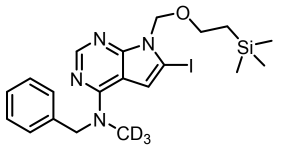

Compound **53** (534 mg, 1.302 mmol) and *N*-benzylmethan-*d*$_3$-amine (243 mg, 1.953 mmol) were reacted as described in General Procedure A. The reaction time was 3 h. Purification by silica-gel chromatography (*n*-pentane/EtOAc - 9:1, R_f = 0.10) gave 632 mg (1.271 mmol, 98%) of a clear oil; ^{1}H NMR (400 MHz, $CDCl_3$) δ 8.29 (s, 1H), 7.38 – 7.23 (m, 5H), 6.77 (s, 1H), 5.61 (s, 2H), 5.01 (s, 2H), 3.64 – 3.55 (m, 2H), 0.98 – 0.88 (m, 2H), -0.04 (s, 9H); ^{13}C NMR (101 MHz, $CDCl_3$) δ 156.1, 153.4, 151.9, 137.6, 128.8 (2C), 127.3, 127.1 (2C), 112.8, 104.9, 76.8, 73.2, 66.3, 53.6, 53.4, 17.8, -1.4 (3C); IR (neat, cm^{-1}): 3026 (w), 2949 (w), 2893 (w), 2069 (w), 1736 (w), 1561 (s), 1494 (m), 1474 (m), 1451 (m), 1431 (m), 1358 (m), 1297 (m), 1261 (m), 1247 (m), 1077 (s), 858 (m), 835 (m), 753 (m), 696 (m); HRMS (ASAP+, m/z): found 498.1267, calcd for $C_{20}H_{25}D_3N_4OSiI$, $[M+H]^+$, 498.1265.

6-Iodo-*N*-methyl-*N*-(2-methylbenzyl)-7-((2-(trimethylsilyl)ethoxy)methyl)-7*H*-pyrrolo[2,3-*d*]pyrimidin-4-amine (61)

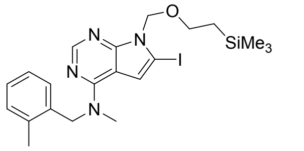

Compound **53** (311 mg, 0.760 mmol) and *N*-methyl-1-(*o*-tolyl)methanamine (320 µL, 2.13 mmol) were reacted as described in General Procedure A. The reaction time was 13.5 h. Purification by silica-gel chromatography (*n*-pentane/EtOAc - 9:1, R_f = 0.21) gave 371 mg (0.729 mmol, 96%) of a faint yellow oil; ^{1}H NMR (400 MHz, $CDCl_3$) δ 8.28 (s, 1H), 7.22 - 7.13 (m, 3H), 7.09-7.07 (m, 1H), 6.68 (s, 1H), 5.60 (s, 2H), 4.96 (s, 2H), 3.61 - 3.57 (m, 2H), 3.32 (s, 3H), 2.32 (s, 3H), 0.95 - 0.91 (m, 2H), -0,05 (s, 9H); ^{13}C NMR (100 MHz, $CDCl_3$) δ 156.2, 153.3, 151.9, 135.9, 135.0, 130.6, 127.2, 126.4, 126.3, 112.8, 105.0, 76.8, 73.2, 66.3, 52.0, 37.2, 19.1, 17.8, -1.4 (3C); IR (neat, cm^{-1}): 3027 (w), 2950 (w), 2894 (w), 1563 (s), 1412 (m), 1294 (m), 1245 (m), 1076 (s), 856 (m), 832 (s), 752 (s); HRMS (ASAP+, m/z): found 509.1234, calcd for $C_{21}H_{30}N_4OSiI$, $[M+H]^+$, 509.1234.

6-Iodo-*N*-methyl-*N*-(3-methylbenzyl)-7-((2-(trimethylsilyl)ethoxy)methyl)-7*H*-pyrrolo[2,3-*d*]pyrimidin-4-amine (62)

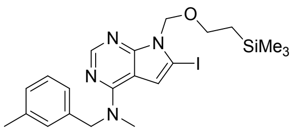

Compound **53** (4.5 g, 10.8 mmol) and *N*-methyl-1-(*m*-tolyl)methanamine (2.53 ml, 16.2 mmol) were treated as described in General Procedure A. The reaction time was 16 h. Purification by silica-gel column chromatography (*n*-pentane/EtOAc - 5:1, R_f = 0.24) gave 4.18 g (8.2 mmol, 76%) of an oil; ^{1}HNMR (400 MHz, $CDCl_3$) δ 8.29 (s, 1H), 7.24 - 7.20 (m, 1H), 7.10 - 7.04 (m, 3H), 6.77 (s, 1H), 5.61 (s, 2H), 4.97 (s, 2H), 3.63 - 3.56 (m, 2H), 3.30 (s, 3H), 2.33 (s, 3H), 0.97 - 0.90 (m, 2H), -0.04 (s, 9H); ^{13}C NMR (101 MHz, $CDCl_3$) δ 156.1, 153.4, 151.9, 138.5, 137.5, 128.7, 128.1, 127.8, 124.2, 112.9, 105.0, 73.2, 66.3, 53.7, 37.1, 21.5, 17.8, -1.4 (3C); IR (neat, cm^{-1}): 3027 (w), 2950 (w), 2894 (w), 1563 (s), 1412 (m), 1294 (m), 1245 (m), 1076 (s), 856 (m), 832 (s), 752 (s); HRMS (ASAP+, m/z): found 509.1244, calcd for $C_{21}H_{30}N_4OSiI$, $[M+H]^+$, 509.1234.

6-Iodo-*N*-methyl-*N*-(4-methylbenzyl)-7-((2-(trimethylsilyl)ethoxy)methyl)-7*H*-pyrrolo[2,3-*d*]pyrimidin-4-amine (63)

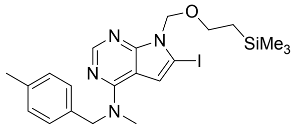

Compound **53** (294 mg, 0.718 mmol) and *N*-methyl-1-(p-tolyl)methanamine (325 µL, 2.16 mmol) were treated as described in General Procedure A. The reaction time was 12 h. Purification by silica-gel chromatography (*n*-pentane/EtOAc - 9:1, R_f = 0.29) gave 359 mg (0.706 mmol, 98%) of a faint yellow oil. ^{1}H NMR (400 MHz, $CDCl_3$) δ 8.28 (s, 1H), 7.15 - 7.14 (m, 4H), 6.76 (s, 1H), 5.60 (s, 2H), 4.96 (s, 2H), 3.61 - 3.57 (m, 2H), 3.30 (s, 3H), 2.34 (s, 3H), 0.95 - 0.91 (m, 2H), -0,04 (s, 9H); ^{13}C NMR (100 MHz, $CDCl_3$) δ 156.1, 153.3, 151.9, 137.0, 134.4, 129.4 (2C), 127.1 (2C), 112.8, 105.0, 73.2, 66.3, 53.5, 37.0, 21.1, 17.8, -1.4 (3C); IR (neat, cm^{-1}): 3114 (w), 2951 (w), 2856 (w), 1570 (s), 1552 (s), 1411 (m), 1246 (m), 1086 (s), 832 (s), 785 (s), 750 (s); HRMS (ASAP+, m/z): found 509.1232, calcd for $C_{21}H_{30}N_4OSiI$, $[M+H]^+$, 509.1234.

6-Iodo-*N*-methyl-N-(pyridin-2-ylmethyl)-7-((2-(trimethylsilyl)ethoxy)methyl)-7*H*-pyrrolo[2,3-*d*]pyrimidin-4-amine (64)

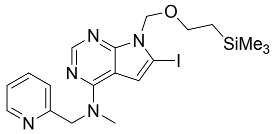

Compound **53** (321 mg, 0.783 mmol) and *N*-methyl-1-(pyridin-2-yl)methanamine (290 µL, 2.36 mmol) were treated as described in General Procedure A. The reaction time was 12 h. Purification by silica-gel chromatography (EtOAc, R_f = 0.31) gave 376 mg (0.758 mmol, 97%) of a faint yellow oil. ^{1}H NMR (600 MHz, $CDCl_3$) δ 8.62 – 8.57 (m, 1H), 8.29 (s, 1H), 7.68 – 7.62 (m, 1H), 7.28 – 7.24 (m, 1H), 7.23 – 7.20 (m, 1H), 6.79 (s, 1H), 5.60 (s, 2H), 5.14 (s, 2H), 3.62 – 3.55 (m, 2H), 3.45 (s, 3H), 0.96 – 0.89 (m, 2H), -0.05 (s, 9H); ^{13}C NMR (151 MHz, $CDCl_3$) δ 157.6, 155.6, 153.2, 151.4, 149.4, 137.2, 122.4, 121.4, 112.9, 105.0, 77.3, 73.3, 66.4, 55.9, 38.1, 17.8, -1.4 (3C); HRMS (ASAP+, m/z): found 496.1027, calcd for $C_{19}H_{27}N_5OSiI$, $[M+H]^+$, 496.1030.

6-Iodo-*N*-methyl-*N*-(pyridin-3-ylmethyl)-7-((2-(trimethylsilyl)ethoxy)methyl)-*7H*-pyrrolo[2,3-*d*]pyrimidin-4-amine (65)

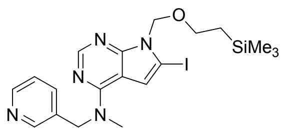

Compound **53** (290 mg, 0.71 mmol) and *N*-methyl-1-(pyridin-3-yl)methanamine (260 µL, 2.13 mmol) were treated as described in General Procedure A. The reaction time was 6.5 h. The crude product was purified by silica-gel column chromatography (EtOAc, R_f = 0.35) giving 348 mg (0.70 mmol, 99%) of a yellow oil. ^{1}H NMR (400 MHz, DMSO-d_6) δ 8.51 - 8.50 (m, 1H), 8.47 - 8.45 (m, 1H), 8.14 (s, 1H), 7.64 - 7.61 (m, 1H), 7.34 - 7.32 (m, 1H), 7.01 (s, 1H), 5.50 (s, 2H), 5.02 (s, 2H), 3.54 - 3.50 (m, 2H), 3.34 (s, 3H), 1.18 (t, *J* = 7.0 Hz, 3H), 0.84 -0.80 (m, 2H), -0.09 (s, 9H); ^{13}C NMR (100 MHz, DMSO-d_6) δ 155.2, 152.8, 151.2, 148.6, 148.3, 134.8, 133.7, 123.6, 112.2, 104.2, 80.7, 72.7, 65.5, 50.5, 37.5, 17.1, -1.4; IR (neat, cm^{-1}): 3030 (w), 2950 (w), 2894 (w), 1563 (s), 1413 (m), 1279 (m), 1246 (m), 1197 (w), 1074 (s), 1024 (m), 907 (m), 856 (m), 832 (s), 752 (s), 710 (s); HRMS (ASAP+, m/z): found 496.1027, calcd for $C_{19}H_{27}N_5OSiI$, $[M+H]^+$, 496.1030.

6-Iodo-*N*-methyl-*N*-(pyridin-4-ylmethyl)-7-((2-(trimethylsilyl)ethoxy)methyl)-7*H*-pyrrolo[2,3-*d*]pyrimidin-4-amine (66)

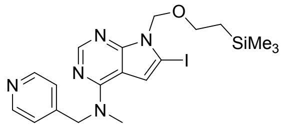

Compound **53** (280 mg, 0.684 mmol) and *N*-methyl-1-(pyridin-4-yl)methanamine (255 µL, 2.05 mmol) were treated as described in General Procedure A. The reaction time was 4 h. Purification by silica-gel chromatography (EtOAc, R_f = 0.16) gave 309 mg (0.623 mmol, 91%) as a faint yellow oil. ^{1}H NMR (400 MHz, $CDCl_3$) δ 8.57 -8.55 (m, 2H), 8.27 (s, 1H), 7.18 - 7.16 (m, 2H), 6.79 (s, 1H), 5.62 (s, 2H), 5.03 (s, 2H), 3.62 - 3.57 (m, 2H), 3.37 (s, 3H), 0.96 - 0.92 (m, 2H), -0,04 (s, 9H); ^{13}C NMR (151 MHz, $CDCl_3$) δ 155.8, 153.4, 151.7, 149.6 (2C), 147.8, 122.2 (2C), 112.5, 105.0, 77.6, 73.3, 66.4, 53.0, 37.7, 17.8, -1.4 (3C); IR (neat, cm^{-1}): 3114 (w), 2951 (w), 2856 (w), 1570 (s), 1552 (s), 1411 (m), 1246 (m), 1086 (s), 832 (s), 785 (s), 750 (s); HRMS (ASAP+, m/z): found 496.1025, calcd for $C_{19}H_{27}N_5OSiI$, $[M+H]^+$, 496.1030.

6-Iodo-*N*-methyl-*N*-((6-methylpyridin-2-yl)methyl)-7-((2-(trimethylsilyl)ethoxy)methyl)-7*H*-pyrrolo[2,3-*d*]pyrimidin-4-amine (67)

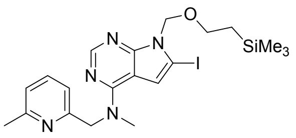

Compound **53** (205 mg, 0.502 mmol) and *N*-methyl-1-(6-methylpyridin-2-yl)methanamine (105 µL, 0.746 mmol) were treated as described in General Procedure A. The reaction time was 2.5 h. The crude product was purified by silica-gel column chromatography (*n*-pentane/EtOAc - 1:1, R_f = 0.26) giving 246 mg (0.482 mmol, 96%) as a yellow oil; ^{1}H NMR (600 MHz, $CDCl_3$) δ 8.28 (s, 1H), 7.54 – 7.48 (m, 1H), 7.08 – 7.04 (m, 1H), 7.01 – 6.97 (m, 1H), 6.76 (s, 1H), 5.60 (s, 2H), 5.09 (s, 2H), 3.61 – 3.55 (m, 2H), 3.42 (s, 3H), 2.59 (s, 3H), 0.95 – 0.90 (m, 2H), -0.05 (s, 9H); ^{13}C NMR (151 MHz, $CDCl_3$) δ 158.2, 157.1, 155.8, 153.3, 151.8, 137.4, 122.0, 117.9, 112.8, 105.0, 77.0, 73.2, 66.3, 56.0, 37.9, 24.3, 17.8, -1.4 (3C); HRMS (ASAP+, m/z): found 510.1180, calcd for $C_{20}H_{29}N_5OSiI$, $[M+H]^+$, 510.1186.

***N*-(2-Fluorobenzyl)-6-iodo-*N*-methyl-7-((2-(trimethylsilyl)ethoxy)methyl)-7*H*-pyrrolo[2,3-*d*]pyrimidin-4-amine (68)**

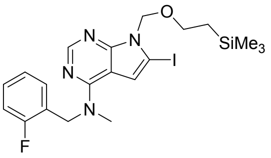

Compound **53** (264 mg, 0.644 mmol) and *N*-methyl-1-(6-methylpyridin-2-yl)methanamine (255 µL, 1.918 mmol) were treated as described in General Procedure A. The reaction time was 2 h. The crude product was purified by silica-gel column chromatography (*n*-pentane/EtOAc - 9:1) giving 299 mg (0.584 mmol, 91%) of a white solid, mp. 92.5 - 94.5 ˚C. ^{1}H NMR (600 MHz, $CDCl_3$) δ 8.29 (s, 1H), 7.29 – 7.20 (m, 2H), 7.12 – 7.04 (m, 2H), 6.77 (s, 1H), 5.61 (s, 2H), 5.07 (s, 2H), 3.62 – 3.56 (m, 2H), 3.36 (s, 3H), 0.96 – 0.89 (m, 2H), -0.05 (s, 9H); ^{13}C NMR (151 MHz, $CDCl_3$) δ 161.0 (d, J = 245.7 Hz, 1C), 156.0, 153.3, 151.7, 129.0, 128.9, 124.5 (d, J = 13.4 Hz, 1C), 124.4 (d, J = 3.5 Hz, 1C), 115.4 (d, J = 21.4 Hz, 1C), 112.8, 105.1, 77.1, 73.2, 66.4, 47.6, 37.5, 17.8, -1.4 (3C); IR (neat, cm^{-1}): 3117 (w), 2949 (w), 2940 (w), 2916 (w), 2854 (w), 1566 (s), 1548 (s), 1488 (m), 1450 (m), 1410 (m), 1377 (m), 1302 (m), 1287 (m), 1274 (m), 1247 (m), 1086 (s), 905 (m), 831 (s), 751 (s); HRMS (ASAP+, m/z): found 513.0980, calcd for $C_{20}H_{27}N_4OFSiI$, $[M+H]^+$, 513.0983.

2-(((6-Iodo-7-((2-(trimethylsilyl)ethoxy)methyl)-7*H*-pyrrolo[2,3-*d*]pyrimidin-4-yl)(methyl)amino)methyl)phenol (69)

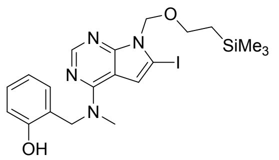

Compound **53** (201 mg, 0.490 mmol), *N*,*N*-diisopropylethylamine (335 µL, 1.957 mmol) and 2-((methylamino)methyl)phenol hydrochloride salt (170 mg, 0.979 mmol) were treated as described in General Procedure A. The reaction time was 3 h. Purification by silica-gel chromatography (*n*-pentane/EtOAc - 6:1, $R_f = 0.19$) gave 186 mg (0.364 mmol, 74%) of a faint yellow oil; ^{1}H NMR (600 MHz, $CDCl_3$) δ 11.77 (s, 1H), 8.29 (s, 1H), 7.27 – 7.24 (m, 2H), 6.94 (dd, J = 8.6, 1.2 Hz, 1H), 6.91 (s, 1H), 6.87 – 6.83 (m, 1H), 5.61 (s, 2H), 4.72 (s, 2H), 3.60 – 3.52 (m, 2H), 3.45 (s, 3H), 0.95 – 0.88 (m, 2H), -0.05 (s, 9H); ^{13}C NMR (151 MHz, $CDCl_3$) δ 156.8, 154.7, 152.6, 150.5, 131.4, 130.3, 122.3, 119.2, 117.9, 113.0, 105.2, 77.6, 73.4, 66.5, 51.9, 37.2, 17.8, -1.4 (3C); HRMS (ES+, m/z): found 511.1024, calcd for $C_{20}H_{28}N_4O_2SiI$, $[M+H]^+$, 511.1026.

2-(((6-Iodo-7-((2-(trimethylsilyl)ethoxy)methyl)-7*H*-pyrrolo[2,3-*d*]pyrimidin-4-yl)(methyl)amino)methyl)-4-methylphenol (70)

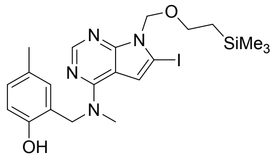

Compound **53** (260 mg, 0.634 mmol) and 4-methyl-2-((methylamino)methyl)phenol (285 mg, 0.695 mmol) were treated as described in General Procedure A. The reaction time was 1.5 h. Purification by silica-gel chromatography (*n*-pentane/EtOAc - 6:1, R_f = 0.20) gave 297 mg (0.567 mmol, 89%) as a brown oil; ^{1}H NMR (400 MHz, $CDCl_3$) δ 11.51 (s, 1H), 8.27 (s, 1H), 7.08 – 7.03 (m, 2H), 6.90 (s, 1H), 6.86 – 6.81 (m, 1H), 5.60 (s, 2H), 4.68 (s, 2H), 3.59 – 3.51 (m, 2H), 3.45 (s, 3H), 2.29 (s, 3H), 0.97 – 0.86 (m, 2H), -0.05 (s, 9H); ^{13}C NMR (101 MHz, $CDCl_3$) δ 154.8, 154.4, 152.7, 150.7, 131.9, 130.7, 128.2, 122.1, 117.6, 113.0, 105.2, 77.4, 73.3, 66.4, 51.8, 37.3, 20.4, 17.8, -1.4 (3C); HRMS (ES+, m/z): found 525.1179, calcd for $C_{21}H_{30}N_4O_2SiI$, $[M+H]^+$, 525.1183.

(S)-2-((6-Iodo-7-((2-(trimethylsilyl)ethoxy)methyl)-7H-pyrrolo[2,3-d]pyrimidin-4-yl)(methyl)amino)-2-phenylethan-1-ol (71)

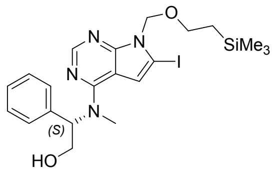

Compound **53** (215 mg, 0.524 mmol) and (S)-2-(methylamino)-2-phenylethan-1-ol (240 mg, 1.587 mmol) were treated as described in General Procedure A. The reaction time was 13 h; Purification by silica-gel chromatography (CH_2Cl_2/i-PrOH - 20:1, R_f = 0.24) gave 197 mg (0.375 mmol, 72%) of a yellow oil; ^{1}H NMR (600 MHz, $CDCl_3$) δ 8.25 (s, 1H), 7.38 – 7.33 (m, 2H), 7.32 – 7.28 (m, 1H), 7.26 – 7.23 (m, 2H), 6.88 (s, 1H), 6.29 (dd, J = 9.6, 4.3 Hz, 1H), 5.62 (s, 2H), 4.35 (dd, J = 11.2, 4.3 Hz, 1H), 4.25 (dd, J = 11.2, 9.6 Hz, 1H), 3.60 (t, J = 8.3 Hz, 2H), 3.11 (s, 3H), 0.98 – 0.90 (m, 3H), -0.03 (s, 9H); ^{13}C NMR (151 MHz, $CDCl_3$) δ 157.1, 153.2, 151.1, 137.1, 128.8 (2C), 127.9, 127.6 (2C), 113.1, 105.7, 77.5, 73.3, 66.4, 62.4, 60.7, 33.5, 17.8, -1.4 (3C); IR (neat, cm^{-1}): 3117 (w), 2949 (w), 2940 (w), 2916 (w), 2885 (w), 2854 (w), 1566 (s), 1548 (s), 1488 (m), 1450 (m), 1410 (m), 1377 (m), 1302 (m), 1286 (m), 1274 (m), 1247 (m), 1208 (m), 1086 (s), 905 (m), 853 (m), 831 (s), 751 (s); HRMS (ES+, m/z): found 525.1182, calcd for $C_{21}H_{30}N_4O_2SiI$, $[M+H]^+$, 525.1183.

(R)-2-((6-Iodo-7-((2-(trimethylsilyl)ethoxy)methyl)-7H-pyrrolo[2,3-d]pyrimidin-4-yl)(methyl)amino)-2-phenylethan-1-ol (72)

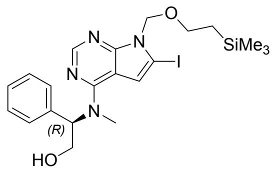

Compound **53** (203 mg, 0.495 mmol) and (R)-2-(methylamino)-2-phenylethan-1-ol (222 mg, 1.47 mmol) were treated as described in General Procedure A. The reaction time was 12.5 h. Purification by silica-gel chromatography (CH_2Cl_2/i-PrOH - 20:1, R_f = 0.24) gave 180 mg (0.342 mmol, 69%) of a yellow oil; ^{1}H NMR (400 MHz, $CDCl_3$) δ 8.28 (s, 1H), 7.43 – 7.26 (m, 5H), 6.91 (s, 1H), 6.33 (dd, J = 9.5, 4.4 Hz, 1H), 5.65 (s, 2H), 4.42 – 4.35 (m, 1H), 4.33 – 4.26 (m, 1H), 3.69 – 3.56 (m, 3H), 3.14 (s, 3H), 1.02 – 0.93 (m, 2H), 0.00 (s, 9H); ^{13}C NMR (101 MHz, $CDCl_3$) δ 157.4, 153.4, 151.4, 137.2, 128.8 (2C), 127.8, 127.7 (2C), 113.0, 105.7, 77.2, 73.2, 66.4, 62.6, 60.5, 33.4, 17.8, -1.4 (3C); HRMS (ES+, m/z): found 525.1180, calcd for $C_{21}H_{30}N_4O_2SiI$, $[M+H]^+$, 525.1183.

3. Suzuki-cross coupling of aminated pyrrolopyrimidines

General procedure B-*Suzuki-cross coupling of aminated pyrrolopyrimidines*

The 4-amino-6-iodo-7-((2-(trimethylsilyl)-ethoxy)methyl)-7*H*-pyrrolo[2,3-*d*]pyrimidine (1.0 equiv.), aryl boronic acid or pinacol ester (1.0 - 1.2 equiv.), $PdCl_2dppf$ (2 - 5 mol%) and potassium carbonate (3.0 equiv.) are charged in an appropriate reaction vessel. The atmosphere is evacuated and back-filled with N_2 three times before adding degassed 1,4-dioxane (6 mL/mmol starting material) and degassed water (3 mL/mmol starting material). The reaction vessel is lowered into an oil-bath set at 60 - 80 ^{0}C and stirred vigorously. Upon reaction completion, the reaction vessel is raised from the oil-bath and allowed to cool for 5 min. before the reaction mixture is transferred to a round-bottomed flask and the volatiles are removed by rotary evaporation. The residue is added water (20 mL/mmol starting material) and extracted with CH_2Cl_2 (3 x 20 mL/mmol starting material). The combined organic layers are washed with brine (20 mL/mmol), dried with anhydrous Na_2SO_4 and filtered. The organic solvent is removed under reduced pressure and the crude product is purified by silica-gel column chromatography. Some transformations were performed with alternative catalyst. This is specified.

(4-(4-(Benzyl(methyl)amino)-7-((2-(trimethylsilyl)ethoxy)methyl)-7*H*-pyrrolo[2,3-*d*]pyrimidin-6-yl)phenyl)methanol (73)

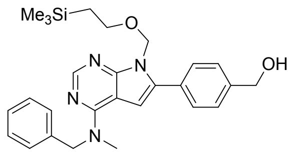

The material was made as described in General Procedure B, starting with **54** (261 mg, 0.529 mmol), 4-hydroxymethylphenylboronic acid and XPhos/XPhos 2. gen. pre-catalyst at 100 °C. The reaction time was 1.25 h. Purification by silica-gel chromatography (*n*-pentane/EtOAc - 1:1, R_f = 0.38) gave 183 mg (0.386 mmol, 73%) of an oil; ^{1}H NMR (600 MHz, DMSO-d_6) δ 8.24 (s, 1H), 7.67 - 7.66 (m, 2H), 7.41 - 7.39 (m, 2H), 7.34 - 7.31 (m, 2H), 7.27 - 7.23 (m, 3H), 6.78 (bs, 1H), 5.53 (s, 2H), 5.24 (t, J = 5.7 Hz, 1H), 5.05 (s, 2H), 4.54 (d, J = 5.7 Hz, 2H), 3.64 - 3.61 (m, 2H), 3.36 (s, 3H), 0.87 - 0.83 (m, 2H), -0.08 (s, 9H); ^{13}C NMR (150 MHz, DMSO-d_6) δ 156.4, 153.0, 151.2, 142.5, 138.3, 136.5, 129.7, 128.5 (2C), 128.3 (2C), 126.9 (3C), 126.6, (2C), 102.1, 102.0, 70.3, 65.7, 62.5, 52.8, 37.4, 17.3, -1.4 (3C); HRMS (ASAP+, m/z): found 474.2448, calcd for $C_{27}H_{34}N_4O_2Si$, $[M]^+$, 474.2451.

N-Benzyl-*N*-methyl-6-phenyl-7-((2-(trimethylsilyl)ethoxy)-methyl)-7*H*-pyrrolo[2,3-*d*]pyrimidin-4-amine (74)

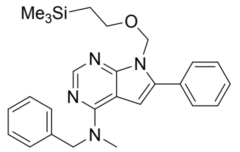

The material was made as described in General Procedure B, starting with **54** (257 mg, 0.52 mmol), phenylboronic acid and XPhos/XPhos 2. gen. pre-catalyst at 100 °C. The reaction time was 4 h. Purification by silica-gel column chromatography (*n*-pentane/EtOAc - 3:1, R_f = 0.38) gave 175 mg (0.39 mmol, 75%) of a yellow oil; ^{1}H NMR (400 MHz, DMSO-d_6) δ 8.25 (s, 1H), 7.72 - 7.70 (m, 2H), 7.49 - 7.45 (m, 2H), 7.42 - 7.32 (m, 1H), 7.34 - 7.32 (m, 1H), 7.29 - 7.22 (m, 3H), 6.80 (s, 1H), 5.55 (s, 2H), 5.06 (s, 2H), 3.58 - 3.64 (m, 2H), 3.37 (s, 3H), 0.87 - 0.81 (m, 2H), -0.09 (s, 9H); ^{13}C NMR (100 MHz, DMSO-d_6) δ 156.5, 153.1, 151.3, 138.2, 136.4, 131.4, 128.7 (2C), 128.5 (4C), 128.1, 126.9 (3C), 102.4, 102.1, 70.3, 65.7, 52.8, 37.4, 17.3, -1.4 (3C); IR (neat, cm^{-1}): 3033 (w), 2945 (w), 2888 (w), 2337 (w), 1568 (s), 1414 (m), 1307 (m), 1248 (m), 1073 (m), 858 (m), 834 (m), 778 (w), 752 (m), 697 (m); HRMS (ASAP+, m/z): found 445.2418, calcd for $C_{26}H_{33}N_4OSi$, $[M+H]^+$, 445.2424.

N-Benzyl-6-(4-methoxyphenyl)-*N*-methyl-7-((2-(trimethylsilyl)ethoxy)methyl)- 7*H*-pyrrolo[2,3-d]pyrimidin-4-amine (75)

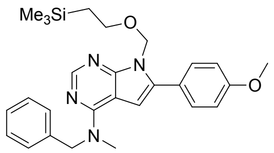

The reaction was performed as describe in General Procedure B, but using the pyrrolopyrimidine **54** (100 mg, 0.202 mmol), 4-methoxyphenyl boronic acid (33 mg, 0.24 mmol), and $Pd_2(dba)_3$ (9.6 mg, 10.1 µmol) at 100 °C. The reaction time was 15 min. The crude product was purified by silica-gel column chromatography (*n*-pentane/EtOAc - 3:1, R_f = 0.45). This gave 67 mg (0.141 mmol, 70%) of a transparent oil; ^{1}H NMR (600 MHz, DMSO-d_6) δ 8.23 (s, 1H), 7.62 (d, *J* = 8.9, 2H), 7.34 - 7.31 (m, 2H), 7.27 - 7.23 (m, 3H), 7.02 (d, *J* = 8.9 Hz, 2H). 6.70 (s, 1H), 5.51 (s, 2H), 5.05 (s, 2H), 3.80 (s, 3H), 3.61 (t, *J* = 8.4 Hz, 2H), 3.36 (s, 3H), 0.85 (t, *J* = 8.4 Hz, 2H), -0.08 (s, 9H); ^{13}C NMR (150 MHz, DMSO-d_6) δ 159.2, 156.3, 152.8, 151.0, 138.3, 136.4, 129.9 (2C), 128.5 (2C), 126.9 (3C), 123.7, 114.1 (2C), 102.1, 101.3, 70.2, 65.6, 55.2, 52.8, 37.3, 17.3, -1.5 (3C); IR (neat, cm^{-1}): 2950 (w), 1612 (w), 1566 (s), 1452 (m),

1307 (m), 1072 (m), 833(s). HRMS (ASAP+, m/z): found: 475.2521, calcd for $C_{27}H_{34}N_4O_2Si$, $[M+H]^+$, 475.2529.

4-(4-(Benzyl(methyl)amino)-7-((2-(trimethylsilyl)ethoxy)methyl)-7*H*-pyrrolo[2,3-*d*]pyrimidin-6-yl)phenol (76)

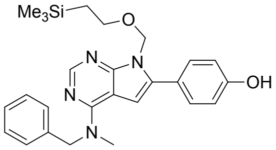

The reaction was performed as describe in General Procedure B, but using the pyrrolopyrimidine **54** (150 mg, 0.303 mmol), (4-hydroxyphenyl)boronic acid (50 mg, 0.36 mmol), and $Pd_2(dba)_3$ (5.6 mg, 6.1 μmol) at 100 °C. The reaction time was 30 min. The crude product was purified by silica-gel column chromatography (*n*-pentane/EtOAc - 3:1, R_f = 0.16). This gave 89 mg (0.194 mmol, 64%) of a transparent oil; ^{1}H NMR (600 MHz, DMSO-d_6) δ 9.69 (s, 1H), 8.21 (s, 1H), 7.50 (d, *J* = 8.8 Hz, 2H), 7.33 - 7.31 (m, 2H), 7.27 - 7.23 (m, 3H), 6.84 (d, *J* = 8.8 Hz, 2H), 6.64 (s, 1H), 5.49 (s, 2H), 5.04 (s, 2H), 3.61 (t, *J* = 8.1 Hz, 2H), 3.35 (s, 3H), 0.84 (t, *J* =8.1 Hz, 2H), -0.80 (s, 9H); ^{13}C NMR (150 MHz, DMSO-d_6) δ 157.6, 156.3, 152.7, 150.8, 138.3, 136.9, 130.0 (2C), 128.5 (2C), 126.9 (3C), 122.0, 115.4 (2C), 102.1, 100.8, 70.2, 65.6, 52.7, 37.3, 17.3,-1.4 (3C); IR (neat, cm^{-1}): 3062 (w), 2950 (w), 2895 (w), 1572 (s), 1489 (m), 1247 (m), 1076 (m), 835 (m); HRMS (ASAP+, m/z): found 461.2373, calcd for $C_{26}H_{33}N_4O_2Si$, $[M+H]^+$, 461.2367.

***N*-Benzyl-6-(4-methoxyphenyl)-*N*-methyl-7-((2-(trimethylsilyl)ethoxy)methyl)-7*H*-pyrrolo[2,3-*d*]pyrimidin-4-amine (77)**

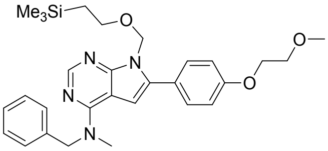

The reaction was performed as describe in General Procedure B, but using the pyrrolopyrimidine **54** (150 mg, 0.303 mmol), (4-(2-methoxyethoxy)phenyl)boronic acid (71 mg, 0.36 mmol), and $Pd_2(dba)_3$ (5.6 mg, 6.1 μmol) at 100 °C. The reaction time was 30 min. The crude product was purified by silica-gel column chromatography (*n*-pentane/EtOAc - 1:1, R_f = 0.65). This gave 130 mg (0.239 mmol, 79%) of a transparent oil; ^{1}H NMR (600 MHz, DMSO-d_6) δ 8.22 (s, 1H), 7.62 (d, *J* = 8.8 Hz, 2H), 7.34 - 7.31 (m, 2H), 7.27 - 7-23 (m, 3H), 7.04 (d, *J* = 8.8 Hz, 2H), 6.70 (s, 1H), 5.51 (s, 2H), 5.05 (s, 2H), 4.14 (t, *J* = 4.8 Hz, 2H), 3.67 (t, *J* = 4.8 Hz, 2H), 3.61 (t, *J* = 8.2 Hz, 2H), 3.36 (s, 3H), 3.31 (s, 3H), 0.85 (t, *J* = 8.2 Hz, 2H),

-0.08 (s, 9H); ^{13}C NMR (150 MHz, DMSO-d_6) δ 158.4, 156.3, 152.8, 151.0, 138.3, 136.3, 129.9 (2C), 128.5 (2C), 126.9 (3C), 123.8, 114.6 (2C), 102.1, 101.3, 70.3, 70.2, 67.0, 65.6, 58.1, 52.7, 37.3, 17.3, -1.5 (3C); IR (neat, cm^{-1}): 3029 (w), 2949 (w), 2892 (w), 1611 (s), 1561 (m), 1247 (s), 1074 (m), 857 (m); HRMS (ASAP+, m/z): found 519.2791, calcd for $C_{29}H_{39}N_4O_3Si$, $[M+H]^+$, 519.2781.

***N*-Benzyl-6-(4-(2-(2-(2-methoxyethoxy)ethoxy)ethoxy)phenyl)-*N*-methyl-7-((2-(trimethylsilyl)-ethoxy)methyl)-7*H*-pyrrolo[2,3-*d*]pyrimidin-4-amine (78)**

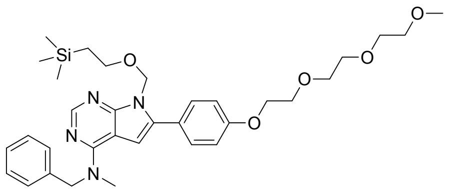

The reaction was performed as describe in General Procedure B, but using the pyrrolopyrimidine **54** (150 mg, 0.303 mmol), 2-(4-(2,5,8,11-tetraoxadodecyl)phenyl)-4,4,5,5-tetramethyl-1,3,2-dioxaborolane and Pd(PPh$_3$)$_4$ (17.6 mg) at 100 °C. The reaction time was 45 min. The crude product was purified by silica-gel column chromatography (*n*-pentane/EtOAc - 3:2, R_f = 0.33). This gave 78 mg (0.128 mmol, 42%) of a yellow oil; ^{1}H NMR (400 MHz, CDCl$_3$) δ 8.39 (s, 1H), 7.58 (d, J = 8.6 Hz, 2H), 7.35-7.26 (m, 5H), 6.97 (d, J = 8.6 Hz, 2H), 6.48 (s, 1H), 5.55 (s, 2H), 5.06 (s, 2H), 4.21-4.13 (m, 2H), 3.91-3.85 (m, 2H), 3.77 - 3.65 (m, 8H), 3.57 - 3.54 (m, 2H), 3.38 (s, 3H), 3.36 (s, 3H), 0.99-0.95 (m, 2H), -0.02 (s, 9H); ^{13}C NMR (100 MHz, CDCl$_3$) δ 158.9, 157.1, 153.5, 151.4, 138.0, 137.4, 130.5 (2C), 128.7 (2C), 127.2, 127.2 (2C), 124.4, 114.8 (2C), 102.9, 102.0, 72.0, 70.9, 70.7 (2C), 70.7, 70.6 (2C), 69.7, 67.5, 66.4, 59.0, 53.8, 37.2, 18.0, -1.4; IR (neat, cm^{-1}): 2948 (w), 2920 (w), 2874 (w), 1568 (s), 1498 (s), 1452 (m), 1415 (m), 1307 (s), 1247 (s), 1072 (s), 834 (s), 765 (m), 729 (s), 697 (m); HRMS (ASCI/ASAP, m/z): found 607.3311, calcd for $C_{33}H_{46}N_4O_5Si$, $[M+H]^+$, 607.3316.

6-(3-((1,3-Dioxolan-2-yl)methoxy)phenyl)-N-benzyl-N-methyl-7-((2-(trimethylsilyl)ethoxy)methyl)-7H-pyrrolo[2,3-d]pyrimidin-4-amine (79)

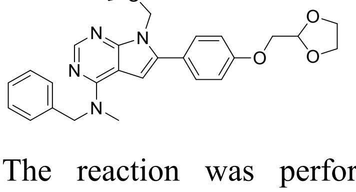

The reaction was performed as describe in General Procedure B, but using the pyrrolopyrimidine **54** (100 mg, 0.202 mmol), (4-((1,3-dioxolan-2-yl)methoxy)phenyl)boronic acid (74 mg, 0.24 mmol), and Pd$_2$(dba)$_3$ (9.3 mg, 10.1 µmol) at 70 °C. The reaction time was

3 h. The crude product was purified by silica-gel column chromatography (*n*-pentane/EtOAc - 2:1, R_f = 0.26). This gave 79 mg (0.145 mmol, 72%) of a transparent oil; ^{1}H NMR (600 MHz, $CDCl_3$) δ 8.40 (s, 1H), 7.34 - 7.31 (m, 3H), 7.29 - 7.27 (m, 4H), 7.20 - 7.15 (m, 1H), 6.97 - 6.95 (m, 1H), 6.55 (s, 1H), 5.58 (s, 1H), 5.30 (t, *J* = 4.1 Hz, 1H) 5.07 (s, 2H), 4.06 (d, *J* = 4.1 Hz, 2H), 4.06 - 4.04 (m, 2H), 3.98 - 3.95 (m, 2H), 3.73 (t, *J* = 8.4 Hz, 2H), 3.37 (s, 3H), 0.97 (t, *J* = 8.4 Hz, 2H), -0.03 (s, 9H); ^{13}C NMR (150 MHz, $CDCl_3$) δ 158.7, 157.3, 153.5, 151.7, 137.8, 137.2, 133.1, 129.7, 128.7 (2C), 128.3, 127.1 (2C), 121.9, 115.0, 114.7, 102.8, 102.1, 101.9, 70.7, 68.8, 66.4, 65.3 (2C), 53.3, 37.2, 18.1, -1.4 (3C); IR (neat, cm^{-1}): 3028 (w), 2950 (w), 2889 (w), 1567 (s), 1414 (w), 1308 (w), 1069 (m), 858 (m). HRMS (ASAP+, m/z): found 547.2733, calcd for $C_{30}H_{39}N_4O_2Si$, $[M+H]^+$, 547.2741.

3-(4-(Benzyl(methyl)amino)-7-((2-(trimethylsilyl)ethoxy)methyl)-7*H*-pyrrolo[2,3-*d*]pyrimidin-6-yl)phenol (80)

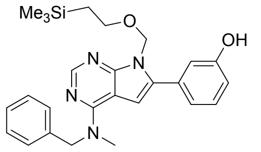

The reaction was performed as describe in General Procedure B, but using the pyrrolopyrimidine **54** (150 mg, 0.303 mmol), (3-hydroxyphenyl)boronic acid (50 mg, 0.36 mmol), and $Pd_2(dba)_3$ (5.6 mg, 6.1 μmol) at 100 °C. The reaction time was 1.5 h. The crude product was purified by silica-gel column chromatography (*n*-pentane/ EtOAc - 3:1, R_f = 0.28). This gave 93 mg (0.201 mmol, 66%) of a transparent oil; ^{1}H NMR (600 MHz, DMSO-d_6) δ 9.57 (s , 1H), 8.23 (s, 1H), 7.34 - 7.31 (m, 2H), 7.27 - 7.23 (m, 4H), 7.12 - 7.11 (m, 1H), 7.06 (s, 1H), 6.82 - 6.80 (m 1H), 6.72 (s, 1H), 5.53 (s, 2H), 5.05 (s, 2H), 3.59 (t, *J* = 8.0 Hz, 2H), 3.36 (s, 3H), 0.84 (t, *J* = 8.0 Hz, 2H), -0.09 (s, 9H); ^{13}C NMR (150 MHz, DMSO-d_6) δ 157.5, 156.5, 152.3, 151.2, 138.2, 136.6, 132.6, 129.6, 128.5 (2C), 126.9 (3C), 119.3, 115.5, 115.1, 102.0, 101.9, 70.3, 65.6, 52.8, 37.4, 17.3, -1.5 (3C); IR (neat, cm^{-1}): 3061 (w), 2951 (w), 1573 (s), 1415 (w) 1320 (w), 1246 (w), 1076 (m), 835 (w); HRMS (ASAP+, m/z): found 461.2373, calcd for $C_{26}H_{33}N_4O2Si$, $[M+H]^+$, 461.2373.

***N*-Benzyl-6-(3-methoxyphenyl)-*N*-methyl-7-((2-(trimethylsilyl)ethoxy)methyl)-7*H*-pyrrolo[2,3-*d*]pyrimidin-4-amine (81)**

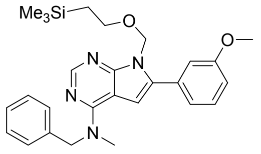

The reaction was performed as describe in General Procedure B, but using the pyrrolopyrimidine **54** (100 mg, 0.202 mmol), 3-methoxyphenylboronic acid (37 mg, 0.24 mmol), and $Pd_2(dba)_3$ (9.3 mg, 10.1 μmol) at 100 °C. The reaction time was 1.5 h. The crude product was purified by silica-gel column chromatography (*n*-pentane/EtOAc - 4:1, R_f = 0.51). This gave 68 mg (0.143 mmol, 71%) of a yellow oil; ^{1}H NMR (600 MHz, $CDCl_3$) δ 8.40 (s, 1H), 7.35 - 7.23 (m, 8H), 6.94 - 6.91 (m, 1H), 6.56 (s, 1H), 5.59 (s, 2H), 5.06 (s, 2H), 3.83 (s, 3H), 3.74 (t, *J* = 8.8 Hz, 2H), 3.37 (s, 3H), 0.97 (t, *J* = 8.8 Hz, 2H), -0.03 (s, 9H); ^{13}C NMR (150 MHz, $CDCl_3$) δ 159.7, 157.3, 153.5, 151.7, 137.8, 137.3, 133.0, 129.6, 128.7 (2C), 127.2, 127.1 (2C), 121.4, 114.4, 113.9, 102.8, 102.0, 70.7, 66.4, 55.3, 53.8, 37.2, 18.1, -1.5 (3C); IR (neat, cm^{-1}) : 3028 (w), 2950 (w), 2835 (w), 1567 (s), 1452 (m), 1308 (m), 1074 (m), 858 (m). HRMS (ASAP+, m/z): found 475.2529, calcd for $C_{27}H_{35}N_4O_2Si$, $[M+H]^+$, 475.2530.

4-(4-(Benzyl(methyl)amino)-7-((2-(trimethylsilyl)ethoxy)methyl)-7*H*pyrrolo[2,3-*d*]pyrimidin-6-yl)-2-fluorophenyl)methanol (82)

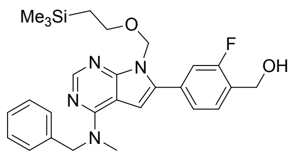

Step 1: Suzuki cross coupling

The compound was made as described in General Procedure B, starting with **54** (995 mg, 2.01 mmol) and 3-fluoro-4-formylphenylboronic acid. Using 3 mol % of catalyst the reaction time was 1 h. The product was purified by silica-gel column chromatography (*n*-pentane/EtOAc - 2:1, R_f = 0.34) giving 743 mg (1.51 mmol, 75%) of a bright yellow solid, mp. 89 - 92 ˚C. ^{1}H NMR (600 MHz, DMSO-d_6) δ 10.23 (s, 1H), 8.28 (s, 1H), 7.92 - 7.89 (m, 1H), 7.86 - 7.83 (m, 1H), 7.82 - 7.78 (m, 1H), 7.34 - 7.31 (m, 2H), 7.28 - 7.24 (m, 3H), 7.20 (br s, 1H), 5.64 (s, 2H), 5.08 (s, 2H), 3.69 - 3.65 (m, 2H), 3.39 (s, 3H), 0.90 - 0.86 (m, 2H), -0.08 (s, 9H); ^{13}C NMR (150 MHz, DMSO-d_6) δ 187.2 (d, *J* = 4.4 Hz), 163.4 (d, *J* = 256.6 Hz), 156.8, 153.9, 152.2, 139.4 (d, *J* = 9.8 Hz), 138.0, 133.7, 129.7 (d, *J* = 2.0 Hz), 128.5 (2C), 127.0 (3C), 124.3 (d, *J* = 1.8 Hz), 122.4 (d, *J* = 8.5 Hz), 115.4 (d, *J* = 22.4 Hz), 105.7, 102.1, 70.5, 65.9, 52.8, 37.6, 17.3, -1.4 (3C); ^{19}F NMR (376 MHz, DMSO-d_6, C_6F_6) δ -124.0 (s); IR (neat, cm^{-1}): 3070 (w), 3029 (w), 2951 (w), 2914 (w), 2862 (w), 1679 (m), 1615 (m), 1568 (s), 1545 (m), 1408 (w), 1311 (m), 1250 (m), 1202 (m), 1084 (s), 930 (w), 858 (m), 833 (s), 776 (m), 727 (s), 691 (m); HRMS (ASAP+, m/z): found 491.2271, calcd for $C_{27}H_{32}N_4O_2FSi$, $[M+H]^+$, 491.2279.

Step 2: reduction of aldehyde

The aldehyde from above (200 mg, 0.41 mmol) was dissolved in dry THF (20 mL) and MeOH (10 mL) under and an N_2-atmosphere and cooled to 0 °C. $NaBH_4$ (47.0 mg, 1.22 mmol) was added over 10 minutes under agitation. The mixture was further stirred for 7 hours at rt. Then, water (30 mL) and EtOAc (40 mL) was added. The phases were separated, and the water phase was extracted with more EtOAc (2 × 40 mL). The combined organic phases were washed with brine (60 mL), dried over Na_2SO_4, filtrated and concentrated in vacuo. The crude product was purified by silica-gel column chromatography (*n*-pentane/EtOAc - 1:1, R_f = 0.30). Drying gave 147 mg (0.299 mmol, 73%) of a white solid, mp. 107 - 110 °C. HPLC purity > 99%, t_R = 30.3 min; ^{1}H NMR (600 MHz, DMSO-d_6) δ 8.25 (s, 1H), 7.58 - 7.52 (m, 3H), 7.35 - 7.30 (m, 2H), 7.28 - 7.23 (m, 3H), 6.91 (br.s, 1H), 5.57 (s, 2H), 5.32 (t, *J* = 5.7 Hz, 1H), 5.06 (s, 2H), 4.59 (d, *J* = 5.7 Hz, 2H), 3.68 - 3.32 (m, 2H), 3.37 (s, 3H), 0.90 - 0.84 (m, 2H), -0.07 (s, 9H); ^{13}C NMR (150 MHz, DMSO-d_6) δ 159.5 (d, *J* = 243.6 Hz), 156.5, 153.2, 151.5, 138.2, 135.0, 131.9 (d, *J* = 8.8 Hz), 129.3 (d, *J* = 5.6 Hz), 128.8 (d, *J* = 15.3 Hz), 128.5 (2C), 127.0 (3C), 124.2 (d, *J* = 2.0 Hz), 114.5 (d, *J* = 23.2 Hz), 103.0, 102.0, 70.3, 65.8, 56.5 (d, *J* = 3.8 Hz), 52.8, 37.5, 17.3, -1.4 (3C); ^{19}F NMR (376 MHz, DMSO-d_6, C_6F_6) δ -121.8 (s); IR (neat, cm^{-1}): 3292 (br w), 3083 (w), 3023 (w), 2944 (w), 2889 (w), 1574 (s), 1548 (m), 1416 (m), 1320 (m), 1248 (m), 1072 (s), 1027 (m), 895 (m), 857 (s), 832 (s), 767 (s), 699 (s). HRMS (ASAP+, m/z): found 492.2352, calcd for $C_{27}H_{33}N_4O_2FSi$ [M]*+ 492.2357.

(5-(4-(Benzyl(methyl)amino)-7-((2-(trimethylsilyl)ethoxy)methyl)-7*H*-pyrrolo[2,3-*d*]pyrimidin-6-yl)-2-fluorophenyl) methanol (83)

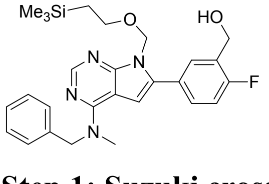

Step 1: Suzuki cross coupling

The compound was made as described in General Procedure B, but starting with **54** (1.00 g 2.03 mmol) and 4-fluoro-3-formylphenylboronic acid. Using 3 mol% of catalyst the reaction time was 90 min. The product was purified by silica-gel column chromatography (*n*-pentane/EtOAc - 2:1, R_f = 0.33) giving 773 mg (1.58 mmol, 78%) of a yellow oil. ^{1}H NMR (600 MHz, DMSO-d_6) δ 10.27 (s, 1H), 8.26 (s, 1H), 8.20 - 8.15 (m, 1H), 8.11 - 8.05 (m, 1H), 7.58 - 7.52 (m, 1H), 7.34 - 7.30 (m, 2H), 7.28 - 7.23 (m, 3H), 6.94 (br s, 1H), 5.54 (s, 2H), 5.06 (s, 2H), 3.62 - 3.59 (m, 2H), 3.37 (s, 3H), 0.89 - 0.86 (m, 2H), -0.09 (s, 9H); ^{13}C NMR (150 MHz, DMSO-d_6) δ 187.6 (d, *J* = 4.4 Hz), 162.9 (d, *J* = 259.5 Hz), 156.6, 153.1, 151.6, 138.2,

136.5 (d, J = 9.1 Hz), 134.0, 129.2, 128.6, 128.5 (2C), 127.0 (3C), 124.0 (d, J = 8.7 Hz), 117.3 (d, J = 20.9 Hz), 103.3, 102.0, 70.2, 65.5, 52.7, 37.4, 17.3, -1.5 (3C); ^{19}F NMR (400 MHz, DMSO-d_6, C_6F_6) δ -124.0 (s); IR (neat, cm^{-1}): 3060 (w), 2945 (w), 2868 (w), 1693 (w), 1567 (s), 1484 (m), 1423 (w), 1312 (m), 1247 (m), 1072 (s), 1026 (m), 857 (m), 832 (s), 762 (m), 695 (s), 576 (m); HRMS (ASAP+, m/z): found 491.2271, calcd for $C_{27}H_{32}N_4O_2FSi$, $[M+H]^+$, 491.2279.

Step 2: Reduction of aldehyde

The compound was made by reduction of the aldehyde formed above as described for compound **78**. Purification by silica-gel column chromatography (*n*-pentane/EtOAc - 3:2, R_f = 0.19) gave 189 mg (0.384 mmol, 92%) of a clear oil. HPLC purity > 99 %, t_R = 30.1 min; ^{1}H NMR (600 MHz, DMSO-d_6) δ 8.27 (s, 1H), 7.80 - 7.85 (m, 1H), 7.66 - 7.60 (m, 1H), 7.35 - 7.30 (m, 2H), 7.29 - 7.23 (m, 4H), 6.79 (br s, 1H), 5.53 (s, 2H), 5.06 (s, 2H), 4.59 (s, 2H), 3.61 - 3.56 (m, 2H), 3.37 (s, 3H), 0.87 - 0.82 (m, 2H), -0.09 (s, 9H); ^{13}C NMR (150 MHz, DMSO-d_6) δ 159.6 (d, J = 246.4 Hz), 156.1, 152.6, 150.7, 138.0, 135.8, 129.6 (d, J = 15.6 Hz), 129.5 (d, J = 4.9 Hz), 128.9 (d, J = 8.2 Hz), 128.6 (2C), 127.5 (d, J = 2.1 Hz), 127.0, 126.9 (2C), 115.3 (d, J = 21.6 Hz), 102.4, 102.0, 70.3, 65.6, 56.7 (d, J = 3.5 Hz), 52.9, 37.5, 17.3, -1.5 (3C); ^{19}F NMR (400 MHz, DMSO-d_6, C_6F_6) δ -122.6; IR (neat, cm^{-1}): 3310 (br w), 3060 (w), 3023 (w), 2945 (w), 2884 (w), 1672 (w), 1569 (s), 1487 (m), 1408 (m), 1246 (m), 1073 (s), 1026 (m), 831 (s), 764 (s), 695 (s), 622 (m); HRMS (ASAP+, m/z): found 492.2354, calcd for $C_{27}H_{33}N_4O_2FSi$, $[M]^+$, 492.2357.

***N*^{1}-(4-(4-(Benzyl(methyl)amino)-7-((2(trimethylsilyl)ethoxy)methyl)-7*H*pyrrolo[2,3-*d*]pyrimidin-6-yl)-2-fluorobenzyl)-*N*^{2}, *N*^{2}-dimethylethane-1,2-diamine (84)**

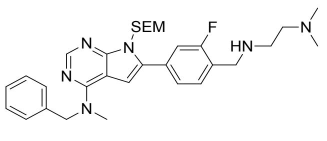

4-(4-(Benzyl(methyl)amino)-7-((2-(trimethylsilyl)ethoxy)methyl)-7*H*-pyrrolo[2,3-*d*]pyrimidin-6-yl)-2-fluorobenzaldehyde (see synthesis of **82**) (200 mg, 0.41 mmol) was dissolved in dry CH_2Cl_2 (4 mL) and *N*^{1}, *N*^{1}-dimethylethane-1,2-diamine (0.14 mL, 1.22 mmol) under an N_2-atmosphere and the reaction mixture was stirred at rt for 3.5 h, before it was concentrated in vacuo. The residue was dissolved in MeOH and $NaBH_4$ (49.5 mg, 1.22 mmol) was added over 10 min. The reaction was stirred at rt for 4.5 h., before the MeOH was evaporated in vacuo. The residue was diluted with water (30 mL) and EtOAc (40 mL) and the phases were separated, before the water phase was extracted with more EtOAc (2 × 30 mL).

The combined organic phases were washed with brine (30 mL), dried over Na_2SO_4, filtrated and concentrated. The crude product was purified by silica-gel column chromatography (CH_2Cl_2/MeOH/25% $NH_{3\ (aq)}$ - 8:1 : 0.1, R_f = 0.21). Drying gave 200 mg (0.360 mmol, 87%) of a slight yellow oil. HPLC purity 97%, t_R = 34.0 min; ^{1}H NMR (600 MHz, DMSO-d_6) δ 8.25 (s,1H), 7.58 - 7.54 (m, 1H), 7.54 - 7.49 (m, 2H), 7.35 - 7.29 (m, 2H), 7.29 - 7.22 (m, 3H), 6.90 (br s, 1H), 5.57 (s, 2H), 5.06 (s, 2H), 3.77 (s, 2H), 3.66 - 3.61 (m, 2H), 3.37 (s, 3H), 2.58 (t, J = 6.4 Hz, 2H), 2.32 (t, J = 6.4 Hz, 2H), 2.11 (s, 6H), 0.88 - 0.83 (m, 2H), -0.08 (s, 9H); ^{13}C NMR (150 MHz, DMSO-d_6) δ 160.4 (d, J = 243.4 Hz), 156.5, 153.2, 151.5, 138.2, 135.0, 131.8 (d, J = 8.8 Hz), 130.5 (d, J = 5.5 Hz), 128.5 (2C), 127.4 (d, J = 15.3 Hz), 127.0 (3C), 124.1 (d, J = 2.0 Hz), 114.6 (d, J = 24.0 Hz), 103.0, 102.0, 70.3, 65.8, 58.7, 52.8, 46.2, 45.8, 45.2 (2C), 37.4, 17.3, -1.4 (3C); ^{19}F NMR (400 MHz, DMSO-d_6, C_6F_6) δ -121.4 (s); IR (neat, cm^{-1}): 3023 (w), 2940 (w), 2883 (w), 2810 (w), 2758 (w), 1620 (w), 1567 (s), 1449 (m), 1310 (m), 1246 (m), 1074 (s), 858 (s), 833 (s), 763 (s), 696 (s), 629 (m). HRMS (ASAP+, m/z): found 563.3320, calcd for $C_{31}H_{44}N_6OFSi$, $[M+H]^+$, 563.3330.

Synthesis of *N*^{1}-(5-(4-(benzyl(methyl)amino)-7-((2(trimethylsilyl)ethoxy)methyl)-7*H*pyrrolo[2,3-*d*]pyrimidin-6-yl)-2-fluorobenzyl)-*N*^{2}, *N*^{2}-dimethylethane-1,2-diamine (85)

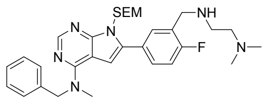

5-(4-(Benzyl(methyl)amino)-7-((2-(trimethylsilyl)ethoxy)methyl)-7*H*-pyrrolo[2,3-d]pyrimidin-6-yl)-2-fluoro-benzaldehyde (see synthesis of **83**) was reacted as described for compound **84**. Purification by silica-gel column chromatography (CH_2Cl_2/MeOH/25% $NH_{3\ (aq)}$ - 95:5:1, R_f = 0.17) gave 183 mg (0.325 mmol, 80%) of a slight yellow oil. HPLC purity 96%, t_R = 33.2 min; ^{1}H NMR (600 MHz, DMSO-d_6) δ 8.24 (s, 1H), 7.73 - 7.77 (1H), 7.62 - 7.58 (m, 1H), 7.34 - 7.30 (m, 2H), 7.28 - 7.23 (m, 4H), 6.77 (br s, 1H), 5.53 (s, 2H), 5.05 (s, 2H), 3.77 (s, 2H), 3.60 - 3.56 (m, 2H), 3.36 (s, 3H), 2.58 (t, J = 6.4 Hz, 2H), 2.31 (t, J = 6.4 Hz, 2H), 2.09 (s, 6H), 0.86 - 0.82 (m, 2H), -0.09 (s, 9H); ^{13}C NMR (150 MHz, DMSO-d_6) δ 160.3 (d, J = 245.8 Hz), 156.5, 153.0, 151.3, 138.2, 135.6, 130.6 (d, J = 5.0 Hz), 128.8 (d, J = 8.5 Hz), 128.5 (2C), 128.1 (d, J = 15.5 Hz), 127.9 (d, J = 2.9 Hz), 126.9 (3C), 115.3 (d, J = 22.6 Hz), 102.3, 102.0, 70.2, 65.6, 58.7, 52.8, 46.2, 46.1, 45.2 (2C), 37.4, 17.3, -1.5 (3C); ^{19}F NMR (400 MHz, DMSO-d_6, C_6F_6) δ -122.3 (s); IR (neat, cm^{-1}): 3024 (w), 2947 (w), 2884 (w), 2816 (w), 2762

(w), 1568 (s), 1488 (m), 1414 (m), 1310(m), 1247 (m), 1074 (s), 934 (w), 858 (m), 833 (s), 765 (s), 696 (s), 552 (w); HRMS (ASAP+, m/z): found 563.3321, calcd for $C_{31}H_{44}N_6OFSi$, $[M+H]^+$, 563.3330.

(4-(4-(Benzylamino)-7-((2-(trimethylsilyl)ethoxy)methyl)-7*H*-pyrrolo[*2,3-d*]pyrimidin-6-yl)phenyl)methanol (86)

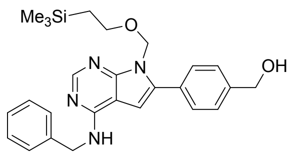

The compound was prepared as described in General Procedure B starting with **55** (79 mg, 0.16 mmol) and 4-hydroxymethylphenylboronic acid. The crude product was purified by silica-gel column chromatography (*n*-pentane/EtOAc - 3:2) giving 62 mg (0.13 mmol, 82%) of a faint yellow oil. TLC (silica, *n*-pentane/EtOAc - 1:1): R_f = 0.27); ^{1}H NMR (400 MHz, $CDCl_3$) δ 8.43 (s, 1H), 7.72 - 7.70 (m, 2H), 7.45 - 7.43 (m, 2H), 7.46 - 7.44 (m, 2H), 7.42 - 7.28 (m, 5H), 6.42 (s, 1H), 5.56 (s, 2H), 5.25 (t, *J* = 5.6 Hz, 1H), 4.87 (d, *J* = 5.6 Hz, 2H), 4.76 (s, 2H), 3.76 - 3.72 (m, 2H), 0.98 - 0.94 (m, 2H), -0.03 (s, 9H); ^{13}C NMR (101 MHz, $CDCl_3$) δ 155.9, 152.3, 152.3, 141.0, 138.8, 138.4, 131.1, 129.3 (2C), 128.8 (2C), 127.8 (2C), 127.6, 127.3 (2C), 103.0, 97.9, 70.7, 66.5, 65.0, 45.3, 18.0, -1.4 (3C); (ASAP+, m/z): found 461.2370, calcd for $C_{26}H_{33}N_4O_2Si$, $[M+H]^+$, 461.2373.

(4-(4-(Benzyl(ethyl)amino)-7-((2-(trimethylsilyl)ethoxy)methyl)-7H-pyrrolo[2,3-d]pyrimidin-6-yl)phenyl)methanol (87)

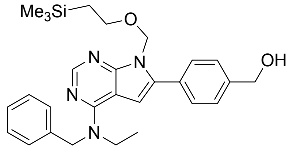

The compound was made as described in General Procedure B, but starting with **56** (81 mg, 0.16 mmol) and 4-hydroxymethylphenylboronic acid. The reaction time was 20 min. The crude product was purified by silica-gel column chromatography (*n*-pentane/EtOAc - 4:1) giving 64 mg (0.13 mmol, 82%) of a faint yellow oil. TLC (silica, *n*-pentane/EtOAc - 1:1): R_f= 0.51; ^{1}H NMR (400 MHz, $CDCl_3$) δ 8.39 (s, 1H), 7.67 - 7.65 (m, 2H), 7.44 - 7.42 (m, 2H), 7.34 - 7.24 (m, 5H), 6.44 (s, 1H), 5.56 (s, 2H), 5.30 (s, 1H), 5.05 (s, 2H), 4.75 (d, *J* = 5.6 Hz, 2H), 3.79 (q, *J* = 7.1 Hz, 2H), 3.77 - 3.72 (m, 2H), 1.73 (t, *J* = 5.8 Hz, 1H), 1.31 (t, *J* = 7.1 Hz, 3H), 0.99 -

0.95 (m, 2H), -0.02 (s, 9H); ^{13}C NMR (100 MHz, $CDCl_3$) δ 156.7, 153.7, 152.0, 140.9, 138.4, 137.3, 131.4, 129.5, 128.8, 127.4, 127.31, 127.26, 102.4, 102.1, 70.8, 66.6, 65.2, 51.5, 43.5, 18.2, 13.3, -1.2 (3C); HRMS (ASAP+, m/z): found 489.2679, calcd for $C_{28}H_{37}N_4O_2Si$, $[M+H]^+$, 489.2686.

(4-(4-(Benzyl(isopropyl)amino)-7-((2-(trimethylsilyl)ethoxy)methyl)-7*H*-pyrrolo[2,3-*d*]pyrimidin-6-yl)phenyl)methanol (88)

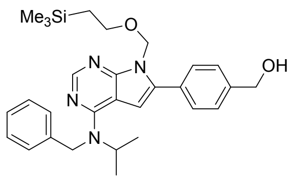

The compound was made as described in General Procedure B, but starting with **57** (131 mg, 0.251 mmol) and 4-hydroxymethylphenylboronic acid. The reaction time was 20 min. The crude product was purified by silica-gel column chromatography (*n*-pentane/EtOAc - 4:1, R_f = 0.07) giving 72 mg (0.14 mmol, 57%) of a faint yellow oil. ^1H NMR (400 MHz, $CDCl_3$) δ 8.39 (s, 1H), 7.57 - 7.56 (m, 2H), 7.40 - 7.39 (m, 2H), 7.30 - 7.29 (m, 4H), 7.25 - 7.21 (m, 1H), 6.28 (s, 1H), 5.53 (s, 2H), 5.34-5.29 (m, 1H), 4.96 (s, 2H), 4.73 (d, *J* = 5.7 Hz, 2H), 3.75 - 3.72 (m, 2H), 1.73 (t, *J* = 5.8 Hz, 1H), 1.26 - 1.25 (m, 6H), 0.97 - 0.94 (m, 2H), -0.03 (s, 9H); ^{13}C NMR (100 MHz, $CDCl_3$) δ 157.4, 153.7, 151.6, 140.7, 140.0, 136.9, 131.2, 129.2, 128.5, 127.2, 126.7, 126.4, 102.7, 102.3, 70.7, 66.4, 65.0, 47.6, 46.8, 20.5, 18.0, -1.4 (3C); HRMS (ASAP+, m/z): found 503.2834, calcd for $C_{29}H_{39}N_4O_2Si$ $[M+H]^+$ 503.2842.

(*R*)-(4-(4-(methyl(1-phenylethyl)amino)-7-((2-(trimethylsilyl)ethoxy)methyl)-7*H*-pyrrolo[2,3-*d*]pyrimidin-6-yl)phenyl)methanol (89)

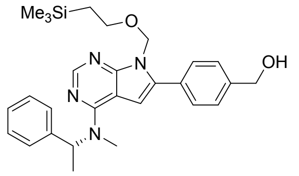

The compound was made as described in General Procedure B, but starting with **58** (124 mg, 0.244 mmol) and 4-hydroxymethylphenylboronic acid. The reaction time was 7 min. The crude product was purified by silica-gel column chromatography (*n*-pentane/EtOAc - 3:2, R_f = 0.27) giving 105 mg (0.215 mmol, 88%) of a yellow oil. ^1H NMR (400 MHz, $CDCl_3$) δ 8.41 (s, 1H), 7.73 – 7.69 (m, 2H), 7.47 – 7.42 (m, 2H), 7.38 – 7.32 (m, 4H), 7.30 – 7.24 (m, 1H), 6.63 (s, 1H), 6.51 (q, *J* = 8.4 Hz, 1H), 5.62 – 5.55 (m, 2H), 4.76 (d, *J* = 5.9 Hz, 2H), 3.79 – 3.73 (m,

2H), 3.08 (s, 3H), 1.74 (t, J = 5.9 Hz, 1H), 1.66 (d, J = 7.0 Hz, 3H), 1.01 – 0.95 (m, 2H), -0.01 (s, 9H); ^{13}C NMR (101 MHz, $CDCl_3$) δ 157.3, 153.6, 151.8, 141.3, 140.8, 136.9, 131.3, 129.3 (2C), 128.5 (2C), 127.2 (2C), 127.1 (3C), 103.1, 102.3, 70.7, 66.5, 65.0, 60.4, 31.8, 18.1, 16.1, -1.4 (3C); IR (neat, cm^{-1}): 3293 (br w), 3029 (w), 2951 (w), 2893 (w), 2874 (w), 1737 (w), 1565 (s), 1548 (m), 1495 (m), 1453 (m), 1413 (m), 1310 (m), 1247 (m), 1072 (m), 1048 (m), 1030 (m), 856 (m), 833 (s), 775 (m), 698 (m), 622 (w); HRMS (ASAP+, m/z): found 489.2682, calcd for $C_{28}H_{37}N_4O_2Si$ $[M+H]^+$, 489.2686.

(*R*)-(4-(4-((1-(4-(*Tert*-butyl)phenyl)ethyl)(methyl)amino)-7-((2-(trimethylsilyl)ethoxy)methyl)-7*H*-pyrrolo[2,3-*d*]pyrimidin-6-yl)phenyl)methanol (90)

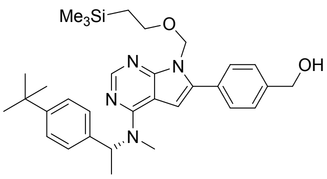

The compound was made as described in General Procedure B, but starting with **59** (65 mg, 0.12 mmol) and 4-hydroxymethylphenylboronic acid. The reaction time was 40 min. The crude product was purified by silica-gel column chromatography (*n*-pentane/EtOAc, 3:1, R_f = 0.27) giving 23 mg (42 μmol, 35%) of an orange oil; $[\alpha]_D^{20}$ = +56.5 (c 1.00, $CHCl_3$); ^{1}H NMR (400 MHz, $CDCl_3$) δ 8.40 (s, 1H), 7.71 - 7.69 (m, 2H), 7.45 - 7.43 (m, 2H), 7.37 - 7.35 (m, 2H), 7.29 - 7.27 (m, 2H), 6.62 (s, 1H), 6.46 (br.s, 1H), 5.58 (s, 2H), 4.75 (s, 2H), 3.78 - 3.73 (m, 2H), 3.09 (s, 3H), 1.88 (br.s, 1H), 1.65 (d, J = 6.9 Hz, 3H), 1.31 (s, 9H), 1.00 - 0.96 (m, 2H), -0.01 (s, 9H); ^{13}C NMR (101 MHz, $CDCl_3$) δ 157.3, 153.5, 151.8, 150.0, 140.8, 138.1, 136.8, 131.3, 129.3 (2C), 127.2 (2C), 126.8 (2C), 125.4 (2C), 103.1, 102.3, 70.7, 66.5, 65.0, 52.8, 34.4, 31.8, 31.4, 18.1, 16.2, -1.4 (3C); HRMS (ASAP+, m/z): found 545.3308, calcd for $C_{32}H_{45}N_4O_2Si$, $[M+H]^+$, 545.3312.

(4-(4-(Benzyl(methyl-*d*$_3$)amino)-7-((2-(trimethylsilyl)ethoxy)methyl)-7*H*-pyrrolo[2,3-*d*]pyrimidin-6-yl)phenyl)methanol (91)

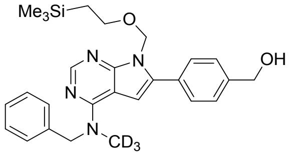

The compound was made as described in General Procedure B, but starting with **60** (200 mg, 0.402 mmol) and 4-hydroxymethylphenylboronic acid. The reaction time was 10 min. The crude product was purified by silica-gel column chromatography (*n*-pentane/EtOAc - 4:3, R_f =

0.23) giving 152 mg (0.319 mmol, 79%) of a faint yellow oil; ^{1}H NMR (400 MHz, $CDCl_3$) δ 8.40 (s, 1H), 7.71 – 7.64 (m, 2H), 7.47 – 7.40 (m, 2H), 7.38 – 7.22 (m, 5H), 6.55 (s, 1H), 5.57 (s, 2H), 5.06 (s, 2H), 4.75 (d, *J* = 5.8 Hz, 2H), 3.79 – 3.70 (m, 2H), 1.75 (t, *J* = 5.9 Hz, 1H), 1.02 – 0.91 (m, 2H), -0.02 (s, 9H); ^{13}C NMR (101 MHz, $CDCl_3$) δ 157.3, 153.5, 151.8, 140.8, 137.9, 137.1, 131.2, 129.3 (2C), 128.7 (2C), 127.3, 127.2 (2C), 127.1 (2C), 102.9, 102.0, 70.7, 66.5, 65.0, 53.8, 36.4 (HMBC), 18.0, -1.4 (3C); IR (neat, cm^{-1}): 3288 (br w), 3028 (w), 2950 (w), 2893 (w), 2871 (w), 2071 (w), 1736 (w), 1565 (s), 1493 (m), 1451 (m), 1358 (m), 1309 (m), 1246 (m), 1073 (s), 1028 (m), 856 (m), 833 (s), 769 (m), 696 (m); HRMS (ASAP+, m/z): found 478.2716, calcd for $C_{27}H_{32}D_3N_4O_2Si$ $[M+H]^+$ 478.2718.

(4-(4-(Methyl(2-methylbenzyl)amino)-7-((2-(trimethylsilyl)ethoxy)methyl)-7H-pyrrolo[2,3-d]pyrimidin-6-yl)phenyl)methanol (92)

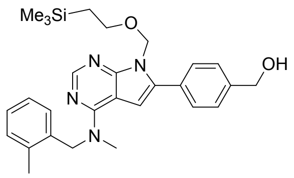

The material was made as described in General Procedure B, but starting with **61** (201 mg, 0.395 mmol) and 4-hydroxymethylphenylboronic acid. The reaction time was 40 min. The crude product was purified by silica-gel column chromatography (*n*-pentane/EtOAc - 2:1) giving 144 mg (0.295 mmol, 75%) of a faint yellow oil. TLC (silica, *n*-pentane/EtOAc - 1:1): R_f = 0.35; ^{1}H NMR (400 MHz, $CDCl_3$) δ 8.39 (s, 1H), 7.66 - 7.64 (m, 2H), 7.44 - 7.42 (m, 2H), 7.22 - 7.18 (m, 2H), 7.17 - 7.13 (m, 2H), 6.45 (s, 1H), 5.56 (s, 2H), 5.01 (s, 2H), 4.75 (d, *J* = 5.8 Hz, 2H), 3.76 - 3.72 (m, 2H), 3.38 (s, 3H), 2.32 (s, 3H), 1.74 (t, *J* = 5.8 Hz, 1H), 0.99 - 0.94 (m, 2H), -0.03 (s, 9H); ^{13}C NMR (100 MHz, $CDCl_3$) δ 157.3, 153.5, 151.7, 140.8, 137.1, 135.8, 135.4, 131.2, 130.5, 129.3, 127.2, 127.1, 126.4, 126.3, 102.9, 101.9, 70.7, 66.5, 65.0, 52.2, 37.3, 19.1, 18.0, -1.4 (3C); HRMS (ASAP+, m/z): found 489.2679, calcd for $C_{28}H_{37}N_4O_2Si$, $[M+H]^+$, 489.2686.

(4-(4-(Methyl(3-methylbenzyl)amino)-7-((2-(trimethylsilyl)ethoxy)methyl)-7*H*-pyrrolo[2,3-*d*]pyrimidin-6-yl)phenyl)methanol (93)

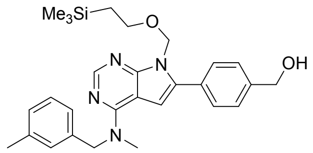

The material was prepared using the general procedure in General Procedure B, but using compound **62** (405 mg, 0.796 mmol), 4-hydroxymethylphenylboronic acid and Pd-PEPPSI-

SIPr as catalyst. The reaction time was 15 min. Purification by flash column chromatography (*n*-pentane/EtOAc - 2:1) gave 259 mg (0.529 mmol, 67%) of a clear oil. ^{1}H NMR (600 MHz, $CDCl_3$) δ 8.39 (s, 1H), 7.69 – 7.64 (m, 2H), 7.45 – 7.40 (m, 2H), 7.24 – 7.18 (m, 1H), 7.11 – 7.06 (m, 3H), 6.55 (s, 1H), 5.56 (s, 2H), 5.03 (s, 2H), 4.74 (d, *J* = 5.2 Hz, 2H), 3.77 – 3.71 (m, 2H), 3.36 (s, 3H), 2.32 (s, 3H), 1.94 (t, *J* = 5.3 Hz, 1H), 1.00 – 0.93 (m, 2H), -0.02 (s, 9H); ^{13}C NMR (151 MHz, $CDCl_3$) δ 157.3, 153.5, 151.7, 140.9, 138.4, 137.8, 137.1, 131.2, 129.3 (2C), 128.6, 128.0, 127.8, 127.2 (2C), 124.2, 102.9, 102.0, 70.7, 66.5, 65.0, 53.8, 37.2, 21.5, 18.0, -1.4 (3C); HRMS (ASAP+, m/z): found 489.2682, calcd for $C_{28}H_{37}N_4O_2Si$, $[M+H]^+$, 489.2686.

(4-(4-(Methyl(4-methylbenzyl)amino)-7-((2-(trimethylsilyl)ethoxy)methyl)-7*H*-pyrrolo[2,3-*d*]pyrimidin-6-yl)phenyl)methanol (94)

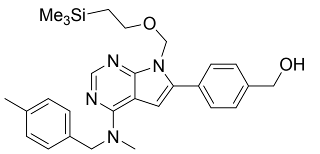

The material was made as described in General Procedure B, but starting with **63** (203 mg, 0.399 mmol) and 4-hydroxymethylphenylboronic acid. The reaction time was 45 min. The crude product was purified by silica-gel column chromatography (*n*-pentane/EtOAc - 2:1) giving 145 mg (0.297 mmol, 74%) of a faint yellow oil. TLC (silica, *n*-pentane/EtOAc - 1:1): R_f = 0.38; ^{1}H NMR (400 MHz, $CDCl_3$) δ 8.39 (s, 1H), 7.69 - 7.67 (m, 2H), 7.44 -7.42 (m, 2H), 7.19 - 7.17 (m, 2H), 7.15 - 7.13 (m, 2H), 6.55 (s, 1H), 5.57 (s, 2H), 5.02 (s, 2H), 4.75 (d, *J* = 5.9 Hz, 2H), 3.76 - 3.72 (m, 2H), 3.35 (s, 3H), 2.33 (s, 3H), 1.73 (t, *J* = 5.9 Hz, 1H), 0.99 - 0.95 (m, 2H), -0.02 (s, 9H); ^{13}C NMR (100 MHz, $CDCl_3$) δ: 157.4, 153.7, 151.6, 140.7, 140.0, 136.9, 131.2, 129.2, 128.5, 127.2, 126.7, 126.4, 102.7, 102.3, 70.7, 66.4, 65.0, 47.6, 46.8, 20.5, 18.0, -1.4 (3C); HRMS (ASAP+, m/z): found 489.2679, calcd for $C_{28}H_{37}N_4O_2Si$, $[M+H]^+$, 489.2686.

(4-(4-(Methyl(pyridin-2-ylmethyl)amino)-7-((2-(trimethylsilyl)ethoxy)methyl)-7*H*-pyrrolo[*2,3-d*]pyrimidin-6-yl)phenyl)methanol (95)

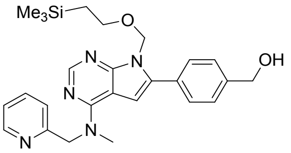

The material was made as described in General Procedure B, but starting with **64** (198 mg, 0.400 mmol) and 4-hydroxymethylphenylboronic acid. The reaction time was 45 min. The

crude product was purified by silica-gel column chromatography (CH_2Cl_2/MeOH - 19:1, R_f = 0.14) giving 153 mg (0.322 mmol, 80%) of a faint yellow oil. ^{1}H NMR (400 MHz, $CDCl_3$) δ 8.60 - 8.59 (m, 1H), 8.39 (s, 1H), 7.67 - 7.66 (m, 2H), 7.64 -7.60 (m, 1H), 7.44 - 7.42 (m, 2H), 7.27 - 7.25 (m, 1H), 7.20 - 7.17 (m, 1H), 6.54 (s, 1H), 5.56 (s, 2H), 5.18 (s, 2H), 4.75 (d, *J* = 5.7 Hz, 2H), 3.76 - 3.71 (m, 2H), 3.48 (s, 3H), 1.75 (t, *J* = 5.8 Hz, 1H), 0.98 - 0.94 (m, 2H), -0.03 (s, 9H); ^{13}C NMR (101 MHz, $CDCl_3$) δ 158.0, 156.7, 153.3, 151.3, 149.5, 141.0, 137.4, 137.0, 130.9, 129.3 (2C), 127.2 (2C), 122.3, 121.2, 102.9, 102.0, 70.7, 66.5, 64.9, 56.2, 38.1, 18.0, -1.4 (3C); HRMS (ASAP+, m/z): found 476.2477, calcd for $C_{26}H_{34}N_5O_2Si$, $[M+H]^+$, 476.2482.

(4-(4-(Methyl(pyridin-3-ylmethyl)amino)-7-((2-(trimethylsilyl)ethoxy)methyl)-7*H*-pyrrolo[2,3-*d*]pyrimidin-6-yl)phenyl)methanol (96)

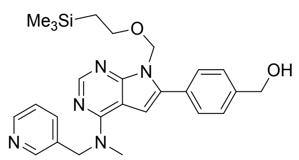

The material was made as described in General Procedure B starting with **65** (124 mg, 0.251 mmol) and 4-hydroxymethylphenylboronic acid. The reaction time was 30 min. Extraction was done using CH_2Cl_2. Purification was by silica-gel column chromatography (CH_2Cl_2/MeOH - 19:1, R_f = 0.11) giving 88 mg (0.19 mmol, 76%) of a faint yellow oil. ^{1}H NMR (400 MHz, $CDCl_3$) δ 8.58 - 8.51 (m, 2H), 8.38 (s, 1H), 7.69 - 7.64 (m, 3H), 7.45 - 7.43 (m, 2H), 7.27 - 7.23 (m, 1H), 6.58 (s, 1H), 5.56 (s, 2H), 5.07 (s, 2H), 4.75 (s, 2H), 3.76 - 3.72 (m, 2H), 3.39 (s, 3H), 0.99 - 0.95 (m, 2H), -0.02 (s, 9H); ^{13}C NMR (151 MHz, $CDCl_3$) δ 156.9, 153.4, 151.5, 148.9, 148.6, 141.3, 137.5, 135.3, 133.7, 130.8, 129.2 (2C), 127.2 (2C), 123.7, 103.0, 101.7, 70.7, 66.5, 64.7, 51.3, 37.4, 18.0, -1.4 (3C); HRMS (ASAP+, m/z): found 476.2477, calcd for $C_{26}H_{34}N_5O_2Si$ $[M+H]^+$ 476.2482.

(4-(4-(Methyl(pyridin-4-ylmethyl)amino)-7-((2-(trimethylsilyl)ethoxy)methyl)-*7H*-pyrrolo[2,3-*d*]pyrimidin-6-yl)phenyl)methanol (97)

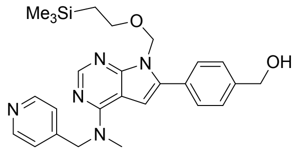

The material was made as described in General Procedure B, but starting with **66** (173 mg, 0.349 mmol) and 4-hydroxymethylphenylboronic acid. The reaction time was 20 min. The

crude product was purified by silica-gel column chromatography (CH_2Cl_2/MeOH - 97:3, R_f = 0.09) giving 141 mg (0.296 mmol, 85%) of a faint yellow oil. ^{1}H NMR (400 MHz, $CDCl_3$) δ 8.56 - 8.55 (m, 2H), 8.38 (s, 1H), 7.70 - 7.68 (m, 2H), 7.46 - 7.44 (m, 2H), 7.21-7.20 (m, 2H), 6.55 (s, 1H), 5.58 (s, 2H), 5.07 (s, 2H), 4.76 (d, J = 4.6 Hz, 2H), 3.76 - 3.72 (m, 2H), 3.43 (s, 3H), 1.78 (t, J = 4.8 Hz, 1H), 0.99 -0.95 (m, 2H), -0.02 (s, 9H); ^{13}C NMR (151 MHz, $CDCl_3$) δ 156.7, 153.4, 151.3, 149.0, 141.2, 137.9, 130.8, 129.3 (2C), 127.3 (2C), 122.5 (2C), 102.9, 101.6, 70.8, 66.6, 64.9, 53.3, 38.0, 18.0, -1.4 (3C); IR (neat, cm^{-1}): 696 (w); HRMS (ASAP+, m/z): found 476.2475, calcd for $C_{26}H_{34}N_5O_2Si$, $[M+H]^+$, 476.2482.

(4-(4-(Methyl((6-methylpyridin-2-yl)methyl)amino)-7-((2-(trimethylsilyl)ethoxy)methyl)-7*H*-pyrrolo[2,3-*d*]pyrimidin-6-yl)phenyl)methanol (98)

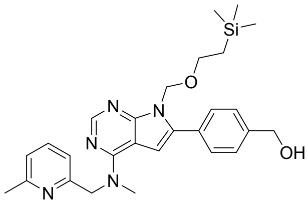

The material was made as described in General Procedure B, but starting with **67** (101 mg, 0.198 mmol) and 4-hydroxymethylphenylboronic acid. The reaction time was 10 min. Purification by flash column chromatography (10 g silica-gel cartridge, *n*-pentane/EtOAc - 1:5, 10 mL/min) yielding 90 mg (0.184 mmol, 93%) of a clear oil. ^{1}H NMR (400 MHz, $CDCl_3$) δ 8.39 (s, 1H), 7.70 – 7.63 (m, 2H), 7.54 – 7.46 (m, 1H), 7.50 – 7.40 (m, 2H), 7.07 – 6.99 (m, 2H), 6.53 (s, 1H), 5.56 (s, 2H), 5.13 (s, 2H), 4.75 (d, J = 5.1 Hz, 2H), 3.78 – 3.69 (m, 2H), 3.47 (s, 3H), 2.57 (s, 3H), 1.79 (t, J = 5.8 Hz, 1H), 1.02 – 0.90 (m, 2H), -0.03 (s, 9H); ^{13}C NMR (151 MHz, $CDCl_3$) δ 158.1, 157.3, 156.9, 153.3, 151.4, 140.9, 137.6, 137.4, 131.0, 129.3 (2C), 127.2 (2C), 122.0, 118.0, 102.9, 102.0, 70.7, 66.5, 65.0, 56.1, 38.1, 24.2, 18.0, -1.4 (3C); HRMS (ES+, m/z): found 490.2635, calcd for $C_{27}H_{36}N_5O_2Si$, $[M+H]^+$, 490.2634.

(4-(4-((2-Fluorobenzyl)(methyl)amino)-7-((2-(trimethylsilyl)ethoxy)methyl)-7*H*-pyrrolo[2,3-*d*]pyrimidin-6-yl)phenyl)methanol (99)

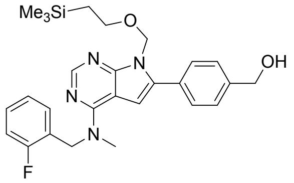

The material was made as described in General Procedure B, but starting with *N*-(2-fluorobenzyl)-6-iodo-*N*-methyl-7-((2-(trimethylsilyl)ethoxy)methyl)-7*H*-pyrrolo[2,3-*d*]pyrimidin-4-amine (**68**) (128 mg, 0.251 mmol) and 4-hydroxymethylphenylboronic acid.

The reaction time was 8 min. Purification by flash column chromatography (25 g silica-gel cartridge, *n*-pentane/EtOAc - 3:2, R_f = 0.27, 10 mL/min) yielding 104 mg (0.212 mmol, 85%) of a clear oil; ^{1}H NMR (400 MHz, $CDCl_3$) δ 8.39 (s, 1H), 7.72 – 7.64 (m, 2H), 7.47 – 7.41 (m, 2H), 7.31 – 7.21 (m, 2H), 7.11 – 7.04 (m, 2H), 6.54 (s, 1H), 5.56 (s, 2H), 5.12 (s, 2H), 4.75 (d, *J* = 5.8 Hz, 2H), 3.78 – 3.68 (m, 2H), 3.42 (s, 3H), 1.78 (t, *J* = 5.9 Hz, 1H), 1.02 – 0.91 (m, 2H), -0.03 (s, 9H); ^{13}C NMR (101 MHz, $CDCl_3$) δ 161.0 (d, *J* = 245.3 Hz), 157.2, 153.5, 151.7, 140.9, 137.2, 131.1, 129.3 (2C), 128.9 (d, *J* = 4.2 Hz), 128.8 (d, *J* = 4.2 Hz), 127.2 (2C), 124.9 (d, *J* = 14.6 Hz), 124.4 (d, *J* = 3.6 Hz), 115.4, 103.0, 101.9, 70.7, 66.5, 65.0, 47.6 (d, *J* = 3.3 Hz), 37.6, 18.0, -1.4 (3C); ^{19}F NMR (376 MHz, $CDCl_3$) δ -118.90 – -118.99 (m); IR (neat, cm^{-1}): 3306 (br w), 2950 (w), 2894 (w), 2873 (w), 1737 (w), 1568 (s), 1499 (m), 1487 (m), 1455 (m), 1415 (m), 1309 (m), 1247 (m), 1073 (m), 1036 (m), 856 (m), 831 (s), 756 (s); HRMS (ES+, m/z): found 493.2431, calcd for $C_{27}H_{34}N_4O_2FSi$, $[M+H]^+$, 493.2435.

2-(((6-(4-(Hydroxymethyl)phenyl)-7-((2-(trimethylsilyl)ethoxy)methyl)-7*H*-pyrrolo[2,3-*d*]pyrimidin-4-yl)(methyl)amino)methyl)phenol (100)

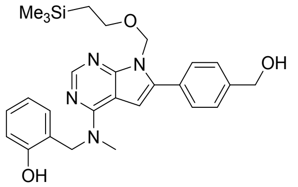

The material was made as described in General Procedure B, but starting with 2-(((6-iodo-7-((2-(trimethylsilyl)ethoxy)methyl)-7*H*-pyrrolo[2,3-*d*]pyrimidin-4-yl)(methyl)amino)methyl)phenol (**69**) (62 mg, 0.122 mmol) and 4-hydroxymethylphenylboronic acid. The reaction time was 10 min. Purification by flash column chromatography (10 g silica-gel cartridge, *n*-pentane/EtOAc - 3:2, R_f = 0.20, 10 mL/min) yielding 50 mg (0.103 mmol, 84%) of a clear oil. ^{1}H NMR (600 MHz, $CDCl_3$) δ 12.03 (s, 1H), 8.39 (s, 1H), 7.73 – 7.68 (m, 2H), 7.48 – 7.44 (m, 2H), 7.31 – 7.23 (m, 2H), 6.98 – 6.94 (m, 1H), 6.88 – 6.83 (m, 1H), 6.69 (s, 1H), 5.56 (s, 2H), 4.78 – 4.74 (m, 4H), 3.74 – 3.68 (m, 2H), 3.52 (s, 3H), 1.81 (s, 1H), 0.99 – 0.92 (m, 2H), -0.03 (s, 9H); ^{13}C NMR (151 MHz, $CDCl_3$) δ 156.9, 152.7, 150.2, 141.2, 138.0, 131.4, 130.7, 130.2, 129.4 (2C), 127.2 (2C), 122.6, 119.2, 117.9, 103.2, 102.1, 70.8, 66.6, 64.9, 51.9, 37.3, 18.0, -1.4 (3C); HRMS (ES+, m/z): found 491.2472, calcd for $C_{27}H_{35}N_4O_3Si$, $[M+H]^+$, 491.2478.

2-(((6-(4-(Hydroxymethyl)phenyl)-7-((2-(trimethylsilyl)ethoxy)methyl)-7*H*-pyrrolo[2,3-*d*]pyrimidin-4-yl)(methyl)amino)methyl)-4-methylphenol (101)

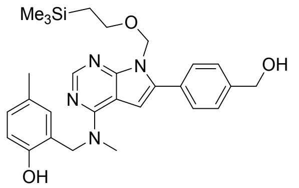

The material was made as described in General Procedure B, but starting with 2-(((6-iodo-7-((2-(trimethylsilyl)ethoxy)methyl)-7*H*-pyrrolo[2,3-*d*]pyrimidin-4-yl)(methyl)amino)methyl)-4-methylphenol (**70**) (150 mg, 0.287 mmol) and 4-hydroxymethylphenylboronic acid. The reaction time was 8 min. Purification by flash column chromatography (25 g silica-gel cartridge, *n*-pentane/EtOAc - 3:2, R_f = 0.22, 10 mL/min) yielding 110 mg (0.217 mmol, 76%) of a clear oil. ^{1}H NMR (600 MHz, $CDCl_3$) δ 11.75 (s, 1H), 8.37 (s, 1H), 7.73 – 7.68 (m, 2H), 7.48 – 7.44 (m, 2H), 7.06 (d, *J* = 7.6 Hz, 2H), 6.87 – 6.83 (m, 1H), 6.68 (s, 1H), 5.56 (s, 2H), 4.77 (s, 2H), 4.71 (s, 2H), 3.74 – 3.68 (m, 2H), 3.52 (s, 3H), 2.30 (s, 3H), 1.77 (s, 1H), 0.98 – 0.91 (m, 2H), -0.03 (s, 9H); ^{13}C NMR (151 MHz, $CDCl_3$) δ 155.9, 154.6, 152.9, 150.5, 141.1, 137.8, 131.9, 130.9, 130.7, 129.4 (2C), 128.1, 127.2 (2C), 122.4, 117.6, 103.2, 102.1, 70.8, 66.6, 65.0, 51.9, 37.3, 20.4, 18.0, -1.4 (3C); HRMS (ASAP+, m/z): found 505.2630, calcd for $C_{28}H_{37}N_4O_3Si$ $[M+H]^+$, 505.2635.

(*S*)-2-((6-(4-(Hydroxymethyl)phenyl)-7-((2-(trimethylsilyl)ethoxy)methyl)-7*H*-pyrrolo[2,3-*d*]pyrimidin-4-yl)(methyl)amino)-2-phenylethan-1-ol (102)

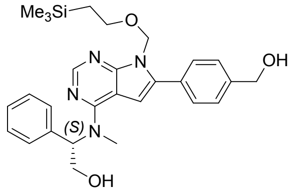

The material was made as described in General Procedure B, but starting with (*S*)-2-((6-iodo-7-((2-(trimethylsilyl)ethoxy)methyl)-7*H*-pyrrolo[2,3-*d*]pyrimidin-4-yl)(methyl)amino)-2-phenylethan-1-ol (**71**) (180 mg, 0.345 mmol) and 4-hydroxymethylphenylboronic acid. The reaction time was 13 min. Purification by flash column chromatography (25 g silica-gel cartridge, CH_2Cl_2/MeOH - 95:5, R_f = 0.15, 10 mL/min) yielded 154 mg (0.306 mmol, 89%) of an orange powder. $[\alpha]_D^{20}$= +27.3 (*c* 0.99, $CHCl_3$); ^{1}H NMR (400 MHz, $CDCl_3$) δ 8.35 (s, 1H), 7.75 – 7.68 (m, 2H), 7.49 – 7.41 (m, 2H), 7.40 – 7.27 (m, 5H), 6.65 (s, 1H), 6.32 (dd, *J* = 9.5, 4.3 Hz, 1H), 5.62 – 5.53 (m, 2H), 4.76 (d, *J* = 4.3 Hz, 2H), 4.40 – 4.33 (m, 1H), 4.33 – 4.25 (m, 1H), 3.83 (s, 1H), 3.79 – 3.71 (m, 2H), 3.17 (s, 3H), 1.81 (t, *J* = 5.1 Hz, 1H), 1.02 – 0.92 (m, 2H), -0.01 (s, 9H); ^{13}C NMR (101 MHz, $CDCl_3$) δ 158.5, 153.5, 151.2, 141.0, 137.6,

137.4, 131.0, 129.3 (2C), 128.8 (2C), 127.75, 127.71 (2C), 127.2 (2C), 103.7, 102.1, 70.7, 66.6, 65.0, 62.7, 60.7, 33.6, 18.0, -1.4 (3C); IR (neat, cm^{-1}): 3298 (br w), 3058 (w), 3029 (w), 2949 (w), 2876 (w), 1566 (s), 1495 (m), 1453 (m), 1412 (m), 1308 (m), 1247 (m), 1069 (m), 1024 (m), 856 (m), 832 (s), 760 (m), 697 (m), 624 (m); HRMS (ASAP+, m/z): found 505.2632, calcd for $C_{28}H_{37}N_4O_3Si$, $[M+H]^+$, 505.2635.

(*R*)-2-((6-(4-(Hydroxymethyl)phenyl)-7-((2-(trimethylsilyl)ethoxy)methyl)-7*H*-pyrrolo[2,3-*d*]pyrimidin-4-yl)(methyl)amino)-2-phenylethan-1-ol (103)

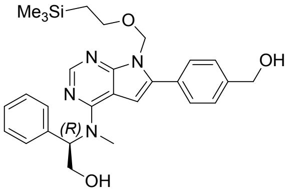

The material was made as described in General Procedure B, but starting with (*R*)-2-((6-iodo-7-((2-(trimethylsilyl)ethoxy)methyl)-7*H*-pyrrolo[2,3-*d*]pyrimidin-4-yl)(methyl)amino)-2-phenylethan-1-ol (**72**) (162 mg, 0.308 mmol) and 4-hydroxymethylphenylboronic acid. The reaction time was 10 min. Purification by flash column chromatography (25 g silica-gel cartridge, CH_2Cl_2/MeOH - 97:3, R_f = 0.06, 10 mL/min) yielded 140 mg (0.278 mmol, 90%) of a yellow oil. $[\alpha]_D^{20}$= -26.0 (*c* 1.00, $CHCl_3$); ^1H NMR (400 MHz, $CDCl_3$) δ 8.35 (s, 1H), 7.75 – 7.68 (m, 2H), 7.49 – 7.42 (m, 2H), 7.41 – 7.25 (m, 5H), 6.65 (s, 1H), 6.32 (dd, *J* = 9.5, 4.3 Hz, 1H), 5.62 – 5.54 (m, 2H), 4.76 (d, *J* = 5.2 Hz, 2H), 4.41 – 4.33 (m, 1H), 4.33 – 4.24 (m, 1H), 3.85 – 3.80 (m, 1H), 3.79 – 3.71 (m, 2H), 3.17 (s, 3H), 1.81 (t, *J* = 5.8 Hz, 1H), 1.03 – 0.92 (m, 2H), -0.01 (s, 9H); ^{13}C NMR (101 MHz, $CDCl_3$) δ 158.5, 153.5, 151.2, 141.0, 137.6, 137.4, 131.0, 129.3 (2C), 128.8 (2C), 127.75, 127.71 (2C), 127.2 (2C), 103.7, 102.1, 70.7, 66.6, 65.0, 62.7, 60.7, 33.6, 18.0, -1.4 (3C); IR (neat, cm^{-1}): 3297 (br w), 3059 (w), 3030 (w), 2949 (w), 2889 (w), 1566 (s), 1495 (m), 1453 (m), 1413 (m), 1308 (m), 1247 (m), 1069 (m), 1023 (m), 856 (m), 832 (s), 761 (m), 734 (m), 670 (s), 624 (m); HRMS (ASAP+, m/z): found 505.2636, calcd for $C_{28}H_{37}N_4O_3Si$, $[M+H]^+$, 505.2635.

***N*-Methyl-*N*-(3-methylbenzyl)-6-phenyl-7-((2-(trimethylsilyl)ethoxy)methyl)-7*H*-pyrrolo[2,3-*d*]pyrimidin-4-amine (104)**

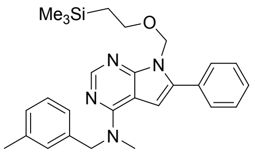

The material was made as described in General Procedure B, but starting with **62** (260 mg, 0.510 mmol) and phenylboronic acid. The reaction time was 10 min. Silica-gel column chromatography purification (*n*-pentane/EtOAc - 1:1, R_f = 0.73) gave 196 mg (0.43 mmol, 84%) of a honey coloured oil; ^{1}H NMR (600 MHz, DMSO-d_6) δ 8.25 (s, 1H), 7.71 (d, J = 7.2 Hz, 2H), 7.50 – 7.45 (m, 2H), 7.44 – 7.38 (m, 1H), 7.20 (t, J = 7.6 Hz, 1H), 7.08 (s, 1H), 7.08 – 7.02 (m, 2H), 6.80 (s, 1H), 5.55 (s, 2H), 5.02 (s, 2H), 3.61 (dd, J = 8.6, 7.5 Hz, 2H), 3.35 (s, 3H), 2.26 (s, 3H), 0.84 (dd, J = 8.5, 7.6 Hz, 2H), -0.10 (s, 9H); ^{13}C NMR (151 MHz, DMSO-d_6) δ 156.5, 153.1, 151.3, 138.2, 137.6, 136.4, 131.5, 128.7 (2C), 128.5(2C), 128.4, 128.1, 127.6, 127.5, 124.0, 102.4, 102.1, 70.3, 65.7, 54.9, 39.5, 37.4, 21.1, 17.3, -1.4 (3C); IR (neat, cm^{-1}): 3027 (s, w), 2950 (w), 2893 (w), 1567 (s), 1413 (s), 1306 (s), 1246 (s), 1070 (s), 856 (s), 832 (s), 775 (s), 752 (s), 695 (s); HRMS (ES+, m/z): found 459.2582, calcd for $C_{27}H_{34}N_4OSi$, $[M+H]^+$, 459.2501.

4-(4-(Methyl(3-methylbenzyl)amino)-7-((2-(trimethylsilyl)ethoxy)methyl)-7*H*-pyrrolo[2,3-*d*]pyrimidin-6-yl)phenol (105)

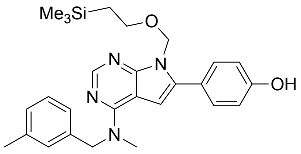

The material was made as described in General Procedure B, but starting with **62** (266 mg, 0.52 mmol) and (4-hydroxyphenyl)boronic acid. The reaction time was 5 min. Silica-gel column chromatography purification (*n*-pentane/EtOAc, gradient 7:1 to 1:1, *n*-pentane/EtOAc 1:1, R_f = 0.78) gave 196 mg (0.41 mmol, 78%) of an off-white wax; ^{1}H NMR (600 MHz, DMSO-d_6) δ: 9.69 (s, 1H), 8.21 (s, 1H), 7.53 – 7.48 (m, 2H), 7.20 (t, *J* = 7.5 Hz, 1H), 7.09 – 6.97 (m, 3H), 6.87 – 6.82 (m, 2H), 6.64 (s, 1H), 5.49 (s, 2H), 5.00 (s, 2H), 3.64 – 3.58 (m, 2H), 3.33 (s, 3H), 2.26 (s, 3H), 0.87 – 0.81 (m, 2H), -0.08 (s, 9H); ^{13}C NMR (151 MHz, DMSO-d_6) δ 157.6, 156.3, 152.8, 150.9, 138.3, 137.6, 136.8, 130.0 (2C), 128.4, 127.6, 127.5, 124.0, 122.1, 115.5 (2C), 102.1, 100.8, 70.2, 65.6, 52.7, 39.5, 37.3, 21.7, 17.3, -1.3(3C). HRMS (ES+, m/z): found 475.2531, calcd for $C_{27}H_{34}N_4O_2Si$, $[M+H]^+$, 475.2451.

6-(4-Fluorophenyl)-*N*-methyl-N-(3-methylbenzyl)-7-((2-(trimethylsilyl)ethoxy)methyl)-7*H*-pyrrolo[2,3-*d*]pyrimidin-4-amine (106)

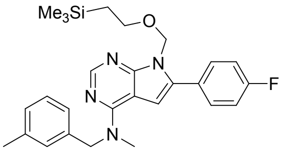

The material was made as described in General Procedure B, but starting **62** (260 mg, 0.510 mmol) and (4-fluorophenyl)boronic acid. The reaction time was 10 min. Silica-gel column chromatography purification (*n*-pentane/EtOAc - 3:2, R_f = 0.68) gave 234 mg (0.49 mmol, 96%) of a brown oil; ^{1}H NMR (600 MHz, DMSO-d_6) δ 8.25 (s, 1H), 7.78 – 7.73 (m, 2H), 7.36 – 7.29 (m, 2H), 7.21 (t, *J* = 7.5 Hz, 1H), 7.10 – 7.02 (m, 3H), 6.81 (s, 1H), 5.54 (s, 2H), 5.02 (s, 2H), 3.63 – 3.58 (m, 2H), 3.35 (s, 3H), 2.26 (s, 3H), 0.84 (m, 2H), -0.09 (s, 9H);^{13}C NMR (151 MHz, DMSO-d_6) δ 162.3 (d, J = 248.0 Hz), 156.5, 154.9, 153.0, 152.1, 151.3, 151.2, 138.2, 137.6, 135.3, 130.7 (d, J = 8.3 Hz, 2C), 128.4, 127.9 (d, J = 3.3 Hz), 127.6, 127.5, 124.1, 115.7 (d, J = 22.3 Hz, 2C), 102.5, 102.0, 70.2, 65.7, 54.9, 39.5, 37.3, 21.1, 17.3, -1.5 (3C); IR (neat, cm^{-1}): 3024 (w), 2950 (s), 2894 (s), 1567 (s), 1547 (s), 1496 (s), 1413 (s), 1308(s), 1246 (s), 1072 (s), 857(s), 833 (s), 765 (s), 732 (s), 692(s). HRMS (ES+, m/z): found 477.2485, calcd for $C_{27}H_{33}FN_4OSi$, $[M+H]^+$, 477.2407.

6-(3-Fluoro-4-methoxyphenyl)-*N*-methyl-*N*-(3-methylbenzyl)-7-((2-(trimethylsilyl)ethoxy)methyl)-7*H*-pyrrolo[2,3-*d*]pyrimidin-4-amine (107)

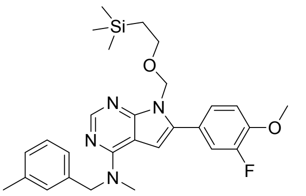

The material was made as described in General Procedure B, but starting **62** (86 mg, 0.17 mmol) and (3-fluoro-4-methoxyphenyl)boronic acid. The reaction time was 5 min. Silica-gel column chromatography purification (*n*-pentane/EtOAc - 2:1, R_f = 0.52) gave 51 mg (0.1 mmol, 59%) of a white solid wax; ^{1}H NMR (600 MHz, DMSO-d_6) δ 8.22 (s, 1H), 7.61 (m, 1H), 7.50 (m, 1H), 7.26 (t, *J* = 8.8 Hz, 1H), 7.19 (t, *J* = 7.5 Hz, 1H), 7.08 – 7.01 (m, 3H), 6.80 (s, 1H), 5.53 (s, 2H), 5.00 (s, 2H), 3.88 (s, 3H), 3.59 (s, 3H), 3.34 (s, 3H), 2.25 (s, 3H), 0.88 – 0.81 (m, 2H), -0.09 (s, 9H); ^{13}C NMR (151 MHz, DMSO-d_6) δ 156.4, 152.9, 152.3, 151.0 (d, J = 245 Hz), 147.09, 147.0 (d = 10.0 Hz), 138.2, 137.6, 135.0, 128.4, 127.6, 127.5, 124.9 (d, J = 3.0 Hz), 124.3 (d, J = 7.0 Hz), 124.0, 115.9 (d = 20.0 Hz), 114.1 (d, J = 3.0 Hz) , 102.2, 102.0, 70.2, 65.7, 56.1, 52.7, 39.5, 37.4, 21.1, 17.3, -1.4 (3C); HRMS (ES+, m/z): found 507.2595, calcd for $C_{28}H_{35}FN_4O_2Si$, $[M+H]^+$, 507.2513.

***N*-Methyl-*N*-(3-methylbenzyl)-6-(pyridin-3-yl)-7-((2-(trimethylsilyl)ethoxy)methyl)-7*H*-pyrrolo[2,3-*d*]pyrimidin-4-amine (108)**

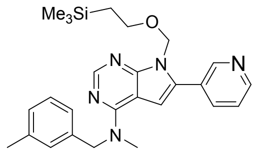

The material was made as described in General Procedure B, but starting with **62** (262 mg, 0.51 mmol) and pyridin-3-ylboronic acid. The reaction time was 50 min. Silica-gel column chromatography purification (*n*-pentane/EtOAc - 5:3, R_f = 0.35) gave 190 mg (0.41 mmol, 81%) of an off-white solid wax; ^{1}H NMR (600 MHz, DMSO-d_6) δ 8.93 – 8.90 (m, 1H), 8.61 (dd, *J* = 4.8, 1.6 Hz, 1H), 8.27 (s, 1H), 8.18 – 8.13 (m, 1H), 7.55 – 7.49 (m, 1H), 7.22 (t, *J* = 7.5 Hz, 1H), 7.11 – 7.04 (m, 3H), 7.00 (s, 1H), 5.59 (s, 2H), 5.04 (s, 2H), 3.64 – 3.58 (m, 2H), 3.38 (s, 3H), 2.27 (s, 3H), 0.88 – 0.82 (m, 2H), -0.09 (s, 9H);^{13}C NMR (151 MHz, DMSO-d_6) δ 156.6, 153.3, 151.6, 148.9, 148.8, 138.1, 137.6, 135.5, 132.9, 128.4, 127.6 (3C), 124.1, 123.6, 103.6, 102.1, 70.3, 65.7, 52.7, 39.5, 37.4, 21.1, 17.3, -1.4 (3C); HRMS (ES+, m/z): found 460.2532, calcd. for $C_{26}H_{33}N_5OSi$, $[M+H]^+$, 460.2454.

6-(4-(2-(2-(2-methoxyethoxy)ethoxy)ethoxy)phenyl)-*N*-methyl-*N*-(3- methylbenzyl)-7-((2-(trimethylsilyl)ethoxy)methyl)-7*H*-pyrrolo[2,3-*d*]pyrimidin-4-amine (109)

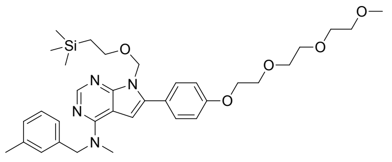

The reaction was performed as describe in General Procedure B, but using the pyrrolopyrimidine **62** (166 mg, 0.326 mmol), 2-(4-(2,5,8,11-tetraoxadodecyl)phenyl)-4,4,5,5-tetramethyl-1,3,2-dioxaborolane and $Pd(PPh_3)_4$ (17.6 mg) at 100 °C. The reaction time was 45 min. The crude product was purified by silica-gel column chromatography (diethyl ether/*n*-pentane - 5:1, R_f = 0.24). This gave 129 mg (0.208 mmol, 64%) of an oil; ^{1}H NMR (400 MHz, $CDCl_3$) δ 8.41 (s, 1H), 7.63 - 7.59 (m, 2H), 7.27 - 7.21 (m, 1H), 7.13 - 7.08 (m, 3H), 7.02 - 6.97 (m, 2H), 6.51 (s, 1H), 5.57 (s, 2H), 5.05 (s, 2H), 4.23 - 4.15 (m, 2H), 3.93 - 3.87 (m 2H), 3.78 - 3.65 (m, 8H), 3.58 - 3.54 (m, 2H), 3.38 (s, 3H), 3.36 (s, 3H), 2.32 (s, 3H), 0.99 - 0.96 (m, 2H), -0.02 (s, 9H); ^{13}C NMR (100 MHz, $CDCl_3$) δ 158.9, 157.2, 153.3, 151.5, 138.4, 137.9, 137.2, 130.5 (2C), 128.6, 128.1, 127.8, 124.5, 124.3, 114.8 (2C), 102.9, 101.2, 72.0 , 70.9, 70.7, 70.6, 70.6, 69.7, 67.5, 66.4, 59.1, 37.2, 21.5, 18.1, -1.4 ; IR (neat cm^{-1}): 2948 (w), 2921 (w), 2873 (w), 1568 (s), 1498 (s), 1414 (m), 1307 (m), 1247 (s), 1070 (s), 834 (s), 767 (m), 693 (m); HRMS (ASCI/ASAP, m/z): found 621.3466, calcd for $C_{34}H_{48}N_4O_5Si$, $[M+H]^+$, 621.3472

N-Methyl-N-(3-methylbenzyl)-6-(4-(trifluoromethyl)phenyl)-7-((2-(trimethylsilyl)ethoxy)methyl)-7H-pyrrolo[2,3-d]pyrimidin-4-amine (110)

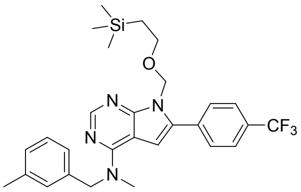

The material was made as described in General Procedure B, but starting with **62** (144 mg, 0.28 mmol) and (4-(trifluoromethyl)phenyl)boronic acid. The reaction time was 5 min. Silica-gel column chromatography purification (*n*-pentane/EtOAc - 3:1, $R_f = 0.64$) gave 103 mg (0.22 mmol, 79%) of an off-white solid wax; ^{1}H NMR (600 MHz, DMSO-d_6) δ 8.28 (s, 1H), 7.97 (d, $J = 7.9$ Hz, 2H), 7.85 (d, $J = 8.2$ Hz, 2H), 7.22 (t, $J = 7.6$ Hz, 1H), 7.10 – 7.04 (m, 3H), 7.01 (s, 1H), 5.62 (s, 2H), 5.04 (s, 2H), 3.65 – 3.59 (m, 2H), 3.38 (s, 3H), 2.28 (s, 3H), 0.87 – 0.83 (m, 2H), -0.09 (s, 9H); ^{13}C NMR (151 MHz, DMSO-d_6) δ 156.7, 153.5, 151.8, 138.1, 137.7, 135.6, 134.6, 128.9 (2C), 128.4, 127.9 (q, J = 30 Hz), 127.6, 127.5, 125.6 (q, J = 3.2 Hz, 2C), 124.1, 126.1 (q, J = 273 Hz), 104.2, 102.1, 70.3, 66.3, 65.7, 52.7, 39.5, 37.4, 21.1, 17.3, -1.5 (3C); HRMS (ES+, m/z): found 527.2453, calcd for $C_{28}H_{33}F_3N_4OSi$, $[M+H]^+$; 527.2375

Methyl 4-(4-(methyl(3-methylbenzyl)amino)-7-((2-(trimethylsilyl)ethoxy)methyl)-7H-pyrrolo[2,3-d]pyrimidin-6-yl)benzoate (111)

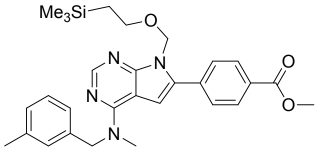

The material was made as described in General Procedure B, but starting with **62** (2.61 g, 5.1 mmol) and (4-(methoxycarbonyl)phenyl)boronic acid. The reaction time was 15 min. Silica-gel column chromatography purification (*n*-pentane/EtOAc - 3:1, $R_f = 0.70$) gave 2.57 g (4.9 mmol, 96%) of a brown oil; ^{1}H NMR (400 MHz, DMSO-d_6) δ 8.26 (s, 1H), 8.03 (d, $J = 8.5$ Hz, 2H), 7.90 (d, $J = 8.5$ Hz, 2H), 7.20 (t, $J = 7.5$ Hz, 1H), 7.02 – 7.09 (m, 3H), 7.01 (s, 1H), 5.59 (s, 2H), 5.03 (s, 2H), 3.88 (s, 3H), 3.67 – 3.57 (m, 2H), 3.37 (s, 3H), 2.26 (s, 3H), 0.89 - 0.81 (m, 2H), -0.09 (s, 9H); ^{13}C NMR (151 MHz, DMSO-d_6) δ 165.8, 156.6, 153.5, 151.7, 138.0, 137.6, 136.0, 134.9, 130.5, 129.4 (2C), 128.4, 128.3 (2C), 127.6 (2C),124.0, 104.0, 102.1, 70.4, 65.7, 54.2,52.1, 37.4, 21.0, 17.2, -1.5 (3C); IR (neat, cm^{-1}): 3015 (w), 2949 (s), 1720(s), 1566 (s), 1434(s), 1413(s), 1274 (s), 1187 (s), 1073 (s), 833(s), 761 (s), 702 (s). HRMS (ES+, m/z): found 517.2632, calcd for $C_{29}H_{36}N_4O_3Si$, $[M+H]^+$, 517.2556.

Methyl 5-(4-(4-(methyl(3-methylbenzyl)amino)-7-((2-(trimethylsilyl)ethoxy)methyl)-7*H*-pyrrolo[2,3-*d*]pyrimidin-6-yl)phenyl)-pentanoate (112)

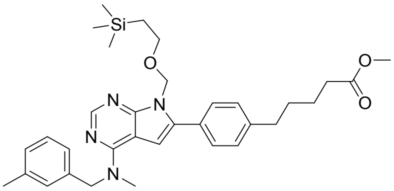

The compound was prepared as described in General Procedure B, using **62** (668 mg, 1.31 mmol) and methyl 5-(4-(4,4,5,5-tetramethyl-1,3,2-dioxaborolan-2-yl)-phenyl)pentanoate.The reaction time was 30 min. Purification by silica-gel chromatography (*n*-pentane/EtOAc - 3:1, $R_f = 0.25$) gave 608 mg (1.06 mmol, 81%) of a yellow oil; ^{1}H NMR (400 MHz, $CDCl_3$) δ 8.40 (s, 1H), 7.59 - 7.57 (m, 2H), 7.26 - 7.20 (m, 3H), 7.09 - 7.07 (m, 3H), 6.53 (s, 1H), 5.57 (s, 2H), 5.02 (s, 2H), 3.75 - 3.71 (m, 2H), 3.67 (s, 3H), 3.35 (s, 3H), 2.69 - 2.65 (m, 2H), 2.37 - 2.32 (m, 3H), 1.72 - 1.68 (m, 4H), 0.99 - 0.94 (m, 2H), -0.03 (s, 9H); ^{13}C NMR (100 MHz, $CDCl_3$) δ 174.1, 157.4, 153.5, 151.7, 142.4, 138.5, 137.9, 137.5, 129.5, 129.2 (2C), 128.8 (2C), 128.7, 128.1, 127.9, 124.3, 103.0, 101.8, 70.8, 66.5, 53.9, 51.6, 37.3, 35.4, 34.0. 30.9, 24.7, 21.6, 18.2, -1.3 (3C); IR (neat, cm^{-1}): 3022 (w), 2948 (w), 2862 (w), 1737 (s), 1568 (s), 1455 (w), 1414 (m), 1338 (w), 1309 (m), 1248 (m), 1076 (m), 1038 (w), 857 (w), 835 (m), 771 (m), 694 (w); HRMS (ASAP+, m/z): found 573.3259, calcd for $C_{33}H_{45}N_4O_3Si$, $[M+H]^+$, 573.3261.

Methyl 7-(4-(4-(methyl(3-methylbenzyl)amino)-7-((2-(trimethylsilyl)ethoxy)methyl)-7*H*-pyrrolo[2,3-*d*]pyrimidin-6-yl)phenyl)-7-oxoheptanoate (113)

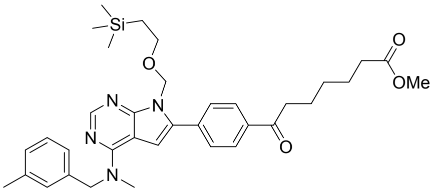

The compound was prepared as described in General Procedure B, using **62** (114 mg, 0.224 mmol) and methyl 7-oxo-7-(4-(4,4,5,5-tetramethyl-1,3,2-dioxaborolan-2-yl) phenyl)heptanoate (78.8 mg, 0.219 mmol), The reaction time was 1.5 h. Purification by silica-gel chromatography (*n*-pentane/EtOAc - 3:1, $R_f = 0.28$) gave 85.5 mg (0.139 mmol, 62%) as a yellow oil; ^{1}H NMR (400 MHz, $CDCl_3$) δ 8.40 (s, 1H), 8.01-7.99 (m, 2H), 7.81 - 7.79 (m, 2H), 7.23 - 7.19 (t, J = 7.2 Hz, 1H), 7.09 - 7.07 (m, 3H), 6.66 (s, 1H), 5.59 (s, 2H), 5.03 (s, 2H), 3.80 - 3.76 (m, 2H), 3.66 (s, 3H), 3.37 (s, 3H), 3.00 -2.97 (t, J = 7.1 Hz, 2H), 2.35 - 2.32 (m, 5H), 1.81 - 1.65 (m, 4H), 1.46 - 1.39 (m, 2H), 1.01 - 0.97 (m, 2H), -0.02 (s, 9H); ^{13}C NMR (100 MHz, $CDCl_3$) δ 199.6, 174.2, 157.5, 154.1, 152.3, 138.5, 137.7, 136.4, 136.2, 136.1, 128.9, (2C), 128.7, 128.5 (2C), 128.2, 127.8, 124.3, 103.5, 103.0, 70.9, 66.7, 53.9, 51.6, 38.4, 37.4,

34.0. 28.9, 24.8, 24.0, 21.6 18.1, -1.3 (3C); IR (neat, cm^{-1}) : 3023 (w), 2948 (w), 2899 (w), 1735 (s), 1681 (s) 1549 (s), 1455 (w), 1309 (m), 1247 (m), 1072 (m), 856 (m), 833 (m), 772 (m), 694 (m); HRMS (ASAP+, m/z): found 615.3370, calcd. for $C_{35}H_{47}N_4O_4Si$, $[M+H]^+$, 615.3367.

2-Fluoro-4-(4-(methyl(3-methylbenzyl)amino)-7-((2-(trimethylsilyl)ethoxy)methyl)-7*H*-pyrrolo[2,3-*d*]pyrimidin-6-yl)benzoic acid (114)

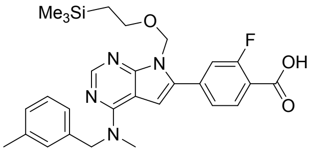

The material was made as described in General Procedure B, but starting with **62** (44 mg, 0.09 mmol) and 4-borono-2-fluorobenzoic acid. The reaction time was 5 min. Silica-gel column chromatography purification (*n*-pentane/EtOAc/AcOH - 175:75:1, R_f = 0.21) gave 36 mg (0.069 mmol, 79%) of a brown oil; ^1H NMR (600 MHz, DMSO-*d_6*) δ 8.35 (s, 1H), 7.97 – 7.92 (m, 1H), 7.79 – 7.68 (m, 2H), 7.29 (t, *J* = 7.6 Hz, 1H), 7.17 – 7.12 (m, 4H), 5.70 (s, 2H), 5.12 (s, 2H), 3.77 – 3.70 (m, 2H), 3.45 (s, 3H), 2.35 (s, 3H), 0.99 – 0.91 (m, 2H), 0.01 (s, 9H); ^{13}C NMR (151 MHz, DMSO-*d_6*) δ 168.3, 163.6 (d, J = 260 Hz), 156.7, 153.55, 151.9, 143.3, 138.06, 137.6, 131.7, 128.5, 127.7, 127.6, 124.1, 123.6 (d, J = 2.4 Hz), 115.7 (d, J = 24 Hz), 102.0, 100.6, 70.4, 65.8, 54.9, 39.52, 37.5, 21.05, 17.28, -1.43 (3C); HRMS (ES+, m/z): found 521.2384, calcd for $C_{28}H_{33}FN_4O_3Si$, $[M+H]^+$, 521.2305.

3-(4-(4-(Methyl(3-methylbenzyl)amino)-7-((2-(trimethylsilyl)ethoxy)methyl)-7H-pyrrolo[2,3-d]pyrimidin-6-yl)phenyl)propanoic acid (115)

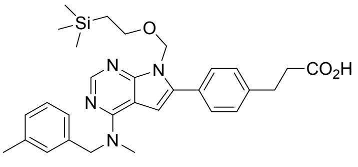

The material was made as described in General Procedure B, but starting with **62** (239mg, 0.47 mmol) and 3-(4-boronophenyl)propanoic acid The reaction time was 15 min. Silica-gel column chromatography purification (*n*-pentane/EtOAc/AcOH - 175:75:1, R_f= 0.4) gave 182 mg (0.34 mmol, 74%) of a white wax; ^1H NMR (600 MHz, DMSO-d_6) δ 8.25 (s, 1H), 7.64 (d, *J* = 7.9 Hz, 2H), 7.35 (d, *J* = 8.1 Hz, 2H), 7.22 (t, *J* = 7.6 Hz, 1H), 7.10 – 7.05 (m, 3H), 6.78 (s, 1H),

5.55 (s, 2H), 5.03 (s, 2H), 3.67 – 3.60 (m, 2H), 3.36 (s, 3H), 2.88 (t, *J* = 7.6 Hz, 2H), 2.57 (t, *J* = 7.7 Hz, 2H), 2.28 (s, 3H), 0.89 – 0.83 (m, 2H), -0.07 (s, 9H);^{13}C NMR (151 MHz, DMSO-d_6) δ 173.8, 156.4, 153.0, 151.2, 141.1, 138.2, 137.6, 136.3, 129.1, 128.6 (2C) 128.4 (3C), 127.6, 127.5, 124.0, 102.1, 102.0, 70.3, 65.7, 52.7, 39.5, 37.3, 35.3, 30.2, 21.1, 17.3, -1.5 (9C); HRMS (ES+, m/z): found 531.2795 (calcd. $C_{30}H_{38}N_4O_3Si$, 531.1742 [M+H]+).

6-(4-(Difluoromethyl)phenyl)-*N*-methyl-*N*-(3-methylbenzyl)-7-((2-(trimethylsilyl)ethoxy)methyl)-7*H*-pyrrolo[2,3-*d*]pyrimidin-4-amine (116)

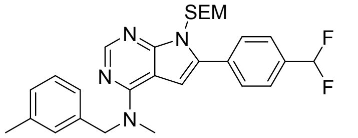

The material was made as described in General Procedure B, starting with **62** (211 mg, 0.42 mmol) and (4-(difluoromethyl)phenyl)boronic acid. The reaction time was 15 min. Silica-gel column chromatography purification (*n*-pentane/EtOAc – 2:1, R_f = 0.7) gave 189 mg (0.37 mmol, 88%) of a beige oil; ^{1}H NMR (600 MHz, DMSO-d_6) δ 8.26 (s, 1H), 7.84 – 7.91 (m, 2H), 7.65– 7.69 (m, 2H), 7.18 – 7.23 (m, 1H), 6.99 – 7.10 (m, 3H), 6.93 (s, 1H), 5.58 (s, 2H), 5.03 (s, 2H), 3.59 – 3.64 (m, 2H), 3.36 (s, 3H), 2.26 (s, 3H), 0.81 – 0.88 (m, 2H), -0.09 (s, 9H); ^{13}C NMR (151 MHz, DMSO-d_6) δ 156.6, 153.3, 151.6, 138.1, 137.6, 135.2, 133.9, 133.5 (t, J_{CF} = 23 Hz), 128.7 (2C), 128.4, 127.6, 127.5, 126.0 (t, J_{CF} = 6 Hz, 2C), 124.0, 114.7 (t, J_{CF} = 237 Hz), 103.4, 102.1, 70.3, 65.7, 52.7,37.4, 21.0, 17.3, -1.5 (3C); HRMS (ES+, m/z): found 509.2549 (calcd. $C_{28}H_{35}F_2N_4OSi$, 509.2469 $[M+H]^+$).

4-(4-(Methyl(3-methylbenzyl)amino)-7-((2-(trimethylsilyl)ethoxy)methyl)-7*H*-pyrrolo[2,3-*d*]pyrimidin-6-yl)benzenesulfonamide (117)

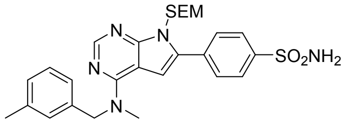

The material was made as described in General Procedure B, but starting with **62** (207 mg, 0.41 mmol) and (4-sulfamoylphenyl)boronic acid. The reaction time was 15 min. Silica-gel column chromatography purification (*n*-pentane/EtOAc – 1:1, R_f= 0.2) gave 140 mg (0.26 mmol, 64%) of a clear oil; ^{1}H NMR (600 MHz, DMSO-d_6) δ 8.27 (s, 1H), 7.93 (d, *J* = 8.4 Hz, 2H), 7.89 (d, *J* = 8.5 Hz, 2H), 7.43 (s, 2H), 7.21 (t, *J* = 7.6 Hz, 1H), 7.11 – 7.03 (m, 3H), 7.00 (s, 1H), 5.60 (s, 2H), 5.04 (s, 2H), 3.65 (dd, *J* = 8.5, 7.5 Hz, 2H), 3.38 (s, 3H), 2.27 (s, 3H), 0.87 (dd, *J* =

8.6, 7.4 Hz, 2H), -0.07 (s, 9H);^{13}C NMR (151 MHz, DMSO-d_6) δ 156.6, 153.5, 151.8, 143.2, 138.1, 137.6, 134.8, 134.7, 128.5 (2C), 127.6, 127.5, 126.0 (2C) 124.1, 104.0, 102.1, 70.4, 65.7, 59.7, 52.7, 37.5, 21.1, 17.3, -1.4(3C).; HRMS (ES+, m/z): found 538.2310 (calcd. $C_{27}H_{36}N_5O_3SSi$, 538.2229 $[M+H]^+$).

Methyl 3-(4-(methyl(3-methylbenzyl)amino)-7-((2-(trimethylsilyl)ethoxy)methyl)-7H-pyrrolo[2,3-d]pyrimidin-6-yl)benzoate (118)

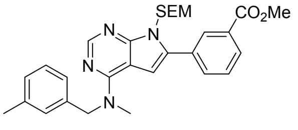

The material was made as described in General Procedure B, but starting with **62** (310 mg, 0.61 mmol) and (3-(methoxycarbonyl)phenyl)boronic acid. The reaction time was 15 min. Silica-gel column chromatography purification (*n*-pentane/EtOAc – 3:1, R_f= 0.55) gave 290 mg (0.56 mmol, 92%) of a white wax; ^{1}H NMR (400 MHz, DMSO-d_6) δ 8.30 (d, *J* = 1.8 Hz, 1H), 8.26 (s, 1H), 7.99 (dt, *J* = 7.9, 1.8 Hz, 2H), 7.63 (t, *J* = 7.8 Hz, 1H), 7.21 (t, *J* = 7.5 Hz, 1H), 7.06 (dd, *J* = 13.9, 6.3 Hz, 3H), 6.92 (s, 1H), 5.54 (s, 2H), 5.03 (s, 2H), 3.88 (s, 3H), 3.66 – 3.57 (m, 2H), 3.37 (s, 3H), 2.27 (s, 3H), 0.93 – 0.79 (m, 2H), -0.09 (s, 9H); ^{13}C NMR (101 MHz, DMSO-d_6) δ 165.9, 156.6, 153.1, 151.6, 138.2, 137.7, 135.1, 133.1, 131.9, 130.2, 129.2, 129.0, 128.6, 128.4, 127.6 (2C), 124.0, 103.2, 70.3, 65.6, 52.3, 39.5, 38.9, 37.4, 21.0, 17.2, -1.5(3C); HRMS (ES+, m/z): found 517.2632, calcd for $C_{29}H_{37}N_4O_3Si$, $[M+H]^+$, 517.2549.

(4-(4-Chloro-7-((2-(trimethylsilyl)ethoxy)methyl)-7*H*-pyrrolo[2,3-*d*]pyrimidin-6-yl)phenyl)methanol (119)

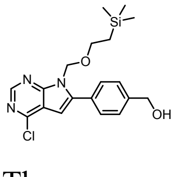

The compound was made following General Procedure B, but starting with **53** (320 mg, 0.782 mmol) and 4-hydroxymethylphenylboronic acid (119 mg, 0.782 mmol). The reaction time was 35 min. The crude product was purified by silica-gel column chromatography (*n*-pentane/EtOAc - 3:1, R_f= 0.18) giving 234 mg (0.601 mmol, 77%) of a pale-yellow solid, mp. 85-86 ˚C; ^{1}H NMR (400 MHz, $CDCl_3$) δ 8.67 (s, 1H), 7.79 (d, *J* = 8.34 Hz, 2H), 7.52 (d, *J* = 8.34 Hz, 2H), 6.71 (s, 1H), 5.62 (s, 2H), 4.80 (d, *J* = 5.85 Hz, 2H), 3.76-3.72 (m, 2H), 1.84-1.82 (m, 1H), 0.99-0.95 (m, 2H), -0.02 (s, 9H); ^{13}C NMR (100 MHz, $CDCl_3$) δ 153.5, 151.5, 150.9, 143.4, 142.2, 129.8 (2C), 129.7, 127.3 (2C), 117.6, 99.5, 71.1, 64.9, 18.0, -1.4 (3C); IR

(cm^{-1}): 3360 (m), 2952 (m), 2895 (m), 1543 (m), 1498 (s), 1351 (s) 1249 (m), 1079 (s), 860 (s), 835 (s), 775 (m), 590 (w); HRMS (ASAP+, m/z): found 390.1405, calcd for $C_{19}H_{25}N_3O_2SiCl$ $[M+H]^+$, 390.1405.

(4-(4-Amino-7-((2-(trimethylsilyl)ethoxy)methyl)-7*H*-pyrrolo[2,3-*d*]pyrimidin-6-yl)phenyl)methanol (120)

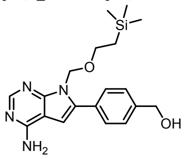

Compound **119** (103 mg, 0.265 mmol), aqueous ammonia (2 mL, 25%) and 1,4-dioxane (2 mL) were sealed in a thick-walled glass reaction vial. The reaction mixture was stirred vigorously under microwave irradiation (140 ˚C, 16 bar, 110 W) for 45 min. The volatiles were removed under reduced pressure and the residue purified by silica-gel column chromatography (CH_2Cl_2/MeOH - 92.5:7.5, R_f = 0.20) which gave 80 mg (0.215 mmol, 81%) of a colorless solid; ^1H NMR (400 MHz, $CDCl_3$) δ 8.37 (s, 1H), 7.77 – 7.70 (m, 2H), 7.51 – 7.44 (m, 2H), 6.45 (s, 1H), 5.56 (s, 2H), 5.12 (s, 2H), 4.78 (s, 2H), 3.77 – 3.69 (m, 2H), 2.01 (s, 1H), 1.00 – 0.92 (m, 2H), -0.03 (s, 9H); ^{13}C NMR (101 MHz, $CDCl_3$) δ 156.2, 152.9, 152.2, 141.2, 139.0, 130.9, 129.4 (2C), 127.3 (2C), 103.1, 97.9, 70.7, 66.6, 65.0, 18.0, -1.4 (3C); HRMS (ES+, m/z): found 371.1905, calcd for $C_{19}H_{27}N_4O_2Si$, $[M+H]^+$, 371.1903.

***N*-(6-(4-(Hydroxymethyl)phenyl)-7-((2-(trimethylsilyl)ethoxy)methyl)-7*H*-pyrrolo[2,3-*d*]pyrimidin-4-yl)benzamide (121)**

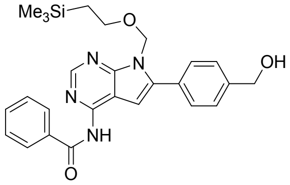

Compound **120** (61 mg, 0.165 mmol), benzoic acid (41 mg, 0.335 mmol), hexafluorophosphate azabenzotriazole tetramethyl uronium (HATU) (126 mg, 0.330 mmol) and *N*,*N*-diisopropylethylamine (56 μL, 0.327 mmol) were dissolved in DMF (0.65 mL). The reaction mixture was heated on an oil-bath (50 ˚C) while stirring for 18.5 h. The reaction mixture was concentrated in vacuo and the resulting residue partitioned between EtOAc (10 mL) and water (10 mL). The layers were separated, and the aqueous phase was extracted with EtOAc (2 × 5 mL). The combined organic phases were washed with water (2 × 5 mL) and brine (10 mL), dried over anhydrous Na_2SO_4, filtered and concentrated in vacuo. Purification by silica-gel chromatography (CH_2Cl_2/*n*-pentane/EtOAc - 100 : 50 : 65, R_f = 0.24 → 100:50:75) gave 37

mg (0.078 mmol, 47%) of a yellow oil; ^1H NMR (400 MHz, $CDCl_3$) δ 8.79 (s, 1H), 8.60 (s, 1H), 8.04 – 7.98 (m, 2H), 7.86 – 7.79 (m, 2H), 7.67 – 7.56 (m, 1H), 7.58 – 7.50 (m, 2H), 7.51 – 7.45 (m, 2H), 7.18 (s, 1H), 5.66 (s, 2H), 4.78 (s, 2H), 3.80 – 3.71 (m, 2H), 1.59 (s, 1H), 1.03 – 0.93 (m, 2H), -0.02 (s, 9H); ^{13}C NMR (101 MHz, $CDCl_3$) δ 165.2, 155.5, 150.5, 149.5, 141.5, 140.7, 133.6, 132.8, 130.6, 129.7 (2C), 129.0 (2C), 127.7 (2C), 127.2 (2C), 108.3, 103.8, 70.9, 66.8, 65.0, 18.0, -1.4 (3C); HRMS (ASAP+, m/z): found 475.2163, calcd for $C_{26}H_{31}N_4O_3Si$, $[M+H]^+$, 475.2160.

4. CSF1R binding assay non-autoinhibited form

Table S1: Average Kd based on the two titrations shown. For the curve images the amount of kinase measured by qPCR (Signal; y-axis) is plotted against the corresponding compound concentration in nM in log10 scale (x-axis). Data points marked with an "x" were not used for Kd determination.

Comp.	Structure	K_d (nM)[a)]	Curve 1	Curve 2
**2**	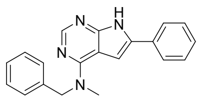	>1000	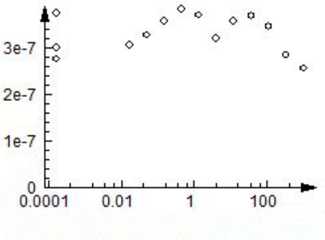	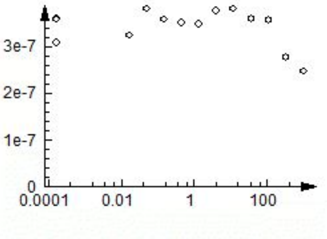
**4**	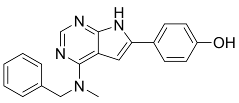	370	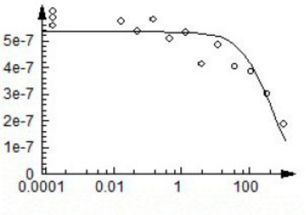	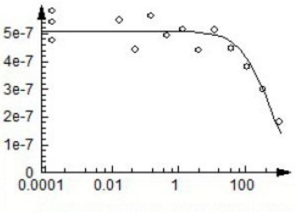
**8**	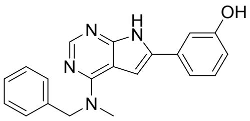	>1000	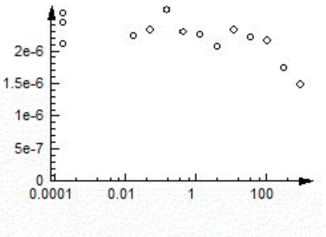	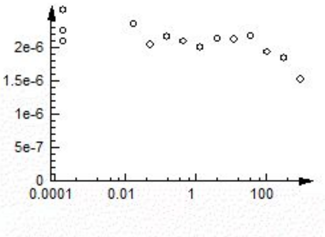
**18**	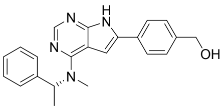	53	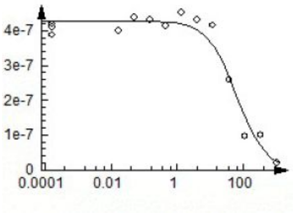	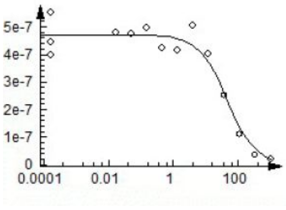
**23**	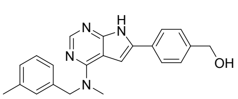	320	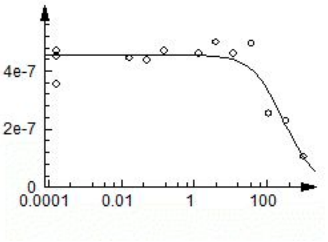	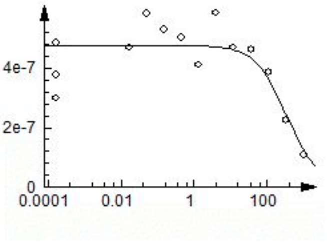
**34**	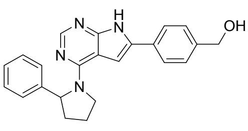	410	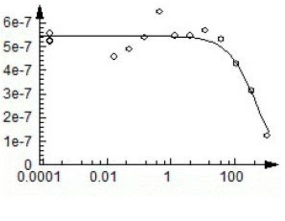	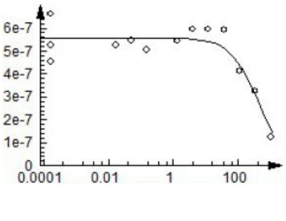

36 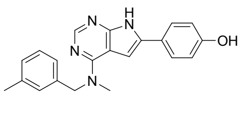 >1000 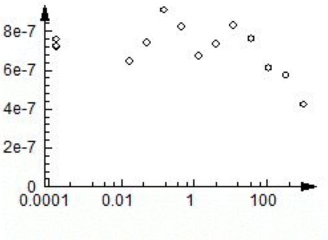 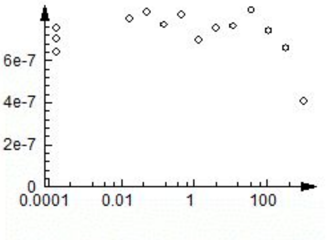

39 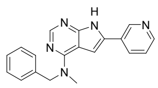 340 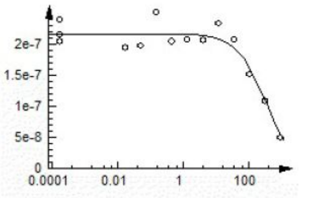 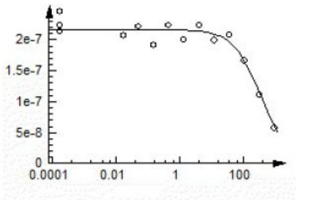

45 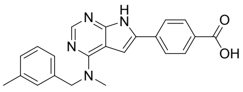 170 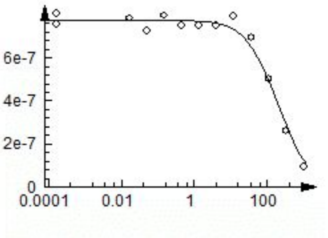 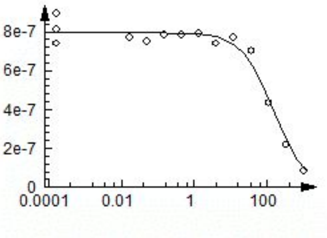

47 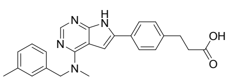 130 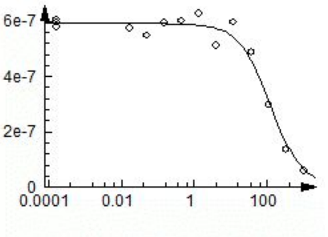 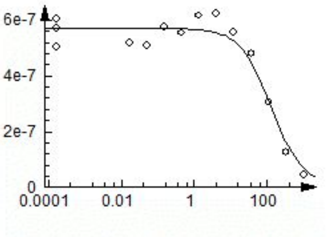

PLX-3397 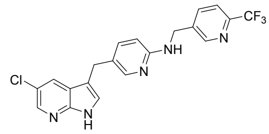 5.8 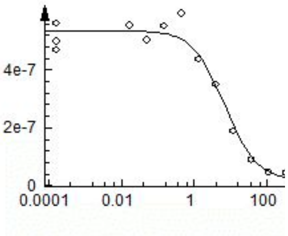 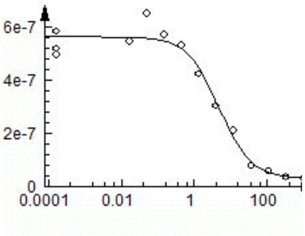

---

a)Binding assay by Eurofins, model of the non-autoinhibited form of the CSF1R kinase, containing amino acid fragment: 564-939

5. CSF1R binding assay autoinhibited form

Table S2: Average Kd based on the two titrations shown. For the curve images the amount of kinase measured by qPCR (Signal; y-axis) is plotted against the corresponding compound concentration in nM in log10 scale (x-axis). Data points marked with an "x" were not used for Kd determination.

Comp	Structure	K_d (nM)[a)]	Curve 1	Curve 2
2	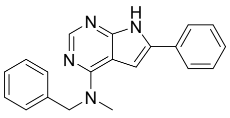	52	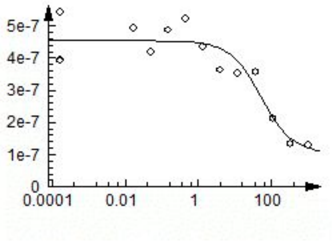	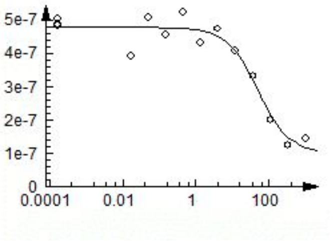
4	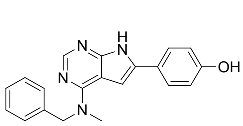	6.9	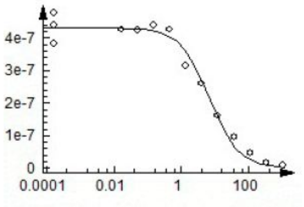	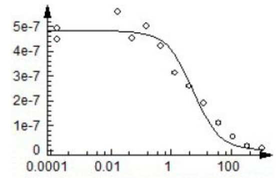
8	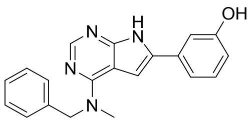	72	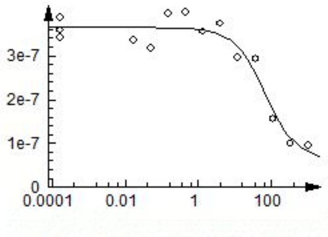	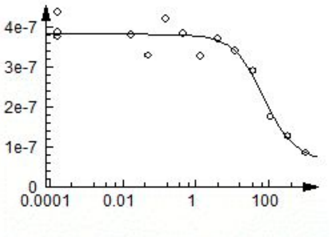
**18**	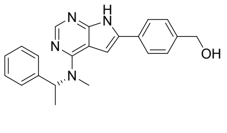	38	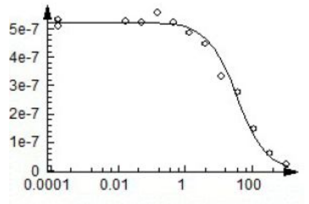	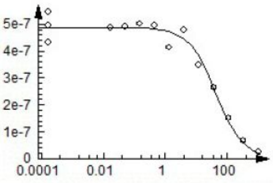
**23**	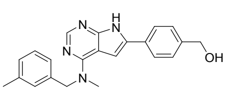	26	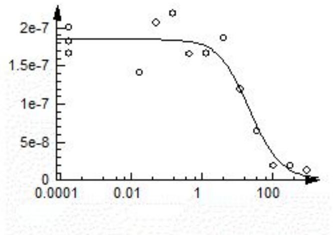	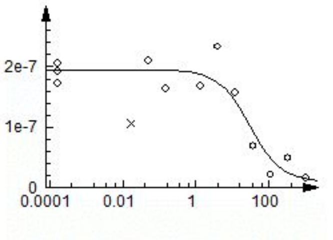
**34**	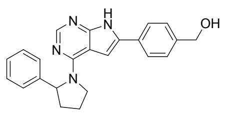	9.5	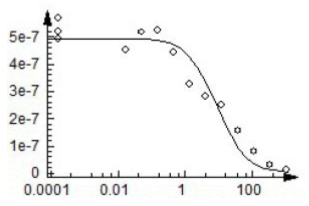	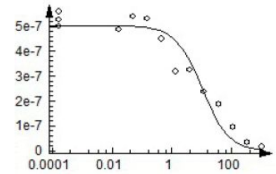

Com p	Structure	K_d (nM)[a)]	Curve 1	Curve 2
**36**	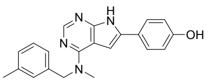	7.9	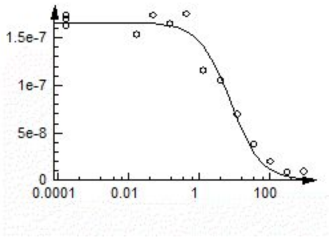	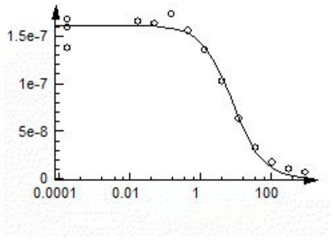
**39**	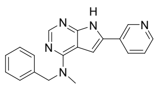	6.8	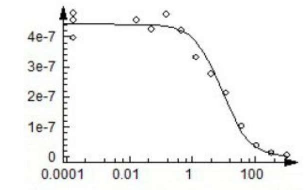	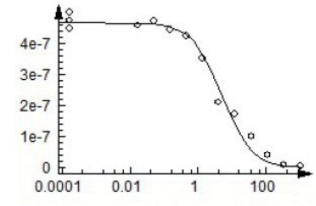
**45**	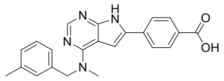	2.3	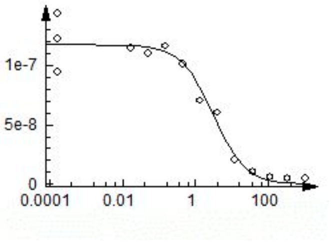	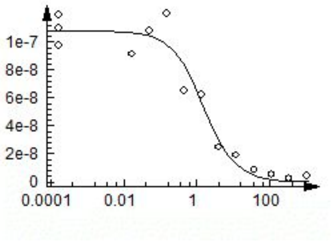
**47**	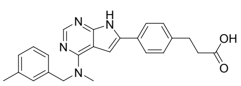	7.2	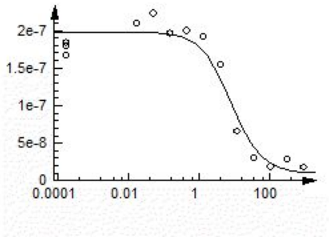	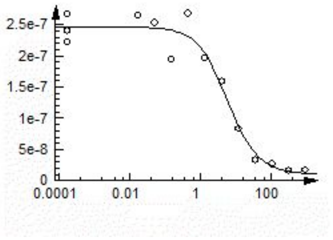
**PLX-3397**	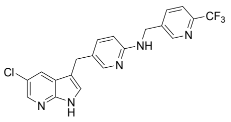	360	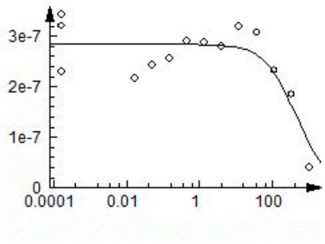	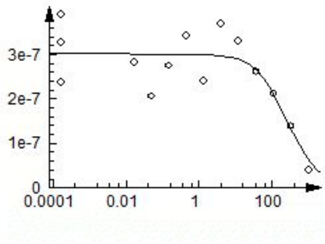

a)Binding assay by Eurofins, model of the autoinhibited form of the CSF1R kinase, containing amino acid fragment: 538-939

6. Kinase panels

Three of the inhibitors, the benzyl alcohol **23** , the benzoic acid **45** and the C-3 carboxylic acid **47** were also assayed in a panel of 468 kinases. The assay (KINOMEscan™, from DiscoverX) is an active site-directed competition binding assay to quantitatively measure interactions between test compounds and relevant human kinases. The assays do not require ATP and thereby report true thermodynamic interaction affinities. Compounds that bind to the kinase active site, and directly or indirectly prevent kinases from binding to the immobilized ligand, will reduce the amount of kinase captured on the solid support giving a low recovery of kinase. Conversely, test molecules that do not bind the kinase have no effect on the amount of kinase captured on the solid support, and a high recovery of kinase will be monitored. Table S3 shows the main off kinase targets.

Table S3: The main off-kinase targets of inhibitors **23**, **45** and **47** when assayed in a panel of 468 kinases at 500 nM test concentration. The assay measures amount of kinase not bond to the inhibitors (recovered kinase). Amount of compound bound to the kinase is calculated from recovery.

**Kinase**	**23 (rec.)**	**45 (rec.)**	**47 (rec)**	**23 (kinase bonded)**	**45 (kinase bonded)**	**47 (kinase bonded)**
CSF1R-autoinhibited	0.45	0	0.2	99.55	100	99.8
EPHB6	0.6	0.2	4.2	99.4	99.8	95.8
ABL1(H396P)-nonphosphorylated	3	5.9	3.6	97	94.1	96.4
PIK3C3/VPS34	4.3	100	75	95.7	0	25
FLT3(D835V)	11	28	3.6	89	72	96.4
ABL1(H396P)-phosphorylated	19	44	25	81	56	75
ABL1(M351T)-phosphorylated	19	46	35	81	54	65
ABL1(E255K)-phosphorylated	20	50	25	80	50	75
ABL1(Q252H)-nonphosphorylated	22	35	25	78	65	75
ABL1-phosphorylated	22	49	29	78	51	71
ABL1(Q252H)-phosphorylated	25	60	40	75	40	60
ABL1(Y253F)-phosphorylated	25	57	32	75	43	68
CSF1R	25	31	11	75	69	89
ABL1-nonphosphorylated	28	38	27	72	62	73
KIT-autoinhibited	29	46	12	71	54	88
EGFR(L858R)	31	62	35	69	38	65
KIT(V559D)	34	77	9.3	66	23	90.7
ROS1	34	35	42	66	65	58
MINK	38	83	42	62	17	58
PDGFRB	38	35	16	62	65	84
TNIK	38	46	25	62	54	75
SRC	39	43	34	61	57	66
EGFR(L747-S752del, P753S)	40	57	40	60	43	60
KIT(L576P)	40	37	15	60	63	85
EGFR	41	62	38	59	38	62
EGFR(L747-E749del, A750P)	42	58	54	58	42	46
ABL1(F317L)-phosphorylated	43	90	64	57	10	36
EGFR(E746-A750del)	43	46	51	57	54	49
EGFR(G719C)	44	70	43	56	30	57
EGFR(G719S)	45	65	33	55	35	67

7. X-ray co-crystal structure

Crystallization

The construct used for crystallization is that of a published structure (pdb entry 4HW7) bearing the mutation S688A and the deletion of residues 696 to 741. Crystals of CSF1R1 in complex with inhibitor **23** were obtained using sitting drop vapour diffusion set-ups. CSF1R1 at a concentration of 10.5 mg/ml (20 mM Hepes / NaOH, 150 mM NaCl, 10 mM DTT, pH 7.0) was pre-incubated with 1.5 mM (5.5-fold molar excess) of **23** (150 mM in DMSO) and 0.1 % V8 for 1 h. 5 mM Benzamidine were added after the partial digest. The protein solution (0.14 □L) was then mixed with 0.28 □L of reservoir solution (0.30 M DL-Malic acid pH 7.00, 23.00 %(w/v) PEG 3350) and equilibrated at 20 °C over 0.06 mLof reservoir solution. Well diffracting crystals appeared within 4 days and grew to full size over 4 days (Figure S1).

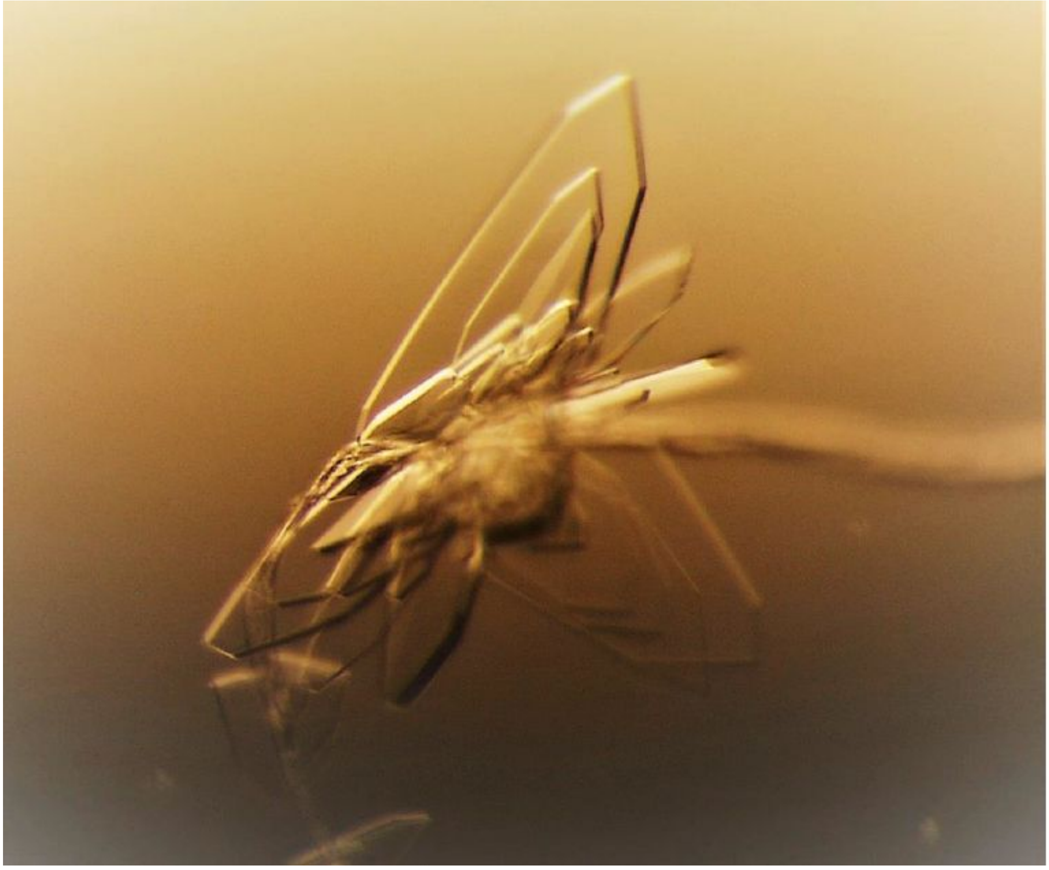

Figure S1: Co-crystals of CSF1R and inhibitor **23**.

Data Collection

A complete 1.9 Å data set of a CSF1R/**23** crystal was collected at the ESRF synchrotron radiation source (Grenoble, FR, beamline ID29) (Table S4).

Table S4: Data collection statistics

**Crystal Identifier**	xcnj18-ntn01-03-id29
**Space group**	P3221
**Unit cell parameters [Å]**	a=81.1, b=81.1, c=143.9<br>α=90.0, β=90.0, γ=120.0
**Resolution [Å]**	26.63-1.89 (1.99-1.89)
**# Unique reflections**	27202 (4022)
**I/σ(I)**	9.3 (2.2)
**Completeness [%]**	96.2 (97.0)
**Multiplicity**	3.1 (2.8)
**Rmeas**	0.080 (0.560)

Structure determination and refinement

Molecular replacement was done using a published structure of CSF1R (pdb accession code 2I1M) as starting model. Several rounds of alternating manual re-building and refinement with REFMAC5 resulted in the final model (Table S5).

Table S5: Refinement statistics

**Crystal Identifier**	xcnj18-ntn01-03-id29
**Resolution [Å]**	26.63-1.89 (1.94-1.89)
**Rwork**	0.139 (0.266)
**Rfree**	0.208 (0.314)
**Completeness [%]**	96.2 (96.9)
**r.m.s.d. bonds [Å]**	0.013
**r.m.s.d. angles [°]**	1.533

Ramachandran Plot
The model has excellent stereochemistry with no outlier in a Ramachandran plot (Figure S2).

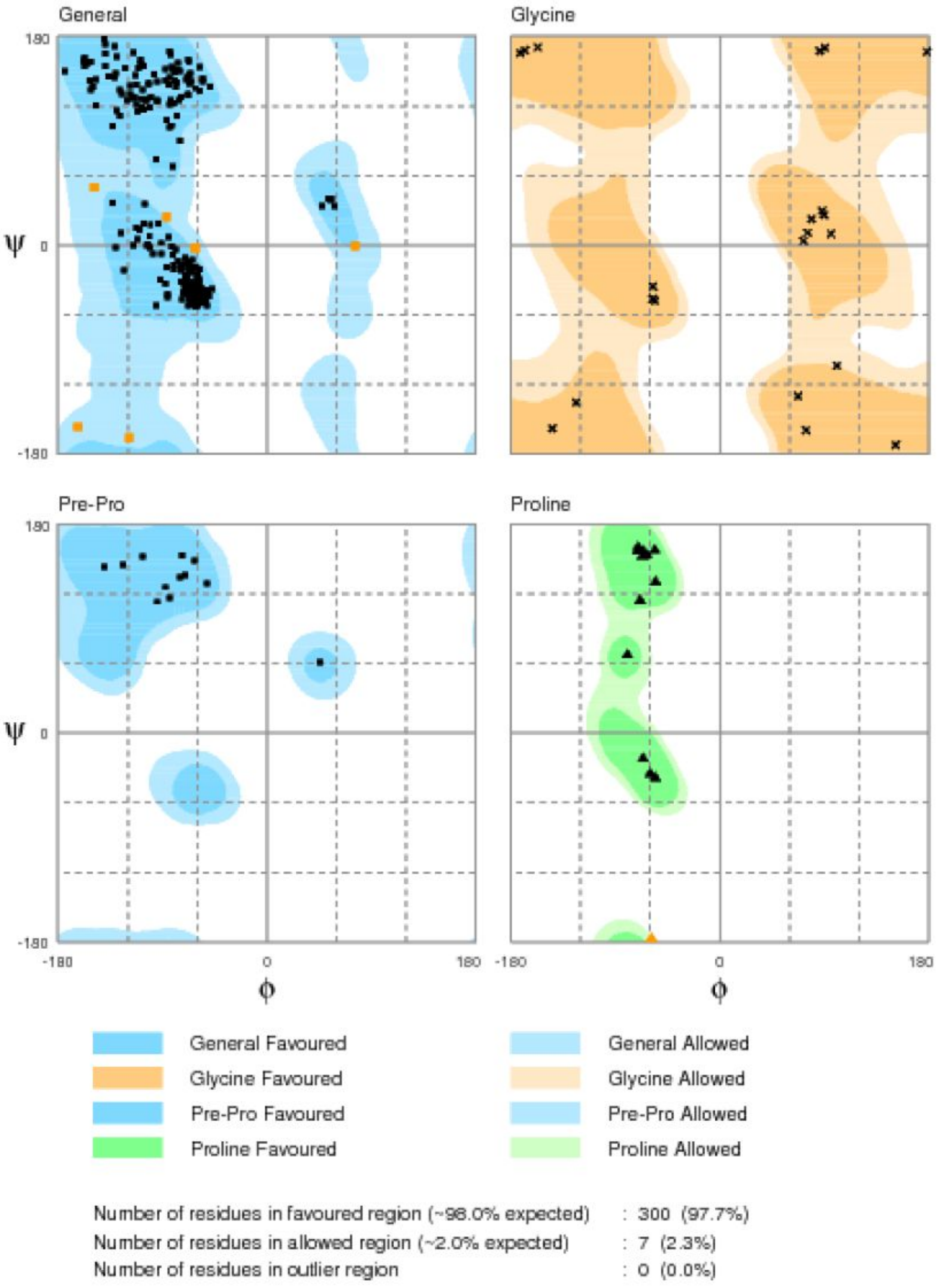

Figure **S2**: Ramachandran Plot

The overall structure (1.9 Å resolution) is in accordance with the published CSF1R structure in complex with inhibitor (pdb entry 4HW7) with an r.m.s.d. of 0.98 Å and considering 311 Cα atoms. Residues 685 to 695 and 742 to 747 around the deletion del696-741 could not be modelled due to flexibility. Clear electron density in the FoFc omit map of the initial model at

the compound binding site revealed the binding of the entire compound and allowed an unambiguous placement of the ligand (Figure S3).

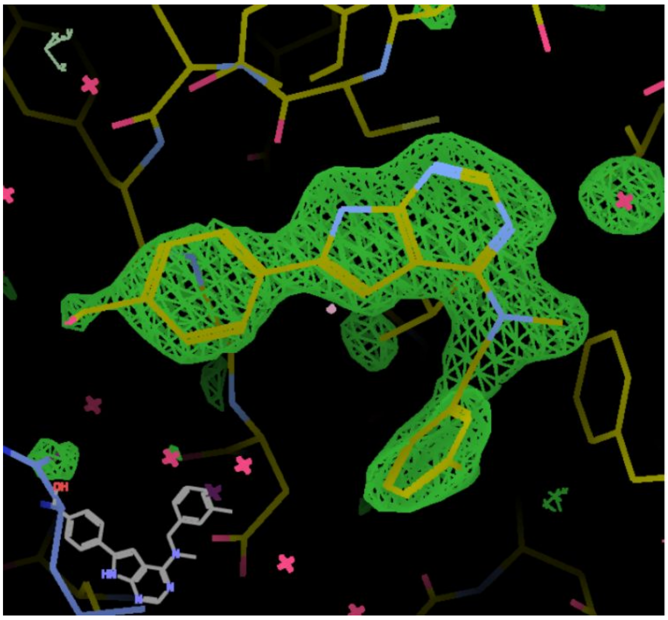

Figure S3: The initial F_oF_c omit map of the model at the compound binding site after refinement with REFMAC5 at a contour level of 3.1 σ.

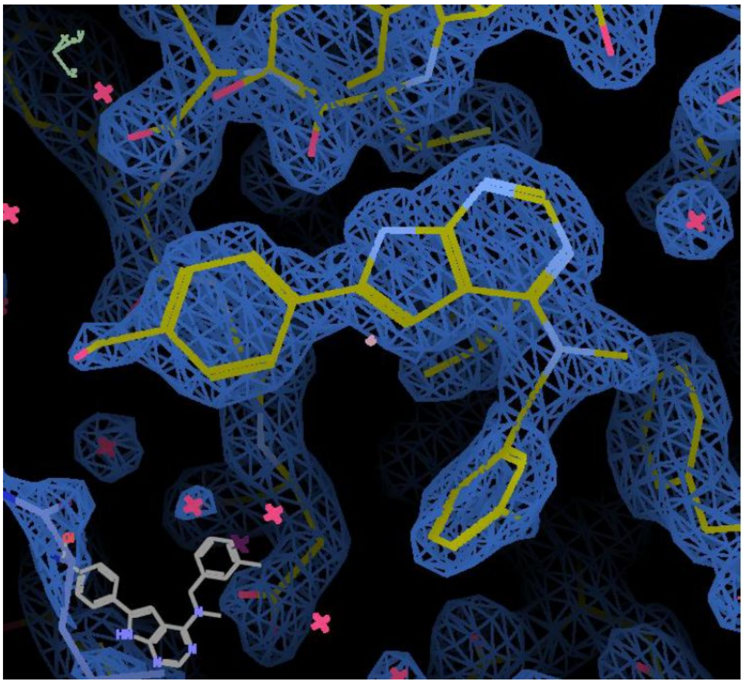

Figure S4: The final $2F_oF_c$ map of the model at the compound binding site after full refinement with REFMAC5 at a contour level of 1.7 σ.

8. HPLC trace key compounds

Compound 4

```
=====================================================================
Acq. Operator   : Frithjof                         Seq. Line :   2
Acq. Instrument : HPLC                              Location : Vial 2
Injection Date  : 1/30/2023 11:20:25 AM                  Inj :   1
                                                  Inj Volume : 5.000 µl
Acq. Method     : C:\CHEM32\1\DATA\FRITHJOF\FRITHJOF FABCSF1RARTIKKELRENHETSANALYSER 2023-01-
                  30 10-17-45\RENHETSANALYSE RASK.M
Last changed    : 1/30/2023 10:17:45 AM by Frithjof
Analysis Method : C:\CHEM32\1\METHODS\FELLES\FLUSH_WATER.M
Last changed    : 1/31/2023 1:17:15 PM by Frithjof
                  (modified after loading)
Method Info     : VASK MED rent vann
```

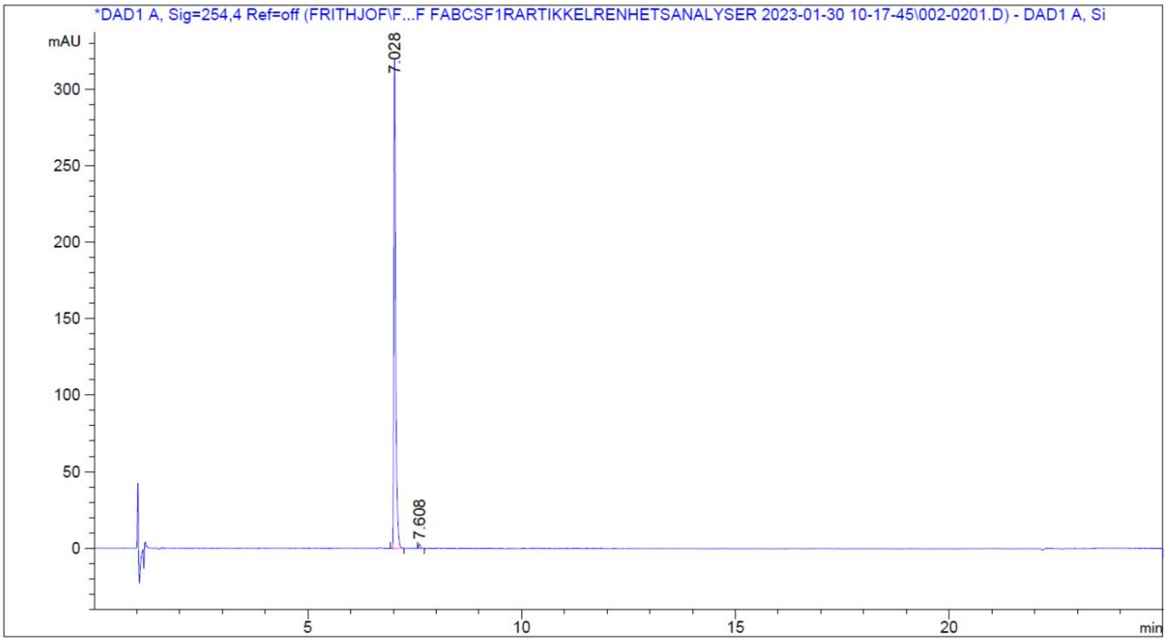


```
=====================================================================
                         Area Percent Report
=====================================================================

Sorted By             :      Signal
Multiplier:                   :      1.0000
Dilution:                     :      1.0000
Use Multiplier & Dilution Factor with ISTDs


Signal 1: DAD1 A, Sig=254,4 Ref=off
 Signal has been modified after loading from rawdata file!
```

Peak #	RetTime [min]	Type	Width [min]	Area [mAU*s]	Height [mAU]	Area %
1	7.028	BB	0.0453	967.88049	320.29950	99.1706
2	7.608	BB	0.0427	8.09433	2.89131	0.8294

Totals : 975.97482 323.19081

```
=====================================================================
                         *** End of Report ***
```

Figure S5. HPLC analysis of compound **8.**

Compound 10

```
======================================================================
Acq. Operator   : Frithjof                        Seq. Line :   4
Acq. Instrument : HPLC                             Location : Vial 3
Injection Date  : 1/30/2023 12:22:04 PM                  Inj :   1
                                                Inj Volume : 5.000 µl
Acq. Method     : C:\CHEM32\1\DATA\FRITHJOF\FRITHJOF FABCSF1RARTIKKELRENHETSANALYSER 2023-01-
                  30 10-17-45\RENHETSANALYSE RASK.M
Last changed    : 1/30/2023 10:17:45 AM by Frithjof
Analysis Method : C:\CHEM32\1\METHODS\FELLES\FLUSH_WATER.M
Last changed    : 1/31/2023 1:17:15 PM by Frithjof
                  (modified after loading)
Method Info     : VASK MED rent vann

Additional Info : Peak(s) manually integrated
```

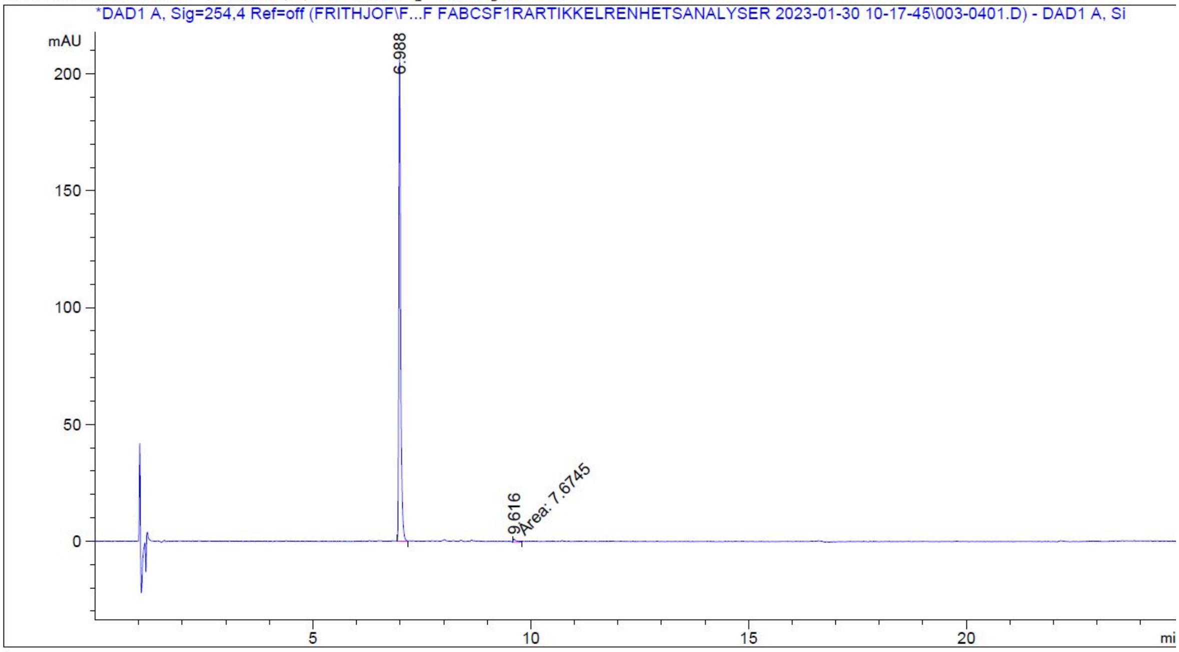


```
======================================================================
                         Area Percent Report
======================================================================

Sorted By             :      Signal
Multiplier:                  :      1.0000
Dilution:                    :      1.0000
Use Multiplier & Dilution Factor with ISTDs


Signal 1: DAD1 A, Sig=254,4 Ref=off
 Signal has been modified after loading from rawdata file!

Peak RetTime Type  Width     Area      Height     Area
  #   [min]        [min]   [mAU*s]     [mAU]       %
----|-------|----|-------|----------|----------|--------|
   1   6.988 BB    0.0440  602.11865  206.56339  98.7415
   2   9.616 MM    0.0913    7.67450    1.40141   1.2585

Totals :                   609.79315  207.96480


======================================================================
                          *** End of Report ***
```

Figure S6. HPLC analysis of compound **10.**

Compound 23

```
========================================================================
Acq. Operator   : Frithjof                          Seq. Line :  16
Acq. Instrument : HPLC                               Location : Vial 9
Injection Date  : 1/30/2023 6:34:29 PM                    Inj :   1
                                                   Inj Volume : 5.000 µl
Acq. Method     : C:\CHEM32\1\DATA\FRITHJOF\FRITHJOF FABCSF1RARTIKKELRENHETSANALYSER 2023-01-
                  30 10-17-45\RENHETSANALYSE RASK.M
Last changed    : 1/30/2023 10:17:45 AM by Frithjof
Analysis Method : C:\CHEM32\1\METHODS\FELLES\FLUSH_WATER.M
Last changed    : 1/31/2023 1:17:15 PM by Frithjof
                  (modified after loading)
Method Info     : VASK MED rent vann

Additional Info : Peak(s) manually integrated
```

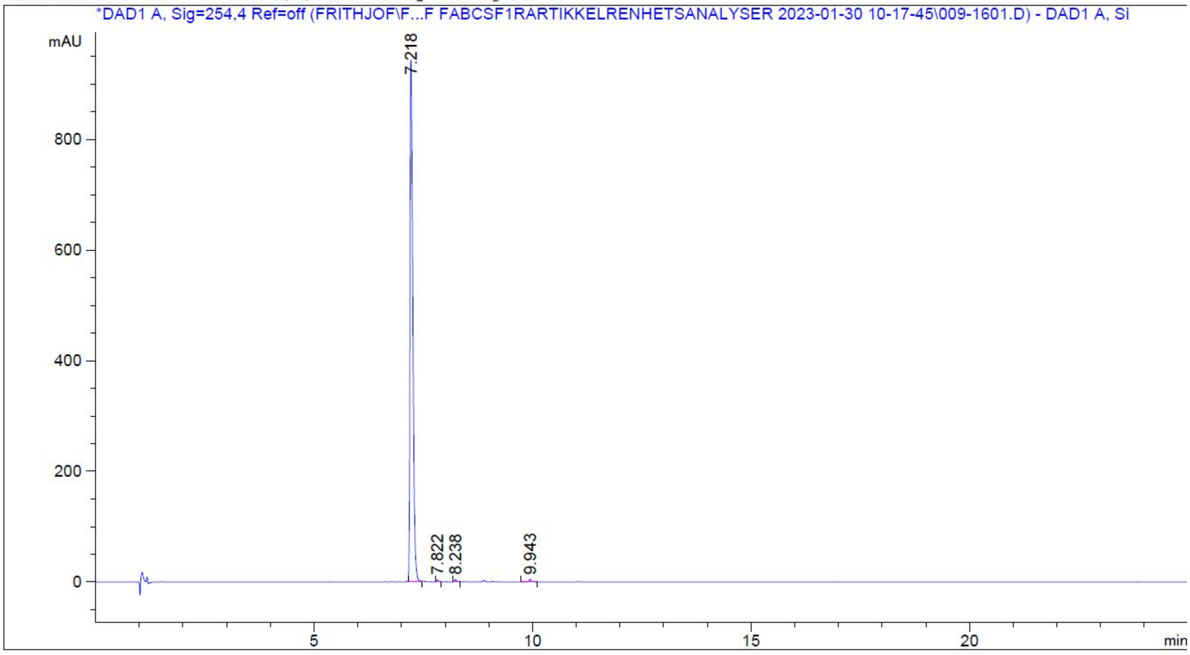


```
========================================================================
                         Area Percent Report
========================================================================

Sorted By             :      Signal
Multiplier:                    :       1.0000
Dilution:                      :       1.0000
Use Multiplier & Dilution Factor with ISTDs


Signal 1: DAD1 A, Sig=254,4 Ref=off
 Signal has been modified after loading from rawdata file!

Peak RetTime Type  Width     Area      Height     Area
  #   [min]        [min]   [mAU*s]     [mAU]        %
----|-------|----|-------|----------|----------|--------|
   1   7.218 VV    0.0715 4238.46387  941.13788 99.0235
   2   7.822 BB    0.0445   10.78479    3.76092  0.2520
   3   8.238 BB    0.0477   12.76518    4.16700  0.2982
   4   9.943 BB    0.0561   18.24645    4.94281  0.4263

Totals :                  4280.26028  954.00861


========================================================================
```

Figure S7. HPLC analysis of compound **23.**

Compound 45

```
======================================================================
Acq. Operator   : Frithjof                        Seq. Line :   4
Acq. Instrument : HPLC                             Location : Vial 3
Injection Date  : 1/4/2021 11:28:26 AM                  Inj :   1
                                                Inj Volume : 5.000 µl
Acq. Method     : C:\CHEM32\1\DATA\FRITHJOF\FRITHJOF FAB91RENHET 2021-01-04 09-54-14\
                  RENHETSANALYSE RASK.M
Last changed    : 11/6/2020 4:17:41 PM by Simen
Analysis Method : C:\CHEM32\1\METHODS\FELLES\FLUSH_WATER.M
Last changed    : 1/31/2023 1:17:15 PM by Frithjof
                  (modified after loading)
Method Info     : VASK MED rent vann

Additional Info : Peak(s) manually integrated
```

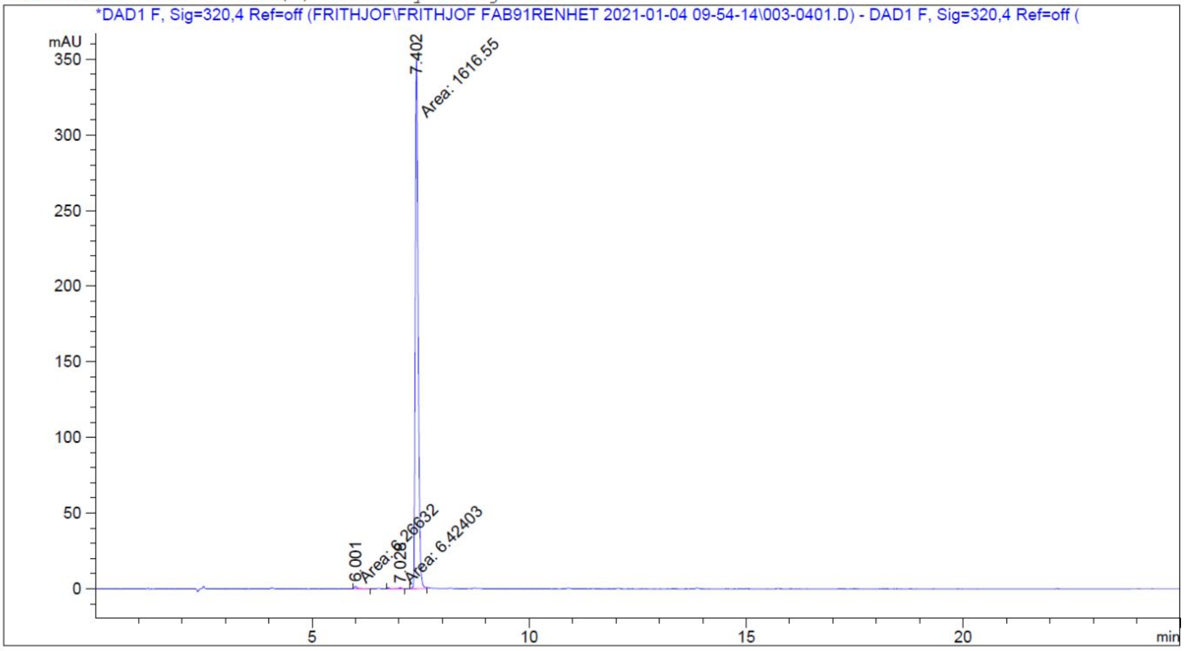


```
======================================================================
                         Area Percent Report
======================================================================

Sorted By             :      Signal
Multiplier:                   :      1.0000
Dilution:                     :      1.0000
Use Multiplier & Dilution Factor with ISTDs


Signal 1: DAD1 F, Sig=320,4 Ref=off
 Signal has been modified after loading from rawdata file!
```

Peak #	RetTime [min]	Type	Width [min]	Area [mAU*s]	Height [mAU]	Area %
1	6.001	MM	0.0682	6.26632	1.53098	0.3846
2	7.028	MM	0.1518	6.42403	7.05478e-1	0.3943
3	7.402	MF	0.0770	1616.55298	350.04135	99.2211

Totals : 1629.24333 352.27780

```
======================================================================
                          *** End of Report ***
```

Figure S8. HPLC analysis of compound **45.**

Compound 47

```
=====================================================================
Acq. Operator   : Frithjof                        Seq. Line :  30
Acq. Instrument : HPLC                             Location : Vial 14
Injection Date  : 1/31/2023 1:50:20 AM                  Inj :   1
                                                 Inj Volume : 5.000 µl
Acq. Method     : C:\CHEM32\1\DATA\FRITHJOF\FRITHJOF FABCSF1RARTIKKELRENHETSANALYSER 2023-01-
                  30 10-17-45\RENHETSANALYSE RASK.M
Last changed    : 1/30/2023 10:17:45 AM by Frithjof
Analysis Method : C:\CHEM32\1\METHODS\FELLES\FLUSH_WATER.M
Last changed    : 1/31/2023 1:17:15 PM by Frithjof
                  (modified after loading)
Method Info     : VASK MED rent vann

Additional Info : Peak(s) manually integrated
```

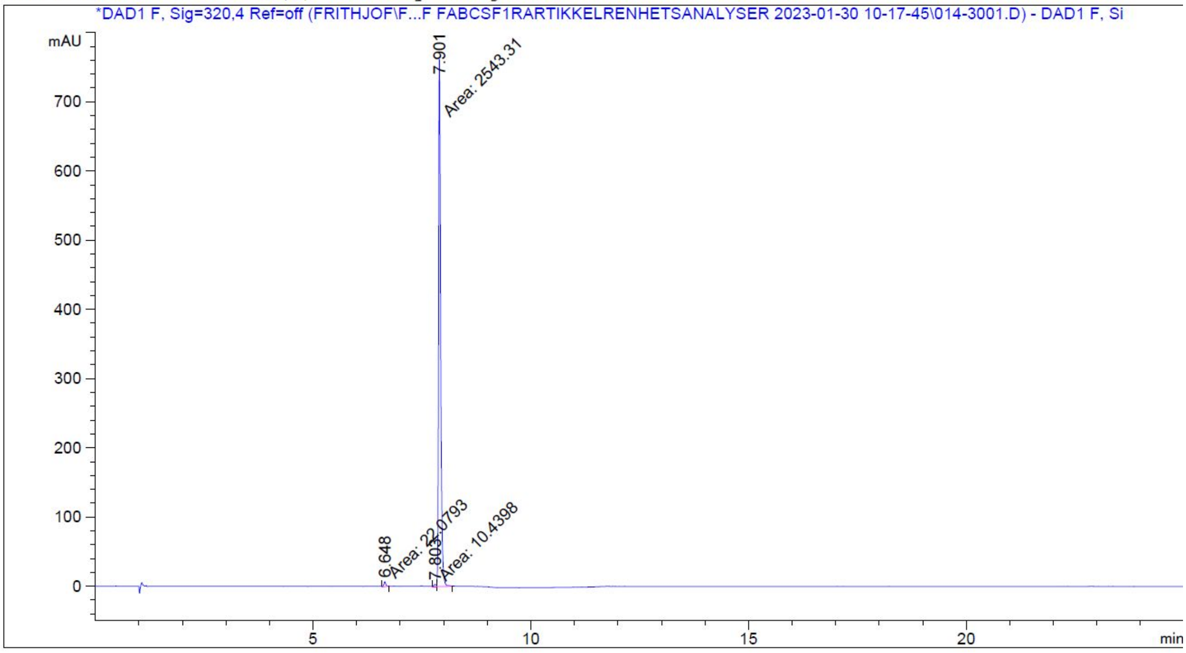


```
=====================================================================
                         Area Percent Report
=====================================================================

Sorted By             :      Signal
Multiplier:                   :      1.0000
Dilution:                     :      1.0000
Use Multiplier & Dilution Factor with ISTDs


Signal 1: DAD1 F, Sig=320,4 Ref=off
 Signal has been modified after loading from rawdata file!

Peak RetTime Type  Width     Area      Height     Area
  #   [min]        [min]   [mAU*s]     [mAU]        %
----|-------|----|-------|----------|----------|--------|
   1   6.648 MM    0.0520   22.07933    7.08087   0.8572
   2   7.803 MF    0.0569   10.43981    3.05844   0.4053
   3   7.901 FM    0.0555 2543.31079  763.41461  98.7375

Totals :                  2575.82993  773.55391


=====================================================================
                          *** End of Report ***
```

Figure S9. HPLC analysis of compound **47.**

9. NMR spectra of key compounds

Compound 1

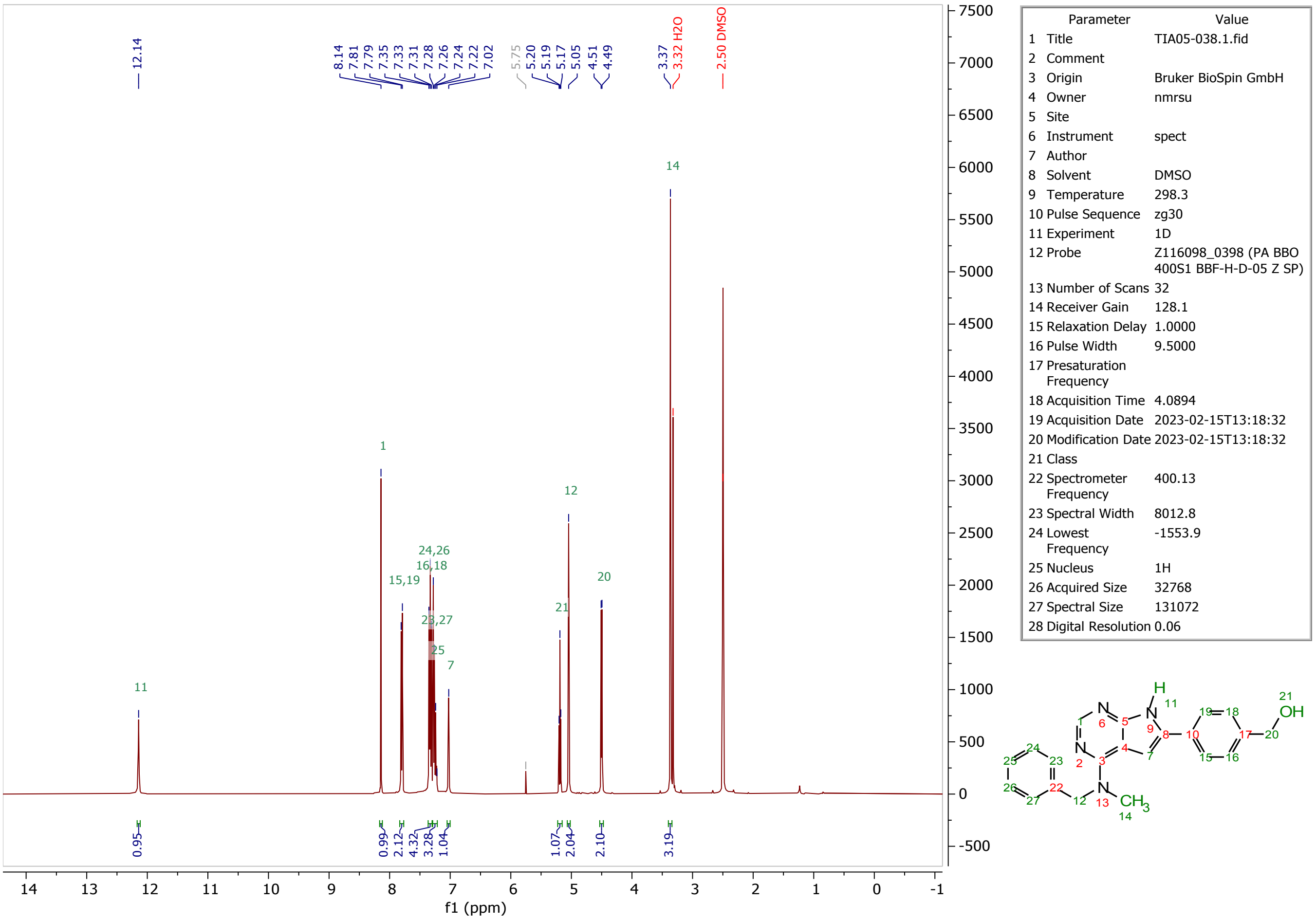

Figure S10. ^{1}H NMR (400 MHz, DMSO-d_6) of compound **1**.

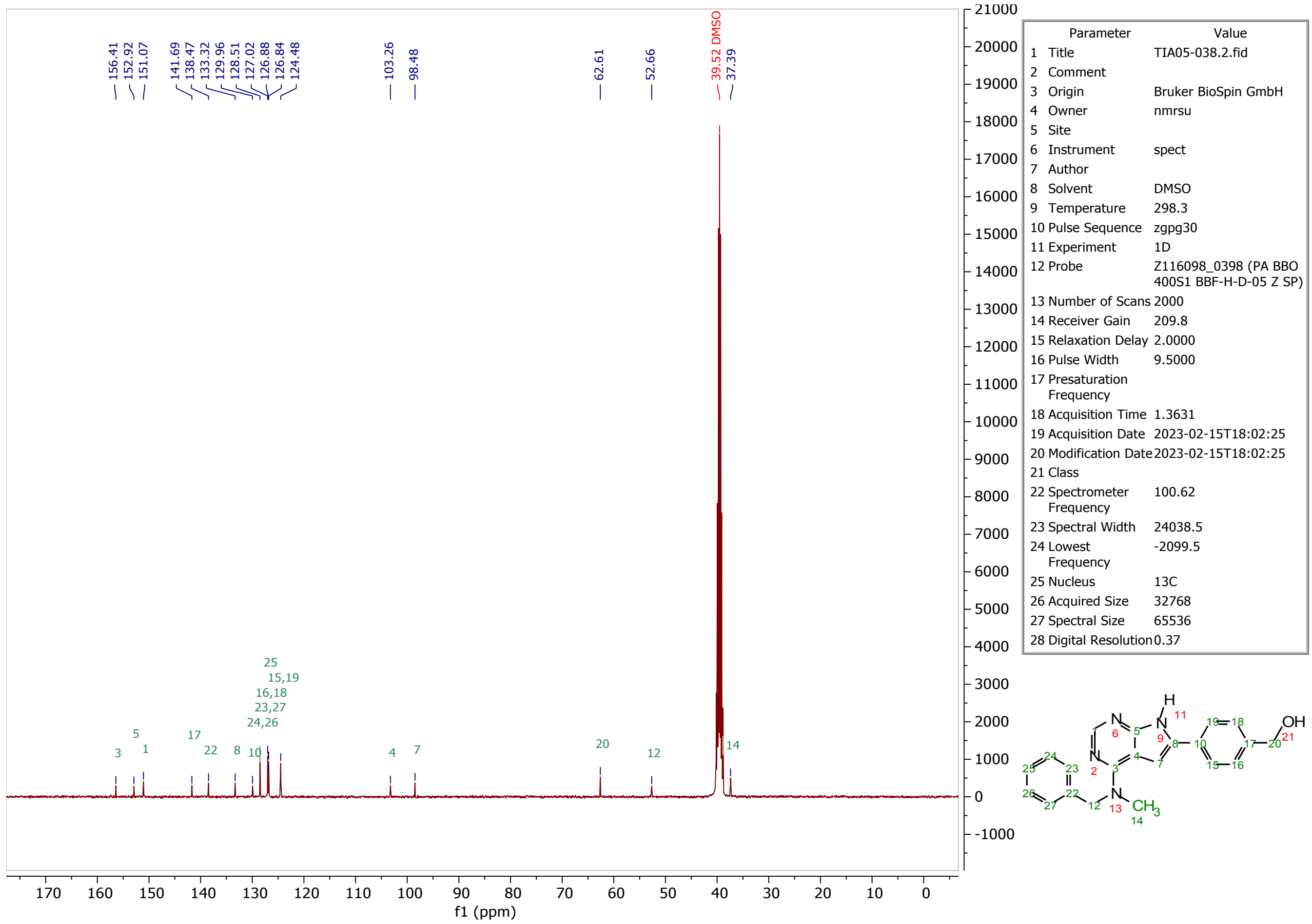

Figure S11. ^{13}C NMR (100 MHz, DMSO-d_6) of compound **1**.

Compound 2

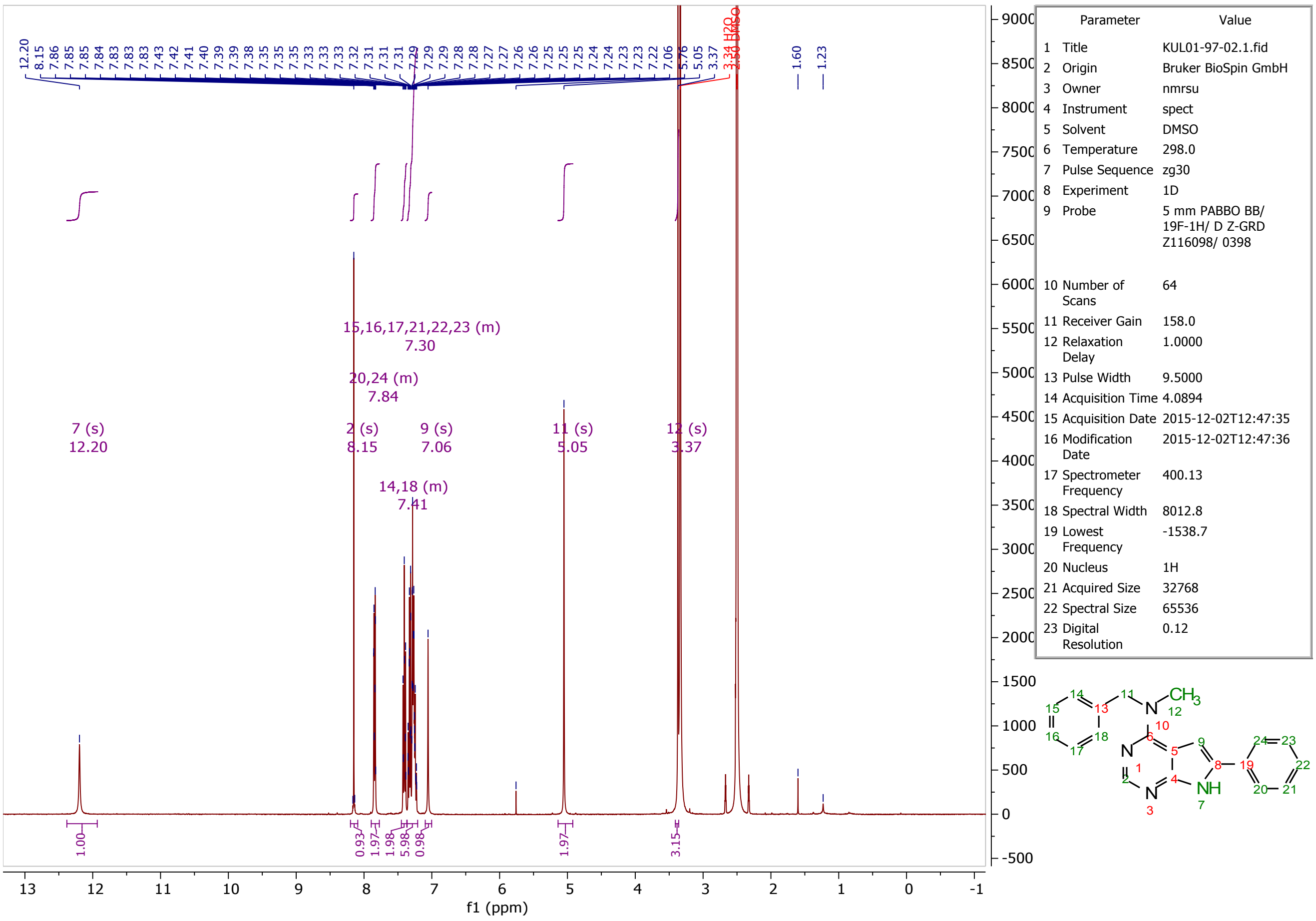

Figure S12. ^{1}H NMR (400 MHz, DMSO-d_6) of compound **2**.

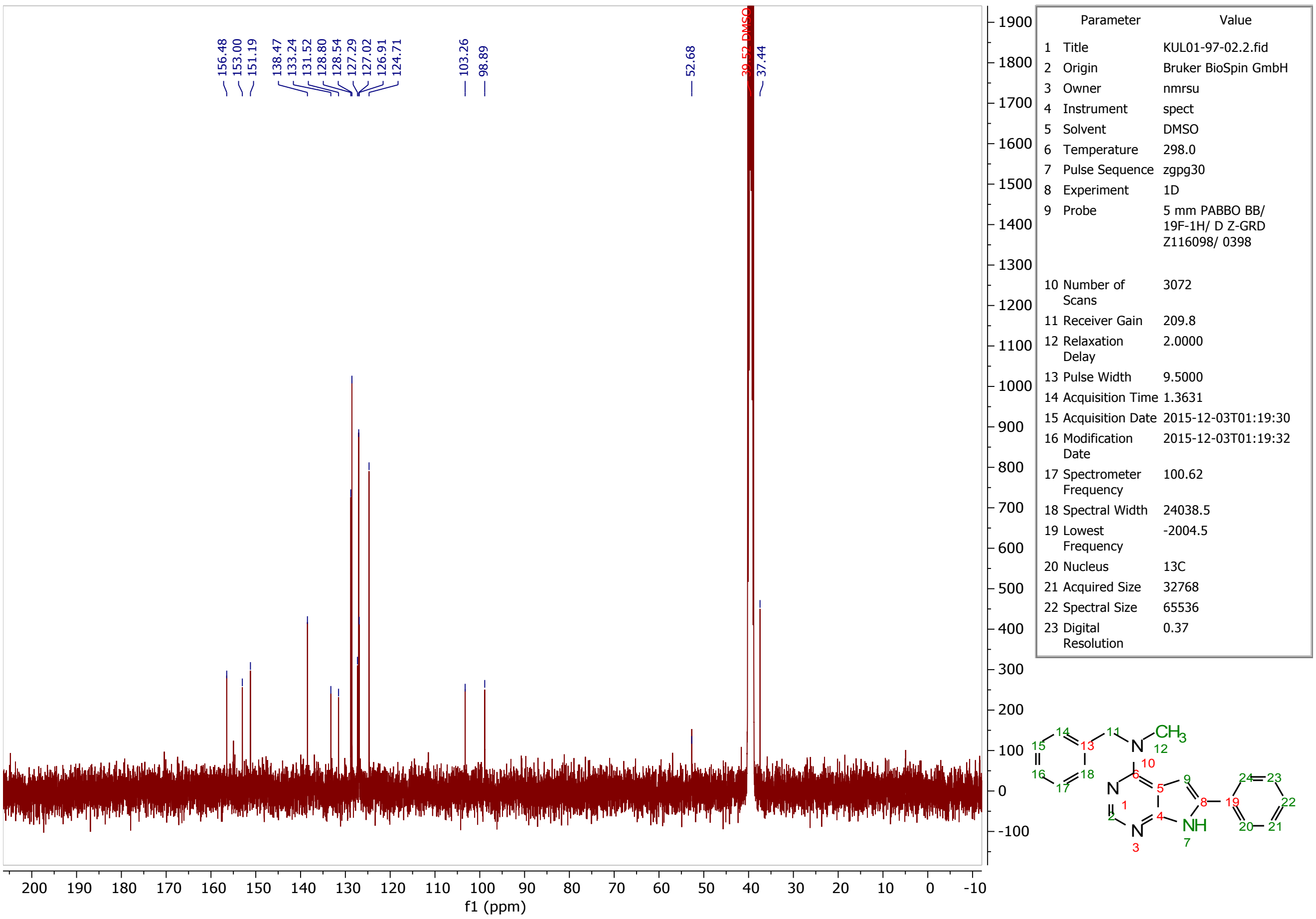

Figure S13. ^{13}C NMR (100 MHz, DMSO-d_6) of compound **2**.

Compound 3

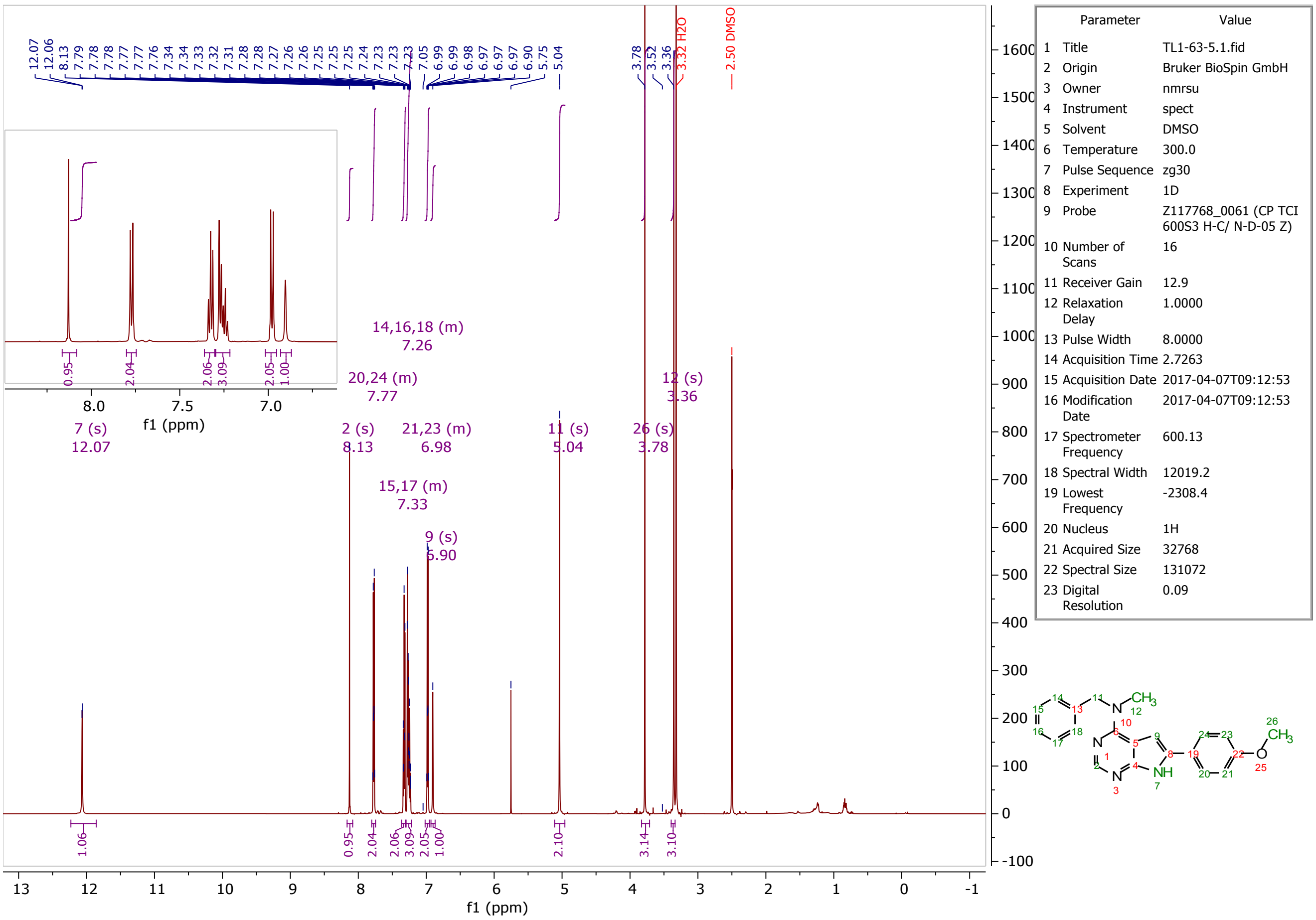

Figure S14. ^{1}H NMR (600 MHz, DMSO-d_6) of compound **3**.

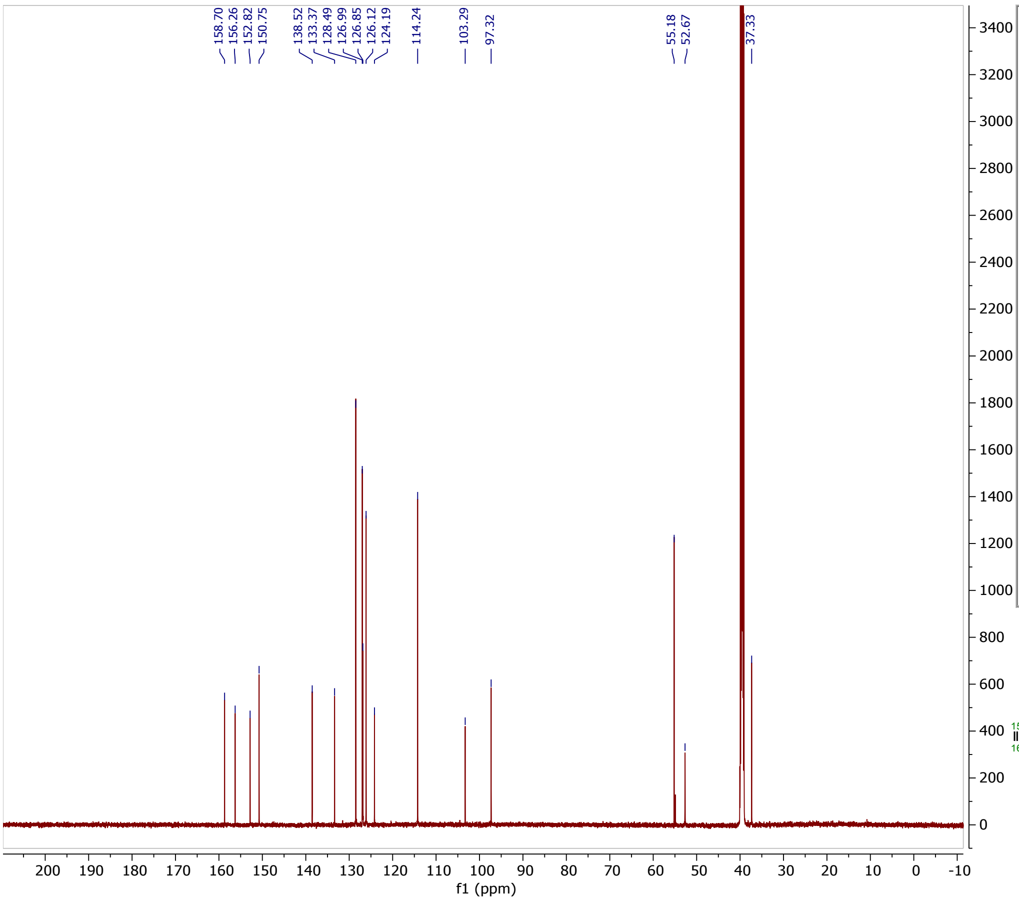

	Parameter	Value
1	Title	TL1-63-5.2.fid
2	Origin	Bruker BioSpin GmbH
3	Owner	nmrsu
4	Instrument	spect
5	Solvent	DMSO
6	Temperature	300.0
7	Pulse Sequence	zgpg30
8	Experiment	1D
9	Probe	Z117768_0061 (CP TCI 600S3 H-C/ N-D-05 Z)
10	Number of Scans	1024
11	Receiver Gain	197.1
12	Relaxation Delay	2.0000
13	Pulse Width	11.4000
14	Acquisition Time	0.9088
15	Acquisition Date	2017-04-07T10:18:05
16	Modification Date	2017-04-07T10:18:05
17	Spectrometer Frequency	150.92
18	Spectral Width	36057.7
19	Lowest Frequency	-3012.4
20	Nucleus	13C
21	Acquired Size	32768
22	Spectral Size	65536
23	Digital Resolution	0.55

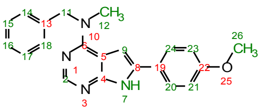

Figure S15. ^{13}C NMR (150 MHz, DMSO-d_6) of compound **3**.

Compound 4

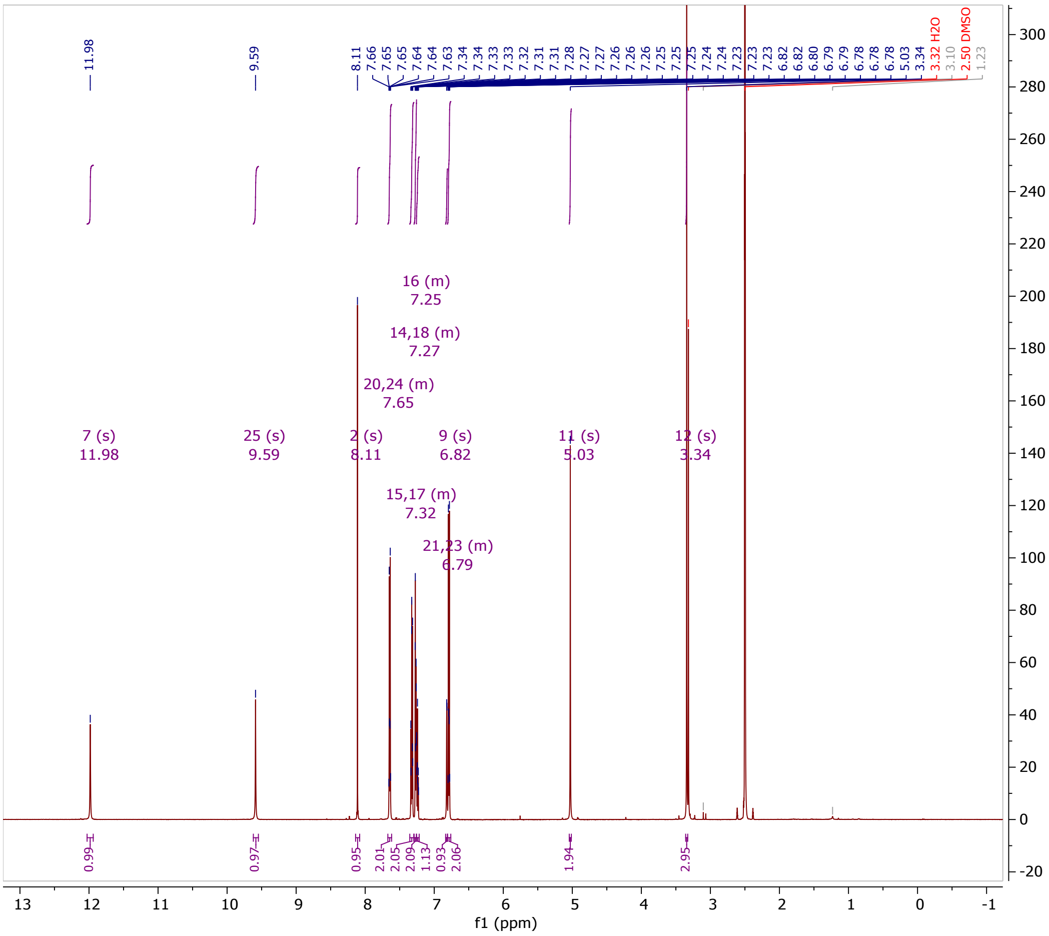

	Parameter	Value
1	Title	TIA05-172-1.1.fid
2	Origin	Bruker BioSpin GmbH
3	Owner	nmrsu
4	Instrument	spect
5	Solvent	DMSO
6	Temperature	298.0
7	Pulse Sequence	zg30
8	Experiment	1D
9	Probe	Z117768_0061 (CP TCI 600S3 H-C/ N-D-05 Z)
10	Number of Scans	16
11	Receiver Gain	9.2
12	Relaxation Delay	2.0000
13	Pulse Width	8.0000
14	Acquisition Time	2.7263
15	Acquisition Date	2020-09-07T13:36:56
16	Modification Date	2020-09-07T13:36:56
17	Spectrometer Frequency	600.18
18	Spectral Width	12019.2
19	Lowest Frequency	-2308.3
20	Nucleus	1H
21	Acquired Size	32768
22	Spectral Size	131072
23	Digital Resolution	0.09

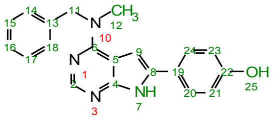

Figure S16. ^{1}H NMR (600 MHz, DMSO-d_6) of compound **4**.

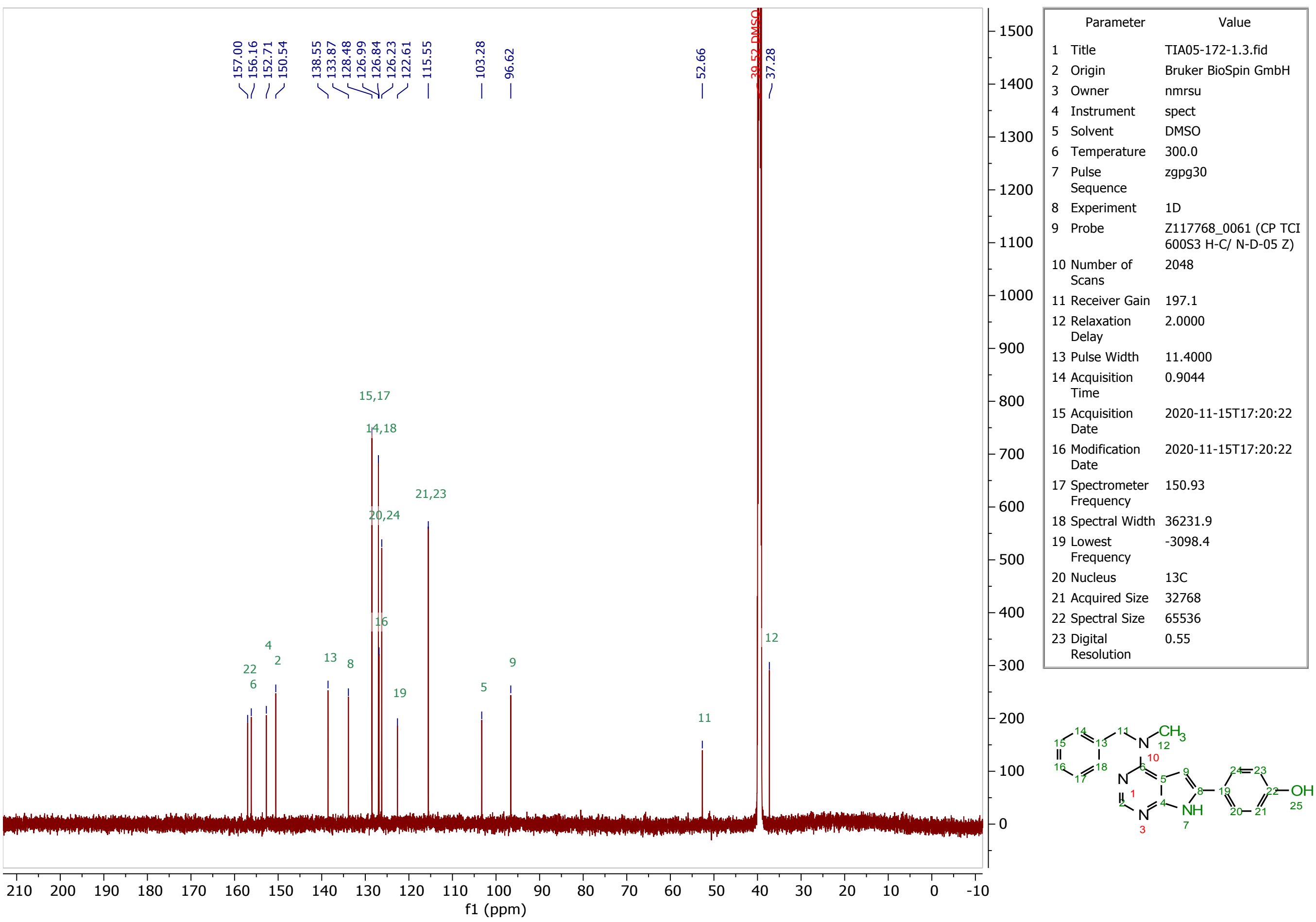

Figure S17. ^{13}C NMR (150 MHz, DMSO-d_6) of compound **4**.

Compound 5

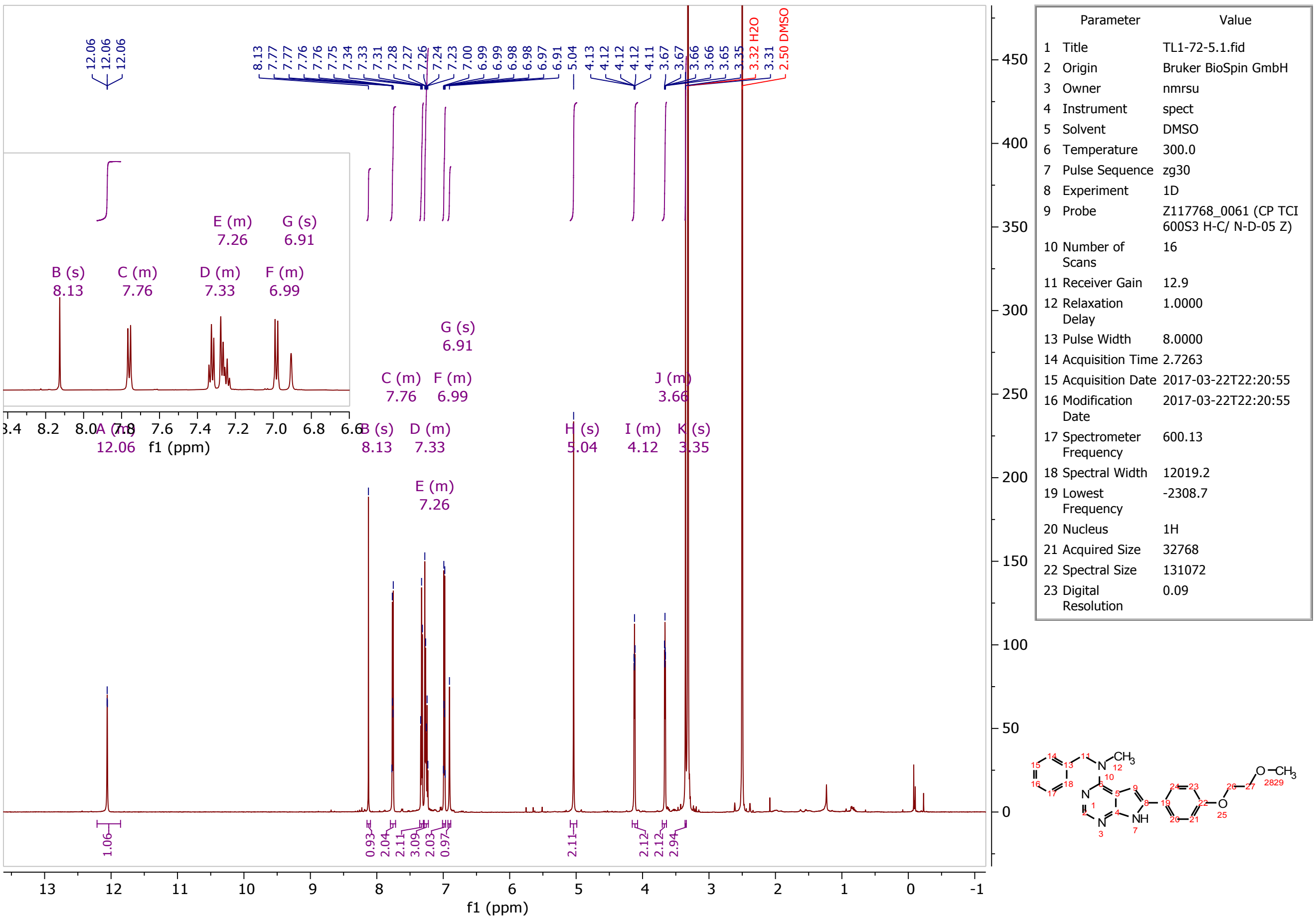

Figure S18. ^{1}H NMR (600 MHz, DMSO-d_6) of compound **5**.

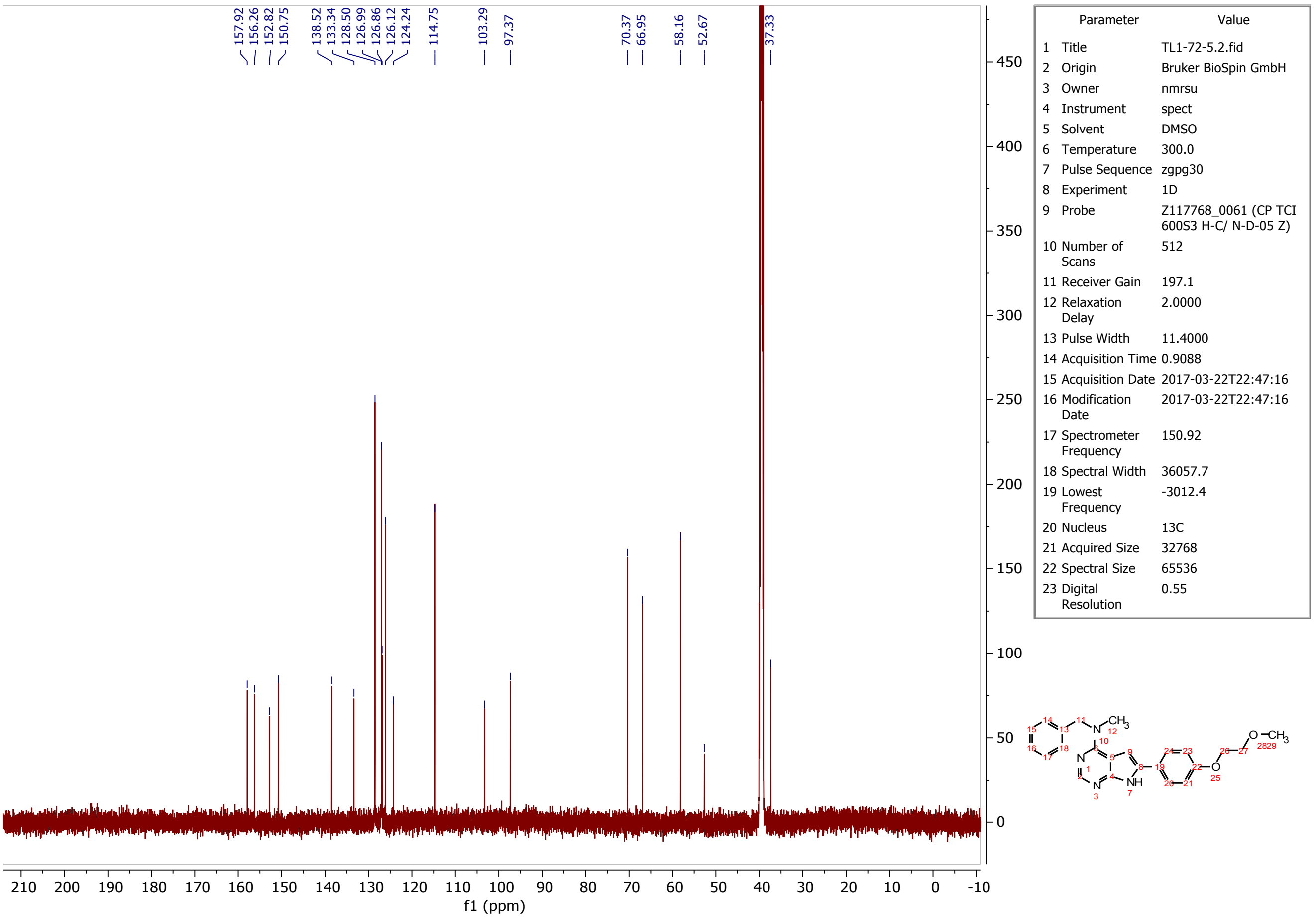

Figure S19. ^{13}C NMR (150 MHz, DMSO-d_6) of compound **5**.

Compound 6

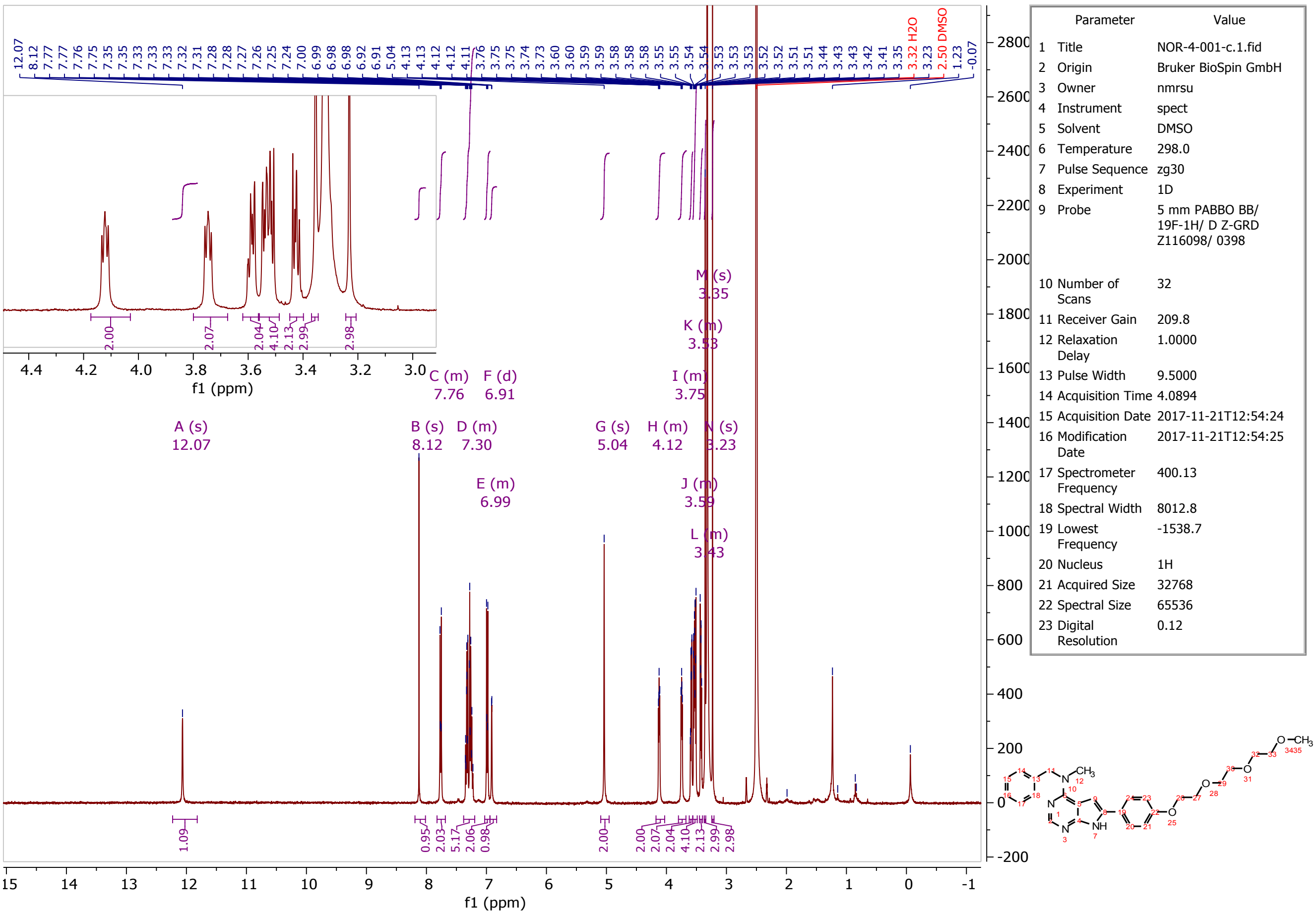

Figure S20. ^{1}H NMR (400 MHz, DMSO-d_6) of compound **6**.

Compound 7

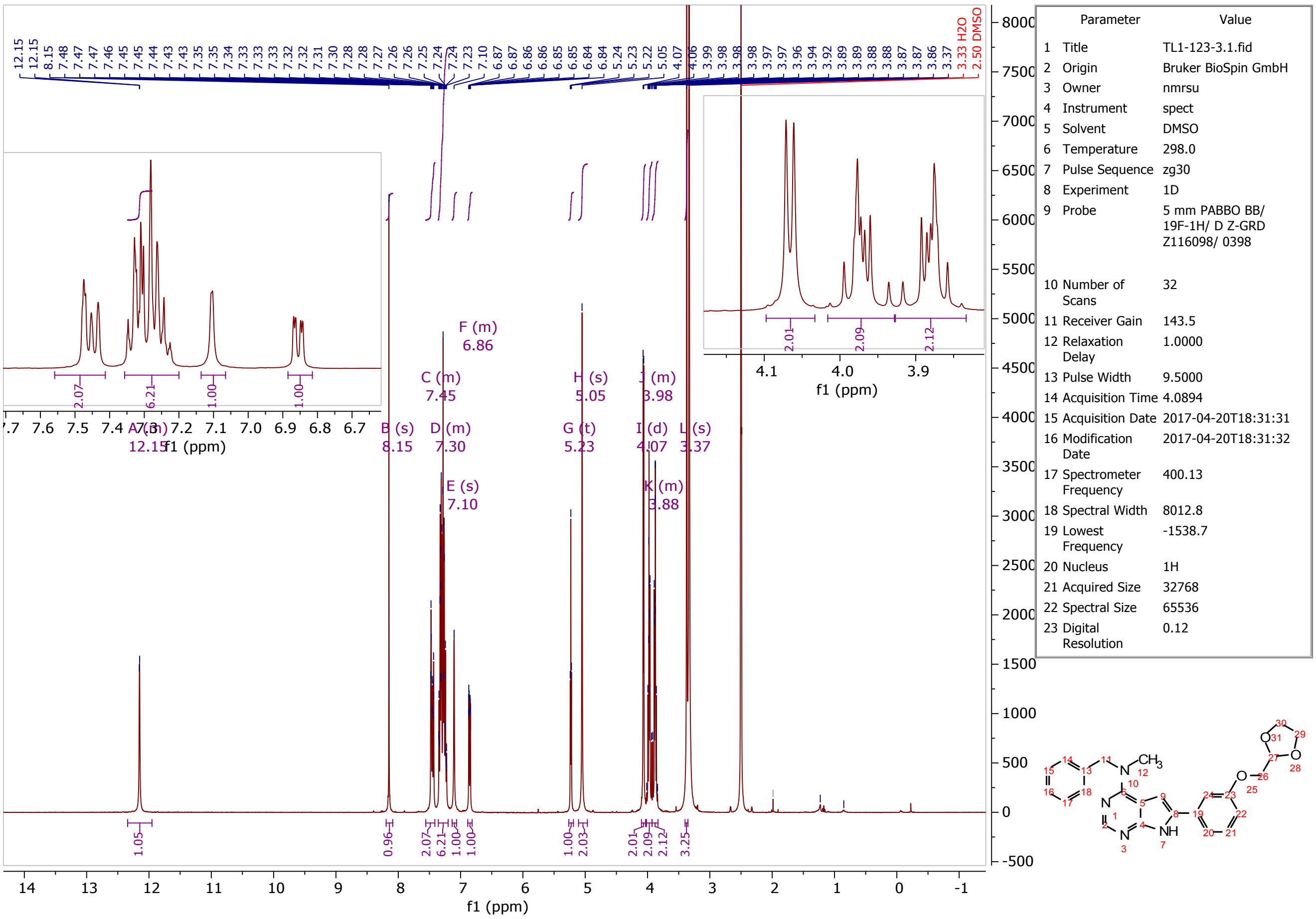

Figure S21. ^{1}H NMR (400 MHz, DMSO-d_6) of compound **7**.

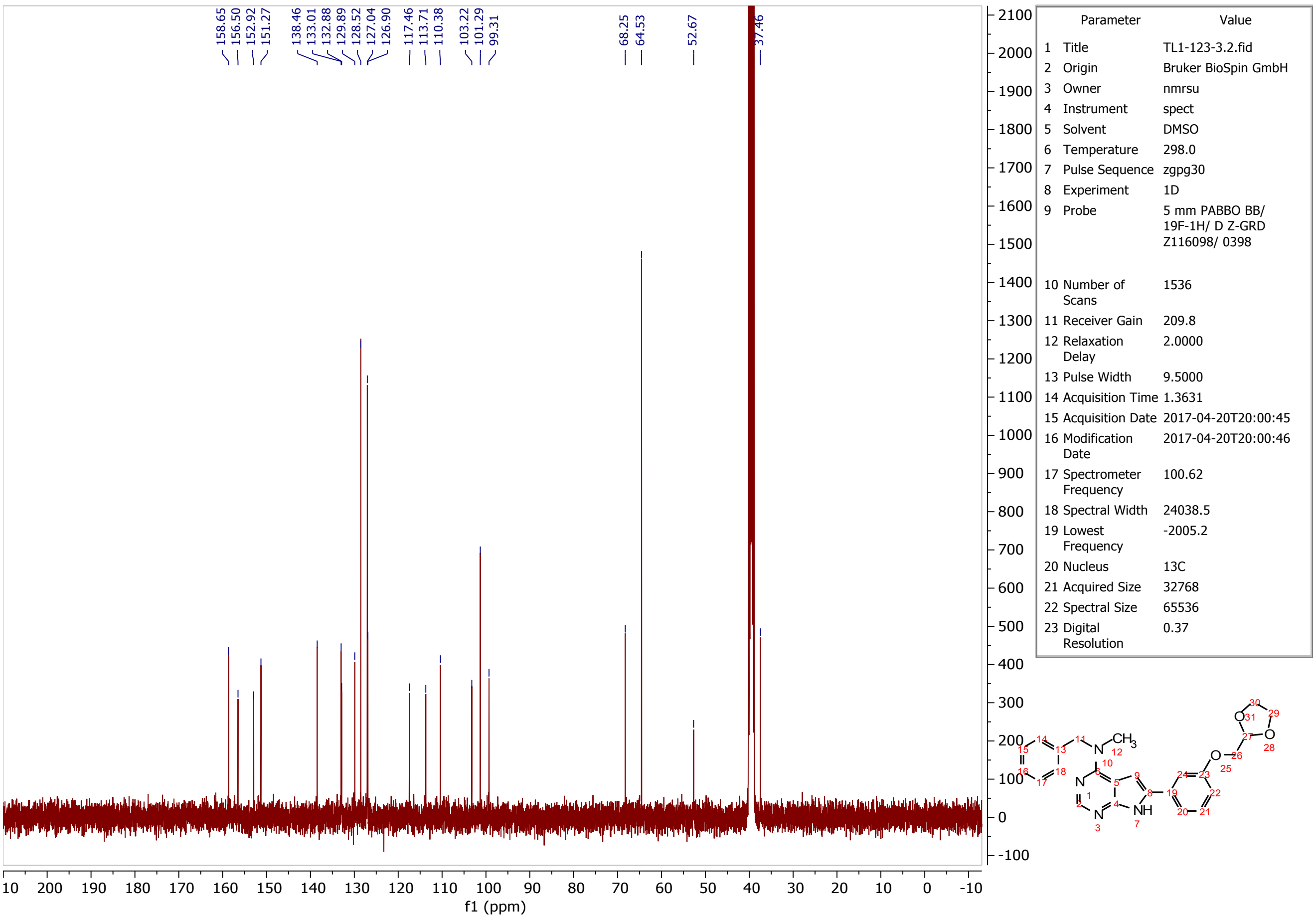

Figure S22. ^{13}C NMR (100 MHz, DMSO-d_6) of compound **7**.

Compound 8

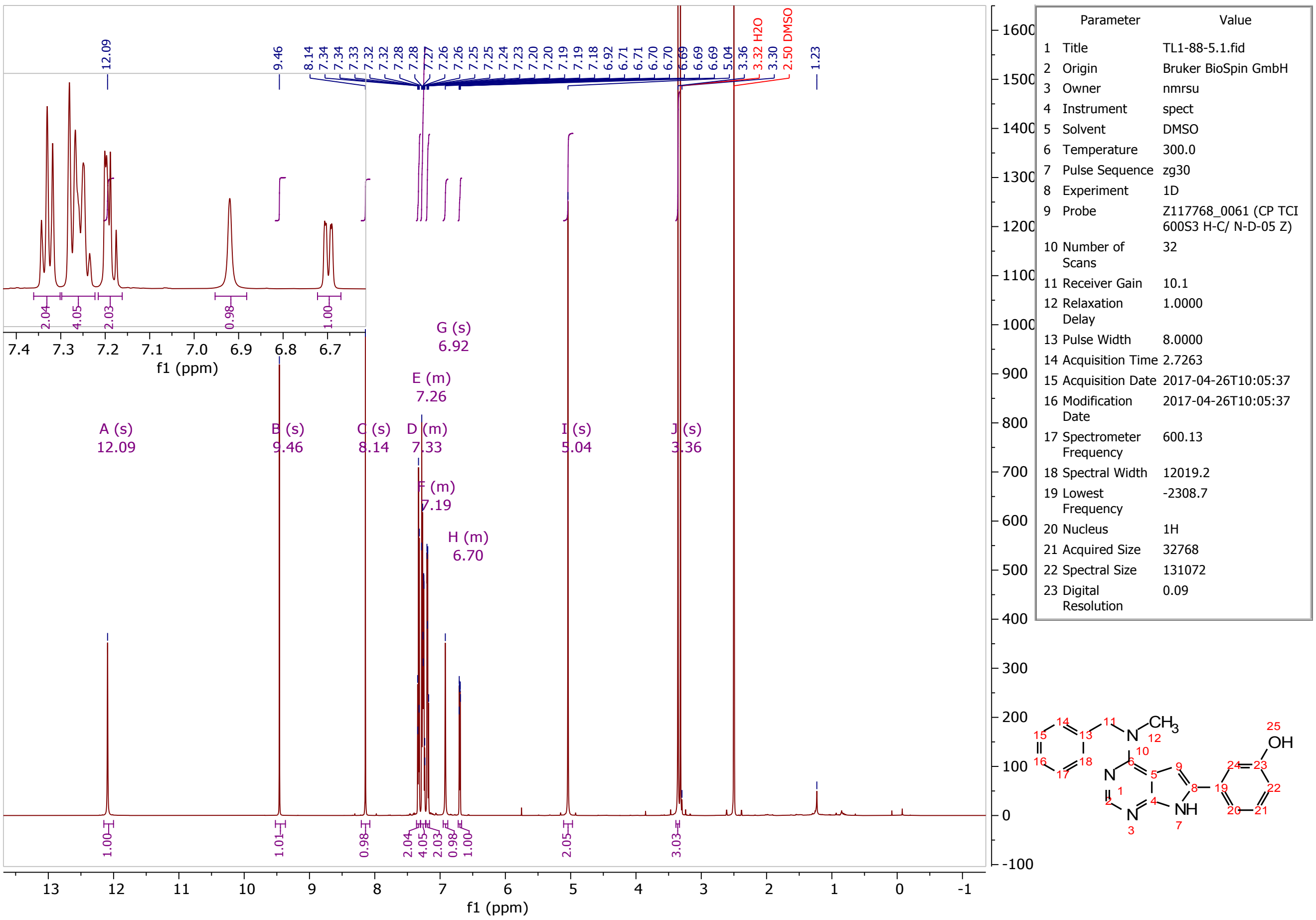

Figure S23. ^{1}H NMR (600 MHz, DMSO-d_6) of compound **8**.

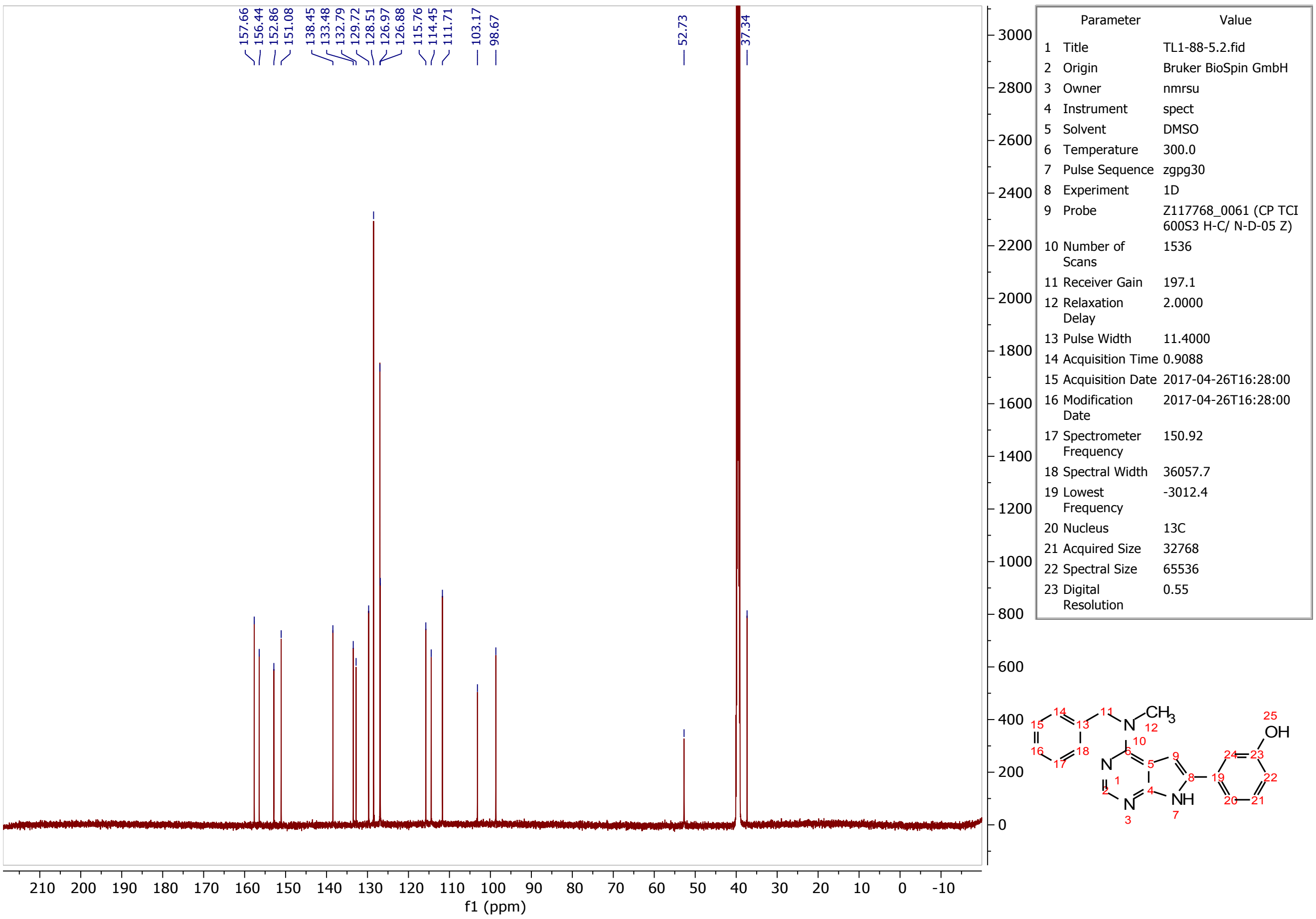

Figure S24. ^{13}C NMR (150 MHz, DMSO-d_6) of compound **8**.

Compound 9

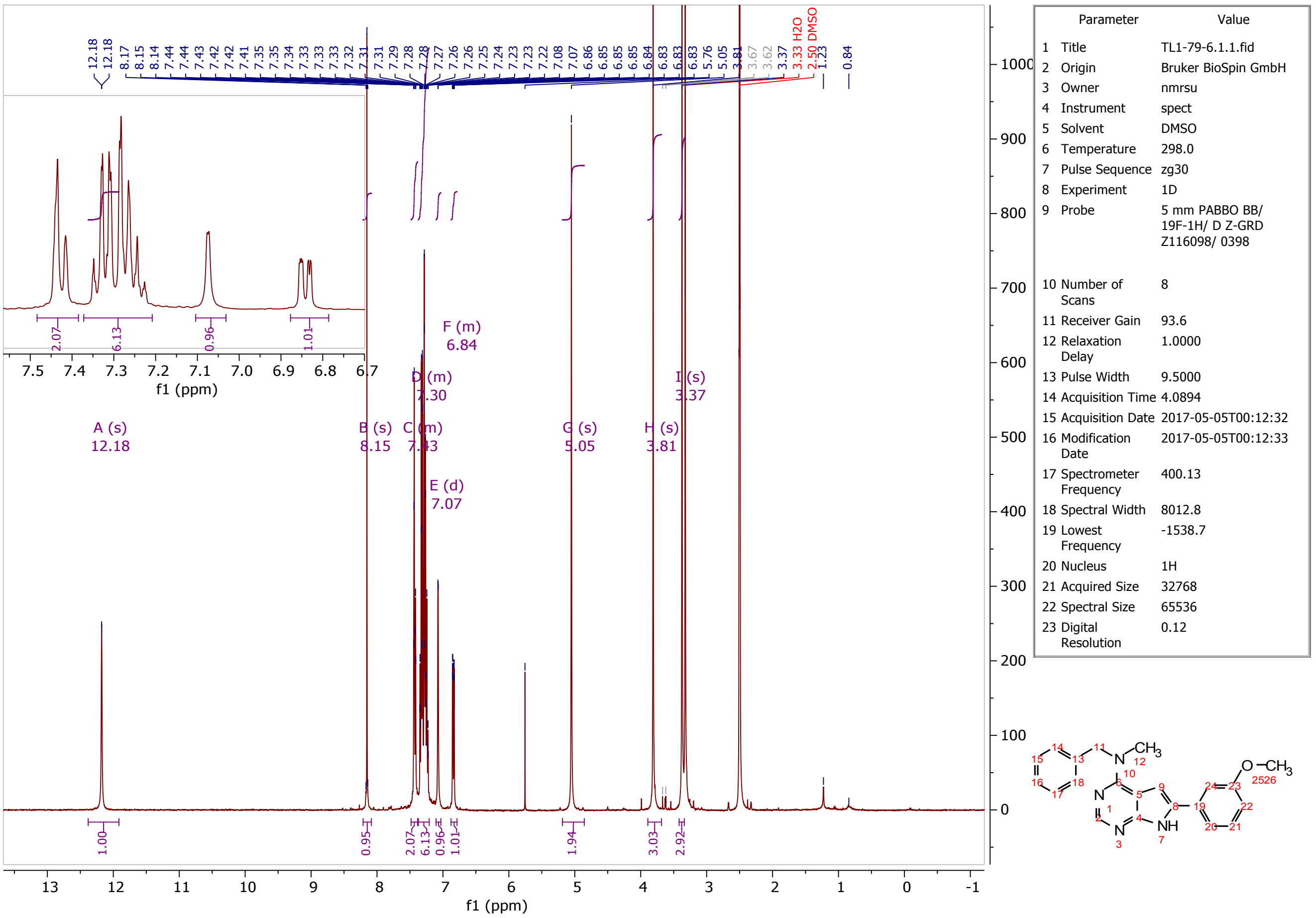

Figure S25. ^{1}H NMR (400 MHz, DMSO-d_6) of compound **9**.

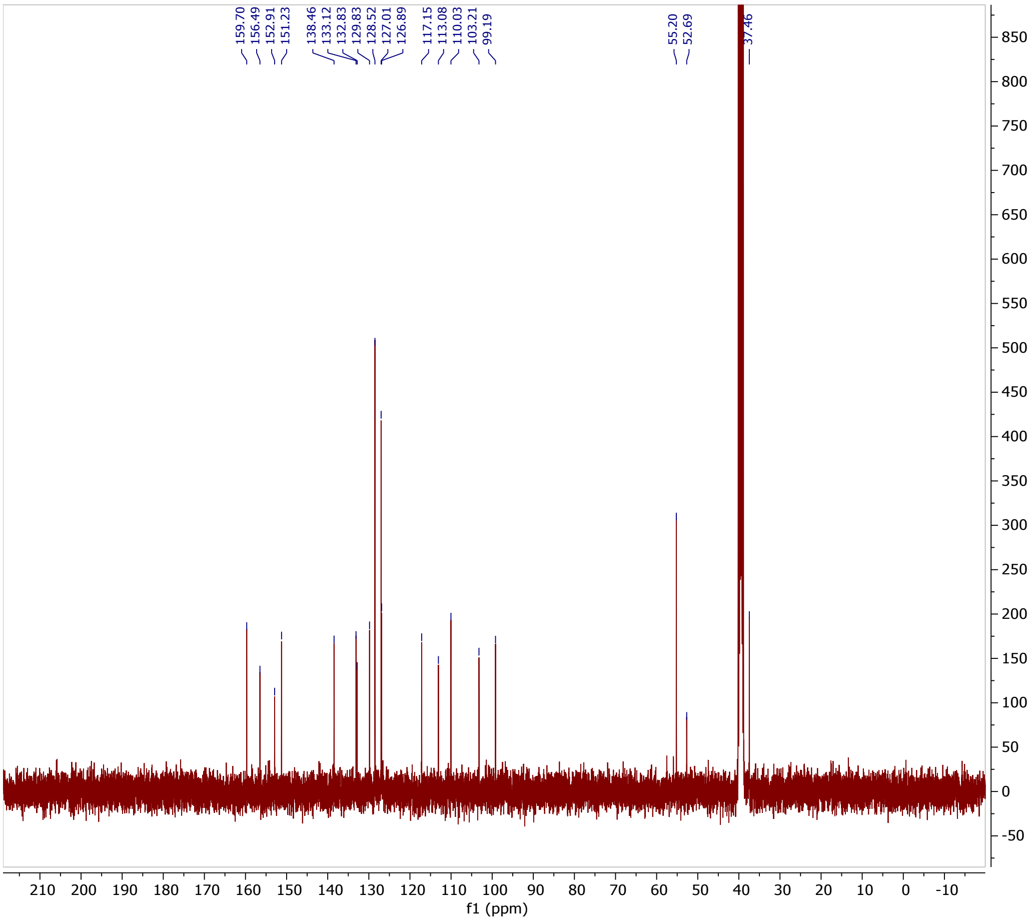

	Parameter	Value
1	Title	TL1-79-6.1.2.fid
2	Origin	Bruker BioSpin GmbH
3	Owner	nmrsu
4	Instrument	spect
5	Solvent	DMSO
6	Temperature	298.0
7	Pulse Sequence	zgpg30
8	Experiment	1D
9	Probe	5 mm PABBO BB/ 19F-1H/ D Z-GRD Z116098/ 0398
10	Number of Scans	512
11	Receiver Gain	209.8
12	Relaxation Delay	2.0000
13	Pulse Width	9.5000
14	Acquisition Time	1.3631
15	Acquisition Date	2017-05-05T00:43:13
16	Modification Date	2017-05-05T00:43:15
17	Spectrometer Frequency	100.62
18	Spectral Width	24038.5
19	Lowest Frequency	-2006.0
20	Nucleus	13C
21	Acquired Size	32768
22	Spectral Size	65536
23	Digital Resolution	0.37

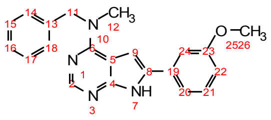

Figure S26. ^{13}C NMR (100 MHz, DMSO-d_6) of compound **9**.

Compound 10

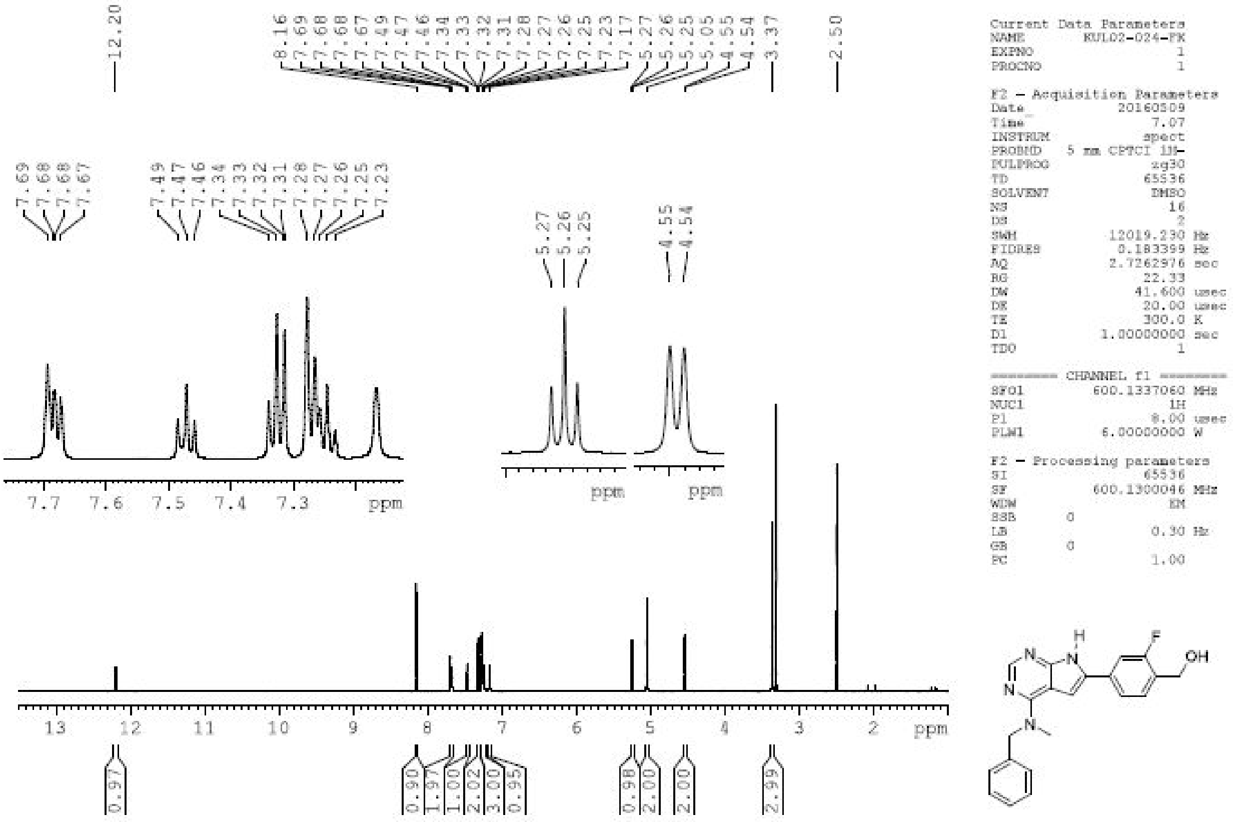

Figure S27. ^{1}H NMR (600 MHz, DMSO-d_6) of compound **10**.

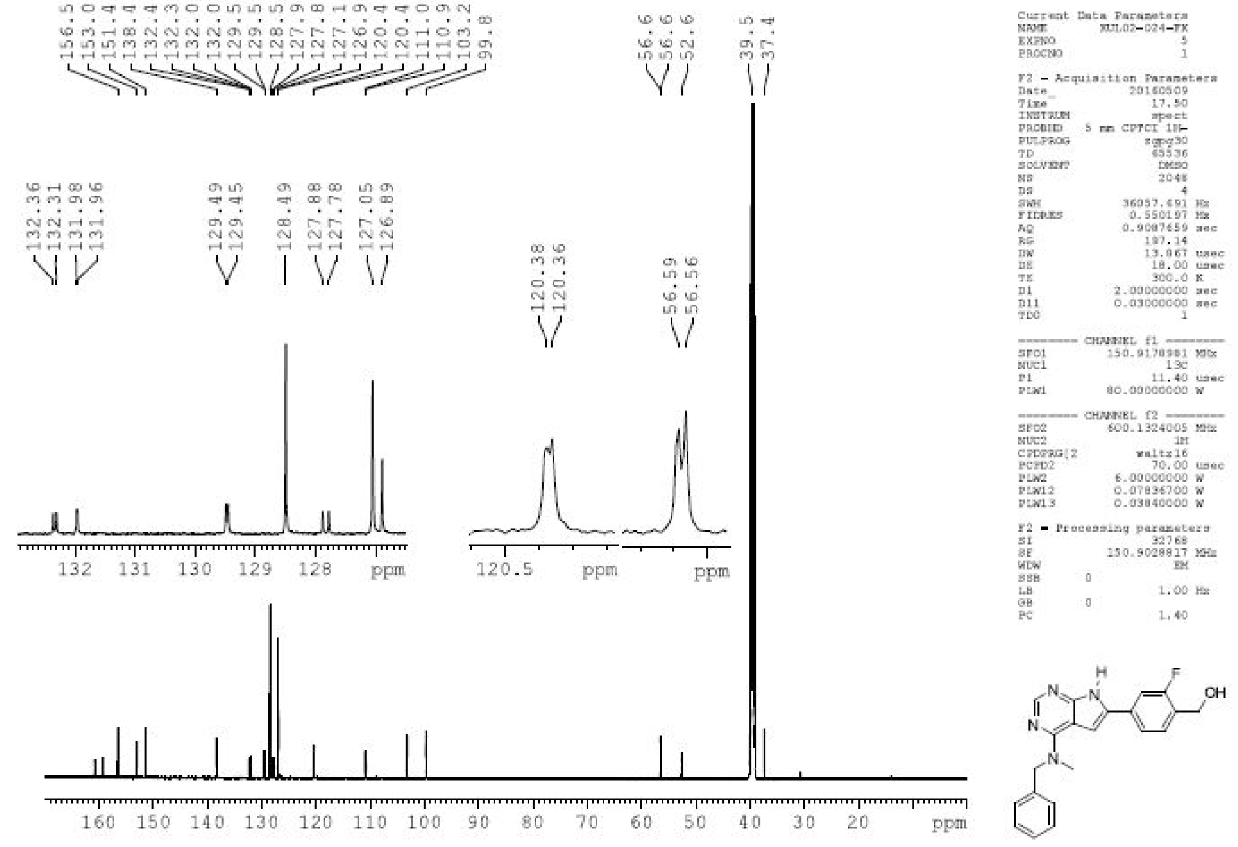

Figure S28. ^{13}C NMR (150 MHz, DMSO-d_6) of compound **10.**

Compound 11

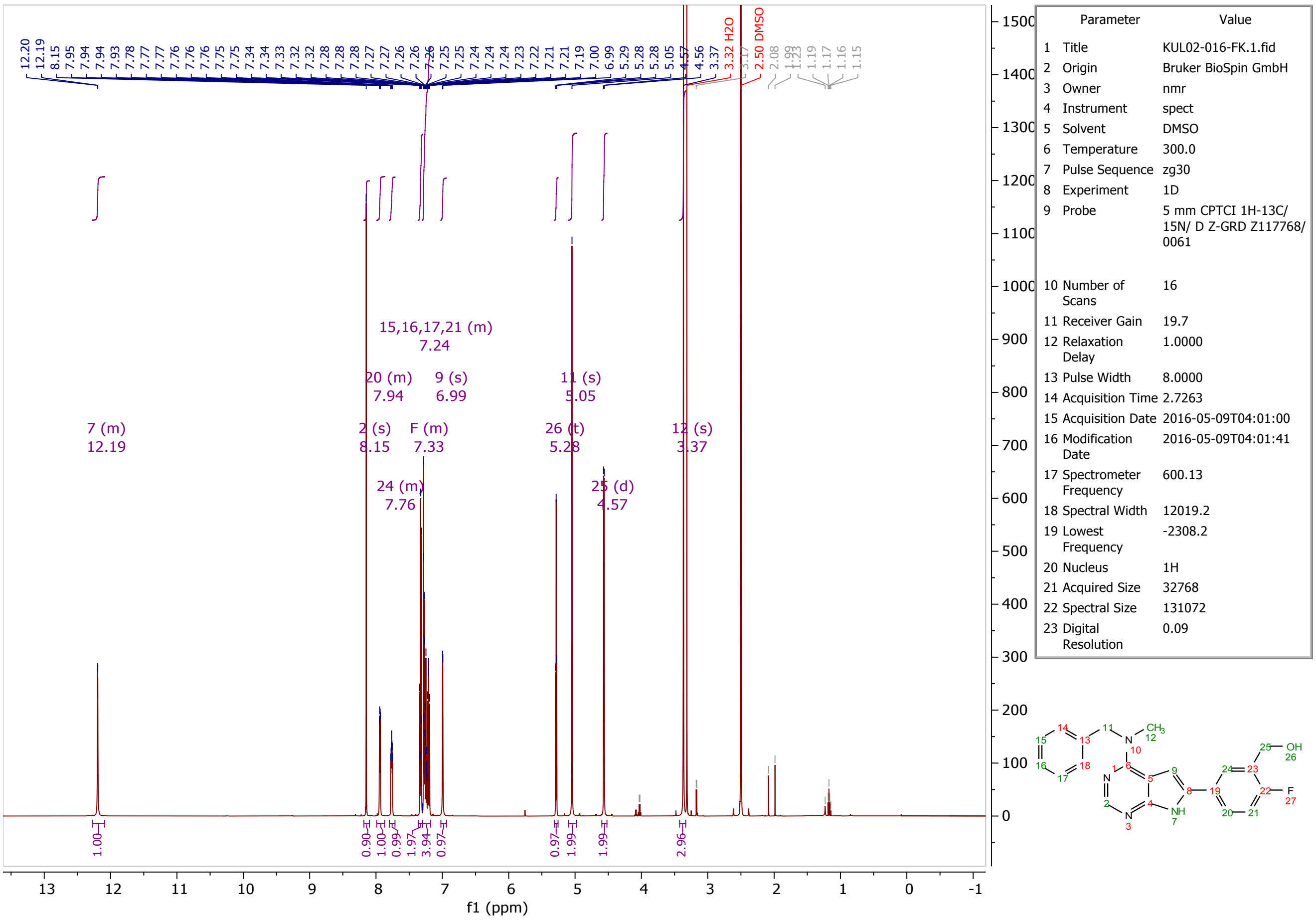

Figure S29. ^{1}H NMR (600 MHz, DMSO-d_6) of compound **11**.

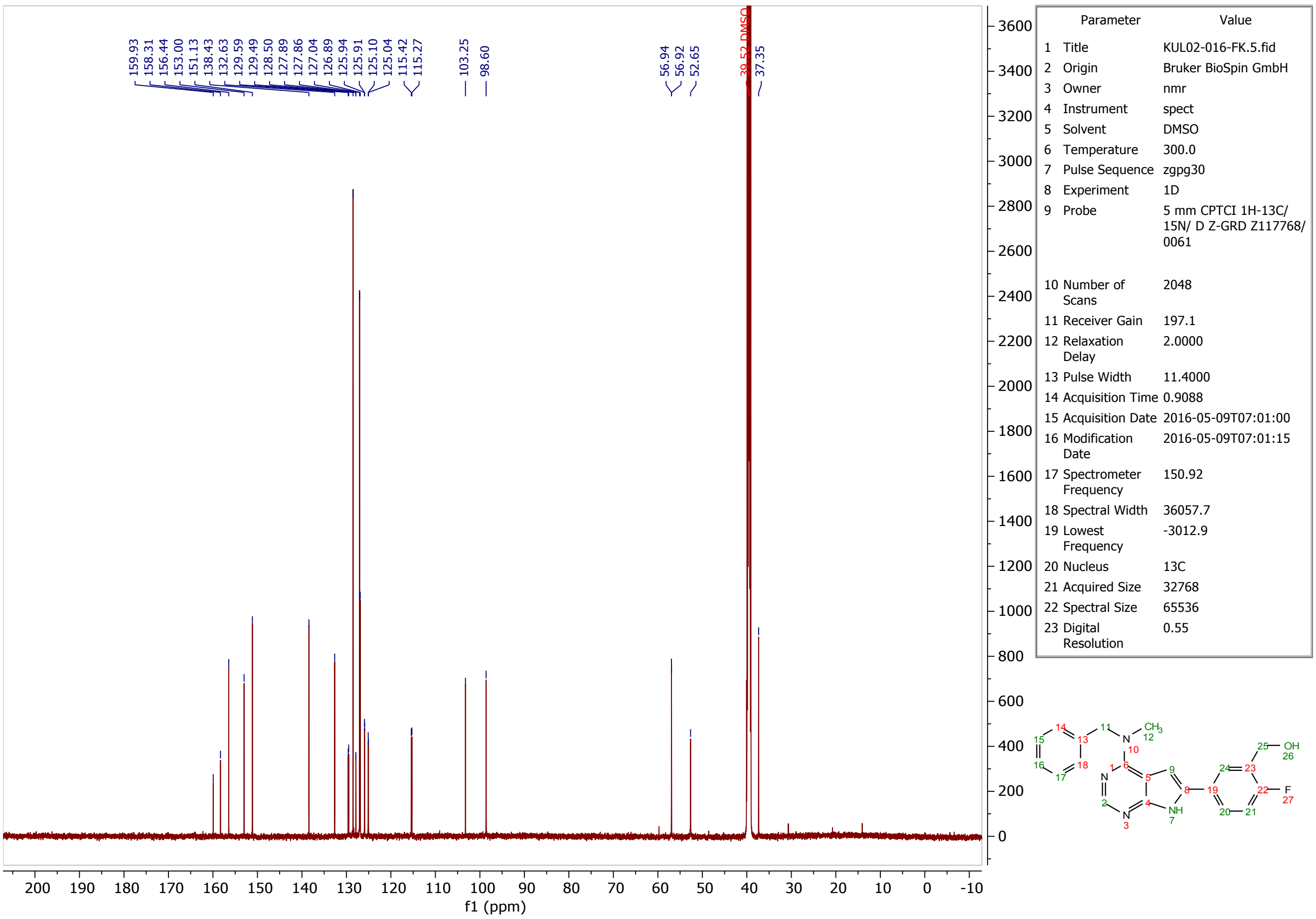

Figure S30. ^{13}C NMR (150 MHz, DMSO-d_6) of compound **11.**

Compound 12

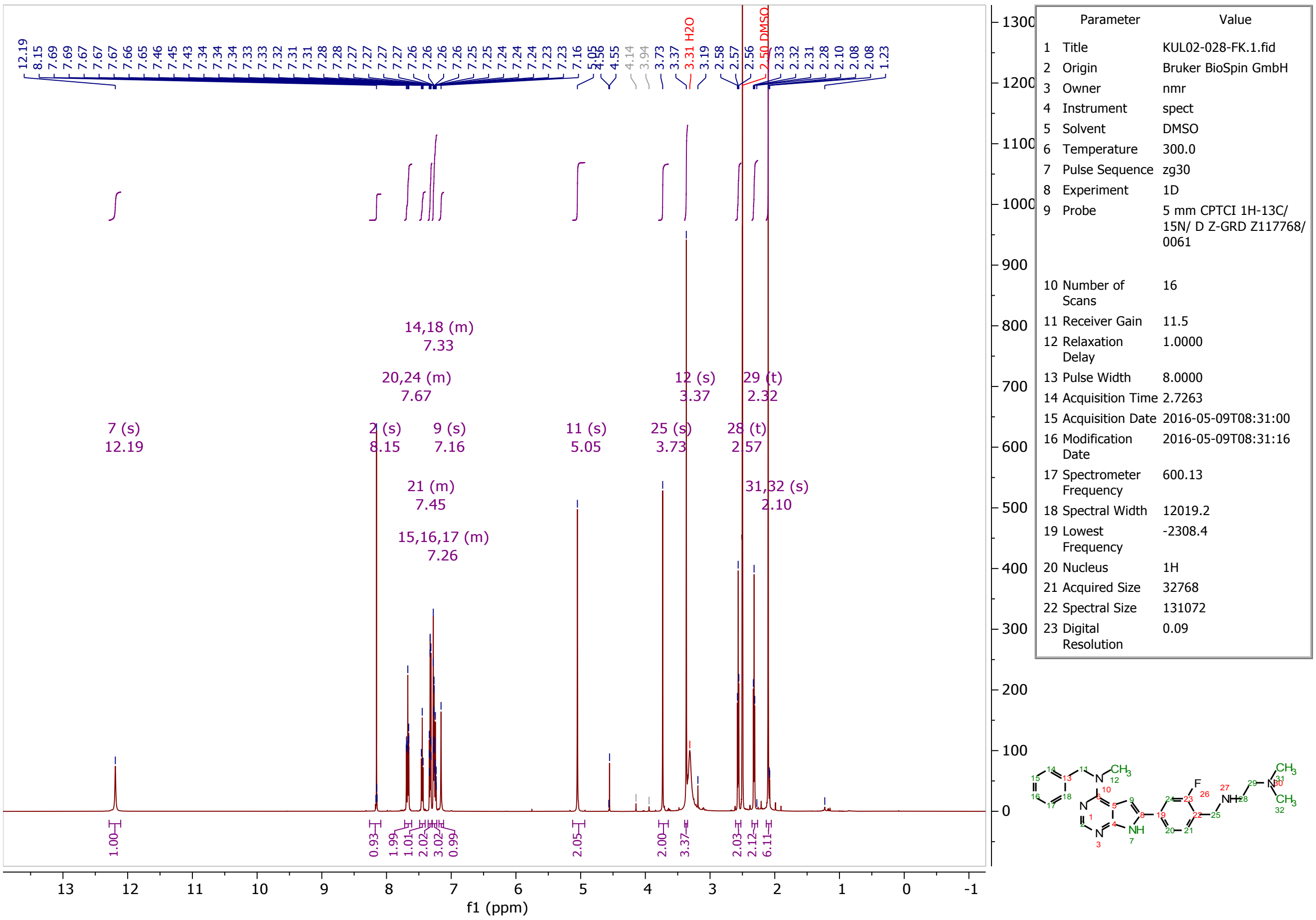

Figure S31. ^{1}H NMR (600 MHz, DMSO-d_6) of compound **12**.

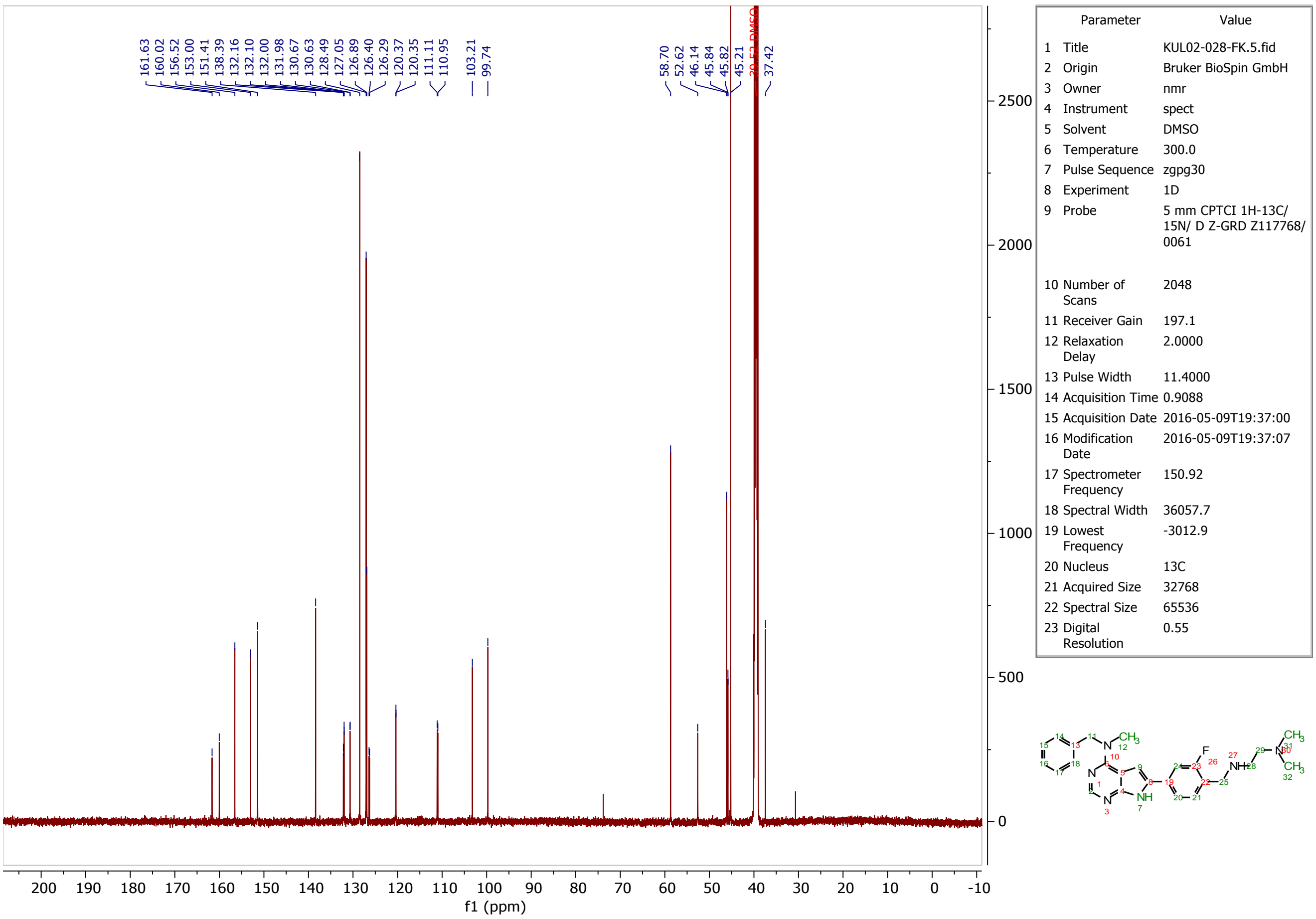

Figure S32. ^{13}C NMR (150 MHz, DMSO-d_6) of compound **12.**

Compound 13

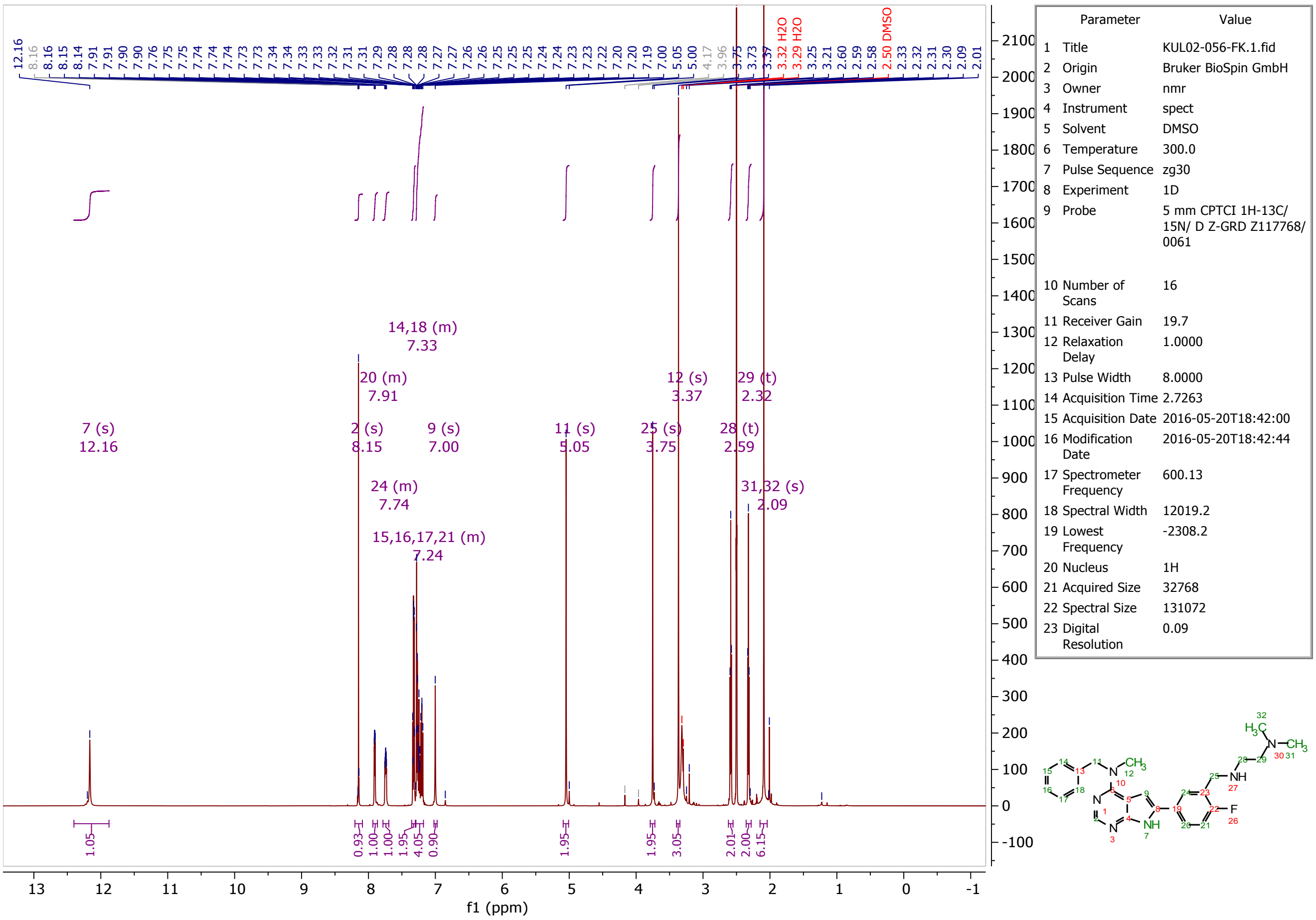

Figure S33. ^{1}H NMR (600 MHz, DMSO-d_6) of compound **13**.

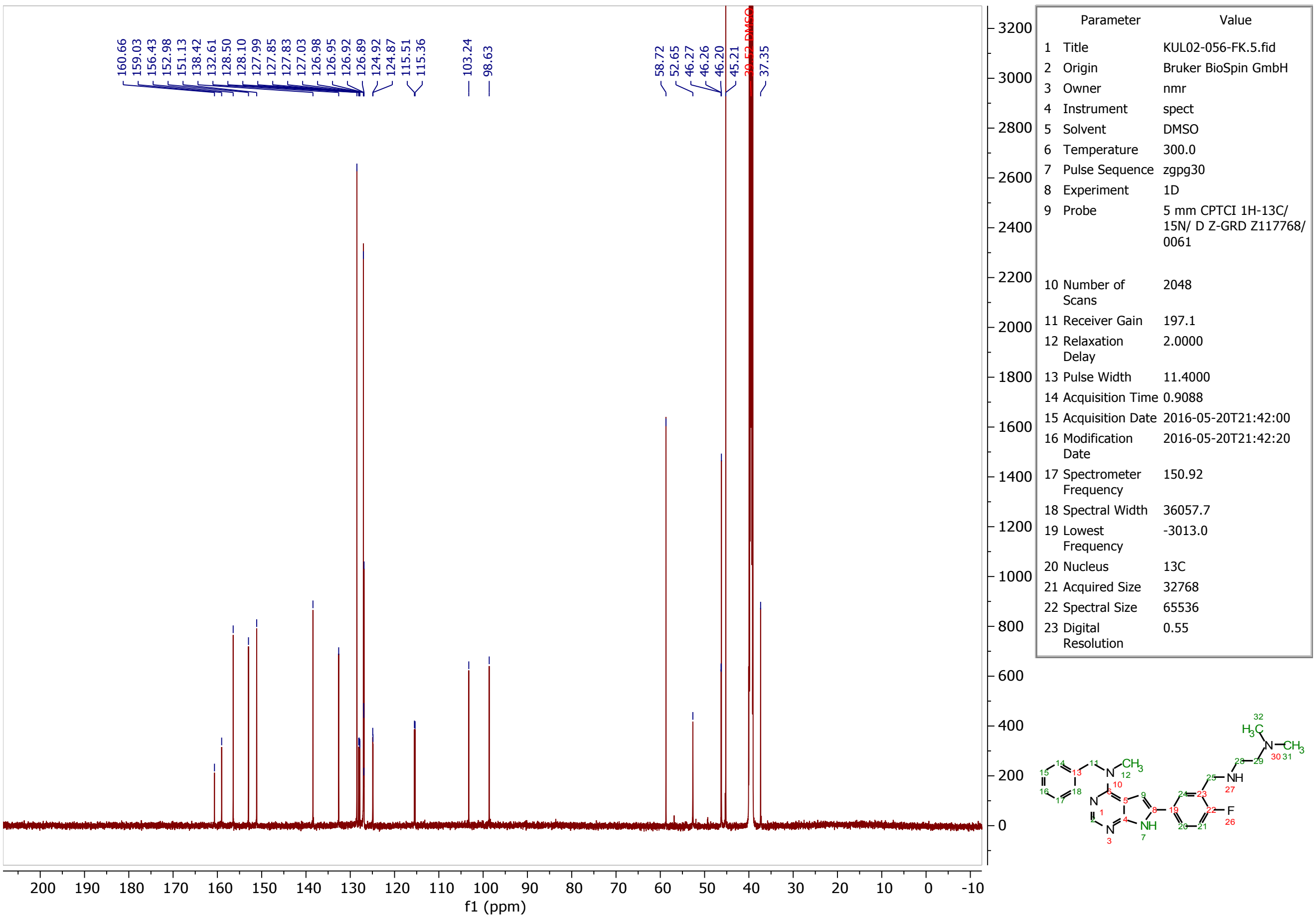

Figure S34. ^{13}C NMR (150 MHz, DMSO-d_6) of compound **13.**

Compound 14

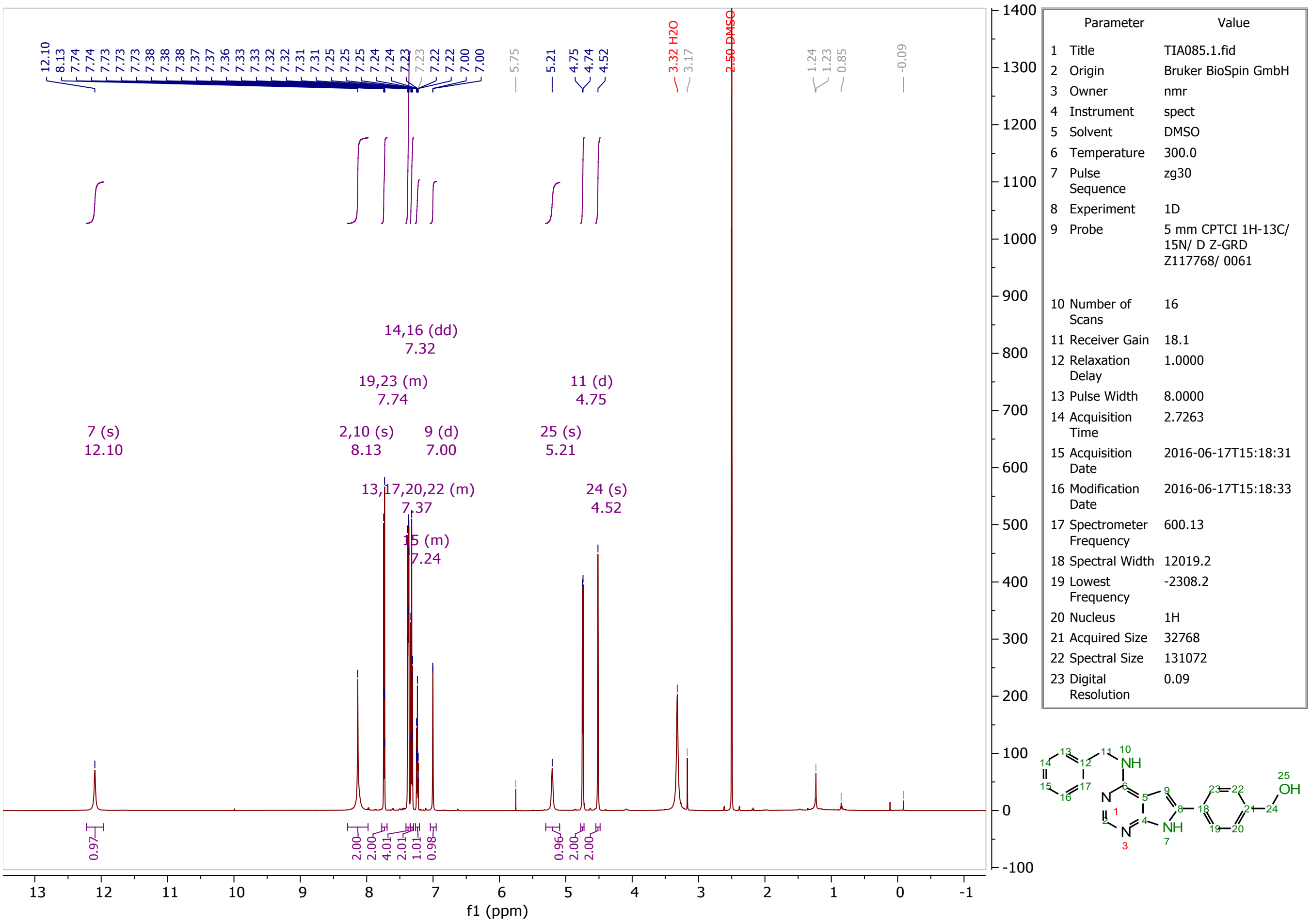

Figure S35. ^{1}H NMR (600 MHz, DMSO-d_6) of compound **14**.

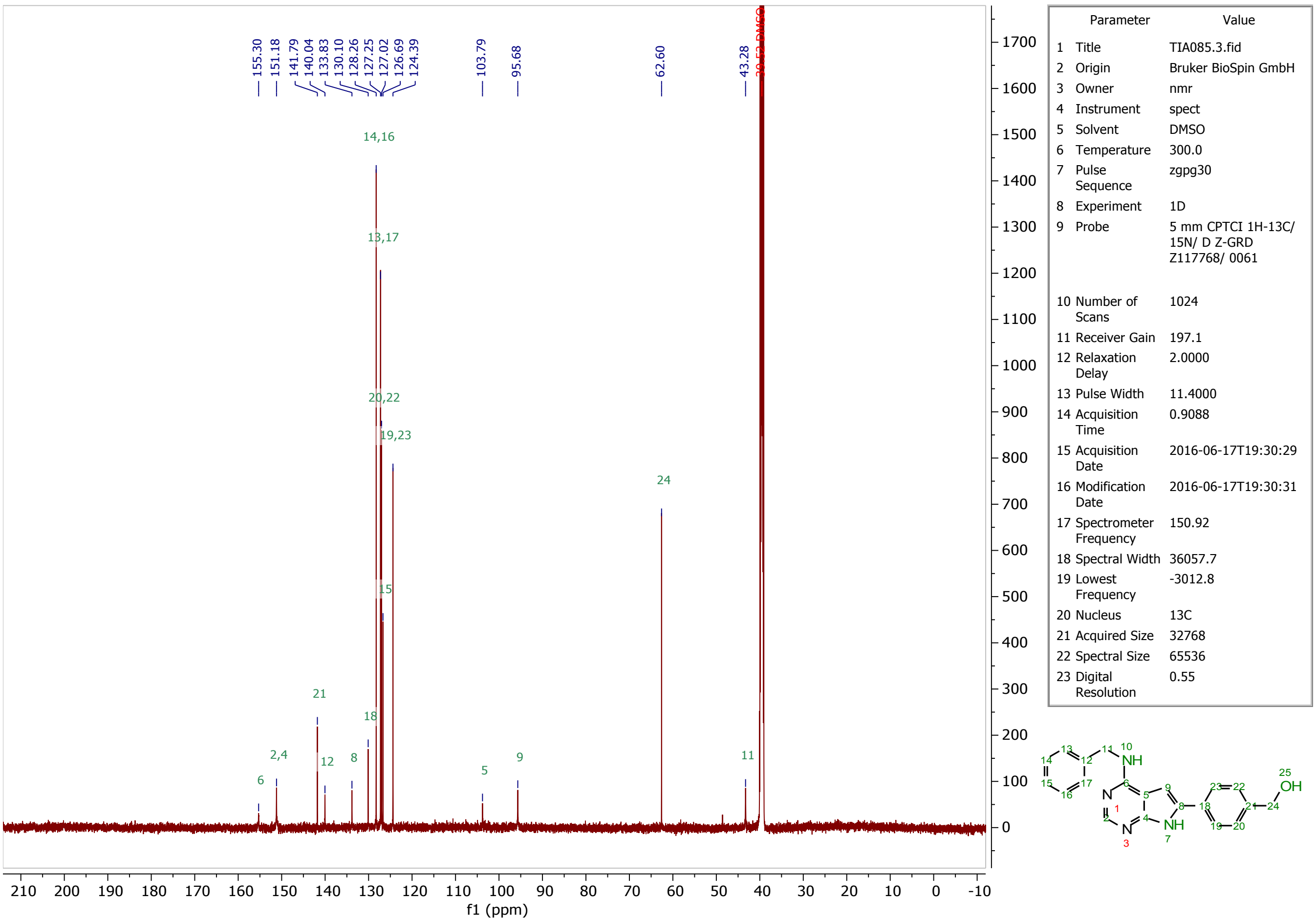

Parameter		Value
1	Title	TIA085.3.fid
2	Origin	Bruker BioSpin GmbH
3	Owner	nmr
4	Instrument	spect
5	Solvent	DMSO
6	Temperature	300.0
7	Pulse Sequence	zgpg30
8	Experiment	1D
9	Probe	5 mm CPTCI 1H-13C/ 15N/ D Z-GRD Z117768/ 0061
10	Number of Scans	1024
11	Receiver Gain	197.1
12	Relaxation Delay	2.0000
13	Pulse Width	11.4000
14	Acquisition Time	0.9088
15	Acquisition Date	2016-06-17T19:30:29
16	Modification Date	2016-06-17T19:30:31
17	Spectrometer Frequency	150.92
18	Spectral Width	36057.7
19	Lowest Frequency	-3012.8
20	Nucleus	13C
21	Acquired Size	32768
22	Spectral Size	65536
23	Digital Resolution	0.55

Figure S36. ^{13}C NMR (150 MHz, DMSO-d_6) of compound **14.**

Compound 15

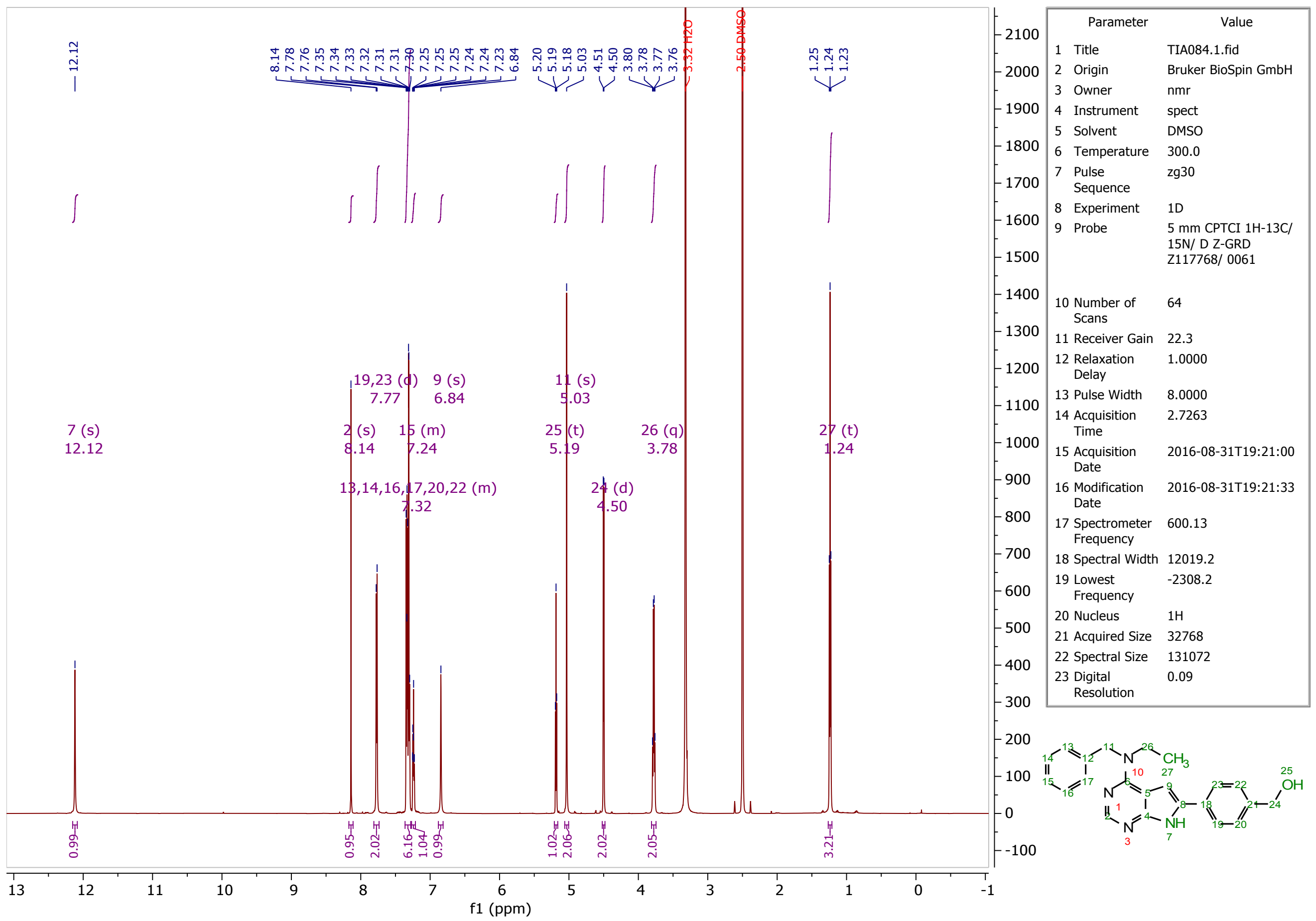

Figure S37. ^{1}H NMR (600 MHz, DMSO-d_6) of compound **15**.

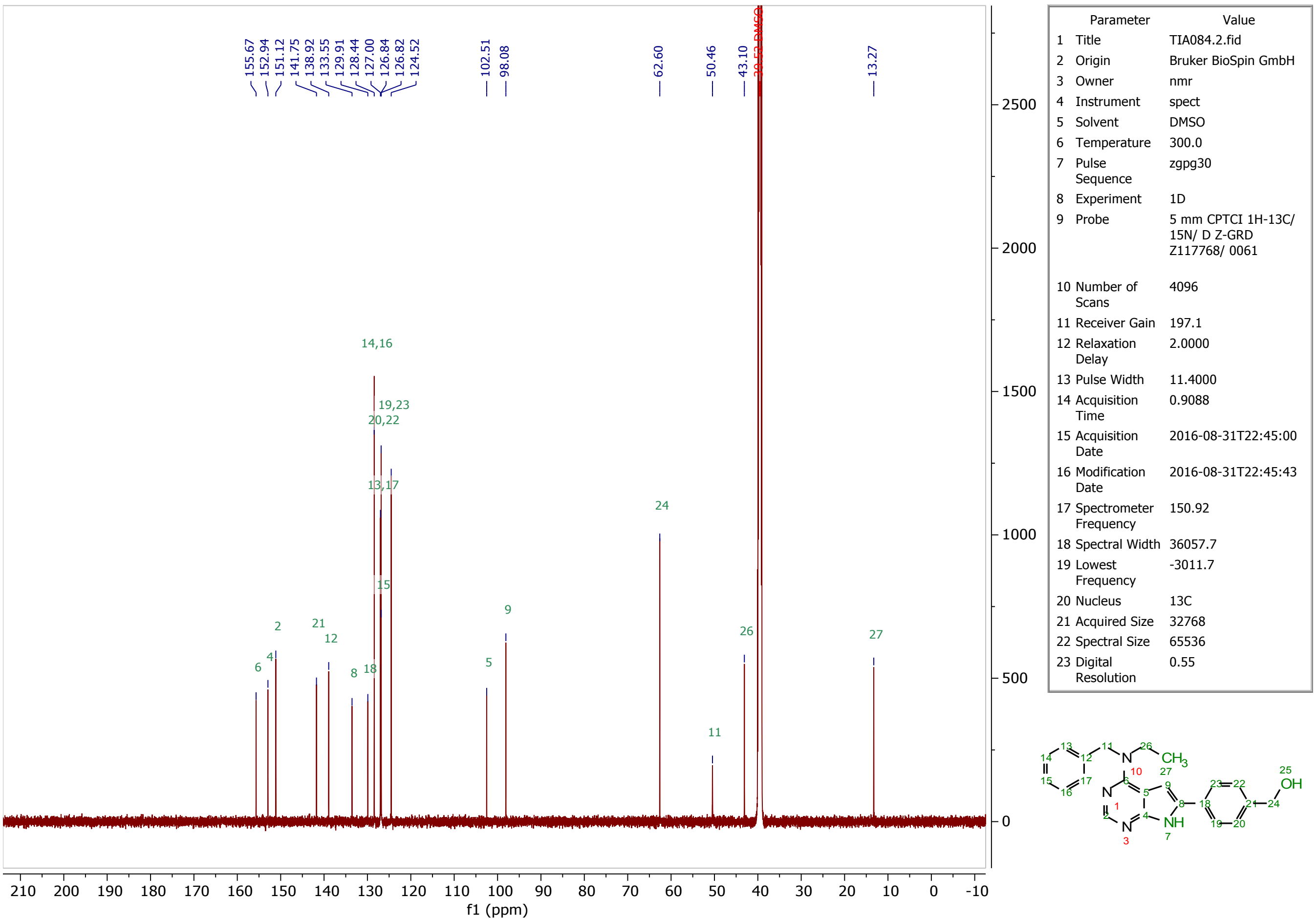

Figure S38. ^{13}C NMR (150 MHz, DMSO-d_6) of compound **15.**

Compound 16

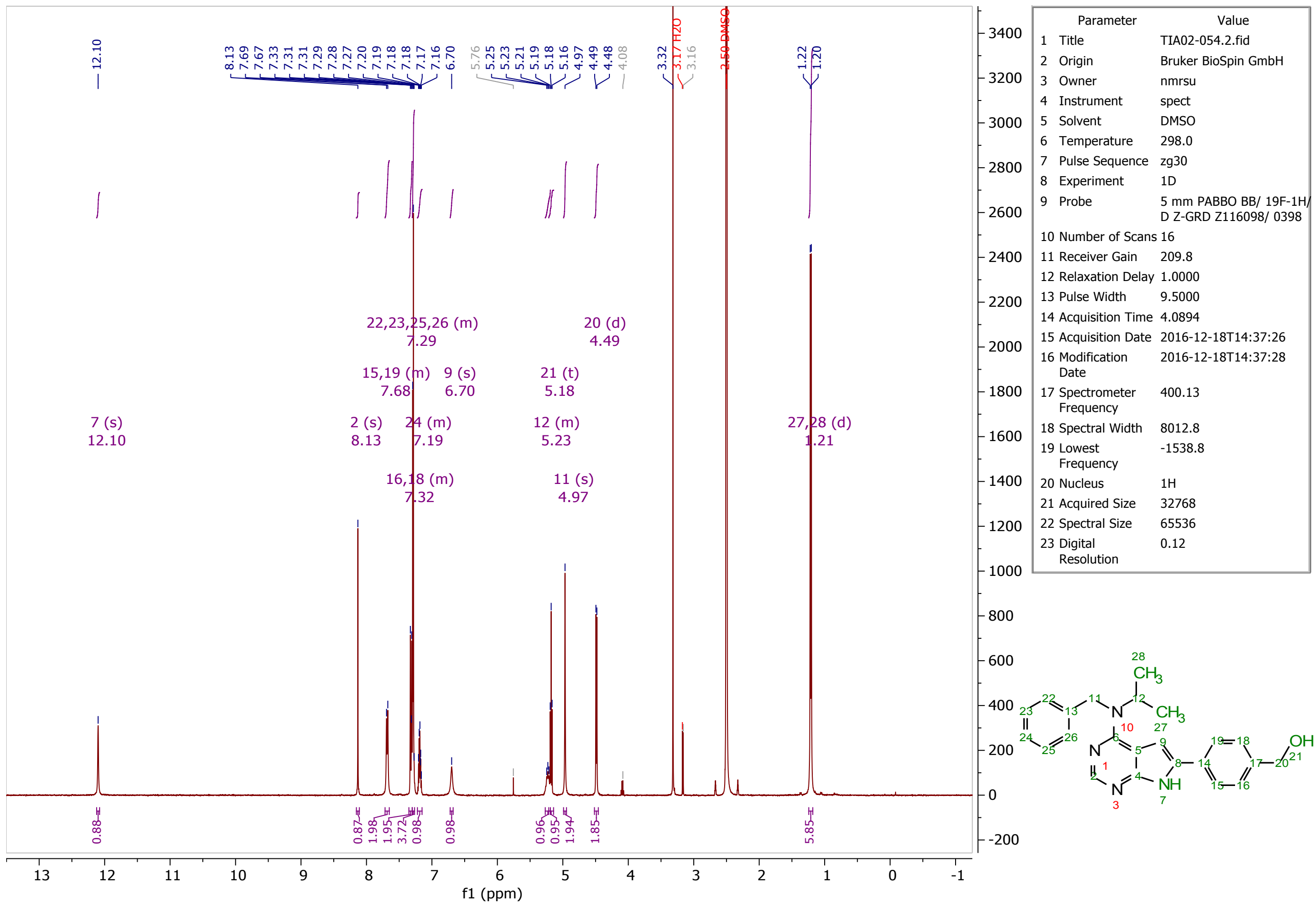

Figure S39. ^{1}H NMR (400 MHz, DMSO-d_6) of compound **16**.

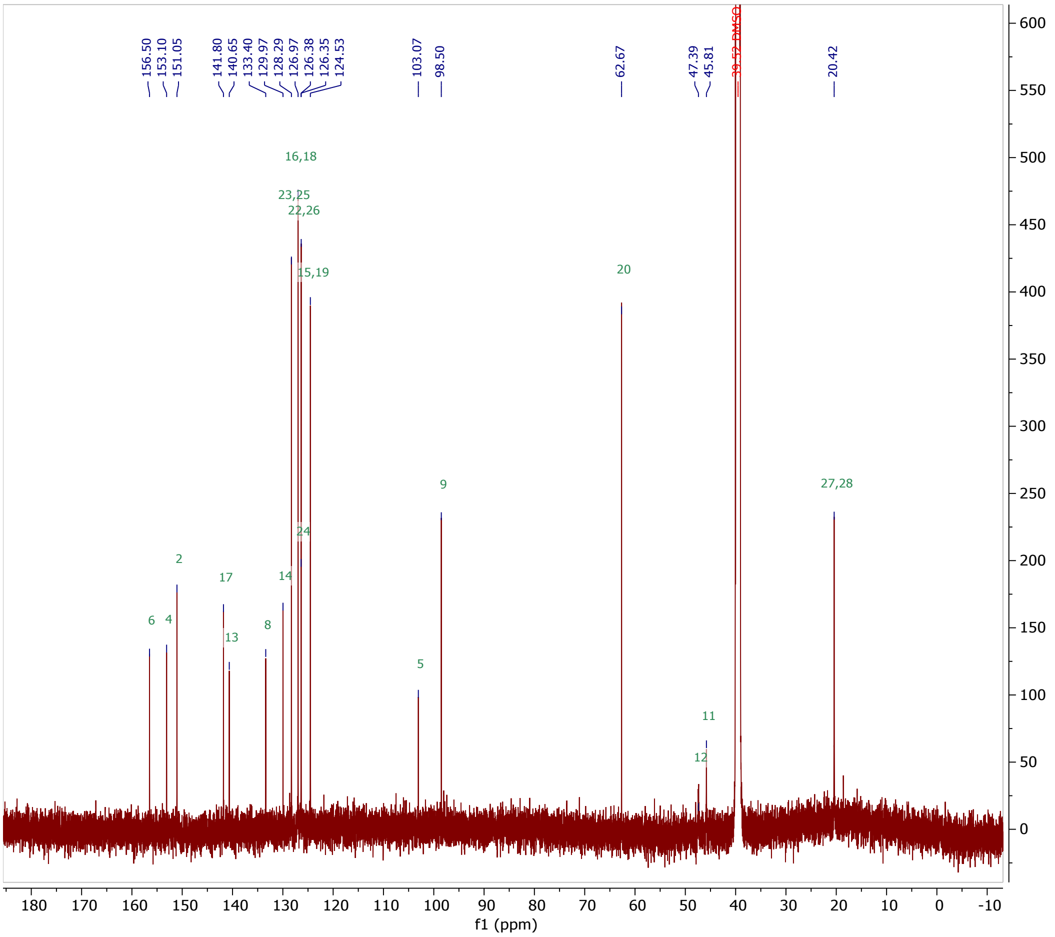

Parameter		Value
1	Title	TIA02-054.2.fid
2	Origin	Bruker BioSpin GmbH
3	Owner	nmrsu
4	Instrument	spect
5	Solvent	DMSO
6	Temperature	300.0
7	Pulse Sequence	zgpg30
8	Experiment	1D
9	Probe	Z117768_0061 (CP TCI 600S3 H-C/ N-D-05 Z)
10	Number of Scans	2048
11	Receiver Gain	197.1
12	Relaxation Delay	2.0000
13	Pulse Width	11.4000
14	Acquisition Time	0.9044
15	Acquisition Date	2021-01-16T06:05:29
16	Modification Date	2021-01-16T06:05:29
17	Spectrometer Frequency	150.93
18	Spectral Width	36231.9
19	Lowest Frequency	-3082.0
20	Nucleus	13C
21	Acquired Size	32768
22	Spectral Size	65536
23	Digital Resolution	0.55

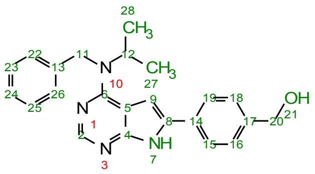

Figure S40. ^{13}C NMR (150 MHz, DMSO-d_6) of compound **16.**

Compound 18

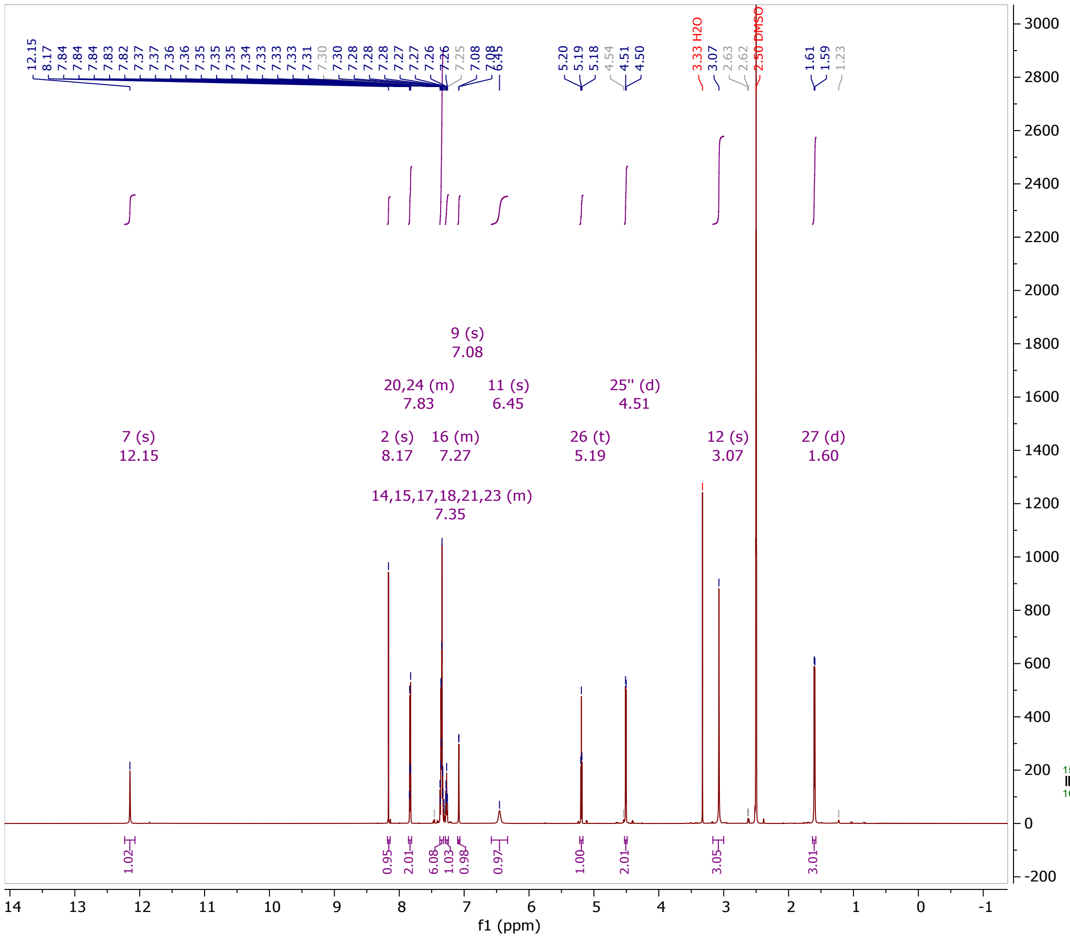

	Parameter	Value
1	Title	TIA05-088-1.1.fid
2	Origin	Bruker BioSpin GmbH
3	Owner	nmrsu
4	Instrument	spect
5	Solvent	DMSO
6	Temperature	298.0
7	Pulse Sequence	zg30
8	Experiment	1D
9	Probe	Z117768_0061 (CP TCI 600S3 H-C/ N-D-05 Z)
10	Number of Scans	16
11	Receiver Gain	8.0
12	Relaxation Delay	2.0000
13	Pulse Width	8.0000
14	Acquisition Time	2.7263
15	Acquisition Date	2020-11-15T12:13:34
16	Modification Date	2020-11-15T12:13:34
17	Spectrometer Frequency	600.18
18	Spectral Width	12019.2
19	Lowest Frequency	-2308.2
20	Nucleus	1H
21	Acquired Size	32768
22	Spectral Size	131072
23	Digital Resolution	0.09

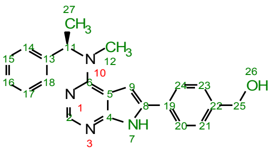

Figure S41. ^{1}H NMR (600 MHz, DMSO-d_6) of compound **18**.

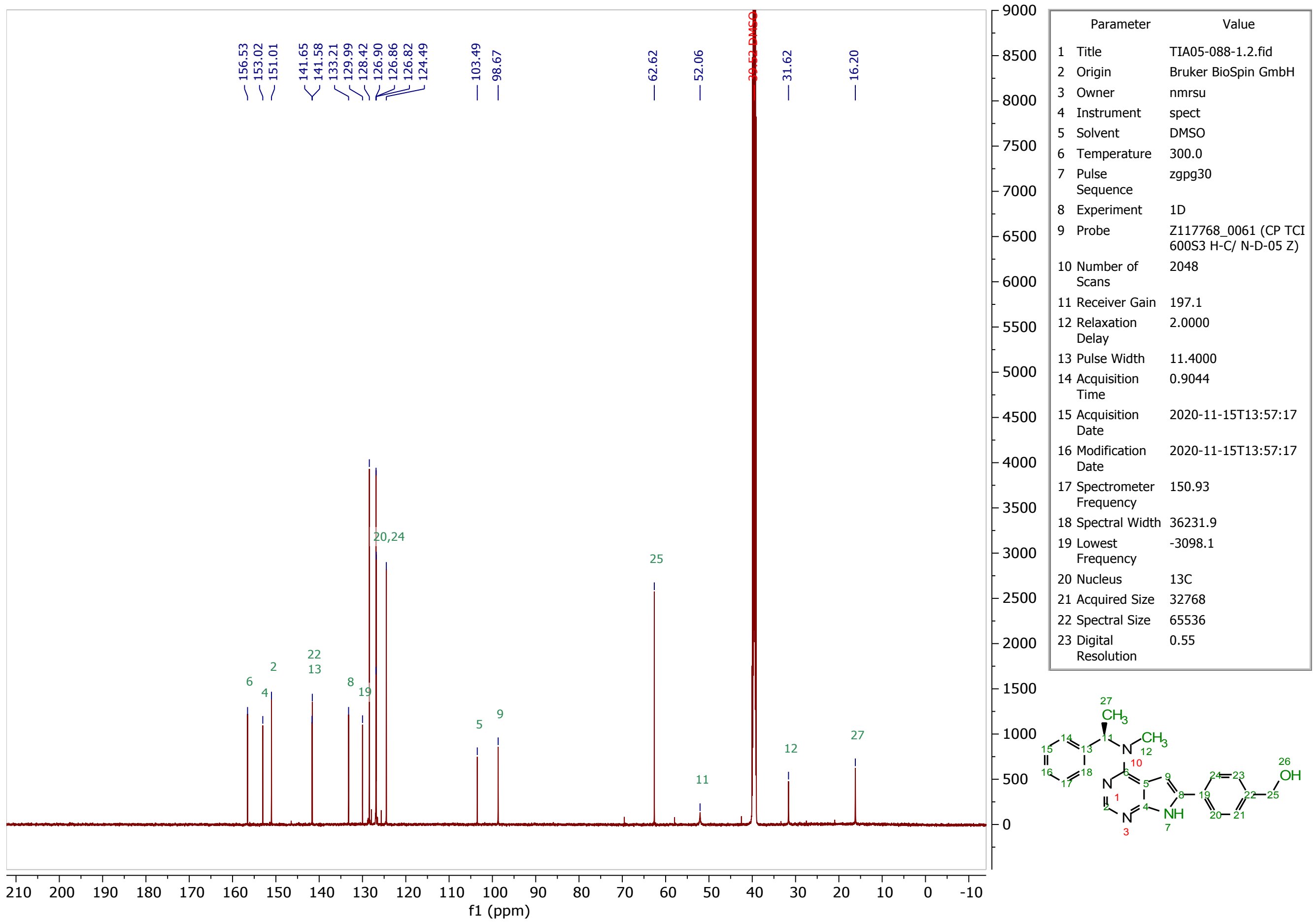

Figure S42. ^{13}C NMR (150 MHz, DMSO-d_6) of compound **18.**

Compound 20

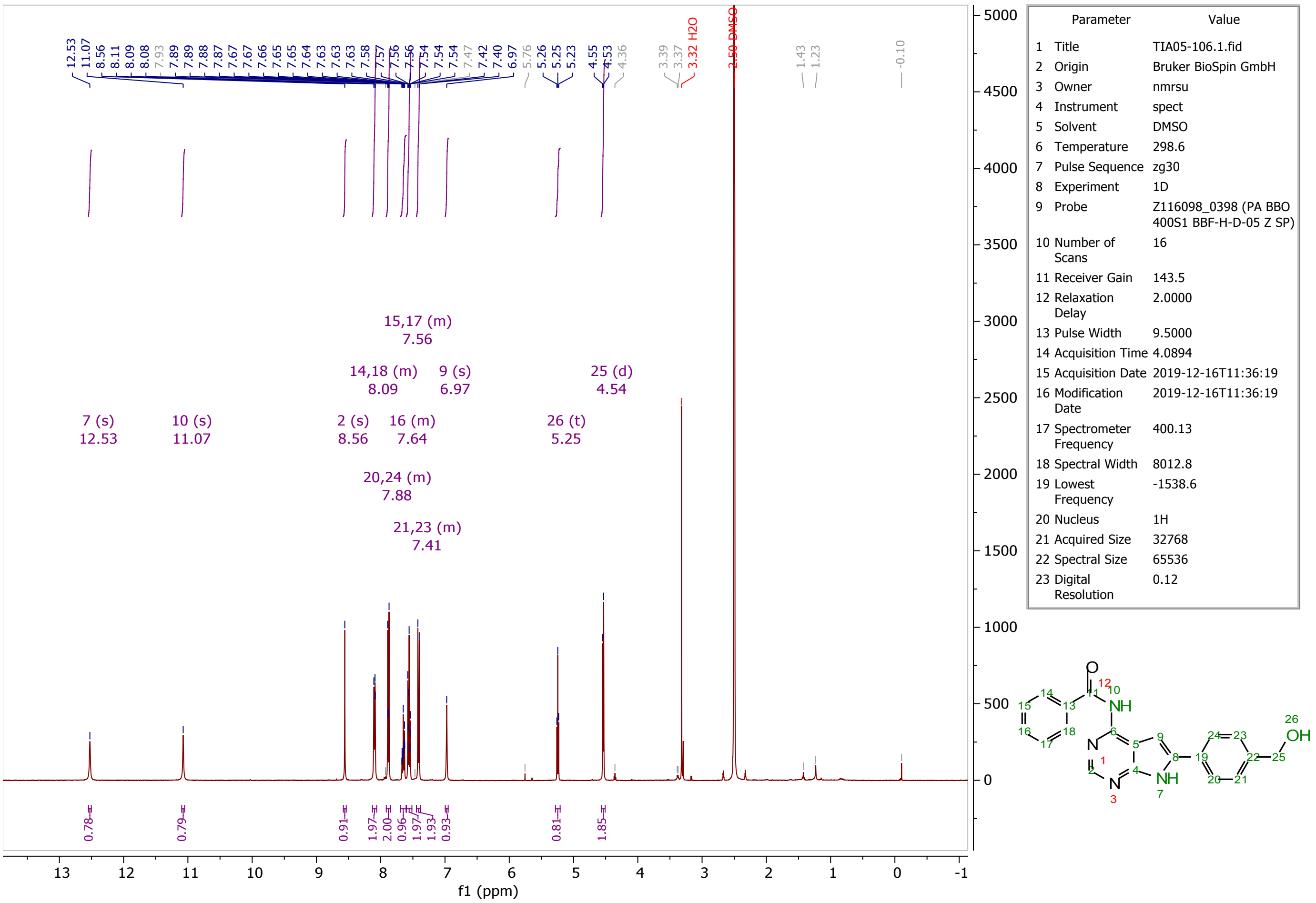

Figure S43. ^{1}H NMR (400 MHz, DMSO-d_6) of compound **20**.

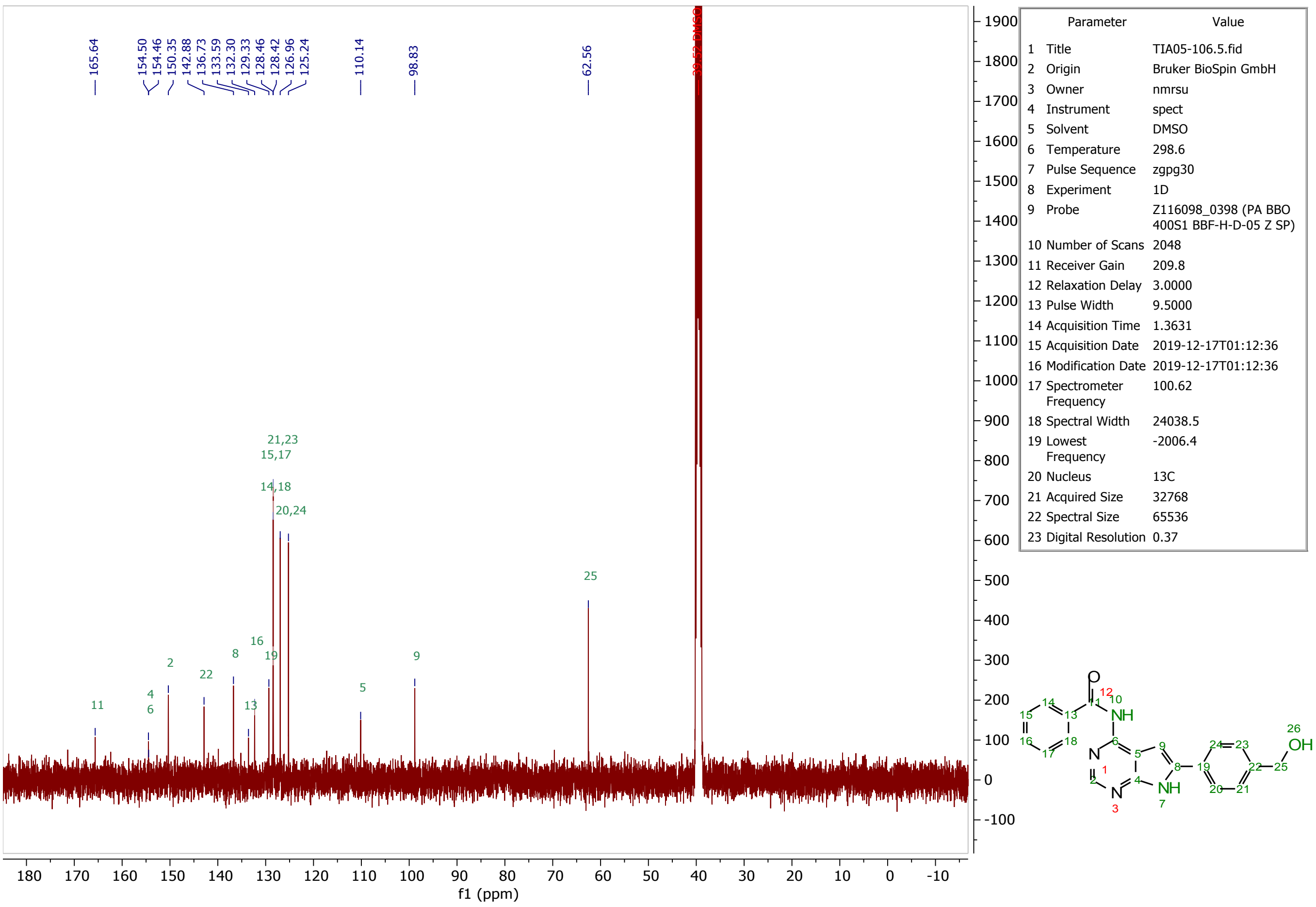

Parameter		Value
1	Title	TIA05-106.5.fid
2	Origin	Bruker BioSpin GmbH
3	Owner	nmrsu
4	Instrument	spect
5	Solvent	DMSO
6	Temperature	298.6
7	Pulse Sequence	zgpg30
8	Experiment	1D
9	Probe	Z116098_0398 (PA BBO 400S1 BBF-H-D-05 Z SP)
10	Number of Scans	2048
11	Receiver Gain	209.8
12	Relaxation Delay	3.0000
13	Pulse Width	9.5000
14	Acquisition Time	1.3631
15	Acquisition Date	2019-12-17T01:12:36
16	Modification Date	2019-12-17T01:12:36
17	Spectrometer Frequency	100.62
18	Spectral Width	24038.5
19	Lowest Frequency	-2006.4
20	Nucleus	13C
21	Acquired Size	32768
22	Spectral Size	65536
23	Digital Resolution	0.37

Figure S44. ^{13}C NMR (100 MHz, DMSO-d_6) of compound **20.**

Compound 21

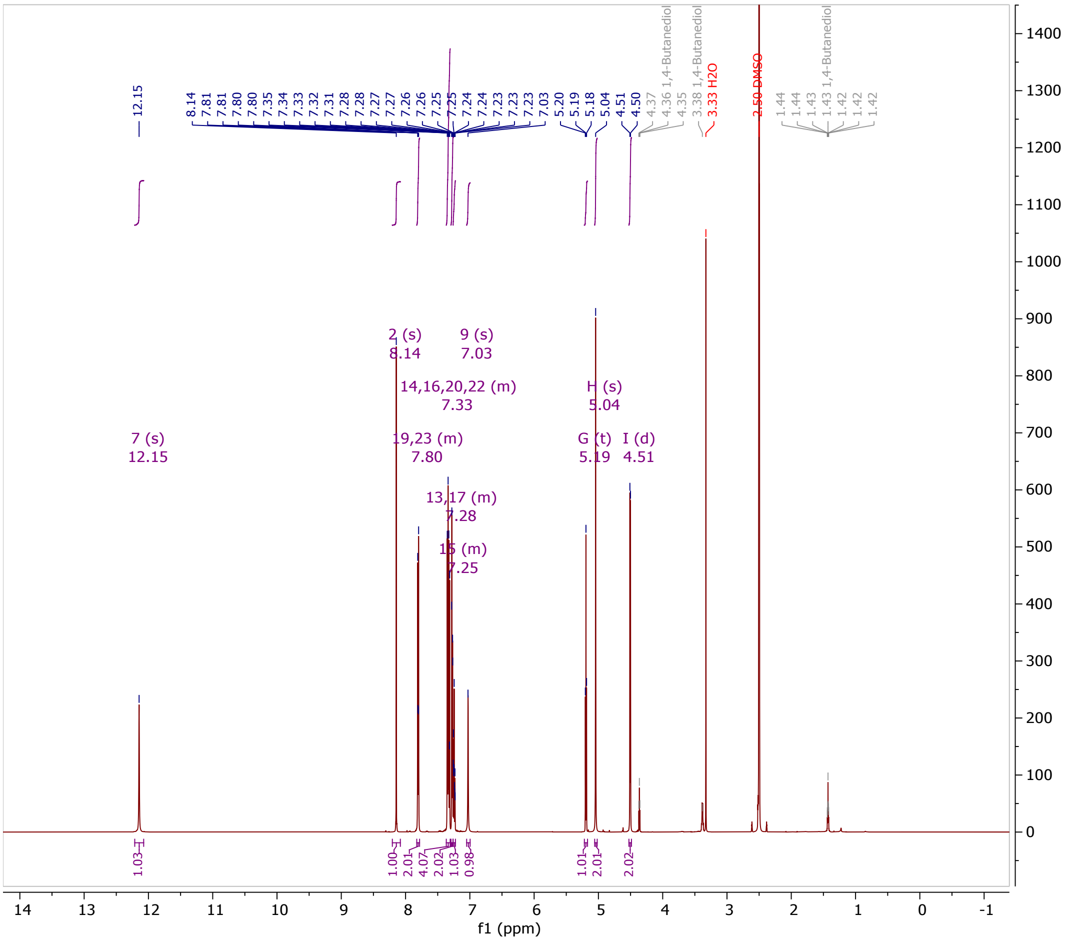

	Parameter	Value
1	Title	TIA05-046-3.1.fid
2	Origin	Bruker BioSpin GmbH
3	Owner	nmrsu
4	Instrument	spect
5	Solvent	DMSO
6	Temperature	298.0
7	Pulse Sequence	zg30
8	Experiment	1D
9	Probe	Z117768_0061 (CP TCI 600S3 H-C/ N-D-05 Z)
10	Number of Scans	16
11	Receiver Gain	8.0
12	Relaxation Delay	2.0000
13	Pulse Width	8.0000
14	Acquisition Time	2.7263
15	Acquisition Date	2020-11-15T08:51:49
16	Modification Date	2020-11-15T08:51:49
17	Spectrometer Frequency	600.18
18	Spectral Width	12019.2
19	Lowest Frequency	-2308.3
20	Nucleus	1H
21	Acquired Size	32768
22	Spectral Size	131072
23	Digital Resolution	0.09

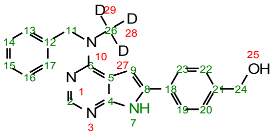

Figure S45. ^{1}H NMR (600 MHz, DMSO-d_6) of compound **21**.

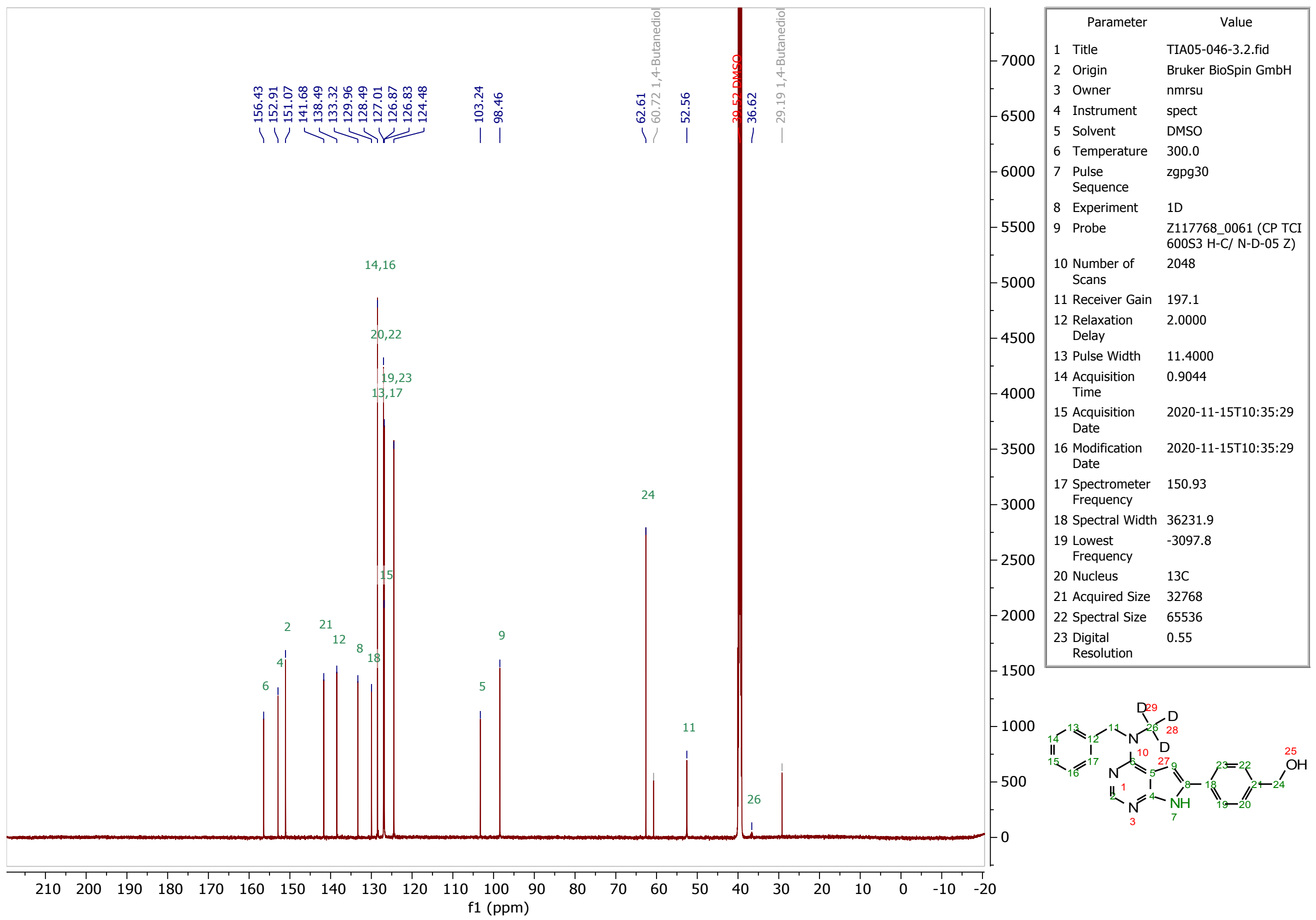

Figure S46. ^{13}C NMR (150 MHz, DMSO-d_6) of compound **21.**

Compound 22

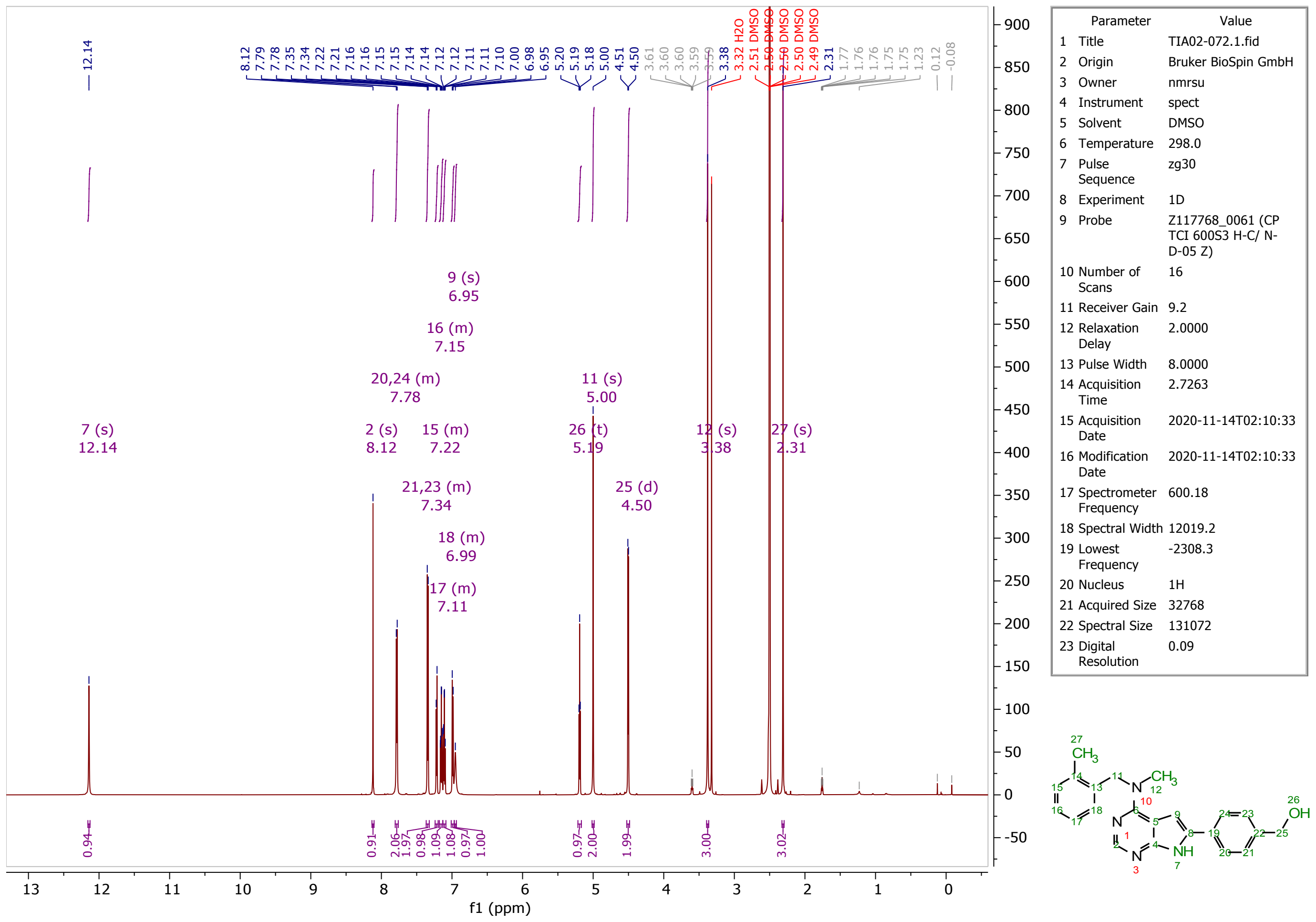

	Parameter	Value
1	Title	TIA02-072.1.fid
2	Origin	Bruker BioSpin GmbH
3	Owner	nmrsu
4	Instrument	spect
5	Solvent	DMSO
6	Temperature	298.0
7	Pulse Sequence	zg30
8	Experiment	1D
9	Probe	Z117768_0061 (CP TCI 600S3 H-C/ N-D-05 Z)
10	Number of Scans	16
11	Receiver Gain	9.2
12	Relaxation Delay	2.0000
13	Pulse Width	8.0000
14	Acquisition Time	2.7263
15	Acquisition Date	2020-11-14T02:10:33
16	Modification Date	2020-11-14T02:10:33
17	Spectrometer Frequency	600.18
18	Spectral Width	12019.2
19	Lowest Frequency	-2308.3
20	Nucleus	1H
21	Acquired Size	32768
22	Spectral Size	131072
23	Digital Resolution	0.09

Figure S47. ^{1}H NMR (600 MHz, DMSO-d_6) of compound **22**.

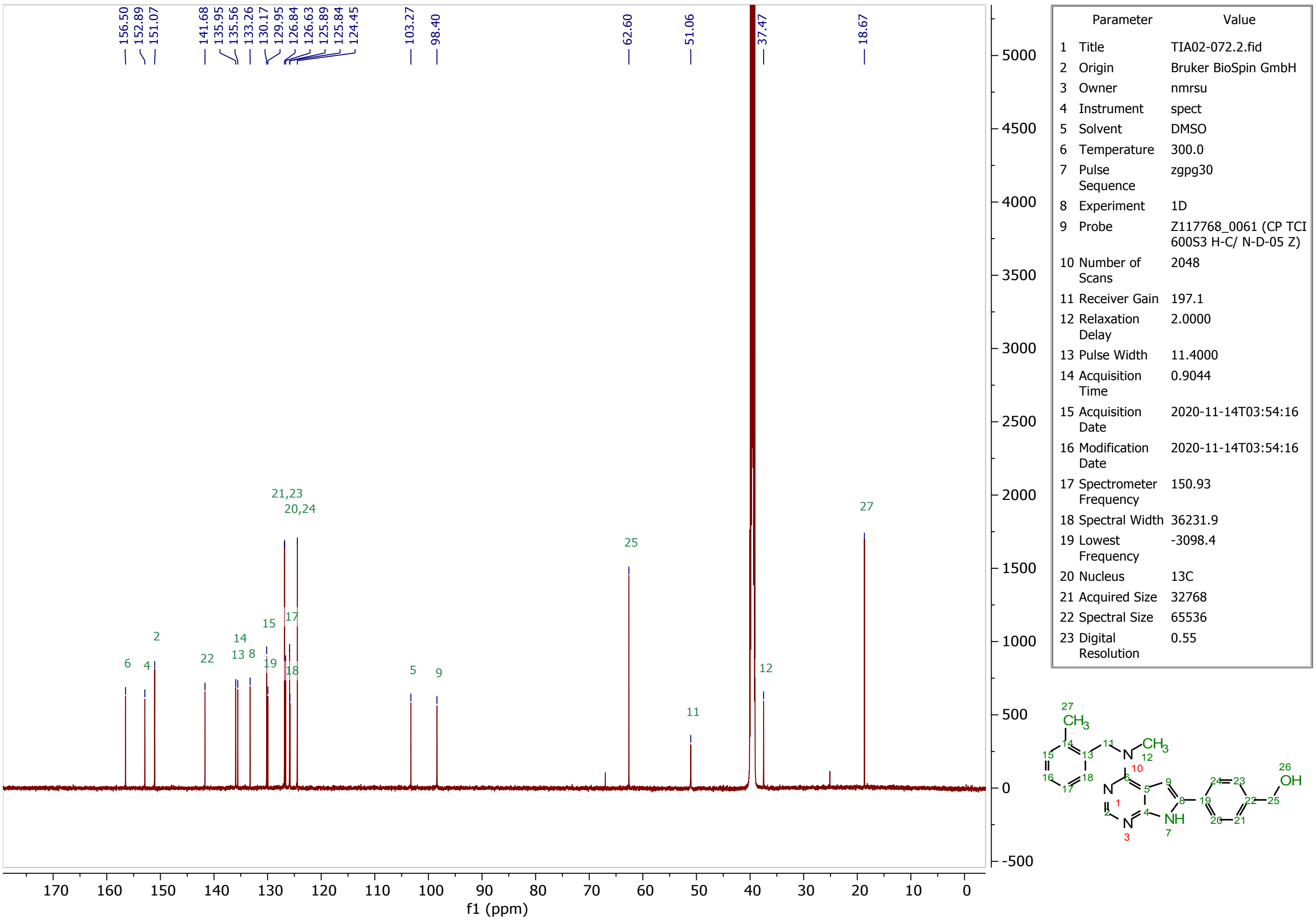

Figure S48. ^{13}C NMR (150 MHz, DMSO-d_6) of compound **22.**

Compound 23

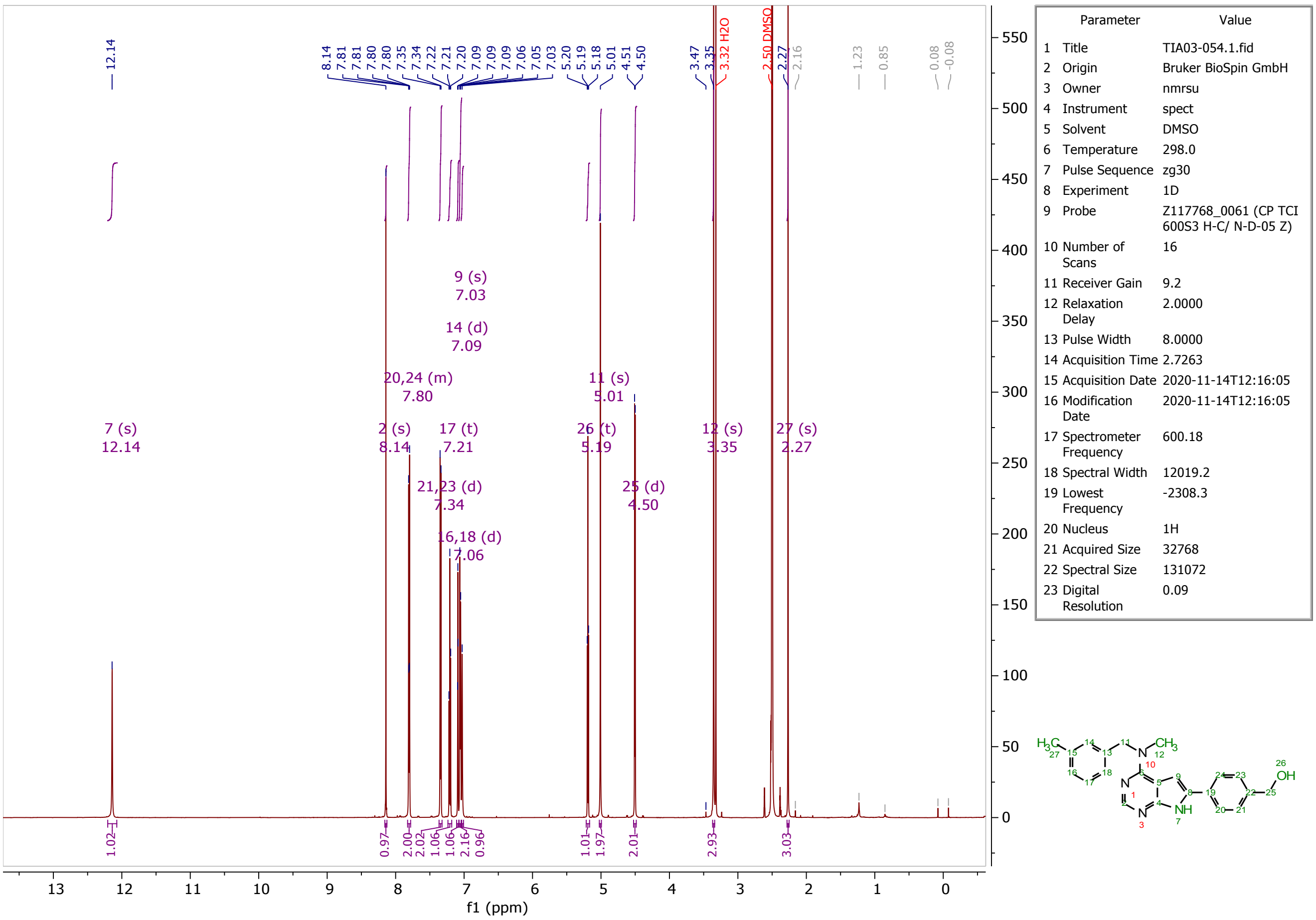

Parameter		Value
1	Title	TIA03-054.1.fid
2	Origin	Bruker BioSpin GmbH
3	Owner	nmrsu
4	Instrument	spect
5	Solvent	DMSO
6	Temperature	298.0
7	Pulse Sequence	zg30
8	Experiment	1D
9	Probe	Z117768_0061 (CP TCI 600S3 H-C/ N-D-05 Z)
10	Number of Scans	16
11	Receiver Gain	9.2
12	Relaxation Delay	2.0000
13	Pulse Width	8.0000
14	Acquisition Time	2.7263
15	Acquisition Date	2020-11-14T12:16:05
16	Modification Date	2020-11-14T12:16:05
17	Spectrometer Frequency	600.18
18	Spectral Width	12019.2
19	Lowest Frequency	-2308.3
20	Nucleus	1H
21	Acquired Size	32768
22	Spectral Size	131072
23	Digital Resolution	0.09

Figure S49. ^{1}H NMR (600 MHz, DMSO-d_6) of compound **20**.

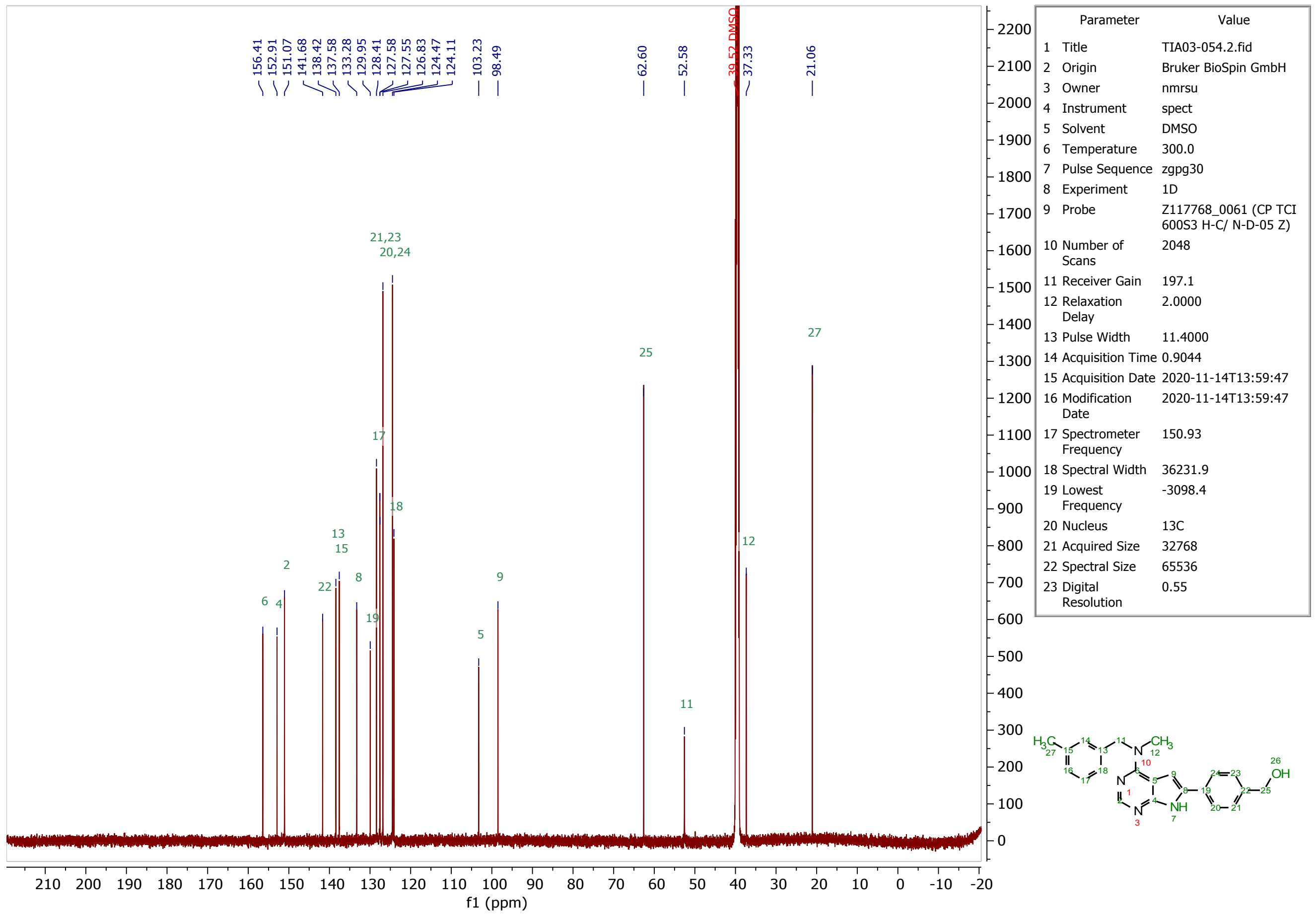

Figure S50. ^{13}C NMR (150 MHz, DMSO-d_6) of compound **22.**

Compound 24

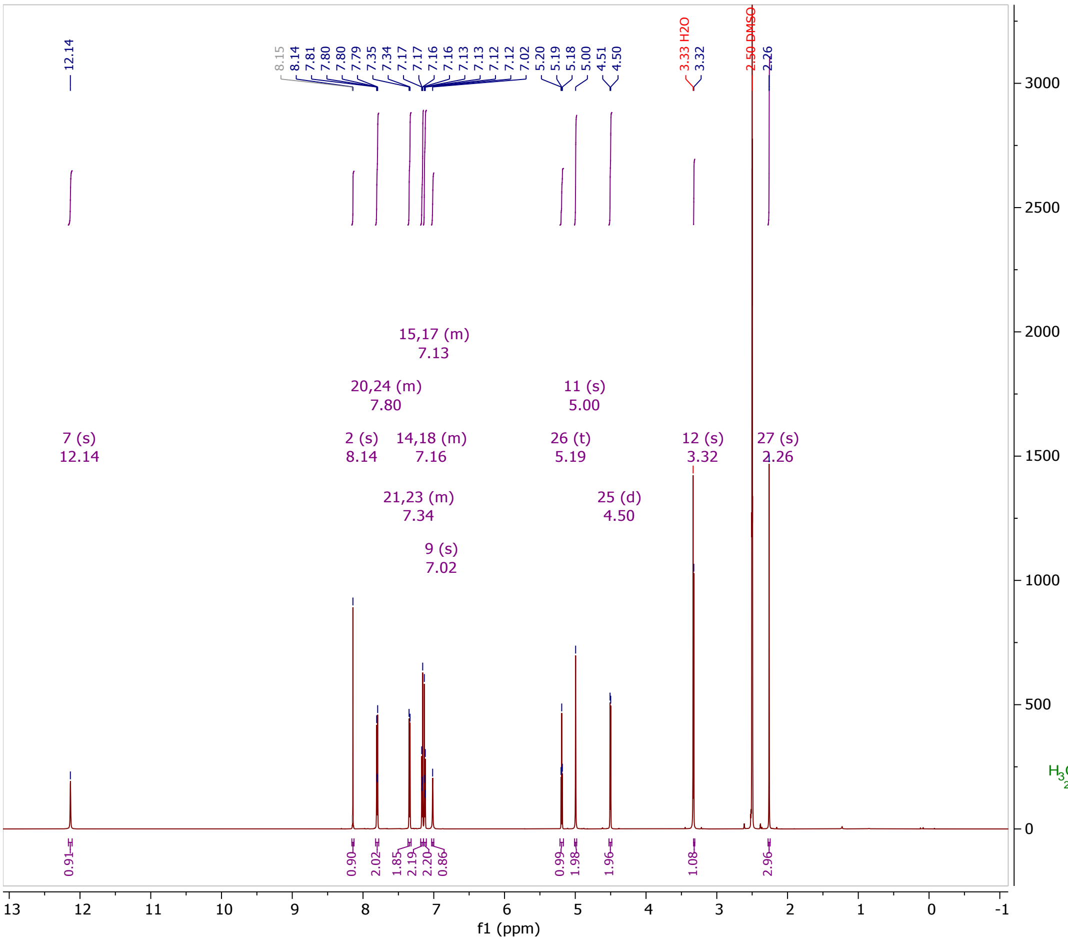

	Parameter	Value
1	Title	TIA02-074.1.fid
2	Origin	Bruker BioSpin GmbH
3	Owner	nmrsu
4	Instrument	spect
5	Solvent	DMSO
6	Temperature	298.0
7	Pulse Sequence	zg30
8	Experiment	1D
9	Probe	Z117768_0061 (CP TCI 600S3 H-C/ N-D-05 Z)
10	Number of Scans	16
11	Receiver Gain	9.2
12	Relaxation Delay	2.0000
13	Pulse Width	8.0000
14	Acquisition Time	2.7263
15	Acquisition Date	2020-11-14T05:32:30
16	Modification Date	2020-11-14T05:32:30
17	Spectrometer Frequency	600.18
18	Spectral Width	12019.2
19	Lowest Frequency	-2308.3
20	Nucleus	1H
21	Acquired Size	32768
22	Spectral Size	131072
23	Digital Resolution	0.09

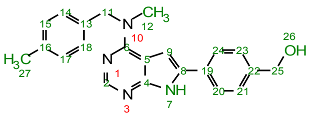

Figure S51. ^{1}H NMR (600 MHz, DMSO-d_6) of compound **24**.

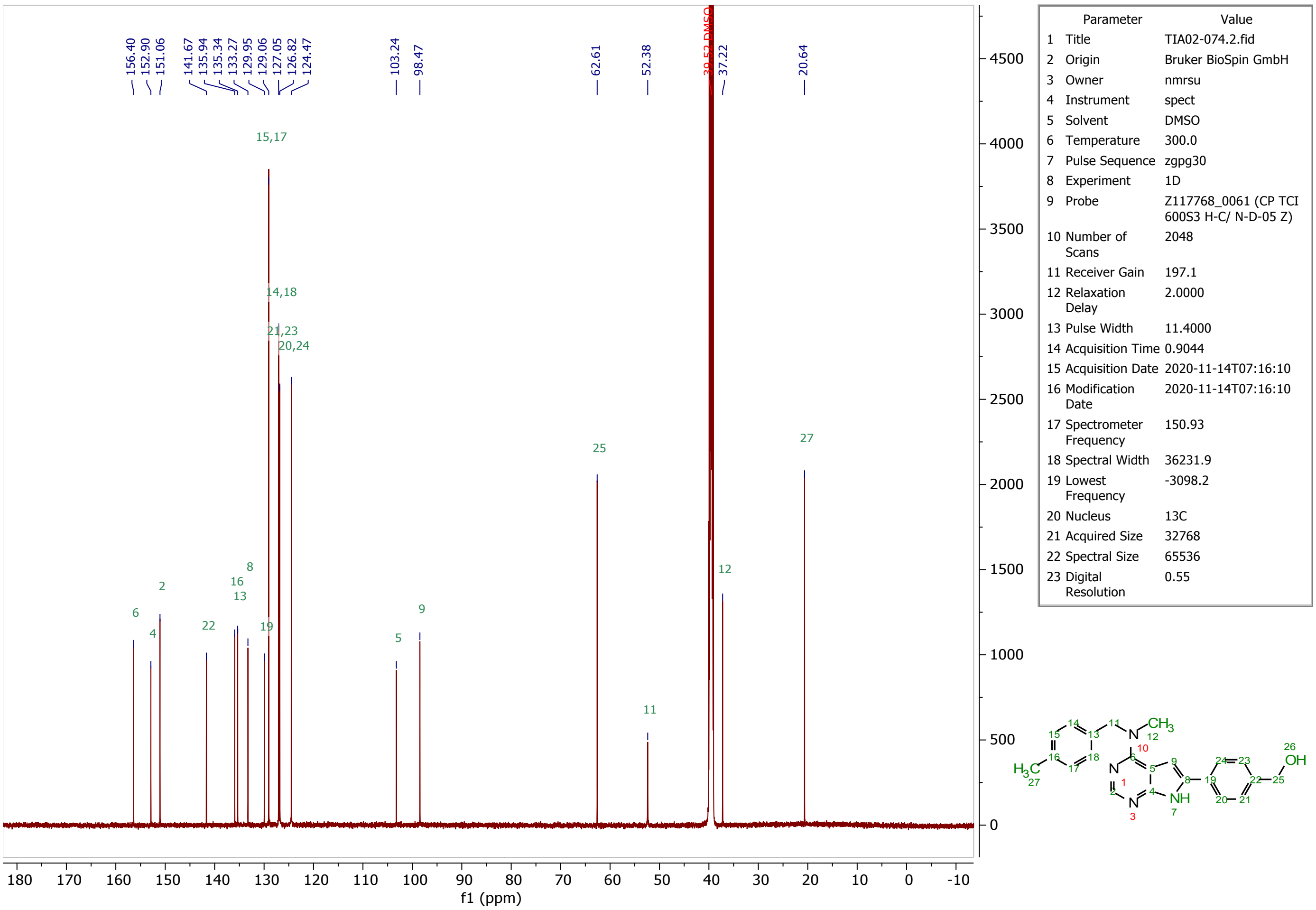

Figure S52. ^{13}C NMR (150 MHz, DMSO-d_6) of compound **24.**

Compound 25

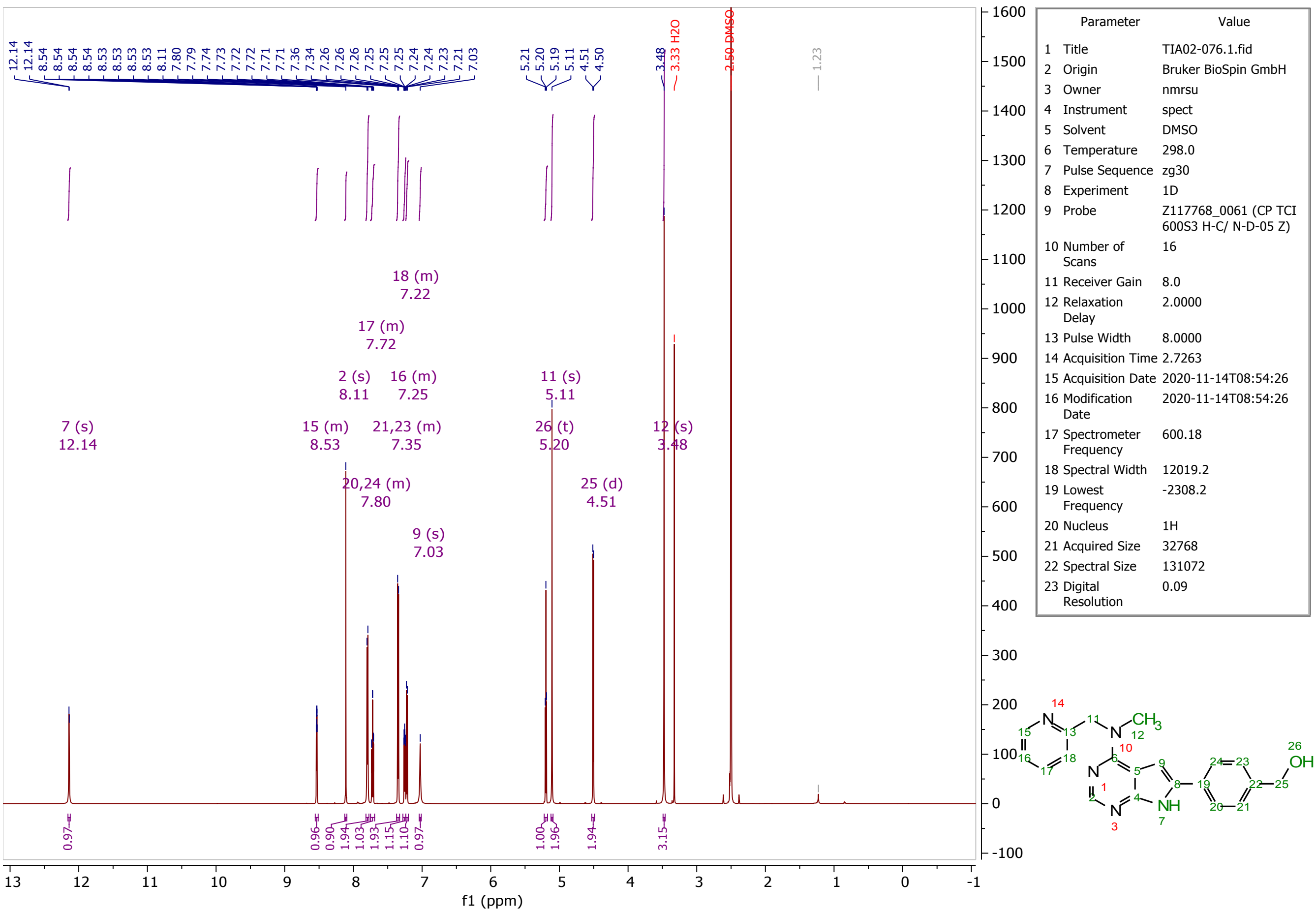

Figure S53. ^{1}H NMR (600 MHz, DMSO-d_6) of compound **25**.

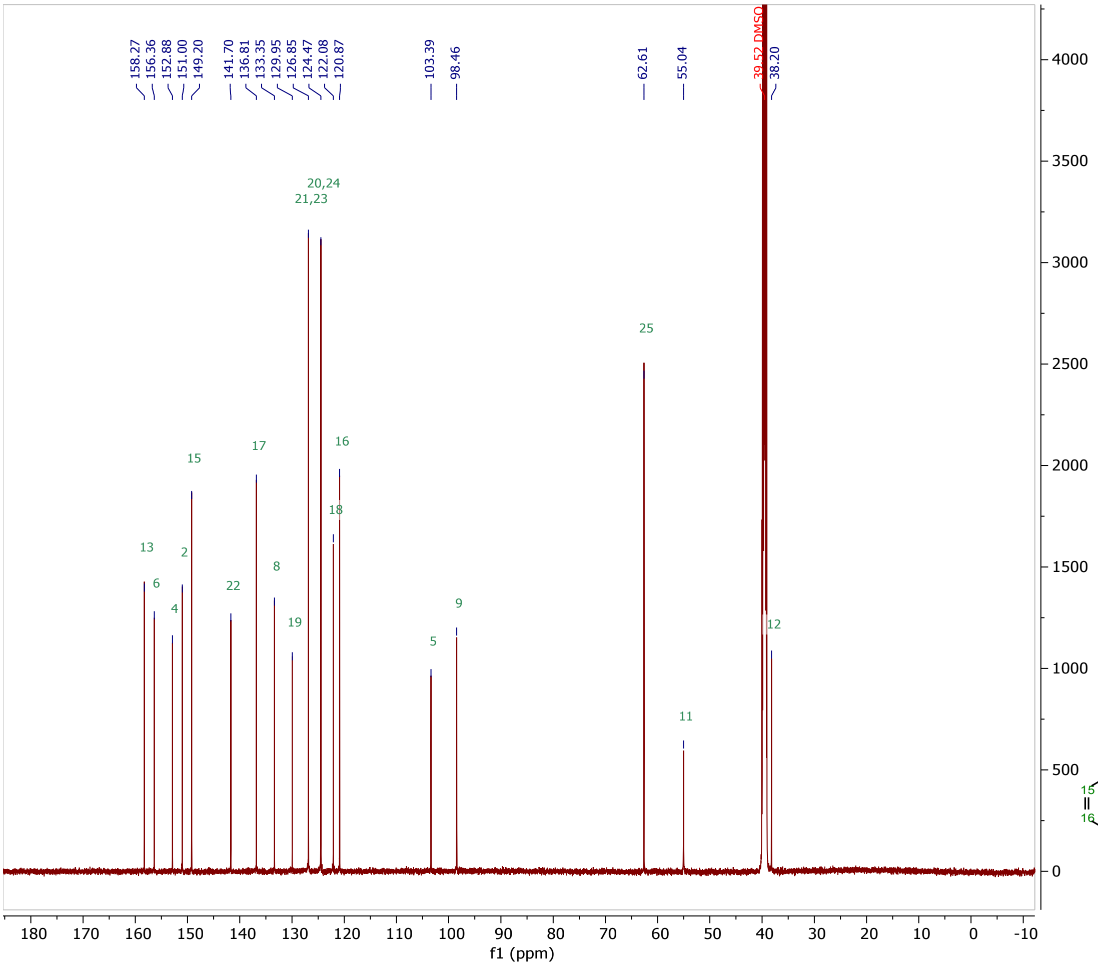

Parameter		Value
1	Title	TIA02-076.2.fid
2	Origin	Bruker BioSpin GmbH
3	Owner	nmrsu
4	Instrument	spect
5	Solvent	DMSO
6	Temperature	300.0
7	Pulse Sequence	zgpg30
8	Experiment	1D
9	Probe	Z117768_0061 (CP TCI 600S3 H-C/ N-D-05 Z)
10	Number of Scans	2048
11	Receiver Gain	197.1
12	Relaxation Delay	2.0000
13	Pulse Width	11.4000
14	Acquisition Time	0.9044
15	Acquisition Date	2020-11-14T10:38:05
16	Modification Date	2020-11-14T10:38:05
17	Spectrometer Frequency	150.93
18	Spectral Width	36231.9
19	Lowest Frequency	-3098.1
20	Nucleus	13C
21	Acquired Size	32768
22	Spectral Size	65536
23	Digital Resolution	0.55

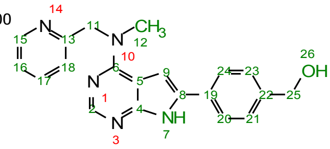

Figure S54. ^{13}C NMR (150 MHz, DMSO-d_6) of compound **25.**

Compound 26

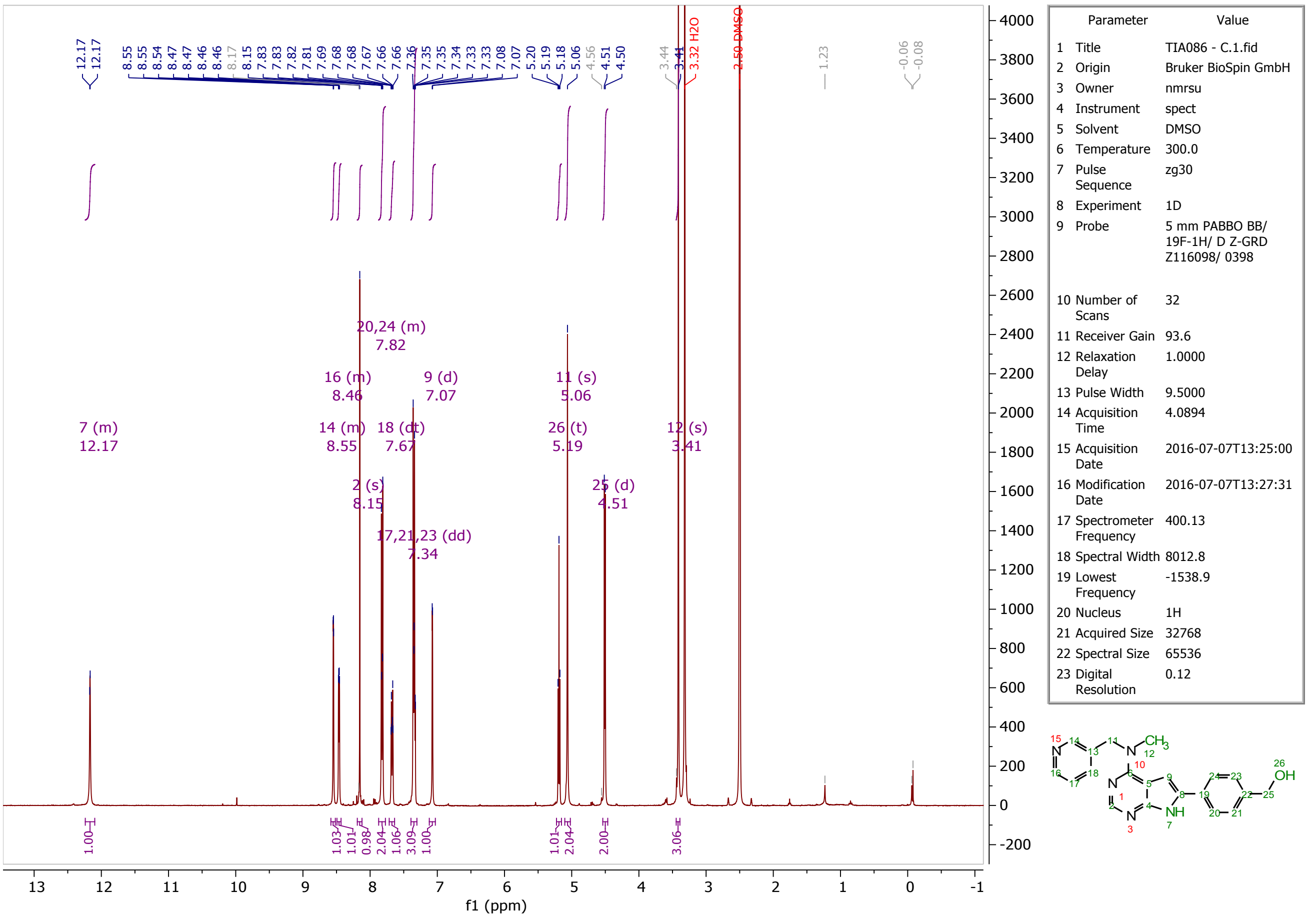

Figure S55. ^{1}H NMR (400 MHz, DMSO-d_6) of compound **26**.

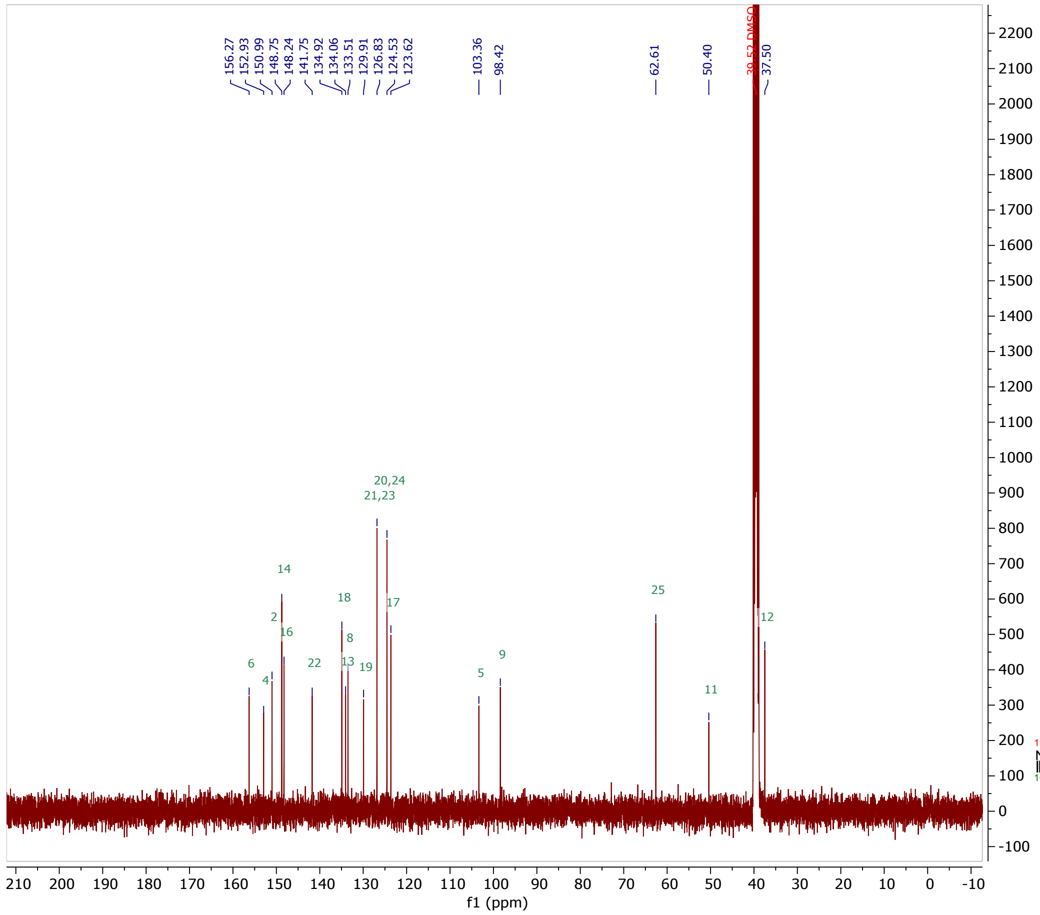

Parameter		Value
1	Title	TIA086 - C.3.fid
2	Origin	Bruker BioSpin GmbH
3	Owner	nmrsu
4	Instrument	spect
5	Solvent	DMSO
6	Temperature	300.0
7	Pulse Sequence	zgpg30
8	Experiment	1D
9	Probe	5 mm PABBO BB/ 19F-1H/ D Z-GRD Z116098/ 0398
10	Number of Scans	2048
11	Receiver Gain	209.8
12	Relaxation Delay	2.0000
13	Pulse Width	9.5000
14	Acquisition Time	1.3631
15	Acquisition Date	2016-07-07T15:36:00
16	Modification Date	2016-07-07T15:36:48
17	Spectrometer Frequency	100.62
18	Spectral Width	24038.5
19	Lowest Frequency	-2007.0
20	Nucleus	13C
21	Acquired Size	32768
22	Spectral Size	65536
23	Digital Resolution	0.37

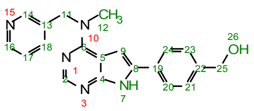

Figure S56. ^{13}C NMR (100 MHz, DMSO-d_6) of compound **26.**

Compound 27

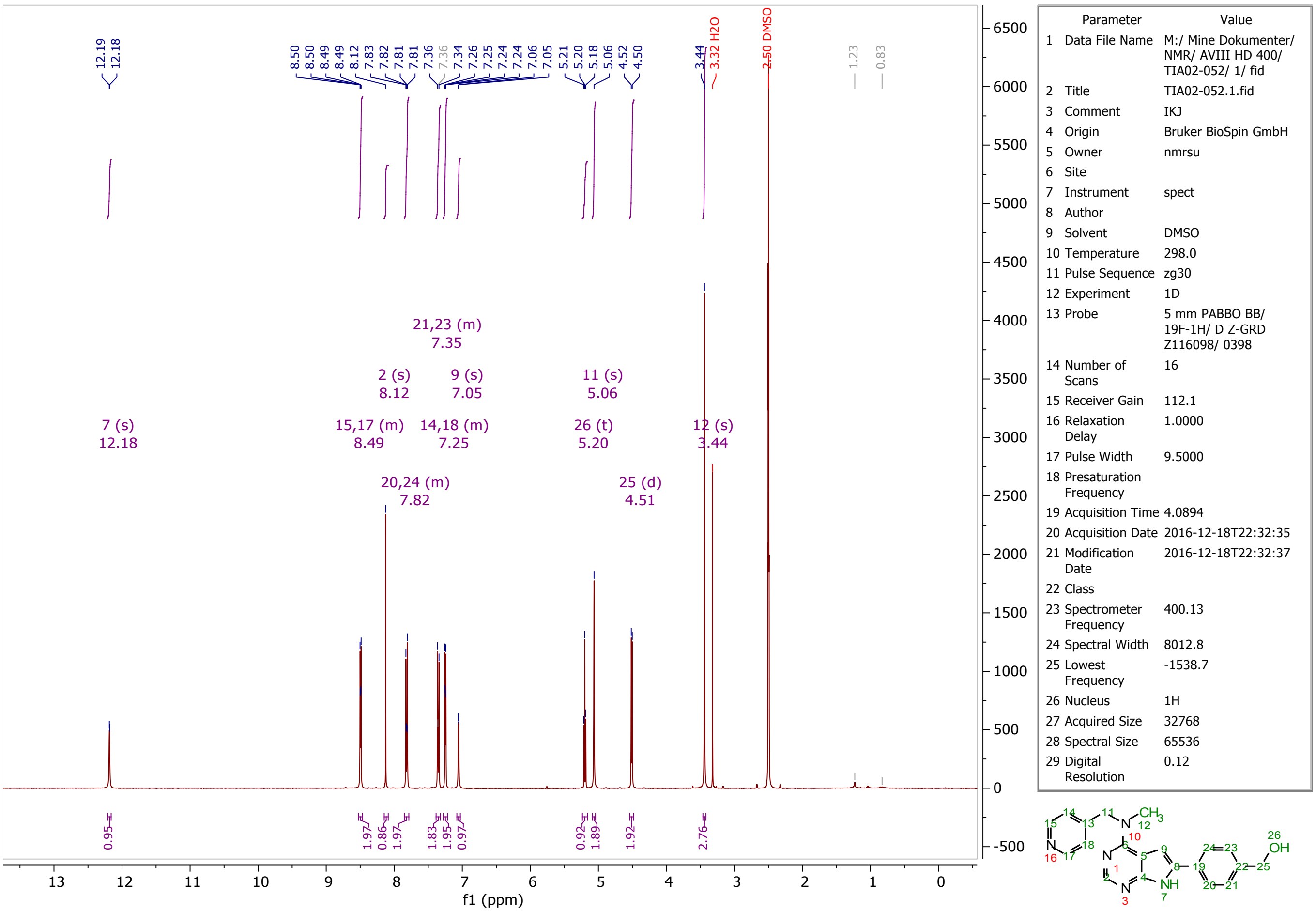

Figure S57. ^{1}H NMR (400 MHz, DMSO-d_6) of compound **27**.

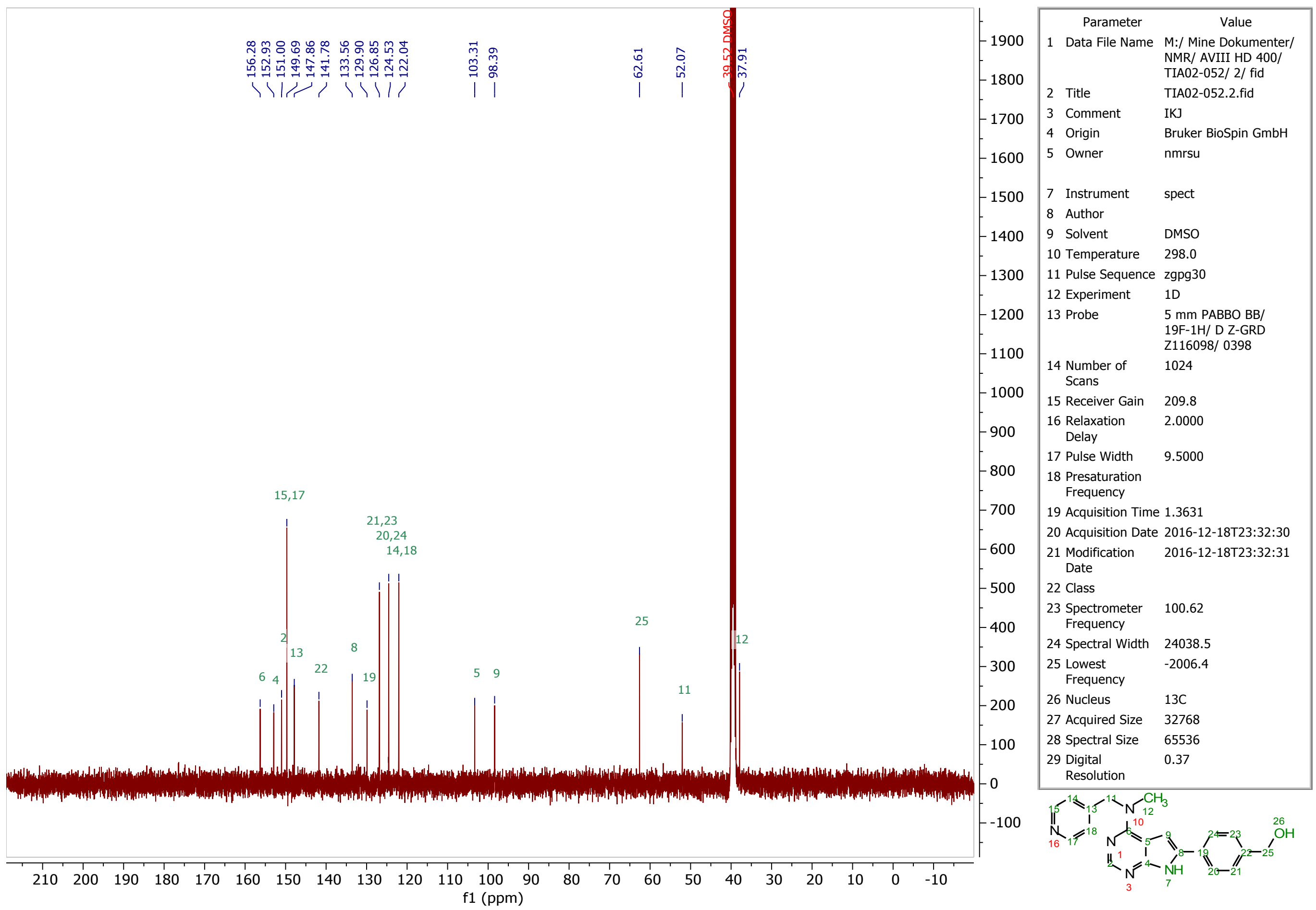

Figure S58. ^{13}C NMR (100 MHz, DMSO-d_6) of compound **27.**

Compound 28

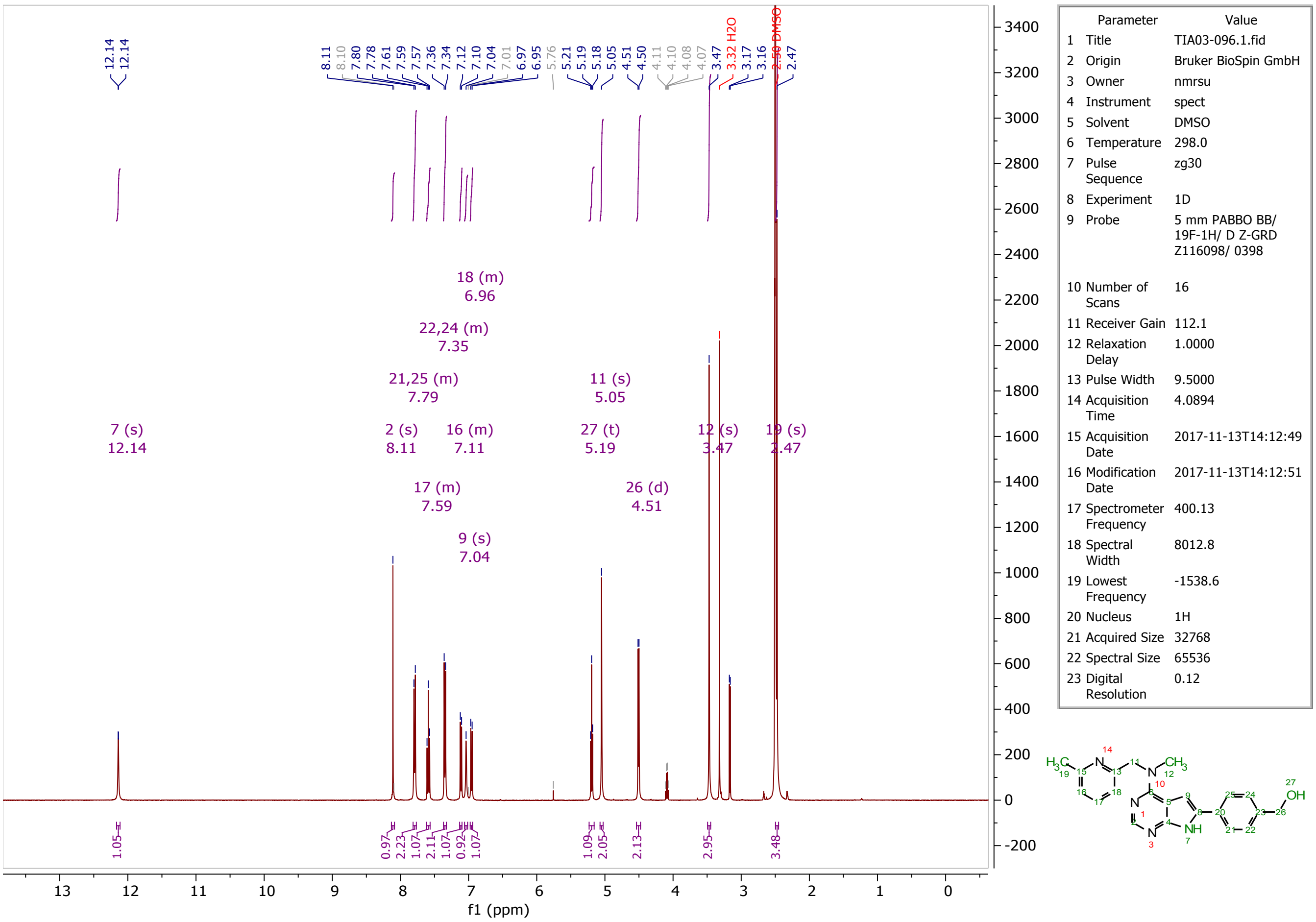

Figure S59. ^{1}H NMR (400 MHz, DMSO-d_6) of compound **28**.

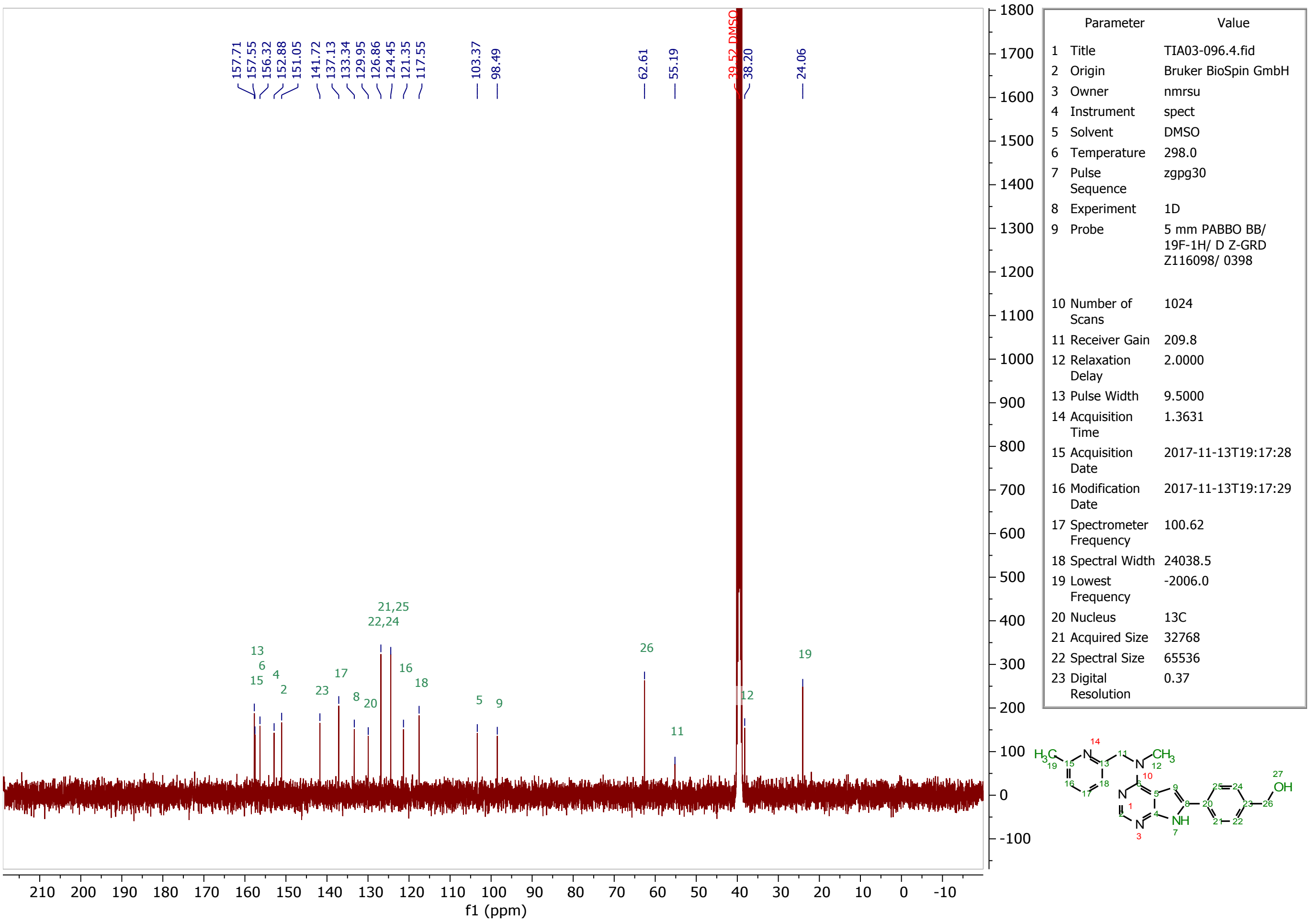

Figure S60. ^{13}C NMR (100 MHz, DMSO-d_6) of compound **28.**

Compound 29

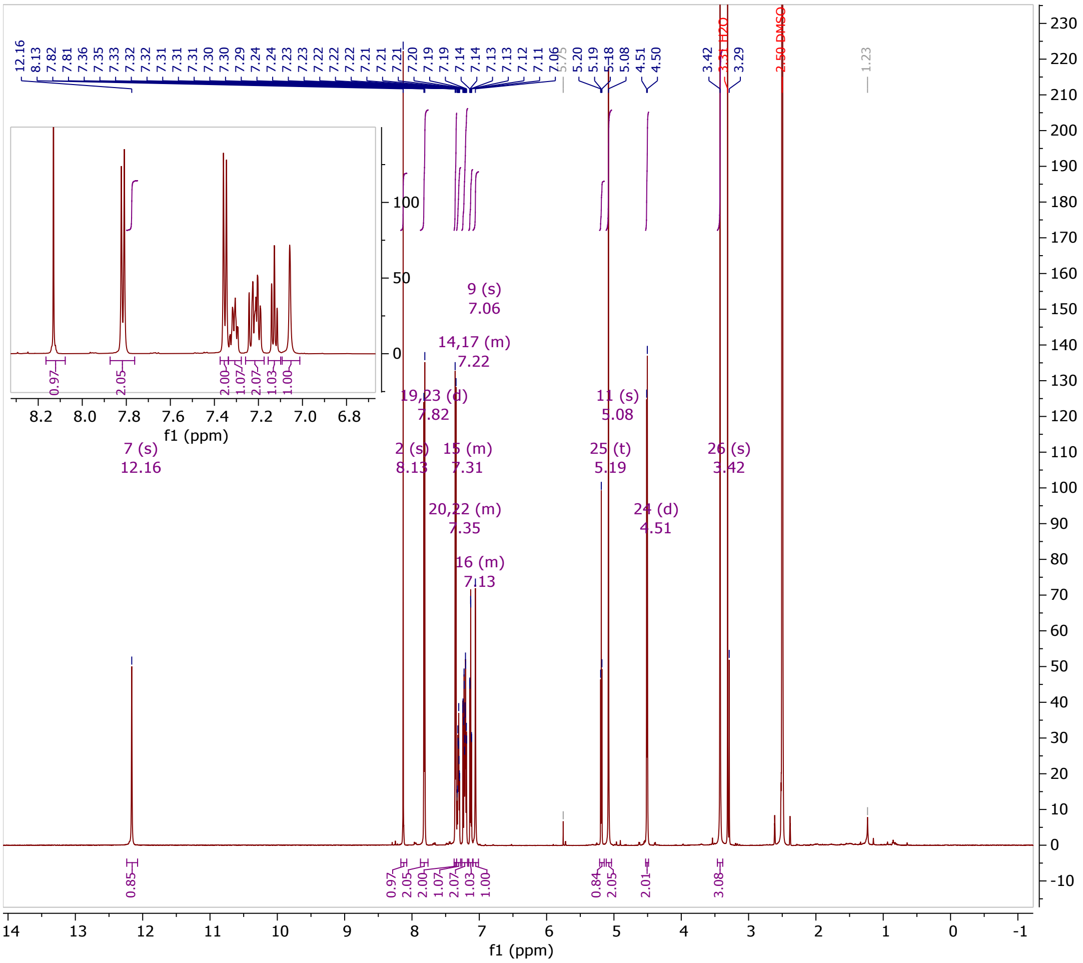

	Parameter	Value
1	Title	TIA05-086-1.1.fid
2	Origin	Bruker BioSpin GmbH
3	Owner	nmrsu
4	Instrument	spect
5	Solvent	DMSO
6	Temperature	300.0
7	Pulse Sequence	zg30
8	Experiment	1D
9	Probe	Z117768_0061 (CP TCI 600S3 H-C/ N-D-05 Z)
10	Number of Scans	16
11	Receiver Gain	10.1
12	Relaxation Delay	2.0000
13	Pulse Width	8.0000
14	Acquisition Time	2.7263
15	Acquisition Date	2019-11-14T09:21:42
16	Modification Date	2019-11-14T09:21:42
17	Spectrometer Frequency	600.18
18	Spectral Width	12019.2
19	Lowest Frequency	-2308.1
20	Nucleus	1H
21	Acquired Size	32768
22	Spectral Size	131072
23	Digital Resolution	0.09

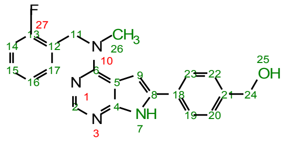

Figure S61. ^{1}H NMR (600 MHz, DMSO-d_6) of compound **29**.

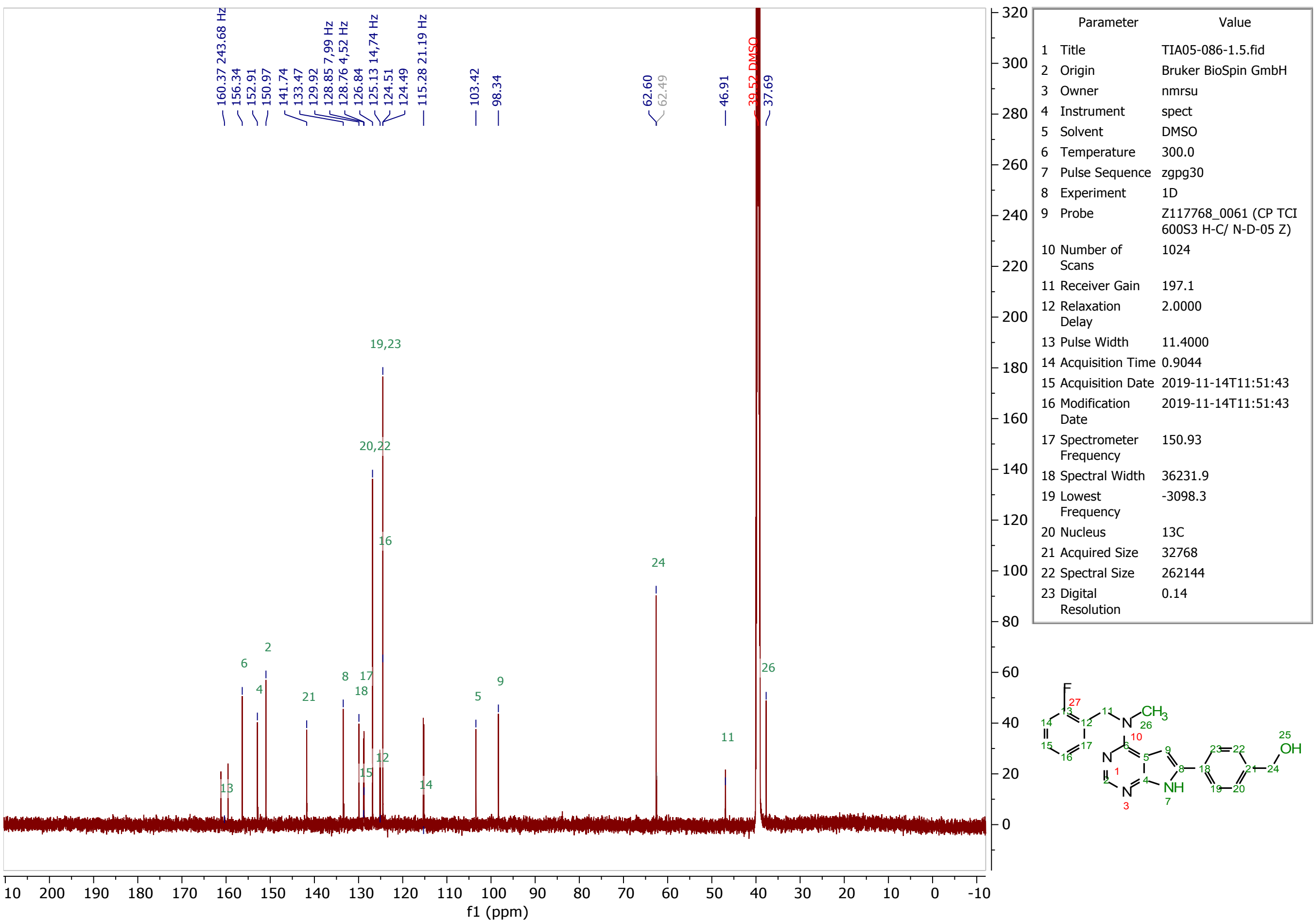

Figure S62. ^{13}C NMR (150 MHz, DMSO-d_6) of compound **29.**

Compound 30

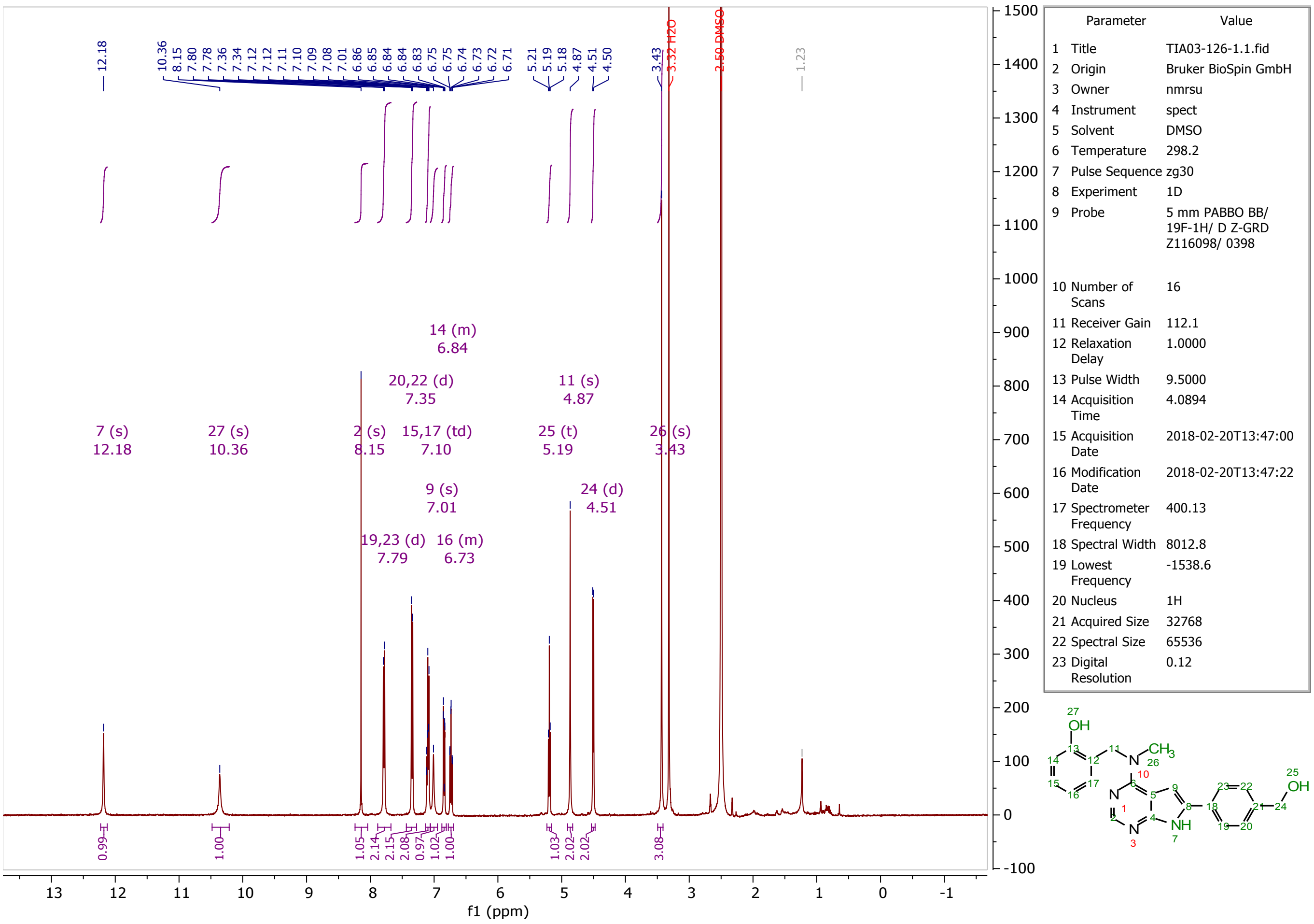

Figure S63. ^{1}H NMR (400 MHz, DMSO-d_6) of compound **30**.

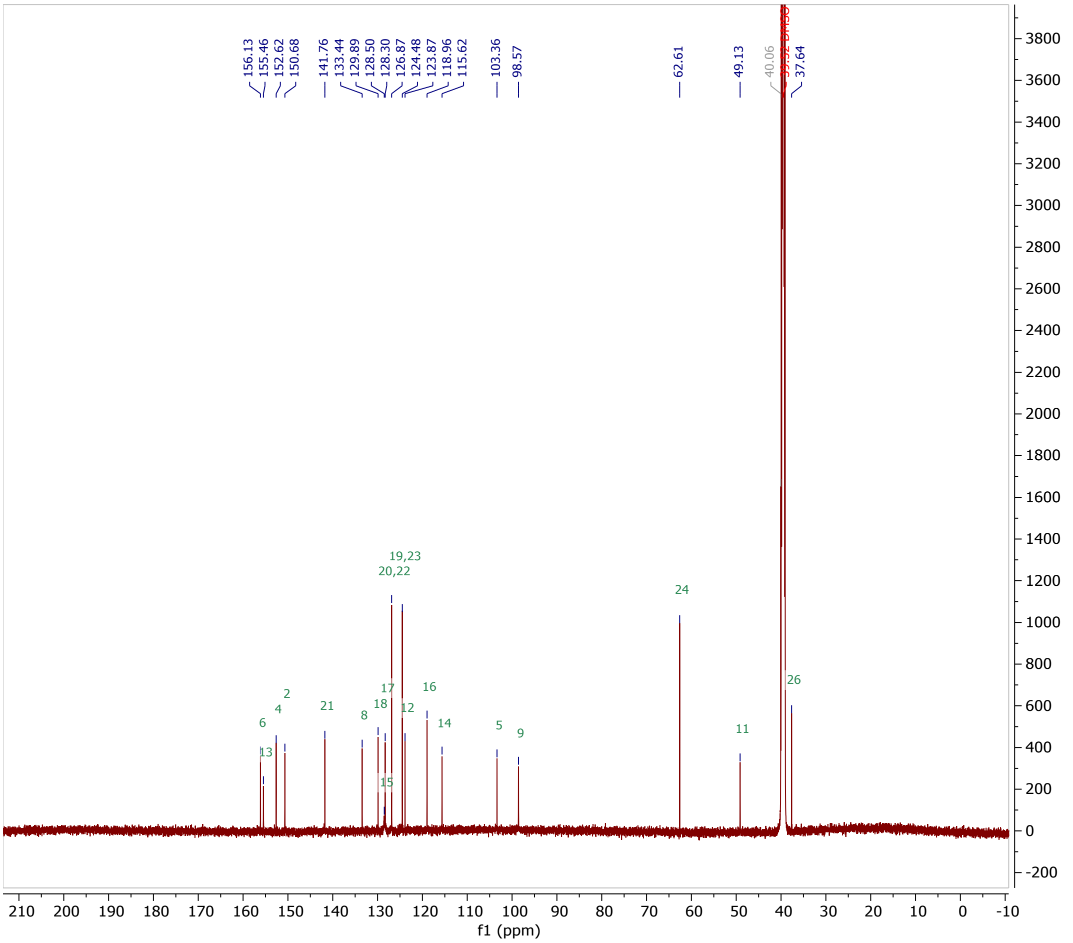

Parameter		Value
1	Title	TIA03-126.2.fid
2	Origin	Bruker BioSpin GmbH
3	Owner	nmrsu
4	Instrument	spect
5	Solvent	DMSO
6	Temperature	300.0
7	Pulse Sequence	zgpg30
8	Experiment	1D
9	Probe	Z117768_0061 (CP TCI 600S3 H-C/ N-D-05 Z)
10	Number of Scans	4096
11	Receiver Gain	197.1
12	Relaxation Delay	2.0000
13	Pulse Width	11.4000
14	Acquisition Time	0.9044
15	Acquisition Date	2021-01-13T02:26:39
16	Modification Date	2021-01-13T02:26:39
17	Spectrometer Frequency	150.93
18	Spectral Width	36231.9
19	Lowest Frequency	-3095.0
20	Nucleus	13C
21	Acquired Size	32768
22	Spectral Size	65536
23	Digital Resolution	0.55

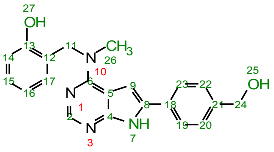

Figure S64. ^{13}C NMR (150 MHz, DMSO-d_6) of compound **30.**

Compound 31

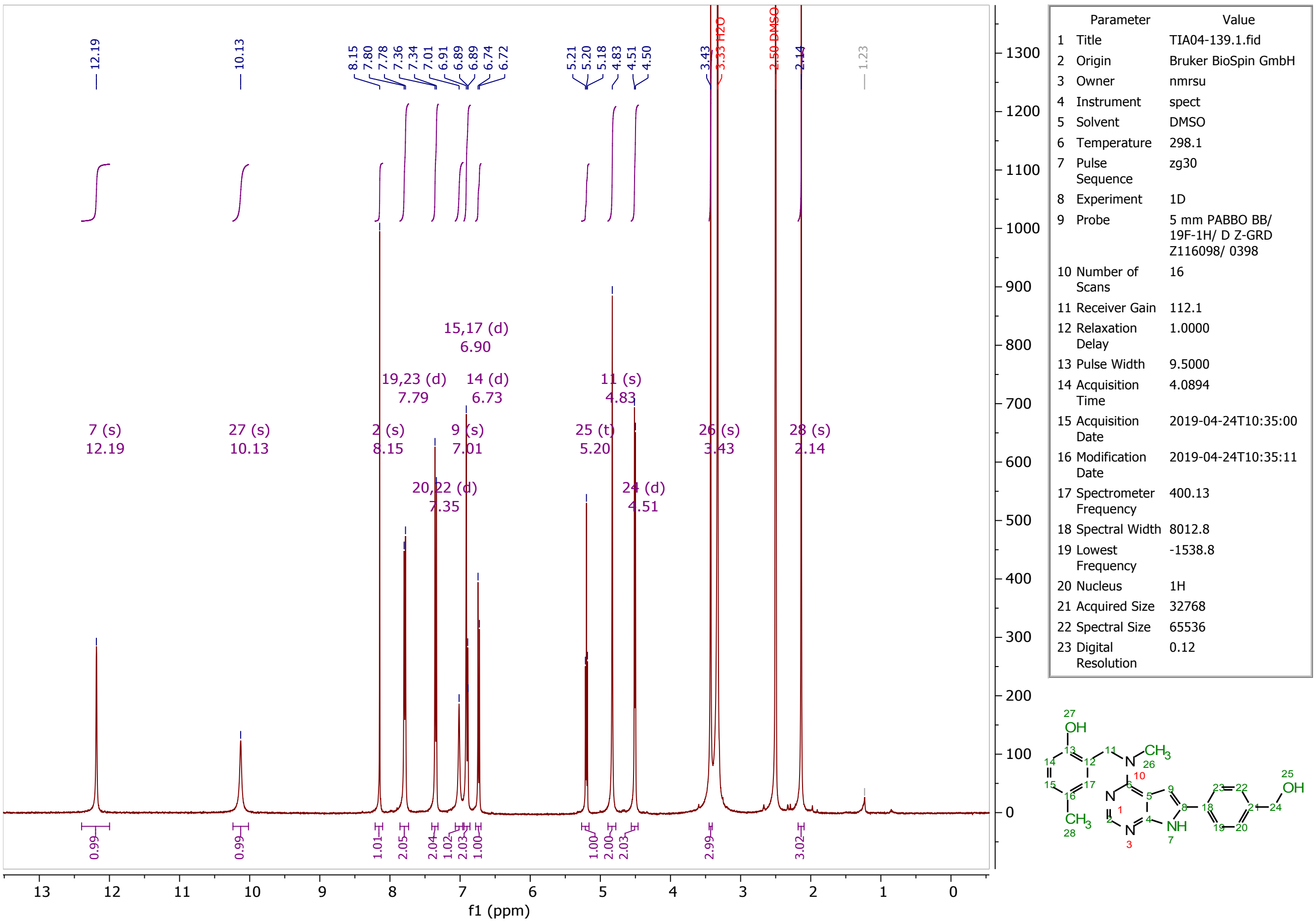

Figure S65. ^{1}H NMR (400 MHz, DMSO-d_6) of compound **31**.

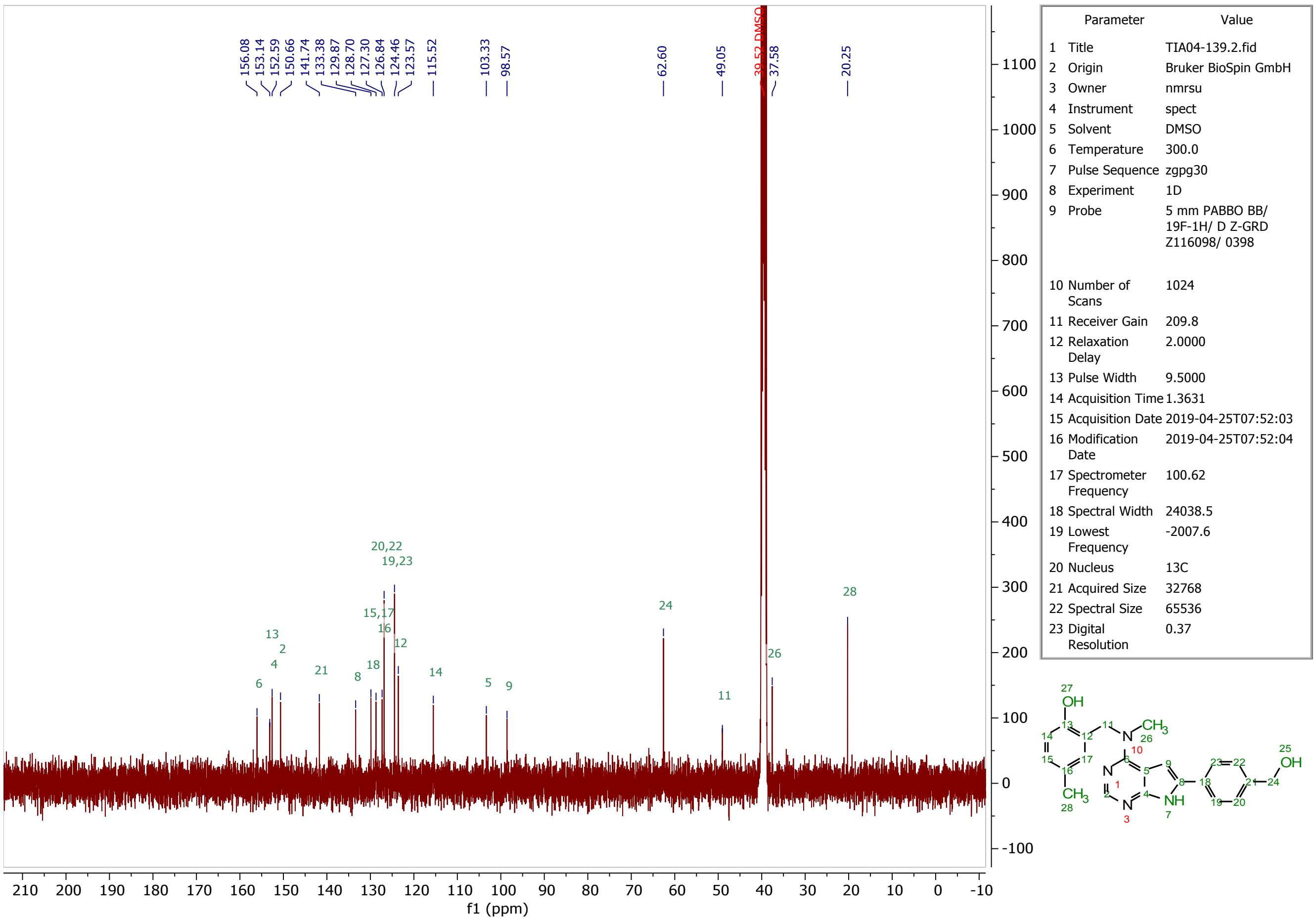

Figure S66. ^{13}C NMR (100 MHz, DMSO-d_6) of compound **31.**

Compound 32

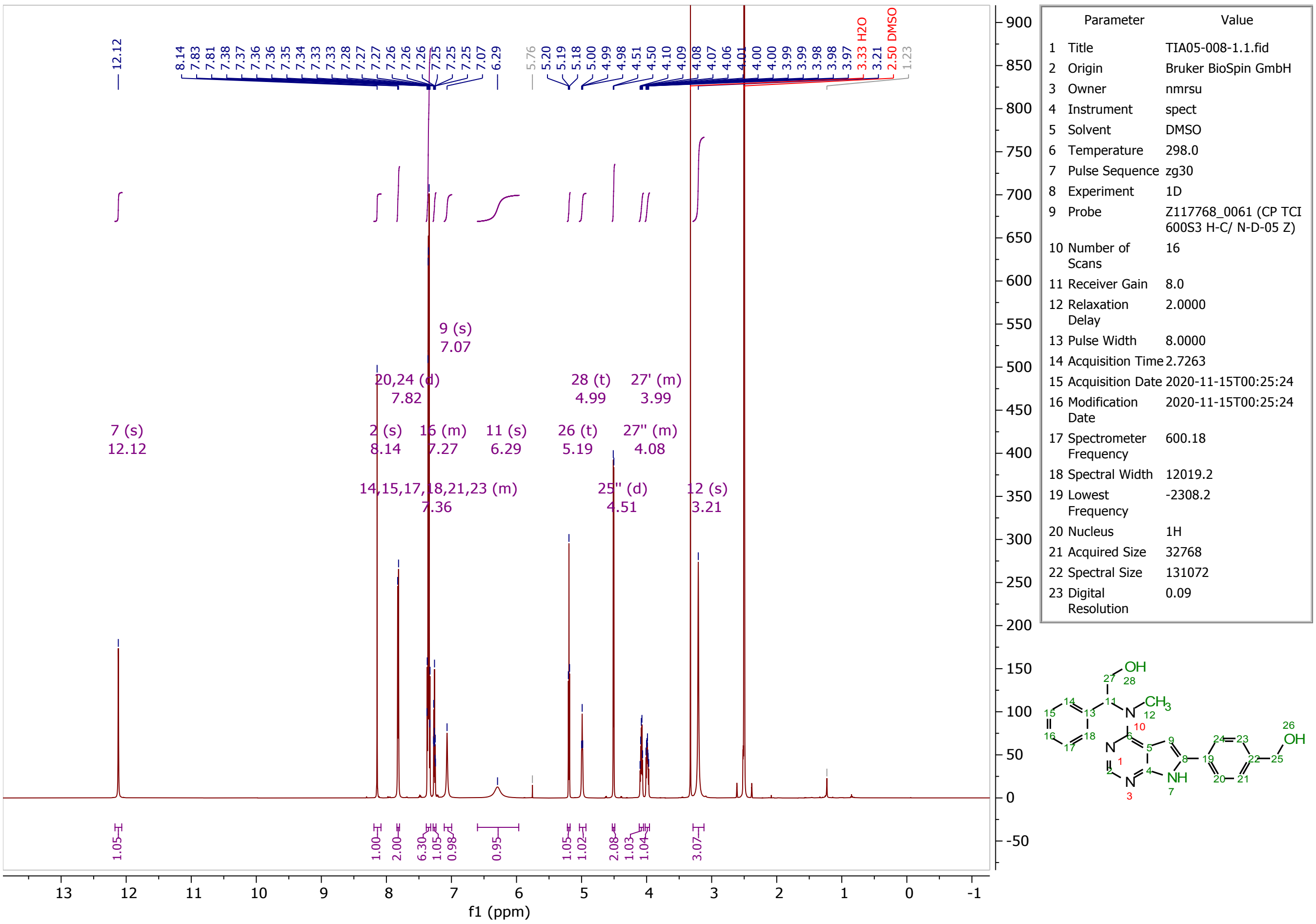

Figure S67. ^{1}H NMR (600 MHz, DMSO-d_6) of compound **32**.

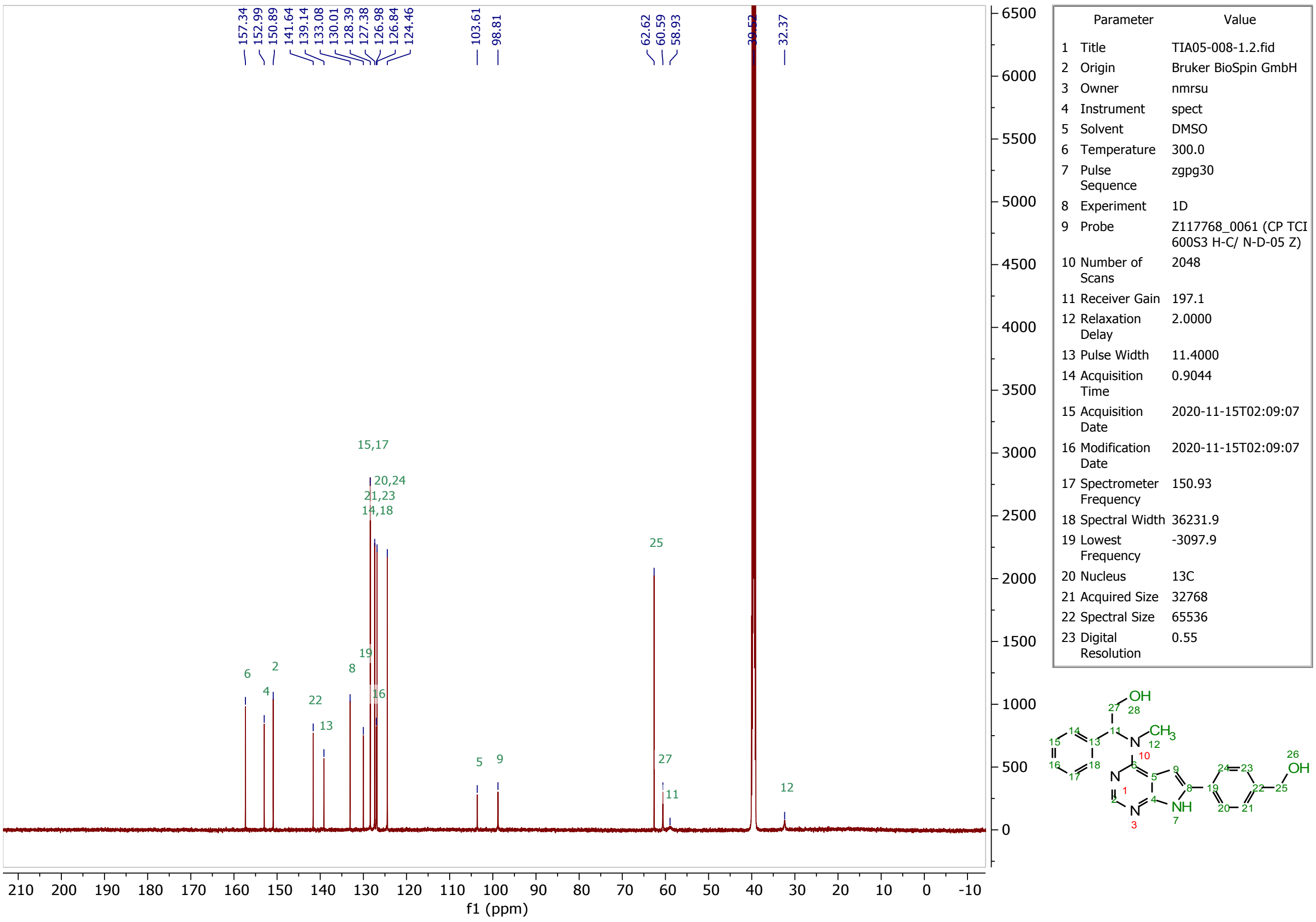

Figure S68. ^{13}C NMR (150 MHz, DMSO-d_6) of compound **32.**

Compound 33

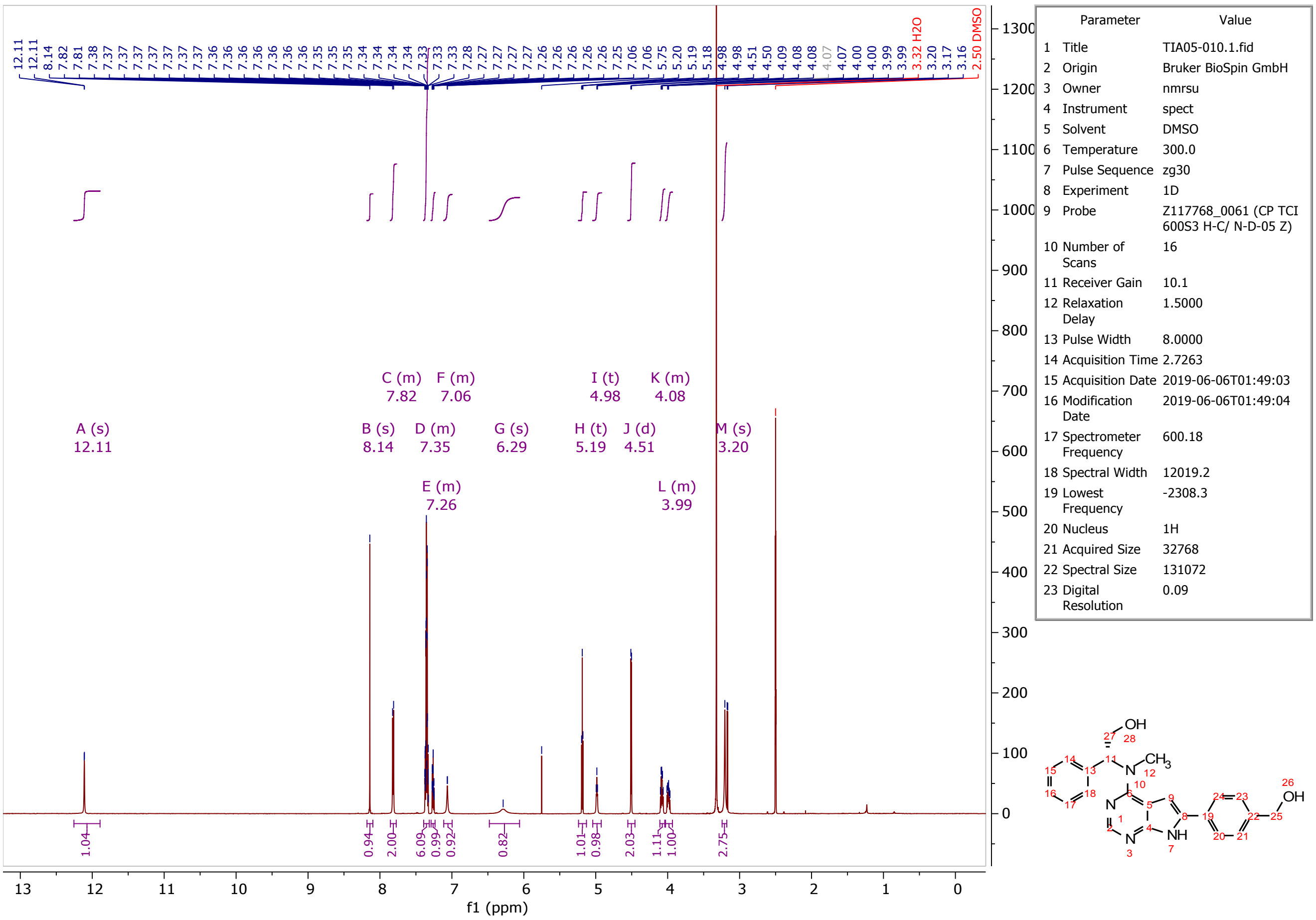

Figure S69. ^{1}H NMR (600 MHz, DMSO-d_6) of compound **33**.

Compound 35

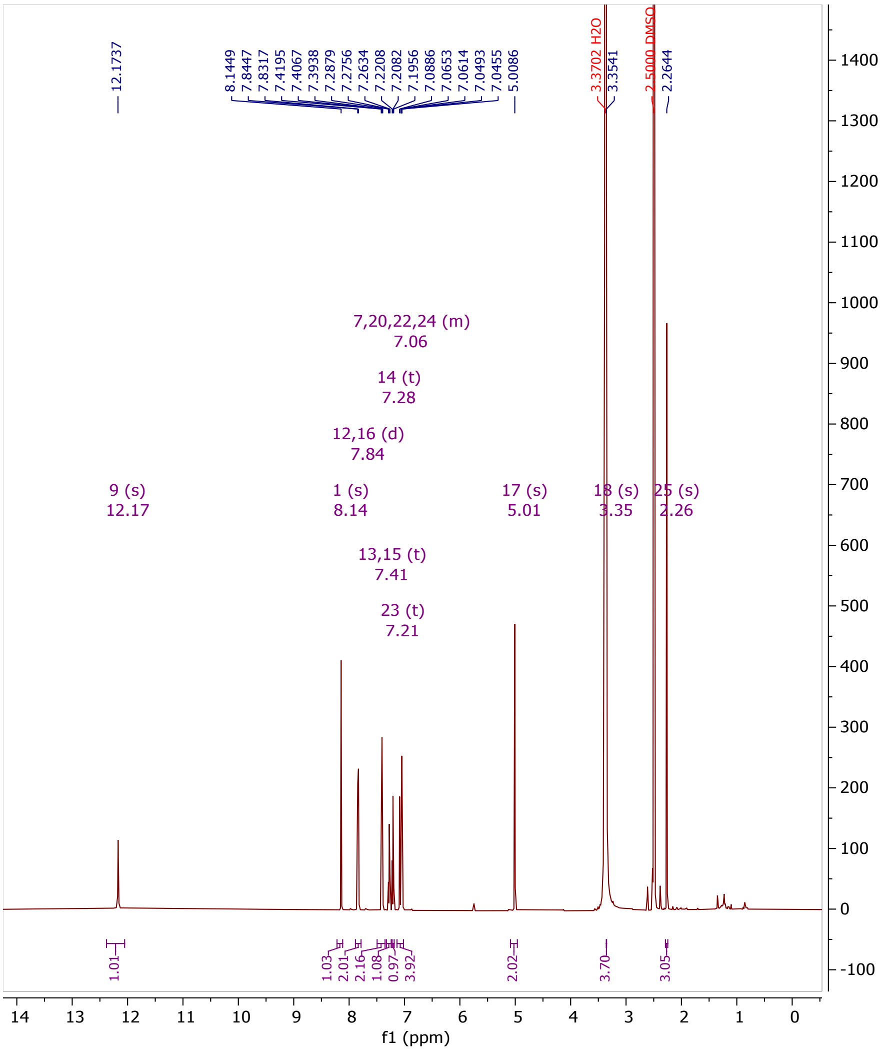

	Parameter	Value
1	Title	FAB01-92EK.1.fid
2	Origin	Bruker BioSpin GmbH
3	Owner	nmrsu
4	Instrument	spect
5	Solvent	DMSO
6	Temperature	298.0
7	Pulse Sequence	zg30
8	Experiment	1D
9	Probe	Z117768_0061 (CP TCI 600S3 H-C/ N-D-05 Z)
10	Number of Scans	64
11	Receiver Gain	8.0
12	Relaxation Delay	1.0000
13	Pulse Width	8.0000
14	Presaturation Frequency	
15	Acquisition Time	2.7263
16	Acquisition Date	2021-01-08T10:39:39
17	Modification Date	2021-01-08T10:39:40
18	Spectrometer Frequency	600.18
19	Spectral Width	12019.2
20	Lowest Frequency	-2357.5
21	Nucleus	1H
22	Acquired Size	32768
23	Spectral Size	131072
24	Digital Resolution	0.09

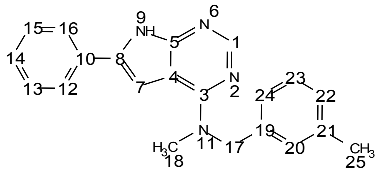

Figure S70. ^{1}H NMR (600 MHz, DMSO-d_6) of compound **35**.

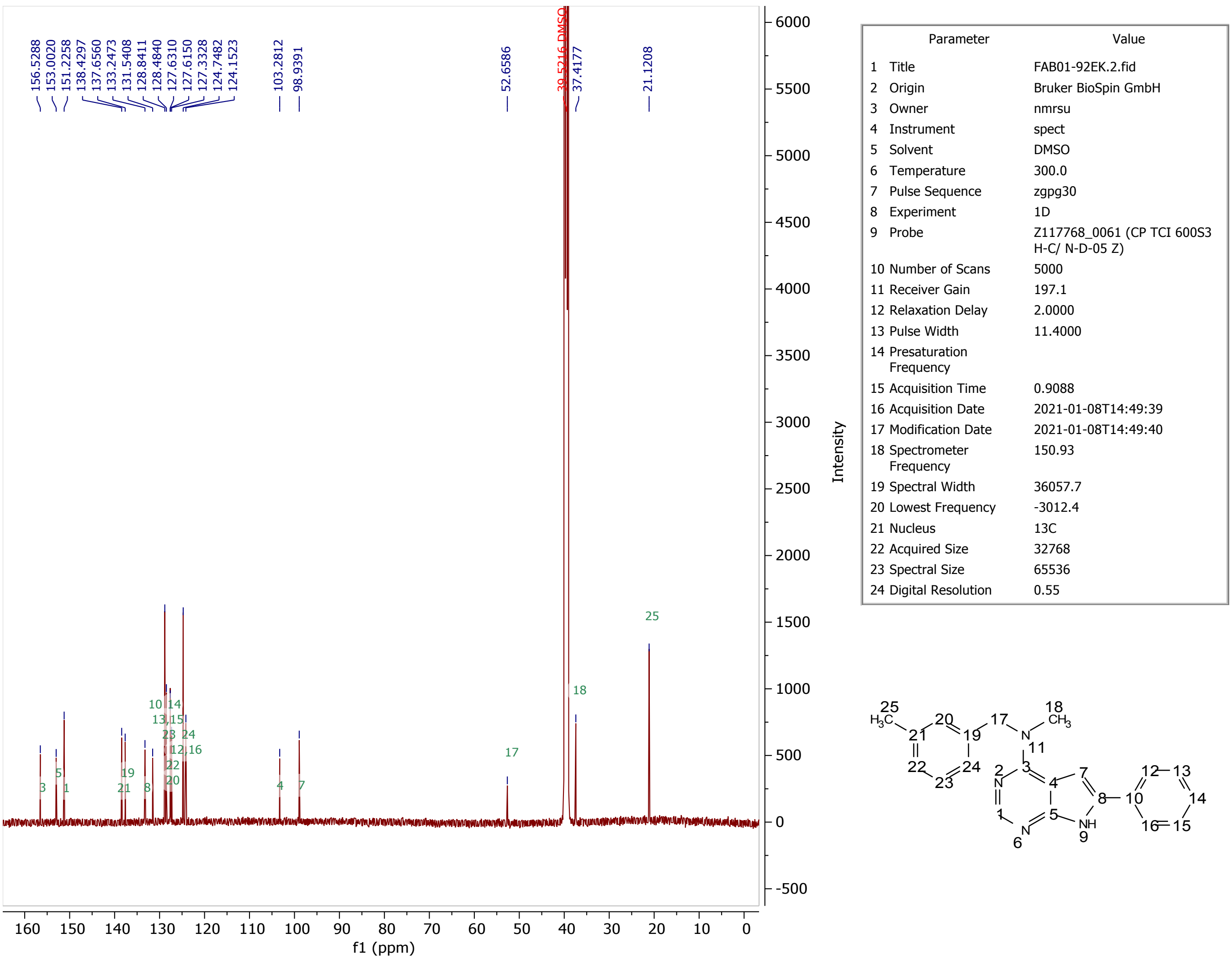

Figure S71. ^{13}C NMR (150 MHz, DMSO-d_6) of compound **35.**

Compound 36

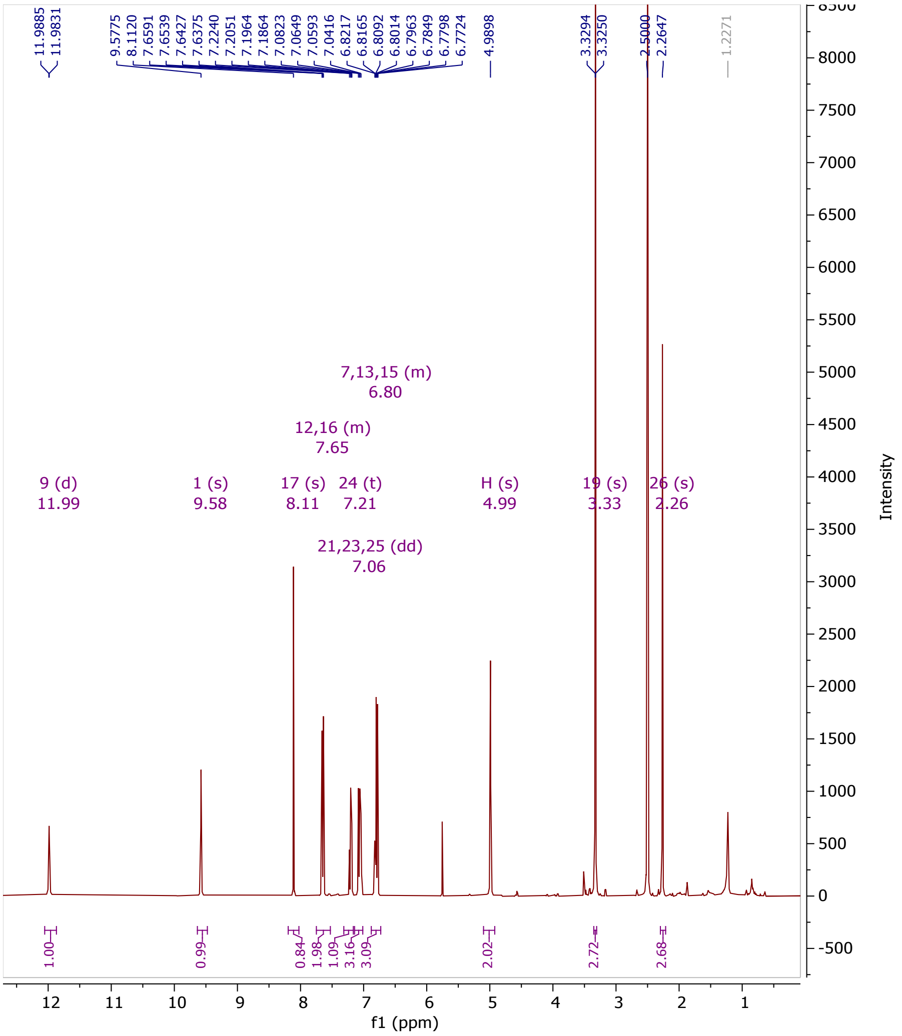

	Parameter	Value
1	Title	FAB-01-89-k3-f1415.1.fid
2	Comment	
3	Origin	Bruker BioSpin GmbH
4	Owner	nmrsu
5	Site	
6	Instrument	spect
7	Author	
8	Solvent	DMSO
9	Temperature	298.3
10	Pulse Sequence	zg30
11	Experiment	1D
12	Probe	Z116098_0398 (PA BBO 400S1 BBF-H-D-05 Z SP)
13	Number of Scans	16
14	Receiver Gain	128.1
15	Relaxation Delay	1.0000
16	Pulse Width	9.5000
17	Presaturation Frequency	
18	Acquisition Time	4.0894
19	Acquisition Date	2023-01-24T11:47:28
20	Modification Date	2023-01-24T11:47:28
21	Class	
22	Spectrometer Frequency	400.13
23	Spectral Width	8012.8
24	Lowest Frequency	-1538.8
25	Nucleus	1H
26	Acquired Size	32768
27	Spectral Size	65536
28	Digital Resolution	0.12

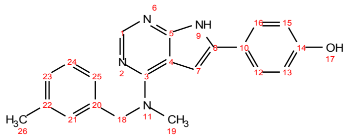

Figure S72. ^{1}H NMR (400 MHz, DMSO-d_6) of compound **36**.

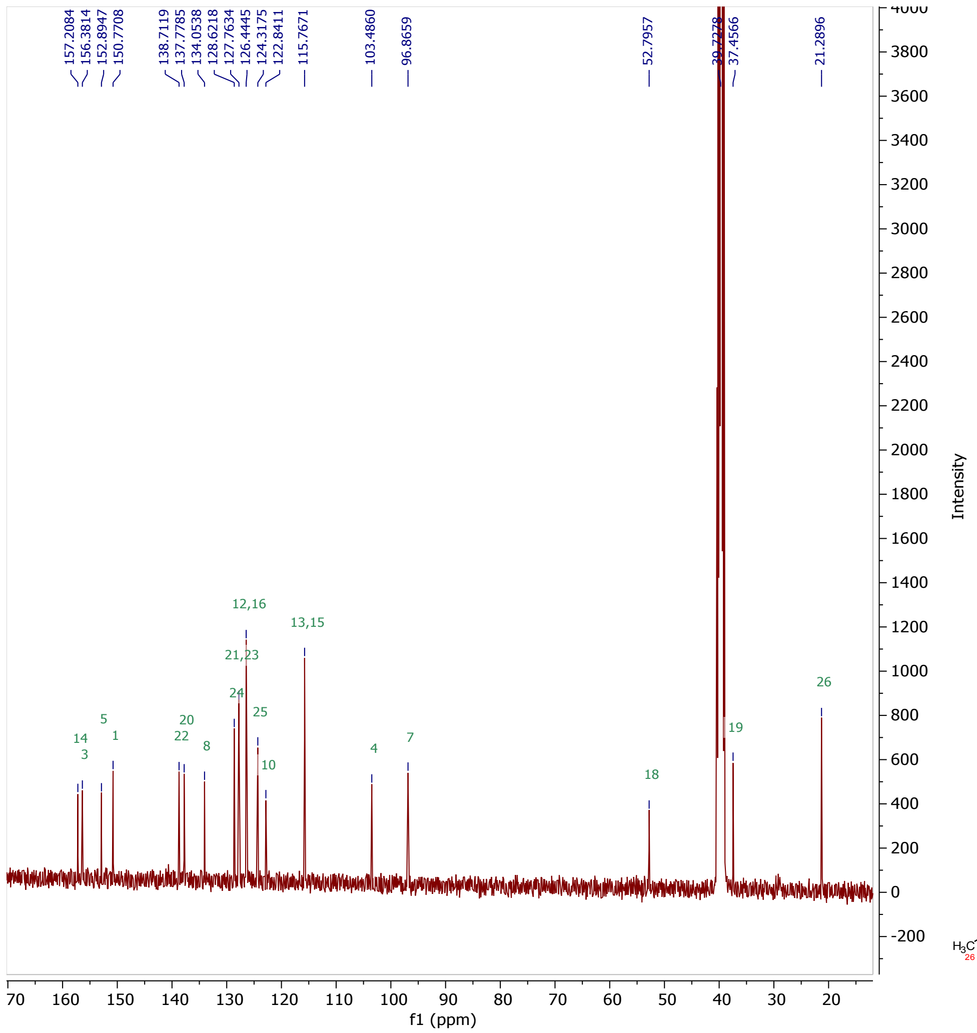

	Parameter	Value
1	Title	FAB-01-89-k3-f1415.2.fid
2	Comment	
3	Origin	Bruker BioSpin GmbH
4	Owner	nmrsu
5	Site	
6	Instrument	spect
7	Author	
8	Solvent	DMSO
9	Temperature	298.3
10	Pulse Sequence	zgpg30
11	Experiment	1D
12	Probe	Z116098_0398 (PA BBO 400S1 BBF-H-D-05 Z SP)
13	Number of Scans	3000
14	Receiver Gain	209.8
15	Relaxation Delay	2.0000
16	Pulse Width	9.5000
17	Presaturation Frequency	
18	Acquisition Time	1.3631
19	Acquisition Date	2023-01-24T18:57:15
20	Modification Date	2023-01-24T18:57:15
21	Class	
22	Spectrometer Frequency	100.62
23	Spectral Width	24038.5
24	Lowest Frequency	-1985.3
25	Nucleus	13C
26	Acquired Size	32768
27	Spectral Size	65536
28	Digital Resolution	0.37

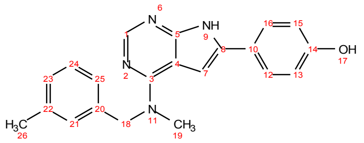

Figure S73. ^{13}C NMR (100 MHz, DMSO-d_6) of compound **36.**

Compound 37

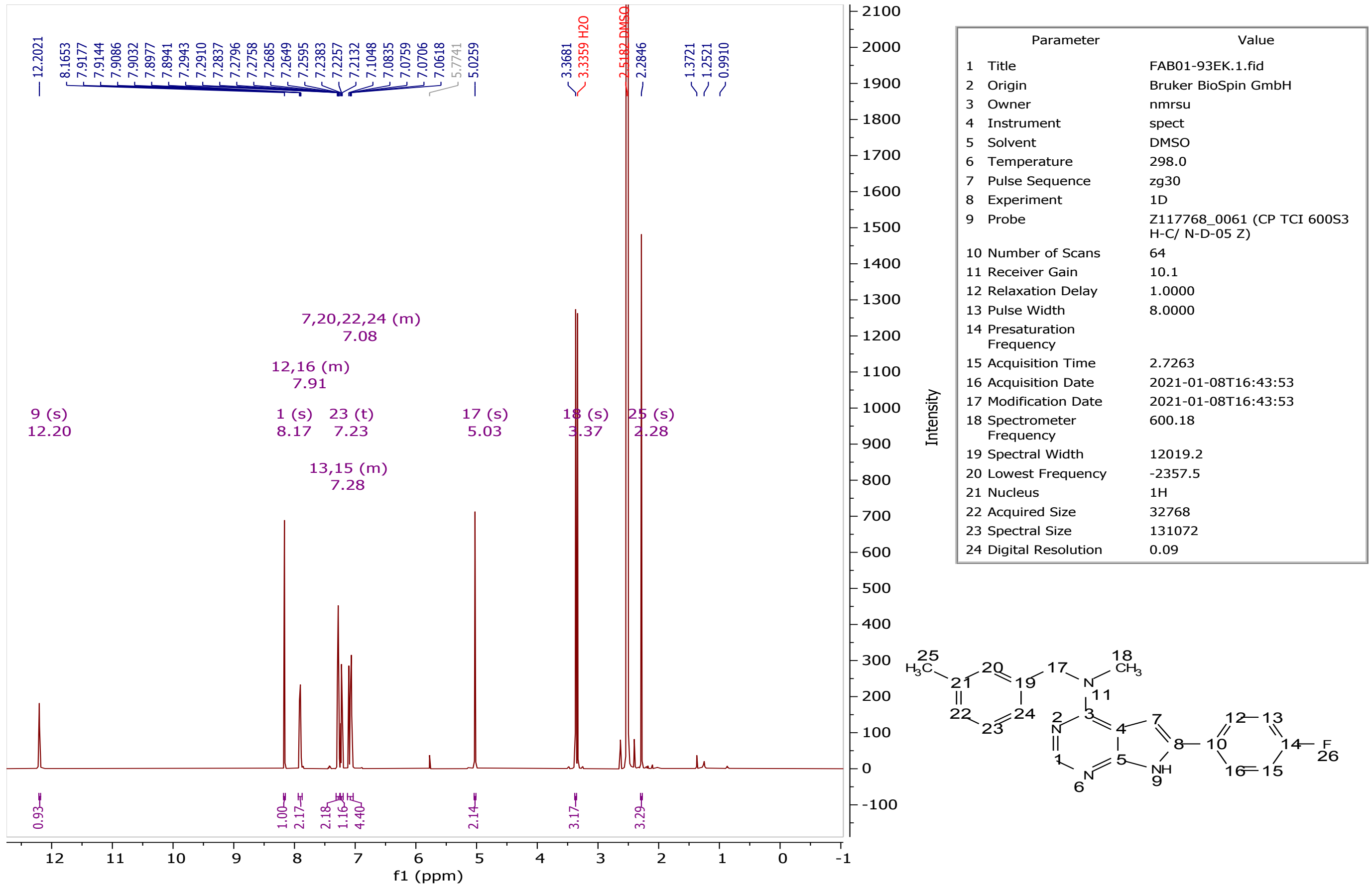

Figure S74. ^{1}H NMR (600 MHz, DMSO-d_6) of compound **37**.

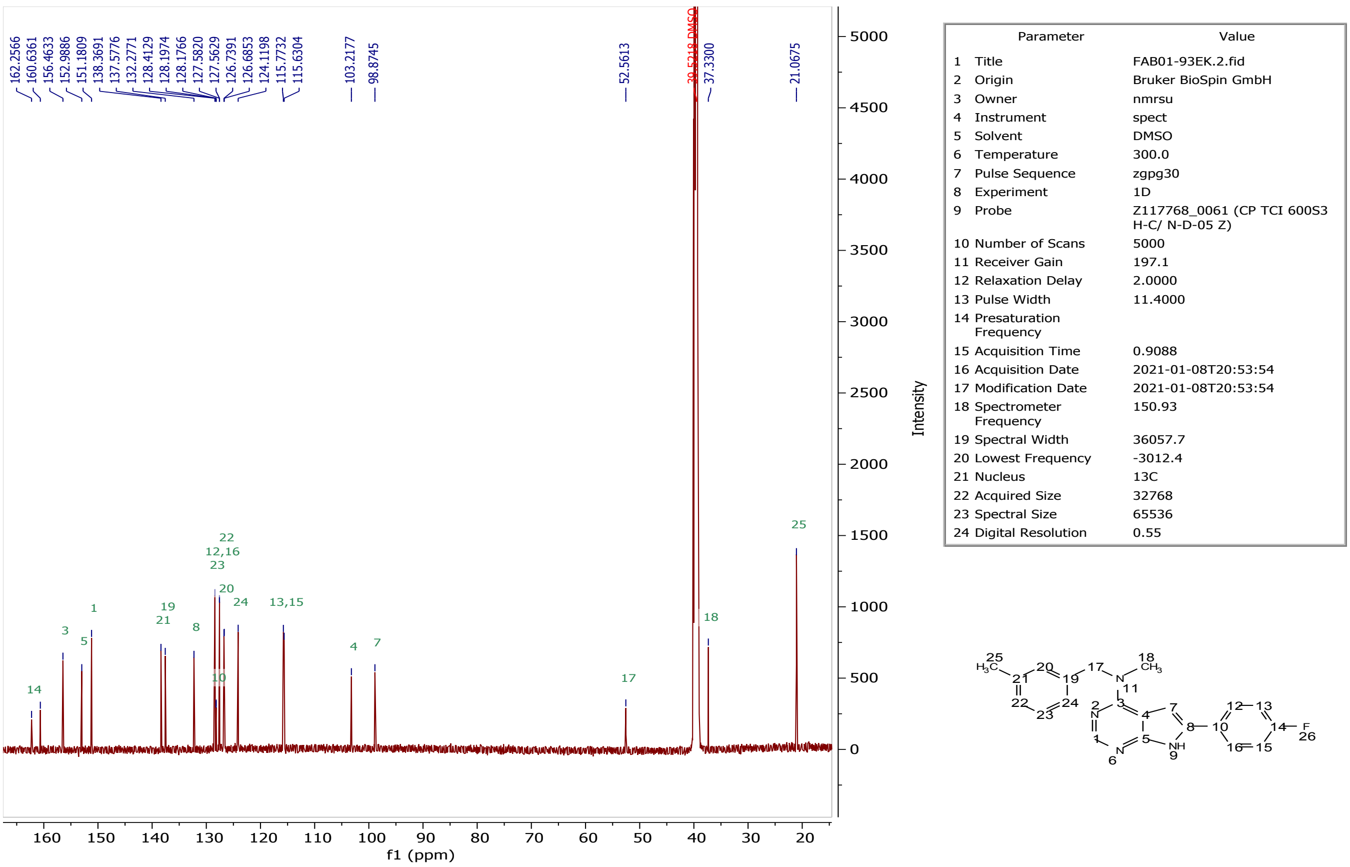

Figure S75. ^{13}C NMR (150 MHz, DMSO-d_6) of compound **37.**

Compound 38

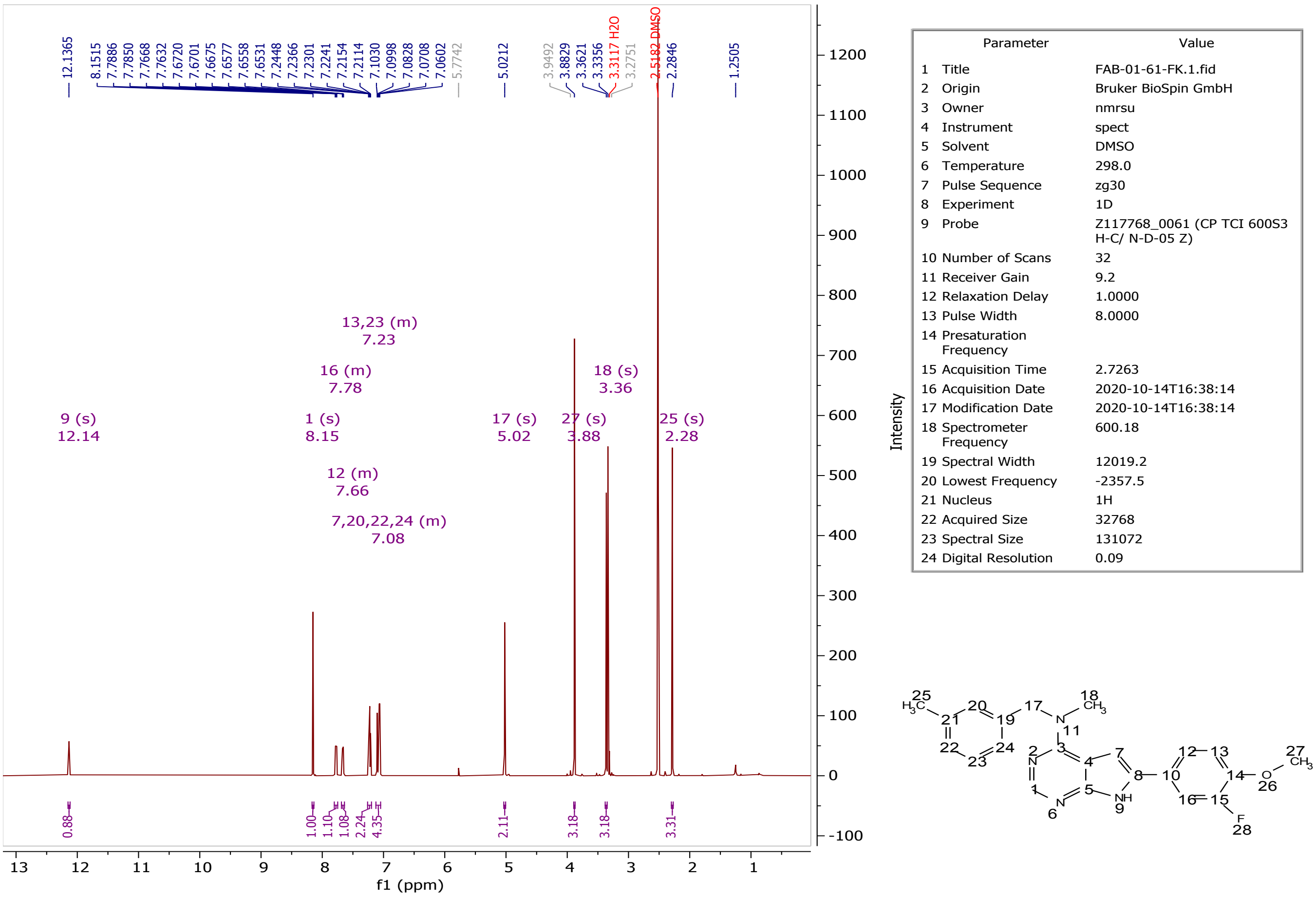

Figure S76. ^{1}H NMR (600 MHz, DMSO-d_6) of compound **38**.

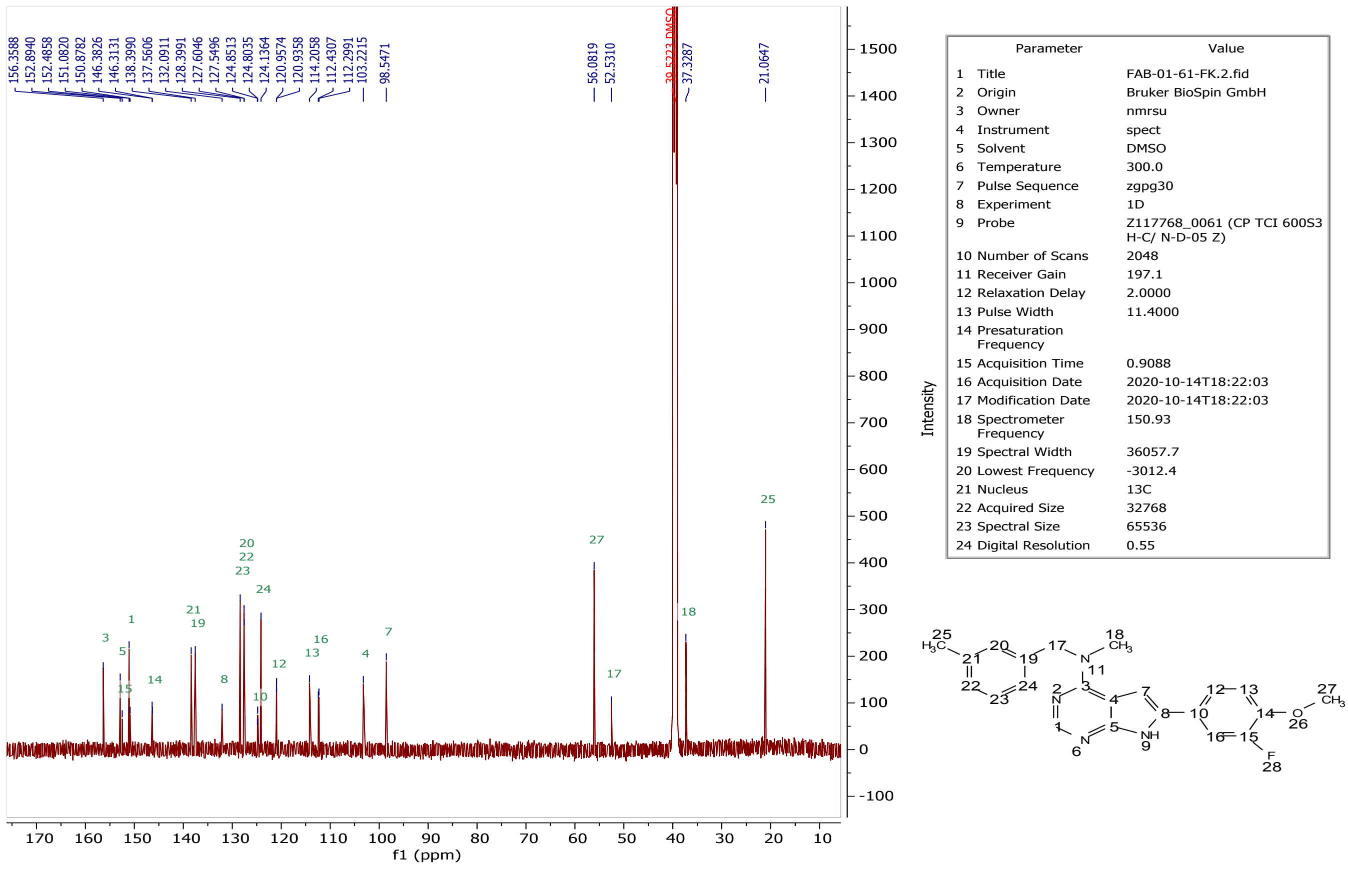

Figure S77. ^{13}C NMR (150 MHz, DMSO-d_6) of compound **38.**

Compound 39

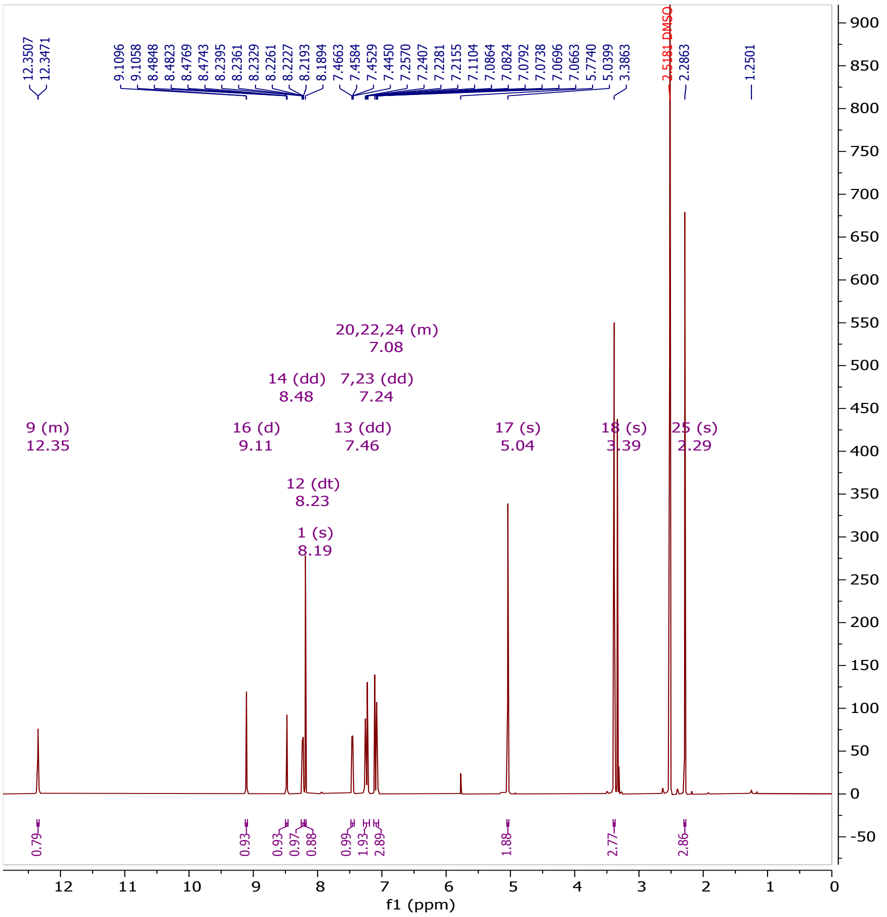

Parameter		Value
1	Title	FAB-01-69.1.fid
2	Origin	Bruker BioSpin GmbH
3	Owner	nmrsu
4	Instrument	spect
5	Solvent	DMSO
6	Temperature	298.0
7	Pulse Sequence	zg30
8	Experiment	1D
9	Probe	Z117768_0061 (CP TCI 600S3 H-C/ N-D-05 Z)
10	Number of Scans	32
11	Receiver Gain	9.2
12	Relaxation Delay	1.0000
13	Pulse Width	8.0000
14	Presaturation Frequency	
15	Acquisition Time	2.7263
16	Acquisition Date	2020-10-15T04:50:59
17	Modification Date	2020-10-15T04:50:59
18	Spectrometer Frequency	600.18
19	Spectral Width	12019.2
20	Lowest Frequency	-2357.5
21	Nucleus	1H
22	Acquired Size	32768
23	Spectral Size	131072
24	Digital Resolution	0.09

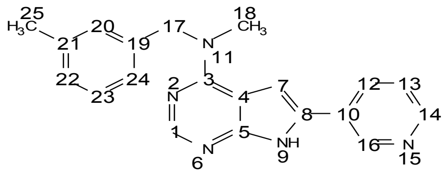

Figure S78. ^{1}H NMR (600 MHz, DMSO-d_6) of compound **39**.

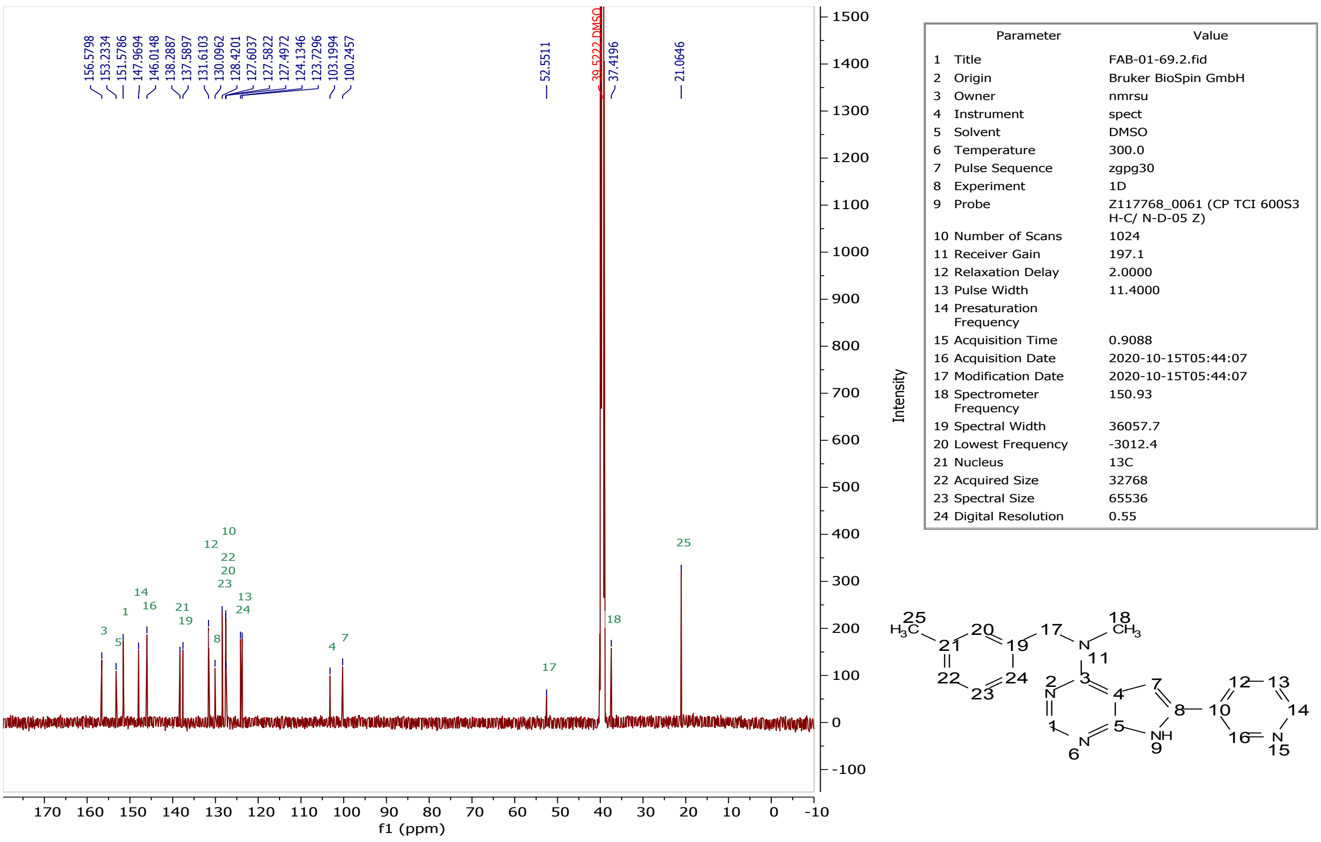

Figure S79. ^{13}C NMR (150 MHz, DMSO-d_6) of compound **39.**

Compound 40

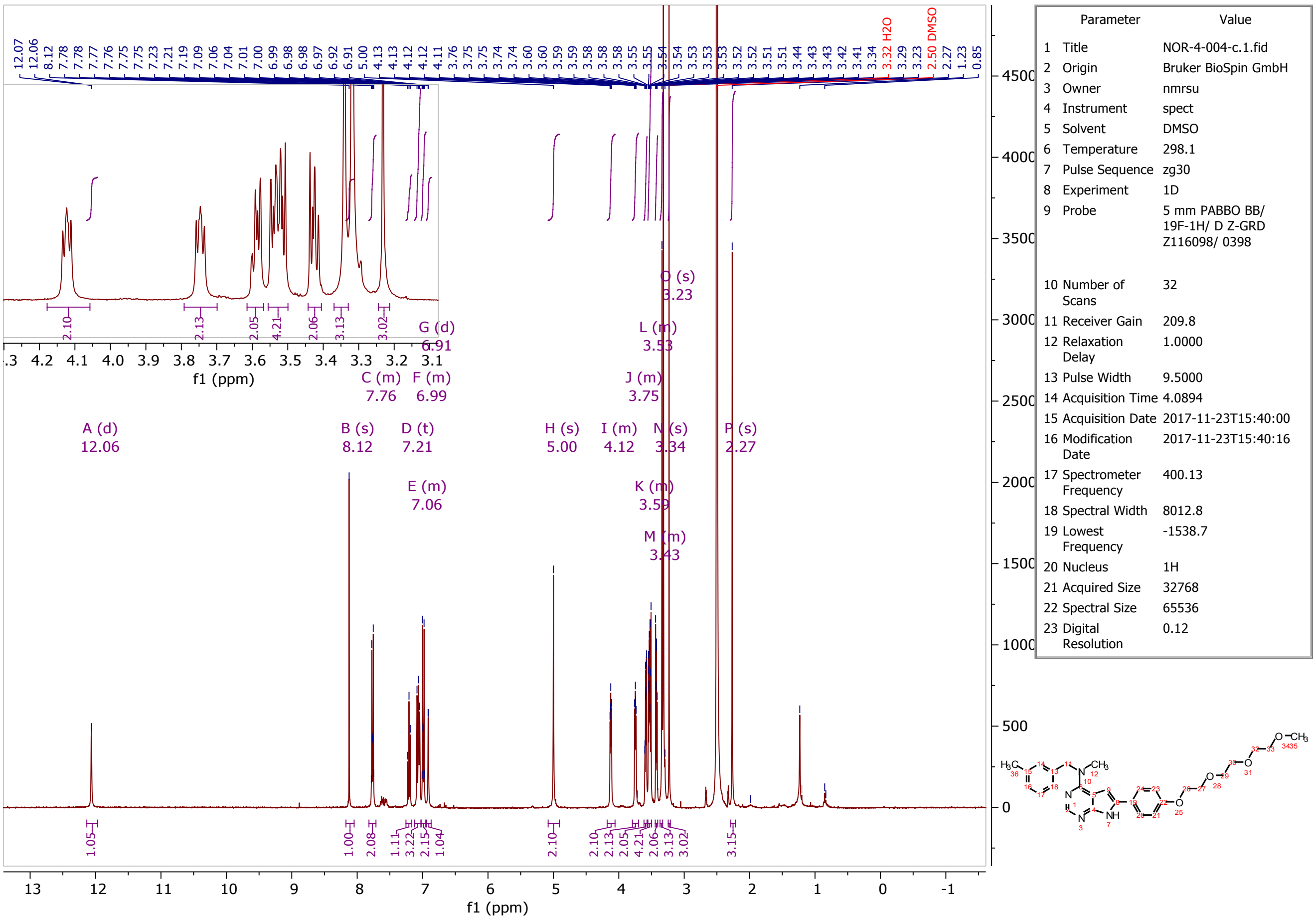

Figure S80. ^{1}H NMR (400 MHz, DMSO-d_6) of compound **40**.

Compound 41

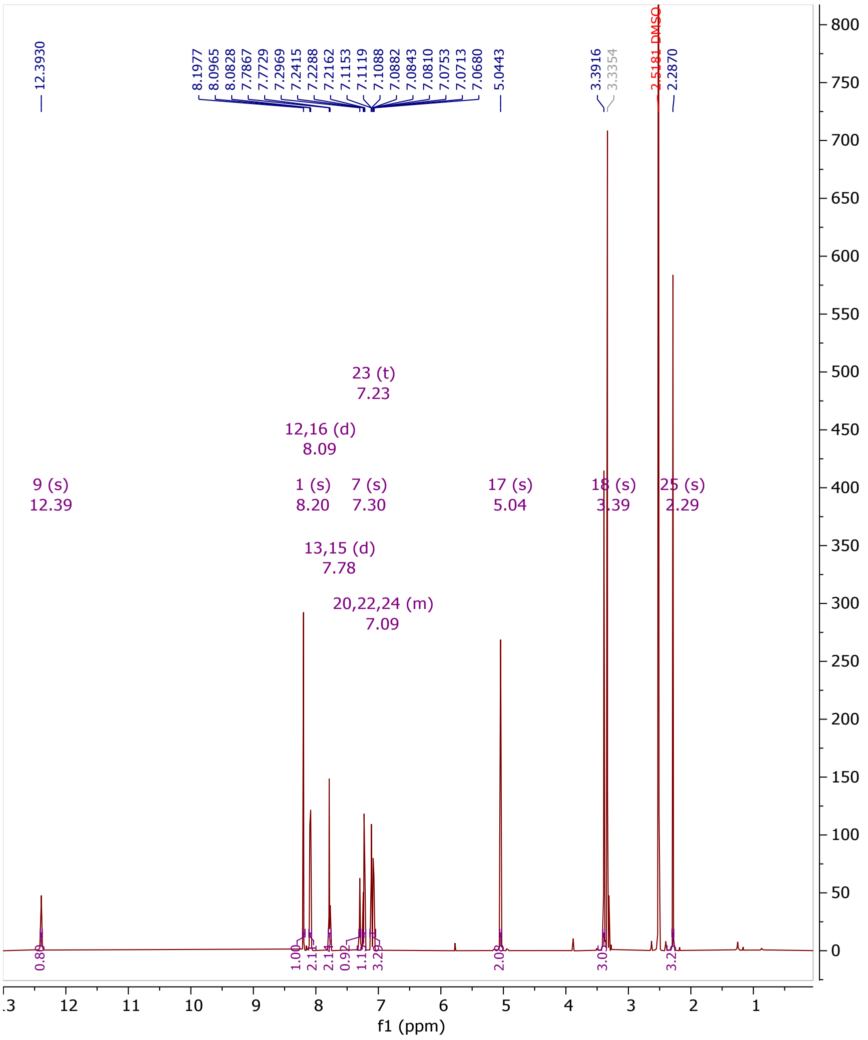

	Parameter	Value
1	Data File Name	F:/ NMR/ Fullkarakterisering acv slutt produkter/ FAB-01-62-FK/ 1/ fid
2	Title	FAB-01-62-FK.1.fid
3	Comment	PROTON DMSO {C:\ Users\ nmrsu\ Documents} frithjob 20
4	Origin	Bruker BioSpin GmbH
5	Owner	nmrsu
6	Site	
7	Instrument	spect
8	Author	
9	Solvent	DMSO
10	Temperature	298.0
11	Pulse Sequence	zg30
12	Experiment	1D
13	Probe	Z117768_0061 (CP TCI 600S3 H-C/ N-D-05 Z)
14	Number of Scans	32
15	Receiver Gain	10.1
16	Relaxation Delay	1.0000
17	Pulse Width	8.0000
18	Presaturation Frequency	
19	Acquisition Time	2.7263
20	Acquisition Date	2020-10-14T22:58:34
21	Modification Date	2020-10-14T22:58:34
22	Class	
23	Spectrometer Frequency	600.18
24	Spectral Width	12019.2
25	Lowest Frequency	-2303.5
26	Nucleus	1H
27	Acquired Size	32768
28	Spectral Size	131072
29	Digital Resolution	0.09

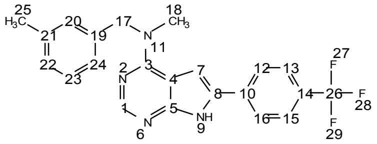

Figure S81. ^{1}H NMR (600 MHz, DMSO-d_6) of compound **41**.

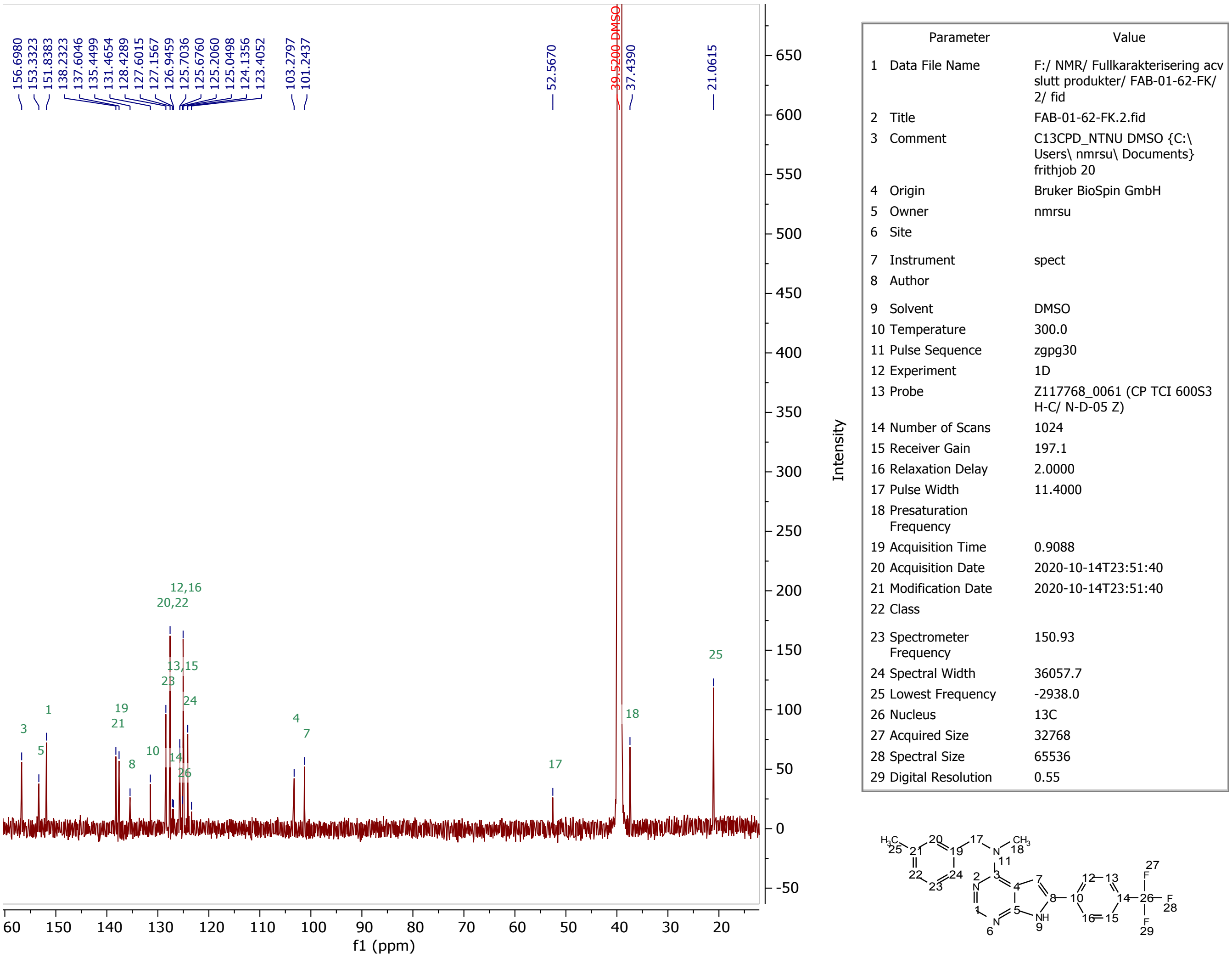

Figure S82. ^{13}C NMR (150 MHz, DMSO-d_6) of compound **41.**

Compound 42

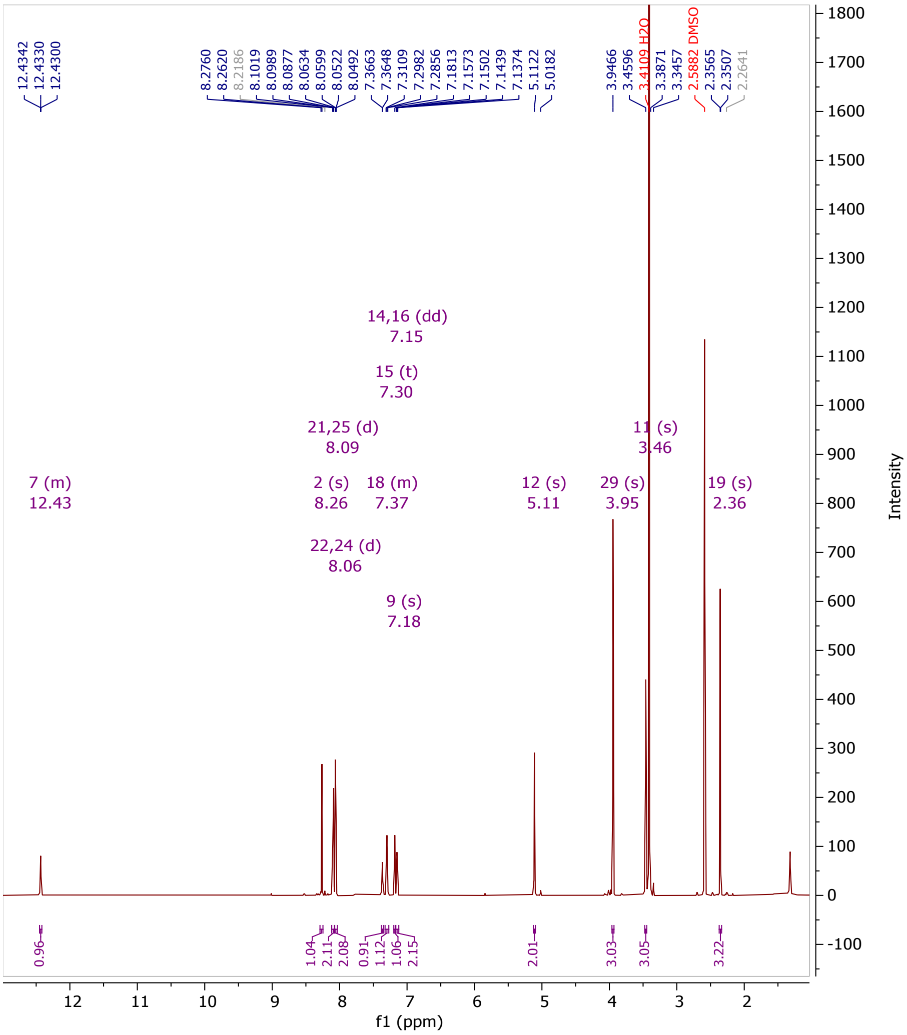

Parameter	Value
1 Title	FAB-01-45FK.1.fid
2 Origin	Bruker BioSpin GmbH
3 Owner	nmrsu
4 Instrument	spect
5 Solvent	DMSO
6 Temperature	298.0
7 Pulse Sequence	zg30
8 Experiment	1D
9 Probe	Z117768_0061 (CP TCI 600S3 H-C/ N-D-05 Z)
10 Number of Scans	32
11 Receiver Gain	9.2
12 Relaxation Delay	1.0000
13 Pulse Width	8.0000
14 Presaturation Frequency	
15 Acquisition Time	2.7263
16 Acquisition Date	2020-10-16T20:19:15
17 Modification Date	2020-10-16T20:19:15
18 Spectrometer Frequency	600.18
19 Spectral Width	12019.2
20 Lowest Frequency	-2357.5
21 Nucleus	1H
22 Acquired Size	32768
23 Spectral Size	131072
24 Digital Resolution	0.09

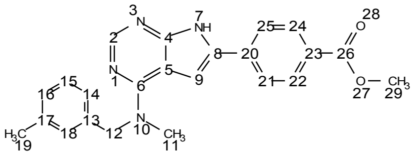

Figure S83. ^{1}H NMR (600 MHz, DMSO-d_6) of compound **42**.

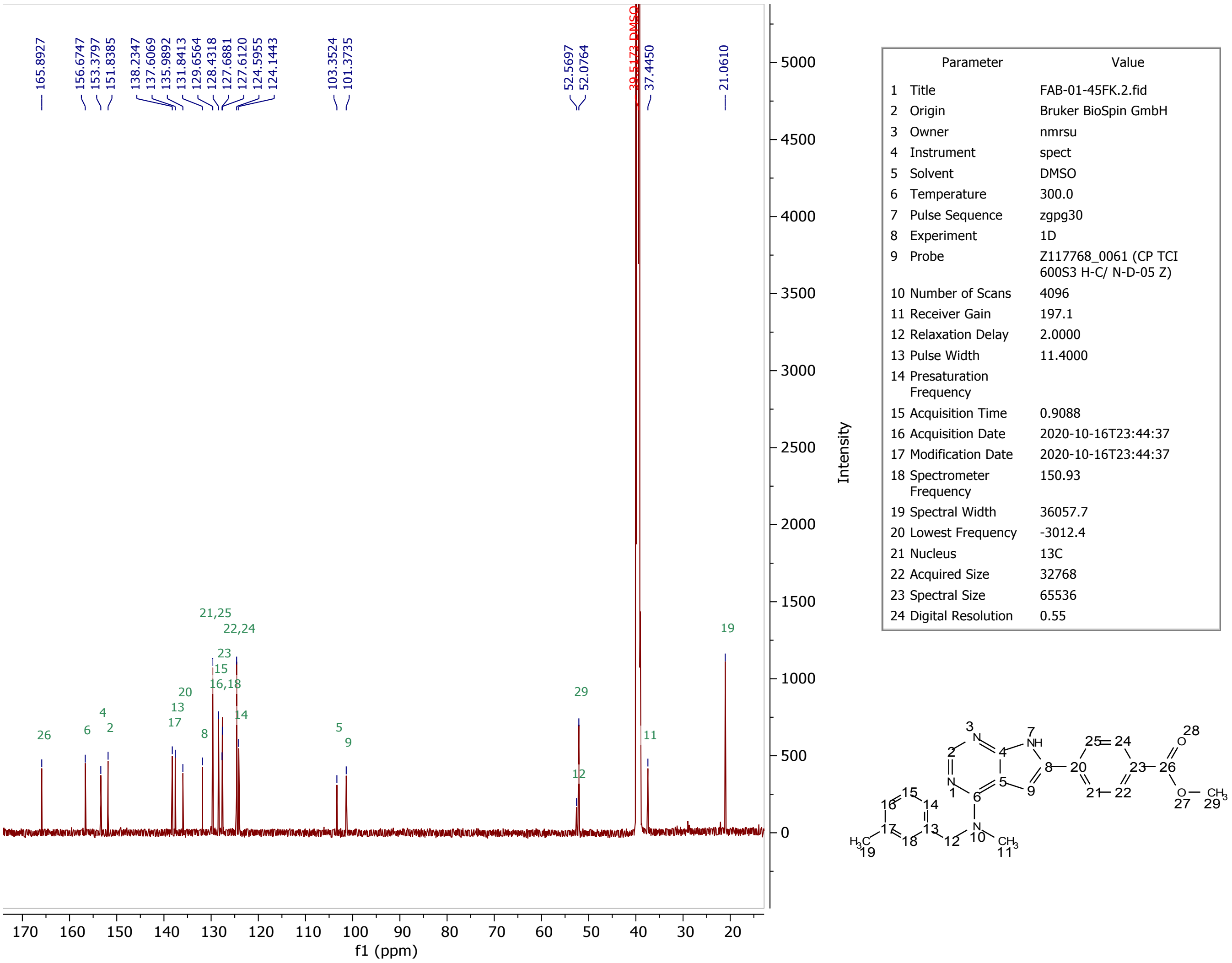

Figure S84. ^{13}C NMR (150 MHz, DMSO-d_6) of compound **42.**

Compound 43

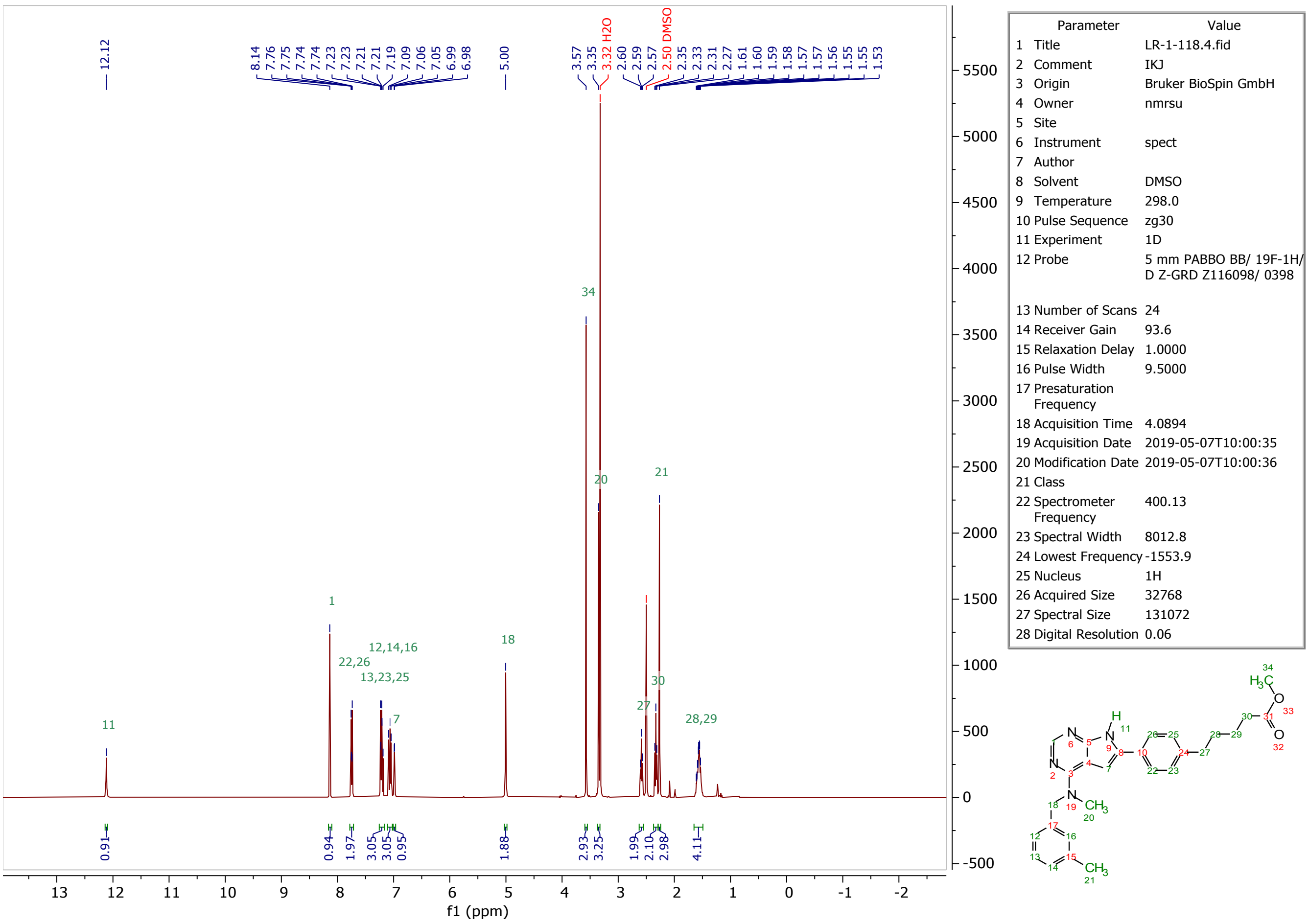

Figure S85. ^{1}H NMR (400 MHz, DMSO-d_6) of compound **43**.

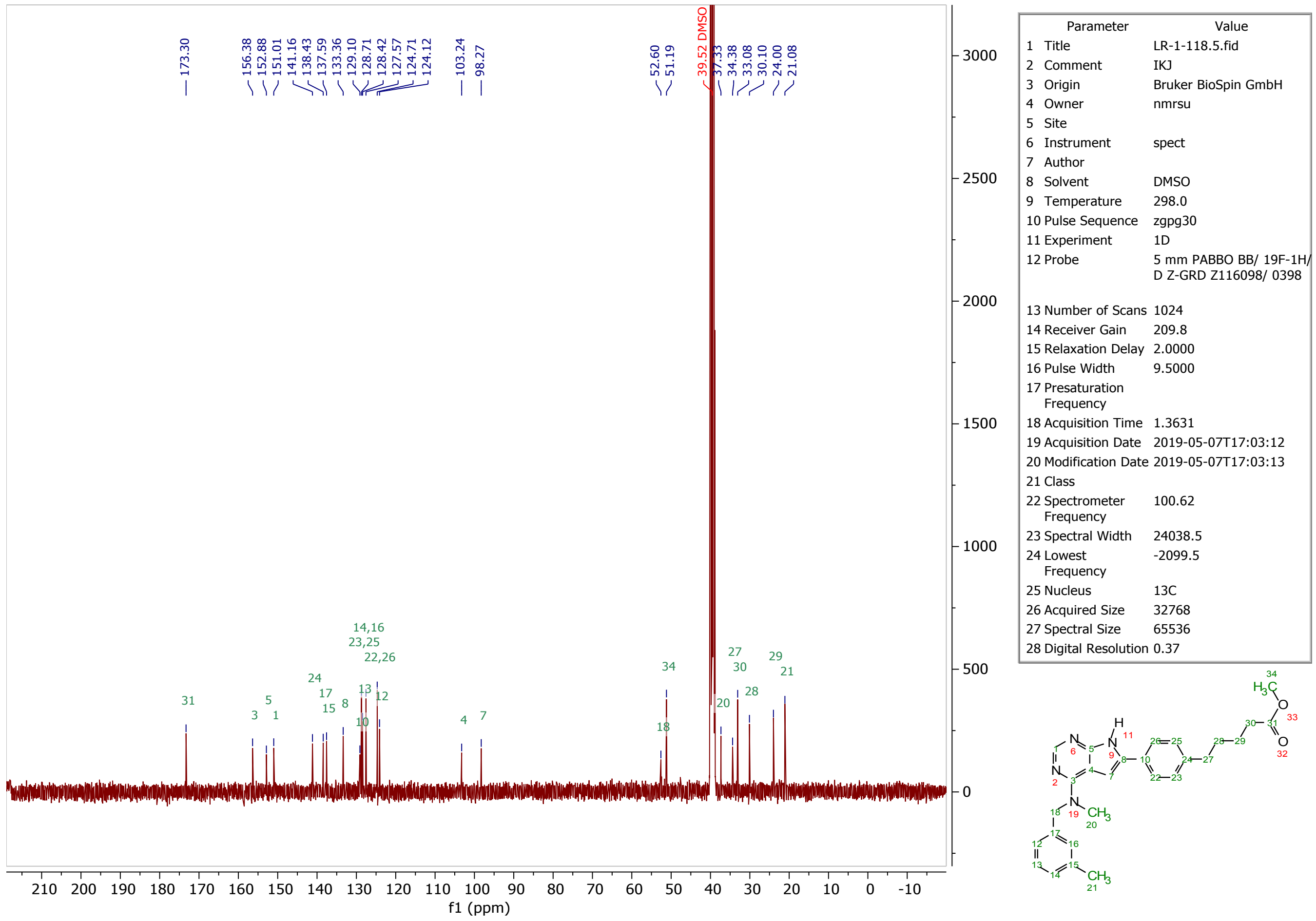

Figure S86. ^{13}C NMR (100 MHz, DMSO-d_6) of compound **43.**

Compound 44

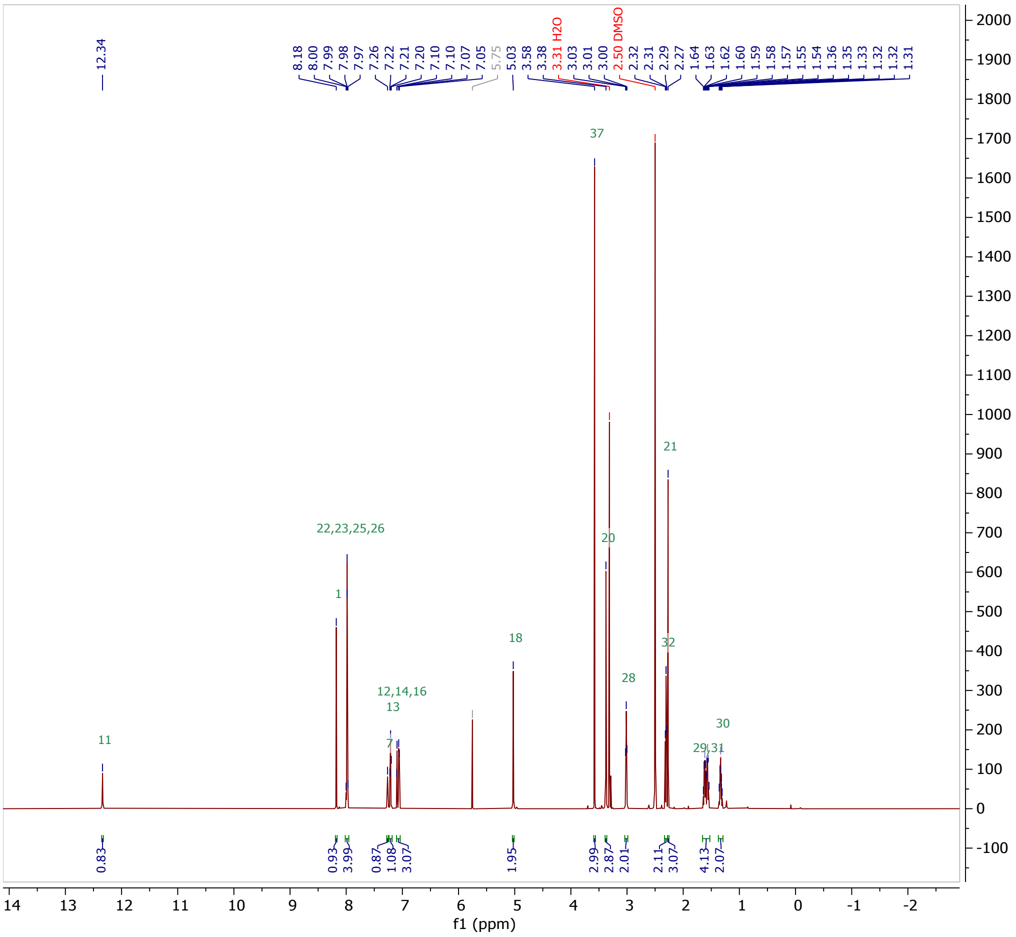

	Parameter	Value
1	Title	LR-1-138 (600MHz).1.fid
2	Comment	
3	Origin	Bruker BioSpin GmbH
4	Owner	nmrsu
5	Site	
6	Instrument	spect
7	Author	
8	Solvent	DMSO
9	Temperature	300.0
10	Pulse Sequence	zg30
11	Experiment	1D
12	Probe	Z117768_0061 (CP TCI 600S3 H-C/ N-D-05 Z)
13	Number of Scans	16
14	Receiver Gain	10.1
15	Relaxation Delay	1.0000
16	Pulse Width	8.0000
17	Presaturation Frequency	
18	Acquisition Time	2.7263
19	Acquisition Date	2019-06-06T14:12:45
20	Modification Date	2019-06-06T14:12:45
21	Class	
22	Spectrometer Frequency	600.18
23	Spectral Width	12019.2
24	Lowest Frequency	-2336.0
25	Nucleus	1H
26	Acquired Size	32768
27	Spectral Size	131072
28	Digital Resolution	0.09

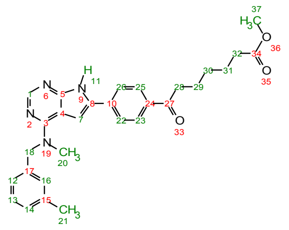

Figure S87. ^{1}H NMR (600 MHz, DMSO-d_6) of compound **44**.

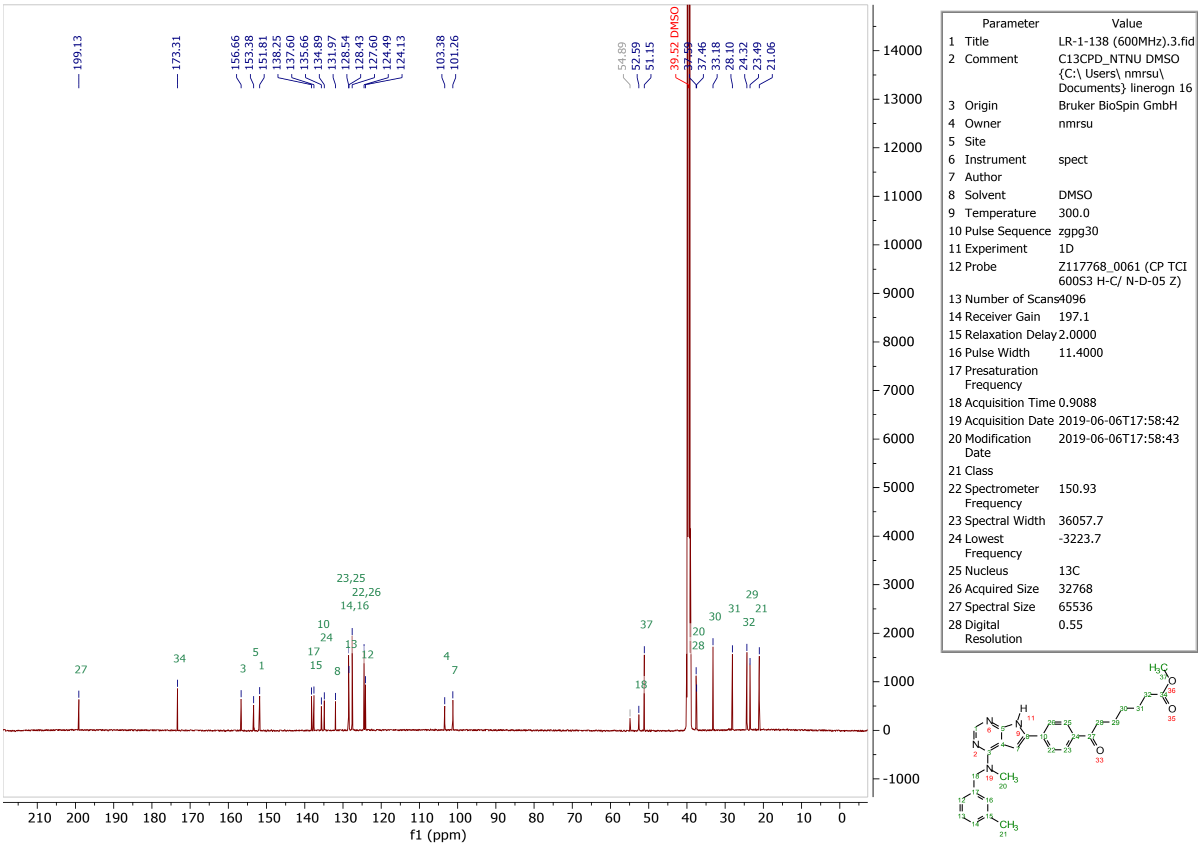

Figure S88. ^{13}C NMR (150 MHz, DMSO-d_6) of compound **44.**

Compound 45

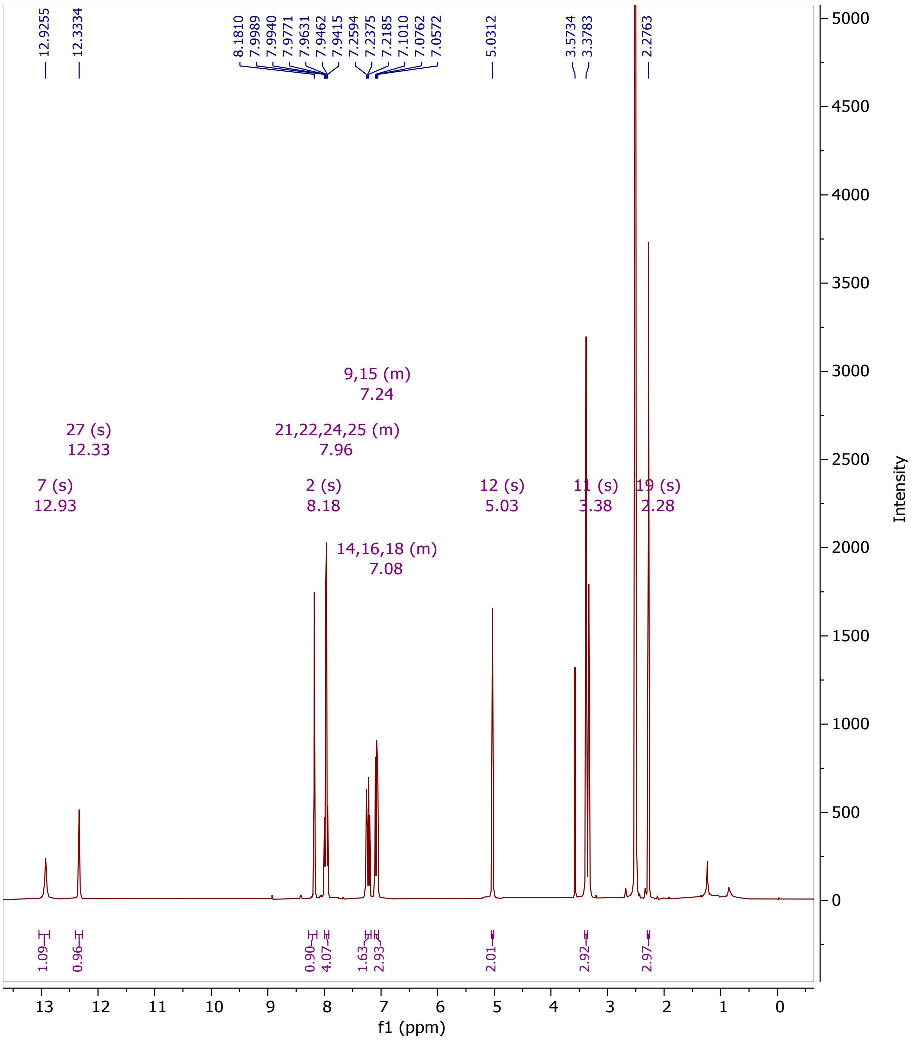

	Parameter	Value
1	Data File Name	F:/ Fullkarakterisering/ Ferdig NMR/ Nye spektere/ FAB-01-91-TEST/ 1/ fid
2	Title	FAB-01-39/91-TEST.1.fid
3	Comment	
4	Origin	Bruker BioSpin GmbH
5	Owner	nmrsu
6	Site	
7	Instrument	spect
8	Author	
9	Solvent	DMSO
10	Temperature	298.3
11	Pulse Sequence	zg30
12	Experiment	1D
13	Probe	Z116098_0398 (PA BBO 400S1 BBF-H-D-05 Z SP)
14	Number of Scans	16
15	Receiver Gain	143.5
16	Relaxation Delay	1.0000
17	Pulse Width	9.5000
18	Presaturation Frequency	
19	Acquisition Time	4.0894
20	Acquisition Date	2023-01-11T14:16:03
21	Modification Date	2023-01-11T14:16:03
22	Class	
23	Spectrometer Frequency	400.13
24	Spectral Width	8012.8
25	Lowest Frequency	-1535.4
26	Nucleus	1H
27	Acquired Size	32768
28	Spectral Size	65536
29	Digital Resolution	0.12

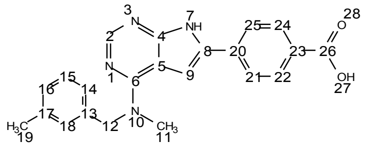

Figure S89. ^{1}H NMR (400 MHz, DMSO-d_6) of compound **45**.

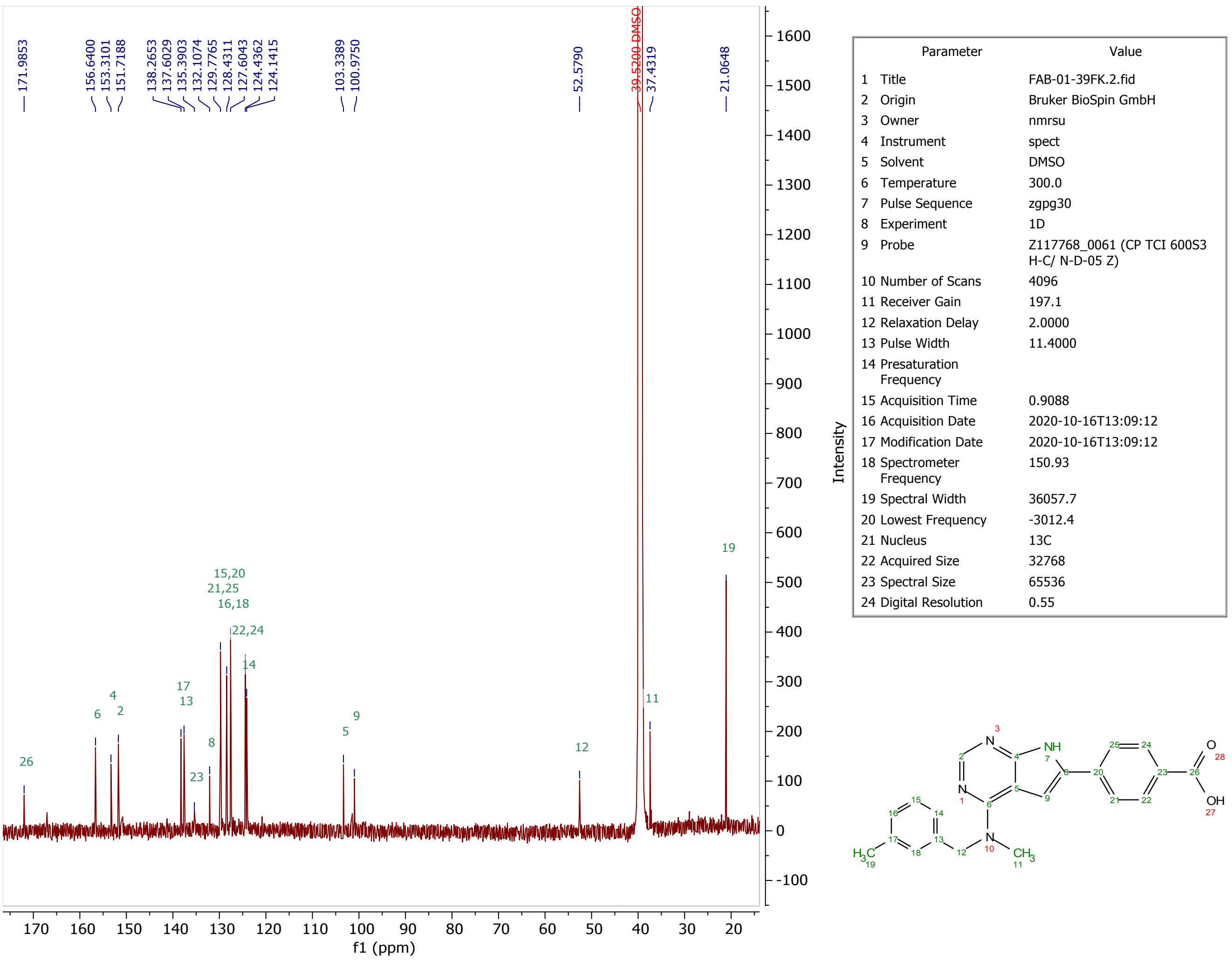

Figure S90. ^{13}C NMR (150 MHz, DMSO-d_6) of compound **45.**

Compound 46

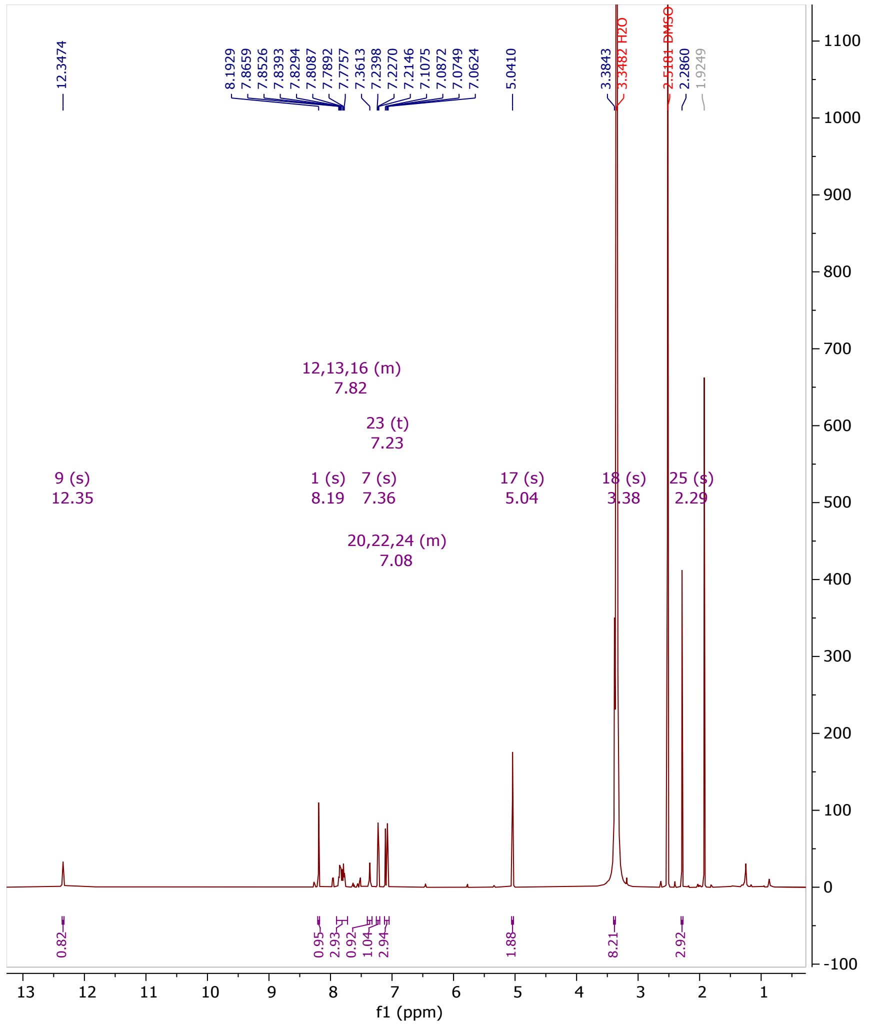

	Parameter	Value
1	Title	FAB-01-63UPKONS.1.fid
2	Origin	Bruker BioSpin GmbH
3	Owner	nmrsu
4	Instrument	spect
5	Solvent	DMSO
6	Temperature	298.0
7	Pulse Sequence	zg30
8	Experiment	1D
9	Probe	Z117768_0061 (CP TCI 600S3 H-C/ N-D-05 Z)
10	Number of Scans	32
11	Receiver Gain	9.2
12	Relaxation Delay	1.0000
13	Pulse Width	8.0000
14	Presaturation Frequency	
15	Acquisition Time	2.7263
16	Acquisition Date	2020-10-17T06:54:09
17	Modification Date	2020-10-17T06:54:10
18	Spectrometer Frequency	600.18
19	Spectral Width	12019.2
20	Lowest Frequency	-2357.5
21	Nucleus	1H
22	Acquired Size	32768
23	Spectral Size	131072
24	Digital Resolution	0.09

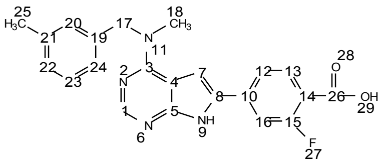

Figure S91. ^{1}H NMR (600 MHz, DMSO-d_6) of compound **46**. The sample contains some acetic acid added during NMR experiment to increase solubility.

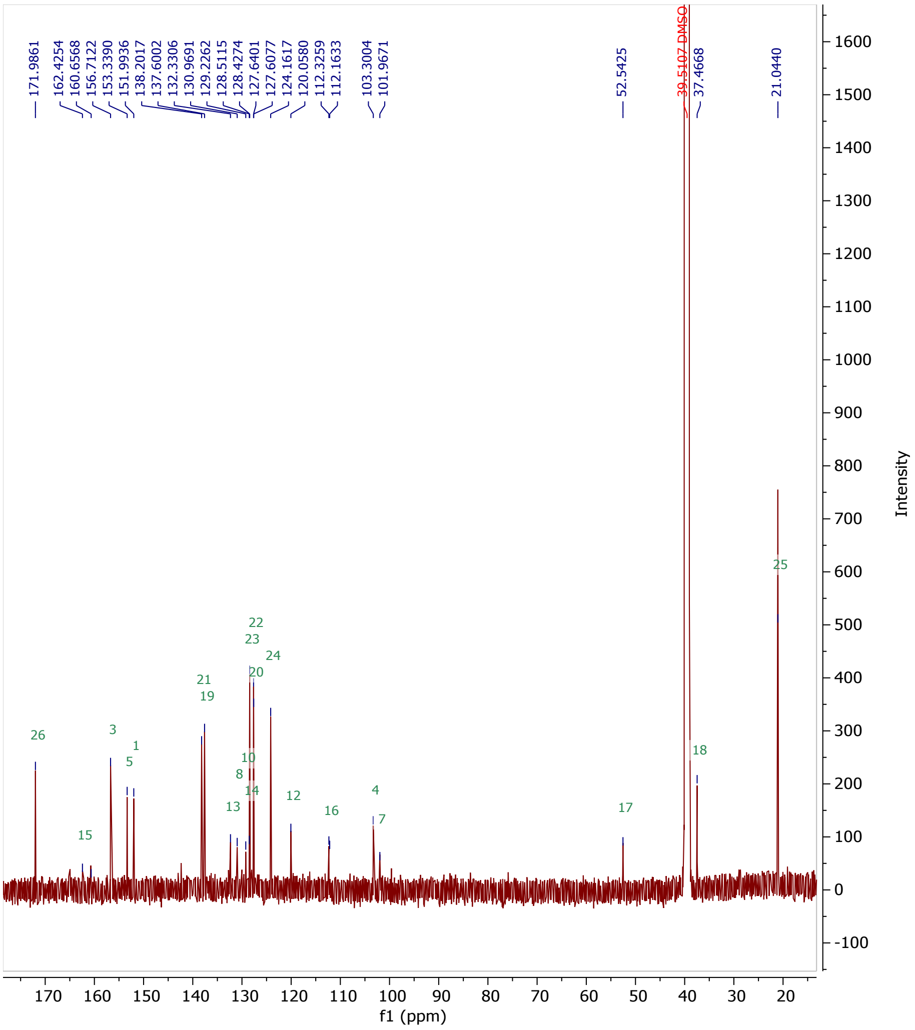

	Parameter	Value
1	Title	FAB-01-63FK_FK2.2.fid
2	Origin	Bruker BioSpin GmbH
3	Owner	nmrsu
4	Instrument	spect
5	Solvent	DMSO
6	Temperature	300.0
7	Pulse Sequence	zgpg30
8	Experiment	1D
9	Probe	Z117768_0061 (CP TCI 600S3 H-C/ N-D-05 Z)
10	Number of Scans	4096
11	Receiver Gain	197.1
12	Relaxation Delay	2.0000
13	Pulse Width	11.4000
14	Presaturation Frequency	
15	Acquisition Time	0.9088
16	Acquisition Date	2020-10-17T10:19:27
17	Modification Date	2020-10-17T10:19:27
18	Spectrometer Frequency	150.93
19	Spectral Width	36057.7
20	Lowest Frequency	-3012.4
21	Nucleus	13C
22	Acquired Size	32768
23	Spectral Size	65536
24	Digital Resolution	0.55

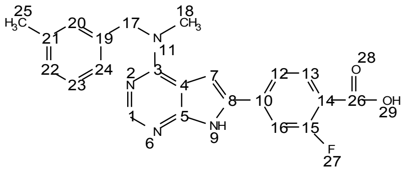

Figure S92. ^{13}C NMR (150 MHz, DMSO-d_6) of compound **46.**

Compound 47

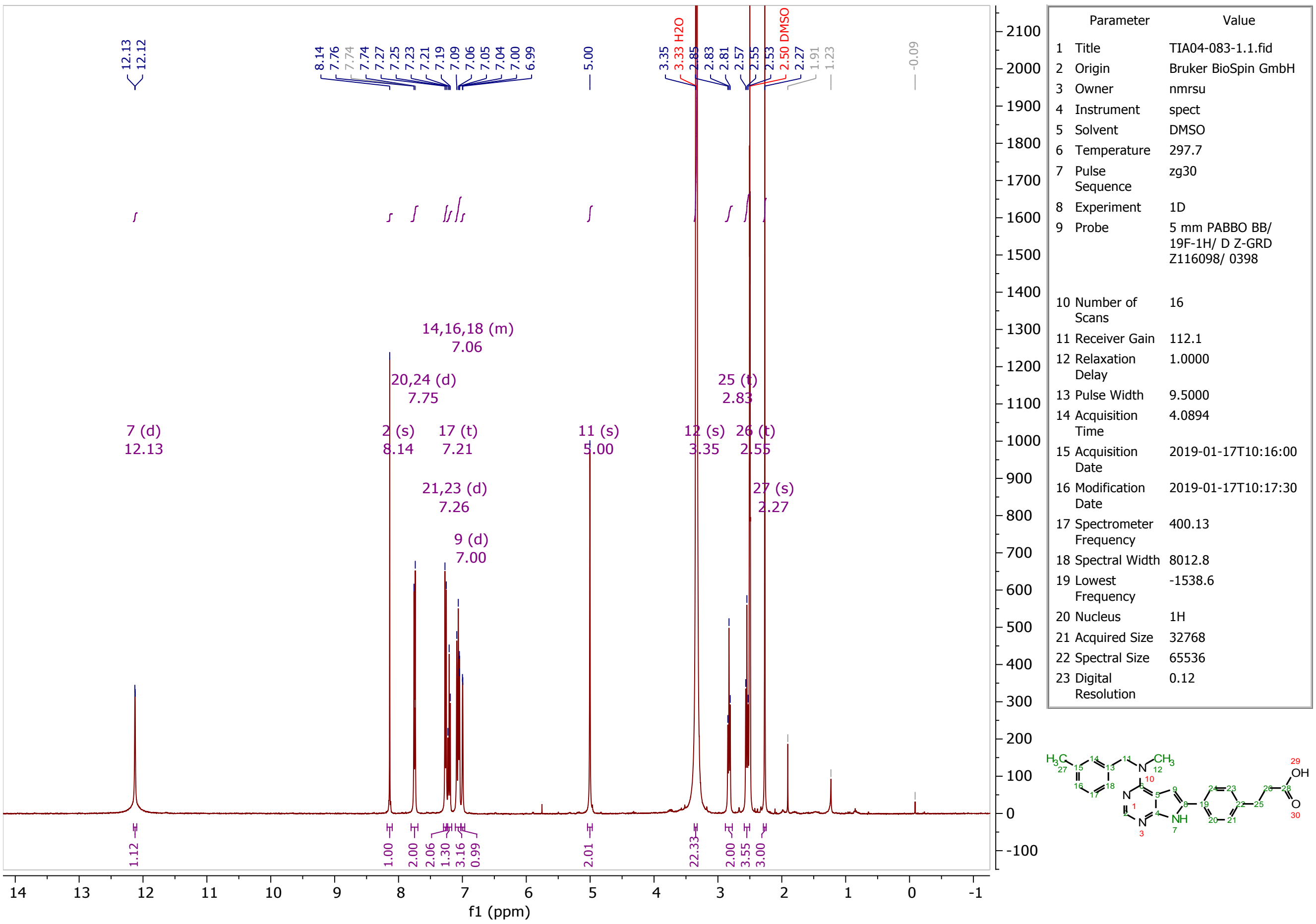

Figure S93. ^{1}H NMR (400 MHz, DMSO-d_6) of compound **47**.

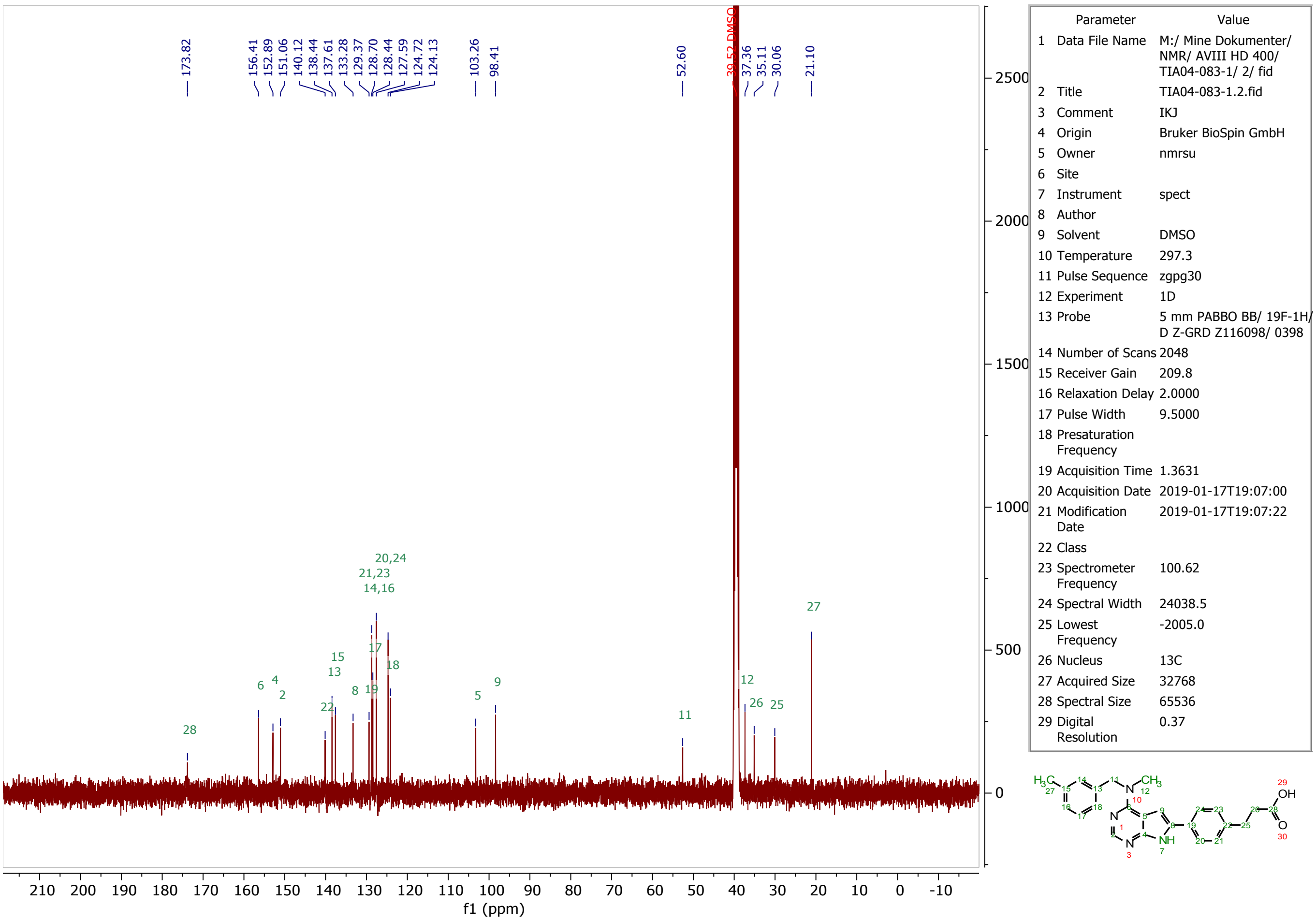

Figure S94. ^{13}C NMR (100 MHz, DMSO-d_6) of compound **47.**

Compound 48

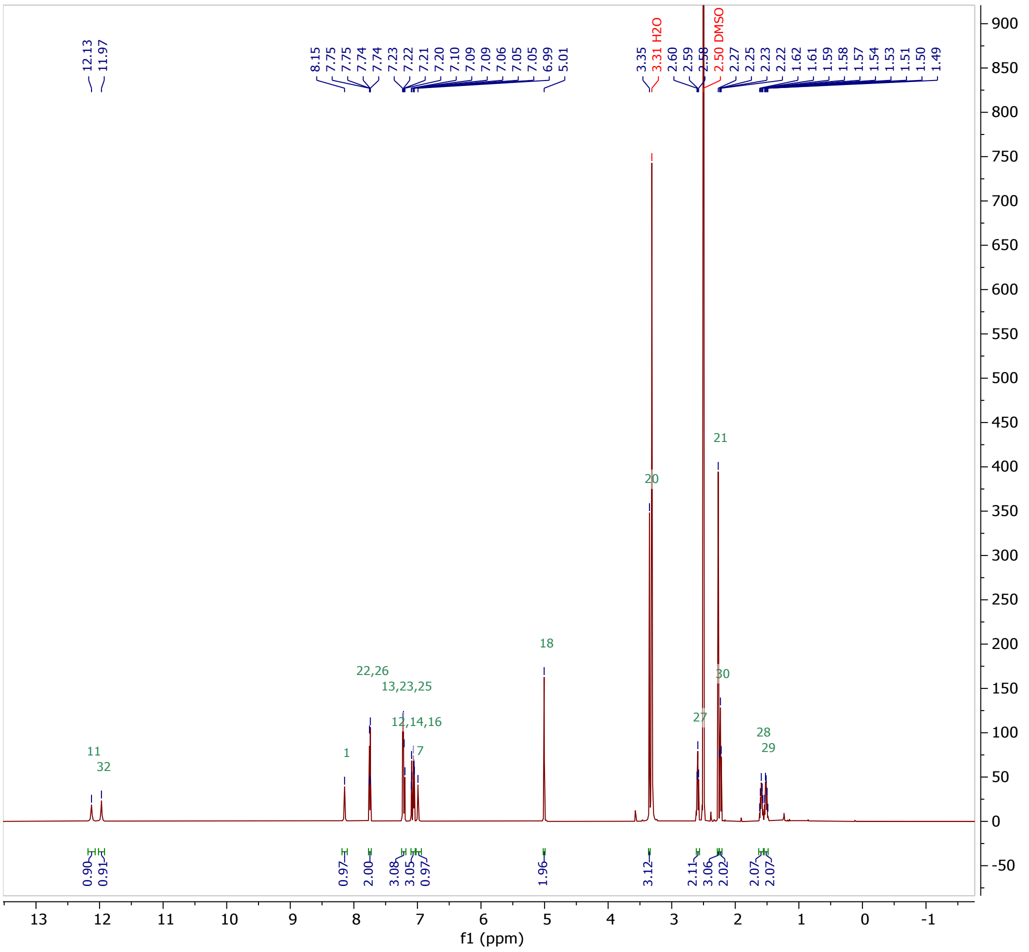

Parameter	Value
1 Title	LR-1-106 (MHz).1.fid
2 Comment	PROTON DMSO {C:\ Users\ nmrsu\ Documents} nmrsu 1
3 Origin	Bruker BioSpin GmbH
4 Owner	nmrsu
5 Site	
6 Instrument	spect
7 Author	
8 Solvent	DMSO
9 Temperature	300.0
10 Pulse Sequence	zg30
11 Experiment	1D
12 Probe	Z117768_0061 (CP TCI 600S3 H-C/ N-D-05 Z)
13 Number of Scans	16
14 Receiver Gain	11.5
15 Relaxation Delay	1.0000
16 Pulse Width	8.0000
17 Presaturation Frequency	
18 Acquisition Time	2.7263
19 Acquisition Date	2019-06-11T13:54:31
20 Modification Date	2019-06-11T13:54:31
21 Class	
22 Spectrometer Frequency	600.18
23 Spectral Width	12019.2
24 Lowest Frequency	-2336.0
25 Nucleus	1H
26 Acquired Size	32768
27 Spectral Size	131072
28 Digital Resolution	0.09

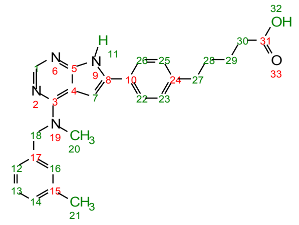

Figure S95. ^{1}H NMR (600 MHz, DMSO-d_6) of compound **48**.

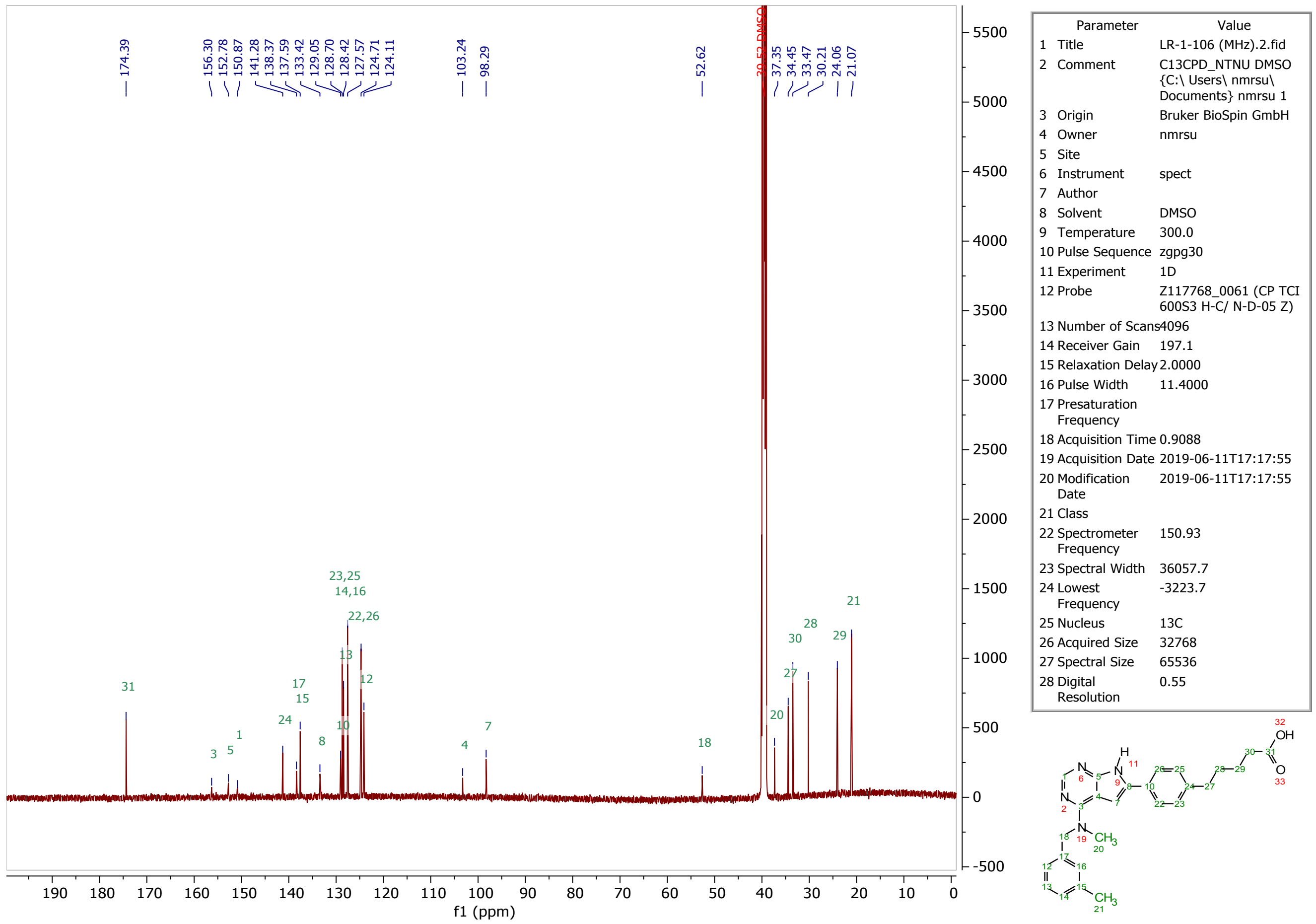

Figure S96. ^{13}C NMR (150 MHz, DMSO-d_6) of compound **48.**

Compound 49

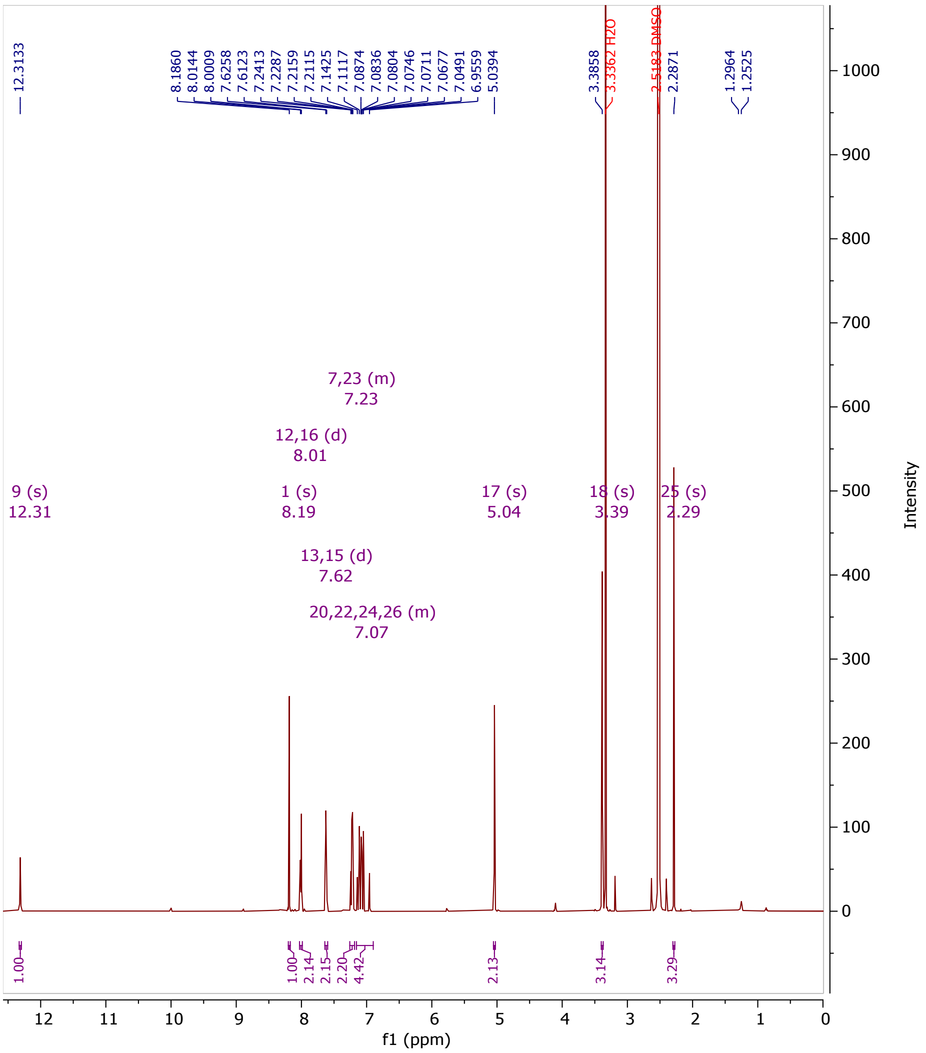

	Parameter	Value
1	Title	FAB-02-13-EKFK.1.fid
2	Origin	Bruker BioSpin GmbH
3	Owner	nmrsu
4	Instrument	spect
5	Solvent	DMSO
6	Temperature	298.0
7	Pulse Sequence	zg30
8	Experiment	1D
9	Probe	Z117768_0061 (CP TCI 600S3 H-C/ N-D-05 Z)
10	Number of Scans	32
11	Receiver Gain	12.9
12	Relaxation Delay	1.0000
13	Pulse Width	8.0000
14	Presaturation Frequency	
15	Acquisition Time	2.7263
16	Acquisition Date	2021-03-23T18:26:10
17	Modification Date	2021-03-23T18:26:10
18	Spectrometer Frequency	600.18
19	Spectral Width	12019.2
20	Lowest Frequency	-2357.5
21	Nucleus	1H
22	Acquired Size	32768
23	Spectral Size	131072
24	Digital Resolution	0.09

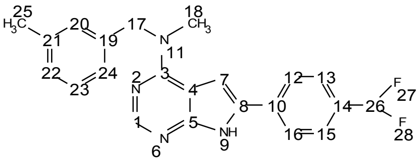

Figure S97. ^{1}H NMR (600 MHz, DMSO-d_6) of compound **49**.

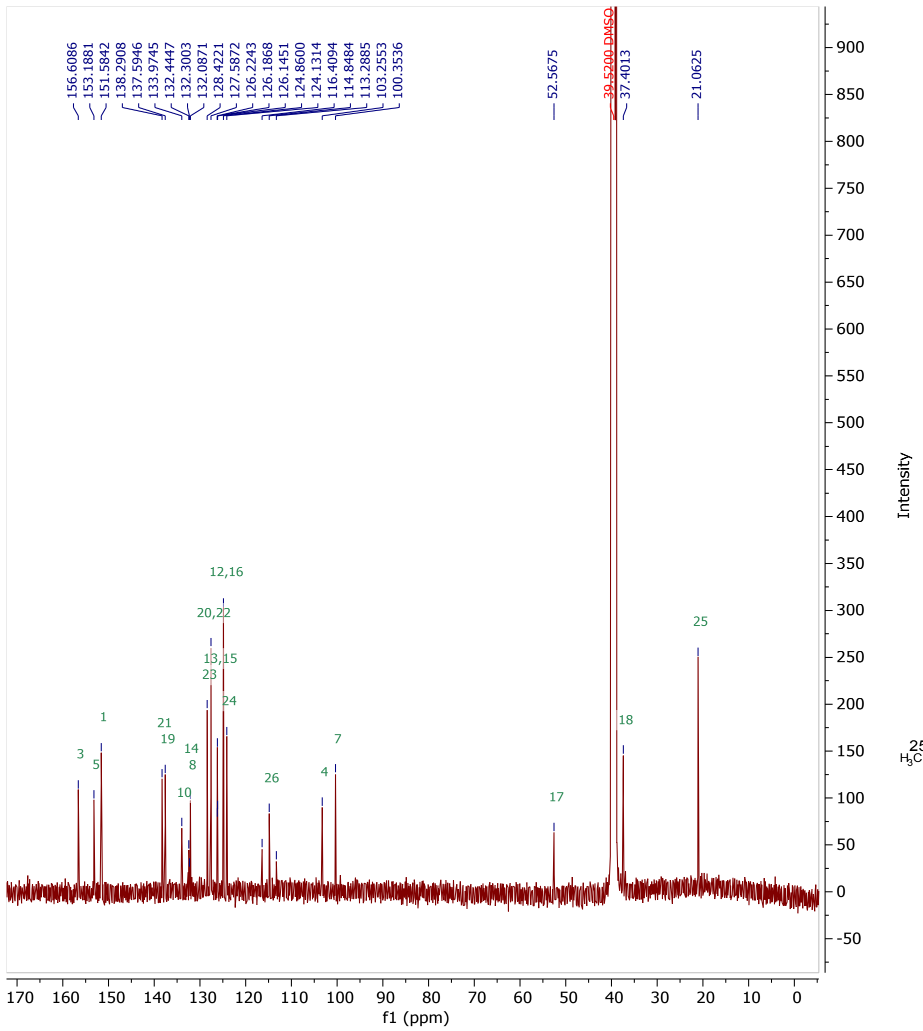

	Parameter	Value
1	Title	FAB-02-13-EKFK.2.fid
2	Origin	Bruker BioSpin GmbH
3	Owner	nmrsu
4	Instrument	spect
5	Solvent	DMSO
6	Temperature	300.0
7	Pulse Sequence	zgpg30
8	Experiment	1D
9	Probe	Z117768_0061 (CP TCI 600S3 H-C/ N-D-05 Z)
10	Number of Scans	2048
11	Receiver Gain	197.1
12	Relaxation Delay	2.0000
13	Pulse Width	11.4000
14	Presaturation Frequency	
15	Acquisition Time	0.9088
16	Acquisition Date	2021-03-23T20:10:25
17	Modification Date	2021-03-23T20:10:25
18	Spectrometer Frequency	150.93
19	Spectral Width	36057.7
20	Lowest Frequency	-3012.4
21	Nucleus	13C
22	Acquired Size	32768
23	Spectral Size	65536
24	Digital Resolution	0.55

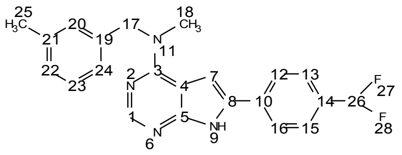

Figure S98. ^{13}C NMR (150 MHz, DMSO-d_6) of compound **49.**

Compound 50

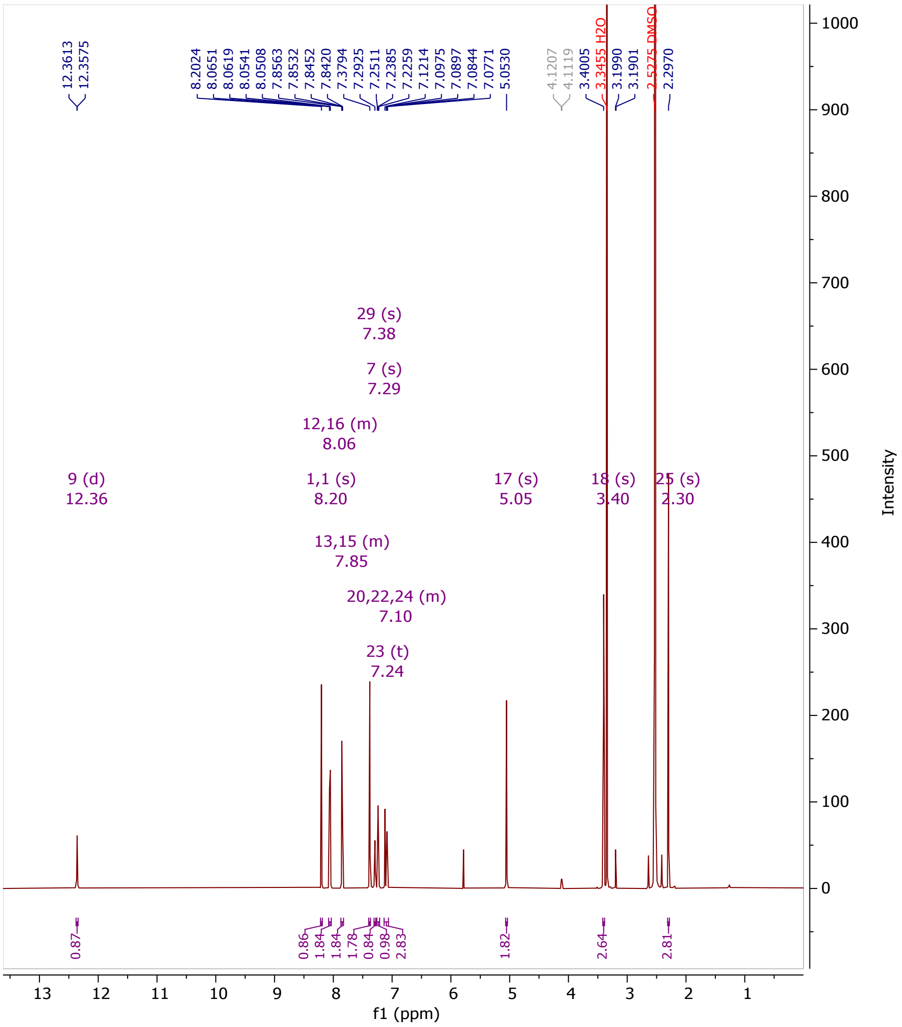

	Parameter	Value
1	Title	FAB-02-15.1.fid
2	Origin	Bruker BioSpin GmbH
3	Owner	nmrsu
4	Instrument	spect
5	Solvent	DMSO
6	Temperature	298.0
7	Pulse Sequence	zg30
8	Experiment	1D
9	Probe	Z117768_0061 (CP TCI 600S3 H-C/ N-D-05 Z)
10	Number of Scans	32
11	Receiver Gain	12.9
12	Relaxation Delay	1.0000
13	Pulse Width	8.0000
14	Presaturation Frequency	
15	Acquisition Time	2.7263
16	Acquisition Date	2021-03-23T22:02:49
17	Modification Date	2021-03-23T22:02:49
18	Spectrometer Frequency	600.18
19	Spectral Width	12019.2
20	Lowest Frequency	-2316.0
21	Nucleus	1H
22	Acquired Size	32768
23	Spectral Size	131072
24	Digital Resolution	0.09

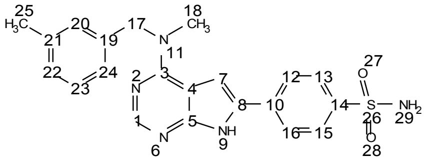

Figure S99. ^{1}H NMR (600 MHz, DMSO-d_6) of compound **50**.

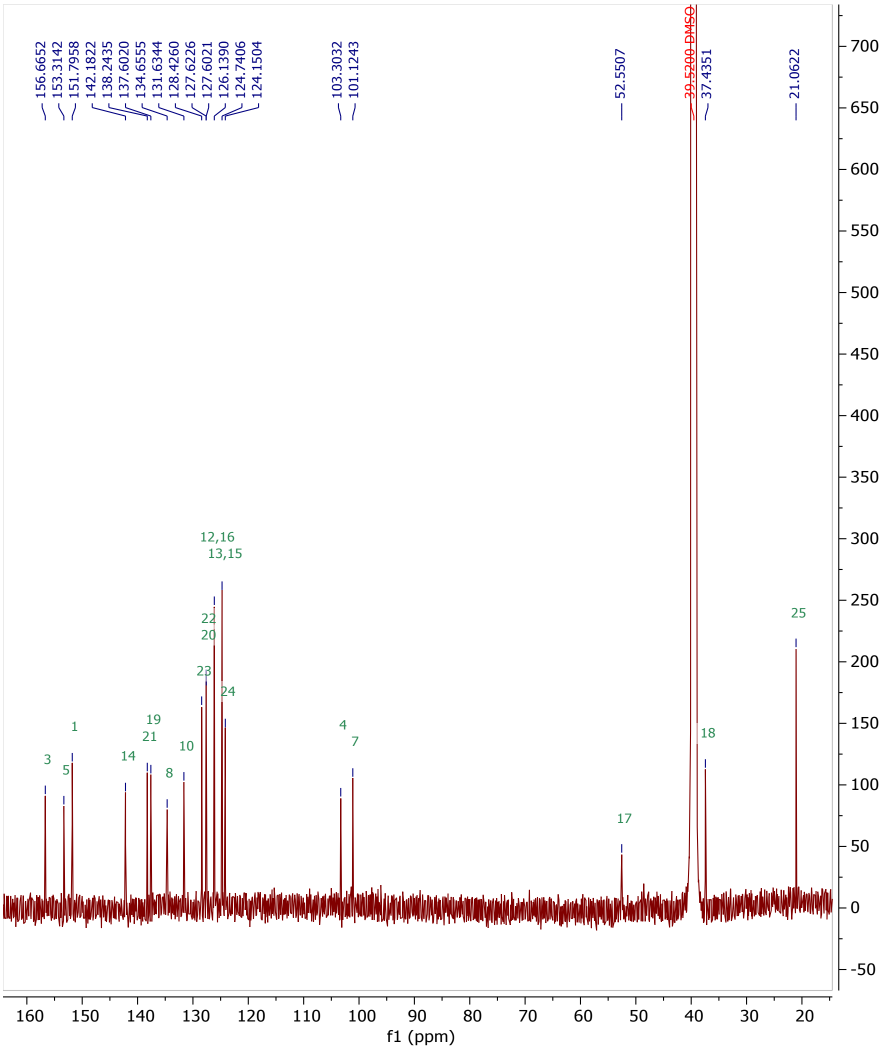

	Parameter	Value
1	Title	FAB-02-15.2.fid
2	Origin	Bruker BioSpin GmbH
3	Owner	nmrsu
4	Instrument	spect
5	Solvent	DMSO
6	Temperature	300.0
7	Pulse Sequence	zgpg30
8	Experiment	1D
9	Probe	Z117768_0061 (CP TCI 600S3 H-C/ N-D-05 Z)
10	Number of Scans	2048
11	Receiver Gain	197.1
12	Relaxation Delay	2.0000
13	Pulse Width	11.4000
14	Presaturation Frequency	
15	Acquisition Time	0.9088
16	Acquisition Date	2021-03-23T23:47:51
17	Modification Date	2021-03-23T23:47:51
18	Spectrometer Frequency	150.93
19	Spectral Width	36057.7
20	Lowest Frequency	-3012.4
21	Nucleus	13C
22	Acquired Size	32768
23	Spectral Size	65536
24	Digital Resolution	0.55

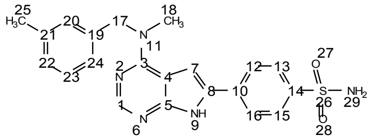

Figure S100. ^{13}C NMR (150 MHz, DMSO-d_6) of compound **50.**

Compound 51

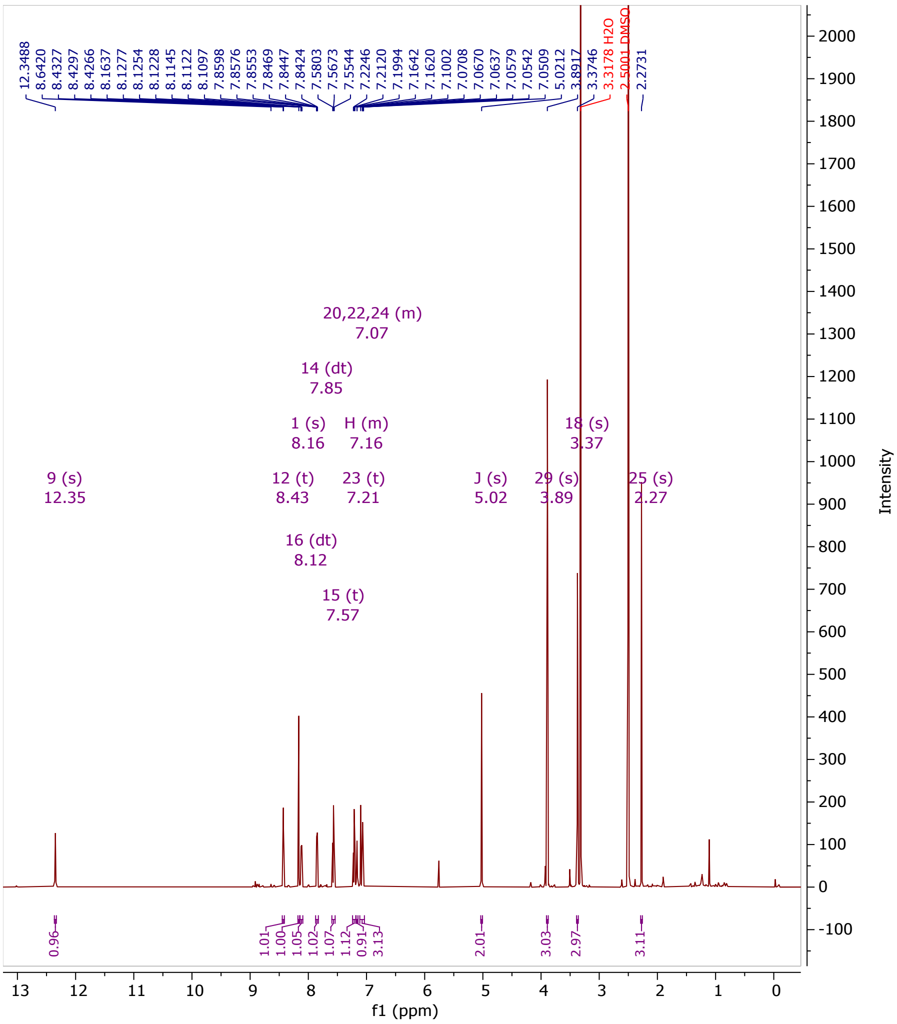

	Parameter	Value
1	Title	FAB02-23.1.fid
2	Origin	Bruker BioSpin GmbH
3	Owner	nmrsu
4	Instrument	spect
5	Solvent	DMSO
6	Temperature	298.0
7	Pulse Sequence	zg30
8	Experiment	1D
9	Probe	Z117768_0061 (CP TCI 600S3 H-C/ N-D-05 Z)
10	Number of Scans	64
11	Receiver Gain	11.5
12	Relaxation Delay	1.0000
13	Pulse Width	8.0000
14	Presaturation Frequency	
15	Acquisition Time	2.7263
16	Acquisition Date	2021-04-24T11:26:23
17	Modification Date	2021-04-24T11:26:23
18	Spectrometer Frequency	600.18
19	Spectral Width	12019.2
20	Lowest Frequency	-2347.3
21	Nucleus	1H
22	Acquired Size	32768
23	Spectral Size	131072
24	Digital Resolution	0.09

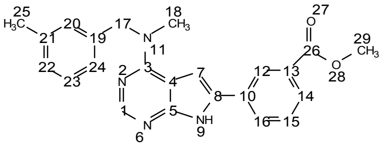

Figure S101. ^{1}H NMR (600 MHz, DMSO-d_6) of compound **51**.

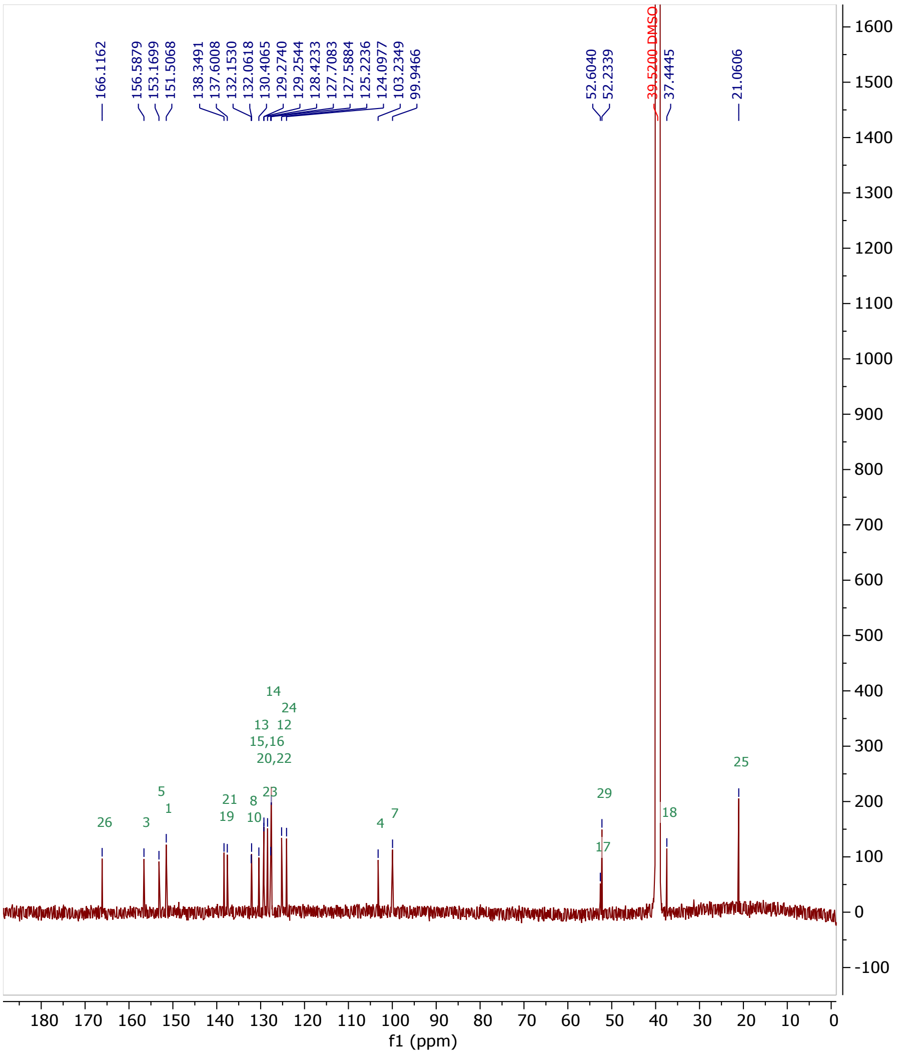

Parameter		Value
1	Title	FAB02-23.2.fid
2	Origin	Bruker BioSpin GmbH
3	Owner	nmrsu
4	Instrument	spect
5	Solvent	DMSO
6	Temperature	300.0
7	Pulse Sequence	zgpg30
8	Experiment	1D
9	Probe	Z117768_0061 (CP TCI 600S3 H-C/ N-D-05 Z)
10	Number of Scans	2048
11	Receiver Gain	197.1
12	Relaxation Delay	2.0000
13	Pulse Width	11.4000
14	Presaturation Frequency	
15	Acquisition Time	0.9044
16	Acquisition Date	2021-04-24T13:10:05
17	Modification Date	2021-04-24T13:10:05
18	Spectrometer Frequency	150.93
19	Spectral Width	36231.9
20	Lowest Frequency	-3098.8
21	Nucleus	13C
22	Acquired Size	32768
23	Spectral Size	65536
24	Digital Resolution	0.55

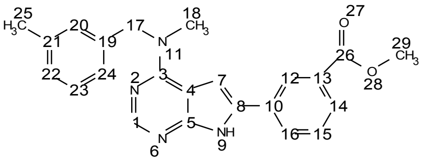

Figure S102. ^{13}C NMR (150 MHz, DMSO-d_6) of compound **51.**

Compound 52

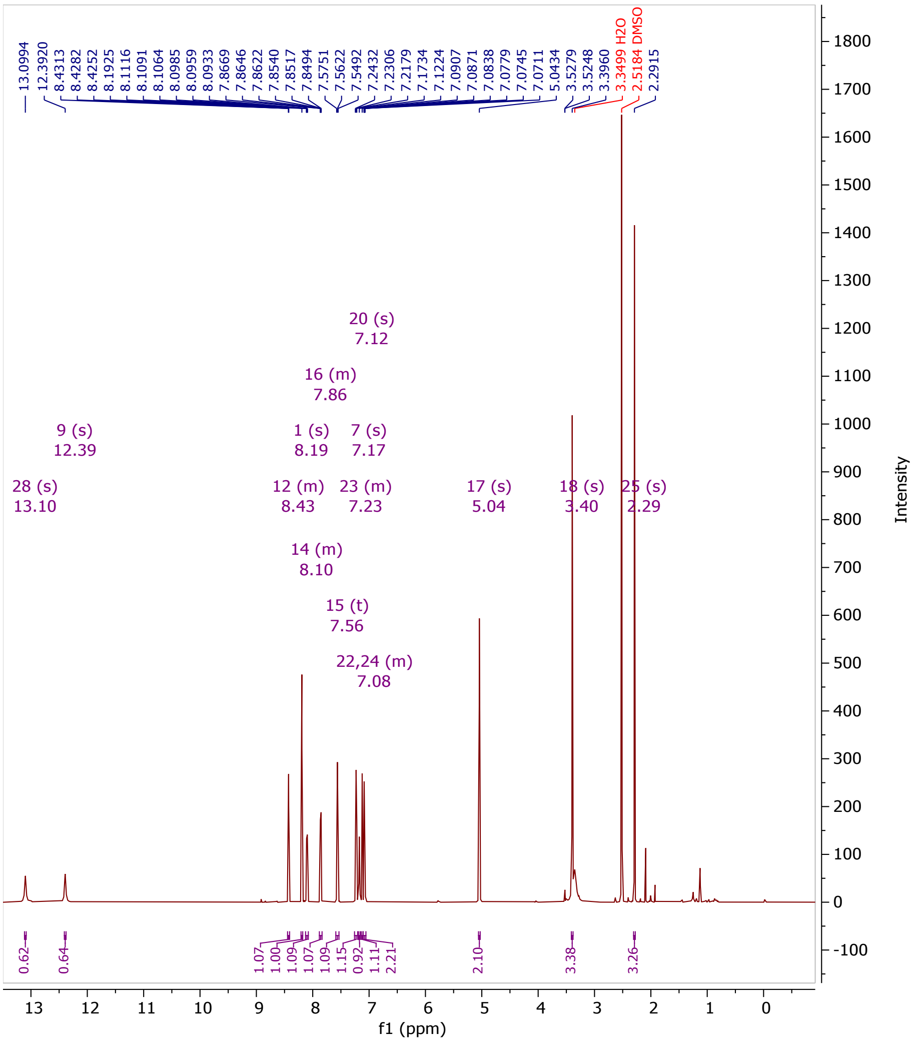

Parameter		Value
1	Title	FAB-02-25.1.fid
2	Origin	Bruker BioSpin GmbH
3	Owner	nmrsu
4	Instrument	spect
5	Solvent	DMSO
6	Temperature	298.0
7	Pulse Sequence	zg30
8	Experiment	1D
9	Probe	Z117768_0061 (CP TCI 600S3 H-C/ N-D-05 Z)
10	Number of Scans	32
11	Receiver Gain	11.5
12	Relaxation Delay	1.0000
13	Pulse Width	8.0000
14	Presaturation Frequency	
15	Acquisition Time	2.7263
16	Acquisition Date	2021-04-28T00:43:06
17	Modification Date	2021-04-28T00:43:06
18	Spectrometer Frequency	600.18
19	Spectral Width	12019.2
20	Lowest Frequency	-2357.5
21	Nucleus	1H
22	Acquired Size	32768
23	Spectral Size	131072
24	Digital Resolution	0.09

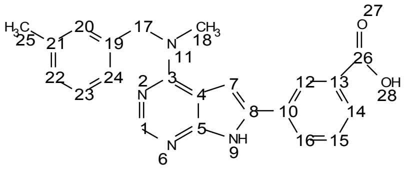

Figure S103. ^{1}H NMR (600 MHz, DMSO-d_6) of compound **52**.

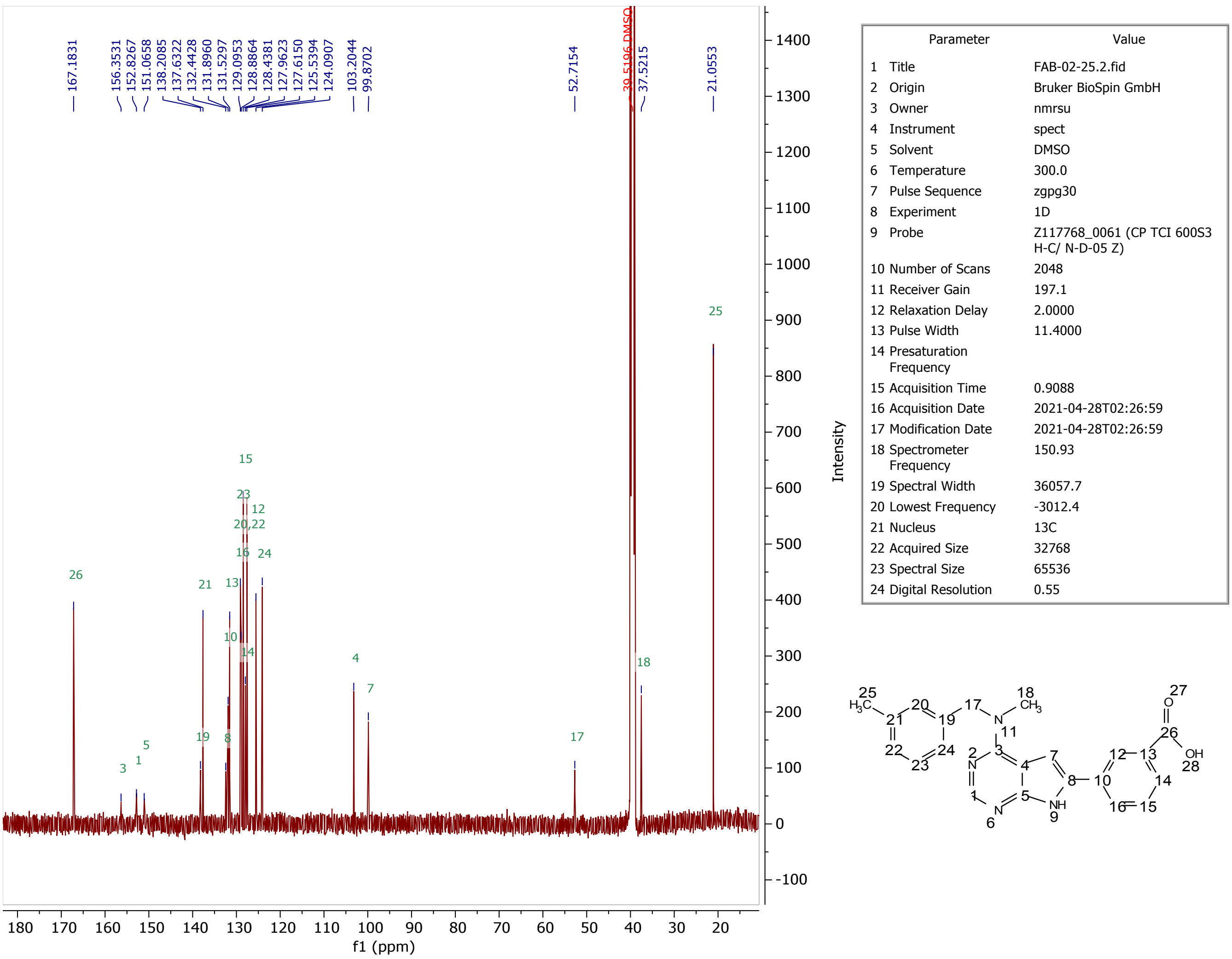

Figure S104. ^{13}C NMR (150 MHz, DMSO-d_6) of compound **52.**

10. Compounds screen for CSF1R activity

Table S6: Structure of compounds screen for CSF1R activity at 500 nM test concentration in duplicates (average values are shown). Most of the compounds were also countered screened towards EGFR at 100 nM test concentration in duplicates. The compounds were from our in-house compound. A reference is provided to those materials which has been published previously, while preparation methods and characterisation of new materials are provided in the following section.

Comp. nr	Structure	CSF1R (%) inhibition	EGFR (%) inhibition	Reference
**S-1**	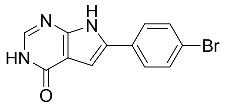	**56**	**2**	**[3]**
**S-2**	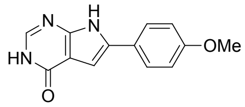	**63**	**ND**	**[3]**
**S-3**	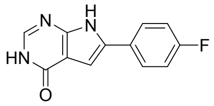	**49**	**ND**	**[3]**
**S-4**	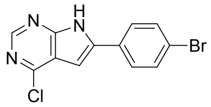	**49**	**ND**	**[3]**
**S-5**	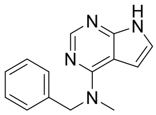	**80**	**13**	**New**
**S-6**	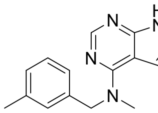	**87**	**ND**	**New**
**S-7**	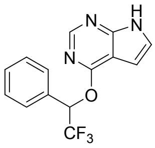	**37**	**-9**	**New**
**S-8**	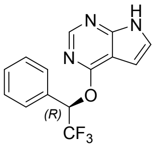	**21**	**-14**	**[4]**
**S-9**	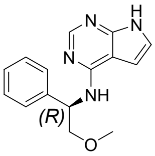	**19**	**-10**	**New**
**S-10**	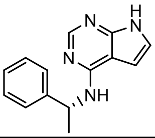	**37**	**65**	**New**
**S-11**	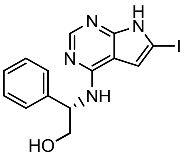	**41**	**73**	**[5]**
**S-12**	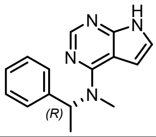	**60**	**ND**	**New**
**S-13**	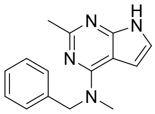	**6**	**ND**	**New**

Comp. nr	Structure	CSF1R (%) inhibition	EGFR (%) inhibition	Reference
**S-14**	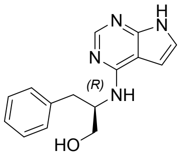	**13**	**-6**	**New**
**S-15**	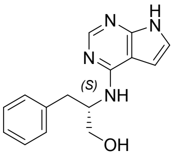	**18**	**3**	**New**
**S-16**	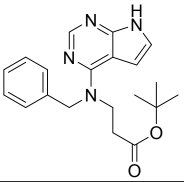	**16**	**ND**	**New**
**S-17**	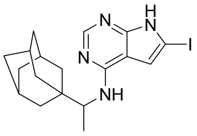	**0**	**4**	**[4]**
**S-18**	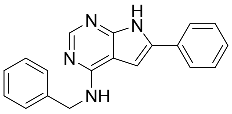	**83**	**97**	**[5]**
**S-19**	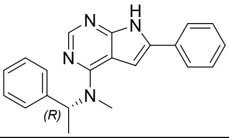			**New**
**S-20**	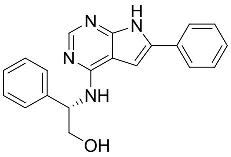	**88**	**96**	**[5]**
**S-21**	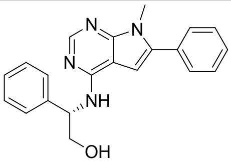	**15**	**8**	**[2]**
**S-22**	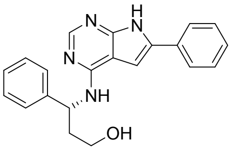	**74**	**99**	**[2]**
**S-23**	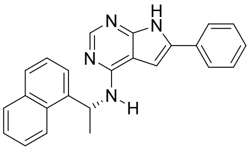	**56**	**ND**	**[6]**
**S-24**	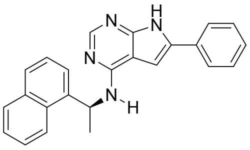	**19**	**3**	**[6]**

Comp. nr	Structure	CSF1R (%) inhibition	EGFR (%) inhibition	Reference
**S-25**	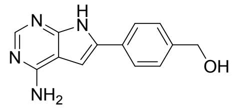	**88**	**9**	**New**
**S-26**	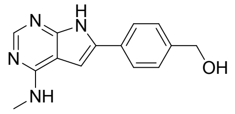	**97**	**30**	**New**
**S-27**	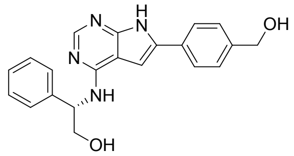	**93**	**97.5**	**[5]**
**S-28**	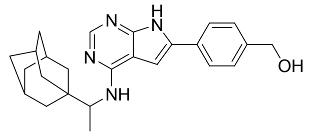	**32**	**7**	**[7]**
**S-29**	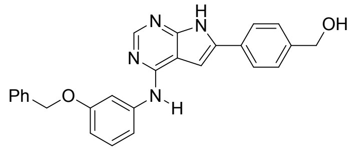	**41**	**3**	**[7]**
**S-30**	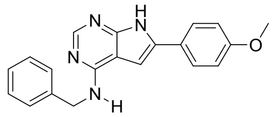	**95**	**95**	**[5]**
**S-31**	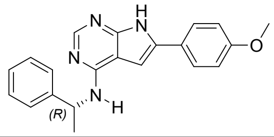	**71**	**95**	**[3]**
**S-32**	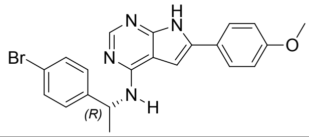	**42**	**63**	**[5]**
**S-33**	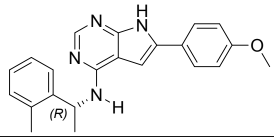	**61**	**91**	**[5]**
**S-34**	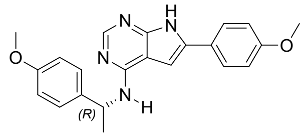	**40**	**9**	**[5]**
**S-35**	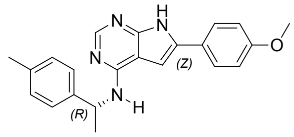	**47**	**44**	**[5]**
**S-36**	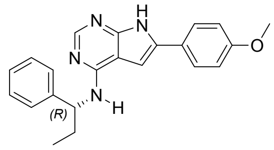	**58**	**99**	**[5]**

Comp. nr	Structure	CSF1R (%) inhibition	EGFR (%) inhibition	Reference
S-37	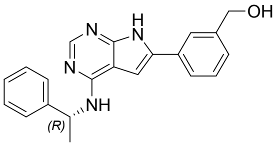	88	101	[5]
S-38	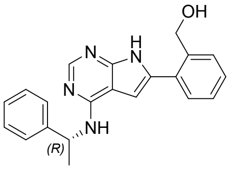	82	101	[5]
S-39	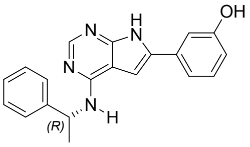	81	94	[5]
S-40	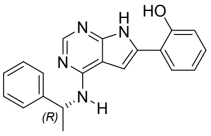	80	97	[2]
S-41	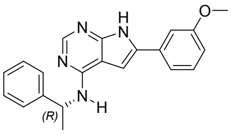	89	96	[5]
S-42	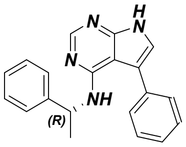	16	73	[8]
S-43	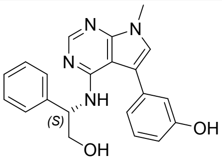	7	76	[7]
S-44	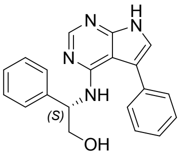	25	99	[8]
S-45	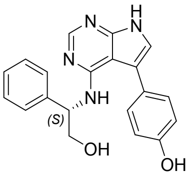	39	100	[8]
S-46	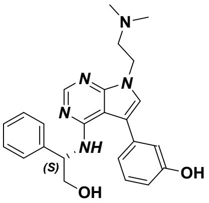	-2	-5	[7]

Comp. nr	Structure	CSF1R (%) inhibition	EGFR (%) inhibition	Reference
**S-47**	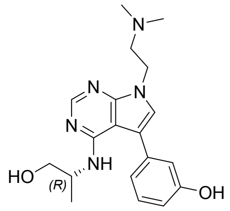	**4**	**-8**	**[7]**
**S-48**	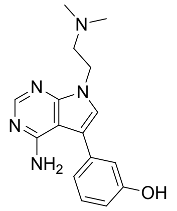	**28**	**0**	**[7]**
**S-49**	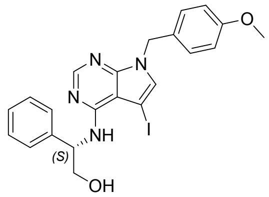	**9**	**-5**	**[8]**
**S-50**	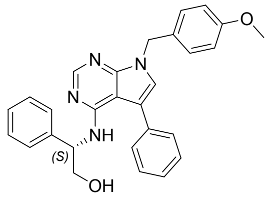	**2**	**4**	**New**
**S-51**	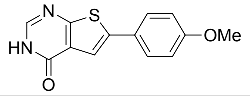	**12**	**ND**	**[9]**
**S-52**	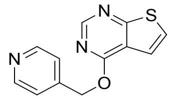	**25**	**-4**	**New**
**S-53**	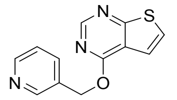	**19**	**-3**	**New**
**S-54**	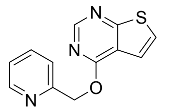	**20**	**-2**	**New**
**S-55**	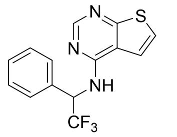	**-2**	**3**	**New**
**S-56**	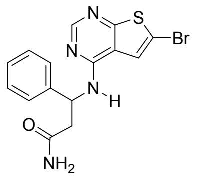	**5**	**53**	**[7]**
**S-57**	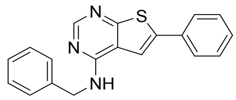	**19**	**17**	**New**

Comp. nr	Structure	CSF1R (%) inhibition	EGFR (%) inhibition	Reference
**S-58**	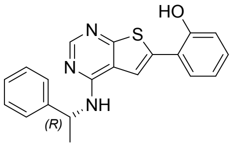	**9**	**79**	**[10]**
**S-59**	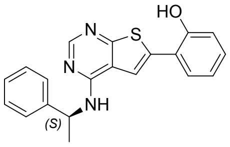	**0**	**1**	**[10]**
**S-60**	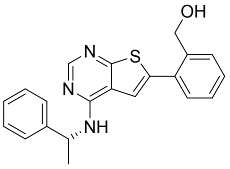	**9**	**70**	**[10]**
**S-61**	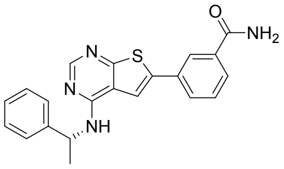	**-2**	**85.5**	**[11]**
**S-62**	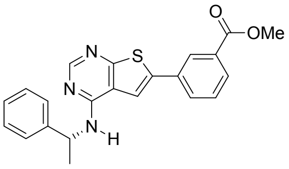	**23**	**70**	**New**
**S-63**	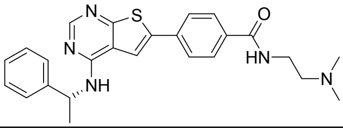	**8**	**93**	**[11]**
**S-64**	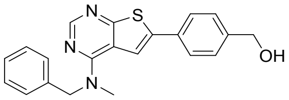	**62**	**40**	**New**
**S-65**	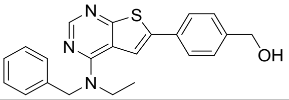	**3**	**8**	**New**
**S-66**	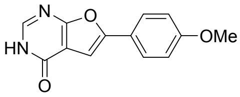	**32**	**ND**	**[12]**
**S-67**	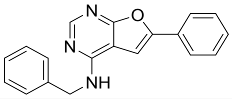	**10**	**60**	**[12]**
**S-68**	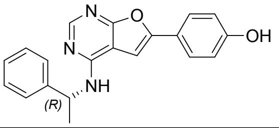	**29**	**94**	**[12]**
**S-69**	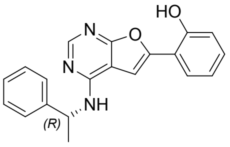	**29**	**96**	**[12]**

Comp. nr	Structure	CSF1R (%) inhibition	EGFR (%) inhibition	Reference
S-70	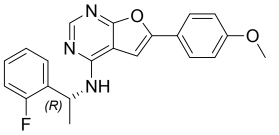	23	68	[12]
S-71	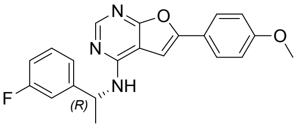	12	70	[12]
S-72	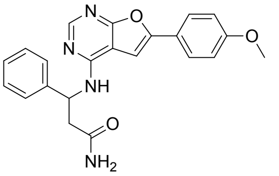	74	78	[12]
S-73	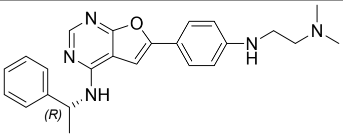	74	101	[12]
S-74	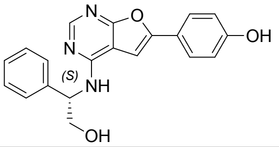	31	99	[12]
S-75	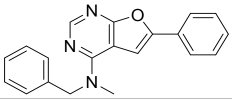	44	5	New
S-76	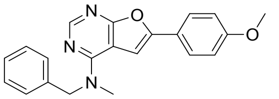	62	12	New
S-77	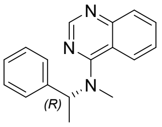	8	ND	New
S-78	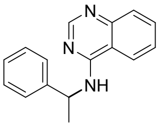	7	ND	New
S-79	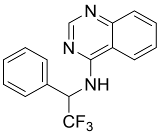	0	ND	New

11. Synthetic protocols for compounds in the CSF1R screen

General Procedure A-Amination of protected pyrrolopyrimidines

4-Chloro-6-iodo-7-((2-(trimethylsilyl)-ethoxy)methyl)-7*H*-pyrrolo[2,3-*d*]pyrimidine (1.00 g, 1 equiv.) was dissolved in dry *n*-BuOH or dioxane (10 mL), added the benzylamine (1.5-3 equiv.) and optionally *N,N*-diisopropylethylamine (3 equiv.). The reaction was stirred at 100-140 ^{o}C for 4-24 h. Following evaporation of solvent, the residue is added water (20 mL) and EtOAc (50 mL). After phase separation the water phase is extracted with more EtOAc (3 × 50 mL). The combined organic phase is then dried over $MgSO_4$ and concentrated at low pressure. The products were purified by silica gel column chromatography as specified below.

General procedure B-*Suzuki-cross coupling of aminated pyrrolopyrimidines*

The 4-amino-6-iodo-7-((2-(trimethylsilyl)-ethoxy)methyl)-7*H*-pyrrolo[2,3-*d*]pyrimidine (1.0 equiv.), aryl boronic acid or pinacol ester (1.0 - 1.2 equiv.), $PdCl_2$dppf (2 - 5 mol%) and potassium carbonate (3.0 equiv.) are charged in an appropriate reaction vessel. The atmosphere is evacuated and back-filled with N_2 three times before adding degassed 1,4-dioxane (6 mL/mmol starting material) and degassed water (3 mL/mmol starting material). The reaction vessel is lowered into an oil-bath set at 60 - 80 ^{0}C and stirred vigorously. Upon reaction completion, the reaction vessel is raised from the oil-bath and allowed to cool for 5 min. before the reaction mixture is transferred to a round-bottomed flask and the volatiles are removed by rotary evaporation. The residue is added water (20 mL/mmol starting material) and extracted with CH_2Cl_2 (3 x 20 mL/mmol starting material). The combined organic layers are washed with brine (20 mL/mmol), dried with anhydrous Na_2SO_4 and filtered. The organic solvent is removed under reduced pressure and the crude product is purified by silica-gel column chromatography. Some transformations were performed with alternative catalyst. This is specified.

General Procedure C-SEM-deprotection

The SEM-protected pyrrolopyrimidine (0.2 mmol, 1 equiv.) was stirred in TFA (2 mL) and CH_2Cl_2 (10 mL) at 50 °C for 3-24 h.. The reaction mixture was then concentrated in *vacuo* before it was taken up in MeOH (10 mL) and NH_3 (20 mL, 25% aqueous) and stirred for 2-24 h at 22 °C. The reaction mixture was concentrated in *vacuo*, and the crude product was purified with silica-gel column chromatography. For some compounds the last step of the procedure was run with $NaHCO_3$ instead of ammonia and THF instead of MeOH.

N-Benzyl-N-methyl-7H-pyrrolo[2,3-d]pyrimidin-4-amine (S-5)

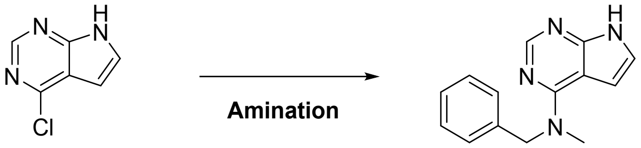

4-Chloro-7H-pyrrolo[2,3-d]pyrimidine (307 mg, 1.99 mmol) and N-methyl-benzylamine (770 µL, 5.97 mmol) were dissolved in n-BuOH (2.0 mL), stirred at reflux for 2.5 h under an atmosphere of nitrogen before the mixture was cooled and the solvent removed under reduced pressure. The residue was partitioned between CH_2Cl_2 (10 mL) and aq. HCl (0.1 M, 20 mL). The layers were separated, and the aqueous phase extracted using CH_2Cl_2 (2 × 10 mL). The combined organic phases were washed with sat. aq. $NaHCO_3$ (20 mL), brine (15 mL), dried (Na_2SO_4), filtered and evaporated under reduced pressure. The crude product was purified by silica-gel column chromatography (CH_2Cl_2/MeOH - 94:6, R_f = 0.19) giving 347 mg (1.458 mmol, 73%) of a white powder; mp. 231.5 - 232.5 ˚C; ^{1}H NMR (600 MHz, DMSO-d_6) δ 11.64 (s, 1H), 8.13 (s, 1H), 7.35 – 7.29 (m, 2H), 7.27 – 7.21 (m, 3H), 7.12 – 7.08 (m, 1H), 6.51 – 6.47 (m, 1H), 5.01 (s, 2H), 3.31 (s, 3H); ^{13}C NMR (151 MHz, DMSO-d_6) δ 156.8, 151.7, 150.8, 138.5, 128.5 (2C), 126.9 (2C), 126.8, 120.8, 101.8, 101.4, 52.7, 37.2; IR (neat, cm^{-1}): 3193 (w), 3097 (w), 2985 (w), 2960 (w), 2847 (w), 1586 (m), 1562 (s), 1512 (m), 1493 (m), 1409 (s), 1321 (m), 1254 (m), 1059 (m), 734 (s); HRMS (ES+, m/z): found 239.1302, calcd for $C_{14}H_{15}N_4$, $[M+H]^+$, 239.1297.

N-Methyl-N-(3-methylbenzyl)-7H-pyrrolo[2,3-d]pyrimidin-4-amine (S-6)

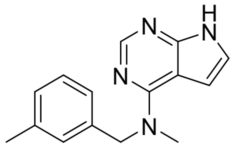

4-Chloro-7H-pyrrolo[2,3-d]pyrimidine (155 mg, 1.01 mmol), N-methyl-1-(m-tolyl)methanamine (200 µL, 1.33 mmol) and N,N-diisopropylethylamine (350 µL, 2.05 mmol) were dissolved in n-BuOH (1.0 mL), stirred at reflux for 3.5 h under an atmosphere of nitrogen before the mixture was cooled and the solvent removed under reduced pressure. The residue was partitioned between CH_2Cl_2 (10 mL) and water (15 mL). The layers were separated, and the aqueous phase extracted using CH_2Cl_2 (2 × 10 mL). The combined organic phases were washed with brine (15 mL), dried (Na_2SO_4), filtered and evaporated under reduced pressure. The crude product was purified by silica-gel column chromatography (CH_2Cl_2/MeOH - 96:4,

R_f = 0.13) giving 195 mg (0.772 mmol, 77%) of an off-white powder, mp. 190 - 194 °C; ^{1}H NMR (400 MHz, DMSO-d_6) δ 11.64 (s, 1H), 8.12 (s, 1H), 7.24 – 7.16 (m, 1H), 7.13 – 7.08 (m, 1H), 7.08 – 6.99 (m, 3H), 6.48 (s, 1H), 4.96 (s, 2H), 3.29 (s, 3H), 2.26 (s, 3H); ^{13}C NMR (101 MHz, DMSO-d_6) δ 156.8, 151.7, 150.8, 138.4, 137.6, 128.4, 127.5, 127.4, 123.9, 120.8, 101.8, 101.4, 52.7, 37.2, 21.1; IR (neat, cm^{-1}): 3194 (w), 3095 (w), 2985 (w), 2959 (w), 2865 (w), 1592 (m), 1567 (s), 1492 (m), 1407 (s), 1345 (m), 1317 (m), 1300 (m), 1252 (m), 1062 (m), 811 (m), 733 (s), 608 (m); HRMS (ES+, m/z): found 253.1458, calcd for $C_{15}H_{17}N_4$, $[M+H]^+$, 253.1453.

(*rac*)-4-(2,2,2-Trifluoro-1-phenylethoxy)-7-((2-(trimethylsilyl)ethoxy)methyl)-7*H*-pyrrolo[2,3-*d*]pyrimidine (S-7)

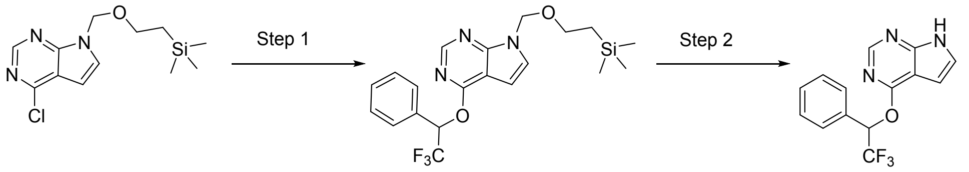

Step 1: 4-(2,2,2-trifluoro-1-phenylethoxy)-7-((2-(trimethylsilyl)-ethoxy)methyl)-7H-pyrrolo-[2,3-*d*]pyrimidine

4-Chloro-7-((2-(trimethylsilyl)ethoxy)methyl)-7*H*-pyrrolo-[2,3-*d*]pyrimidine [2] (300 mg, 1.06 mmol) dissolved in acetonitrile (3 mL) was added to a mixture of Cs_2CO_3 (448 mg, 1.37 mmol, 1.3 equiv.) and 2,2,2-trifluoro-1-phenylethan-1-ol (1.37 mmol, 1.3 equiv.) in acetonitrile (2 mL). The reaction mixture was stirred at reflux for 4 h. The solvent was removed in vacuo, and the residue dissolved in EtOAc (25 mL), extracted with water (3 × 10 mL) and back-extracted with EtOAc (4 × 7.5 mL). The combined organic fractions were washed with sat. aq. $NaHCO_3$ (15 mL) and brine (2 × 10 mL), dried over anhydrous Na_2SO_4, filtered and concentrated in vacuo. Purification by column chromatography with silica-gel (*n*-pentane/EtOAc - 4:1, R_f = 0.53), yielded 327 mg (0.77 mmol, 73%) as a clear oil; ^{1}H NMR (400 MHz, DMSO-d_6) δ 8.42 (s, 1H), 7.67 - 7.65 (m, 3H), 7.47 - 7.42 (m, 3H), 7.10 (q, J = 7.0 Hz, 1H), 6.76 (d, J = 3.6 Hz, 1H), 5.60 (s, 2H), 3.49 (t, J = 8.0 Hz, 2H), 0.80 (t, J = 8.1 Hz, 2H), -0.13 (s, 9H); ^{13}C NMR (100 MHz, DMSO-d_6) δ 159.5, 152.7, 150.1, 131.6, 129.7, 128.8 (2C), 128.7, 127.8 (2C), 123.7 (q, J = 281.8 Hz), 104.8, 98.2, 72.7, 72.3 (q, J = 32.0 Hz), 65.6, 17.1, -1.5 (3C); ^{19}F NMR (564 MHz, DMSO-d_6, decoupled) δ -77.2; IR (neat, cm^{-1}): 2954, 1602, 1262, 1131, 1061, 699; HRMS (ASAP+, m/z): found 424.1673, calcd for $C_{20}H_{25}N_3O_2F_3Si$, $[M+H]^+$, 424.1668.

Step 2: (*rac*)-4-(2,2,2-trifluoro-1-phenylethoxy)-7*H*-pyrrolo-[2,3-*d*]pyrimidine (S-7)

(*rac*)-4-(2,2,2-Trifluoro-1-phenylethoxy)-7-((2-(trimethylsilyl)ethoxy)-methyl)-7*H*-pyrrolo-[2,3-*d*]pyrimidine (123 mg, 0.29 mmol) was dissolved in acetonitrile (2.5 mL) and cooled in an ice bath. Boron trifluoride diethyl etherate (0.72 mmol, 2.5 equiv.) was added dropwise over 30 min, and the mixture allowed to reach at 22 °C for 2 h. After cooling in an ice bath, water (0.8 mL) was added. This was stirred at 22 °C for 1 h, before cooling and dropwise addition of ammonium (1.5 mL 8.3% aq). After 2 h of stirring, EtOAc (25 mL) was added, and the reaction quenched with sat. aq. $NaHCO_3$ (2 × 10 mL) and water (2 × 10 mL). The aqueous phase was back-extracted with EtOAc (3 × 15 mL). Drying the combined organic fractions with brine (20 mL) and anhydrous Na_2SO_4, filtration and solvent evaporation gave a white solid raw product. Purification by column chromatography with silica-gel (*n*-pentane/EtOAc - 3:2, R_f = 0.26), yielded 58 mg (0.20 mmol, 69%) as a white solid, mp. 152 °C; HPLC purity: 98% (Method A); ^{1}H NMR (400 MHz, DMSO-d_6) δ 12.25 (s, 1H), 8.34 (s, 1H), 7.67 - 7.65 (m, 2H), 7.49-7.48 (m, 1H), 7.47 - 7.41 (m, 3H), 7.09 (q, *J* = 7.1 Hz, 1H), 6.66 (d, *J* = 3.5 Hz, 1H); ^{13}C NMR (100 MHz, DMSO-d_6) δ 159.3, 153.2, 149.6, 131.7, 129.7, 128.7 (2C), 127.8 (2C), 125.5, 123.8 (q, *J* = 280.8 Hz), 104.4, 97.6, 72.0 (q, *J* = 31.8 Hz); ^{19}F NMR (564 MHz, DMSO-d_6, decoupled) δ -77.2; IR (neat, cm^{-1}): 3110 (w, br), 2990 (w, br), 2846 (w, br), 1614 (m), 1580 (m), 1492 (m), 1360 (m), 1318 (m), 1258 (m), 1127 (s), 1060 (s), 1006 (m), 892 (s), 746 (s), 701 (s), 605 (s); HRMS (ASAP+, m/z): found 294.0853, calcd for $C_{14}H_{11}N_3OF_3$,$[M+H]^+$, 294.0854.

(*R*)-*N*-(2-Methoxy-1-phenylethyl)-7*H*-pyrrolo[2,3-*d*]pyrimidin-4-amine (S-9)

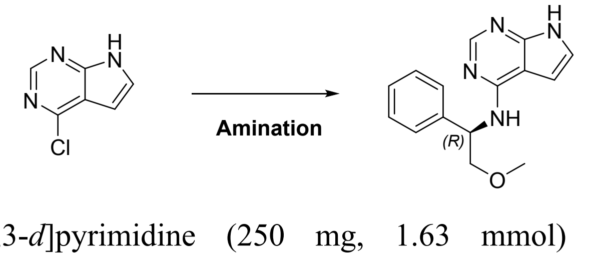

4-Chloro-7*H*-pyrrolo[2,3-*d*]pyrimidine (250 mg, 1.63 mmol) and (*R*)-2-methoxy-1-phenylethan-1-amine (738 mg, 4.88 mmol, 3 equiv.) were dissolved in *n*-BuOH (5 mL), under an N_2-atmosphere, and stirred at reflux for 28 h, before the mixture was cooled and the solvent removed under reduced pressure. Water (25 mL) was added to the mixture was extracted with EtOAc (3 × 25 mL). The combined organic fractions were washed with brine (25 mL), 5 M NaOH (15 mL) and brine (10 mL) and dried over Na_2SO_4 followed by solvent evaporation. Purification by silica-gel column chromatography (EtOAc/MeOH - 19:1, R_f = 0.24) yielded

282 mg (1.05 mmol, 65%) of an off-white solid, mp. 69-71 °C; $[\alpha]_D^{20}$ = +131.3 (c 1.02, DMSO). HPLC purity: 99% (Method A); ^{1}H NMR (400 MHz, DMSO-d_6) δ 11.49 (s, 1H), 8.04 (s, 1H), 7.73 (m, 1H), 7.46 - 7.44 (m, 2H), 7.32 - 7.29 (m, 2H), 7.23 - 7.20 (m, 1H), 7.09 - 7.08 (m, 1H), 6.71 - 6.70 (m, 1H), 5.65 - 5.64 (m, 1H), 3.75 - 3.60 (m, 2H), 3.30 (s, 3H); ^{13}C NMR (100 MHz, DMSO-d_6) δ 155.6, 151.2, 150.3, 141.5, 128.1 (2C), 127.0 (2C), 126.8, 120.8, 102.6, 98.8, 75.1, 58.0, 52.6; IR (neat, cm^{-1}): 3118 (w), 2981 (w), 2867 (w), 1600 (m), 1577 (s), 1470 (m), 1347 (m), 1309 (m), 1101 (s), 882 (m), 725 (m), 698 (s), 602 (m); HRMS (ASAP+, m/z): found 269.1401, calcd for $C_{15}H_{17}N_4O$, $[M+H]^+$, 269.1402.

(*R*)-*N*-(1-Phenylethyl)-7*H*-pyrrolo[2,3-*d*]pyrimidin-4-amine (S-10)

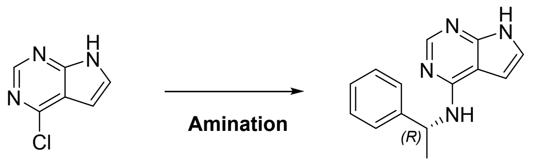

4-Chloro-7*H*-pyrrolo[2,3-*d*]pyrimidine (200 mg, 1.31 mmol) and (*R*)-1-phenylethan-1-amine (475 mg, 3.92 mmol) were added *n*-BuOH (10 mL) and reacted at 145 °C for 18 h. The solution was cooled to 22 °C and water (30 mL) was added, the reaction mixture was extracted with EtOAc (3 × 50 mL), dried over Na_2SO_4 and concentrated *in vacuo*. Column chromatography on silica-gel (EtOAc/*n*-pentane - 9:1, R_f = 0.21) gave 220 mg (0.93mmol, 71%) of a white solid, mp. 146-149 °C; HPLC purity: 98%; ^{1}H NMR (400 MHz, DMSO-d_6) δ 11.47 (s, 1H), 8.03 (s, 1H), 7.71 - 7.69 (m, 1H), 7.42 - 7.41 (m, 2H), 7.31 - 7.27 (m, 2H), 7.20-7.17 (m, 1H), 7.07 - 7.06 (m, 1H), 6.64 (s, 1H), 5.53 - 5.46 (m, 1H), 1.52 (d, J = 7.0 Hz, 3H); ^{13}C NMR (100 MHz, DMSO-d_6) δ 155.7, 151.8, 150.7, 146.1, 128.6 (2C), 126.8, 126.5 (2C), 121.2, 103.1, 99.3, 49.1, 23.3; HRMS (APCI/ASAP, m/z): found 239.1296, calcd for $C_{14}H_{15}N_4$, $[M+H]^+$, 239.1297.

(*R*)-*N*-Methyl-*N*-(1-phenylethyl)-7*H*-pyrrolo[2,3-*d*]pyrimidin-4-amine (S-12)

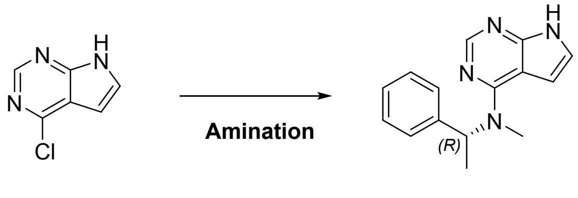

4-Chloro-7*H*-pyrrolo[2,3-*d*]pyrimidine (300 mg, 1.95 mmol) and (*R*)-*N*-methyl-1-phenylethan-1-amine (790 mg, 5.84 mmol) were added *n*-BuOH (10 mL) and reacted at 145

°C for 27 h under a N_2 atmosphere. The solution was cooled to 22 °C and water (30 mL) was added, the reaction mixture was extracted with EtOAc (3 × 50 mL), dried over Na_2SO_4 and concentrated *in vacuo*. Column chromatography on silica-gel (EtOAc, R_f = 0.31) gave 290 mg (1.15 mmol, 59%) of a brown solid, mp. 139-141 °C; $[\alpha]_D^{20}$ = +151.2 (c 1.02, DMSO); ^1H NMR (400 MHz, DMSO-d_6) δ 11.65 (s, 1H), 8.14 (s, 1H), 7.38 - 7.24 (m, 5H), 7.15 - 7.09 (m, 1H), 6.58 - 6.54 (m, 1H), 6.46 - 6.36 (m, 1H), 3.01 (s, 3H), 1.57 (d, J = 7.0 Hz, 3H); ^{13}C NMR (100 MHz, DMSO-d_6) δ 159.6, 151.8, 150.8, 141.6, 128.4 (2C), 126.9, 126.8 (2C), 120.7, 102.1, 101.7, 52.0, 31.5, 16.3; IR (neat, cm^{-1}): 3184, 3080, 2971, 2862, 1560, 1488, 1412, 1309, 1056, 730, 686; HRMS (ASAP+, m/z): found 253.1456, calcd for $C_{15}H_{17}N_4$, $[M+H]^+$, 253.1453.

***N*-Benzyl-*N*,2-dimethyl-7*H*-pyrrolo[2,3-*d*]pyrimidin-4-amine (S-13)**

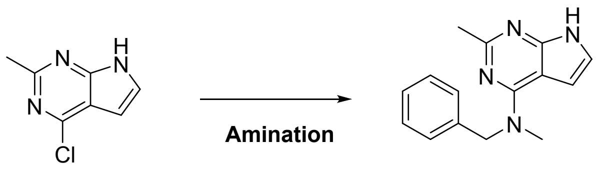

4-Chloro-2-methyl-7*H*-pyrrolo[2,3-*d*]pyrimidine (167 mg, 0.993 mmol) and *N*-methyl-benzylamine (385 µL, 2.98 mmol) were dissolved in *n*-BuOH (1.0 mL), stirred at reflux for 2 h under an atmosphere of nitrogen before the mixture was cooled and the solvent removed under reduced pressure. The residue was partitioned between CH_2Cl_2 (10 mL) and water (15 mL). The layers were separated and the aqueous phase extracted using CH_2Cl_2 (2 × 10 mL). The combined organic phases were washed with brine (15 mL), dried (Na_2SO_4), filtered and evaporated under reduced pressure. The crude product was purified by silica-gel column chromatography (CH_2Cl_2/MeOH - 96:4, R_f = 0.29) giving 156 mg (0.617 mmol, 62%) of an off-white powder, mp. 216 - 217.5 ˚C. ^1H NMR (400 MHz, DMSO-d_6) δ 11.41 (s, 1H), 7.36 – 7.28 (m, 2H), 7.28 – 7.19 (m, 3H), 7.02 – 6.98 (m, 1H), 6.42 – 6.39 (m, 1H), 4.99 (s, 2H), 3.27 (s, 3H), 2.38 (s, 3H); ^{13}C NMR (101 MHz, DMSO-d_6) δ 158.8, 156.7, 152.8, 138.6, 128.5 (2C), 127.0 (2C), 126.8, 120.1, 101.2, 99.5, 52.5, 36.9, 25.7; IR (neat, cm^{-1}): 3197 (w), 3081 (w), 3029 (w), 2970 (w), 2829 (w), 2715 (w), 2661 (w), 1554 (s), 1493 (m), 1418 (m), 1401 (s), 1354 (m), 1333 (m), 1266 (m), 963 (m), 807 (s), 729 (s), 713 (s), 695 (s), 664 (m), 594 (m), 463 (m); HRMS (ES+, m/z): found 253.1458, calcd for $C_{15}H_{17}N_4$, $[M+H]^+$, 253.1453.

(*R*)-2-((7*H*-Pyrrolo[2,3-*d*]pyrimidin-4-yl)amino)-3-phenylpropan-1-ol (S-14)

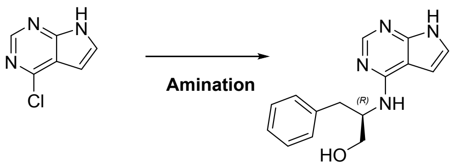

4-Chloro-7*H*-pyrrolo[2,3-*d*]pyrimidine (237 mg, 1.55 mmol) and (*R*)-2-amino-3-phenylpropan-1-ol (701 mg, 4.64 mmol) were dissolved in 1,4-dioxane (4 mL), stirred at reflux for 45 h. under an N_2 atmosphere, before the mixture was cooled and the solvent removed under reduced pressure. The residue was purified by silica-gel column chromatography (CH_2Cl_2/MeOH - 9:1, R_f = 0.24). The crude product was further purified by recrystallization from water giving 243 mg (0.91 mmol, 58%) of an off-white solid; mp. 216-219 °C (dec.); $[\alpha]_D^{20}$ = +142.9 (*c* 1.00, CH_2Cl_2/MeOH); ^{1}H NMR (400 MHz, DMSO-d_6) δ 11.44 (s, 1H), 8.05 (s, 1H), 7.29 - 7.28 (m, 2H), 7.24 - 7.21 (m, 2H), 7.14 - 7.11 (m, 2H), 7.03 (m, 1H), 6.59 (m, 1H), 4.87 (t, *J* = 5.5 Hz, 1H), 4.50 - 4.42 (m, 1H), 3.57 - 3.52 (m, 1H), 3.47 - 3.42 (m, 1H), 3.03 - 2.98 (m, 1H), 2.88 - 2.82 (m, 1H); ^{13}C NMR (100 MHz, DMSO-d_6) δ 155.9, 151.3, 150.1, 139.8, 129.1, 128.1, 125.8, 120.6, 102.6, 98.7, 62.8, 53.4, 36.7; HRMS (ASAP+, m/z): found 269.1400, calcd for $C_{15}H_{17}N_4O$, $[M+H]^+$, 269.1402.

(*S*)-2-((7*H*-Pyrrolo[2,3-*d*]pyrimidin-4-yl)amino)-3-phenylpropan-1-ol (S-15)

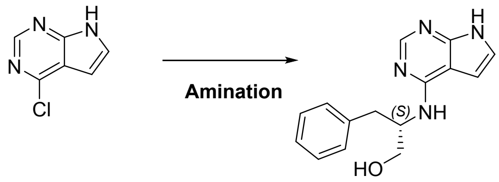

4-Chloro-7*H*-pyrrolo[2,3-*d*]pyrimidine (100 mg, 0.65 mmol) and (*S*)-2-amino-3-phenylpropan-1-ol (162 mg, 1.07 mmol) were dissolved in *n*-BuOH (0.5 mL), stirred at reflux for 9 h. under an atmosphere of nitrogen, before the mixture was cooled and the solvent removed under reduced pressure. The residue was purified by silica-gel column chromatography (CH_2Cl_2/MeOH - 9:1, R_f = 0.24). The crude product was further purified by recrystallization from EtOH giving 88 mg (0.33 mmol, 51%) of an off-white solid; mp. 214 - 218 °C (dec.); $[\alpha]_D^{20}$= -140.4 (*c* 1.00, CH_2Cl_2/MeOH); ^{1}H NMR (400 MHz, DMSO-d_6) δ 11.43 (s, 1H), 8.04 (s, 1H), 7.30 - 7.27 (m, 2H), 7.25 - 7.21 (m, 2H), 7.15 - 7.10 (m, 2H), 7.04 - 7.02 (m, 1H), 6.59 - 6.58 (m, 1H), 4.87 (t, *J* = 5.5 Hz, 1H), 4.50 - 4.41 (m, 1H), 3.57 - 3.52 (m, 1H), 3.47 - 3.41 (m, 1H), 3.03 - 2.98 (m, 1H), 2.88 - 2.82 (m, 1H); ^{13}C NMR (100 MHz, DMSO-d_6)

δ 155.9, 151.3, 150.1, 139.8, 129.1, 128.1, 125.8, 120.6, 102.6, 98.7, 62.8, 53.4, 36.7; HRMS (ASAP+, m/z): found 269.1403, calcd for $C_{15}H_{17}N_4O$, $[M+H]^+$, 269.1397.

Tert-butyl 3-(benzyl(7*H*-pyrrolo[2,3-*d*]pyrimidin-4-yl)amino)propanoate (S-16)

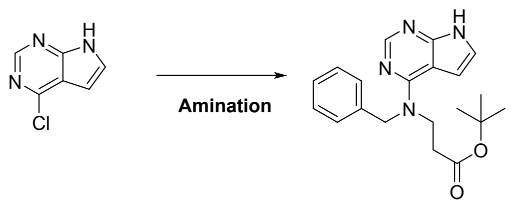

4-Chloro-7*H*-pyrrolo[2,3-*d*]pyrimidine (206 mg, 1.34 mmol) and *tert*-butyl 3-(benzylamino)propanoate (948 mg, 4.03 mmol) were dissolved in *n*-BuOH (1 mL), stirred at reflux for 16 h. under an atmosphere of nitrogen, before the mixture was cooled and the solvent removed under reduced pressure. The residue was partitioned between CH_2Cl_2 (10 mL) and water (10 mL). The layers were separated, and the aqueous phase extracted using more CH_2Cl_2 (2 × 10 mL). The combined organic phases were dried (Na_2SO_4), filtered and evaporated under reduced pressure. Purification by silica-gel column chromatography (CH_2Cl_2/MeOH - 9/1, R_f = 0.49) gave 328 mg (0.93 mmol, 70%) of a white solid; mp. 142 - 144 °C; ^{1}H NMR (400 MHz, $CDCl_3$) δ 10.78 (s, 1H), 8.36 (s, 1H), 7.34 - 7.24 (m, 5H), 7.01 (m, 1H), 6.40 (m, 1H), 5.08 (s, 2H), 4.00 (t, *J* = 7.6 Hz, 2H), 2.72 (t, *J* = 7.6 Hz, 2H), 1.44 (s, 9H); ^{13}C NMR (100 MHz, $CDCl_3$) δ 171.3, 157.1, 152.2, 151.4, 138.1, 128.9, 127.3, 127.1, 120.5, 102.2, 102.0, 81.0, 52.4, 45.3, 34.5, 28.2; HRMS (ASAP+, m/z): found 353.1974, calcd for $C_{20}H_{25}N_4O_2$, $[M+H]^+$, 353.1978

(*R*)-*N*-Methyl-6-phenyl-*N*-(1-phenylethyl)-7*H*-pyrrolo[2,3-*d*]pyrimidin-4-amine (S-19)

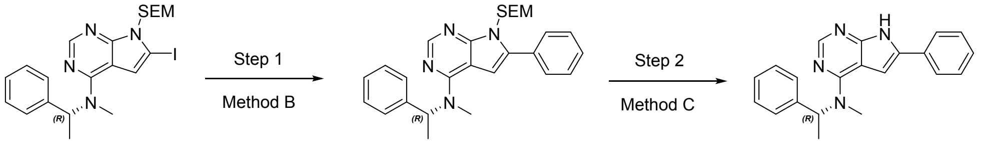

Step 1: (*R*)-*N*-Methyl-6-phenyl-*N*-(1-phenylethyl)-7-((2-(trimethylsilyl)ethoxy)methyl)-7H-pyrrolo[2,3-*d*]pyrimidin-4-amine

Compound **58** (244 mg, 0.48 mmol, 1 equiv.) was treated with phenylboronic acid as described in General Procedure B. Purification by silica-gel column chromatography (*n*-pentane/diethyl ether/MeOH, 12:4:0.5, R_f= 0.45) gave 186 mg (0.40 mmol, 84%) of a dark orange oil; $[\alpha]_D^{20}$ =

+59.1 (c 1.00, $CHCl_3$); ^1H NMR (400 MHz, DMSO-d_6) δ 8.26 (s, 1H), 7.75 - 7.73 (m, 2H), 7.50 - 7.46 (m, 2H), 7.43 - 7.39 (m, 1H), 7.36 - 7.34 (m, 2H), 7.34 - 7.32 (m, 2H), 7.29 - 7.25 (m, 1H), 6.87 (s, 1H), 6.47 (br.s, 1H), 5.56 (s, 2H), 3.64 - 3.60 (m, 2H), 3.07 (s, 2H), 1.61 - 1.59 (m, 3H), 0.87 - 0.83 (m, 2H), -0.08 (s, 9H); ^{13}C NMR (100 MHz, DMSO-d_6) δ 156.6, 153.2, 151.2, 141.4, 136.3, 131.5, 128.7 (2C), 128.6 (2C), 128.4 (2C), 128.1, 127.0, 126.8 (2C), 102.6, 102.4, 70.3, 65.7, 52.3, 31.7, 17.3, 16.2, -1.4 (3C); IR (neat, cm^{-1}): 3038 (w), 2945 (w), 2893 (w), 1566 (s), 1494 (m), 1456 (w), 1413 (m), 1307 (m), 1247 (w), 1072 (m), 1031 (w), 858 (m), 834 (m), 777 (w), 751 (m), 698 (m); HRMS (ASAP+, m/z): found 459.2579, calcd for $C_{27}H_{35}N_4OSi$, $[M+H]^+$, 459.2580.

Step 2: (*R*)-*N*-Methyl-6-phenyl-*N*-(1-phenylethyl)-7*H*-pyrrolo-[2,3-*d*]pyrimidin-4-amine

The SEM protected intermediate above (171 mg, 0.374 mmol) was treated as described in General Procedure C. The crude product was purified by silica-gel column chromatography ($MeOH/CH_2Cl_2$ - 1:9, R_f = 0.48). Drying gave 43 mg (0.13 mmol, 36%) of a pale yellow solid, mp. 177 - 180 °C, $[\alpha]_D^{20} =$ +86.7 (c 0.50, $CHCl_3$); ^1H NMR (400 MHz, DMSO-d_6) δ 12.20 (bs, 1H), 8.17 (s, 1H), 7.90 - 7.85 (m, 2H), 7.44 - 7.38 (m, 2H), 7.37 - 7.32 (m, 4H), 7.30 - 7.24 (m, 2H), 7.14 - 7.09 (m, 1H), 6.50 - 6.42 (m, 1H), 3.08 (s, 3H), 1.59 - 1.61 (m, 3H); ^{13}C NMR (100 MHz, DMSO-d_6) δ 156.6, 153.1, 151.1, 141.6, 133.1, 131.6, 128.8 (2C), 128.5 (2C), 127.3 (2C), 126.94, 126.89, 124.7 (2C), 103.5, 99.1, 52.1, 31.7, 16.2; IR (neat, cm^{-1}): 3215 (w), 3085 (w), 2961 (w), 2851 (w), 2737 (w), 2358 (w), 2332 (w), 1594 (m), 1560 (s), 1497 (m), 1411 (m), 1320 (m), 1320 (m), 1070 (w), 924 (w), 750 (m), 697 (m); HRMS (ASAP+, m/z): found 329.1763, calcd for $C_{21}H_{21}N_4$ $[M+H]^+$ 329.1766.

(4-(4-Amino-7*H*-pyrrolo[2,3-*d*]pyrimidin-6-yl)phenyl)methanol (S-26)

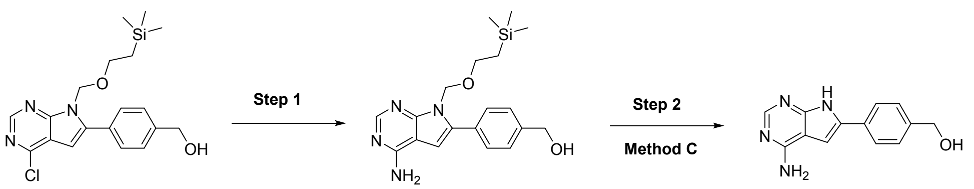

Step 1: (4-(4-Amino-7-((2-(trimethylsilyl)ethoxy)methyl)-7*H*-pyrrolo[2,3-*d*]pyrimidin-6-yl)phenyl)methanol

Compound **119** (103 mg, 0.265 mmol), aqueous ammonia (2 mL, 25%) and 1,4-dioxane (2 mL) were sealed in a thick-walled glass reaction vial. The reaction mixture was stirred

vigorously under microwave irradiation (140 ˚C, 16 bar, 110 W) for 45 min. The volatiles were removed under reduced pressure and the residue purified by silica-gel column chromatography (CH_2Cl_2/MeOH - 92.5:7.5, R_f = 0.20) which gave 80 mg (0.215 mmol, 81%) of a colourless solid; ^{1}H NMR (400 MHz, $CDCl_3$) δ 8.37 (s, 1H), 7.77 – 7.70 (m, 2H), 7.51 – 7.44 (m, 2H), 6.45 (s, 1H), 5.56 (s, 2H), 5.12 (s, 2H), 4.78 (s, 2H), 3.77 – 3.69 (m, 2H), 2.01 (s, 1H), 1.00 – 0.92 (m, 2H), -0.03 (s, 9H); ^{13}C NMR (101 MHz, $CDCl_3$) δ 156.2, 152.9, 152.2, 141.2, 139.0, 130.9, 129.4 (2C), 127.3 (2C), 103.1, 97.9, 70.7, 66.6, 65.0, 18.0, -1.4 (3C); HRMS (ES+, m/z): found 371.1905, calcd for $C_{19}H_{27}N_4O_2Si$, $[M+H]^+$, 371.1903.

Step 2: (4-(4-Amino-7*H*-pyrrolo[2,3-*d*]pyrimidin-6-yl)phenyl)methanol (S-26)

The material above (12.3 mg, 0.033 mmol) was dissolved in CH_2Cl_2 (1.5 mL) and *i*-PrOH (0.1 mL). Trifluoroacetic acid (0.32 mL) was added and the mixture was heated on an oil-bath (50 ˚C) under an atmosphere of nitrogen for 2 h. The volatiles were removed under reduced pressure and the residue was added tetrahydrofuran (2.0 mL) and methanolic ammonia (2.5 mL, 7 M) and stirred at rt for 10 min. The volatiles were removed under reduced pressure and the residue was purified by silica-gel column chromatography (CH_2Cl_2/7 M NH_3 (in MeOH) - 85:15, R_f = 0.36) giving 7.1 mg (0.029 mmol, 90%) of an off-white powder; ^{1}H NMR (600 MHz, DMSO-d_6) δ 11.96 (s, 1H), 8.03 (s, 1H), 7.75 – 7.70 (m, 2H), 7.39 – 7.35 (m, 2H), 6.96 (s, 2H), 6.88 (s, 1H), 5.20 (t, *J* = 5.7 Hz, 1H), 4.51 (d, *J* = 5.7 Hz, 2H); ^{13}C NMR (151 MHz, DMSO-d_6) δ 157.1, 151.9, 151.8, 141.7, 133.5, 130.2, 127.0 (2C), 124.3 (2C), 103.5, 95.7, 73.8, 62.6; IR (neat, cm^{-1}): 3460 (w), 3309 (w), 3182 (w), 3111 (w), 3005 (w), 2921 (w), 2888 (w), 2847 (w), 2735 (w), 1640 (s), 1591 (s), 1545 (m), 1490 (m), 1322 (m), 1029 (m), 1012 (m), 840 (m), 802 (m), 781 (s), 759 (m), 630 (m), 460 (s), 448 (s); HRMS (ES+, m/z): found 241.1091, calcd for $C_{13}H_{13}N_4O$, $[M+H]^+$, 241.1089.

(4-(4-(Methylamino)-7*H*-pyrrolo[2,3-*d*]pyrimidin-6-yl)phenyl)methanol (S-27)

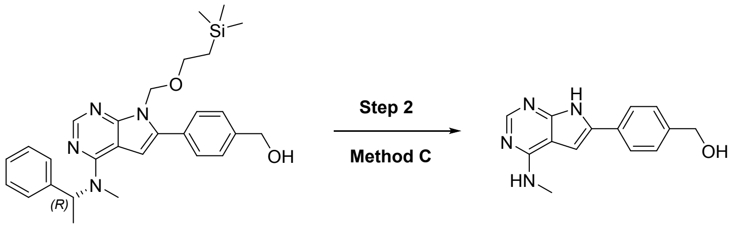

Compound **89** (99 mg, 0.203 mmol) was treated as described in General procedure C using $NaHCO_3$ in the second step. This gave a by-product which was isolated by silica-gel column

chromatography (CH_2Cl_2/7 M NH_3 (in MeOH) - 92.5:7.5, R_f = 0.20). Drying gave 12 mg (0.047 mmol, 23%) of an off-white solid, mp > 267 °C (decomp.). ^{1}H NMR (400 MHz, DMSO-d_6) δ 11.98 (s, 1H), 8.13 (s, 1H), 7.77 – 7.70 (m, 2H), 7.43 (q, *J* = 5.0 Hz, 1H), 7.40 – 7.31 (m, 2H), 6.88 (s, 1H), 5.21 (t, *J* = 5.7 Hz, 1H), 4.52 (d, *J* = 5.8 Hz, 2H), 2.98 (d, *J* = 4.6 Hz, 3H); ^{13}C NMR (151 MHz, DMSO-d_6) δ 156.3, 151.9, 151.2, 141.6, 133.3, 130.2, 127.0 (2C), 124.3 (2C), 103.9, 95.3, 62.6, 27.2; IR (neat, cm^{-1}): 3452 (w), 3340 (w), 3201 (w), 3112 (w), 3071 (w), 3002 (w), 2927 (w), 2867 (w), 2732 (w), 1595 (s), 1544 (m), 1522 (m), 1485 (m), 1439 (m), 1396 (m), 1356 (m), 1310 (m), 1005 (m), 902 (m), 823 (m), 806 (m), 756 (s), 635 (m); HRMS (ES+, m/z): found 255.1251, calcd for $C_{14}H_{15}N_4O$ $[M+H]^+$ 255.1246.

(*S*)-2-((7-(4-Methoxybenzyl)-5-phenyl-7*H*-pyrrolo[2,3-*d*]pyrimidin-4-yl)amino)-2-phenylethan-1-ol (S-50)

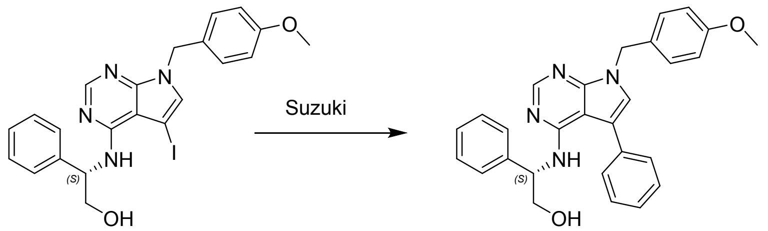

(*S*)-2-((5-Iodo-7-(4-methoxybenzyl)-7*H*-pyrrolo[2,3-*d*]pyrimidin-4-yl)amino)-2-phenylethan-1-ol[8] (347 mg, 0.694 mmol), phenylboronic acid (89 mg, 0.730 mmol). XPhos (20 mg, 42.0 μmol, 10 mol%), XPhos 2*nd* Gen. (34 mg, 43.2 μmol, 28 mol%), K_2CO_3 (401 mg, 2.90 mmol) and water/1,4-dioxane (1/1, 8 mL) were mixed under an N_2 atmosphere. The reaction mixture was then stirred at 100 °C for 18 h, before the solvent was removed and the product was diluted with water (20 mL). The water phase was extracted with EtOAc (3 × 30 mL) and the combined organic phases were washed with brine (30 mL), dried over Na_2SO_4, filtered and concentrated *in vacuo*. Purification was by silica-gel column chromatography (EtOAc, R_f = 0.63) to give 194 mg (0.431 mmol, 62%) of a colourless foam after drying, mp. 74 - 75 °C; HPLC purity: 99% (Method A); $[\alpha]_D^{20}$= -12.0 (*c* = 1.00, $CHCl_3$); ^{1}H NMR (400 MHz, DMSO-d_6) *δ* 8.18 (s, 1H), 7.55 - 7.53 (m, 2H), 7.50 - 7.47 (m, 2H), 7.44 (s, 1H), 7.39 - 7.36 (m, 1H), 7.31 - 7.25 (m, 6H), 7.22 - 7.18 (m, 1H), 6.87 (d, *J* = 8.7 Hz, 2H), 5.99 (d, *J* = 7.4 Hz, 1H), 5.34 - 5.31 (m, 3H), 4.95 (t, *J* = 5.0 Hz, 1H), 3.74 - 3.54 (m, 5H); ^{13}C NMR (100 MHz, DMSO-d_6) *δ* 158.7, 155.6, 151.5, 149.6, 141.4, 134.6, 129.9, 129.14 (2C), 129.10 (2C), 128.4 (2C), 128.1 (2C), 126.9, 126.71, 126.65 (2C), 123.2, 115.3, 113.9 (2C) , 100.2, 64.6, 55.5, 55.1, 46.7; IR (neat,

cm^{-1}): 3410, 3059, 2834, 1585, 1466, 1174, 1067, 756, 698; HRMS (APCI/ASAP, *m/z*): found 451.2135, calcd for $C_{28}H_{27}N_4O_2$, $[M+H]^+$, 451.2134.

4-(Pyridin-4-ylmethoxy)thieno[2,3-d]pyrimidine (S-52)

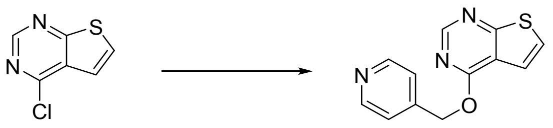

4-Chlorothieno[2,3-*d*]pyrimidine (256 mg, 1.50 mmol), pyridin-4-ylmethanol (164 mg, 1.50 mmol, 1 equiv.) and Cs_2CO_3 (489 mg, 1.50 mmol, 1 equiv.) were stirred in acetonitrile (2 mL) under a N_2 atmosphere at 80 ^oC for 6 h. The reaction mixture was cooled to rt and diluted with EtOAc (40 mL), washed with saturated aq. solution of $NaHCO_3$ (2 × 30 mL), water (4 × 20 mL) and brine (2 × 20 mL). The organic phase was dried over anhydrous Na_2SO_4 and concentrated in vacuo. The crude product was adsorbed on Celite and purified by silica-gel column chromatography (EtOAc, R_f = 0:23). This yielded 244 mg (1.01 mmol, 67%) of a brown solid; mp. 82 - 83 ^oC; HPLC purity > 99%; ^1H NMR (400 MHz, DMSO-*d*$_6$) δ 8.69 (s, 1H), 8.58 (d, *J* = 6.0 Hz, 2H), 7.88 (d, *J* = 5.9 Hz, 1H) 7.58 (d, *J* = 5.9 Hz, 1H), 7.49 (d, *J* = 5.9 Hz, 2H), 5.68 (s, 2H); ^{13}C (100 MHz, DMSO-*d*$_6$) δ 168.3, 162.7, 153.0, 149.8 (2C), 145.3, 126.8, 121.8, 118.5 (2C), 66.0; IR (neat, cm^{-1}): 3050, 1575, 1533, 1347, 1034, 789, 699, 556; HRMS (ASAP+, m/z): found 244.0544, calcd for $C_{12}H_{10}N_30S$, $[M+H]^+$, 244.0548.

4-(Pyridin-3-ylmethoxy)thieno[2,3-d]pyrimidine (S-53)

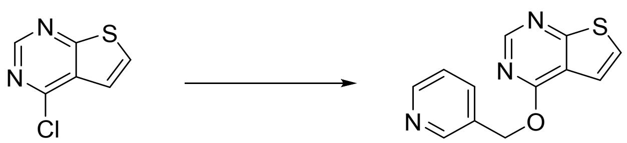

The material was prepared as described in the section above, but using pyridin-3-ylmethanol. Purification by silica-gel column chromatography (EtOAc/*n*-pentane - 4:1, R_f = 0.26) yielded 253 mg (1.04 mmol, 72%) of a green solid, mp. 77 - 79 ^oC; HPLC purity > 99%; ^1H NMR (400 MHz, DMSO-*d*$_6$) δ 8.76 (s, 1H), 8.72 (s, 1H), 8.57 (d, *J* = 4.8 Hz, 1H), 7.96 (d, *J* = 7.8 Hz, 1H), 7.85 (d, *J* = 5.9 Hz, 1H), 7.44 (dd, *J* = 7.8 and 4.8 Hz, 1H), 5.66 (s, 2H); ^{13}C (100 MHz, DMSO-*d*$_6$) δ 168.1, 162.9, 153.0, 149.5, 149.4, 136.1, 131.8, 126.7, 123.6, 118.4, 65.6; IR (neat, cm^{-1}): 3037, 1566, 1526, 1340, 1046, 739, 557; HRMS (ASAP+, m/z): found 244.0541, calcd for $C_{12}H_{10}N_3OS$, $[M+H]^+$, 244.0545.

4-(Pyridin-2-ylmethoxy)thieno[2,3-*d*]pyrimidine (S-54)

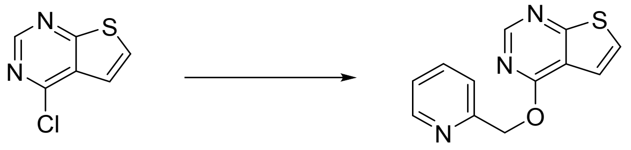

The material was prepared as described above, but using pyridin-2-ylmethanol (170 µL, 1.76 mmol, 1.2 equiv.). Purification by silica-gel column chromatography (EtOAc/*n*-pentane 4 : 1, R_f = 0.40) yielded 311 mg (1.28 mmol, 86%) of an off-white solid, mp. 65-66 °C; HPLC purity > 99%; ^{1}H NMR (400 MHz, DMSO-d_6) δ 8.68 (s, 1H), 8.57 (m, 1H), 7.87 (d, J = 6.0 Hz, 1H), 7.82 (t, J = 3.4 Hz, 1H), 7.55 (m, 1H), 7.35 (dd, J = 5.0 and 4.1 Hz, 1H), 5.69 (s, 2H); ^{13}C (100 MHz, DMSO-d_6) δ 168.2, 162.9, 155.7, 153.0, 149.2, 137.0, 126.7, 123.0,121.6, 118.5, 118.4, 68.3; IR (neat, cm^{-1}): 3064, 1528, 1429, 1340, 1031, 713; HRMS (ASAP+, m/z): found 244.0543, calcd for $C_{12}H_{10}N_3OS$, $[M+H]^+$, 244.0545.

(*rac*)-*N*-(2,2,2-Trifuoro-1-phenylethyl)thieno[2,3-*d*]pyrimidin-4-amine (S-55)

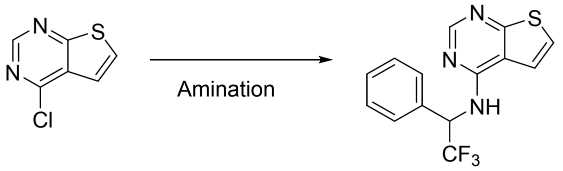

4-Chlorothieno[2,3-*d*]pyrimidine (200 mg, 1.17 mmol) and (*rac*)-2,2,2-trifluoro-1-phenylethan-1-amine (616 mg, 3.52 mmol, 3 eq.) were dissolved in *n*-BuOH (3 mL), under an N_2 atmosphere, and stirred at reflux for 26 h, before the mixture was cooled and the solvent removed under reduced pressure. EtOAc (25 mL) and water (25 mL) were added to the residue, the phases were separated, and the aqueous phase extracted with more EtOAc (2 × 10 mL). The combined organic fractions were washed with brine (20 mL), dried over anhydrous Na_2SO_4 and the solvent removed in vacuo. Purification by silica-gel column chromatography (*n*-pentane/EtOAc - 1:1, R_f = 0.21), yielded 202 mg (0.65 mmol, 56%) of a purple solid, mp. 163-164 °C; HPLC purity > 99%, t_R =22.9 min; ^{1}H NMR (400 MHz, DMSO-d_6) δ 8.78 (d, J = 8.8 Hz, 1H), 8.46 (s, 1H), 8.00 - 7.98 (m, 1H), 7.72 - 7.67 (m, 3H), 7.48 - 7.38 (m, 3H), 6.65 - 6.50 (m, 1H); ^{13}C NMR (100 MHz, DMSO-d_6) δ 166.7, 155.9, 153.0,133.3, 129.0, 128.6, 128.5 (2C), 125.0 (q, J = 282.3 Hz), 123.6, 119.7, 116.4, 53.9 (q, J = 30.5 Hz); ^{19}F NMR (564 MHz, DMSO-d_6, decoupled): -74.0; IR (neat, cm^{-1}): 3228 , 3154, 3118, 3071, 3004, 2944, 1590,

1545, 1499, 1358, 1309, 1263, 1171, 1114, 1084, 965, 884, 801, 693, 603; HRMS (ASAP+, m/z): found 310.0628, calcd for $C_{14}H_{11}N_3F_3S$, $[M+H]^+$, 310.0628.

_N_-Benzyl-6-phenylthieno[2,3-_d_]pyrimidin-4-amine (S-57)

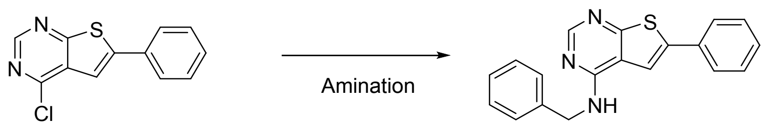

4-Chloro-6-phenylthieno[2,3-*d*]pyrimidine [9] (100 mg, 0.41 mmol) and benzylamine (0.135 mL, 133 mg, 1.24 mmol) were diluted with *n*-BuOH and heated at 140 °C. for 25 h, before the mixture was cooled and the solvent removed under reduced pressure. EtOAc (25 mL) and water (25 mL) were added to the residue, the phases were separated, and the aqueous phase extracted with more EtOAc (2 × 10 mL). The combined organic fractions were washed with brine (20 mL), dried over anhydrous Na_2SO_4 and the solvent removed in vacuo. Purification was by crystallization form EtOAc at -18 °C. The solid obtained by filtration was washed with cold diethyl ether (3 × 20 mL) and dried to give 60 mg (0.19 mmol, 47%) of slight beige needle crystals; mp. 200 - 201 °C; HPLC purity > 99%; ^{1}H NMR (400 MHz, DMSO-d_6) δ 8.35 (s, 1H), 8.51 (t, J = 5.7 Hz, 1H), 8.08 (s, 1H), 7.70 – 7.65 (m, 2H), 7.53 – 7.47 (m, 2H), 7.43 – 7.31 (m, 5H), 7.29 – 7.23 (m, 1H), 4.77 (d, J = 5.8 Hz, 2H); ^{13}C NMR (100 MHz, DMSO-d_6) δ 165.1, 156.6, 154.0, 139.3, 138.2, 133.2, 129.4, 128.6, 128.4 (2C), 127.4 (2C), 126.9, 125.6 (2C), 117.5, 115.3, 43.4; IR (neat, cm^{-1}): 3018, 690; HRMS (ESI, 70eV, m/z): found 318.1053,calcd for $C_{19}H_{16}N_3S$, $[M+H]^+$ 318,1059.

Methyl (_R_)-3-(4-((1-phenylethyl)amino)thieno[2,3-_d_]pyrimidin-6-yl)benzoate (S-62)

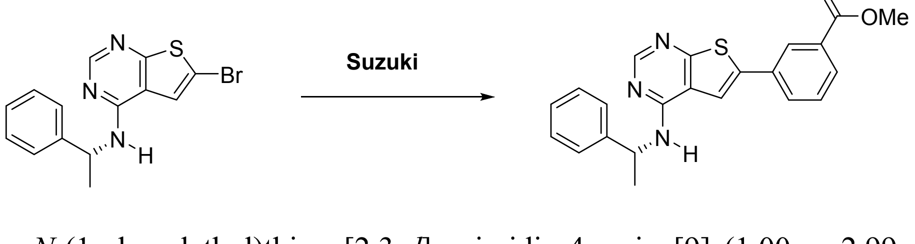

(*R*)-6-Bromo-*N*-(1-phenylethyl)thieno[2,3-*d*]pyrimidin-4-amine[9] (1.00 g, 2.99 mmol) was mixed with (3-(methoxycarbonyl)phenyl)boronic acid (807 mg, 4.49 mmol, 1.5 equiv.), fine powdered K_2CO_3 (1.24 g, 8.97 mmol, 3 equiv.), PEPPSI-SIPR (102 mg, 0.1495 mmol, 0.05 equiv.). The air was exchanged with nitrogen gas and degassed 1,4-dioxane/water (1/1 by vol.

%, 30 mL) was added. The reaction was then stirred at 80 °C for 1 h. under a N_2 atmosphere. The reaction mixture was concentrated, diluted with EtOAc (40 mL) and water (30 mL). After phase separation, the water phased was extracted with more EtOAc (2 × 40 mL). Following drying over Na_2SO_4 and concentration, the crude material was absorbed onto Celite. The material was purified by silica-gel column chromatography (CH_2Cl_2/EtOAc - 5:1, R_f = 0.32) to yield 946 mg (2.42 mmol, 81%) of a white solid; mp. 79 - 82 °C; ^{1}H NMR (400 MHz, DMSO-d_6) δ 8.35 (d, J = 7.9 Hz, 1H), 8.33 (s, 1H), 8.30 (s, 1H), 8.29 - 8.27 (m, 1H), 7.98 - 7.93 (m, 1H), 7.93 - 7.87 (m. 1H), 7.64 (t, J = 7.8, 1H), 7.47 - 7.41 (m, 2H), 7.36 - 7.29 (m, 2H), 7.25 -7.19 (m, 1H), 5.56 - 5.47 (m, 1H), 3.91 (s, 3H), 1.58 (d, J = 7.1 Hz, 3H); ^{13}C NMR (100 MHz, DMSO-d_6,) δ 165.8, 165.3, 155.9, 154.2, 144.6, 136.6, 133.7, 130.6, 130.3, 130.1, 128.9, 128.3 (2C), 126.7, 126.1 (2C), 125.4, 117.4, 116.6, 52.4, 49.1, 22.5; IR (neat, cm^{-1}): 2977, 2955, 1721, 1574, 1513, 1286, 1195, 750, 697. HRMS (ES+, m/z): found 390.1279, calcd for $C_{22}H_{20}N_3O_2S$, $(M+H)^+$, 390.1276.

Synthesis of (4-(4-(benzyl(methyl)amino)thieno[2,3-*d*]pyrimidin-6-yl)phenyl)methanol (S-64)

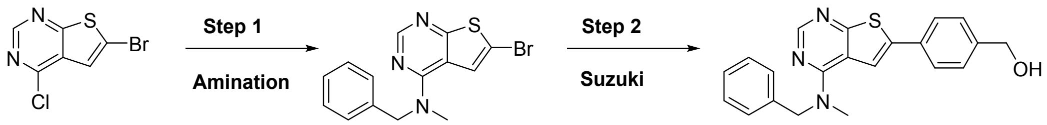

Step 1: *N*-benzyl-6-bromo-*N*-methylthieno[2,3-*d*] pyrimidine-4-amine

To 6-bromo-4-chlorothieno[2,3-*d*]pyrimidine (1.06 g, 4.22 mmol) was added *N*-benzylmethylamine (0.97 g, 8 mmol, 1.9 equiv.) and *i*-PrOH (12 mL). The mixture was stirred under an N_2-atmosphere for 24 h at 80 °C until full conversion. After removal of the solvent, the product was diluted with diethyl ether (150 mL) and washed with water (3 × 50 mL). The combined organic phase was then washed with a saturated NaCl solution (20 mL), dried over anhydrous Na_2SO_4, filtered, and concentrated *in vacuo.* Purification using silica-gel column chromatography (*n*-pentane/EtOAc – 5:1, R_f = 0.30) gave 1.16 g (3.48 mmol, 82%) of a pale yellow solid; mp. 129 - 130 °C, ^{1}H NMR (400 MHz, DMSO-d_6) δ 8.35 (s, 1H), 7.71 (s, 1H), 7.38 - 7.31 (m, 2H), 7.30 - 7.24 (m, 3H), 5.03 (s, 2H), 3.35 (s, 3H); ^{13}C NMR (150 MHz, DMSO-d_6/$CDCl_3$) δ 169.4, 156.4, 152.5, 136.9, 128.3 (2C), 126.9, 126.7 (2C), 124.5, 115.6, 108.6, 53.6, 38.2; IR (neat, cm^{-1}): 1546, 1500, 1403, 1019, 969, 731, 697, 465; HRMS (TOF, ASAP+, m/z): found 334.0019, calcd for $C_{14}H_{13}N_3S^{79}Br$, $(M+H)^+$, 334.0014.

Step 2: (4-(4-(Benzyl(methyl)amino)thieno[2,3-*d*]pyrimidin-6-yl)phenyl)methanol

N-Benzyl-6-bromo-*N*-methylthieno[2,3-*d*]pyrimidine-4-amine (281 mg, 0.840 mmol) were mixed with (4-(hydroxymetyl)phenyl)boronic acid (190 mg, 1.25 mmol, 1.5 equiv.), powdered K_2CO_3 (430 mg, 3.83 mmol), $Pd(PPh_3)_4$ (13 mg, 11.6 μmol, 0.01) and degassed 1,4-dioxane/water (1:1 by vol.%, 6 mL). The reaction was stirred at 80 °C for 3.5 h under a N_2 atmosphere. The solvent was removed and the product was diluted with water (25 mL) and EtOAc (50 mL). The phases were separated, and the water phase was extracted with more EtOAc (2 × 25 mL). The combined organic phases were washed with brine (15 mL), dried over Na_2SO_4, filtered and concentrated in vacuo. The crude product was purified by silica-gel column chromatography (*n*-pentane/EtOAc - 2:1, R_f= 0.35) followed by re-crystallisation from acetonitrile (3 mL). This gave 185 mg (0.511 mmol, 61%) of an off-white solid, mp. 137 – 138 °C (decomp); ^{1}H NMR (400 MHz, DMSO-d_6) δ 8.36 (s, 1H), 7.86 (s, 1H), 7.72 – 7.65 (m, 2H), 7.42 – 7.23 (m, 7H), 5.25 (t, *J* = 5.7 Hz, 1H), 5.10 (s, 2H), 4.52 (d, *J* = 5.7 Hz, 2H), 3.47 (s, 3H); ^{13}C NMR (101 MHz, DMSO-d_6) δ 167.9, 157.4, 152.7, 143.0, 137.7, 136.4, 131.5, 128.6 (2C), 127.1 (3C), 126.9 (2C), 125.6 (2C), 117.6, 116.4, 62.5, 53.7, 39.8; HRMS (TOF/ASAP, m/z): found 362.2332, calcd for $C_{21}H_{20}N_3OS$, $[M+H]^+$, 362.11327.

Synthesis of (4-(4-(Benzyl(ethyl)amino)thieno[2,3-*d*]pyrimidin-6-yl)phenyl)methanol (S-65)

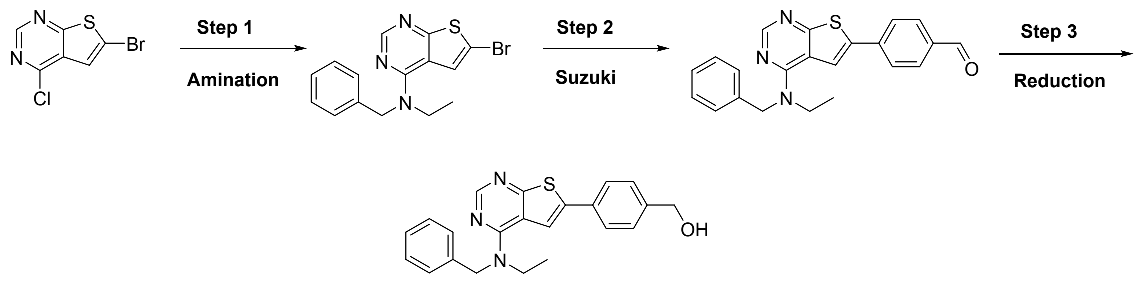

Step 1: *N*-Benzyl-6-bromo-*N*-ethylthieno[2,3-d]pyrimidin-4-amine

6-Bromo-4-chlorothieno[2,3-*d*]pyrimidine (2.05 g, 8.20 mmol) and *N*-ethylbenzylamine (2.4 mL, 16 mmol) were mixed with *i*-PrOH (4 mL), and stirred at 80 °C under a N_2 atmosphere until full conversion. The mixture was then cooled to 22 °C and diluted with water (40 mL) and diethyl ether (40 mL). After phase separation, the water phase was extracted with more diethyl ether (2 × 40 mL). The combined organic phases were washed with brine (20 mL), filtered and concentrated in vacuo. The crude product was purified by silica-gel column

chromatography (*n*-pentane/EtOAc - 6:1, R_f = 0.29). This gave 2.16 g (6.20 mmol, 76%) of a white solid, mp. 114 - 115 °C; HPLC purity > 99%; ^{1}H NMR (400 MHz, DMSO-*d*$_6$) δ 8.37 (s, 1H), 7.42 (s, 1H), 7.38-7.34 (m, 2H), 7.29 - 7.27 (m, 3H), 5.01 (s, 2H), 3.75 (q, *J* = 6.9 Hz, 2H), 1.23 (t, *J* = 7.0 Hz, 3H); ^{13}C NMR (100 MHz, DMSO-*d*$_6$) δ 169.9, 156.3, 153.5, 138.1, 129.2 (2C), 127.6, 127.1, (2C), 125.3, 115.5, 109.4, 52.0, 44.8, 13.2, IR (neat, cm^{-1}): 3059, 1541, 1485, 1358, 1296, 1025, 970, 708, 693, 675; HRMS(ESI/APCI): found 348.0175, calcd. for $C_{15}H_{14}{}^{79}BrN^3S$, $[M+H]^+$, 348.0170.

Step 2: 4-(4-(benzyl(ethyl)amino)thieno[2,3-d]pyrimidin-6-yl)benzaldehyde

N-Benzyl-6-bromo-*N*-ethylthieno[2,3-*d*]pyrimidin-4-amine (503 mg, 1.35 mmol) were mixed with 4-formylphenyl boronic acid (263 mg, 1.75 mmol), powdered K_2CO_3 (560 mg, 4.05 mmol, 3 equiv.), $Pd(PPh_3)_4$ (16 mg, 0.0135 mmol, 0.01 equiv.) and degassed 1,4-dioxane/water (1:1 by vol.%, 12 mL). The reaction was stirred at 80 °C for 20 h. under a N_2 atmosphere. The solvent was removed and the residue was diluted with water (100 mL) and CH_2Cl_2 (100 mL). The phases were separated, and the water phase was extracted with more CH_2Cl_2 (2×25 mL). The combined organic phases were washed with brine (50 mL), dried over Na_2SO_4, filtered and concentrated in vacuo. The crude product was purified by silica-gel column chromatography (EtOAc/*n*-pentane - 1:3, R_f = 0.26). This gave 250 mg (0.71 mmol, 47%) of a yellow solid, mp. 135 – 137 °C (decomp); HPLC purity: 95%; ^{1}H NMR (400 MHz, DMSO-*d*$_6$) δ 10.0 (s, 1H), 8.42 (s, 1H), 7.98 - 7.96 (m, 2H), 7.89 - 7.87 (m, 2H), 7.83 (s, 1H), 7.39 - 7.33 (m, 4H), 7.28 - 7.26 (m, 1H), 5.14 (s, 2H), 3.89 (q, *J* = 7.0 Hz, 2H), 1.30 (t, *J* = 7.2 Hz, 3H); ^{13}C NMR (100 MHz, DMSO-*d*$_6$) δ 192.8, 169.3, 157.4, 153.9, 139.0, 138.4, 136.0, 135.3, 130.8 (2C), 129.2 (2C), 127.6, 127.2 (2C), 126.8 (2C), 120.9, 116.1, 52.2, 45.1, 13.3; IR (neat, cm^{-1}): 3058, 3028,2979, 2931, 2870, 2824, 2735, 1696, 1600, 1450, 1323, 1169, 817, 774; HRMS(ESI/APCI, m/z): found 374.1325, calcd. for $C_{22}H_{19}N_3OS$, $[M+H]^+$, 374.1327.

Step 3: (4-(4-(Benzyl(ethyl)amino)thieno[2,3-d]pyrimidin-6-yl)phenyl)methanol

4-(4-(Benzyl(ethyl)amino)thieno[2,3-d]pyrimidin-6-yl)benzaldehyde (140 mg, 0.40 mmol) was dissolved in THF/MeOH (3:1, 20 mL) and mixed with $NaBH_4$ (15 mg, 0.40 mmol, 1 equiv.). The reaction was stirred for 30 min. at rt before additional $NaBH_4$ (1 equiv.) was added. The mixture was stirred for another 30 min., and the mixture then concentrated to about 5 mL. The residue was diluted with EtOAc (50 mL) and water (50 mL), the phases were separated, and the water phase was extracted with more EtOAc (2 × 25 mL). The combined organic phases were washed with brine (25 mL), dried over Na_2SO_4, filtered and concentrated in vacuo. The

crude product was purified by silica-gel chromatography (EtOAc/*n*-pentane - 1:1, R_f =0.25). This gave 120 mg (0.33 mmol, 87%) of a white solid, mp. 160 - 161 °C; HPLC purity > 99%; ^{1}H NMR (400 MHz, DMSO-d_6) δ 8.43 (s, 1H), 7.65 (s, 1H), 7.63 (s, 2H), 7.44-7.37 (m, 6H), 7.34 - 7.30 (m, 1H), 5.32 (t, *J* = 5.8 Hz, 1H), 5.15 (s, 2H), 4.57 (d, *J* = 5.7 Hz, 2H), 3.92 (q, *J* = 7.0 Hz, 2H), 1.34 (t, *J* = 7.3 Hz, 3H); ^{13}C NMR (100 MHz, DMSO-d_6) δ 168.4, 157.2, 153.3, 143.6, 138.5, 137.1, 131.9, 129.2 (2C), 127.6 (2C), 127.5, 127.1 (2C), 126.1 (2C), 117.8, 116.2, 62.9, 52.2, 45.0, 13.4; IR (neat, cm^{-1}): 3284, 2978, 1548, 1450, 1300, 1041, 810, 773, 697; HRMS(ESI/APCI, m/z): found 376,1480, calcd for $C_{22}H_{21}N_3OS$, $[M+H]^+$, 376.1484.

_N_-Benzyl-_N_-methyl-6-phenylfuro[2,3-_d_]pyrimidin-4-amine (S-75)

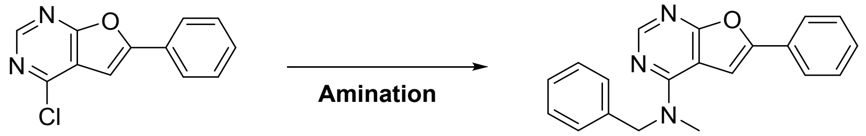

4-Chloro-6-phenylfuro[2,3-*d*]pyrimidine [12] (227 mg, 0.984 mmol) was mixed with *N*-methyl-1-phenylmethanamine (358 mg, 2.95 mmol, 3.0 equiv.) in *n*-BuOH (4 mL) under a N_2-atmosphere. The reaction was stirred for 22 h at 130 ° C. After cooling to 22 °C most of the *n*-BuOH was removed by evaporation. The residue was taken up in EtOAc (50 mL) and water (15 mL). The phases were separated, and the organic phase was washed with more water (15 mL) and brine (15 mL) and dried over Na_2SO_4. The concentrated material was absorbed onto Celite and purified by silica-gel column chromatography (*n*-pentane/EtOAc - 1:1 R_f= 0.52). This gave 242 mg (0.767 mmol, 78%) of a white solid, mp. 120 - 122 °C; ^{1}H NMR: (400 MHz, $CDCl_3$) δ 8.42 (s, 1H); 7.77 (d, *J* = 7.9 Hz, 2H), 7.44 - 7.27 (m, 8H), 6.91 (s, 1H), 5.04 (s, 2H), 3.37 (s, 3H); ^{13}C NMR (100 MHz, $CDCl_3$) δ 167.5, 158.2, 153.4, 151.2, 137.2, 129.5, 129.0 (2C), 128.9 (2C), 128.8, 127.7, 127.2 (2C), 124.6 (2C), 102.6, 100.3, 53.9, 37.2; IR (neat, cm^{-1}): 3031, 3218, 1582, 1560, 1504, 1265, 1066, 785, 734; HRMS (ES, m/z): found 316.1451, calcd for $C_{20}H_{18}N_3O$, $(M+H)^+$, 316.1450.

_N_-Benzyl-6-(4-methoxyphenyl)-_N_-methylfuro[2,3-_d_]pyrimidin-4-amine (S-76)

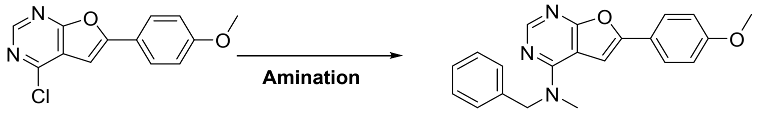

4-Chloro-6-(4-methoxyphenyl)furo[2,3-*d*]pyrimidine[12] (100 mg, 0.384 mmol) was mixed with *N*-methyl-1-phenylmethanamine (155 mg, 1.28 mmol, 3.3 equiv.) in *n*-BuOH (4 mL)

under a N_2-atmosphere. The reaction was stirred for 22 h at 130 ° C. After cooling to 22 °C most of the *n*-BuOH was removed by evaporation. The residue was taken up in EtOAc (50 mL) and water (15 mL). The phases were separated, and the organic phase was washed with more water (15 mL) and brine (15 mL) and dried over Na_2SO_4. The concentrated material was absorbed onto Celite and purified using neutral Al_2O_3 column chromatography (CH_2Cl_2, R_f= 0.33). This gave 120 mg (0.347 mmol, 90%) of an off-white solid, mp. 106 - 108 °C; ^{1}H NMR (400 MHz, $CDCl_3$) δ 8.41 (s, 1H); 7.71 (d, *J* = 8.8 Hz, 2H), 7.40 - 7.28 (m, 5H), 6.96 (d, *J* = 8.8 Hz, 2H), 6.97 (s, 1H), 5.04 (s, 2H), 3.86 (s, 3H), 3.37 (s, 3H); ^{13}C NMR: (100 MHz, $CDCl_3$) δ 167.3, 160.2, 158.0, 153.0, 151.4, 137.4, 128.9 (2C), 127.7 (2C), 127.2, 126.2 (2C), 122.3, 114.4 (2C), 102.8, 98.5, 55.5, 53.9, 37.2; IR (neat, cm^{-1}): 3031, 2836, 1583, 1502, 1251, 1066, 1045, 833, 776; HRMS (ES, m/z): found 346.1562, calcd for $C_{21}H_{20}N_3O_2$, $(M+H)^+$, 346.1556.

(*R*)-*N*-methyl-*N*-(1-phenylethyl)quinazolin-4-amine (S-77)

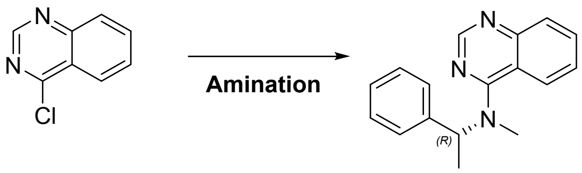

4-Chloroquinazoline (297 mg, 1.81 mmol) and (734 mg, 5.43 mmol) was added *n*-BuOH (5 mL) and agitated at 145 °C for 2 h. The solution was cooled to 22 °C and water (50 mL) was added. The reaction mixture was extracted with EtOAc (3 × 50 mL), dried over Na_2SO_4 and concentrated *in vacuo*. Purification by silica-gel column chromatography (EtOAc/*n*-pentane - 2:1, R_f= 0.32) gave 420 g (1.59 mmol, 88%) of a yellow oil; HPLC purity: 99%; $[\alpha]_D^{20}$ = +153.3 (*c* 1.00, DMSO); ^{1}H NMR (400 MHz, DMSO-d_6) δ 8.55 (s, 1H), 8.09 (d, *J* = 8.5, 1H), 7.80 - 7.74 (m, 2H), 7.49 - 7.41 (m, 1H), 7.40 - 7.33 (m, 4H), 7.32 - 7.26 (m, 1H), 6.09 (q, *J* = 6.9 Hz, 1H), 3.03 (s, 3H), 1.67 (d, *J* = 6.9 Hz, 3H); ^{13}C NMR (100 MHz, DMSO-d_6) δ 162.7, 153.4, 151.8, 140.6, 132.4, 128.5 (2), 127.8, 127.1, 127.0 (2C), 125.7, 124.8, 115.6, 56.5, 34.4, 16.3; IR (neat, cm^{-1}): 2971, 2867, 1504, 1393, 1345, 1066, 1019, 766, 686; HRMS (ACPI/ASAP, m/z): found 264.1498, calcd for $C_{17}H_{18}N_3$, $[M+H]^+$, 264.1501,

***N*-(1-Phenylethyl)quinazolin-4-amine (S-78)**

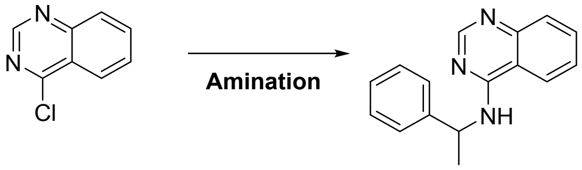

4-Chloroquinazoline (202 mg, 1.22 mmol) and (*Rac*)-1-phenylethan-1-amine (215 mg, 1.77 mmol) was added *n*-BuOH (5 mL) and agitated at 145 °C for 24 h. The solution was cooled to 22 °C and water (20 mL) was added. The reaction mixture was extracted with EtOAc (3 × 30 mL), dried over Na_2SO_4 and concentrated *in vacuo*. Column chromatography on silica-gel (EtOAc, R_f = 0.30) gave 246 mg (0.99 mmol, 81%) of a white solid, mp. 164 - 166 °C (lit.[13] 106 - 108 °C); ^{1}H NMR (400 MHz, DMSO-d_6) δ 8.48 - 8.48 (m, 2H), 8.41 (s, 1H), 7.80 - 7.74 (m, 1H), 7.69 - 7.66 (m, 1H), 7.56 - 7.51 (m, 1H), 7.45 - 7.43 (m, 2H), 7.33 - 7.28 (m, 2H), 7.22 - 7.18 (m, 1H), 5.61 (m, 1H), 1.59 (d, *J* = 7.3 Hz, 3H). ^{1}H NMR corresponded with literature values when conducted in $CDCl_3$[13].

***N*-(2,2,2-Trifluoro-1-phenylethyl)quinazolin-4-amine (S-79)**

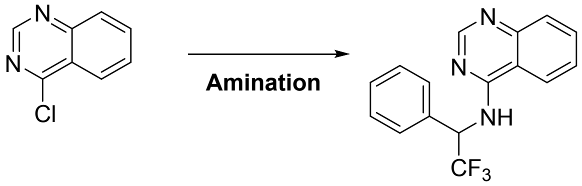

4-Chloroquinazoline (102 mg, 0.620 mmol) was mixed with 2,2,2-trifluoro-1-phenylethan-1-amine (327 mg, 1.87 mmol) in *n*-BuOH (2 mL). The mixture was heated under a N_2 atmosphere for 5 h at reflux. The reaction mixture was concentrated, diluted with water (20 mL), extracted with CH_2Cl_2 (3 × 20 mL) and dried over Na_2SO_4. Purification by silica-gel column chromatography (EtOAc, R_f = 0.62) gave 124 mg (0.41 mmol, 66%) of a white solid; mp. 155 - 156 °C; ^{1}H NMR (400 MHz, DMSO-d_6) δ 8.88 (d, *J* = 9.4 Hz, 1H), 8.69 (d, *J* = 8.5 Hz, 1H), 8.58 (s, 1H), 7.89 - 7.82 (m, 1H), 7.81 - 7.73 (m, 3H), 7.66 - 7.59 (m, 1H), 7.50 – 7.37 (m, 3H), 7.74 - 6.62 (m, 1H); ^{13}C NMR (101 MHz, DMSO-d_6) δ 159.0, 154.2, 149.5, 133.3, 133.2, 129.0, 128.6 (4C), 127.7, 126.2, 125.0 (q, *J* = 283.1 Hz), 123.3, 114.6, 54.3 (q, *J* = 30.8 Hz). HRMS (ES, m/z): found 304.1064, calcd for $C_{16}H_{13}N_3F_3$, $[M+H]^+$, 304.1062.

12. References

[1] N.G.M. Davies, H. Browne, B. Davis, M.J. Drysdale, N. Foloppe, S. Geoffrey, B. Gibbons, T. Hart, R. Hubbard, M.R. Jensen, H. Mansell, A. Massey, N. Matassova, J.D. Moore, J. Murray, R. Pratt, S. Ray, A. Robertson, S.D. Roughley, J. Schoepfer, K. Scriven, H. Simmonite, S. Stokes, A. Surgenor, P. Webb, M. Wood, L. Wright, P. Brough, Targeting conserved water molecules: Design of 4-aryl-5-cyanopyrrolo[2,3-d]pyrimidine Hsp90 inhibitors using fragment-based screening and structure-based optimization, Bioorg. Med. Chem., 2012, **20**, 6770-6789, https://doi.org/10.1016/j.bmc.2012.08.050

[2] J. Han, S. Henriksen, K.G. Nørsett, E. Sundby, B.H. Hoff, Balancing potency, metabolic stability and permeability in pyrrolopyrimidine-based EGFR inhibitors, Eur. J. Med. Chem., 2016, **124**, 583-607, http://dx.doi.org/10.1016/j.ejmech.2016.08.068

[3] S.J. Kaspersen, C. Sørum, V. Willassen, E. Fuglseth, E. Kjøbli, G. Bjørkøy, E. Sundby, B.H. Hoff, Synthesis and in vitro EGFR (ErbB1) tyrosine kinase inhibitory activity of 4-N-substituted 6-aryl-7H-pyrrolo[2,3-d]pyrimidine-4-amines, Eur. J. Med. Chem, 2011, **46**, 6002-6014, https://doi:10.1016/j.ejmech.2011.10.012

[4] F.H. Blindheim, A.T. Malme, B. Dalhus, E. Sundby, B.H. Hoff, Synthesis and Evaluation of Fused Pyrimidines as E. coli Thymidylate Monophosphate Kinase Inhibitors, ChemistrySelect, 2021, **6**, 12852-12857, https://doi.org/10.1002/slct.202103796

[5] S.J. Kaspersen, J. Han, K.G. Nørsett, L. Rydså, E.B. Kjøbli, S., G. Bjørkøy, E. Sundby, B.H. Hoff, Identification of new 4-*N*-substituted 6-aryl-7*H*-pyrrolo[2,3-*d*]pyrimidine-4-amines as highly potent EGFR-TK inhibitors with Src-family activity, Eur. J. Pharm. Sci., 2014, **59**, 69-82, http://dx.doi.org/10.1016/j.ejps.2014.04.011

[6] S.J. Kaspersen, E. Sundby, C. Charnock, B.H. Hoff, Activity of 6-aryl-pyrrolo[2,3-*d*]pyrimidine-4-amines to *Tetrahymena*, Bioorg. Chem., 2012, **44**, 35-41, http://dx.doi.org/10.1016/j.bioorg.2012.06.003

[7] A.C. Reiersølmoen, J. Han, E. Sundby, B.H. Hoff, Identification of fused pyrimidines as interleukin 17 secretion inhibitors, Eur. J. Med. Chem., 2018, **155**, 562-578, https://doi.org/10.1016/j.ejmech.2018.06.019

[8] A.C. Reiersølmoen, T.I. Aarhus, S. Eckelt, K.G. Nørsett, E. Sundby, B.H. Hoff, Potent and selective EGFR inhibitors based on 5-aryl-7H-pyrrolopyrimidin-4-amines, Bioorg. Chem., 2019, **88**, 102918,

[9] S. Bugge, S.J. Kaspersen, E. Sundby, B.H. Hoff, Route selection in the synthesis of C-4 and C-6 substituted thienopyrimidines, Tetrahedron, 2012, **68**, 9226-9233, doi:10.1016/j.tet.2012.08.090

[10] S. Bugge, S.J. Kaspersen, S. Larsen, U. Nonstad, G. Bjørkøy, E. Sundby, B.H. Hoff, Structure-activity study leading to identification of a highly active thienopyrimidine based EGFR inhibitor, Eur. J. Med. Chem., 2014, **75**, 354-374, doi: 10.1016/j.ejmech.2014.01.042

[11] S. Bugge, A.F. Buene, N. Jurisch-Yaksi, I.U. Moen, E.M. Skjoensfjell, E. Sundby, B.H. Hoff, Extended structure-activity study of thienopyrimidine-based EGFR inhibitors with evaluation of drug-like properties, Eur. J. Med. Chem., 2016, **107**, 255-274, 10.1016/j.ejmech.2015.11.012

[12] J. Han, S.J. Kaspersen, S. Nervik, K.G. Nørsett, E. Sundby, B.H. Hoff, Chiral 6-aryl-furo[2,3-d]pyrimidin-4-amines as EGFR inhibitors, Eur. J. Med. Chem, 2016, **119**, 2778-2799,

[13] Z. Shen, X. He, J. Dai, W. Mo, B. Hu, N. Sun, X. Hu, An efficient HCCP-mediated direct amination of quinazolin-4(3H)-ones, Tetrahedron, 2011, **67**, 1665-1672,
